# Management strategies and outcomes of basilar trunk aneurysms: a systematic review and meta-analysis

**DOI:** 10.1007/s00423-025-03959-3

**Published:** 2026-01-20

**Authors:** Zhichao Tian, Feng Gu, Bohan Li, Jiahao Meng, Xinyu Tao, Guannan Jiang, Ronghui Fu, Zhong Wang, Wanchun You

**Affiliations:** Department of Neurosurgery & Brain and Nerve Research Laboratory, The First Affiliated Hospital of Soochow University, 188 Shizi Street, Suzhou, 215006 Jiangsu Province China

**Keywords:** Basilar trunk aneurysms, Endovascular treatments, Open surgery, Outcomes, Meta-analysis

## Abstract

**Background:**

Due to the complex anatomical structure surrounding the basilar artery trunk, basilar trunk aneurysms (BTAs) can result in severe complications and poor prognosis for patients. The treatments for BTAs still remain challenging and uncertain.

**Methods:**

We conducted a comprehensive search of Embase, MEDLINE, Cochrane Library databases using medical subject headings and free-text terms, with the last search completed on July 1st, 2024. Both single-arm and two-arm meta-analysis were performed to compare the safety and effectiveness of different treatments for BTAs. Both fixed-effects models and random-effects models were calculated. When the heterogeneity was over 50%, we chose the random-effects model.

**Results:**

We identified 21 studies enrolling 593 participants and 599 aneurysms. The summary favorable outcome proportion was 0.46 (95% CI: 0.303 to 0.625) for open surgery and 0.75 (95% CI: 0.671 to 0.819) for endovascular treatments in the random-effects model, as the I^2^ for heterogeneity was 66% (*P* < 0.01) for open surgery and 53% for endovascular treatments (*P* < 0.01). Significant differences were observed between the two subgroups in the single-arm meta-analysis (*P* < 0.01). In the direct comparisons of good outcomes between open surgery and endovascular treatments in BTAs, no statistically significant difference was observed. The relative risk (RR) was 0.82 (95% CI: 0.549 to 1.224) and the P value was 0.5226. The comparison revealed no statistically significant changes in mortality, complications and complete occlusion (*P* > 0.05).

**Conclusion:**

No statistically significant difference was observed between the open surgery and endovascular treatments. The mortality and complication outcomes revealed no distinctions between the two subgroups, regardless of whether direct or indirect comparisons was conducted. The influence of institutional expertise emerged as a critical factor in treatment outcomes. Furthermore, more effective controls and larger sample size are required to achieve more credible and conclusive results.

**Supplementary Information:**

The online version contains supplementary material available at 10.1007/s00423-025-03959-3.

## Introduction

Intracranial aneurysm is a life-threatening cerebrovascular disorder with a prevalence of approximately 3%.^1^ They are characterized by abnormal dilatation of the intracranial arteries, which markedly increases the risk of mortality and long-term disability [1, 2]. Basilar trunk aneurysms (BTAs) refer to aneurysms that are located from the basilar origin to the origin of the superior cerebellar artery [3, 4]. Owing to the complex neurovascular anatomy surrounding the basilar trunk, ruptured BTAs frequently lead to severe neurological complications and poor clinical outcomes. Moreover, even in the absence of rupture, acute dissecting or chronic dolichoectatic aneurysms may cause mass effects or ischemic events, leading to serious outcomes. These anatomical and pathophysiological challenges also complicate management, making BTAs particularly difficult to treat safely and effectively.

Research on this challenging and intractable condition has progressed for decades, yet optimal management remains elusive. Traditional clipping surgeries can address some regular saccular BTAs but are limited in treating large and fusiform aneurysms [5, 6]. Endovascular treatments also present many complications, particularly concerning perforator vessel protection [7, 8]. The application of flow diversion in BTAs has also remained controversial in recent years [9, 10]. For exceptionally complex aneurysms, recent advances in bypass and revascularization techniques have demonstrated potential benefits [11]. Despite these developments, the management of BTAs continues to be associated with considerable uncertainty [12]. Notably, no comprehensive synthesis of treatment-specific outcomes or comparisons across major management strategies has been available to guide clinical decision-making. To address this gap, we conducted a systematic review and meta-analysis to summarize current evidence and evaluate the effectiveness of different treatment modalities for BTAs.

## Methods

**Fig. 1:**
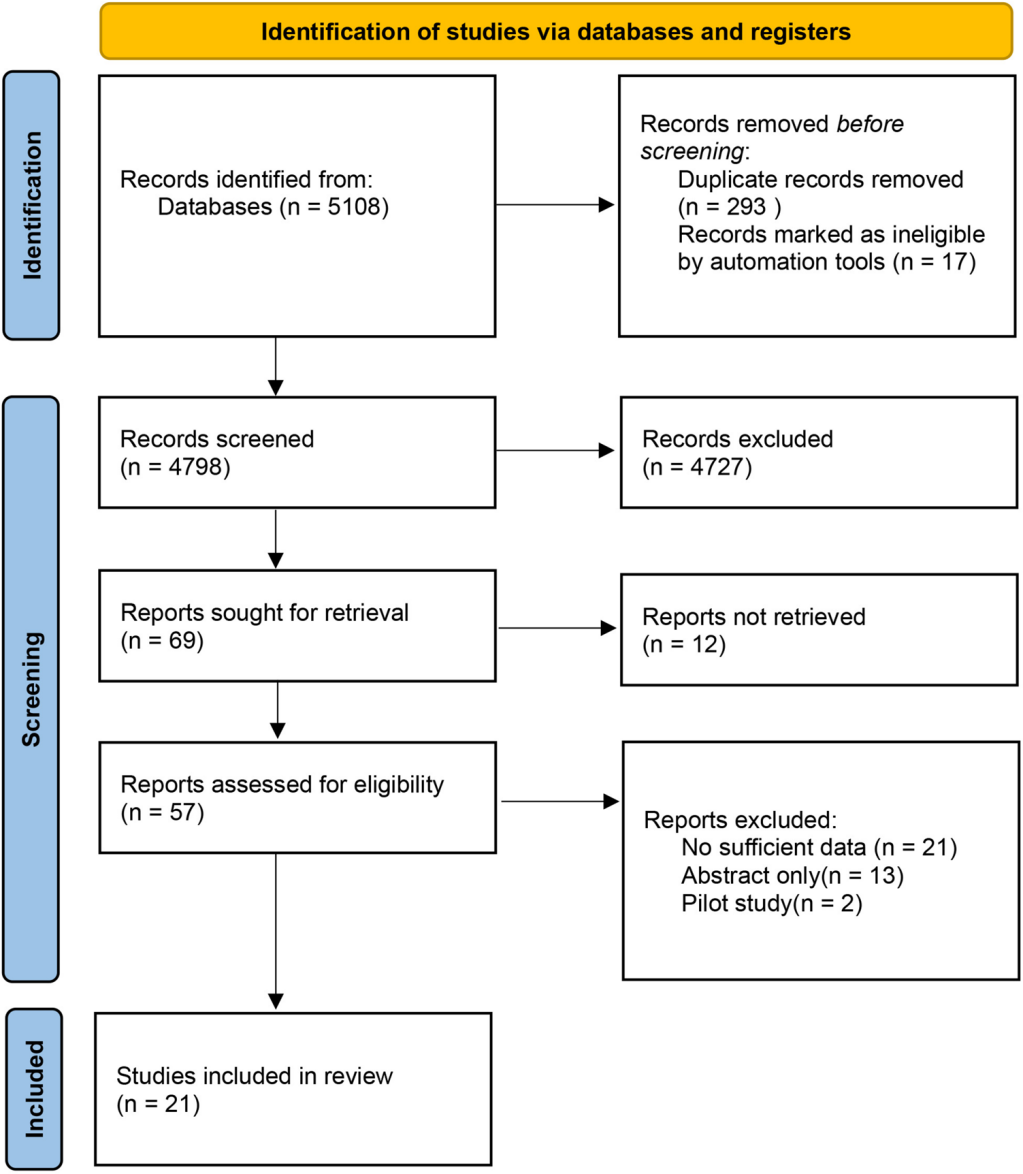
PRISMA flow diagram

This systematic review and meta-analysis was conducted in accordance with the Preferred Reporting Items for Systematic Reviews and Meta-Analyses (PRISMA) guidelines [13]. A PRISMA flow diagram (Fig. 1) was generated to illustrate the entire study selection process. The review protocol was not prospectively registered.

### Literature search

A systematic literature search was conducted in Embase, MEDLINE, Cochrane Library databases, with the last search completed on July 1st, 2024. Both Medical Subject Headings (MeSH) and free-text terms were employed to ensure comprehensive coverage. A comprehensive literature search was performed in each database using a combination of Medical Subject Headings (MeSH) and keywords. Boolean operators “OR” and “AND” were applied to expand and refine the search scope. The search strategy included the following terms: (“Basilar artery” OR “vertebrobasilar” OR “posterior circulation” OR “dolichoectatic” OR “basilar trunk artery”) AND (“aneurysm”). Moreover, we searched the reference lists of included studies.

### Selecting criteria

#### Inclusion criteria

(1) at least ten patients with BTAs confirmed by CTA, MRA or DSA were included in the study; (2) all patients with BTAs received open surgery or endovascular treatment; (3) sufficient results for single proportions or binary outcome meta-analysis;

#### Exclusion criteria

(1) abstract only; (2) not written in English; (3) letters; (4) pilot study; (5) case-control study.

Two authors independently performed the study selection process. Initially, titles and abstracts were screened to identify potentially eligible studies. Full texts of these studies were then retrieved and assessed for inclusion based on predefined criteria. Any discrepancies between the two reviewers were resolved through discussion with a senior author.

### Data extraction

Data extraction was independently performed by two authors and subsequently verified by a third author for accuracy. Extracted information included study author and year of publication, sample size, mean patient age, sex distribution, number of aneurysms, mean aneurysm size, aneurysm morphology, number of ruptured aneurysms, details of surgical or endovascular techniques, outcome assessment measures, and mean follow-up duration.

### Primary outcomes

Patient outcomes were evaluated using widely accepted measures, including the modified Rankin Scale (mRS) and the Glasgow Outcome Scale (GOS). In most included studies, a GOS score of 4–5 or an mRS score of 0–2 was considered as a favorable outcome. For the purposes of our meta-analysis, we identified the mRS 0–2 as a favorable outcome. Whenever possible, outcome assessments were based on uniform follow-up periods within each study, with preference given to longer-term follow-up data to better capture sustained clinical effects.

### Secondary outcomes

Secondary outcomes included mortality, treatment-related complications, and complete aneurysm occlusion rate. And outcome assessments were based on uniform follow-up periods within each study, with preference given to long-term follow-up to more accurately reflect sustained treatment effects.

### Quality assessment

The methodological quality of all included studies was assessed using the Newcastle-Ottawa Scale (NOS) [14]. Two authors independently evaluated each study across three domains: selection of study participants, comparability of study groups, and assessment of exposure or outcomes. Studies scoring more than 6 points were considered high quality. The potential reporting bias in our meta-analysis was assessed by the funnel plots, with asymmetry indicating reporting bias.

### Statistical analysis

For the single-arm meta-analysis, proportions of primary and secondary outcomes were calculated along with 95% confidence intervals (CIs). Binary outcomes were used to calculate risk ratios (RRs) and corresponding 95% CIs for comparing different treatment modalities. Both fixed-effects models and random-effects models were calculated. The heterogeneity of the meta-analysis was evaluated by the Cochran Q-test and I^2^ index [15], with I^2^ values exceeding 25%,50%, and 75% representing low, moderate, and high heterogeneity, respectively [16]. When the heterogeneity was over 50%, we chose the random-effects model. All statistical analyses were performed using R software version 4.1.1, with the "metaprop" and "metabin" packages. Shapiro-Wilk test [17] was used to assess data normality. A *P*-value < 0.05 was considered significant for all analyses, and tests are two-tailed.

## Results

### Literature search and inclusion

A total of 5,108 records were identified through the systematic search of MEDLINE, Embase, and the Cochrane Library, based on the predefined search strategy. The detailed search strategy is presented in Supplementary Material. Following title and abstract screening, 69 studies were selected for full-text review, of which 12 could not be retrieved. Ultimately, 21 studies met the inclusion criteria and were included in the analysis. The detailed process is illustrated in Fig. 1.

### Research characteristics

All 21 studies were retrospective cohort studies. Among them, 5 studies contained both endovascular treatment and open surgery for BTAs, while the remaining studies used only one of the two treatments. Specifically, there were 5 studies on open surgery and 11 studies on endovascular treatment. Overall, 593 patients with 599 aneurysms were included, with five patients presenting with multiple aneurysms. The detailed characteristics can be found in Table 1. Complications are summarized in Table 2.

**Table 1:** Characteristics of including studies

Authors, Year	Number of objects	Mean aneurysm size (mm)	Number of ruptured aneurysms (%)	Surgical treatment(cases)	Outcomes
Cho et al. [28]	15	6.4	7(43.75%)	Stents(15)	mRS, angiographic follow-up, complications, mortality
Higa et al. [32]	22	9.3	19(86.36%	Clipping(11), Coiling(11)	angiographic follow-up, complications, mortality
Kalani et al. [40]	11	NA	0(0%)	Bypass + Clipping(7), Bypass + endovascular treatment(4)	mRS, angiographic follow-up, complications, mortality
Lawton et al. [11]	37	17	NA	Bypass(16)	mRS, angiographic follow-up, complications, mortality
Mu et al. [34]	21	> 12	1(4.8%)	Stents(10), SAC(11)	mRS, angiographic follow-up, complications, mortality
Nakatomi et al. [6]	32	27.6	2(6.25%)	IPO(7), RPO(6), Clipping(5), Bypass(3)	mRS, angiographic follow-up, complications, mortality
Pandey et al. [24]	23	6.2	13(56.52%)	Coiling(23)	GOS, angiographic follow-up, complications, mortality
Peerless et al. [35]	23	NA	NA	Clipping(23)	angiographic follow-up, complications, mortality
Qu et al. [29]	14	20.1	NA	Stents(2), SAC(3), PAO(3), LVA(1),coiling(5)	GOS, angiographic follow-up, complications, mortality
Saliou et al. [4]	52	10.6	8(15.38%)	Stents, SAC, Coiling, Bypass, FD, Trapping, Flow reversal*	mRS, angiographic follow-up, complications, mortality
Seifert et al. [19]	24	NA	22(91.67%)	Clipping(24)	angiographic follow-up, complications, mortality
Sim et al. [10]	40	10.6	27(67.50%)	Coiling(9), SAC(17), Stents(6), FD(5), vertebral artery occlusion(3)	GOS, angiographic follow-up, complications, mortality
Tjahjadi et al. [20]	14	5.5	19(100%)	Clipping(14)	mRS, GOS, angiographic follow-up, complications, mortality
Uda and Duckwiler [30]	39	NA	27(65.85%)	Coiling(39)	GOS, angiographic follow-up, complications, mortality
Van Oel et al. [21]	13	21	3(23.08%)	Stents, Coiling, FD*	GOS, angiographic follow-up, complications, mortality
Wallace et al. [31]	12	8.9	3(23.08%)	PED, PED + Coiling*	mRS, angiographic follow-up, complications, mortality
Wang et al. [36]	28	7.5	10(35.71%)	SAC(28)	mRS, angiographic follow-up, complications, mortality
Wu et al. [25]	34	14.5	3(8.82%)	FD(13), Stents(11), SAC(10)	mRS, angiographic follow-up, complications, mortality
Yu et al. [22]	16	NA	15(93.75%)	Coiling(8), Balloon-assisted coil(2), SAC(2), Coiling + PAO(2)	GOS, angiographic follow-up, complications, mortality
Zhang et al. [26]	12	NA	NA	Bypass(7), Stents(4)	mRS, angiographic follow-up, complications, mortality
Zhong et al. [27]	111	10.2	26(23.42%)	BA + Coiling(7), BA + Stents(4), BA + FD(1), BA(99)	mRS, angiographic follow-up, complications, mortality
Summary	593	> 12.35	/	/	/

*SAC* Stent-assisted coiling; *IPO* Immediately proximal parent artery occlusion; *RPO* Remotely proximal parent artery occlusion; *FD *Flow diverter; *PED* Pipeline embolization device; *GOS* Glasgow outcome scale; *mRS* Modified rankin scale; *BA* Balloon angioplasty

* The statistical data are not available in several articles

**Table 2:** Complications of including studies

Variables	Number
Open surgery	
Clipping	77
Bypass	37
Intervention	
Stents	52
Coiling	97
SAC	68
FD	19
Complications	
Open surgery	
Hemorrhage	1
Ischemia	12
Thrombosis	0
Rebleeding	1
Stenosis	0
Myocardial infarction	0
Pneumonia	2
Hydrocephalus	1
Intraoperative rupture	4
Catheter-induced spasm	0
Stent misplaced	0
Fractured PED delivery wire	0
Intervention	
Hemorrhage	2
Ischemia	22
Thrombosis	5
Rebleeding	3
Stenosis	1
Myocardial infarction	1
Pneumonia	5
Hydrocephalus	1
Intraoperative rupture	3
Catheter-induced spasm	1
Stent misplaced	0
Fractured PED delivery wire	1

*SAC* Stent-assisted coiling; *FD* Flow diverter; *PED* Pipeline embolization device

### Quality assessment

Based on the Newcastle–Ottawa Scale (NOS) assessment, the overall methodological quality of the included studies ranged from moderate to high. Most studies clearly described their selection criteria, comparability between groups, and outcome assessment. The general quality of evidence supports the reliability of the pooled findings in this meta-analysis. The inclusion criteria for study subjects were unclear in six studies [6, 18–22]. Most studies provided adequate follow-up to evaluate patient prognosis. Further details on the quality assessment are presented in Table 3. The potential reporting bias in our meta-analysis was evaluated using funnel plots, as presented in Figure S1. Inspection of the funnel plots revealed no evident asymmetry, suggesting a low likelihood of significant publication bias across the included studies.

**Table 3:** Newcastle–Ottawa quality assessments scale

Study	Selection				Comparability	Outcome			Score
	Representativeness of the exposed cohort	Selection of the non exposed cohort	Ascertainment of exposure	Demonstration that outcome of interest was not present at start of study	Comparability of cohorts on the basis of the design or analysis	Assessment of outcome	Was follow-up long enough for outcomes to occur	Adequacy of follow up of cohorts	
Cho et al. [28]	*	-	**	-	*	*	*	*	7
Higa et al. [32]	*	-	**		*	*	*	*	7
Kalani et al. [33]	*	-	**	-	*	*	*	*	7
Lawton et al. [11]	*	-	**	*	*	**	*	*	9
Mu et al. [34]	**-**	-	**	-	**	*	*	*	7
Nakatomi et al. [6]	-	-	**	-	*	*	*	*	6
Pandey et al. [24]	*	-	**	-	*	**	*	*	8
Peerless et al. [35]	*	-	*	-	*	*	-	*	5
Qu et al. [29]	*	-	**	-	*	*	-	*	6
Saliou et al. [4]	*	-	**	-	*	*	*	*	7
Seifert et al. [19]	-	-	**	-	*	*	*	*	6
Sim et al. [10]	*	-	**	*	*	*	*	*	8
Tjahjadi et al. [20]	-	-	**	-	*	**	-	*	6
Uda and Duckwiler [30]	*	-	**	-	*	*	*	*	7
Van Oel et al. [21]	-	-	**	-	*	*	*	*	6
Wallace et al. [31]	*	-	**	-	**	*	-	*	7
Wang et al. [36]	*	-	**	-	*	*	*	*	7
Wu et al. [25]	*	-	**	-	*	*	*	*	7
Yu et al. [22	-	-	**	-	*	*	*	*	6
Zhang et al. [26]	*	-	**	-	*	*	*	*	7
Zhong et al. [27]	*	-	**	-	*	*	*	*	7

### Primary outcome

**Fig. 2:**
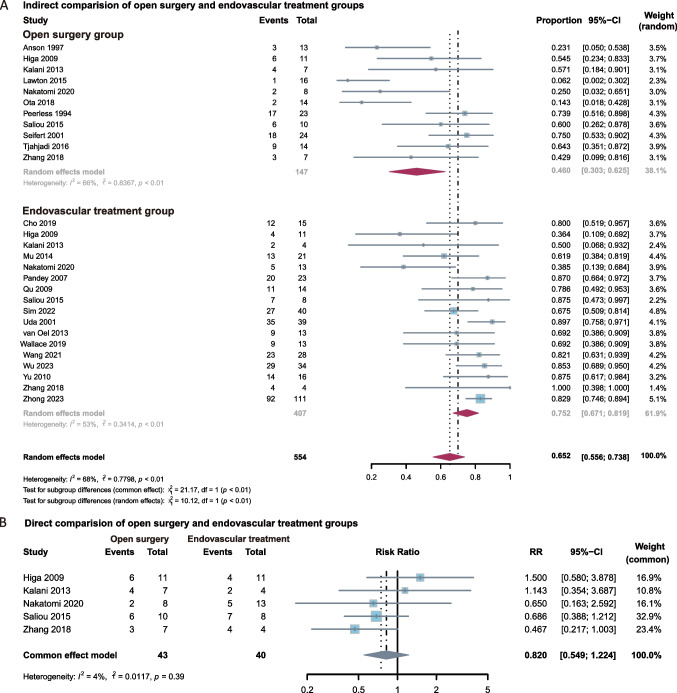
**A**, Forest plot of indirect comparisons evaluating favorable outcomes between open surgery and endovascular treatment for BTAs. **B**, Forest plot of direct comparisons evaluating favorable outcomes between open surgery and endovascular treatment for BTAs

In the single-arm random-effects meta-analysis, the summary proportion of favorable outcomes was 0.46 (95% CI: 0.303–0.625) for open surgery and 0.75 (95% CI: 0.671–0.819) for endovascular treatments, with heterogeneity I^2^ values of 66% (*P* < 0.01) and 53% (*P* < 0.01), respectively (Fig. 2A). The overall summary proportion of favorable outcomes across all studies was 0.65 (95% CI: 0.556–0.738). Significant differences were observed between the two subgroups in the single-arm analysis (*P*<0.01). A leave-one-out sensitivity analysis was conducted and demonstrated that the results were robust and minimally influenced by any individual study (Figure S2). In the direct comparison of binary outcome meta-analysis, contrary to the single-arm results, no obvious difference was found between the endovascular treatment group and the open surgery group. The I^2^ for heterogeneity was 4% (*P *= 0.39) so we chose the fixed-effects model. The RR was 0.82 (95% CI: 0.549 to 1.224) and the P value was 0.5226 (Fig. 2B). However, several studies defined mRS 0–3 as a good outcome [6, 18, 23–27]. To maintain consistency and facilitate comparison, we followed the definitions provided by the original studies.

### Secondary outcome

All 21 included studies reported mortality outcomes. Treatment-related complications at discharge or during follow-up were documented in 13 studies, and nine studies reported complete aneurysm occlusion rates [10, 20, 24, 25, 27–31]. 

**Fig. 3:**
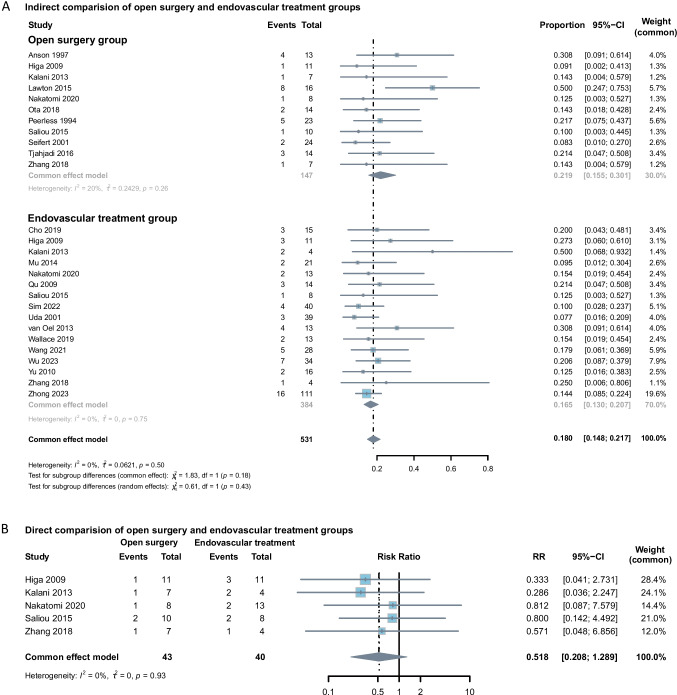
**A**, Forest plot of indirect comparisons assessing mortality between open surgery and endovascular treatment for BTAs. **B**, Forest plot of direct comparisons assessing mortality between open surgery and endovascular treatment for BTAs

The summary mortality was estimated as 0.219 (95% CI: 0.155 to 0.301) for the open surgery group and 0.165 (95% CI: 0.130 to 0.207) for the endovascular treatment group. Heterogeneity was low for both groups (*P* = 0.26 for the open surgery group and *P* = 0.75 for the endovascular treatment group; Fig. 3). No obvious difference between endovascular treatment and open surgery groups was found in either single-arm meta-analysis or binary outcome meta-analysis (*P* = 0.18 for single-arm and *P* = 0.16 for binary outcome.

**Fig. 4:**
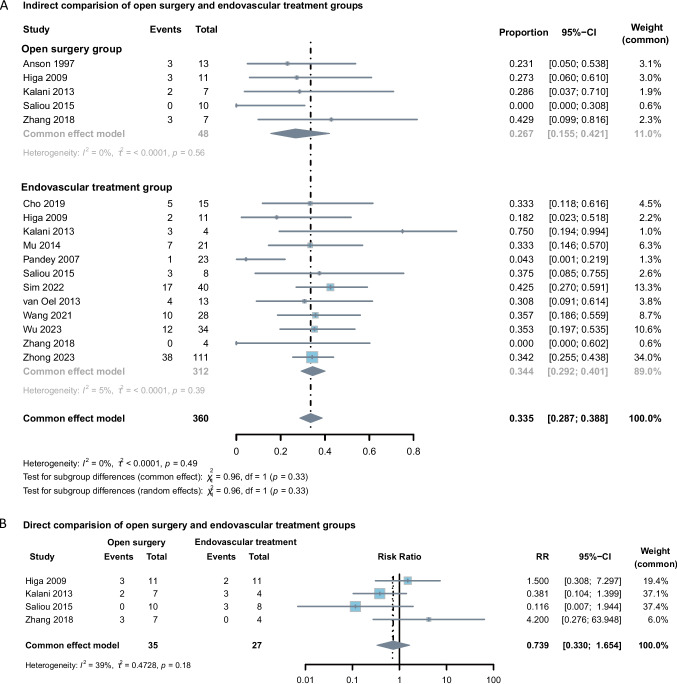
**A**, Forest plot of indirect comparisons evaluating treatment-related complications between open surgery and endovascular treatment for patients with BTAs. **B**, Forest plot of direct comparisons evaluating treatment-related complications between open surgery and endovascular treatment for patients with BTAs

**Fig. 5:**
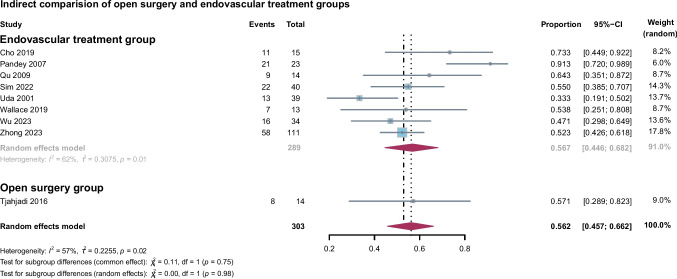
Forest plot of comparing complete versus incomplete occlusion rates between open surgery and endovascular treatment for BTAs

In the single-arm analysis, no obvious difference between the endovascular treatment and open surgery group was found in terms of both complication proportions (*P* = 0.33 for the fixed effects model; Fig. 4) and complete occlusion rates (*P* = 0.75 for the fixed effects model; Fig. 5). Direct comparisons confirmed these findings, showing no significant differences between the two groups (*P* = 0.46).

## Discussion

In the present study, we systematically reviewed the literature and performed a single-arm meta-analysis including 593 patients from 21 studies. To our knowledge, this is the first meta-analysis directly comparing endovascular treatment and open surgery for BTAs. The results of the single-arm analysis suggested that endovascular treatment tended to have higher favorable outcome proportions compared to open surgery. However, in the two-arm meta-analysis of studies including both treatment groups, no significant differences were observed. Regarding mortality, treatment-related complications and complete occlusion rates, both single-arm and two-arm analyses found no significant difference between the two groups. The observed disparity in outcomes between single-modality and dual-modality centers is significant and may indicate underlying variations in treatment practices or institutional expertise. In institutions specializing in a single modality, patients are limited to one treatment option (either endovascular treatment or open surgery). Conversely, treatment decisions are influenced not only by the specific characteristics of the aneurysm but also by the practitioner's expertise, which may introduce bias. In contrast, dual-modality centers likely benefit from multidisciplinary decision-making, enabling a comprehensive evaluation of each patient based on aneurysm characteristics, patient condition and relevant factors. This approach facilitates personalized treatment strategies that optimize patient outcomes. Patients presenting with complex aneurysms may derive significant benefits from high-volume, dual-modality centers, where treatment decisions can be collaboratively formulated by experienced neurosurgeons. The institutional expertise may influence patients’ outcome.

BTAs are associated with a high risk of rupture and growth, leading to high mortality and disability rates [4, 6, 37]. Even unruptured aneurysms can cause significant neurological dysfunction due to the mass effect on critical anatomical structures around the BTA, including the brainstem and cranial nerves. Additionally, high rates of acute dissecting aneurysms caused fatal ischemic events [4]. Therefore, timely intervention is generally recommended once BTAs are identified. However, treatment carries risks, including the potential for permanent neurological deficits or death. In our study, regardless of treatment methods, the summary proportion of favorable outcomes was relatively low which accounted for 65.2%, while the summary mortality reached 18.1%. Meanwhile, the specific parent artery, which primarily supplies the brainstem with important branch arteries and many perforator arteries, presents special vessel-protection requirements to the treatment of BTAs. Consequently, treatment-related complications such as cranial nerve injury and ischemic or hemorrhagic events are frequently reported [25, 27]. In our meta-analysis, the summary complication rate was up to 33.5% with low heterogeneity. The complete occlusion rate was also unsatisfactory, with a summary rate of 56.2% in our study. The immediate and follow-up complete occlusion rates of aneurysms are independent risk factors for the outcome [27]. Large, fusiform, and dolichoectatic morphologies are common in this region, making it difficult to fully exclude the aneurysm while preserving critical branch and perforating arteries that arise from the parent vessel [10].

The management of BTAs remains uncertain and continues to be a subject of considerable debate despite ongoing advances in treatment strategies. The development of novel endovascular devices has positioned endovascular therapy as the first-line approach for posterior circulation aneurysms [27]. Currently, the most widely used treatment methods for BTAs are endovascular techniques, including flow diversion, stent-assisted coil embolization, and covered stents [38, 39]. However, the early outcomes of endovascular treatment for large and dolichoectatic BTAs have been less favorable than those observed in other aneurysm types, largely due to the frequent involvement of perforator arteries [27, 40–42]. Although two recent studies have demonstrated that coils, stents, and flow diversion can yield satisfactory rates of favorable outcomes, the associated mortality and complication rates remain high, particularly in large BTAs [10, 27]. Open surgical approaches—including clipping, bypass, and trapping—represent another major treatment option for large BTAs. Yet, these procedures are technically demanding, with key challenges involving adequate aneurysm exposure and the risk of injury to perforating vessels and cranial nerves [43]. Advances in bypass techniques have contributed to improved patient outcomes, although complication and mortality rates remain substantial [11, 44]. Combined open surgical approaches and endovascular strategies have also been described, but only in a limited number of cases [4, 26]. Overall, because of the high risk and complexity of treating BTAs, individualized treatment strategies should be developed using advanced techniques [11, 33, 43, 44].

In our review of the literature, few meta-analyses focused specifically on BTAs. One study investigated the natural history of vertebrobasilar dolichoectatic and fusiform aneurysms without analyzing treatment [4]. Sonmez et al. compared reconstructive and deconstructive endovascular treatments in vertebrobasilar dissecting aneurysms [39]. Another recent single-arm meta-analysis conducted by Bin-Alamer et al. has focused on different management strategies in dolichoectatic vertebrobasilar aneurysms [45]. Our study had a similar summary favorable outcome proportion at 65.2% compared to their previous outcome of 52%. This study reported that regardless of the treatment modality, patients exhibited a poor prognosis and high mortality, emphasizing the need for individualized patient management until more standardized guidelines become available. Consistent with other retrospective series, our findings also demonstrated no apparent advantage between the two treatment arms, and further highlighted the importance of center-specific expertise and multidisciplinary decision-making in optimizing individualized treatment selection and improving patient outcomes.

However, in our study, endovascular treatment tended to have better outcomes than open surgery in single-arm analysis, which was conflict with their results. The single-arm result also conflicted with the two-arm result in our analysis. Considering the relatively higher heterogeneity between studies and the potential bias of single-arm meta-analysis, we believe the two-arm meta-analysis result is more appropriate. The advantage of endovascular treatment in single-arm analyses likely reflects a combination of factors including selective inclusion of favorable cases, smaller sample sizes, and institutional or operator-specific biases. Single-arm studies are more prone to institutional practices, operator expertise, and patient selection criteria, which may lead to an overestimation of treatment efficacy. In contrast, two-arm comparative studies include a broader and more heterogeneous patient population, reducing selection bias and providing a more balanced assessment of treatment efficacy. In the two-arm analysis, the observed equivalence in outcomes between endovascular and microsurgical treatment of basilar artery aneurysms may reflect advances in both procedural techniques and perioperative management. The availability of cerebral revascularization techniques provides an important adjunct in the management of complex BTAs. Open microsurgery—particularly when combined with extracranial-to-intracranial or in situ bypass techniques—offers the unique advantage of securing cerebral perfusion in cases with complex hemodynamic compromise. Microsurgical approaches also have benefited from skull base techniques, intraoperative neurophysiological monitoring, which together reduce surgical complications. Endovascular therapy benefits from advanced endovascular devices, such as flow diverters and stent-assisted coiling and would reduce periprocedural complications. From a pathophysiological perspective, both strategies ultimately aim to achieve aneurysm exclusion from normal cerebral circulation and preserve critical perforating arteries. The convergence of these technical and biological factors may explain the comparable efficacy observed between the two treatments. Our analysis identified a discrepancy between the pooled results of indirect and direct comparisons concerning favorable outcomes. Indirect comparisons aggregate data from multiple independent studies, potentially introducing heterogeneity due to differences in baseline characteristics and treatment strategies. In contrast, direct comparisons are conducted within the same study population, thereby minimizing confounding variables associated with inter-study differences. Consequently, direct comparisons provide a more precise and reliable evaluation. The results for mortality and complications also showed that no significant variations were observed between the two subgroups, whether in direct comparison or indirect comparisons.

### Limitations

To the best of our knowledge, this is the first systematic review and meta-analysis focusing specifically on the BTAs. Furthermore, our study is the first two-arm meta-analysis on vertebrobasilar aneurysms. Nonetheless, several limitations should be acknowledged. First, given the low incidence of BTAs [6, 24], the sample sizes of included studies were not large which may introduce publication bias. Second, only five studies reported outcomes for both endovascular and open surgical treatments, limiting the statistical power and generalizability of the two-arm analysis. Although the heterogeneity of the five studies was low, potential bias could still exist in the direct comparison meta-analysis. The observed heterogeneity may partly reflect variations in aneurysm morphology, rupture status, inclusion of pediatric or dolichoectatic cases. These clinical and anatomical differences can influence treatment selection, procedural complexity, and outcomes. Finally, we were unable to perform subgroup analyses based on aneurysm size, morphology, rupture status, or preoperative conditions due to insufficient data. This study may be subject to selection bias, as treatment decisions are often influenced by the clinician’s experience and the specific characteristics of individual patients. Additionally, variations in treatment techniques across different institutions may affect the comparability of clinical outcomes. Due to limitations in the available data and case volume, most included studies did not report detailed information, which prevented us from performing a further analysis to verify the stability of results. Future large-sample or multicenter studies are needed to address these limitations.

## Conclusion

Overall, the evidence-based systematic review and meta-analysis suggest that no significant difference was observed between the open surgery and endovascular treatments for favorable outcomes, mortality, complication outcomes or complete occlusion, regardless of whether a direct or indirect comparison were conducted. The influence of institutional expertise emerged as a critical determinant of treatment outcomes. Furthermore, more effective controls and larger sample size are required to achieve more credible and conclusive results. Further investigations are anticipated.

## Supplementary Information

Below is the link to the electronic supplementary material.

Supplementary file1 (ZIP 55327 KB)

## Data Availability

The datasets used and analyzed during the current study are available from the corresponding author on reasonable request. All publicly available data is cited.
