# Supplementary material for: Management strategies and outcomes of basilar trunk aneurysms: a systematic review and meta-analysis
Source: Langenbecks Arch Surg. 2026 Jan 20;411(1):71. doi: 10.1007/s00423-025-03959-3 (PMC12852281; doi:10.1007/s00423-025-03959-3)
Supplement: Supplementary file 1 — Supplementary file1 (ZIP 55327 KB) [file 423_2025_3959_MOESM1_ESM.zip › Search strategy.docx]

A medical subject headings (MeSH) term and keyword search of each database was conducted using the Boolean operators “OR”and “AND”. The search terms used were as follows: “Basilar artery” OR “vertebrobasilar” OR “posterior circulation” OR “dolichoectatic” OR “basilar trunk artery” AND aneurysm.
