# Supplementary material for: Management strategies and outcomes of basilar trunk aneurysms: a systematic review and meta-analysis
Source: Langenbecks Arch Surg. 2026 Jan 20;411(1):71. doi: 10.1007/s00423-025-03959-3 (PMC12852281; doi:10.1007/s00423-025-03959-3)
Supplement: Supplementary file 1 — Supplementary file1 (ZIP 55327 KB) [file 423_2025_3959_MOESM1_ESM.zip › Supplementary figures.docx]

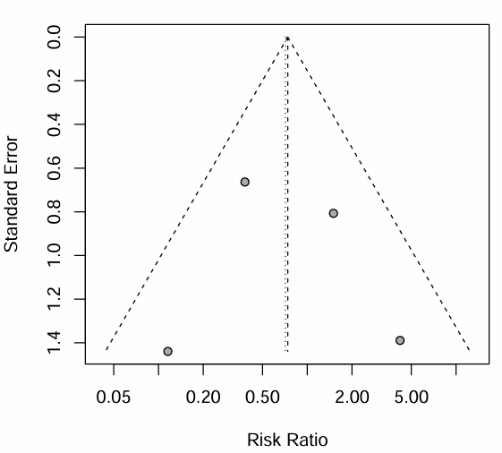

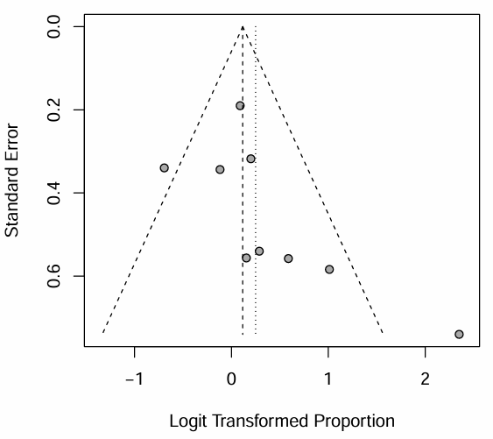

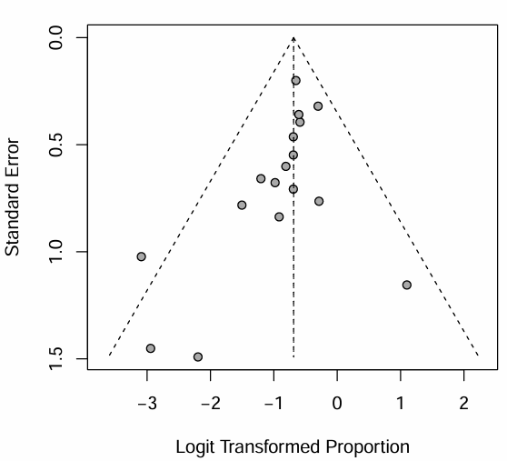

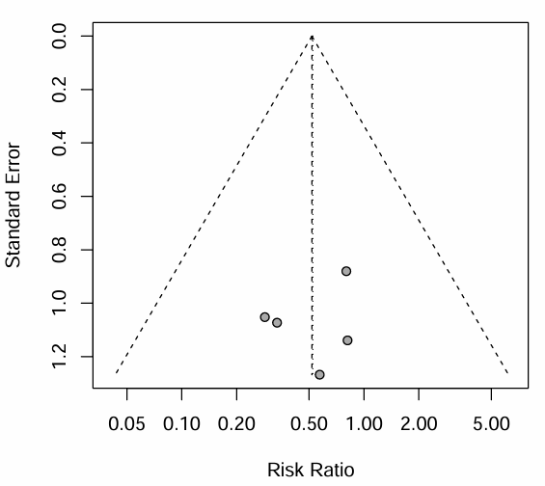

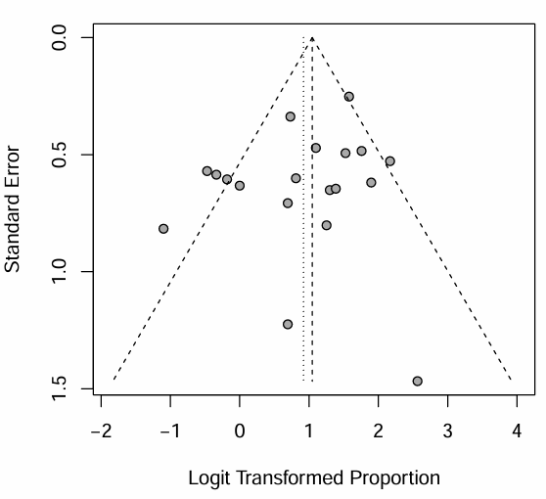

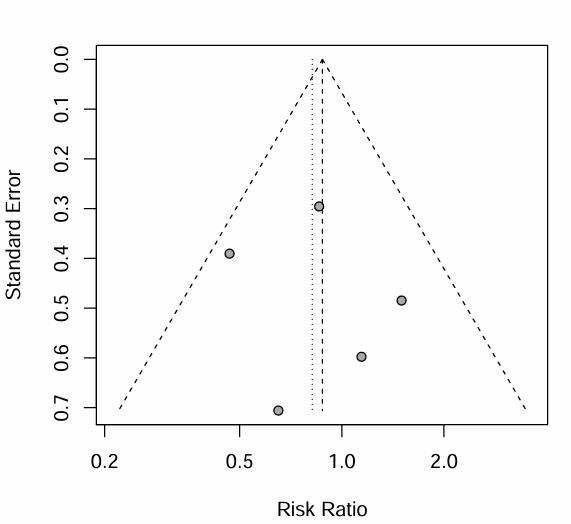

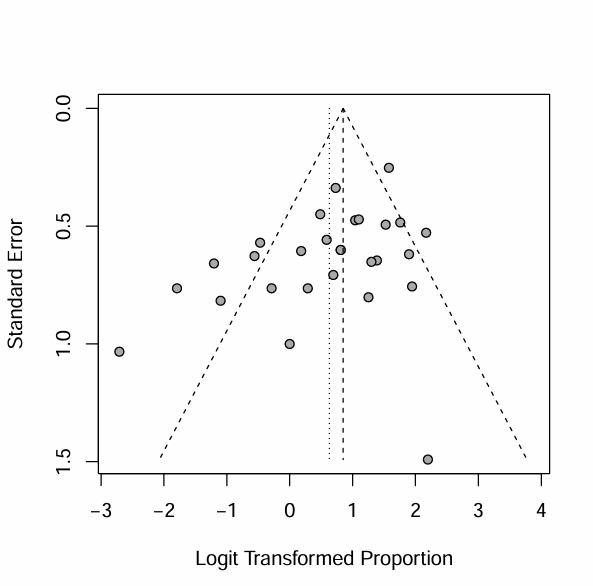
**Figure S1. Funnel plot of potential reporting bias in our meta-analysis**

**Figure S2. Leave-one-out sensitivity analysis.**

**
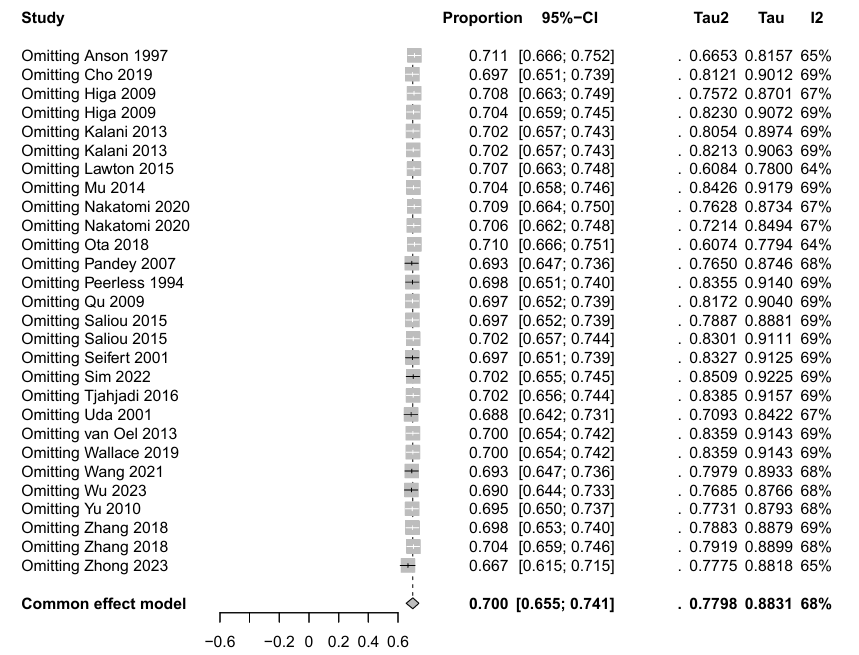
**
