# Supplementary material for: Management strategies and outcomes of basilar trunk aneurysms: a systematic review and meta-analysis
Source: Langenbecks Arch Surg. 2026 Jan 20;411(1):71. doi: 10.1007/s00423-025-03959-3 (PMC12852281; doi:10.1007/s00423-025-03959-3)
Supplement: Supplementary file 1 — Supplementary file1 (ZIP 55327 KB) [file 423_2025_3959_MOESM1_ESM.zip › Table S1.docx]

**Table S1. Characteristics of including studies.**

| Authors, Year | Number of objects | Mean age (years) | Sex (Men/Women) | Number of aneurysms | Mean aneurysm size (mm) | Morphology | Number of ruptured aneurysms(%) | Surgical Treatment(cases) | Outcomes | Mean  follow-up (months) |
| --- | --- | --- | --- | --- | --- | --- | --- | --- | --- | --- |
| Cho 2019 | 15 | 59.7 | 11/4 | 16 | 6.4 | 13 Saccular  2 Dissection  1 Fusiform | 7(43.75 %) | Stents(15) | mRS, angiographic follow-up, complications, mortality | 23.5 |
| Higa 2009 | 22 | 58.1 | 4/18 | 22 | 9.3 | 22 Saccular | 19(86.36% | Clipping(11), Coiling(11) | angiographic follow-up, complications, mortality | >6 |
| Kalani 2013 | 11 | 50.7 | 8/3 | 12 | NA | 5 Saccular  7 Fusiform | 0(0%) | Bypass+ Clipping(7), Bypass+ endovascular treatment(4) | mRS, angiographic follow-up, complications, mortality | 71.6 |
| Lawton 2015 | 37 | 63 | NA | 37 | 17 | 37 Dolichoectatic | NA | Bypass(16) | mRS, angiographic follow-up, complications, mortality | 44.4 |
| Mu 2014 | 21 | 46.5 | 20/1 | 21 | >12 | 11 Saccular  5 Dolichoectatic | 1(4.8%) | Stents(10), SAC(11) | mRS, angiographic follow-up, complications, mortality | 25.6 |
| Nakatomi 2020 | 32 | 56.8 | 20/12 | 32 | 27.6 | NA | 2(6.25%) | IPO(7), RPO(6), Clipping(5), Bypass(3) | mRS, angiographic follow-up, complications, mortality | 45.2 |
| Pandey 2007 | 23 | 47 | NA | 23 | 6.2 | NA | 13(56.52 %) | Coiling(23) | GOS, angiographic follow-up, complications, mortality | NA |
| Peerless 1994 | 23 | 46.8 | NA | 23 | NA | NA | NA | Clipping(23) | angiographic follow-up, complications, mortality | NA |
| Qu 2009 | 14 | 37 | 5/9 | 15 | 20.1 | 9 Saccular  6 Fusiform | NA | Stents(2), SAC(3), PAO(3), LVA(1),coiling(5) | GOS, angiographic follow-up, complications, mortality | 22.1 |
| Saliou 2015 | 52 | 56 | 23/29 | 52 | 10.6 | 9 Saccular  9 Dissection | 8(15.38%) | Stents, SAC, Coiling, Bypass, FD,  Trapping, Flow reversal* | mRS, angiographic follow-up, complications, mortality | 33.0 |
| Seifert 2001 | 24 | 49 | 14/10 | 24 | NA | 3 Fusiform | 22(91.67%) | Clipping(24) | angiographic follow-up, complications, mortality | >12 |
| Sim 2022 | 40 | 50.5 | 19/21 | 40 | 10.6 | 29 Saccular  11 Fusiform | 27(67.50%) | Coiling(9), SAC(17), Stents(6),  FD(5), vertebral artery occlusion(3) | GOS, angiographic follow-up, complications, mortality | 26.3 |
| Tjahjadi 2016 | 14 | 47.9 | NA | 14 | 5.5 | NA | 19(100%) | Clipping(14) | mRS, GOS, angiographic follow-up, complications, mortality | 6.2 |
| Uda 2001 | 39 | 51 | 10/29 | 41 | NA | 4 Fusiform | 27(65.85%) | Coiling(39) | GOS, angiographic follow-up, complications, mortality | 21.0 |
| van Oel 2013 | 13 | 59.7 | 7/6 | 13 | 21 | NA | 3(23.08%) | Stents, Coiling, FD* | GOS, angiographic follow-up, complications, mortality | 18.0 |
| Wallace 2019 | 12 | 8.9 | 9/3 | 13 | 8.9 | 5 Saccular  4 Dissection  7 Fusiform  1 Blister | 3(23.08%) | PED, PED+ Coiling* | mRS, angiographic follow-up, complications, mortality | 9.2 |
| Wang 2021 | 28 | 53.7 | 19/9 | 28 | 7.5 | 21 Saccular  3 Fusiform  5 spherical | 10(35.71%) | SAC(28) | mRS, angiographic follow-up, complications, mortality | 16.0 |
| Wu 2023 | 34 | 60.2 | 21/34 | 34 | 14.5 | 5 Saccular  29 Dissection/Fusiform | 3(8.82%) | FD(13), Stents(11), SAC(10) | mRS, angiographic follow-up, complications, mortality | 21.0 |
| Yu 2010 | 16 | 45.8 | 11/5 | 16 | NA | 11 Saccular  4 Dissection  1 Fusiform | 15(93.75%) | Coiling(8), Balloon-assisted coil(2),  SAC(2), Coiling+PAO(2) | GOS, angiographic follow-up, complications, mortality | 12-24 |
| Zhang 2018 | 12 | 57.3 | 9/3 | 12 | NA | NA | NA | Bypass(7), Stents(4) | mRS, angiographic follow-up, complications, mortality | 18.4 |
| Zhong 2023 | 111 | 58 | 81/30 | 111 | 10.2 | 69 Saccular  31 Fusiform | 26(23.42%) | BA+Coiling(7), BA +Stents(4), BA +FD(1),  BA(99) | mRS, angiographic follow-up, complications, mortality | 36.0 |
| Summary | 593 | 55.4 | 291*/226* | 599 | ＞12.35 | 209Saccular  74Fusiform | / | / | / | 26.5* |

**SAC, Stent-assisted coiling; IPO, Immediately proximal parent artery occlusion; RPO, Remotely proximal parent artery occlusion; FD, Flow diverter; PED,** **Pipeline Embolization Device; GOS, Glasgow Outcome Scale; mRS, Modified Rankin Scale; BA, Balloon angioplasty. * The statistical data are not available in several articles.**
