# Supplementary material for: Management strategies and outcomes of basilar trunk aneurysms: a systematic review and meta-analysis
Source: Langenbecks Arch Surg. 2026 Jan 20;411(1):71. doi: 10.1007/s00423-025-03959-3 (PMC12852281; doi:10.1007/s00423-025-03959-3)
Supplement: Supplementary file 1 — Supplementary file1 (ZIP 55327 KB) [file 423_2025_3959_MOESM1_ESM.zip › literature.zip!/meta/anson 1997.pdf]

J Neurosurg 84:185–193, 1996

Characteristics and surgical treatment of dolichoectatic and fusiform aneurysms

JOHN A. ANSON, M.D., MICHAEL T. LAWTON, M.D., AND ROBERT F. SPETZLER, M.D.

Division of Neurological Surgery, Barrow Neurological Institute, St. Joseph's Hospital and Medical Center, Phoenix, Arizona; and Division of Neurosurgery, University of New Mexico, Albuquerque, New Mexico

✔ Dolichoectatic and fusiform aneurysms represent a small subset of cerebral aneurysms and are often among the most difficult to treat. A consecutive series of 40 patients with 41 of these two types of aneurysms is presented, including their clinical characteristics and surgical treatments. Common to all aneurysms was the pathological involvement of a length of blood vessel with separate inflow and outflow sites (nonsaccular). However, dolichoectatic aneurysms have markedly different symptoms and surgical treatments depending on their location in either the anterior or posterior circulation. Anterior circulation aneurysms involved the petrous internal carotid artery (ICA) in one, the supraclinoid ICA in three, the middle cerebral artery in 13, and the anterior cerebral artery in four patients. Posterior circulation aneurysms involved the basilar artery in 13, the vertebral artery in six, and the posterior inferior cerebellar artery in one patient. Various surgical procedures were performed, including direct clipping, trapping with bypass, proximal occlusion, resection with reanastomosis, transposition, aneurysmorrhaphy with thrombectomy, and wrapping. There was no surgical mortality in the patient series, and treatment was effective in many patients. Overall, outcome at late follow up was good (Glasgow Outcome Scale scores 1–2) in 78% of patients. Patients with anterior circulation aneurysms had better outcomes than patients with posterior circulation aneurysms, with good outcomes in 90% and 65% of the cases, respectively. Dolichoectatic and giant serpentine aneurysms may develop from smaller fusiform aneurysms and represent a spectrum of the same pathological entity. Arterial dissection may also play a role in the initial development of these aneurysms.

KEY WORDS • **aneurysm** • **bypass** • **dissection** • **dolichoectasia** • **fusiform** • **serpentine** • **subarachnoid hemorrhage**

FUSIFORM and dolichoectatic aneurysms are uncommon cerebral aneurysms, which often have clinical presentations and therapeutic considerations that differ from those associated with saccular aneurysms. There appears to be a spectrum of dolichoectatic aneurysms ranging from small fusiform aneurysmal dilations of a single vessel to giant dolichoectatic aneurysms filled largely with thrombus. The latter have also been described as giant serpentine aneurysms. These aneurysms, particularly the large partially thrombosed lesions, often produce clinical symptoms by compression of surrounding neural structures, by distal embolization, or by causing subarachnoid hemorrhage (SAH).

Because these aneurysms lack a definable neck that can be clipped to preserve the parent vessel, they are particularly difficult to treat surgically. Often the vessel of origin is either circumferentially involved by fusiform aneurysmal dilation or completely lost in a large thrombosed mass with distal branches arising from the aneurysm dome. Eliminating the risks of hemorrhage or compression without compromising distal cerebral blood supply in that vascular territory often requires unusual and technically difficult approaches. This report describes the surgical techniques used to treat various fusiform and giant dolichoectatic aneurysms during an 8-year period. Because both the clinical presentations of and the surgical approaches to lesions of the anterior and posterior circulation tended to differ, these two groups are differentiated throughout this report.

Clinical Material and Methods

Patient Population

Between 1986 and 1994, 40 patients (25 males, 15 females) with symptomatic dolichoectatic aneurysms were treated at the Barrow Neurological Institute. The patients ranged in age from 5 to 77 years (mean 45 years). Thirty-eight patients underwent surgery and two were treated medically with anticoagulation therapy. Pre- and postoperative neurological function was evaluated using the Glasgow Outcome Scale[31] (GOS), in which a GOS score of 1 is a good recovery, 2 is moderate disability, 3 is severe disability, 4 is vegetative, and 5 is dead.

TABLE 1
*Clinical characteristics of patients with anterior circulation fusiform and dolichoectatic aneurysms**

Case No.	Age (yrs), Sex	Location	Aneurysm Type	Presentation	Treatment	Complications	GOS Score	Follow Up (yrs)
1	28, F	rt ICA	fusiform	incidental†	clip reconstruction, supraclinoid ICA	pneumothorax	1	4.5
2	73, M	rt ICA	giant fusiform	CVA	wrapped	none	5	0.3
3	12, M	lt ICA	giant dolicho	incidental (head trauma)	clip reconstruction, supraclinoid ICA	none	1	0.3
4	14, M	lt MCA	giant serpentine	incidental (sinusitis)	STA–MCA bypass, distal occlusion; proximal occlusion, thrombectomy	none	1	2.0
5	29, M	rt MCA	giant fusiform	SAH	trapping, STA–MCA bypass	EDH	1	3.0
6	74, F	rt MCA	giant dolicho	incidental (recurrent epistaxis)	resection, end-to-end reanastomosis	none	1	3.0
7	62, M	lt MCA	giant fusiform	CVA	wrapped	none	5	5.0
8	35, F	lt MCA	giant dolicho	CVA	thrombectomy, aneurysmorrhaphy; trapping, STA–MCA bypass	MCA occlusion (postop Day 3)	2	4.8
9	39, M	rt MCA	dolicho	TIA (episodic face, hand numbness)	resection, STA–MCA bypass	none	1	6.0
10	70, M	lt MCA	giant dolicho	SAH	trapping, STA–MCA bypass	bypass occlusion	2	2.8
11	77, F	rt MCA	giant fusiform	incidental (herpes zoster)	wrapped	none	1	6.0
12	17, F	lt MCA	giant fusiform	CVA	ATA–MCA side-to-side bypass	none	1	4.5
13	44, M	lt MCA	giant fusiform	incidental (chest pain)	clip reconstruction, MCA	none	1	4.0
14	21, F	lt MCA, M_2	giant dolicho	incidental (otitis)	resection (efferent vessel thrombosed)	none	1	0.1
15	42, F	lt MCA	giant dolicho	CVA‡	resection, end-to-end reanastomosis	none	2	0.5
16	61, F	rt MCA	giant dolicho	incidental (syncope)	resection, end-to-end reanastomosis	EDH	1	0.8
17	64, F	lt A_2 ACA	dolicho	progressive vision loss	distal occlusion, A_2–A_2 side-to-side bypass	none	1	3.2
18	18, F	azygous ACA	giant dolicho	seizures	clip reconstruction, ACA	none	1	3.0
19	34, F	rt A_2 ACA	giant fusiform	seizures	resection, thrombosed aneurysm with extensive collateral circulation	none	1	3.3
20	44, F	lt A_1 ACA	fusiform	SAH	clip reconstruction, ACA	none	2	3.0

* Abbreviations: ACA = anterior cerebral artery; ATA = anterior temporal artery; CVA = cerebrovascular accident; dolicho = dolichoectatic; EDH = epidural hematoma; GOS = Glasgow Outcome Scale;[31] ICA = internal carotid artery; MCA = middle cerebral artery; SAH = subarachnoid hemorrhage; STA = superficial temporal artery; TIA = transient ischemic attack.

† Revealed in follow-up computerized tomography after craniopharyngioma resection.

‡ Reflects status after unsuccessful clipping attempt.

Aneurysm Location

Twenty-one aneurysms were located in the anterior circulation (Table 1), and 20 aneurysms were located in the posterior circulation (Table 2); one patient (Case 21) had two symptomatic dolichoectatic aneurysms along the basilar artery and the petrous internal carotid artery (ICA). Among the anterior circulation aneurysms, one involved the petrous ICA, three involved the supraclinoid ICA, 13 involved the middle cerebral artery (MCA), and four involved the anterior cerebral artery (ACA). Among the posterior circulation aneurysms, 13 involved the basilar artery, six involved the vertebral artery, and one involved the posterior inferior cerebellar artery (PICA). Eight patients had a total of 12 asymptomatic saccular aneurysms that were also detected on angiography.

Clinical Presentation

Six patients with anterior circulation aneurysms presented with thromboembolic symptoms (transient ischemic attacks (TIAs) and/or infarction). Three patients presented with SAH. One patient's vision diminished progressively due to compression of the optic apparatus by an ACA aneurysm, and two patients had aneurysms detected during evaluation for seizures. Eight patients had asymptomatic lesions identified during evaluation of apparently unrelated complaints. Preoperative neurological deficits were present in eight patients with anterior circulation aneurysms, including three patients with expressive aphasia, four patients with cranial nerve deficits, and six patients with abnormal motor examinations.

Patients with posterior circulation aneurysms most often presented with symptoms of brainstem compression and cerebellar dysfunction (10 patients), including quadriparesis, ataxia, and respiratory distress. There were no incidental, asymptomatic posterior circulation aneurysms. Four patients presented with SAH, and five patients presented with thromboembolic symptoms (including one patient with "locked-in" syndrome secondary to a brainstem infarct). Evaluation for a seizure revealed an aneurysm in one patient. Thirteen patients with posterior circulation aneurysms had neurological deficits, including 10 patients with cranial nerve deficits and eight patients with abnormal motor examinations. Overall, 11 patients had a clear history of systemic hypertension.

Surgical Treatment

Of the anterior circulation lesions, five aneurysms were treated directly with clip reconstruction of the parent ves-

TABLE 2

*Clinical characteristics of patients with posterior circulation fusiform and dolichoectatic aneurysms**

Case No.	Age (yrs), Sex	Location	Aneurysm Type	Presentation	Treatment	Complications	GOS Score	Follow Up (yrs)
21	13, F	1. VBJ	giant dolicho	progressive headache, nausea/vomiting, diplopia, ataxia	BA: thrombectomy, aneurysmorrhaphy (HCA);	LCND, pneumonia, herpes encephalitis	2	3.0
		2. petrous ICA	giant dolicho		ICA: trapping, ICA–MCA bypass			
22	64, M	BA	giant dolicho	progressive hemiparesis, ataxia, diplopia, ventilator dependence; S/P bilat VA occlusions	thrombectomy, aneurysmorrhaphy	none	5	0.5
23	57, M	BA	giant dolicho	locked-in syndrome	anticoagulation	none	3	2.8
24	70, M	BA	giant dolicho	ataxia, quadriparesis	thrombectomy, aneurysmorrhaphy	LCND, pneumonia, SCV thrombosis	5	1.3
25	43, F	BA	giant dolicho	progressive hemiparesis, ataxia, dysarthria	BA constricting snare (HCA); thrombectomy	ICH	3	5.0
26	45, M	BA	giant serpentine	progressive hemiparesis, ataxia, dysarthria	STA–SCA bypass; proximal balloon occlusion; thrombectomy	none	1	1.5
27	58, M	BA	giant dolicho	progressive hemiparesis, ataxia, diplopia, dysarthria; S/P rt VA occlusion & coils	STA–SCA bypass; distal clip occlusion; thrombectomy	EDH	5	0.8
28	59, M	BA	dolicho	hemianesthesia, dizziness	mobilization, transposition	none	2	0.8
29	66, M	BA	dolicho	episodic dizziness, vertigo, syncope	anticoagulation	none	1	1.0
30	39, M	lt VBJ	dolicho	episodic hemiparesis, hemianesthesia	mobilization, transposition	none	1	3.5
31	9, M	lt VBJ	giant dolicho	progressive headache, hemiparesis	thrombectomy, aneurysmorrhaphy (HCA), proximal occlusion (distal lt VA)	LCND, pneumonia	1	4.7
32	47, M	lt VBJ	giant dolicho	progressive hemiparesis, diplopia, dysphagia	STA–SCA bypass; proximal occlusion (distal lt VA)	none	2	0.1
33	63, M	lt VBJ	giant dolicho	progressive diplopia, facial numbness, dysphagia, gait ataxia	proximal occlusion (distal lt VA)	none	3	2.5
34	5, M	lt VA	giant fusiform	SAH	proximal occlusion, PICA–PICA bypass; occlusion lt PICA; distal coil occlusion	none	1	3.0
35	61, M	lt VA	fusiform	episodic paraparesis, facial numbness	mobilization, transposition	none	1	4.8
36	57, M	rt VA	fusiform	seizure	proximal occlusion (rt VA)	none	5	1.5
37	38, M	rt VA	fusiform	SAH	clip reconstruction, VA	pneumonia	2	4.5
38	38, F	rt VA	giant fusiform	TIA (acute ataxia, crossed hypesthesia)	proximal occlusion (rt VA), OA EAS	cerebrospinal fluid leak	1	3.0
39	42, M	rt VA	giant fusiform	SAH	clip reconstruction, VA	none	1	4.8
40	70, M	rt PICA	fusiform	SAH	resection, end-to-side reanastomosis	none	1	3.8

*Abbreviations: BA = basilar artery; dolicho = dolichoectatic; EAS = encephaloarteriosynangiosis; EDH = epidural hematoma; GOS = Glasgow Outcome Scale;[31] HCA = hypothermic circulatory arrest; ICA = internal carotid artery; ICH = intracerebral hematoma; LCND = lower cranial nerve deficit; MCA = middle cerebral artery; OA = occipital artery; PICA = posterior inferior cerebellar artery; SAH = subarachnoid hemorrhage; SCA = superior cerebellar artery; SCV = subclavian vein; STA = superficial temporal artery; S/P = status post; TIA = transient ischemic attack; VA = vertebral artery; VBJ = vertebrobasilar junction.

sel. Six aneurysms were resected in conjunction with a revascularization procedure, except for Cases 14 and 19. Three aneurysms (Cases 2, 7, and 11) treated early in this series were wrapped with muslin. The remaining seven patients underwent bypass, with either parent vessel occlusion or aneurysm trapping and are represented by two cases in Figs. 1 and 2.

Of the 20 posterior circulation aneurysms, 18 were treated surgically. Direct clip reconstruction was possible in only two patients with vertebral artery aneurysms. Four giant lower basilar/vertebrobasilar junction aneurysms were treated by varying degrees of thrombectomy, endarterectomy, and aneurysmorrhaphy using clips to reconstruct the parent vessel, which is illustrated by Case 22 (Fig. 3). All four aneurysms were exposed via the far-lateral approach and two required hypothermic circulatory arrest. In eight patients, the parent vessel was occluded to alter the blood flow through the aneurysm and promote thrombosis of its lumen. The vessels in five of these eight patients were revascularized. Three cases of vertebral artery dolichoectasia causing lower cranial nerve palsies were treated with mobilization and transposition of the vessels to relieve neural compression. One patient with a small fusiform PICA aneurysm had this lesion excised via primary reanastomosis of the vessel. Two patients with posterior circulation aneurysms (Cases 23 and 29) were treated with anticoagulation therapy.

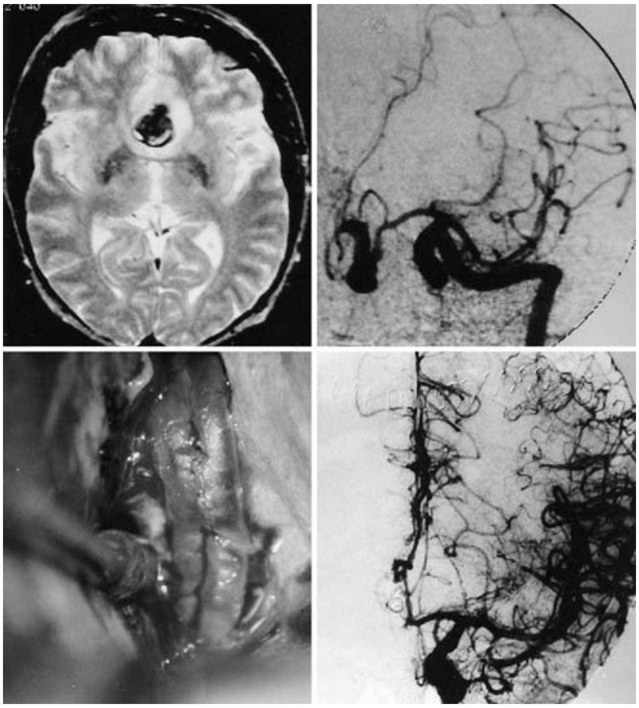

FIG. 1. Case 17. Imaging studies and introperative photograph in a patient with an anterior cerebral artery (ACA) aneurysm. *Upper Left:* A T_2-weighted magnetic resonance image demonstrating an ACA aneurysm in the interhemispheric fissure with some intraluminal thrombus. *Upper Right:* Preoperative left carotid angiogram (submental view) showing a fusiform aneurysm of the left ACA with decreased distal flow. *Lower Left:* Intraoperative photograph showing clip occlusion of the distal end of the aneurysm performed via an interhemispheric approach, with a concomitant distal side-to-side anastomosis of the two A_2 ACA vessels. *Lower Right:* Postoperative angiogram showing obliteration of the aneurysm with improved distal flow through the bypass. The patient's outcome was good.

Results

Surgical Outcome and GOS Scores

There was no surgical mortality in this patient series. Twelve patients (30%) encountered complications. Four patients had postoperative hematomas that required evacuation; two patients had thrombosis of the parent vessel or bypass that required revision; three patients had new lower cranial nerve deficits, and one patient had a cerebrospinal fluid leak requiring a shunt. Other miscellaneous complications included four patients with pneumonia, one with pneumothorax, one with catheter-induced subclavian vein thrombosis, and one with postoperative herpes encephalitis.

For the entire series, the mean follow-up period was 2.8 years. Overall, 23 patients made good recoveries (GOS score 1, 58%), eight had moderate disabilities (GOS score 2, 20%), three had severe disabilities (GOS score 3, 8%), and six died (GOS score 5, 15%). Patients with anterior circulation aneurysms had better outcomes than patients with posterior circulation aneurysms. In the former group, 18 patients (90%) received GOS scores of 1 or 2, and two patients died. The latter group included 13 patients (65%) with scores of 1 or 2, three patients with severe disabilities, and four patients who died.

Three patients were neurologically worse (by one GOS grade) after treatment. At late follow up, one of these patients had returned to his preoperative condition and one patient had improved compared to his preoperative condition. Neurologically, the third patient remained worse with increased lower cranial nerve deficits after aneurysmorrhaphy and died 1.3 years after treatment.

Five other patients in this series died. Two patients (Cases 22 and 27) with giant basilar artery aneurysms, treated previously at other institutions with vertebral artery occlusions, presented to this institution with progressive, severe lower cranial nerve deficits (GOS score 3). They did not improve with further treatment and died of respiratory complications 6 and 9 months later, respectively. Three patients died from an unrelated illness: one patient (Case 7) died from pneumonia 5 years after treatment (GOS score 1); one patient (Case 36) was diagnosed with acute myelogenous leukemia during aneurysm treatment and died from his disease 1.5 years later (GOS score 1); and one patient (Case 2) developed acute renal failure 4 months after treatment and ultimately died from a bleeding gastric ulcer (GOS score 2).

Many patients presented with progressive symptoms and steady neurological deterioration. Importantly, only one patient continued to deteriorate over time. This elderly patient presented with multiple deficits related to brainstem compression by a giant vertebrobasilar aneurysm. His clinical condition was no worse after treatment (occlusion of the distal vertebral artery), but at his 2.5-year follow-up examination he had become more disabled and dependent on others for daily support. In the remaining patients, the neurological course, as reflected in GOS scores, either stabilized (25 patients, 74%) or improved (eight patients, 24%).

Discussion

Incidence and Location

Dolichoectatic and fusiform aneurysms represent a small portion of intracranial aneurysms. Two large autopsy series of aneurysm patients that total more than 16,000 cases revealed only 15 fusiform type aneurysms (< 0.1%).[22,28] A more recent large series reviewing 50,000 cerebral angiograms identified only 31 patients with fusiform-type aneurysms (0.06%),[63] and another series of 538 vertebrobasilar aneurysms reported by Pia[45] identified four fusiform lesions (0.7%).

Classically, these lesions have been thought to affect primarily the vertebrobasilar circulation.[10,22,46,57] In our series, however, there was a much larger proportion of anterior circulation lesions, as has been noted previously.[28,35,63] In a review by Yu, *et al.*,[63] of 31 patients with ectasia of intracranial arteries, the carotid circulation was involved in 14 patients, the vertebrobasilar system in eight, and both in nine. In Housepian and Pool's[28] large autopsy series, the anterior and posterior circulation were involved equally. A recent review of what are referred to as giant serpentine aneurysms, which may represent an

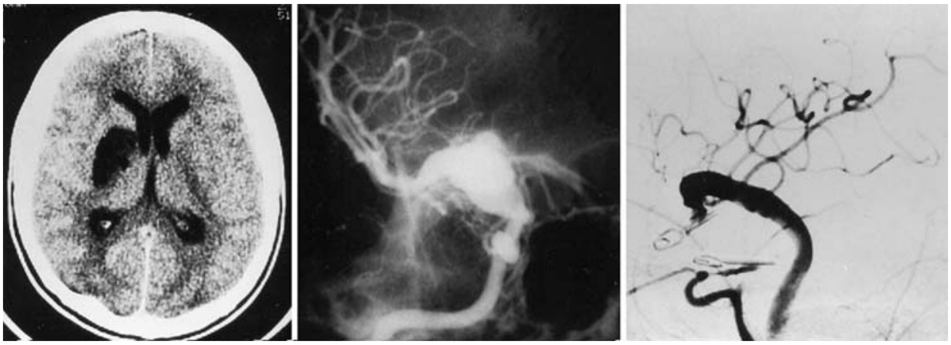

FIG. 2. Case 5. Imaging studies in a patient with a giant fusiform aneurysm. *Left:* Computerized tomography scan revealing an infarction of the right basal ganglia. *Center:* Angiography (right anteroposterior view) showing stenosis of the proximal right supraclinoid internal carotid artery (ICA), then a giant fusiform aneurysm of the distal ICA extending into the proximal M_1 segment of the middle cerebral artery (MCA). There was no filling of the right A_1 segment from the right carotid, but both anterior cerebral arteries filled from the left ICA. At surgery the complex aneurysm was trapped between clips on the ICA and MCA, after a saphenous vein bypass graft was performed from the superficial temporal artery trunk to the MCA. *Right:* Postoperative angiography (right lateral view) showing good flow through the bypass and no filling of the aneurysm.

extreme example of dolichoectatic aneurysms, reported anterior circulation involvement in 26 of 39 patients, including 18 on the MCA.[59] Our series also identified the MCA as the most commonly involved vessel in anterior circulation lesions. The reason for the predominance of MCA lesions is not clear but may be related to the greater length of that vessel.

Carotid ectasia may be more likely than vertebrobasilar lesions to become symptomatic, thereby accounting for their higher representation in clinical series. In contrast, vertebrobasilar lesions often may be asymptomatic and hence not be discovered until autopsy.[63] In a review of autopsies at the Mayo Clinic by Nijensohn, *et al.*,[42] only seven (30%) of 23 patients with vertebrobasilar ectasia were symptomatic.

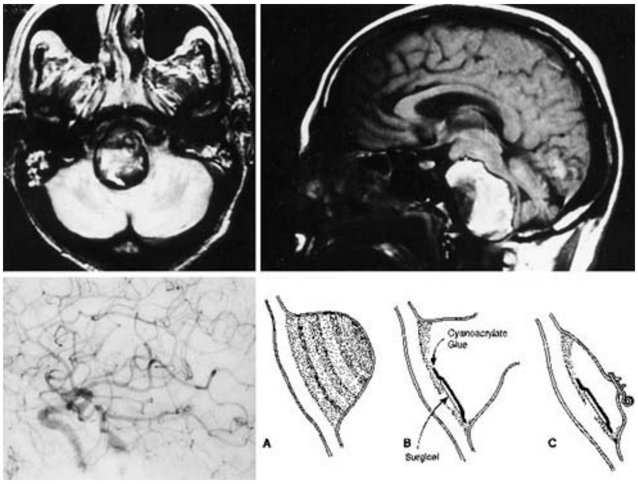

FIG. 3. Case 22. Imaging studies and drawing. Axial *(upper left)* and sagittal *(upper right)* magnetic resonance images demonstrating a giant dolichoectatic basilar artery aneurysm markedly compressing and displacing the brainstem. *Lower Left:* Right lateral carotid angiogram showing the patient previously had both vertebral arteries endovascularly occluded leaving only retrograde flow from the anterior circulation down the basilar artery to perfuse his brainstem. The goals of surgery were to decompress the brainstem and preserve this critical blood flow. After a far-lateral approach to the aneurysm, the aneurysm wall was opened and thrombus was removed until the residual lumen was entered. *Lower Right:* Drawing depicting the fenestration into the lumen, which was then sealed using Surgicel covered with cyanoacrylate adhesive. The posterior walls of the aneurysm were plicated down and over the sealed lumen with small hemoclips. Encountering the lumen during thrombectomy maximized the decompression, and reconstructing the wall maintained brainstem blood flow.

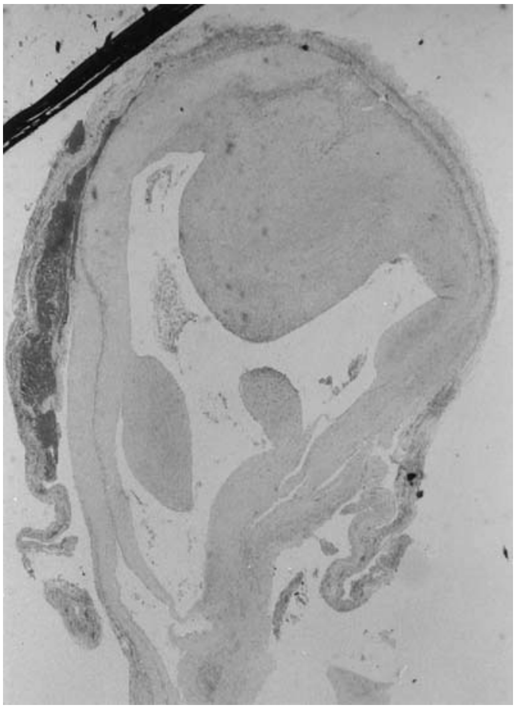

FIG. 4. Case 40. Pathological specimen showing attenuation of the vessel wall and intimal disruption.

Clinical Characteristics

Dolichoectatic aneurysms cause a remarkable diversity of clinical symptoms that can be grouped according to three mechanisms: compression, rupture, or ischemia. Compression of surrounding neural structures is the most common cause of symptoms with dolichoectatic aneurysms.[35,43,59]

One-third of the patients in this series had symptoms referable to compression of either surrounding cerebrum or adjacent cranial nerves. Lower cranial neuropathies secondary to compression are extremely common with vertebrobasilar dolichoectatic aneurysms and were seen in nine of our patients. Trigeminal neuralgia[12,16,36,43,46] and hemifacial spasm[16,19,21,32,33,46] have been reported, but facial numbness and weakness can also occur, as seen in this series. Oculomotor paralysis has been described, usually from third nerve compression.[3,26,61] Diplopia also reflects involvement of the trochlear[16] and abducens nerves.[7,35] Visual symptoms may also be caused by compression of the optic nerve or chiasm,[2,41] as in Case 17. Other reported compressive cranial nerve syndromes include sensorineural deafness from acoustic nerve compression,[7,41] dysarthria and dysphagia,[41] multiple lower cranial nerve palsies,[18,22,41,43] glossopharyngeal neuralgia,[6] supranuclear ophthalmoplegia, and medial longitudinal fasciculus syndromes.[6,16,55]

Enlarging basilar artery dolichoectatic aneurysms can also produce symptoms by direct compression of the brainstem and cerebellum,[4,46] as in 10 patients in our series. Ataxia, quadriparesis, and, in extreme cases, hypoventilation and a decreased level of consciousness also occur. In one series of 132 patients with megadolichovertebrobasilar anomaly, 41 had primarily cerebellar or pyramidal pathology.[46]

Hydrocephalus is another common result of mass effect from basilar artery dolichoectasia,[5,17,43,47] from direct deformation or from obstruction of the third ventricle or foramen of Monro.[11,47] Because some patients had no direct outflow obstruction, transmission of arterial pulsations from the ectatic vessel has been proposed to cause a "water-hammer" effect at the foramen of Monro, producing functional obstruction and hydrocephalus.[5,17] In these cases the pressure within the dilated lateral ventricles remains low in accordance with Pascal's law.[5,17]

Compared to dolichoectatic aneurysms, rupture is more common with giant saccular aneurysms.[14,27,58] Subarachnoid hemorrhage occurred in only seven (18%) of the 40 patients in this series, an incidence that is at the low end of the range reported in other patient series (19%–40%).[22,35,43]

Cerebral ischemia, the other major cause of symptoms from dolichoectatic aneurysms,[25,35,43,44,51,56,63] occurred in six patients with anterior circulation aneurysms and in four patients with posterior circulation aneurysms. The formation of thrombus within the lumen of these aneurysms causes ischemic symptoms: by directly impairing flow through the vessel lumen;[35] by blocking the lumen of perforating vessels originating along the length of the dolichoectasia;[25] and by causing embolization to distal vessels.[38,56] Brainstem infarction also occurs because of distortion of the paramedian branches as the basilar artery becomes ectatic and elongated.[43,51]

Surgical Treatment

The surgical approach to dolichoectatic aneurysms often is technically difficult and requires careful planning and execution. Although the specific approach is tailored for each patient based on the aneurysm's location, size, configuration, and flow characteristics, a few general considerations apply to most patients. Preoperative evaluation must include complete angiographic evaluation of not only the aneurysm but patterns of collateral flow, evidence of intraluminal thrombus, evidence of distal hypoperfusion, and potential vessels for bypass. Magnetic resonance (MR) imaging is particularly helpful for demonstrating the presence of thrombus within larger dolichoectatic aneurysms. Angiography, however, may underestimate the true size of the lesion by showing only the residual filling lumen.

We approached all anterior circulation aneurysms located on the ICA, MCA, or A_1 ACA through a standard pterional craniotomy with extensive drilling of the sphenoid wing. Occasionally an orbitozygomatic osteotomy is needed to increase the exposure. Preserving the superficial temporal artery during the skin incision is important in case distal bypass becomes necessary. We approached the three aneurysms on the A_2 branch of the ACA via an interhemispheric approach with partial resection of the falx.

We approached the posterior circulation aneurysms through either a large posterior suboccipital craniotomy or, more frequently, through the far-lateral approach (transcondylar),[1,24,50,53] which includes resection of the posterolateral third of the occipital condyle. This approach provides the best exposure of the intradural vertebral artery and was used for most of the vertebrobasilar lesions. By enlarging the medial extent of the bone opening, the distal PICAs can be exposed for a PICA–PICA bypass, as in Case 34. Occasionally, the transpetrosal approaches may be needed for midbasilar artery aneurysms as was the case in one patient in this series.

All surgeries were performed with electroencephalography and evoked potential monitoring, and barbiturate medications were titrated to the point of electroencephalography burst suppression for cerebral protection.[62] In three cases of vertebrobasilar dolichoectatic aneurysms, the extreme size of the aneurysm prevented safe or complete dissection without prolonged interruption of blood flow. These cases were therefore performed under hypothermic circulatory arrest, as reported previously.[54] This procedure is particularly useful for giant basilar dolichoectatic lesions because the surgeon can open the aneurysm, perform an internal thrombectomy, and reconstruct the parent vessel during the period of circulatory arrest.

When dolichoectatic aneurysms compress the brainstem, the goal of surgery must be directed toward decompression, either directly or by proximal vessel ligation. Drake[15] reported success with proximal occlusion in selected giant posterior circulation aneurysms but has stressed that morbidity and mortality rates may be substantial with this technique. We prefer a direct approach initially and to achieve decompression by thrombectomy of the aneurysm with either clipping or reconstruction of the aneurysm neck.

Our usual technique for aneurysm thrombectomy was

to open the aneurysm dome after either local or global circulatory arrest and to remove thrombus with an ultrasonic aspirator when possible. The ultrasonic aspirator is very effective for delicate removal of hardened thrombus, because it does not subject the aneurysm to the traction or manipulation that would be applied by piecemeal removal with cup forceps or pituitary rongeurs.[30,60] In most cases the softened aneurysm neck could then be clipped or occluded. In Case 22 (Fig. 3), however, an internal lumen was reconstructed using Surgicel and cyanoacrylate adhesive through the opening in the aneurysm dome. The lumen was thereby reconstituted through the space created by thrombus removal, with minimal exposure and manipulation of the basilar dolichoectasia and surrounding structures.

Most fusiform and dolichoectatic aneurysms do not have discrete necks like saccular aneurysms, and direct clipping generally refers to external reconstruction of the parent vessel lumen with clips. Occasionally, clip reconstruction does not obliterate the aneurysm completely and the residual portion can be wrapped with cotton to reinforce the wall.

Trapping and distal bypass were effective, particularly for anterior circulation lesions. Although proximal ICA and MCA aneurysms are typically considered most suitable for this approach using extracranial donor vessels, the use of other intracranial donor vessels permits trapping and bypass of aneurysms in other locations. The distal A_2 branch of the ACA can be anastomosed in a side-to-side fashion to provide bilateral distal flow after trapping a more proximal aneurysm. Similarly, the tonsilar loops of the PICA can be anastomosed to provide bilateral flow after occlusion of one proximal vessel. This consideration is important in PICA aneurysms proximal to the choroidal point, in which ligation would interrupt flow to important brainstem and cerebellar branch vessels.[29]

In certain cases fusiform aneurysms can be resected by direct reanastomosis of the proximal and distal ends of the vessel.[13,37,52] The length of the involved segment must be limited, with enough redundancy available in the vessel to avoid tension. All four patients in this series treated with this technique had excellent results.

The use of anticoagulation therapy alone also needs to be considered, particularly in patients with symptoms caused by progressive thrombosis or distal embolization. Anticoagulation therapy has been reported to be effective in patients with fusiform aneurysms and ischemic symptoms. In a series of 13 patients with fusiform aneurysms, the seven treated with anticoagulant agents had no recurrent symptoms or hemorrhagic complications during an 18-month period.[16] Similarly, in a study by Yu, *et al.*,[63] two of nine patients with ischemic symptoms treated with anticoagulation therapy were alive after 10 years, whereas seven untreated patients died of progressive brainstem ischemia. It has been suggested that all patients with vertebrobasilar fusiform aneurysms be placed on antiplatelet therapy, even those without ischemic symptoms.[43]

Pathophysiological Findings

Dandy[10] probably first stated that cerebral artery dolichoectasia are of arteriosclerotic origin. Later reports also support the theory of atherosclerotic degeneration of the vascular wall as the initial pathogenic factor in the development of these lesions.[9,22] Housepian and Pool[28] described grossly evident cerebral arteriosclerosis in 10 fusiform aneurysms as part of their 1958 autopsy series of 113 aneurysms. Their findings included intimal thickening and hyalinization, with lipid-laden phagocytes and cholesterin clefts.

Atherosclerosis, however, may not be the only or even most common cause of dolichoectatic aneurysms.[39,40,48] These lesions are so rare, despite the prevalence of atherosclerotic vascular disease, that other factors must contribute to their development.[40] Still, atheroma may enhance the effect of a congenital defect of the media.[8]

In the pathological analysis of 34 dolichoectatic ICAs, atherosclerotic disease was not a significant finding.[48] Rather, the media was irregular without atheromatous plaques. Multiple gaps were present, and the width of the internal elastic laminae varied. The thinning of the media was associated with atrophy of the muscle fibers and hyalinization of connective tissue. Although atherosclerosis may cause a vessel to widen by weakening its wall, it does not elongate vessels as observed in the patients in this and other series.[20,23,34,48,49,63] Hegedüs[23] also has identified severe reticular fiber deficiency in the media associated with elastic lamina defects.

Only two aneurysms in our series were analyzed pathologically. The vessel wall was attenuated from loss of elastic and smooth-muscle tissue, with some fibrous tissue and areas of intimal disruption (Fig. 4). At surgery, the gross appearance of many of the aneurysms was strikingly similar to the appearance of more typical cases of intracranial dissection. Often the proximal aspect of the aneurysm was dilated abruptly and more tapered distally. The surfaces of the lesions were dusky with prominent small vessels, similar to the appearance of dissection when exposed surgically. The association of this gross appearance with pathological findings of intimal disruption suggests that unrecognized arterial dissection may play a role in the initial development of these aneurysms.

Defects in the internal elastic laminae are a consistent pathological finding in most dolichoectatic aneurysms.[20,23,34,42,48] Progressive extreme enlargement of the vessel originating from the intimal defect may be an important factor in the pathogenesis of these lesions.[20,34,63] Recently, MR imaging has demonstrated that four cases of dolichoectasia with typical appearances were, in fact, dissections with aneurysmal dilation.[39] Separate flow patterns in the original constricted true lumen and slower parallel flow in the surrounding false lumen were visible.

The occurrence of these aneurysms in all age groups also supports dissection as an underlying etiological factor. In this series, seven patients were aged 18 years or younger and almost half were younger than 40 years, thus contradicting the idea of atherosclerosis as the pathogenic process. Although the large proportion of young patients also suggests the possibility of congenital abnormalities, only one of these patients had evidence of other aneurysms or vasculopathies.

Previous reports have suggested that congenital vascular defects are worsened by atherosclerotic disease;[8,40,48] however, the usual absence of other vascular lesions or atherosclerosis implies that this factor may be important in only a small number of cases. Fusiform, dolichoectatic,

and giant serpentine aneurysms may therefore represent a spectrum of nonsaccular aneurysms that share a common gross and microscopic appearance, and these lesions may arise from the same pathophysiological mechanism: intimal disruption from dissection. The uniform presence of intimal defects, a thinned or absent smooth-muscle layer, the frequent absence of atherosclerosis, the occurrence in all age groups, and the gross and MR-imaging appearance of these lesions support this hypothesis.

References

1. Anson JA, Spetzler RF: Endarterectomy of the intradural vertebral artery via the far lateral approach. **Neurosurgery 33:** 804–811, 1993
2. Bloch S, Ronthal M, Danziger J: Visual field defects produced by basilar artery ectasia. **Clin Radiol 31:**335–339, 1980
3. Boeri R, Passerini A: The megadolichobasilar anomaly. **J Neurol Sci 1:**475–484, 1964
4. Bollensen E, Buzanoski JH, Prange HW: Brainstem compression by basilar artery anomalies as visualized by MRI. **J Neurol 238:**49–50, 1991
5. Breig A, Ekbom K, Greitz T, et al: Hydrocephalus due to elongated basilar artery. A new clinicoradiological syndrome. **Lancet 1:**874–875, 1967
6. Brihaye J, Périer O, Smulders J, et al: Glossopharyngeal neuralgia caused by compression of the nerve by an atheromatous vertebral artery. **J Neurosurg 13:**299–302, 1956
7. Campbell JB, Pearman K, Nahl SS: Basilar artery ectasia: a rare cause of sensorineural deafness. **J Laryngol Otol 100:** 333–335, 1986
8. Carmichael R: The pathogenesis of non-inflammatory cerebral aneurysms. **J Pathol Bacteriol 62:**1–19, 1950
9. Courville CB: Arteriosclerotic aneurysms of the circle of Willis. Some notes on their morphology and pathogenesis. **Bull L A Neurol Soc 27:**1–13, 1962
10. Dandy WE: **Intracranial Arterial Aneurysms.** New York, Hafner, 1944
11. de los Reyes RA, Kantrowitz AB, Boehm FH, et al: Transcallosal, transventricular approach to a basilar apex aneurysm. **Neurosurgery 31:**597–602, 1992
12. Deeb ZL, Jannetta PJ, Rosenbaum AE, et al: Tortuous vertebrobasilar arteries causing cranial nerve syndromes: screening by computed tomography. **J Comput Assist Tomogr 3:** 774–778, 1979
13. Dolenc V: End-to-end suture of the posterior inferior cerebellar artery after the excision of a large aneurysm: case report. **Neurosurgery 11:**690–693, 1982
14. Drake CG: Giant intracranial aneurysms: experience with surgical treatment in 174 patients. **Clin Neurosurg 26:**12–95, 1979
15. Drake CG: Ligation of the vertebral (unilateral or bilateral) or basilar artery in the treatment of large intracranial aneurysms. **J Neurosurg 43:**255–274, 1975
16. Echiverri HC, Rubino FA, Gupta SR, et al: Fusiform aneurysm of the vertebrobasilar arterial system. **Stroke 20:**1741–1747, 1989
17. Ekbom K, Greitz T, Kalmér M, et al: Cerebrospinal fluid pulsations in occult hydrocephalus due to ectasia of basilar artery. **Acta Neurochir 20:**1–8, 1969
18. Frasson F, Ferrari G, Fugazzola C, et al: Megadolichobasilar anomaly causing brainstem syndrome. **Neuroradiology 13:** 279–281, 1977
19. Gibson WPR, Wallace D: Basilar artery ectasia. An unusual cause of a cerebello-pontine lesion and hemifacial spasm. **J Laryngol Otol 89:**721–731, 1975
20. Greitz T, Löfstedt S: The relationship between the third ventricle and the basilar artery. **Acta Radiol 42:**85–109, 1954
21. Harsh GR IV, Wilson CB, Hieshima GB, et al: Magnetic resonance imaging of vertebrobasilar ectasia in tic convulsif. Case report. **J Neurosurg 74:**999–1003, 1991
22. Hayes WT, Bernhardt H, Young JM: Fusiform arteriosclerotic aneurysm of the basilar artery. Five cases including two ruptures. **Vasc Surg 1:**171–178, 1967
23. Hegedüs K: Ectasia of the basilar artery with special reference to possible pathogenesis. **Surg Neurol 24:**463–469, 1985
24. Heros RC: Lateral suboccipital approach for vertebral and vertebrobasilar artery lesions. **J Neurosurg 64:**559–562, 1986
25. Hirsh LF, Gonzalez CF: Fusiform basilar aneurysm simulating carotid transient ischemic attacks. **Stroke 10:**598–601, 1979
26. Hopkins EW, Poser CM: Posterior cerebral artery ectasia. An unusual cause of ophthalmoplegia. **Arch Neurol 29:**279–281, 1973
27. Hosobuchi Y: Direct surgical treatment of giant intracranial aneurysms. **J Neurosurg 51:**743–756, 1979
28. Housepian EM, Pool JL: A systematic analysis of intracranial aneurysms from the autopsy file of the Presbyterian Hospital, 1914 to 1956. **J Neuropathol Exp Neurol 17:**409–423, 1958
29. Hudgins RJ, Day AL, Quisling RG, et al: Aneursyms of the posterior inferior cerebellar artery. A clinical and anatomical analysis. **J Neurosurg 58:**381–387, 1983
30. Hylton PD, Reichman OH: Endaneurysmal microendarterectomy in the treatment of giant cerebral aneurysms: technical note. **Neurosurgery 23:**674–679, 1988
31. Jennett B, Bond M: Assessment of outcome after severe brain damage. A practical scale. **Lancet 1:**480–484, 1975
32. Katayama Y, Tsubokawa T, Miyazaki S, et al: Growth of totally thrombosed giant aneurysm within the posterior cranial fossa. Diagnostic and therapeutic considerations. **Neuroradiology 33:**168–170, 1991
33. Kerber CW, Margolis MT, Newton TH: Tortuous vertebrobasilar system: a cause of cranial nerve signs. **Neuroradiology 4:**74–77, 1972
34. Ley A: Compression of the optic nerve by a fusiform aneurysm of the carotid artery. **J Neurol Neurosurg Psychiatry 13:** 75–86, 1950
35. Little JR, St Louis P, Weinstein M, et al: Giant fusiform aneurysm of the cerebral arteries. **Stroke 12:**183–188, 1981
36. Lye RH: Basilar artery ectasia: an unusual cause of trigeminal neuralgia. **J Neurol Neurosurg Psychiatry 49:**22–28, 1986
37. Madsen JR, Heros RC: Giant peripheral aneurysm of the posterior inferior cerebellar artery treated with excision and end-to-end anastomosis. **Surg Neurol 30:**140–143, 1988
38. Maruyama M, Asai T, Kuriyama Y, et al: Positive platelet scintigram of a vertebral aneurysm presenting thromboembolic transient ischemic attacks. **Stroke 20:**687–690, 1989
39. Mizutani T, Aruga T: "Dolichoectatic" intracranial vertebrobasilar dissecting aneurysm. **Neurosurgery 31:**765–773, 1992
40. Moffie D: Fusiform aneurysm of intracranial arteries. **Psychiatr Neurol Neurochir 71:**85–91, 1968
41. Moseley IF, Holland IM: Ectasia of the basilar artery: the breadth of the clinical spectrum and the diagnostic value of computed tomography. **Neuroradiology 18:**83–91, 1979
42. Nijensohn DE, Saez RJ, Reagan TJ: Clinical significance of basilar artery aneurysms. **Neurology 24:**301–305, 1974
43. Nishizaki T, Tamaki N, Takeda N, et al: Dolichoectatic basilar artery: a review of 23 cases. **Stroke 17:**1277–1281, 1986
44. Pessin MS, Chimowitz MI, Levine SR, et al: Stroke in patients with fusiform vertebrobasilar aneurysms. **Neurology 39:** 16–21, 1989
45. Pia HW: Classification of vertebro-basilar aneurysms. **Acta Neurochir 47:**3–30, 1979
46. Resta M, Gentile MA, Di Cuonzo F, et al: Clinical-angiographic correlations in 132 patients with megadolichovertebrobasilar anomaly. **Neuroradiology 26:**213–216, 1984
47. Rozario RA, Levine HL, Scott RM: Obstructive hydrocephalus

secondary to an ectatic basilar artery. **Surg Neurol 9:**31–34, 1978
48. Sacks JG, Lindenburg R: Dolicho-ectatic intracranial arteries. Symptomatology and pathogenesis of arterial elongation and distention. **Johns Hopkins Med J 125:**95–106, 1969
49. Segal HD, McLaurin RL: Giant serpentine aneurysm. Report of two cases. **J Neurosurg 46:**115–120, 1977
50. Sen CN, Sekhar LN: Surgical management of anteriorly placed lesions at the craniocervical junction—an alternative approach. **Acta Neurochir 108:**70–77, 1991
51. Shirakuni T, Tamaki N, Matsumoto S, et al: Megadolichobasilar anomaly associated with brain stem infarction: a case report. **J Comput Tomogr 9:**79–81, 1985
52. Smith RR, Parent AD: End-to-end anastomosis of the anterior cerebral artery after excision of a giant aneurysm. Case report. **J Neurosurg 56:**577–580, 1982
53. Spetzler RF, Grahm TW: The far lateral approach to the inferior clivus and the upper cervical region: technical note. **Barrow Neurol Inst Q 6:**35–38, 1990
54. Spetzler RF, Hadley MN, Rigamonti D, et al: Aneurysms of the basilar artery treated with circulatory arrest, hypothermia, and barbiturate cerebral protection. **J Neurosurg 68:**868–879, 1988
55. Stark RJ: Supranuclear ophthalmoplegia with basilar artery aneurysms. **Surg Neurol 12:**447–452, 1979
56. Steel JG, Thomas HA, Strollo PJ: Fusiform basilar aneurysm as a cause of embolic stroke. **Stroke 13:**712–716, 1982
57. Stehbens WE: **Pathology of the Cerebral Blood Vessels.** St Louis: CV Mosby, 1972 pp 351–470
58. Sundt TM Jr, Piepgras DG: Surgical approach to giant intracranial aneurysms. Operative experience with 80 cases. **J Neurosurg 51:**731–742, 1979
59. Suzuki S, Takahashi T, Ohkuma H, et al: Management of giant serpentine aneurysms of the middle cerebral artery—review of literature and report of a case successfully treated by STA-MCA anastomosis only. **Acta Neurochir 117:**23–29, 1992
60. Symon L, Vajda J: Surgical experiences with giant intracranial aneurysms. **J Neurosurg 61:**1009–1028, 1984
61. Trobe JD, Glaser JS, Quencer RC: Isolated oculomotor paralysis. The product of saccular and fusiform aneurysms of the basilar artery. **Arch Ophthalmol 96:**1236–1240, 1978
62. Wilkinson E, Spetzler RF, Carter LP, et al: Intraoperative barbiturate therapy during temporary vessel occlusion in man, in Spetzler RF, Carter LP, Selman WR, et al (eds): **Cerebral Revascularization for Stroke.** New York: Thieme-Stratton, 1985, pp 397–405
63. Yu YL, Moseley IF, Pullicino P, et al: The clinical picture of ectasia of the intracerebral arteries. **J Neurol Neurosurg Psychiatry 45:**29–36, 1982

Manuscript received June 2, 1994.
Accepted in final form August 4, 1995.
Address reprint requests to: Robert F. Spetzler, M.D., Neuroscience Publications, Barrow Neurological Institute, 350 West Thomas Road, Phoenix, Arizona 85013-4496.
