# Supplementary material for: Management strategies and outcomes of basilar trunk aneurysms: a systematic review and meta-analysis
Source: Langenbecks Arch Surg. 2026 Jan 20;411(1):71. doi: 10.1007/s00423-025-03959-3 (PMC12852281; doi:10.1007/s00423-025-03959-3)
Supplement: Supplementary file 1 — Supplementary file1 (ZIP 55327 KB) [file 423_2025_3959_MOESM1_ESM.zip › literature.zip!/meta/Cho 2015.pdf]

Taylor & Francis
Taylor & Francis Group

Neurological Research

A Journal of Progress in Neurosurgery, Neurology and Neurosciences

ISSN: 0161-6412 (Print) 1743-1328 (Online) Journal homepage: https://www.tandfonline.com/loi/yner20

# Saccular or dissecting aneurysms involving the basilar trunk: Endovascular treatment and clinical outcome

Kwang-Chun Cho, Pyoung Jeon, Byung Moon Kim, Soo Mee Lim, Woo Sang Jung, Jung-Jae Kim & Sang Hyun Suh

**To cite this article:** Kwang-Chun Cho, Pyoung Jeon, Byung Moon Kim, Soo Mee Lim, Woo Sang Jung, Jung-Jae Kim & Sang Hyun Suh (2019) Saccular or dissecting aneurysms involving the basilar trunk: Endovascular treatment and clinical outcome, Neurological Research, 41:7, 671-677, DOI: 10.1080/01616412.2019.1611185

**To link to this article:** https://doi.org/10.1080/01616412.2019.1611185

Published online: 02 May 2019.

Submit your article to this journal

Article views: 59

View related articles

View Crossmark data

Full Terms & Conditions of access and use can be found at
https://www.tandfonline.com/action/journalInformation?journalCode=yner20

NEUROLOGICAL RESEARCH
2019, VOL. 41, NO. 7, 671–677
https://doi.org/10.1080/01616412.2019.1611185

ORIGINAL RESEARCH PAPER

# Saccular or dissecting aneurysms involving the basilar trunk: Endovascular treatment and clinical outcome

Kwang-Chun Cho[a], Pyoung Jeon[b], Byung Moon Kim[c], Soo Mee Lim[d], Woo Sang Jung[e], Jung-Jae Kim[f] and Sang Hyun Suh[g]

[a]Department of Neurosurgery, Catholic Kwandong University College of Medicine, International St. Mary's Hospital, Incheon, Korea; [b]Department of Radiology, Samsung Medical Center, Sungkyunkwan University School of Medicine, Seoul, Korea; [c]Department of Radiology, Yonsei University College of Medicine, Seoul, Korea; [d]Department of Radiology, College of Medicine, Ehwa Woman's University, Ewha Womans University Seoul Hospital, Seoul, Korea; [e]Department of Radiology, Ajou University Hospital, Ajou University College of Medicine, Suwon, Korea; [f]Department of Neurosurgery, College of Medicine, Ewha Womans University, Ewha Womans University Seoul Hospital, Seoul, Korea; [g]Department of Radiology, Gangnam Severance Hospital,Yonsei University College of Medicine, Seoul, Korea

**ABSTRACT**

**Objectives**: Basilar artery trunk aneurysms (BTAs) are a rare pathology and difficult to treat. We present our experience regarding angiographic results and clinical outcomes for 16 BTAs treated by reconstructive endovascular treatment (EVT) using stent or balloon.

**Methods**: Between January 2003 and December 2014, 15 patients (mean age, 58.6 years; 11 males) with 16 BTAs were enrolled. Clinical manifestation, outcomes and procedural complications were evaluated retrospectively, and follow-up angiography was performed 12 and 24 months after procedure.

**Results**: Subarachnoid hemorrhage (SAH) developed in seven aneurysms and nine were found incidentally. In one case, SAH followed by acute infarction on pons. The location of the aneurysms was the pure basilar artery (BA) trunk in 13 and the junction of the BA and the superior cerebellar artery in 3. Reconstructive EVT was technically successful in 15 aneurysms (93.8%) and failed in one due to the difficulty of vascular access. Stent/balloon-assisted coiling was performed in 13 aneurysms and sole stent therapy in two aneurysms. One patient had periprocedural complication of acute in-stent thrombosis. All treated patients had no symptoms with the usual activities except three patients, who died from myocardial infarction, aneurysmal rebleeding, and cerebellar infarction. Angiographic follow-up was performed in nine aneurysms; three aneurysms were recanalized (33.3%) and six aneurysms had no interval change (66.7%). There was no significant event during the follow-up period (mean, 23.5 months).

**Conclusion**: In the treatment of BTAs, reconstructive EVT may provide a feasible and safe option to microsurgery.

**ARTICLE HISTORY**
Received 31 July 2018
Accepted 20 April 2019

**KEYWORDS**
Basilar artery; basilar artery trunk aneurysm; reconstructive; endovascular treatment

## Objectives

Basilar artery trunk aneurysms (BTAs) are a rare pathology of intracranial aneurysms with a low incidence of less than 1% [1,2]. Although various surgical approaches and results for BTAs have been published in case reports and some articles [1,3–12], surgical management for these aneurysms is still difficult because of the anatomical complexity with the presence of cranial nerves and perforators to the brainstem, the deep and narrow surgical field, and lack of the surgical experience.

Initially, proximal artery occlusion using a detachable balloon was performed to treat BTAs [13,14] and the introduction of Guglielmi detachable coils made it possible to embolize them selectively without parent artery sacrifice [15–24]. Recent development of devices and techniques has led to reconstructive endovascular treatment (EVT) [23,25,26].

In this study, we tried to present our experience of reconstructive EVT of BTAs and compare with the published clinical data for BTAs.

## Methods

### *Patients*

The institutional review board approved this retrospective study, and informed consent was obtained from the patients or their families before the procedure. A database of patients with BTA, who underwent endovascular treatment for these aneurysms in three tertiary hospitals from 2003 to 2014, was reviewed retrospectively.

According to the previous reports, BTAs were defined as aneurysms in the BA, excluding BA apex and vertebrobasilar artery (VBA) junction aneurysm [27,28]. In this study, 13 aneurysms were located in the BA itself and three at the BA-superior cerebellar artery (SCA) junction; the

**CONTACT** Sang Hyun Suh suhsh11@yuhs.ac Department of Radiology, Gangnam Severance Hospital, Yonsei University College of Medicine, 211 Eonju-ro, Gangnam-gu, Seoul 06273, Korea
© 2019 Informa UK Limited, trading as Taylor & Francis Group

latter were incorporated with the SCA. Two aneurysms were associated with fenestration of the BA.

Diagnostic cerebral angiograms were performed in all patients before the endovascular procedure to evaluate the characteristics of BTAs and the vascular anatomy of the posterior circulation for endovascular treatment options, which were decided in consideration of the patient's clinical condition, age, comorbid disease, and the expected complications associated with the procedure.

### Endovascular procedure

EVT was performed in all patients under general anesthesia. EVT was attempted immediately after diagnostic angiography in patients with SAH and electively in asymptomatic patients. All patients had heparinization (activated clotting time >250 s) immediately after or during EVT and heparinization was maintained for 24 h after the procedure. For stent-assisted coiling (SAC), a dual antiplatelet regimen (clopidogrel, 75 mg/day and aspirin, 325 mg/day) was started 3 days before EVT and heparinization was maintained as above. In emergent cases, clopidogrel, 300 mg, and aspirin, 500 mg, were administered orally 1 day after the procedure.

In the procedure, a 5F or 6F guiding catheter system (Envoy; Codman Neurovascular, Raynham, MA, USA) was introduced via the femoral artery. Through the vertebral artery, a microcatheter (Prowler 14 microcatheter, Codman Neurovascular; Excelsior SL-10 microcatheter, Boston Scientific, Natick, Massachusetts) was carefully guided into the aneurysm sac of the BA. Using the jailing technique, a self-expandable intracranial stent (Neuroform stent, Boston Scientific; Enterprise stent, Codman Neurovascular; Solitaire *AB* stent, ev3 neurovascular, Irvine, CA, USA) or balloon occlusion catheter (Hyperglide, ev3 neurovascular) was inserted in the BA itself or from the posterior cerebral artery (PCA) to the BA in order to protect the parent artery from coil protrusion. Afterward, a detachable coil was delivered into the aneurysm sac via a jailed microcatheter, and this process was repeated until coil embolization was achieved with preservation of the incorporated arteries. In small aneurysms less than 2 mm in size, two stents overlapped in the BA without coiling. A vascular closure device (Perclose; Abbott Vascular Devices, Redwood City, California) was used to seal off the femoral artery puncture. All treated patients were maintained with heparin for 24 h after the procedure; clopidogrel, 75 mg/day, and aspirin, 150 mg/day, were also administered for 6 months in cases of stent implantation. After the procedure, external ventricular drainage (EVD) was performed if hydrocephalus was detected.

### Clinical outcome and follow-up

All patients were evaluated by postoperative angiography to confirm the status of aneurysm coiling. Angiographic findings were categorized as either complete occlusion (no filling of contrast agent in the aneurysm sac) or incomplete occlusion (residual filling of contrast agent in the remnant aneurysm sac). Periprocedural and postoperative complications, such as parent artery occlusion/dissection, intraoperative rupture, thromboembolism, and retroperitoneal hematoma, were also evaluated. A follow-up angiography was performed 12, and 24 months after the procedure to confirm recanalization and regrowth of the coiled aneurysm, patency of the BA, SCA and PCA, stent migration and in-stent restenosis. These angiographies were reviewed and classified into three categories as (1) progressive thrombosis, (2) no interval change, and (3) recanalization of the aneurysm sac. They were then compared with the previous postoperative and initial angiographies by two neuroradiologists (S.H.S, P.J.). Clinical outcomes were assessed according to the modified Rankin Scale (mRS), 6, 12, and 24 months after discharge by a neurosurgeon.

## Results

Fifteen patients (11 men and 4 women) with 16 BTAs were enrolled in this study, and their mean age was 58.6 years (range, 29–78 years; Table 1). SAH developed in seven aneurysms and nine were found incidentally. In one case, SAH followed by acute pontine infarction (patient 7). Thirteen aneurysms were of the wide-necked saccular type (dome-to-neck ratio <1.5), one fusiform aneurysm (patient 2), and two dissecting aneurysms (patient 5 and 7). One patient had two BTAs with different projection (patient 9).

Reconstructive EVT was successful in all aneurysms but one (patient 8), in which delivery of the stent into the BA failed due to stenosis and tortuosity of both vertebral arteries. SAC was performed in 12 aneurysms and balloon-assisted procedure in one. In two ruptured aneurysm of the BA, sole-stent therapy was applied with two overlapping stents.

Of 15 aneurysms with reconstructive EVT, complete occlusion was achieved in 11 (11/15, 73.3%) and incomplete occlusion in four (4/15, 26.7%). In three of the aneurysms incorporated with the SCA, all of the involved arteries showed good patency on the postoperative angiography. Among the 15 aneurysms with EVT, there was one case of periprocedural acute in-stent thrombosis during the procedure and was spontaneously resolved; three patients expired due to myocardial infarction, aneurysmal rebleeding, and cerebellar infarction. Due to the presence of hydrocephalus, EVD was performed in four patients with previous SAH. Twelve patients recovered well with no neurological symptom (mRS score 0–1) upon discharge.

The follow-up period was 1–45 months (mean, 23.5 months). Follow-up angiographies were obtained for nine aneurysms (9/12, 75.0%), and no evidence of recanalization was found in 6 aneurysms with EVT (6/

Table 1. Characteristics of patients with basilar artery trunk aneurysms and clinical outcomes.

| No | Sex | Age | Location | Morphology | Sx | HH | Size (mm) | Stent/size (mm) | Occlusion | Complications | Outcome (mRS) | Angiographic FU(mo) | FU results |
|---|---|---|---|---|---|---|---|---|---|---|---|---|---|
| 1 | M | 50 | BA | Saccular | SAH | 4 | 5 | NF/3x20 | Complete | Acute in-stent thrombosis | 1 | 20 | NI |
| 2 | M | 78 | BA | Fusiform | SAH | 2 | 8.8 | NF/4x15 | Incomplete | None | 1 | 20 | R |
| 3 | M | 55 | BA | Saccular | SAH | 4 | 14.5 | Sol/6x20 | Complete | Expired due to MI | 6 | NA | |
| 4 | M | 70 | BA | Saccular | SAH | 2 | 4.8 | NF/4.5x20 | Incomplete | None | 0 | 30 | R |
| 5† | M | 72 | BA | Dissection | SAH | 2 | 1 | ENT(2) 4x22 & 4x20 | Incomplete | Expired due to rebleeding | 6 | NA | |
| 6 | M | 52 | BA | Saccular | SAH | 3 | 0.97 | ENT(2) 4.5x22 & 4.5x14 | Incomplete | None | 1 | 24 | NI |
| 7 | F | 75 | BA | Dissection | Infarction → SAH | 2 | 6.54 | ENT(2) 4x22 & 4x17 | Complete | Expired due to cerebellar infarction | 6 | NA | |
| 8 | F | 64 | BA | Saccular | UA | | 7.1 | NF/3x20 | Fail | Access failure | NA | NA | |
| 9†† | M | 66 | BA | Saccular | UA | | 3.5 | NF/4x20 | Complete | None | 0 | 29 | NI |
| | | | BA | Saccular | UA | | 2 | NF/4x20 | Complete | None | 0 | 29 | NI |
| 10 | F | 56 | BA | Saccular | UA | | 6.9 | ENT/4x22 | Complete | None | 0 | NA | |
| 11§ | M | 51 | BA | Saccular | UA | | 10.4 | Balloon | Complete | None | 0 | NA | |
| 12 | M | 48 | BA | Saccular | UA | | 9.9 | NF/4.5x20 | Complete | None | 0 | 38 | R |
| 13 | M | 29 | SCA | Saccular | UA | | 8.2 | ENT/4.5x22 | Complete | None | 0 | NA | |
| 14 | F | 60 | SCA | Saccular | UA | | 3.8 | NF/4x15 | Complete | None | 0 | 38 | NI |
| 15 | M | 70 | SCA | Saccular | UA | | 2.5 | ENT/4.5x22 | Complete | None | 0 | 12 | NI |

† = overlapping stents; †† = two aneurysms in the basilar artery; § = balloon-assisted coiling; M = male; F = female; UA = unruptured aneurysm; HH = hunt and hess scale; NA = not available; Sx = symptoms; SAH = subarachnoid hemorrhage; R = recanalization of the aneurysm sac; NI = no interval change; FU = follow-up period; mRS = modified Rankin Scale; S = saccular; F = fusiform; MI = myocardial infarction.

9, 66.7%). Coil compaction with contrast filling of the aneurysm sac was noted in three aneurysms (3/9, 33.3%), two of which were initially proven to be incomplete occlusion. There was no evidence of aneurysm rupture, in-stent stenosis, stent migration or parent artery occlusion during the follow-up period. The surviving patients were able to carry out all usual duties and activities without symptoms (mRS 0–1).

### *Illustrative case*

A 75-year-old woman with a left-side weakness and left facial palsy was admitted to our hospital. Initial brain CT revealed no intracranial hemorrhage and acute infarction on pons was found in diffusion weighted image (Figure 1a and b). Brain CT angiography (CTA) and cerebral angiography confirmed a dissecting aneurysm on basilar trunk just proximal to the SCA (Figure 1c and d). About 24 h after angiography, the patient had mental deterioration abruptly. On brain CT, extensive subarachnoid hemorrhage (SAH) was noted on the basal cistern (Figure 1e). We performed reconstructive EVT using multiple stents (Enterprise 4 × 17, 4 × 22) and coils (Figure 1f) and EVD was performed on right Kocher's point. However, follow-up brain CT on 24 h later showed the diffuse brain swelling and multifocal hypoxic brain damage (Figure 1g). The patient was expired 11 days after procedure.

## Discussion

Since various surgical techniques and approaches for treatment of BTAs were introduced by Drake et al. [5,29], small case reports and some articles on microsurgery addressing this issue have been published covering subjects ranging from surgical deftness to the great experiences of few neurosurgeons [1,12,16,28,30–33]. Despite this accumulation of evidences, BTAs are still difficult to manage surgically for most neurosurgeons. On the other hand, EVT of BTAs has become available due to: (1) the accessibility of the BA without interference of the cranial nerves and perforating arteries, (2) the unsophisticated anatomy of the BA and 3) the evolution of the device and its related technology. The results for EVT of BTAs also showed favorable clinical outcomes with low morbidity and mortality [19,20,23,24,34]. In this study using a stent or balloon, we also showed 93.8% of technical success rate and excellent clinical outcome (78.6%). Moreover, our study showed good clinical outcome and complete occlusion were obtained in all cases of unruptured BTAs after coil embolization, except one failed case.

Recently, the incomplete occlusion rate is not high even in the cases of ruptured aneurysms. However, as shown in our study, incomplete occlusion rate seems

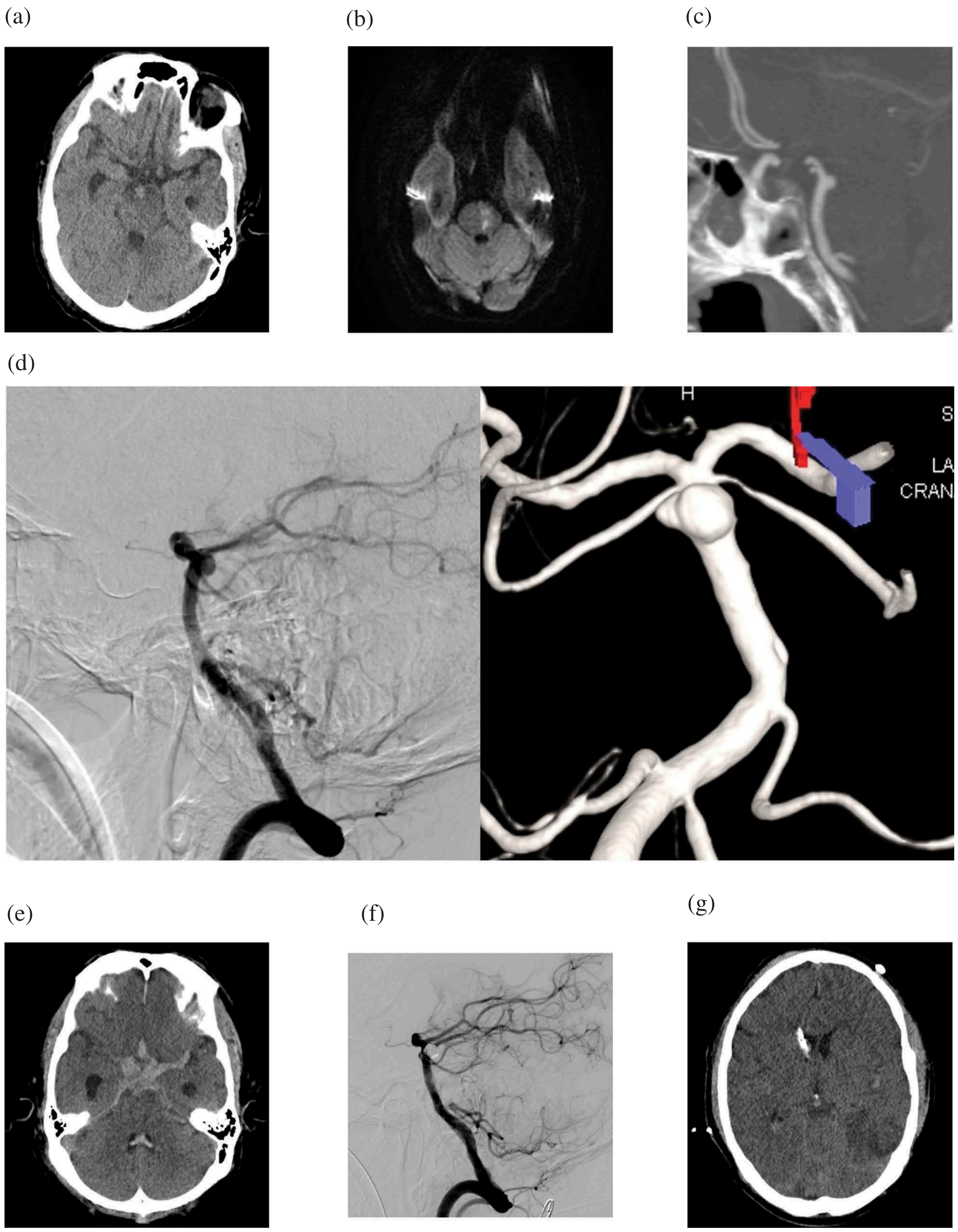


Figure 1. A 75-year-old woman with a dissecting BA trunk aneurysm. (a) Initial brain CT reveals no intracranial hemorrhage. (b) Diffusion weighted image shows acute infarction on pons. (c) Brain CT angiography shows a dissecting aneurysm in basilar trunk artery. (d) Digital subtraction angiography (DSA) depicts a dissecting aneurysm directed posteriorly. (e) Brain CT 24 h after the DSA shows a subarachnoid hemorrhage. (f) Control angiography reveals complete obliteration of aneurysm sac after reconstructive stent-assisted coiling. (g) Follow-up brain CT shows diffuse brain swelling and multifocal hypoxic brain damage.

to be high for the ruptured BTAs. Uda et al. reported 25 ruptured cases and 17 cases (68.0%) are occluded incompletely [19]. Chung et al. report 80.0% incomplete rate in five ruptured cases [35]. In our series, all four incomplete occlusion were ruptured cases and incomplete rate are 66.7% in ruptured 6

cases. It may be because of concerns about thromboembolic events, well-known procedural complications of reconstructive EVT using a stent or balloon, which derive from the thrombogenicity of the coils, balloon and stent in the circulatory system. To prevent this complication, it is important to perform EVT accurately and quickly.

In our study, the initial angiographic results showed 73.3% complete occlusion and 26.7% incomplete occlusion with preservation of all the incorporated arteries. Follow-up angiographies revealed stable results without coil compaction in 66.7% (6/9) and recanalization of the aneurysms in 33.3% (3/9) of cases, two of which initially had incomplete occlusion. Pandey et al. reported 20.9% (complete 16.2%; incomplete 50%) of the recanalization rate in 45 BTAs [23]. Uda et al. reported 31.4% complete occlusion at the initial angiographies and 31.0% (complete 0%; incomplete 42.9%) recanalization was found in the follow-up angiographies of 35 BTA with EVT [19]. These varied results may be interpreted as follows: (1) most of the treated BTAs had wide-necked or fusiform morphology and were large in size, (2) all procedures consisted of selective endosaccular coiling with preservation of the BA or the involved arteries, and (3) most of all the recanalized cases were identified as initially incomplete occlusion. Lozier et al. concluded that the incidence of recurrence was substantial in wide-necked aneurysms and incompletely coiled aneurysm of posterior circulation [36] (Table 2).

In the prognosis of the patients, preserving perforators during surgical or endovascular procedure of BTAs is a major concern. Contrary to other intracranial aneurysm, BTAs are densely related with the perforating arteries, and some BTAs may compress, stretch, distort, or embolize certain perforating branches of the BA. Therefore, there is still controversy about stent implantation in the perforator-rich vessels like BA [37]. Although animal studies with stent implantation showed no occlusion of small-diameter vessel after stenting [38,39], Wakhloo et al. reported a symptomatic perforator stroke in SAC of BTA [40]. Fortunately, we performed SAC on 12 wide-necked BTAs with no neurologic deficits. To eschew a devastating situation, the perforating arteries from the BA should be preserved carefully as possible under the adequate anticoagulation/antiplatelet therapy, especially in reconstructive EVT using thrombogenic materials like coil, stent or balloon. In using antiplatelet agents, it is necessary to evaluate clopidogrel resistance before procedure of the elective cases because a significant number (28%) of individuals do not respond to clopidogrel [41]. Because of the variable of clopidogrel nonresponse rates, considerable usage should be needed. Although abciximab is a rescue regimen in thromboembolic events of SAC [42], care should be taken to use glycoprotein IIb/IIIa inhibitor in this situation because of the substantial risk of rebleeding with ruptured aneurysms. Brinjikji et al. report a trend toward improved recanalization rates with glycoprotein IIb/IIIa inhibitors compared with thrombolytics [43]. Furthermore, they suggest that eptifibatide/tirofiban treatment results in significantly higher recanalization rates than treatment with abciximab, in their meta-analysis.

In addition, dissecting aneurysms seem to have a higher risk of mortality. In our cases, three patients expired due to myocardial infarction, aneurysmal rebleeding, and cerebellar infarction. All two patients with dissecting aneurysms were expired. Dissecting BTA is rare and main clinical presentation is SAH. In our study, comparing the saccular BTAs, dissecting BTAs have unfavorable outcome. Although EVT has recently been as the first option in posterior circulation aneurysms treatment, it should be approached with caution in the cases of dissecting aneurysms.

There are several limitations in this study. First, the number of the enrolled patients was too small to conclude the safety and feasibility of reconstructive EVT. However, BTA is extremely rare among all intracranial aneurysms and therapeutic experience is also minimal. Second, we have concerns about the durability of coiled aneurysms in the long-term follow-up period, during which coil compaction was found in 3 cases of our study. In review of the literatures (Table 2), BTAs have a high recurrence rate especially in the cases of incompletely occlusion [19,20,23,35]. Therefore, if BTA was incompletely embolized, it is essential to monitor them meticulously. Finally,

**Table 2.** Endovascular treatment of basilar trunk aneurysms: analysis of previous studies.

| Authors and year | An. cases | Initial complete occlusion rate | Initial occlusion | | Angiographic F/U cases | Angiographic F/U periods (mean months) | Recanalization rates | Good outcome |
|---|---|---|---|---|---|---|---|---|
| Uda et a19., 2001 | 35 | 31.4 % | Complete | 11 | 8 | 14.3 | 0 % | 90.9 % |
| | | | Incomplete | 24 | 21 | 19.8 | 42.9 % | |
| van Rooij et al.20., 2003 | 8 | 75.0 % | Complete | 6 | 5 | 15.0 | 0 % | 87.5 % |
| | | | Incomplete | 2 | 2 | 23.0 | 100 % | |
| Pandey et al.23., 2007 | 45 | 82.2 % | Complete | 37 | 37 | N/A | 16.2 % | 85.7 % |
| | | | Incomplete | 8 | 6 | N/A | 50 % | |
| Chung et al.35., 2011 | 8 | 37.5 % | Complete | 3 | 3 | 14.3 | 0 % | 75.0 % |
| | | | Incomplete | 5 | 3 | 16.0 | 0 % | |
| Present study | 15 | 73.3 % | Complete | 11 | 6 | 27.7 | 16.7% | 78.6 % |
| | | | Incomplete | 4 | 3 | 24.7 | 66.7% | |

An = aneurysm; F/U = follow-up; N/A = not available.

there is controversy regarding management of acute ruptured BA dissection. In our case (patient 5), selective coiling was impossible because the aneurysm was too small and involved perforator, so we had no choice but to treat this uncoilable lesion with multiple overlapping stents. Some authors have reported that multiple overlapping stents prevent rebleeding and heal the pseudoaneurysm [44–46]. High-metal-surface area constructs, such as Pipeline embolization devices (eV3 neurovascular), may be seriously considered as an alternative if circumstances allow.

In the treatment of BTAs, reconstructive EVT may provide a feasible and safe option to microsurgery. Further follow-up and experience will be necessary to determine the long-term results of reconstructive EVT of BTAs.

## Disclosure statement

No potential conflict of interest was reported by the authors.

## Notes on contributors

*Kwang-Chun Cho, M.D.* is a neurosurgeon and majored in cerebrovascular disease. He is undergoing endovascular surgery as well as scalpel surgery for cerebrovascular diseases. He is currently an assistant professor at Catholic Kwandong University College of Medicine and has several publications in the application of cerebrovascular diseases.

*Pyoung Jeon, M.D., Ph.D.* is a neuroradiologist as a professor at Samsung Medical Center of Sungkyunkwan University School of Medicine and an experienced expert in neurointervention for cerebrovascular diseases.

*Byung Moon Kim, M.D., Ph.D.* is a neuroradiologist and the present chairman of the Korean Society of Interventional Neuroradiology (KSIN). He is one of the best experts in neurointervention for cerebrovascular diseases. He has a lot of publications in the application of cerebrovascular diseases and neuroimaging.

*Soo Mee Lim, M.D., Ph.D.* is a neuroradiologist and an expert in neurointervention for cerebrovascular diseases. She is currently a professor at Ewha Womans University College of Medicine.

*Woo Sang Jung, M.D.* is a neuroradiologist and is undergoing endovascular treatment for cerbrovascular diseases. He is currently a clinical assistant professor at Ajou University College of Medicine.

*Jung-Jae Kim, M.D.* is a neurosurgeon and majored in cerebrovascular disease. He is currently a clinical assistant professor at Ewha Womans University College of Medicine.

*Sang Hyun Suh, MD, PhD* has been serving as a professor of the Department of Radiology at the Gangnam Severance Hospital, Yonsei University. He authored more than 116 scientific publications including 85 SCI(E) publications as well as two book chapters for neuroradiology and neurointervention.

## ORCID

Jung-Jae Kim http://orcid.org/0000-0002-4669-8577

## References

[1] Rice BJ, Peerless SJ, Drake CG. Surgical treatment of unruptured aneurysms of the posterior circulation. J Neurosurg. 1990;73:165–173.
[2] Origitano TC, Anderson DE, Tarassoli Y, et al. Skull base approaches to complex cerebral aneurysms. Surg Neurol. 1993;40:339–346.
[3] Drake CG. Bleeding aneurysms of the basilar artery. Direct surgical management in four cases. J Neurosurg. 1961;18:230–238.
[4] Jamieson KG. Aneurysms of the vertebrobasilar system; Surgical intervention in 19 cases. J Neurosurg. 1964;21:781–797.
[5] Drake CG. Surgical treatment of ruptured aneurysms of the basilar artery. Experience with 14 cases. J Neurosurg. 1965;23:457–473.
[6] Drake CG. Further experience with surgical treatment of aneurysm of the basilar artery. J Neurosurg. 1968;29:372–392.
[7] Kawase T, Toya S, Shiobara R, et al. Transpetrosal approach for aneurysms of the lower basilar artery. J Neurosurg. 1985;63:857–861.
[8] Sugita K, Kobayashi S, Takemae T, et al. Aneurysms of the basilar artery trunk. J Neurosurg. 1987;66:500–505.
[9] Kawase T, Bertalanffy H, Otani M, et al. Surgical approaches for vertebro-basilar trunk aneurysms located in the midline. Acta Neurochir (Wien). 1996;138:402–410.
[10] Ogilvy CS, Barker FG 2nd, Joseph MP, et al. Transfacial transclival approach for midline posterior circulation aneurysms. Neurosurgery. 1996;39:736–741. discussion 742.
[11] Seifert V. Direct surgery of basilar trunk and vertebrobasilar junction aneurysms via the combined transpetrosal approach. Neurol Med Chir (Tokyo). 1998;38(Suppl):86–92.
[12] Sanai N, Tarapore P, Lee AC, et al. The current role of microsurgery for posterior circulation aneurysms: a selective approach in the endovascular era. Neurosurgery. 2008;62:1236–1249. discussion 1249–1253.
[13] Zeumer H, Bruckmann H, Adelt D, et al. Balloon embolization in the treatment of basilar aneurysms. Acta Neurochir (Wien). 1985;78:136–141.
[14] Fox AJ, Vinuela F, Pelz DM, et al. Use of detachable balloons for proximal artery occlusion in the treatment of unclippable cerebral aneurysms. J Neurosurg. 1987;66:40–46.
[15] Pierot L, Boulin A, Castaings L, et al. Selective occlusion of basilar artery aneurysms using controlled detachable coils: report of 35 cases. Neurosurgery. 1996;38:948–953. discussion 953–944.
[16] Taki W, Nakahara I, Sakai N, et al. Large and giant middle to lower basilar trunk aneurysms treated by surgical and interventional neuroradiological methods. Neurol Med Chir (Tokyo). 1998;38:826–834. discussion 834–825.
[17] Nagashima H, Okudera H, Orz Y, et al. Endovascular treatment of basilar trunk aneurysm associated with fenestration of the basilar artery. Neurosurg Rev. 1999;22:219–221.

[18] Matsubara S, Satoh K, Satomi J, et al. Guglielmi detachable coil embolization for ruptured lower-midbasilar trunk aneurysms–a report of five cases. Neuroradiology. 2001;43:884–890.

[19] Uda K, Murayama Y, Gobin YP, et al. Endovascular treatment of basilar artery trunk aneurysms with Guglielmi detachable coils: clinical experience with 41 aneurysms in 39 patients. J Neurosurg. 2001;95:624–632.

[20] Van Rooij WJ, Sluzewski M, Menovsky T, et al. Coiling of saccular basilar trunk aneurysms. Neuroradiology. 2003;45:19–21.

[21] Hassan T, Ezura M, Takahashi A. Treatment of giant fusiform aneurysms of the basilar trunk with intra-aneurysmal and basilar artery coil embolization. Surg Neurol. 2004;62:455–462. discussion 462.

[22] Ponce FA, Albuquerque FC, McDougall CG, et al. Combined endovascular and microsurgical management of giant and complex unruptured aneurysms. Neurosurg Focus. 2004;17:E11.

[23] Pandey AS, Koebbe C, Rosenwasser RH, et al. Endovascular coil embolization of ruptured and unruptured posterior circulation aneurysms: review of a 10-year experience. Neurosurgery. 2007;60:626–636. discussion 636–627.

[24] Higa T, Ujiie H, Kato K, et al. Basilar artery trunk saccular aneurysms: morphological characteristics and management. Neurosurg Rev. 2009;32:181–191. discussion 191.

[25] Higashida RT, Smith W, Gress D, et al. Intravascular stent and endovascular coil placement for a ruptured fusiform aneurysm of the basilar artery. Case report and review of the literature. J Neurosurg. 1997;87:944–949.

[26] Lanzino G, Wakhloo AK, Fessler RD, et al. Efficacy and current limitations of intravascular stents for intracranial internal carotid, vertebral, and basilar artery aneurysms. J Neurosurg. 1999;91:538–546.

[27] Solomon RA, Stein BM. Surgical approaches to aneurysms of the vertebral and basilar arteries. Neurosurgery. 1988;23:203–208.

[28] Peerless SJ, Hernesniemi JA, Gutman FB, et al. Early surgery for ruptured vertebrobasilar aneurysms. J Neurosurg. 1994;80:643–649.

[29] Drake CG. The surgical treatment of aneurysms of the basilar artery. J Neurosurg. 1968;29:436–446.

[30] Day JD, Giannotta SL, Fukushima T. Extradural temporopolar approach to lesions of the upper basilar artery and infrachiasmatic region. J Neurosurg. 1994;81:230–235.

[31] Mizoi K, Yoshimoto T, Takahashi A, et al. Direct clipping of basilar trunk aneurysms using temporary balloon occlusion. J Neurosurg. 1994;80:230–236.

[32] Seifert V, Stolke D. Posterior transpetrosal approach to aneurysms of the basilar trunk and vertebrobasilar junction. J Neurosurg. 1996;85:373–379.

[33] Aziz KM, van Loveren HR, Tew JM Jr., et al. The Kawase approach to retrosellar and upper clival basilar aneurysms. Neurosurgery. 1999;44:1225–1234. discussion 1234–1226.

[34] Fujimoto K, Kawai S, Yonezawa T, et al. Basilar trunk aneurysms with associated fenestration treated by using Guglielmi detachable coils: two cases reports. J Stroke Cerebrovasc Dis. 2007;16:84–87.

[35] Chung J, Park H, Lim YC, et al. Endovascular treatment of basilar artery trunk aneurysms. Acta Neurochir (Wien). 2011;153:2137–2145.

[36] Lozier AP, Connolly ES Jr., Lavine SD, et al. Guglielmi detachable coil embolization of posterior circulation aneurysms: a systematic review of the literature. Stroke. 2002;33:2509–2518.

[37] Al-Ali F, Cree T, Hall S, et al. Predictors of unfavorable outcome in intracranial angioplasty and stenting in a single-center comparison: results from the borgess medical center-intracranial revascularization registry. AJNR Am J Neuroradiol. 2011;32:1221–1226.

[38] Wakhloo AK, Schellhammer F, de Vries J, et al. Self-expanding and balloon-expandable stents in the treatment of carotid aneurysms: an experimental study in a canine model. AJNR Am J Neuroradiol. 1994;15:493–502.

[39] Kallmes DF, Ding YH, Dai D, et al. A new endoluminal, flow-disrupting device for treatment of saccular aneurysms. Stroke. 2007;38:2346–2352.

[40] Wakhloo AK, Mandell J, Gounis MJ, et al. Stent-assisted reconstructive endovascular repair of cranial fusiform atherosclerotic and dissecting aneurysms: long-term clinical and angiographic follow-up. Stroke. 2008;39:3288–3296.

[41] Muller-Schunk S, Linn J, Peters N, et al. Monitoring of clopidogrel-related platelet inhibition: correlation of nonresponse with clinical outcome in supra-aortic stenting. AJNR Am J Neuroradiol. 2008;29:786–791.

[42] T SS R, Grzyska U, Zeumer H, et al. Abciximab is a safe rescue therapy in thromboembolic events complicating cerebral aneurysm coil embolization: single center experience in 42 cases and review of the literature. Stroke. 2009;40:1750–1757.

[43] Brinjikji W, Morales-Valero SF, Murad MH, et al. Rescue treatment of thromboembolic complications during endovascular treatment of cerebral aneurysms: a meta-analysis. AJNR Am J Neuroradiol. 2015;36:121–125.

[44] Lim J, Suh SH, Lee KY, et al. Endovascular treatment of iatrogenic intracranial pseudoaneurysm following stent angioplasty. J Neuroimaging. 2012;22:194–196.

[45] Fiorella D, Albuquerque FC, Deshmukh VR, et al. Endovascular reconstruction with the Neuroform stent as monotherapy for the treatment of uncoilable intradural pseudoaneurysms. Neurosurgery. 2006;59:291–300. discussion 291–300.

[46] Kim BM, Suh SH, Park SI, et al. Management and clinical outcome of acute basilar artery dissection. AJNR Am J Neuroradiol. 2008;29:1937–1941.
