# Supplementary material for: Management strategies and outcomes of basilar trunk aneurysms: a systematic review and meta-analysis
Source: Langenbecks Arch Surg. 2026 Jan 20;411(1):71. doi: 10.1007/s00423-025-03959-3 (PMC12852281; doi:10.1007/s00423-025-03959-3)
Supplement: Supplementary file 1 — Supplementary file1 (ZIP 55327 KB) [file 423_2025_3959_MOESM1_ESM.zip › literature.zip!/meta/coert2007.pdf]

J Neurosurg 106:855–865, 2007

Surgical and endovascular management of symptomatic posterior circulation fusiform aneurysms

Bert A. Coert, M.D.,[1] Steven D. Chang, M.D.,[1] Huy M. Do, M.D.,[1,2] Michael P. Marks, M.D.,[1,2] and Gary K. Steinberg, M.D., Ph.D.[1]

[1]Department of Neurosurgery, and [2]Division of Neuroradiology, and Stanford Stroke Center, Stanford University School of Medicine, Stanford, California

Object. Patients with fusiform aneurysms can present with subarachnoid hemorrhage (SAH), mass effect, ischemia, or unrelated symptoms. The absence of an aneurysm neck impedes the direct application of a clip and endovascular coil deployment. To evaluate the effects of their treatments, the authors retrospectively analyzed a consecutive series of patients with posterior circulation fusiform aneurysms treated at Stanford University Medical Center between 1991 and 2005.

Methods. Forty-nine patients (mean age 53 years, male/female ratio 1.2:1) treated at the authors' medical center form the basis of the analysis. Twenty-nine patients presented with an SAH. The patients presenting without SAH had cranial nerve dysfunction (five patients), symptoms of mass effect (eight patients), ischemia (six patients), or unrelated symptoms (one patient). The aneurysms were located on the vertebral artery (VA) or posterior inferior cerebellar artery (PICA) (21 patients); vertebrobasilar junction (VBJ) or basilar artery (BA) (18 patients); and posterior cerebral artery (PCA) (10 patients). Pretreatment clinical grades were determined using the Hunt and Hess scale; for patients with unruptured aneurysms (Hunt and Hess Grade 0) functional subgrades were added. Outcome was evaluated using the Glasgow Outcome Scale (GOS) score during a mean follow-up period of 33 months.

Overall long-term outcome was good (GOS Score 4 or 5) in 59%, poor (GOS Score 2 or 3) in 16%, and fatal (GOS Score 1) in 24% of the patients. In a univariate analysis, poor outcome was predicted by age greater than 55 years, VBJ location, pretreatment Hunt and Hess grade in patients presenting with SAH, and incomplete aneurysm thrombosis after endovascular treatment. In a multivariate analysis, age greater than 55 years was the confounding factor predicting poor outcome. Stratification by aneurysm location removed the effect of age. Of 13 patients with residual aneurysm after treatment, five (38%) subsequently died of SAH (three patients) or progressive mass effect/brainstem ischemia (two patients).

Conclusions. Certain posterior circulation aneurysm locations (PCA, VA–PICA, and BA–VBJ) represent separate disease entities affecting patients at different ages with distinct patterns of presentation, treatment options, and outcomes. Favorable overall long-term outcome can be achieved in 90% of patients with PCA aneurysms, in 60% of those with VA–PICA aneurysms, and in 39% of those with BA–VBJ aneurysms when using endovascular and surgical techniques. The natural history of the disease was poor in patients with incomplete aneurysm thrombosis after treatment.

Key Words • **fusiform aneurysm** • **posterior circulation aneurysm** • **occlusion** • **coil placement** • **outcome**

Different terms have been used to describe aneurysms that are not amenable to direct clip or coil therapy. Some of the terms, such as "dissecting aneurysm," implicate a probable mechanism of origin, whereas others such as "dolichoectatic" and "fusiform" are morphologically descriptive. This type of aneurysm occurs less frequently than its saccular counterpart, and patient demographics, clinical presentation, and natural history are distinctly different. Data on natural history and treatment results for these aneurysms are limited, but reports of frequent early rebleedings and poor outcome have resulted in a heightened sense of urgency.[15] Authors of a study in which nonsaccular aneurysms with angiographic features of acute dissection were excluded and in which 40% of patients were asymptomatic, demonstrated annual hemorrhage rates of approximately 1 to 2%.[6]

Posterior circulation fusiform aneurysms present difficult treatment challenges. Proximal or parent artery occlusions (Hunterian ligation), trapping procedures, and clip reconstructions are surgical techniques used to treat aneurysms that are not amenable to direct clip application. Endovascular options include parent vessel coil occlusion, stent placement, or combinations of coil and stent therapies. The selection of the treatment modality by our neurovascular team was based on the anticipated effectiveness of

Abbreviations used in this paper: BA = basilar artery; GOS = Glasgow Outcome Scale; PCA = posterior cerebral artery; PICA = posterior inferior cerebellar artery; SAH = subarachnoid hemorrhage; TIA = transient ischemic attack; VA = vertebral artery; VBJ = vertebrobasilar junction.

the treatment, taking into consideration the characteristics of the aneurysms, including morphological features, location, and orientation. To appraise the effect of our treatments we retrospectively analyzed a consecutive series of patients with posterior circulation fusiform aneurysms treated at Stanford University Medical Center.

Clinical Material and Methods

Patient Population

Between 1991 and 2005, 49 patients were treated at the authors' institution. The mean and median ages were 53 and 55 years for men and 52 and 50 years for women, respectively (see Fig. 1 for the distribution of age in subgroups based on location). The male/female distribution was 27:22 (ratio 1.2:1). Twenty-one aneurysms (43%) were located on the VA (20 patients) or on the PICA (one patient), 10 (20%) were at the PCA, and 18 (37%) on the BA or VBJ. Based on the three locations, patient ages ($p = 0.01$, Kruskal–Wallis test) and pretreatment grades ($p = 0.04$, Kruskal–Wallis test) were different between the locations. Twenty-seven patients (55%) underwent endovascular treatment, 19 (39%) underwent surgery, and three (6%) received a combination of endovascular and surgical treatments. These three patients underwent endovascular parent vessel occlusions after undergoing an extracranial–intracranial bypass procedure. Four patients treated endovascularly underwent shunt placement for hydrocephalus (one patient 2 months before and the other three patients 1, 2.5, and 9 months after parent vessel occlusion).

Neurological Assessment

The Hunt and Hess scale[9] was used for grading a patient's neurological condition before treatment. This scale does not grade symptoms caused by mass effect or ischemia in the absence of an SAH and thus fails to account for the fact that brainstem compression and cranial nerve deficits can seriously affect quality of life. The neurological condition in patients presenting with symptoms related to mass effect or ischemia such as TIA, stroke, cranial nerve dysfunction, dysarthria, or postural unsteadiness in addition to no SAH was graded Hunt and Hess Grade 0 with an added three-point functional subgrade (Table 1). Twenty-nine patients (59%) presented with SAH. A diagnosis of SAH varied based on aneurysm location—81% of patients in the VA–PICA group presented with SAH, whereas 60% of those in the PCA group and 33% of those in the BA–VBJ group presented with SAH (Table 2). In the other 20 patients (presenting without SAH), symptoms were attributed to brainstem compression in six (30%), cranial nerve dysfunction in four (20%), TIA or stroke in six (30%) patients, and mass effect caused by hydrocephalus in two (10%). Two patients (10%) presented with headache but no neurological deficits.

TABLE 1

Definition of Hunt and Hess unruptured aneurysm subclassifications

Hunt & Hess Grade	Definition
0_1	independent & able to work
0_2	independent but unable to work
0_3	dependent & unable to work

Outcome Assessment

Decisions about optimal treatment were made by our neurovascular team, consisting of neurosurgeons, interventional neuroradiologists, and in certain cases, stroke neurologists. The selection of treatment was based on the aneurysm location and morphological features as well as the expected results of endovascular and surgical treatment. The GOS[11] was used to assess outcome (a GOS score of 1, dead; 2, vegetative state; 3, severely disabled; 4, moderately disabled; and 5, good outcome). The mean follow-up period was 33 months (median 12 months, range 3–142

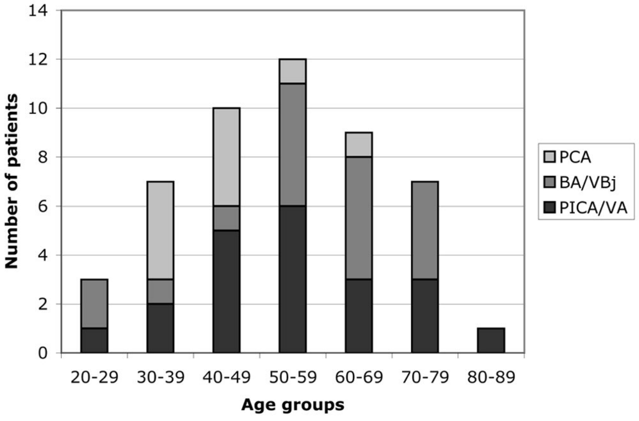

FIG. 1. Graph showing the number of patients per age group. Twenty-two (45%) of the 49 patients were between 40 and 60 years of age.

TABLE 2

*Summary of characteristics in patients with VA–PICA, PCA, and BA–VBJ aneurysms**

Characteristic	No. of Patients (%)† VA–PICA	PCA	BA–VBJ
no. of patients	21	10	18
median age in yrs (range)	52 (20–82)	43 (31–62)	60 (24–79)
median aneurysm size in mm (range)	12 (7–37)	17 (4–29)	22 (6-42)
pretreatment H & H grade			
0 ($_{1–3}$)	4 (19)	4 (40)	12 (67)
I–II	8 (38)	4 (40)	1 (6)
III–IV	9 (43)	2 (20)	5 (28)
treatment modality			
op	4 (19)	9 (90)	6 (33)
embol	16 (76)	1 (10)	10 (56)
embol & op	1 (5)	0 (0)	2 (11)
GOS score			
1	4 (24)	0 (0)	8 (44)
2–3	4 (24)	1 (10)	3 (17)
4–5	13 (62)	9 (90)	7 (39)

* Embol = embolization; H & H = Hunt & Hess.
† Unless otherwise specified.

months). Patients, referring physicians, and/or first-degree relatives were contacted by telephone to obtain follow-up data.

Statistical Analysis

Data were analyzed using version 9.1 SAS software (SAS, Inc.). Results were considered statistically significant for two-sided data if the probability value was less than or equal to 0.05 and somewhat significant if the probability value was less than 0.10. The chi-square test for independence was used to determine the association between outcome and categorical/ordinal clinical measures. If the null hypothesis of independence was rejected, partial chi-square statistics were evaluated to determine the cells that contributed most to the overall chi-square. The Cochran–Armitage test for trend was used to determine the linearity of the binomial proportions of the dichotomous factor across the ordinal levels of outcome. The nonparametric Kruskal–Wallis test was used to determine the association between continuous clinical measures and the outcome. Generalized multinomial logistic regression analyses were conducted to evaluate the joint effect of clinical factors on outcome. In the multivariable models, outcome was recoded into three groups and covariates included those factors significantly associated with outcome in the univariate analyses. For stratified analysis based on aneurysm location, outcome was grouped into four GOS categories: 0 (0_1, 0_2, and 0_3), 1, 2 to 3, and 4 to 5.

Results

Twenty-nine patients (59%) presented with SAH. In the 20 patients (41%) presenting without SAH, neurological status in five patients (25%) was Hunt and Hess Grade 0_1 (independent and able to work), in 11 (55%) it was Grade 0_2 (independent but unable to work), and in four patients (20%) it was Grade 0_3 (dependent and unable to work) (Table 1). In 13 (45%) of the 29 patients who presented with SAH, neurological status was Hunt and Hess Grade I or II and in 16 (55%) it was Grade III or IV.

After a mean follow-up duration of 33 months, outcome in 29 patients (59%) was reflected by a GOS score of 4 or 5, in eight (16%) by a score of 2 or 3, and in 12 patients (24%) by a score of 1 (death) (Fig. 2). Comparing outcome at 33 months with that documented during a mean follow-up period of 13 months,[5] we noticed an increase in mortality rate (GOS Score 1) from 10 to 25%, whereas the number of patients in the group with severe disability (GOS Score 3) decreased from 37 to 17%. The number of patients in the GOS Score 5 group (good outcome) increased from 28 to 59%.

The overall mean GOS score was 3.5. The nonparametric Kruskal–Wallis test for continuous variables revealed a significantly different outcome between the two age groups (age $\leq$ 55 years compared with $>$ 55 years; $p < 0.05$) (Fig. 3). The mean GOS score varied from 4.3 in patients less than 45 years of age to 2.0 in those greater than 70 years of age. Of the seven patients who were over age 70 years, five died (71%), and the outcome in two (29%) was reflected by a GOS score of 4 or 5. The median age in this series was 55 years. In patients less than age 55 years the mean GOS was 4.2, the mortality rate was 7% (two of 29), and a GOS score

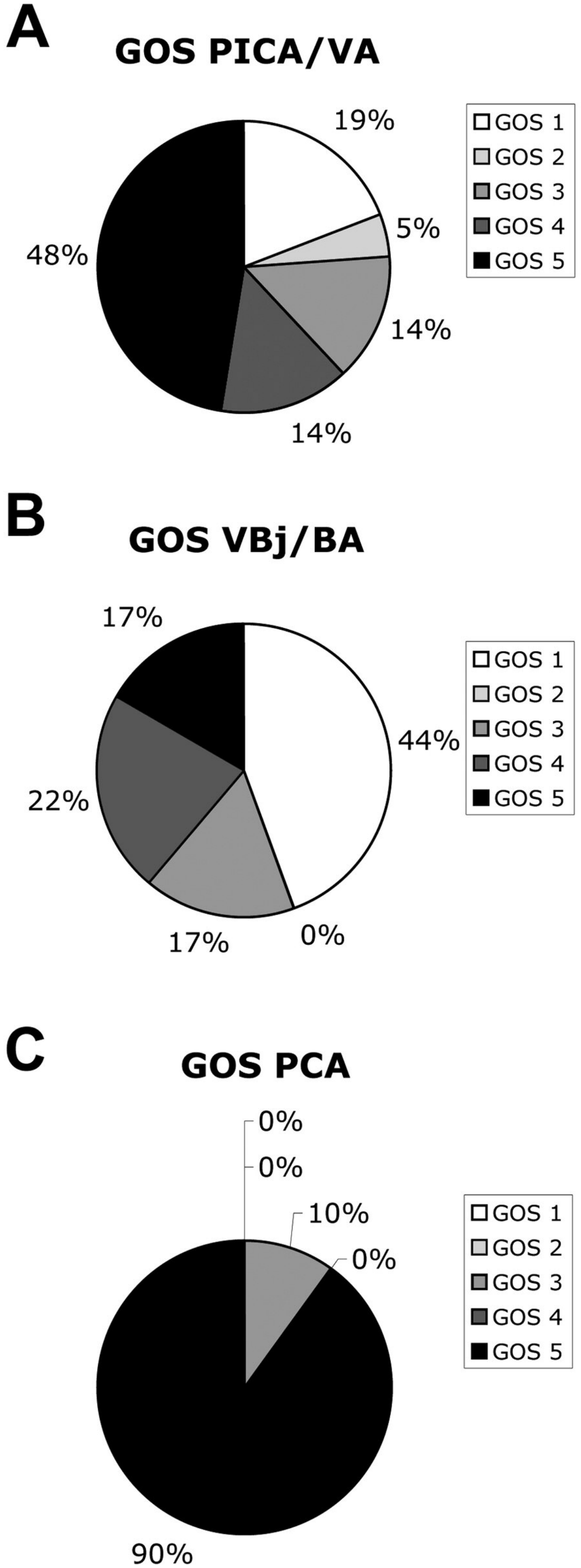

FIG. 2. Pie charts demonstrating outcomes after an average follow-up period of 33 months in patients with PICA–VA (A), BA–VBJ (B), and PCA (C) aneurysms (GOS Score 1, dead; 2, vegetative state; 3, severely disabled; 4, moderately disabled; and 5, good outcome).

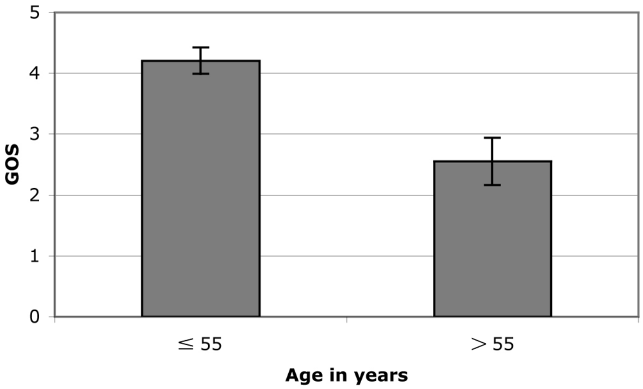

FIG. 3. Bar graph depicting the mean GOS score (± standard error of the mean [SEM]) in patients 55 years of age and younger and patients older than 55 years of age.

of 4 or 5 was achieved in 76% (22 of 29), whereas in patients greater than 55 years of age the mean GOS score was 2.6, the mortality rate was 50% (10 of 20), and a GOS score of 4 or 5 was achieved in 35% (seven of 25) (mean GOS scores $p < 0.05$; Fig. 3). Mean age did not differ between men and women.

Age at presentation was found to vary according to aneurysm locations. In the subgroups based on location, there was no relationship between age and outcome.

The overall chi-square test indicated an association between outcome and aneurysm location. The mean GOS scores varied from 4.8 in the PCA group to 3.7 in the VA–PICA and 2.7 in the BA–VBJ groups. We observed that PCA aneurysms were associated with significantly better outcome ($p < 0.05$). Favorable long-term outcomes (GOS Score 4–5) were obtained in 90% of patients with PCA aneurysms, 60% of those with VA–PICA aneurysms, and 39% of those with BA–VBJ aneurysms. The association in the partial chi-square test was mostly the result of differences in the number of patients with GOS Scores 1 and 5. The mean age of patients with PCA aneurysms was 42 years, significantly younger than that in patients with aneurysms in all other locations ($p < 0.05$). Patients with BA–VBJ aneurysms presented with SAH in only nine (50%) of 18 cases, compared to 17 (81%) of 21 cases in which VA aneurysms were present. In univariate analysis, patients with PCA aneurysms fared significantly better than those with BA–VBJ aneurysms ($p < 0.05$). None of the patients with PCA aneurysms died, whereas eight (44%) of 18 patients with BA–VBJ aneurysms died.

The mean aneurysm size was 19 mm (median 17 mm, range 6–42 mm). Only a minority (14%) of the aneurysms were smaller than 10 mm. Patients with giant (≥ 2.5-cm) aneurysms (23%) did not fare worse than those with smaller (< 2.5-cm) aneurysms ($p = 0.39$). Among the three groups with different aneurysm locations, the median size varied (Table 2) and the ranges differed widely. Analysis showed that aneurysm size was not significantly different among the three groups.

Fourteen (52%) of the 27 endovascularly treated patients presented with SAH as did 13 (68%) of the 19 surgically treated patients (Table 2 demonstrates the treatment modality stratified by aneurysm location). A comparison of the surgically and endovascularly treated groups showed that patients presenting with SAH did not have significantly different pretreatment Hunt and Hess scores. The chi-square test revealed that endovascular treatment was associated with worse outcome ($p = 0.05$, two-sided probability for trend). In the stratified analysis based on aneurysm location differences in treatment modality were noted ($p = 0.09$, Kruskal–Wallis test) between the three locations. Within the three location groups treatment modality was not associated with outcome ($p = 1.0$, Wilcoxon test)

The univariate analysis showed that, overall, patients with Hunt and Hess Grade I and II neurological status did better than those with Hunt and Hess Grade III and IV status ($p < 0.05$) (Fig. 4). Of the 16 patients with pretreatment Hunt and Hess Grade III to IV status, six (38%) had a good outcome (GOS Score 4–5), whereas of the 13 patients with Hunt and Hess I to II status, 11 (85%) had a good outcome (GOS Score 4–5). Of patients in whom a Hunt and Hess Grade 0 was present, outcome was not significantly different regardless of the following added functional subscale: Hunt and Hess Grade 0_1 (independent and able to work), Grade 0_2 (independent but unable to work), and Grade 0_3 (dependent and unable to work). The mean GOS scores at the long-term follow-up examination were 2.5, 3.5, and 4.0, for the PCA, VA–PICA, and BA–VBJ groups, respectively. Pretreatment clinical grades varied among the three aneurysm locations ($p = 0.04$, Kruskal–Wallis test). There was no association between pretreatment clinical grade and outcome within the location groups ($p = 0.59$, Wilcoxon test).

Comparison of our outcomes obtained at a mean follow-up duration of 13 months[5] and the current follow-up data representing a 33-month period revealed that of the 16 patients with severe disability at 13 months, two (13%) had died during the longer-term period and seven (44%) had recovered to GOS score of 4 or 5. Four of the 23 patients (17%) with GOS Score 4 or 5 outcome at a mean follow up of 13 months had died during the longer-term period; death was attributed to rehemorrhage in three patients (75%) and unknown cause in one (25%).

Posttreatment Angiography

Posttreatment angiograms (see examples in Figs. 5–10) were available for review in 35 (71%) of 49 patients. Five patients died before a follow-up angiogram could be obtained. In two of these patients complete thrombosis was confirmed at autopsy. Complete or virtually complete thrombosis was confirmed on early posttreatment angiography in 23 (66%) of 35 patients. A slight reduction in the size of the lesion was noted in one patient and there was a significant residual lesion in 10 patients. Of this group with residual aneurysms documented on posttreatment imaging, five (45%) of 11 patients died of SAH (three patients) or mass effect/brainstem ischemia (two patients) after treatment. None of the patients who died of posttreatment SAH had presented with SAH originally.

Summary of Deficits

Neurological status worsened within 30 days of treatment in 14 (29%) of 49 cases, in seven of which (50%) it resulted in death. Neurological complications were more prevalent in the BA–VBJ group (44%) than either the VA–

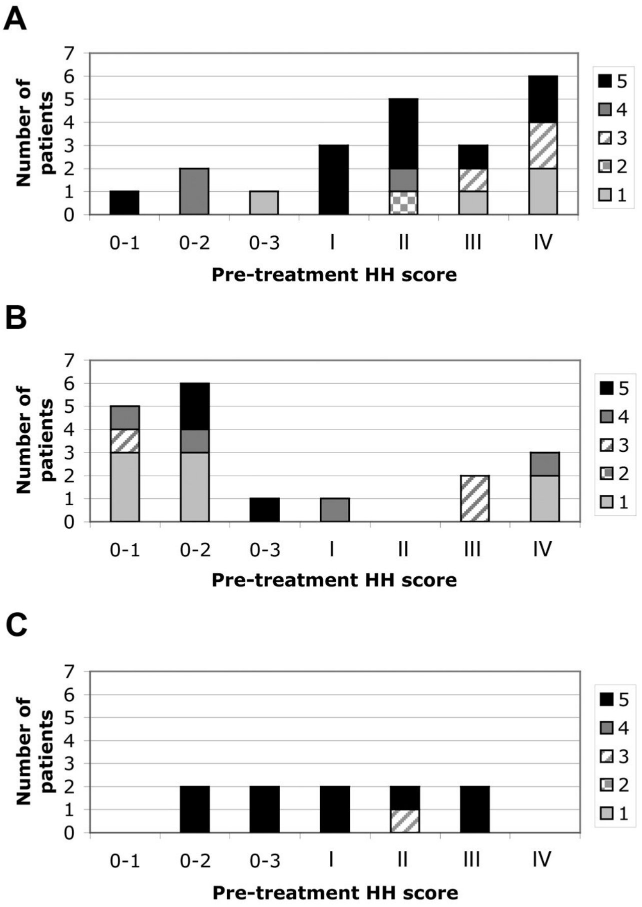

FIG. 4. Bar graphs showing the relationship between pretreatment conditions (using the Hunt and Hess grade [HH]) and outcome (using the GOS score [1–5]) for the three locations (A [PICA–VA], B [BA–VBJ], and C [PCA]). The GOS scores are shown on the y axis and the Hunt and Hess grades on the x axis; Grade 0-1, 0-2, and 0-3 correspond to Grade 0_1, 0_2, and 0_3 as defined in text.

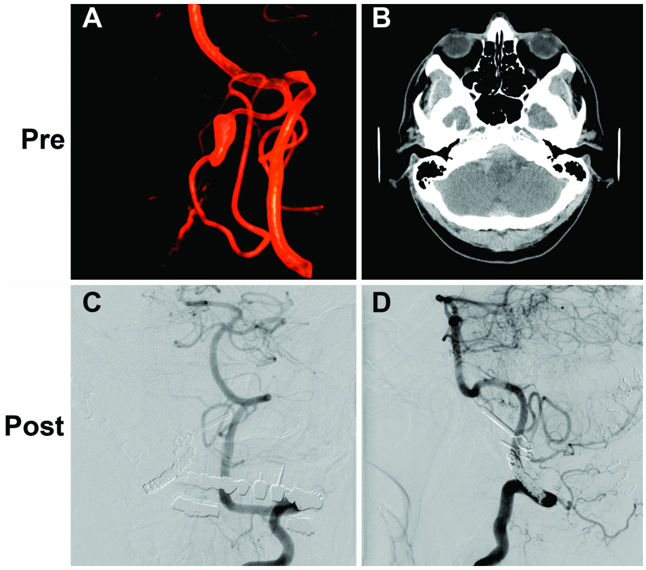

FIG. 5. Images obtained in a 52-year-old man who presented with SAH due to a ruptured 10 × 10–mm right VA aneurysm. A and B: The patient had undergone right VA coil occlusion after which he recovered. A follow-up angiogram revealed that the aneurysm had increased in size. The patient underwent a right occipital artery–PICA bypass and trapping of the aneurysm. His follow-up GOS score was 5 (good). C and D: Follow-up angiograms show that the aneurysm was no longer filled and that the bypass was intact. Post = postoperative; Pre = preoperative.

PICA (19%) or PCA (20%) groups. Overall, patients who experienced a neurological decline after treatment fared significantly worse during the long-term follow-up period ($p = 0.02$). In five patients this manifested as new or worsening of unilateral hemiparesis, in six as worsening of dysarthria and/or dysphagia, and in two as mild tetraparesis, and in one patient as transient aphasia. Neurological deficits were attributed to ischemia in seven patients and progression of brainstem compression in four. Severe vasospasm required prolonged intensive care unit stays for five (17%) of the 29 patients presenting with SAH. Medical complications included aspiration pneumonia in two patients and myocardial infarction in one. In four cases hydrocephalus required placement of a ventriculoperitoneal shunt.

Summary of Deaths

Recurrent SAH was confirmed to be the cause of death in three patients (occurring 1, 3, and 13 months after treatment), whereas increasing mass effect or brainstem ischemia was the cause of death in three patients (at 1, 2, and 8 months after treatment). In all of these patients there was residual aneurysm filling (partial thrombosis) after treatment. One patient died as a result of an unrelated cardiac event 7 months after treatment, and in four patients the exact cause of death at 2, 3, 4, and 9 months could not be determined. In eight patients the BA or VBJ was the location of the aneurysm, and in three patients the VA was involved. Most deaths (eight of 11) occurred early, within the first 6 months after treatment.

Discussion

Natural History

In the evaluation of the efficacy and safety of therapies, it is important compare treatment outcome with the expected natural history without treatment. Data on the natural history of untreated posterior circulation fusiform aneurysms are limited. In general the natural history is thought to be unfavorable,[3,15] although more benign courses have been described.[6,19] In the series described by Mizutani et al.[15] early aneurysm rupture occurred before treatment in 30 (71%) of the 42 patients mostly with VA dissecting aneurysms and presenting with SAH.[15] Eventually 29 (69%) of the 42 patients underwent treatment. Most of the remaining 13 patients (11 [85%] of 13), who received conservative treatment due to poor clinical condition or anatomical constraints, died.[15]

Attempts to identify higher- and lower-risk groups have resulted in a number of classifications. A classification proposed by Mizutani et al.[16] was based on correlating imaging findings, clinical features, and intraoperative appear-

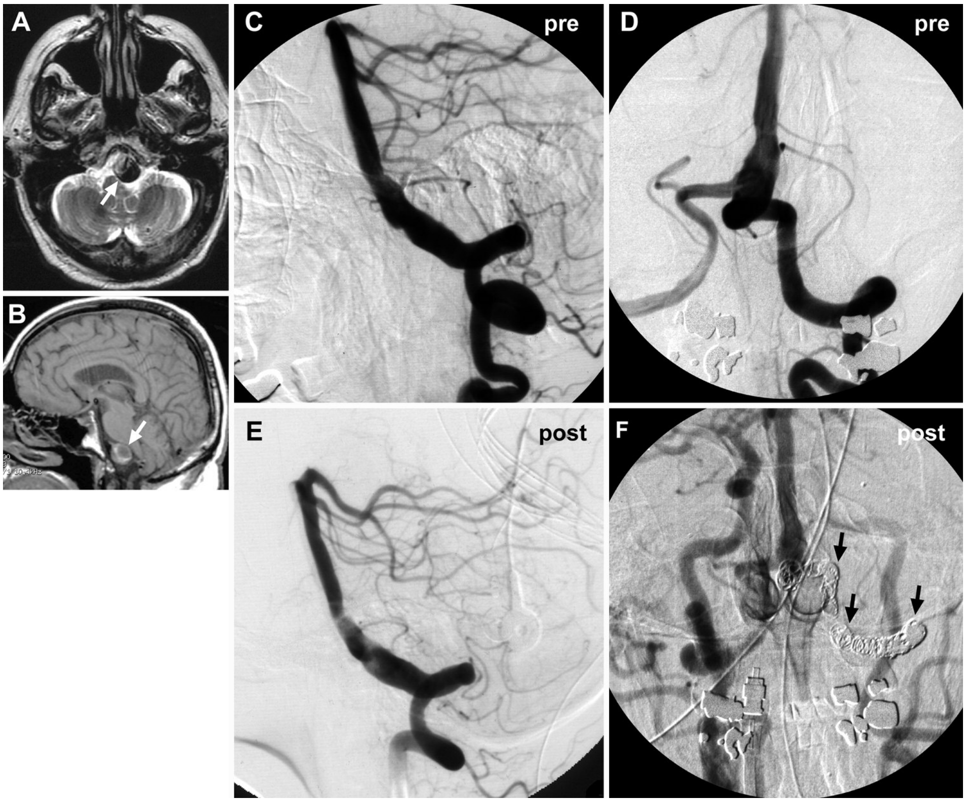

FIG. 6. Magnetic resonance (MR) images and angiograms acquired in a 71-year-old man who presented with gradually developing neck pain, right arm numbness, dysphagia, and an unsteady gait. The MR images (axial T_2-weighted [A] and sagittal T_1-weighted [B]) reveal a 2-cm atheromatous fusiform aneurysm of the left VA reaching to the VBJ and compressing the brainstem. The left VA is occluded distal to the common PICA–AICA origin (C and D). This patient did well directly after treatment (E and F) and at long-term follow up. *Arrows* indicate site of occlusion.

ance to pathological findings, although pathology reports were only available in a portion of the 85 patients. Four types were distinguished. Type I was the classic dissecting aneurysm that most frequently presented with an SAH, had a so-called pearl-and-string appearance on angiography, and exhibited a high rebleeding rate. In Type II, or segmental ectasia, the aneurysm was mostly asymptomatic and had smooth contours on angiography with no thrombus. None of the patients in this group exhibited signs of clinical deterioration. Type III was the dolichoectatic dissecting aneurysm, which presented with cranial nerve deficits, hydrocephalus, or brainstem dysfunction. Angiography generally revealed luminal thrombus and aneurysm growth. Hemorrhage was seen frequently during follow up, accounting for the observed 50% mortality rate. Finally, the Type IV, or saccular aneurysm arising from the arterial trunk, presented with SAH and had a high mortality rate due to rebleeding (three of four patients). Although no pathology data were available for most of our 49 patients, these were mostly Mizutani Types I and III lesions, which have high rehemorrhage rates with associated morbidity and death.[16]

In the study published by Flemming et al.,[6] only five (3%) of 159 patients presented with SAH. Dissecting aneurysms were excluded from their study and 40% of patients had symptoms unrelated to the aneurysm. Differentiation between dissecting and nondissecting nonsaccular intracranial aneurysms is not always possible based on imaging. In ultrasonography studies specific abnormalities that are diagnostic for dissection aneurysms such as intramural hematomas or intimal flaps have been reported to present in less than one third of cases.[24]

In a series of patients with unruptured fusiform aneurysm, aneurysm size (diameter $\geq$ 10 mm) was found to be a risk factor for future growth, as measured on computed tomography angiography.[14] The authors of a study of patients with mostly unruptured nonsaccular VB aneurysms demonstrated that aneurysms of 10 mm in diameter or greater enlarged over time and that this feature was predictive of future rupture.[6] In 86% of the patients in our study the diameter of the aneurysm was 10 mm or larger (mean 19 mm, median 17 mm), which suggests greater risk of future rupture.

Morphology Features

In a study on vertebrobasilar dissecting aneurysms published by Pozatti and colleagues,[19] patients were included with angiographic evidence of the pearl-and-string sign in six (38%) of 16 cases, a double lumen in one (0.6%) of 16 cases, and fusiform dilation in four (25%) of 16 patients. Two (50%) of the four patients with fusiform dilations presented with SAH, and without treatment they all fared well. Data reported by other authors, however, especially on patients presenting with SAH, strongly contradict this benign

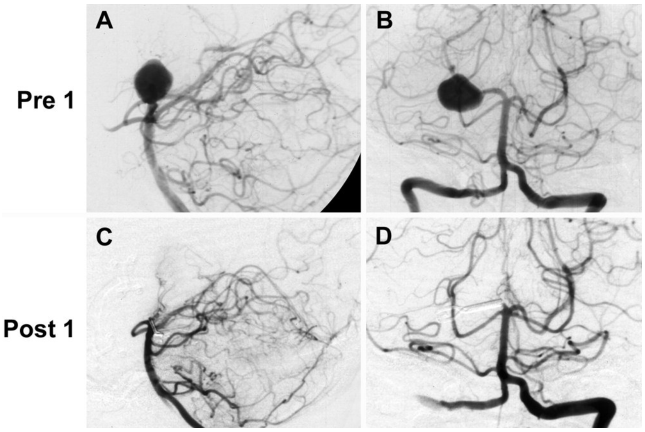

FIGS. 7 AND 8. Angiograms acquired in a 32-year-old woman who presented with headaches. Left VA injection anteroposterior (AP; Fig. 7A) and lateral (Fig. 7B) images reveal a 2-cm right PCA fusiform aneurysm not visible on the right common carotid artery (CA) image (Fig. 8A). After passing the balloon test occlusion (BTO), a deliberate proximal P_2 clip occlusion was performed via an orbitozygomatic approach after induction of mild hypothermia and during electrophysiological monitoring. The patient fared well. The postoperative left VA angiograms (lateral [Fig. 7C] and AP [Fig. 7D]) reveal occlusion of the aneurysm. The lateral CA angiogram (Fig. 8C) reveals filling of the right PCA and a small residual aneurysm base through a small right posterior communicating artery (PCoA). The patient's headaches resolved but recurred 1 year later when follow-up lateral CA angiography revealed recanalization of the aneurysm base through an enlarged PCA (Fig. 8B). Fourteen months after the first surgery, the patient underwent a second surgical procedure. First the distal right PCoA was subjected to test occlusion; after it was clear that this was tolerated, the distal right PCoA was permanently occluded. The final lateral right CA image (Fig. 8D) confirms complete thrombosis of the aneurysm. This patient did well (GOS score of 5) at follow up.

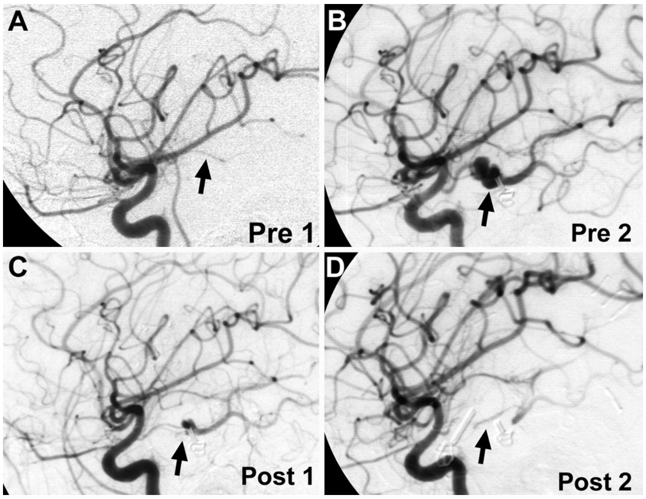

clinical course, with 30[4] to 85%[15] rebleeding rates in untreated patients. Rebleeding in the former study occurred most frequently during the acute stage[4] for which an explanation was found in blood clot development over time in neuropathological studies.[26] Late rehemorrhage has been described up to 11 years after initial presentation.[20] In our study, posttreatment SAH occurred after incomplete thrombosis in three patients at 1, 3, and 13 months. None of these patients originally presented with SAH. Likewise, in Flemming and associates' study[6] none of the nine patients in whom rebleeding occurred during the follow-up period had originally presented with SAH.

The risk of hemorrhage has been related to aneurysm morphological features. Transitional and fusiform aneurysms have been found to be associated with increased hemorrhage rates.[6] "Transitional" has been defined as a uniform aneurysmal dilation of an entire arterial segment greater than 1.5-fold its normal size with superimposed dilation of a portion of the involved arterial segment.[13] The risk of (recurrent) posterior circulation ischemia was found to exceed the risk of rehemorrhage in this series of mostly asymptomatic unruptured aneurysms, and cerebral ischemia and BA thrombosis were found to be the most common causes of death.[6] The different anatomical locations in the present study were associated with differences in presentation (hemorrhage compared with mass effect) and age at presentation. Two thirds of the patients with BA–VBJ aneurysms presented with symptoms of mass effect, whereas most patients with PCA and VA aneurysms presented with an SAH (Table 2).

The effects of local vessel curvature and aneurysm size on aneurysm hemodynamics was studied in a computational fluid dynamic analysis performed by Hoi et al.[8] They found that a high degree of parent vessel curvature and wide-necked aneurysm were associated with increased pressure, wall sheer stress, and aneurysm growth. Although the authors of in vitro studies have suggested that partial coil therapy and stent placement dramatically decreased the slipstream, reducing the risk of future rupture,[8,10] partial thrombosis failed to protect against late-onset neurological complication in our present series and previous endovascular[12] and surgical[23] series.

Clinical Outcome After Treatment

Given the generally poor natural history of untreated symptomatic posterior circulation fusiform aneurysms larger than 10 mm, we consider our overall long-term good outcome (GOS Score 4 to 5) of 90% for PCA and 62% for VA to reflect favorable clinical results. As expected the results for BA and VBJ aneurysms were less favorable: good outcome (GOS 4–5) in 39% and death in 44%.

Twenty-seven percent (three of 11) of deaths in our series at long-term follow up were caused by SAH and were confirmed on imaging and/or at autopsy. Of the 16 patients who underwent treatment in Flemming and colleagues' series,[6] three (19%) suffered rebleeding after treatment compared with six (4%) of 143 untreated patients. Data on the clinical outcome after SAH in these nine patients was not provided. In the study by Mizutani et al.[15] of 42 patients with dissecting aneurysms presenting with SAH, 29 patients (69%) underwent treatment. Outcome was good in 14 patients (52%) and poor in 11 (38%); four died (14%).[1] Leibowitz and colleagues[12] divided a series of consecutive fusiform aneurysms in patients undergoing endovascular parent vessel occlusion into two groups based on clinical presentation, suspected neuropathological findings, and clinical course. One group was composed of patients with acutely dissecting aneurysms that typically presented with SAH; the other group contained patients with chronic fusiform aneurysms, which gradually enlarged and presented with mass effect or

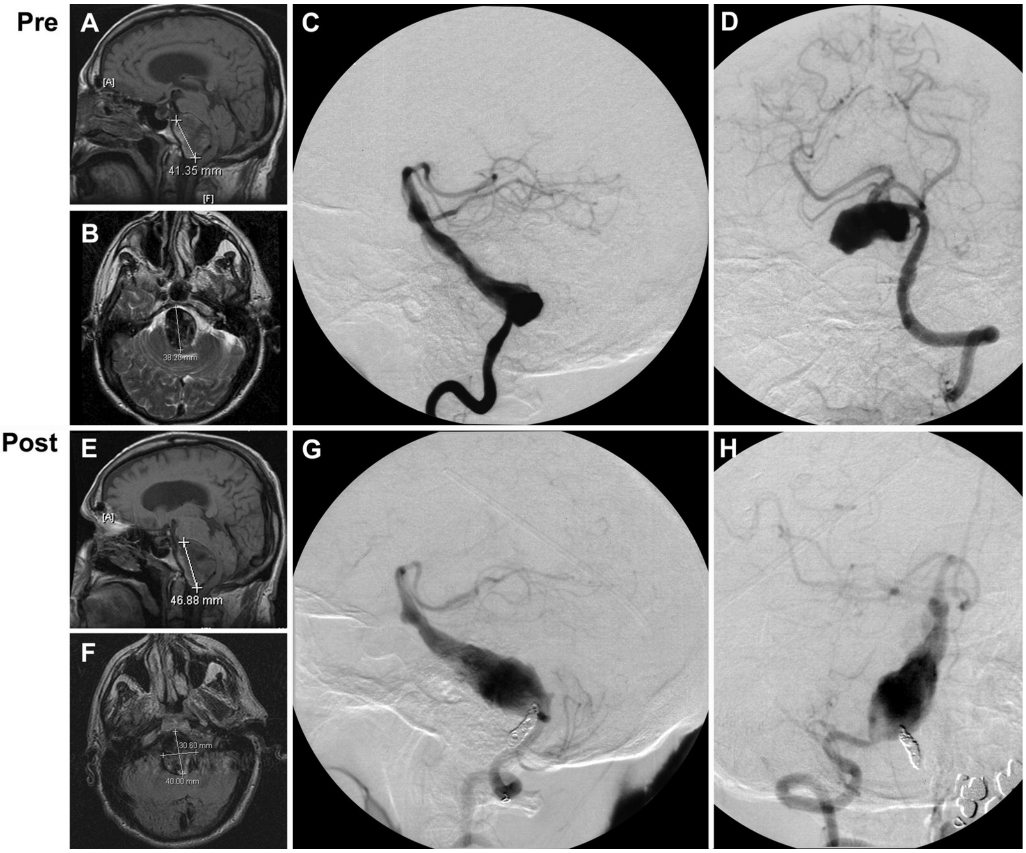

FIG. 9. Imaging studies obtained in a 66-year-old man who presented with multiple episodes of disequilibrium, weakness, and numbness and was found to harbor a large fusiform VBJ aneurysm causing TIAs, obstructive hydrocephalus, and brainstem compression, as shown on sagittal (A) and axial (B) MR images and left VA angiograms (lateral [C] and AP [D]). A ventriculoperitoneal shunt was placed. After BTO of the left VA was tolerated, the distal left VA was permanently occluded by placing coils. The aneurysm was still filling from the right VA. Increased mass effect of the aneurysm on the brainstem is demonstrated on follow-up sagittal (E) and axial (F) MR images. Right AP (G) and lateral (H) VA angiograms confirm the increased size. This patient did well initially but died 13 months later at home of an SAH (GOS score of 1).

infarction. Parent vessel occlusion in the acutely dissecting aneurysm group resulted in angiographically confirmed complete thrombosis whereas in the second group complete thrombosis was neither attempted nor obtained. The outcomes were significantly worse in patients with gradually enlarging aneurysms presenting with mass effect and with incomplete thrombosis after treatment ($p < 0.02$), with four (57%) of seven patients dying compared with no deaths in the six patients in the acute dissecting aneurysm group with complete thrombosis after treatment. Death was attributed to continued mass effect on the brainstem in two patients and unrelated cardiac causes in the other two patients.

In the long-term outcome series of 201 vertebrobasilar aneurysms reported by Steinberg et al.,[23] patients were treated with Hunterian ligation, and outcome was good to excellent in 73%, poor in 3%, and 24% of patients died. A comparison of the present data and that of the aforementioned series published in 1993 reveals an important difference in aneurysm size. In the 1993 series the majority (174 [87%] of 201) of aneurysms were giant (> 2.5 cm) in size whereas in the present series a minority (11 [22%] of 49) were giant in size. In the Steinberg et al. population all arterial ligations were performed surgically using clips or tourniquets, whereas 27 (55%) of our 49 patients underwent endovascular therapy. Authors of other series reported that patients presenting with mass effect and brainstem compression fared significantly worse than those presenting with SAH.[7,12,23] In the present study, there was no significant difference in outcome between those patients with and without an SAH presentation ($p = 0.18$) (Fig. 11).

Lower cranial nerve palsy was found to be a major cause of morbidity in a treatment series of patients with VA and PICA aneurysms, increasing the duration of intensive care unit stay and the incidence of nosocomial pneumonia. Seventy-five percent of lower cranial nerve palsies were found to resolve completely within 6 months.[1] Worsening of the neurological condition posttreatment involved cranial nerve dysfunction in four of 49 patients (8%) in our present population of patients.

In a multivariate analysis, we found that age greater than 55 years was strongly associated with worse outcome ($p < 0.001$). When stratified based on location, this effect of age on outcome was removed. Patients with BA–VBJ aneurysm were older, had larger aneurysms, presented with mass effect and/or ischemic symptoms, and fared worse. In their clinicopathological study of dissecting vertebro-

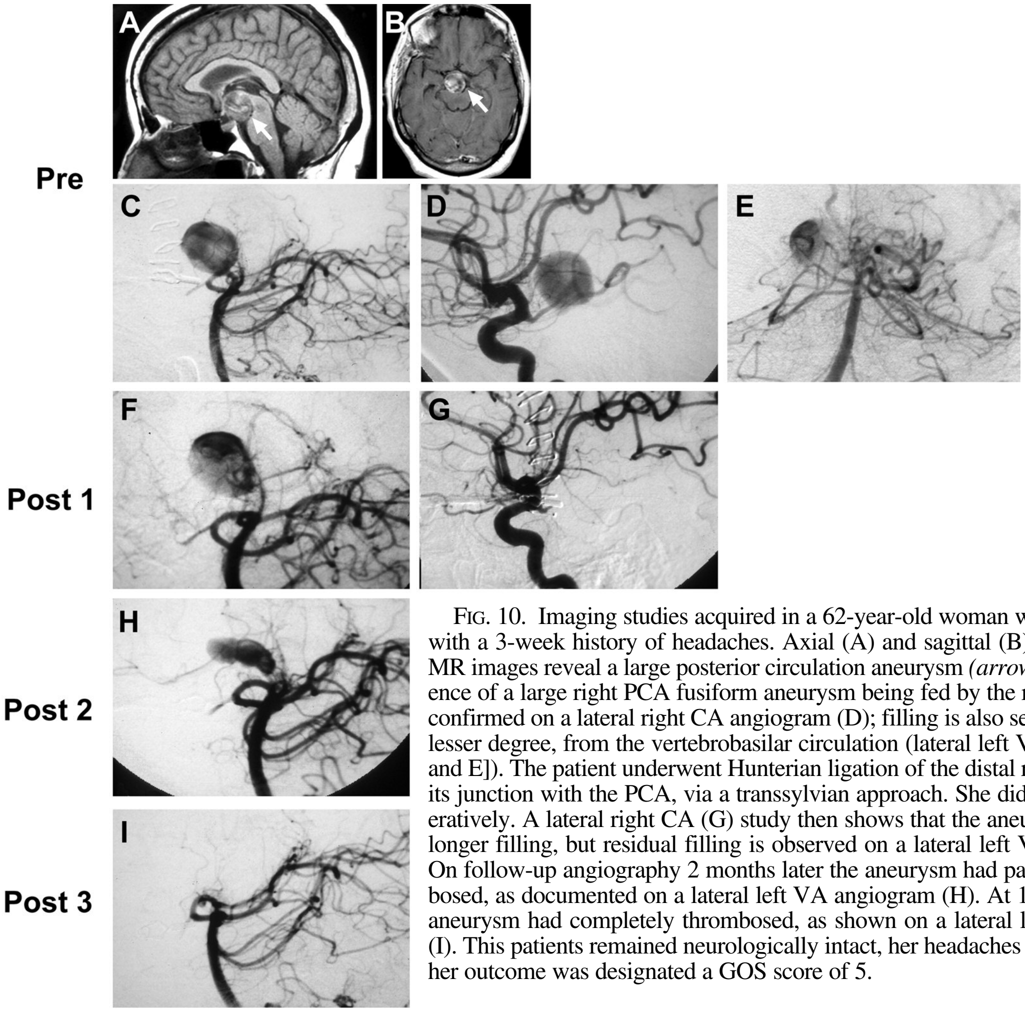

FIG. 10. Imaging studies acquired in a 62-year-old woman who presented with a 3-week history of headaches. Axial (A) and sagittal (B) T_1-weighted MR images reveal a large posterior circulation aneurysm *(arrows)*. The presence of a large right PCA fusiform aneurysm being fed by the right PCoA is confirmed on a lateral right CA angiogram (D); filling is also seen, though to lesser degree, from the vertebrobasilar circulation (lateral left VA studies [C and E]). The patient underwent Hunterian ligation of the distal right PCoA at its junction with the PCA, via a transsylvian approach. She did well postoperatively. A lateral right CA (G) study then shows that the aneurysm was no longer filling, but residual filling is observed on a lateral left VA study (F). On follow-up angiography 2 months later the aneurysm had partially thrombosed, as documented on a lateral left VA angiogram (H). At 11 months the aneurysm had completely thrombosed, as shown on a lateral left VA study (I). This patients remained neurologically intact, her headaches resolved, and her outcome was designated a GOS score of 5.

basilar aneurysms, Sasaki et al.[21] noted two groups distinct in age at presentation, clinical course, and aneurysm location. One group (mean age at presentation 38 years) had brainstem infarctions resulting from subintimal dissections at the VBJ. In the other group (mean age at presentation 50 years) SAH due to a subadventitial VA dissection was the cause of death.

Aneurysm Location

Patients with PCA aneurysms in the present study were younger, more often underwent surgery, and had better outcomes, whereas patients with BA–VBJ aneurysms were older and had poorer outcomes. Favorable long-term outcome was achieved in 90% of patients with PCA aneurysms, 60% of those with VA–PICA aneurysms, and in only 39% of those with BA–VBJ aneurysms. The BA location was also found to be predictive of a worse outcome in a study published by Seifert et al.[22] In 24 BA and VBJ aneurysms, they found that patients with aneurysms smaller than 10 mm fared better. Brainstem perforating vessels incorporated into the aneurysm sac can complicate treatment of fusiform BA aneurysms. Perforating artery occlusion due to thrombus may already have occurred at the time of diagnosis with collateral supply to the perforating artery territory.[25] Three cases of basilar trunk endovascular occlusion have been reported in cases of giant and fusiform aneurysms of the BA with good outcome.[25] Good results were reported in 72% of the 46 BA cases managed surgically in Steinberg and associates' study[23] (24% mortality rate), whereas only 26% of the patients were reported to make a good recovery (mortality rate 53%) in a report by Amin-Hanjani and colleagues.[2] Yoshimoto et al.[27] reported favorable outcomes in four (40%) of 10 patients with BA dissection aneurysms, of whom four presented with SAH. Two of the four patients who presented with SAH underwent endovascular treatment. One of the two patients died and the other had a good recovery. In our present series 10 (56%) of the 18 patients with BA–VBJ aneurysms were treated endovascularly; seven (39%) fared well at long-term follow up, and eight died (44%). The complexity of treatment of this particular type of aneurysm has been illustrated by the report of a staged bilateral VA occlusion with extracranial–intracranial bypass for large fusiform proximal basilar trunk aneurysm.[18] In this case treatment failed to prevent further expansion of the aneurysm, necessitating surgical decompression and a trapping procedure. The patient eventually died of BA thrombosis.[18]

Andoh and coworkers[3] reported on a series of 10 patients with fusiform VA aneurysms who presented with SAH;

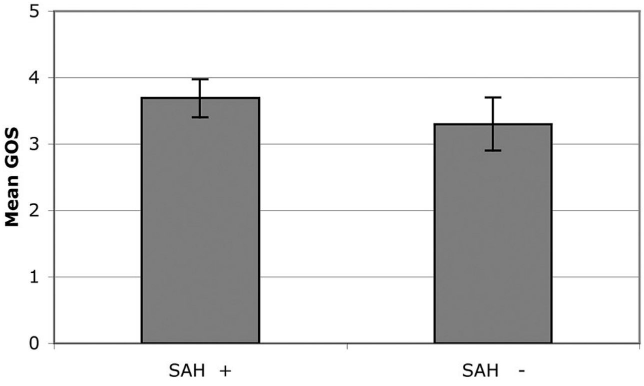

FIG. 11. Bar graph demonstrating mean GOS scores (± SEM) obtained in patients presenting with (+) and without (−) SAH.

outcome was graded as good in eight (80%) of 10 patients and two (20%) died. These rates compare with those in the present series of patients with VA aneurysm; there were nine (60%) of 15 patients with GOS scores of 4 to 5 and a 21% mortality rate (12 [80%] of 15 of whom presented with SAH).

In one patient (2%) in the present study the aneurysm involved the PICA. This patient presented with SAH, had a pretreatment Hunt and Hess Grade IV SAH because of a rehemorrhage, underwent aneurysm trapping, and recovered completely (GOS score of 5). Nussbaum et al.[17] reported on seven surgically treated patients with fusiform PICA aneurysms who had all presented with SAH. Six aneurysms were trapped with distal revascularization of the occipital artery, and one patient underwent excision of the aneurysm and end-to-end anastomosis. Outcome was good in six (86%) of these patients and poor in one.

Hunterian proximal ligation or endovascular parent vessel occlusion of VBJ aneurysms has resulted in virtually complete thrombosis in 46 to 84% of patients in previous studies[12,23] and 60% in the present series. Incomplete thrombosis was associated with a poor subsequent natural history due to rebleeding and increased mass effect or brainstem ischemia in our study and prior reports.[12,23]

Conclusions

We present a series of patients treated for symptomatic posterior circulation fusiform aneurysms. Technical advancements and growing surgical experience with endovascular techniques have resulted in an increased percentage of patients undergoing endovascular therapies. Potentially important differences in inclusion criteria, patient and aneurysm characteristics, and treatment paradigms complicate direct comparisons among series. The low prevalence and variable clinical course of these aneurysms may explain contradictory observations of natural history and recommendations for therapy that appear in the literature. In our series of 49 patients, three different location groups (VA–PICA, PCA, and BA–VBJ) were distinguished. Age less than or equal to 55 years was strongly associated with more favorable outcome. Once the strong effect of age was taken into account in multinomial models, the effect of other factors such as pretreatment clinical grade, treatment modality, and residual aneurysm on outcome did not remain statistically significant. However, stratification by aneurysm location removed the effect of age on outcome. In fact, the three posterior circulation fusiform aneurysm locations (PCA, VA–PICA, and BA–VBJ) appear to represent different disease entities, affecting patients at different ages and with distinct patterns of presentation. Favorable overall long-term outcome can be achieved in 90% of patients with PCA aneurysms, 60% of those with VA–PICA aneurysms, and 39% of those with BA–VBJ aneurysms when using endovascular and surgical techniques. Treatment of fusiform aneurysms resulted in immediate worsening of the neurological condition in 29% of patients, but overall long-term results were good in 58% of patients. The natural history for patients with incomplete aneurysm thrombosis despite treatment was poor, with 38% of these patients dying of SAH or brainstem compression/ischemia. Greater knowledge of the natural history and pathophysiology of posterior circulation fusiform aneurysms and critical evaluation of the effects of current treatments on clinical outcome may lead to an improvement of outcome in patients harboring these challenging vascular lesions.

Acknowledgments

We thank Beth Hoyte for preparation of the figures, David Schaal for editorial assistance, and Paige Bracci for performing the statistical analysis.

References

1. Al-khayat H, Al-Khayat H, Beshay J, Manner D, White J: Vertebral artery-posteroinferior cerebellar artery aneurysms: clinical and lower cranial nerve outcomes in 52 patients. **Neurosurgery 56:**2–11, 2005
2. Amin-Hanjani S, Ogilvy CS, Buonanno FS, Choi IS, Metz LN: Treatment of dissecting basilar artery aneurysm by flow reversal. **Acta Neurochir (Wien) 139:**44–51, 1997
3. Andoh T, Shirakami S, Nakashima T, Nishimura Y, Sakai N, Yamada H, et al: Clinical analysis of a series of vertebral aneurysm cases. **Neurosurgery 31:**987–993, 1992
4. Aoki N, Sakai T: Rebleeding from intracranial dissecting aneurysm in the vertebral artery. **Stroke 21:**1628–1631, 1990
5. Coert BA, Dodd RL, Marcellus ML, Do HM, Marks MM, Chang SD, et al: Posterior circulation fusiform aneurysms: results of surgical, endovascular, and combined treatment. **Annual Meeting of AANS Program 73:**1043, 2005 (Abstract)
6. Flemming KD, Wiebers DO, Brown RD Jr, Link MJ, Nakatomi H, Huston J III, et al: Prospective risk of hemorrhage in patients with vertebrobasilar nonsaccular intracranial aneurysm. **J Neurosurg 101:**82–87, 2004
7. Gobin YP, Viñuela F, Gurian JH, Guglielmi G, Duckwiler GR, Massoud TF, et al: Treatment of large and giant fusiform intracranial aneurysms with Guglielmi detachable coils. **J Neurosurg 84:** 55–62, 1996
8. Hoi Y, Meng H, Woodward SH, Bendok BR, Hanel RA, Guterman LR, et al: Effects of arterial geometry on aneurysm growth: three-dimensional computational fluid dynamics study. **J Neurosurg 101:**676–681, 2004
9. Hunt WE, Hess RM: Surgical risk as related to time of intervention in the repair of intracranial aneurysms. **J Neurosurg 28:** 14–20, 1968
10. Imbesi SG, Kerber CW: Analysis of slipstream flow in a wide-necked basilar artery aneurysm: evaluation of potential treatment regimens. **AJNR Am J Neuroradiol 22:**721–724, 2001

11. Jennett B, Bond M: Assessment of outcome after severe brain damage. **Lancet 1:**480–484, 1975
12. Leibowitz R, Do HM, Marcellus ML, Chang SD, Steinberg GK, Marks MP: Parent vessel occlusion for vertebrobasilar fusiform and dissecting aneurysms. **AJNR Am J Neuroradiol 24:** 902–907, 2003
13. Mangrum WI, Huston J III, Link MJ, Wiebers DO, McClelland RL, Christianson TJH, et al: Enlarging vertebrobasilar nonsaccular intracranial aneurysms: frequency, predictors and clinical outcome of growth. **J Neurosurg 102:**72–79, 2005
14. Matsubara S, Hadeishi H, Suzuki A, Yasui N, Nishimura H: Incidence and risk factors for the growth of unruptured cerebral aneurysms: observation using serial computerized tomography angiography. **J Neurosurg 101:**908–914, 2004
15. Mizutani T, Aruga T, Kirino T, Miki Y, Saito I, Tsuchida T: Recurrent subarachnoid hemorrhage from untreated ruptured vertebrobasilar dissecting aneurysms. **Neurosurgery 36:**905–913, 1995
16. Mizutani T, Miki Y, Kojima H, Suzuki H: Proposed classification of nonatherosclerotic cerebral fusiform and dissecting aneurysms. **Neurosurgery 45:**253–260, 1999
17. Nussbaum ES, Mendez A, Camarata P, Sebring L: Surgical management of fusiform aneurysms of the peripheral posteroinferior cerebellar artery. **Neurosurgery 53:**831–835, 2003
18. O'Shaughnessy BA, Getch CC, Bendok BR, Parkinson RJ, Batjer HH: Progressive growth of a giant dolichoectatic vertebrobasilar aneurysm after complete Hunterian occlusion of the posterior circulation: case report. **Neurosurgery 55:**1223, 2004
19. Pozzati E, Andreoli A, Limoni P, Casmiro M: Dissecting aneurysms of the vertebrobasilar system: study of 16 cases. **Surg Neurol 41:**119–124, 1994
20. Sagoh M, Hirose Y, Murakami H, Akaji K, Katayama M, Hayashi T: Late hemorrhage from persistent pseudoaneurysm in vertebral artery dissection presenting with ischemia: case report. **Surg Neurol 52:**480–484, 1999
21. Sasaki O, Ogawa H, Koike T, Koizumi T, Tanaka R: A clinicopathological study of dissecting aneurysms of the intracranial vertebral artery. **J Neurosurg 75:**874–882, 1991
22. Seifert V, Raabe A, Stolke D: Management-related morbidity and mortality in unselected aneurysms of the basilar trunk and vertebrobasilar junction. **Acta Neurochir (Wien) 143:**343–349, 2001
23. Steinberg GK, Drake CG, Peerless SJ: Deliberate basilar or vertebral artery occlusion in the treatment of intracranial aneurysms. Immediate results and long-term outcome in 201 patients. **J Neurosurg 79:**161–173, 1993
24. Sturzenegger M, Mattle HP, Rivoir A, Baumgartner RW: Ultrasound findings in carotid artery dissection: analysis of 43 patients. **Neurology 45:**691–698, 1995
25. Wenderoth JD, Khangure MS, Phatouros CC, ApSimon HT: Basilar trunk occlusion during endovascular treatment of giant and fusiform aneurysms of the basilar artery. **AJNR Am J Neuroradiol 24:**1226–1229, 2003
26. Yamaura A, Watanabe Y, Saeki N: Dissecting aneurysms of the intracranial vertebral artery. **J Neurosurg 72:**183–188, 1990
27. Yoshimoto Y, Hoya K, Tanaka Y, Uchida T: Basilar artery dissection. **J Neurosurg 102:**476–481, 2005

Manuscript submitted July 22, 2005.

Accepted October 3, 2006.

This study was supported in part by funding from the William Randolph Hearst Foundation, Bernard and Ronnie Lacroute, John and Dodie Rosekrans, and Russell and Beth Siegelman.

Current address for Dr. Coert: Department of Neurosurgery, AMC Amsterdam, The Netherlands.

Address reprint requests to: Gary K. Steinberg, M.D., Ph.D., Department of Neurosurgery, Stanford University, 300 Pasteur Drive, Room R200, Stanford, California. email: gsteinberg@stanford.edu.
