# Supplementary material for: Management strategies and outcomes of basilar trunk aneurysms: a systematic review and meta-analysis
Source: Langenbecks Arch Surg. 2026 Jan 20;411(1):71. doi: 10.1007/s00423-025-03959-3 (PMC12852281; doi:10.1007/s00423-025-03959-3)
Supplement: Supplementary file 1 — Supplementary file1 (ZIP 55327 KB) [file 423_2025_3959_MOESM1_ESM.zip › literature.zip!/meta/higa2008.pdf]

Neurosurg Rev (2009) 32:181–191
DOI 10.1007/s10143-008-0163-3

ORIGINAL ARTICLE

Basilar artery trunk saccular aneurysms: morphological characteristics and management

Takashi Higa · Hiroshi Ujiie · Koichi Kato · Hiroyasu Kamiyama · Tomokatsu Hori

Received: 8 February 2008 / Revised: 26 June 2008 / Accepted: 4 August 2008 / Published online: 13 September 2008
© Springer-Verlag 2008

Abstract The purpose of this retrospective study was to report the morphological characteristics and results of surgical and endovascular treatment of basilar artery (BA) trunk saccular aneurysms. Twenty-two patients with 22 BA trunk saccular aneurysms underwent surgery including endovascular intervention. In this series, BA trunk aneurysms showed characteristic features such as so-called lateral aneurysm (41%), multiple aneurysms (32%), including two de novo aneurysms, and various vascular anomalies. Eleven craniotomies for neck clipping were performed for 11 ruptured aneurysms. However, in one of these cases, we abandoned neck clipping, because of concern for neck tearing, and embolized it later. Five ruptured and five unruptured aneurysms were successfully treated by endovascular surgery. Another one incompletely embolized aneurysm had grown to a huge size, and the patient underwent a Hunterian ligation with a flow reconstruction. The unusually high incidence of various associated vascular anomalies suggests that focal wall weakness must be based on the mechanism of aneurysm initiation. Most patients presented with subarachnoid hemorrhage. The pretreatment neurological state was predictive for clinical outcome. And, clinical outcomes in this series were not affected by the choice of treatment. However, considering that three of 11 surgical cases needed subsequent treatment, endovascular surgery should be considered as a first choice.

T. Higa (✉) · H. Ujiie · K. Kato · T. Hori
Department of Neurosurgery, Neurological Institute,
Tokyo Women's Medical University,
8-1 Kawada-cho,
Shinjuku-ku, Tokyo 162-8666, Japan
e-mail: thiga@nij.twmu.ac.jp

H. Kamiyama
Department of Neurosurgery, Asahikawa Red Cross Hospital,
Asahikawa, Japan

Keywords Intracranial aneurysm · Subarachnoid hemorrhage · Surgical approach · Hunterian ligation · Endovascular surgery · Embolization

Introduction

Aneurysms of the basilar artery (BA) trunk are rare and among the most difficult to manage surgically [1, 2, 6, 9, 30, 36, 39, 41]. Anatomically, the BA trunk is located in the depth of the valley surrounded by the bilateral petrous bones. Surgical access to this area is hampered by a dense collection of vital cranial nerves and perforating arteries to the brain stem [2]. Furthermore, the difficulty in treating these aneurysms is compounded by their rarity, which limits the acquisition of experience in treating these lesions. As a result, the rarity and unique morphological features of BA trunk aneurysms make it difficult to individually tailor the treatment strategy. However, an endovascular approach to this area is comparatively easy because of the simple access to the BA. Thus, the first choice of treatment has changed in the last 10 years.

The objective of this retrospective analysis was investigation of the morphological characteristics of the BA trunk saccular aneurysms, evaluation of surgical strategies and results of their management, and review of the relevant literature.

Materials and methods

The clinical data for this study were obtained from a retrospective analysis of 22 patients with 22 BA trunk saccular aneurysms who underwent surgical treatment including endovascular surgery at the Department of

Neurosurgery, Neurological Institute, Tokyo Women's Medical University and affiliated hospitals between 1984 and 2007 [20, 53]. In this series, fusiform aneurysms (three cases) were excluded. There were 18 women (82%) and four men (18%), ranging from 20 to 88 years with a mean age of 58.1 years.

Sixteen patients presented with subarachnoid hemorrhage (SAH), three with coexisting aneurysms which developed SAH, one with a mass effect on the brain stem, while two patients were incidentally discovered.

Before 1996 (cases 1 to 11), we mainly performed direct surgery. All surgeries were performed or supervised by the senior author (TH: Tomokatsu Hori). In 1996, we introduced endovascular surgery, and coil embolization was selected as the first choice of treatment for BA trunk saccular aneurysms. Since then, direct surgery has been performed only when the aneurysm was not suitable for endosaccular embolization or the patient was intolerant to bilateral vertebral artery (VA) occlusion.

All endovascular procedures were performed in the neuroangiographic suite via the transfemoral arterial route by the author (TH: Takashi Higa). All endovascular surgeries except for one incidentally discovered aneurysm case were performed under general anesthesia. All the procedures were performed under full systemic heparinization to maintain the activated clotting time around twice the base value. In this series, Guglielmi detachable coil (GDC, Boston Scientific, Fremont, CA, USA) was mainly used to fill the aneurysm. In one case before 1997, when the GDC was approved in Japan, interlocking detachable coil (IDC, Boston Scientific) was used. The detachable coils were delivered under road-mapping guidance, and the aneurysm was packed as densely as possible.

Postoperative angiography was performed in all surgical cases within 1 month. And, initial angiographic results of clipping were classified as follows: successful clipping or partial clipping. Those of endovascular cases were classified as follows: complete occlusion (CO), neck remnant (NR), or dome filling (DF). Results of follow-up angiography were also classified in the same way, and clinical outcomes were evaluated 6 months later.

The preoperative neurological function was evaluated according to the Hunt and Kosnik (H&K) grade [21]. The neurological outcomes, including deficits prior to surgery, were divided into four categories as follows, at least 6 months following the initial surgical treatment: Good recovery (GR), patients who are essentially normal or patients with a slight disability who are nevertheless productive and essentially capable of living a normal life with only minimal limitations; moderately disabled (MD), patients with moderate neurological deficits who require family or nursing assistance but enjoy their lives at home; severely disabled (SD), patients with serious neurological deficits who totally depend on others or are bedridden; dead (D).

Results

Aneurysm size and location

The aneurysms were small in 16 (diameter <11 mm), large in five (11 to 25 mm), and giant in one (>25 mm). Of the 22 aneurysms, seven arose from the branch of the anterior inferior cerebellar artery (AICA), including one aneurysm associated with a persistent proatlantal artery, four were associated with BA fenestration, one was located at the branching of a persistent primitive hypoglossal artery, and one was located at the branching of the high-positioned posterior inferior cerebellar artery (PICA). Nine lateral saccular aneurysms incorporated with the BA trunk were demonstrated on the upper half of the BA trunk (six cases) and lower half (three cases). Seven cases were associated with multiple aneurysms. In two cases, de novo aneurysms which caused SAH were observed: a BA trunk ruptured aneurysm (case 9; Fig. 1) and a BA trunk ruptured aneurysm with a posterior cerebral artery unruptured aneurysm (case 12; Fig. 2). All cases are shown in Table 1.

Surgical treatment

Ten aneurysms were directly clipped as shown in Table 1. There were no procedure-related mortalities. However, we lost one patient due to serious brain damage at SAH onset and chronic renal failure.

Three of 11 patients treated by direct surgery required a second operation.

In one patient (case 7), the aneurysm at the proximal part of the BA fenestration was approached via a subtemporal approach. However, it was impossible to dissect the aneurysm neck between the BA fenestration and was finished with incomplete clipping. A second operation was performed via a retrosigmoid translabyrinthine approach to expose the entire aneurysm complex. However, severe adhesion around the aneurysm neck, bilateral BA fenestration, the facial nerve, and the previously applied clip did not allow fine dissection of the aneurysm neck, and this resulted in bleeding from the ipsilateral BA fenestration. A second clip was eventually applied involving one of the BA fenestrations. The patient recovered quite well, except for hearing loss.

In one patient with a large ruptured aneurysm at the AICA (case 8), neck clipping was done via an anterior transpetrosal route; however, clipping was deemed to be risky due to the presence of a stiff and broad neck with a high risk of tearing the neck at clipping. Later, the aneurysm was embolized using detachable coils. The patient was discharged without any neurological deficits.

In one patient with a de novo ruptured upper BA trunk aneurysm (case 9), a postoperative angiography taken

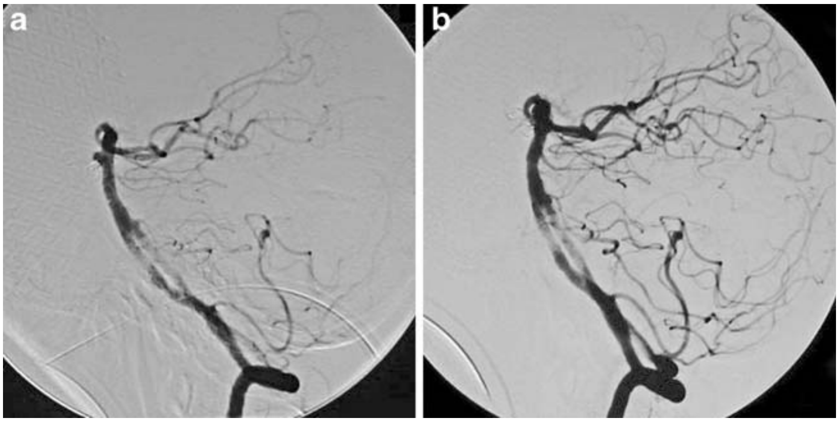

Fig. 1 Case 9. A 58-year-old woman with SAH in H&K grade IV. A postoperative angiography taken 3 weeks after surgery showing aneurysm regeneration just beside the first clip (*left*). A second surgical exposure confirming a newly developed aneurysm from the wall of the BA itself and then re-clipped with wrapping (*right*)

3 weeks after surgery showed aneurysm regeneration just beside the first clip. A second surgical exposure confirmed a newly developed aneurysm from the wall of the BA itself. The clip was replaced and reinforced with wrapping. The patient was unchanged before and after the second surgery.

Endovascular treatment

Eleven patients were initially treated with endovascular surgery using detachable coils as shown in Table 1. Coil embolization was technically feasible in all cases: five ruptured aneurysms and six unruptured aneurysms, including three aneurysms associated with coexisting ruptured aneurysms and two incidentally discovered aneurysms. In two cases, a balloon assist technique was used to prevent the coils from protruding into the BA trunk (cases 12 and 18).

There was one case which required postembolization surgical treatment. In this case (case 15; Fig. 3), the aneurysm was treated with IDC at first, but a dense packing could not be obtained. This incompletely embolized aneurysm had grown to a huge size over the subsequent 5 years, and the patient underwent a Hunterian ligation and a high flow bypass using a radial artery graft [22, 27, 29]. However, the huge thrombosed aneurysm did not shrink, and brain stem syndrome remained, leading to fatal aspiration pneumonia at a nursing home 18 months later. Another ten aneurysms were embolized without any procedure-related complications in the acute stage. However, in one case of a large BA trunk aneurysm with a wide neck (case 21), brain stem infarction occurred 3 weeks after the procedure regardless of oral administration of an antiplatelet agent.

In seven (64%) aneurysms, CO was achieved, while NR was found in three (27%) aneurysms, and DF remained in one (9%). Follow-up angiography was obtained in eight surviving cases: there was CO in six aneurysms and NR in two aneurysms. Neither re-bleeding nor recanalization of the aneurysm was observed during the follow-up period, ranging from 9 to 72 months with a mean of 28.8 months.

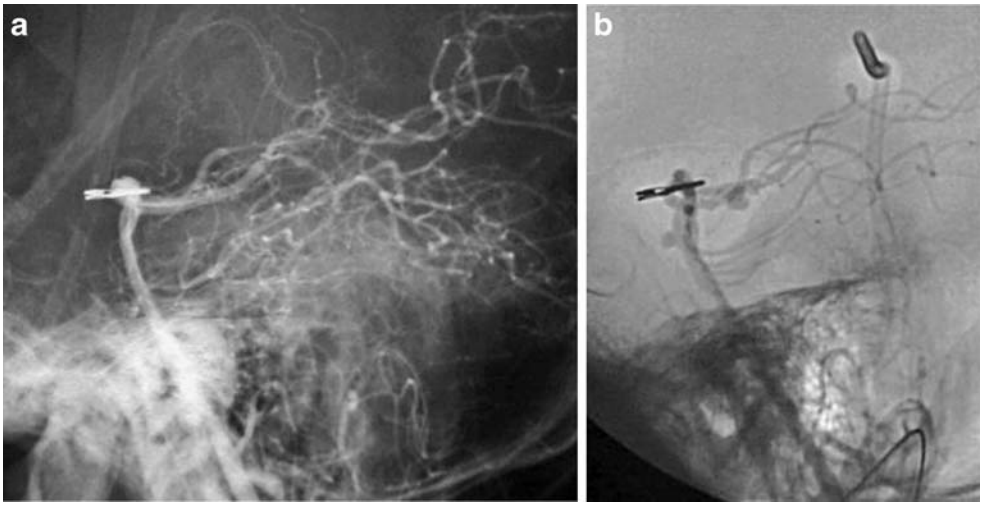

Fig. 2 Case 12. A 72-year-old woman with SAH of a poor clinical grade, with a medical history of SAH due to a ruptured BA tip aneurysm which was successfully clipped via the right pterional approach 6 years earlier (*left*). Angiography disclosed two de novo aneurysms on the upper BA trunk and the left PCA (*right*). Coil embolization using a balloon assist technique was performed

Table 1 Summary of 22 patients with the basilar artery trunk saccular aneurysms

Case no.	Age/sex	Aneurysm size (mm)/type	Presenting clinical sign	Initial treatment	Complications	Outcome	Remarks
1	20 F	18 mm/saccular Lat at the upper BA	SAH (III)	Clipping (pterional approach)	BA stenosis	MD	Drilling of PCP
2	64 F	5 mm/saccular Bra at the AICA	SAH (IV)	Clipping (pterional approach)		SD	Severe vasospasm
3	71 F	12 mm/saccular Lat at the upper BA	SAH (IV)	Clipping (STT approach)		D	Died of chronic renal failure due to renal TB, mirror aneurysms, multiple aneurysms
4	51 M	15 mm/saccular Bra at the AICA	SAH (II)	Clipping (STT approach)		GR	
5	46 F	8 mm/saccular Bra at the PPHA	SAH (II)	Clipping (STT approach)		GR	
6	73 F	6 mm/saccular Bra at the highly positioned PICA at VBJ	SAH (II)	Clipping (STT approach)		GR	
7	45 M	8 mm/saccular Bif at the BA fenestration	SAH (III)	Clipping (STT, PTP approach)	Clipping revision	GR	Second operation due to partial clip
8	37 F	15 mm/saccular Bra at the AICA	SAH (II)	Exploration (ATP approach)		GR	Coiling as a second treatment
9	58 F	5 mm/saccular Lat at the upper BA	SAH (IV)	Clipping (anterior temporal approach)	Clipping revision	SD	PH:SAH, ICH, de novo aneurysm, multiple aneurysms, second operation due to growth of aneurysm remnant
10	48 F	7 mm/saccular Bra at the AICA	SAH (II)	Clipping (PTP approach)		GR	
11	64 F	8 mm/saccular Bra at the AICA	SAH (III)	Clipping (transcondylar approach)		SD	Severe vasospasm
12	72 F	3 mm/saccular Lat at the upper BA	SAH (V)	Coiling		D	De novo aneurysm, multiple aneurysms, PH: SAH, balloon assist technique, died of initial brain damage
13	66 F	3 mm/saccular Bif at the BA fenestration	SAH (IV) due to ruptured BA-SCA aneurysm	Coiling		D	Multiple aneurysms, died of initial brain damage
14	50 F	8 mm/saccular Bif at the BA fenestration	Incidental	Coiling		GR	PH: pituitary adenoma
15	50 M	35 mm/saccular Bif at the BA fenestration	Mass effect	Coiling	Regrowth after coiling	MD	Multiple aneurysms, bilateral VA clipping and high flow bypass, died of pneumonia 18 months later
16	67 F	3 mm/saccular Lat at the upper BA	SAH (IV)	Coiling		D	Died of initial brain damage
17	52 F	4 mm/saccular Bra at the AICA	Incidental	Coiling		GR	
18	63 F	10 mm/saccular Lat at the upper BA	SAH (III)	Coiling		GR	Balloon assist technique
19	74 F	4 mm/saccular Lat at the lower BA	SAH (IV) due to ruptured VA-PICA aneurysm	Coiling		SD	Multiple aneurysms

20	45 F	5 mm/saccular Bra at the AICA	SAH (II)	Coiling		GR	Persistent proatlantal artery
21	88 M	12 mm/saccular Lat at the upper BA	SAH (II)	Coiling	Brain stem infarction	SD	
22	74 F	10 mm/saccular Lat at the lower BA	SAH (III) due to upper BA fusiform aneurysm	Coiling		SD	Multiple BA trunk aneurysms

AICA Anterior inferior cerebellar artery, *ATP* anterior transpetrosal, *BA* basilar artery, *Bif* bifurcation aneurysm, *Bra* branching aneurysm, *D* dead, *F* female, *GR* good recovery, *ICH* intracerebral hemorrhage, *Lat* lateral aneurysm, *M* male, *MD* moderately disabled, *PCA* posterior cerebral artery, *PCP* posterior clinoid process, *PH* past history, *PICA* posterior inferior cerebellar artery, *PPHA* persistent primitive hypoglossal artery, *PTP* posterior transpetrosal, *RA* radial artery, *SAH* subarachnoid hemorrhage, *SCA* superior cerebellar artery, *SD* severely disabled, *STT* subtemporal transtentorial, *TB* tuberculosis, *VA* vertebral artery, *VBJ* vertebrobasilar junction

Outcome

Of 11 cases initially treated by clipping, six showed GR, one was MD, three were SD, and one was D. On the other hand, four showed GR, one was MD, four were SD, and two were D in endovascular cases. Comparison of the outcomes in two groups of patients did not reveal statistically significant differences according to chi-square test with continuity correction (p=0.667).

Discussion

Definition of a BA trunk aneurysm

Posterior circulation aneurysms constitute 10–20% of the aneurysms seen in most surgical series [25, 39, 44]. The majority of posterior circulation aneurysms arise at the BA bifurcation, with the next most common location being the origin of the PICA from the VA. BA trunk aneurysms are very rare and can be defined as aneurysms originating from the region of the vertebrobasilar junction to the origin of the superior cerebellar artery (SCA): vertebrobasilar (VB) junction aneurysms, aneurysms arising at the site of a BA fenestration, BA-AICA aneurysms, lateral type saccular aneurysms at the BA trunk, and fusiform aneurysm involving the BA trunk [49]. BA trunk aneurysms are considered to comprise less than 1% of intracranial aneurysms [36, 41].

Characteristics of BA trunk saccular aneurysms

Saccular aneurysms generally arise at the apex of arterial bifurcations [12, 40, 43]. It is generally believed that hemodynamic stress due to main blood stream impingement at apices causes flow-induced remodeling, subsequently initiating aneurysmal lesions. However, the BA trunk does not have a definite bifurcation at which the axial stream impinges. This must be why BA trunk aneurysms are rare. The unusually high incidence of associated vascular anomalies, lateral aneurysms, and multiple aneurysms suggests that the focal fragility of a vessel wall must be based on aneurysm formation on the BA trunk. It is well established that anomalies of the circle of Willis and variations in the normal cerebrovascular anatomy are related to cerebral aneurysms [24, 28] and that they can be explained by inherent defects in the medial vessel wall [23, 27]. In the present study, one aneurysm arose from the origin of the persistent primitive hypoglossal artery, one was associated with the persistent proatlantal artery, and four were formed at the BA fenestration. The persistent primitive hypoglossal artery and BA fenestration are rarely encountered, with incidences of 0.02–0.03% [28] and 0.3–0.6% [4,

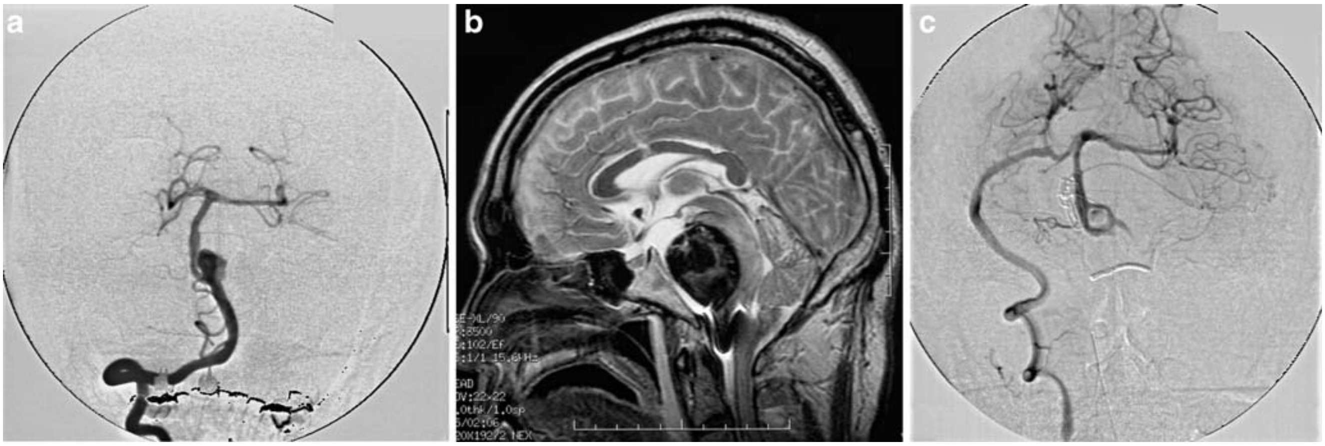

Fig. 3 Case 15. A 50-year-old man underwent coil embolization of a BA fenestration aneurysm using IDCs 5 years earlier (*left*). The incompletely embolized aneurysm grew into a thrombosed giant aneurysm, compressing the brain stem and cerebellum (*center*). A ventriculo-peritoneal shunt and Hunterian ligation under a high flow bypass between the extracranial vertebral artery and the posterior cerebral artery were performed (*right*)

5, 51], respectively. Aneurysm formation at the point of a rare vascular anomaly suggests that aneurysms result from an inherent weakness of the vessel wall.

The risk of de novo aneurysms was reported to be 1.1% to 1.8% per year [7, 31, 42]. The mean time of interval to diagnosis of de novo aneurysms is quite variable and has been reported from 7.3 to 15.5 years [33, 42]. Considering that the growth rates of individual aneurysms are not equal and that some aneurysms may be too small to detect or may exist as a weak site when the first angiograms are conducted, to confirm the time of initiation of de novo aneurysms is difficult, and they should be categorized as a special case of multiple aneurysms in which the lesions appear at different times rather than coincidentally.

In the present study, multiple aneurysms were demonstrated in 32% of cases. We think multiple occurrences of aneurysm lesions at different arterial geometries suggest that aneurysm initiation is associated with vessel wall fragility. Lateral aneurysms accounted for nine of 22 aneurysms: the upper half of the BA trunk in six aneurysms and the lower half in three. David et al. [7] reported that broad-based aneurysms, which arose from the superior BA incorporating the vessel wall, often showed regrowth after complete clipping. They suggested that the aneurysm neck was incorporated with the wall of the basilar artery itself. Similarly, we observed immediate aneurysm formation after complete neck clipping of a lateral aneurysm illustrated in Fig. 1. Presumably, microsurgical dissection around neck injures to the thin and fragile vessel wall later leads to aneurysm generation. These findings also suggest that focal wall weakness may play an important role in aneurysm formation on the BA trunk. However, the rare incidence of aneurysms on the BA trunk paradoxically means that most aneurysms may develop under hemodynamic stress.

Surgical treatment

In general, exposure of the proximal and distal vessels of the aneurysm before dissection around the neck is important in aneurysm surgery to control any unexpected premature ruptures [3]. The pterional approach as advocated by Yasargil [58] was developed to manage anterior circulation aneurysms. The removal of the anterior and posterior clinoid processes, decompression, and the possible deflection of the optic nerve enhance exposure of the upper basilar complex. However, the direction in which to clip the aneurysm neck is still limited to an anteroposterior trajectory, and this often results in a blind clip application. In this respect, an anterior temporal approach [18] or half and half approach [11] was found to provide wider exposure for large and complex lesions. Later, we considered that a modified anterior temporal approach [54], in which we dissected and separated the superficial sylvian veins from the temporal lobe, is better than an ordinary pterional approach. Slackening and posteriorly shifting the temporal lobe provides satisfactory exposure behind the internal carotid artery and allows more lateral access to the lesion. It is suggested that this approach is ideal for aneurysms of the BA apex and the superior trunk of the BA. However, this approach was used only in one case in our series.

Drake [8, 9] recommended a subtemporal transtentorial route to aneurysms located in the middle third of the BA. Sugita et al. [49] successfully clipped the aneurysm neck in eight of nine cases with BA trunk aneurysms by using this approach. When it was necessary to expose the BA trunk even further downward, we had to perform bone removal of the medial edge of the petrous bone. However, clipping the aneurysms is often difficult due to the limited viewing

angle and approach route. In such cases, an additional transcondylar or transpetrosal translabylinthine approach should be chosen to view the aneurysm neck from various angles. We used this approach in five of 11 cases.

Kawase et al. [26] devised a modification of the subtemporal transtentorial approach, which involves a subtemporal craniotomy followed by an extradural dissection along the floor of the middle fossa to the petrous ridge. A tentorial incision between the fifth cranial nerve medially and the seventh and eighth complex laterally exposes the middle third of the basilar artery. This extradural approach protects the venous drainage of the temporal lobe and is very useful for some specific cases; however, this approach often restricts the viewing direction for the clipping route, and it is sometimes impossible to clip the aneurysm neck parallel to the BA trunk when the neck is broad, as shown in the present case 8. In addition, it requires skull base surgery techniques and is thought to be difficult for non-expert neurosurgeons.

In 1979, Kasdon and Stein [23] and later Hashi et al. [17] developed a combined approach with subtemporal transtentorial and posterior transpetrosal presigmoid exposure for low-lying BA aneurysms. They believed that such an approach was advisable for lesions located between the VB junction and the AICA, which could not otherwise be exposed by the subtemporal transtentorial approach alone. Thereafter, various posterior transpetrosal approaches have been developed from the supra/infra transtentorial and lateral suboccipital approach: briefly, the transpetrosal transsigmoid, transpetrosal presigmoid retrolabylinthine, transpetrosal translabylinthine, and transcochlear approaches (total petrosectomy) [14, 35, 45, 46]. We used this approach in one case after failing to clip the aneurysm via a subtemporal approach. As a result, any approach to this area was achieved through the deep and narrow surgical corridor, and this resulted in incomplete neck clipping that later required clip replacement. Furthermore, early postoperative angiography is recommended because a residual neck or regrowth of the neck after clipping was seen in two of 11 cases. This percentage is higher than previous reports in which aneurysm remnants immediately after surgery were approximately 4% [55].

Alternative methods for treating unclippable aneurysms are wrapping or proximal vessel occlusion [10, 48]. Steinberg et al. [46] reported the surgical outcome of 201 patients undergoing an attempted Hunterian ligation without any revascularization in which a successful long-term outcome was achieved in 75% of the patients. They reported that the size of the posterior communicating artery was a good predictor of tolerance to BA occlusion. The presence of an adequate caliber of posterior communicating artery allowed retrograde filling of the BA. However, to avoid any ischemic complication, we reconstructed a high flow bypass using a radial artery graft between the extracranial VA and the posterior cerebral artery (PCA) before Hunterian ligation. Intraoperative angiography may help to confirm sufficient blood flow via the bypass.

Endovascular treatment

Several approaches have been used to access the BA trunk as described above, but these approaches have been successful only in the hands of a limited number of skilled neurosurgeons. On the other hand, endovascular access to the BA trunk is not difficult in general. In addition, the technology for endovascular surgery has progressed rapidly in the last decade [57]; therefore, we introduced these techniques in view of their minimum invasiveness in 1996. Since then, endovascular surgery has offered a striking therapeutic benefit because endovascular access is not affected by complex arteries of the BA trunk such as many perforating arteries and cranial nerves, which may often cause the problems in a surgical approach. Since then, we have selected endovascular surgery as the first choice of treatment for BA trunk aneurysms with anticipated surgical difficulties.

There was no procedure-related mortality in this series, compared with rates ranging from 0% to 3.8% in other series of posterior circulation [32, 34, 47]. A thromboembolic complication occurred in one case despite oral administration of an antiplatelet agent. As in other reported series, thromboembolic events seem to be the most common complication, and special attention must be paid to platelet aggregation after coil embolization, especially in wide-neck aneurysms.

No subsequent bleeding was observed in eight patients who survived during the follow-up period ranging from 9 to 72 months, with a mean of 28.5 months. The prevention of rebleeding by coil embolization in this series was as good as that reported in other endovascular series, ranging from 0% to 3.7% [11, 13, 38].

It is presumed that the main reason to prevent rebleeding even in neck remnant cases is to exclude bleeding point from the circulation. The goal of both direct clipping and endovascular surgery is complete exclusion of the aneurysm from the circulation. Clot formation in the sac does not mean the aneurysm is completely cured, particularly with giant aneurysms or aneurysms with a wide neck in which the main blood stream impinges upon the orifice of the aneurysm and may disturb endothelialization of the aneurysm orifice. This mechanism may induce the regrowth of aneurysms packed with coils, which occurred in the present case 15. In this case, IDC which was not suitable for endosaccular embolization was used because GDCs were not available in Japan in those days. In addition, most aneurysms in endovascular cases are small and lateral or

Table 2 Results of surgery in patients with the basilar artery trunk aneurysms

Authors (year)	Number of patients	Type of aneurysm (giant aneurysm)		Presenting clinical sign				Treatment	Outcome			
		Saccular	Fusiform	SAH	Mass	Stroke	Incidental		GR	MD	SD	D
Gianotta & Maceri (1988)	3	3	0	2	1	0	0	Clipping, 3	3	0	0	0
Rice, et al. (1990)	8	5	3	0	1	2	5	Clipping, 5; Hunterian, 1; wrapping, 1; exploration, 1	7	1	0	0
Origitano, et al. (1993)	3	3 (1)	0	3	0	0	0	Clipping, 2; trapping, 1	2	0	1	0
Day, et al. (1994)	12	12 (2)	0	11	1	0	0	Clipping, 10; Hunterian, 1; exploration, 1	8	2	0	2
Mizoi, et al. (1994)	5	4	1 (1)	5	0	0	0	Clipping, 5	5	0	0	0
Peerless, et al. (1994)	23	23 (G:5)	0	23	0	0	0	Clipping, 18; Hunterian, 5	11	6	1	5
Anson, et al. (1996)	11	0	11 (G:10)	0	11	0	0	Clip reconstruction of BA, 6; Hunterian, 3; transposition, 2 (STA-SCA bypass, 3)	6	0	2	3
Seifert & Strolke (1996)	7	7 (G:2)	0	4	2	0	1	Clipping, 6; thrombectomy, 1	6	0	0	1
Taki, et al. (1998)	3	2 (G:1)	1 (G:1)	2	0	1	0	Hunterian, 3 (RA high flow bypass, 1)	1	1	1	0
Aziz, et al. (1999)	11	11	0	10	0	0	1	Clipping, 11	7	3	1	0
Uda, et al. (2001)	26	24 (G:1)	4 (G:3)	19	4	0	3	Coiling, 23; bilateral VA occlusion, 2; unilateral VA occlusion, 1	23	0	0	3
Peluso, et al. (2007)	10	10 (G:1)	0	9	1	0	0	Coiling, 9; bilateral VA occlusion, 1	7	0	1	2
Present series (2008)	22	22 (G:1)	0	16	1	3	2	Clipping, 10; Hunterian, 1; coiling, 11	10	2	6	4
Total	144	126 (G:14)	20 (G:15)	104	22	6	12	Clipping, 76; Hunterian ligation, 14; exploration or wrapping, 3; transposition of BA, 2; endovascular trapping, 1; uni or bilateral VA occlusion, 4; coiling, 43, thrombectomy, 1	96 (66.7%)	15 (10.4%)	13 (9.0%)	20 (13.9%)

branching types which show a high tendency toward complete obliteration after coil embolization. This is shown in our previous report [19].

The results of both direct surgery and endovascular surgery in this series were recognized to be acceptable compared with the previous reports [11, 32, 34, 38, 47, 56]. There was no significant difference between these treatments, and pre-treatment neurological state may be predictive for clinical outcome. However, direct surgery required skull base techniques that may result in many postoperative complications. Furthermore, there were three of 11 cases initially treated by direct surgery by a skilled neurosurgeon which required subsequent treatment in this series. On the other hand, an endovascular approach to the BA trunk is easy compared with a surgical approach. Therefore, it is assumed that endovascular surgery for BA trunk aneurysms is an effective therapeutic alternative to clipping that is associated with low morbidity and mortality rates.

Nowadays, endovascular technology appears to be an effective and safe alternative treatment for patients with cerebral aneurysms. In the near future, even for wide-neck or fusiform aneurysms, coil embolization with stent deployment [15] or covered stent [16] may provide more stable and complete aneurysm obliteration. Endovascular therapeutic devices will undergo further modifications and refinements, which will provide a more stable and complete aneurysm occlusion.

Comparison of the previously published data and our series

A review of the literature [1, 2, 6, 14, 33, 35–37, 41, 45, 50, 52, 56] identified 144 previously reported patients with 146 BA trunk aneurysms, including 124 patients with 126 saccular aneurysms and 20 patients with 20 fusiform aneurysms. Reports of these cases included the presenting symptoms, aneurysm site and type, treatment, and outcome for individual patients, which were reviewed. The mean patient age, preoperative neurological conditions, and follow-up period were not known because some studies did not mention any details of individual records. Saccular aneurysms included 14 giant aneurysms (11%), and fusiform aneurysms included 15 giant aneurysms (75%). The presenting symptoms of the saccular aneurysms were SAH in 104 cases including coexisting aneurysms (84%), a mass effect in eight aneurysms (6%), and incidental in 12 (10%). In contrast, the fusiform aneurysms showed SAH in three aneurysms (15%), a mass effect in 14 aneurysms (70%), and stroke in three (15%). Saccular aneurysms tended to show SAH as the initial symptom; on the other hand, a fusiform aneurysm mainly presented with a mass effect.

In the treatment of all cases, including the present series, clipping of the aneurysm neck was performed in 76 of 144 cases, in which six cases were treated with a reconstruction of the BA using a clipping technique, a Hunterian ligation in 14 cases, exploration or wrapping in three cases, transposition of BA in two cases, endosaccular embolization in 43 cases, endovascular trapping in one case, unilateral or bilateral VA occlusion in four cases, and thrombectomy in one case.

The surgical outcomes of all cases were GR, 96 of 144 (66.7%); MD, 15 of 144 (10.4%); SD, 13 of 144 (9.0%); and D, 20 of 144 (13.7%). These data are shown in Table 2.

Conclusion

In our series, BA trunk aneurysms were frequently associated with other vascular anomalies, multiple aneurysms, and lateral aneurysms. Most cases with saccular aneurysms presented with SAH. Pretreatment neurological state may be predictive for clinical outcome. And, clinical outcomes in this series were not affected by the choice of treatment. However, endovascular surgery is more preferable because of minimum invasiveness. To manage of BA trunk saccular aneurysms, endovascular surgery should be considered as the first choice. Advances in new device technology will allow us to provide better clinical and anatomical outcomes for such challenging lesions.

Finally, because of the high propensity of de novo aneurysms, multiple aneurysms and various vascular anomalies, close angiographic follow-up is indispensable.

References

1. Anson JA, Lawton MT, Spetzler RF (1996) Characteristics and surgical treatment of dolichoectatic and fusiform aneurysms. J Neurosurg 84:185–193
2. Aziz KMA, van Loveren HR, Tew JM Jr, Chicoine MR (1999) The Kawase approach to retrosellar and upper clival basilar aneurysms. Neurosurgery 44:1225–1236
3. Batjer HH, Samson D (1986) Intraoperative aneurysmal rupture: incidence, outcome, and suggestions for surgical management. Neurosurgery 18:701–707
4. Byrne JV, Tasker AD (1997) Basilar artery fenestration in association with aneurysms of the posterior cerebral circulation. Neuroradiology 39:185–189
5. Campos J, Fox AJ, Vinuela F (1987) Saccular aneurysms in basilar artery fenestration. Am J Neuroradiol 8:233–236
6. Day JD, Fukushima T, Giannotta SL (1997) Crainal base approaches to posterior circulation aneurysms. J Neurosurg 87:544–554
7. David CA, Vishteh AG, Spetzler RF, Lemole M, Lawton MT, Partovi S (1999) Late Angiographic follow-up review of surgically treated aneurysms. J Neurosurg 91:396–401
8. Drake CG (1968) The surgical treatment of aneurysms of the basilar artery. J Neurosurg 29:436–446
9. Drake CG (1979) The treatment of aneurysms of the posterior circulation. Clin Neurosurg 26:96–144

10. Drake CG, Peerless SJ (1997) Giant fusiform intracranial aneurysms: review of 120 patients treated surgically from 1965 to 1992. J Neurosurg 87:141–162
11. Eskridge JM, Song JK (1998) Endovascular embolization of 150 basilar tip aneurysms with Guglielmi detachable coils: results of the Food and Drug Administration multicenter clinical trial. J Neurosurg 89:81–86
12. Ferguson GG (1972) Physical factors in the initiation, growth, and rupture of human intracranial saccular aneurysms. J Neurosurg 37:666–677
13. Feuerberg I, Lindquist C, Lindqvist M, Steiner L (1987) Natural history of postoperative aneurysm rests. J Neurosurg 66:30–34
14. Giannotta SL, Maceri DR (1988) Retrolabylinthine transsigmoid approach to basilar trunk and vertebrobasilar artery junction aneurysms. Technical note. J Neurosurg 69:461–466
15. Lanzino G, Wakhloo AK, Fessler RD, Hartney ML, Guterman LR, Hopkins LN (1999) Efficacy and current limitations of intravascular stents for intracranial internal carotid, vertebral, and basilar artery aneurysms. J Neurosurg 91:538–546
16. Pero G, Denegri F, Valvassori L, Boccardi E, Scialfa G (2006) Treatment of a middle cerebral artery giant aneurysm using a covered stent. J Neurosurg 104:965–968
17. Hashi K, Nin K, Shimotake K (1982) Transpetrosal combined supratentorial and infratentorial approach for midline vertebrobasilar aneurysms. In: Brock M (ed) Modern Neurosurgery, vol 1. Springer, Berlin, pp 442–448
18. Heros RC, Lee SH (1993) The combined pterional/anterior temporal approach for aneurysms of the upper basilar complex: technical report. Neurosurgery 33:244–251
19. Higa T, Ujiie H, Hori T (2007) Factors affecting successful embolization of intracranial aneurysms with Guglielmi detachable coils. J Tokyo Wom Med Univ 77:28–37
20. Hori T, Tanabe M, Okamoto H, Numata H, Hokama Y, Watanabe T, Ishii T, Teraoka A (1994) Basilar artery trunk aneurysms—eight operative experiences and 2 autopsy findings. Surg Cereb Stroke 22:495–504
21. Hunt WE, Kosnik EJ (1974) Timing and perioperative care in intracranial aneurysm surgery. Clin Neurosurg 21:79–89
22. Kamiyama H (1994) Bypass with radial artery graft. No Shinkei Geka 22:911–924
23. Kasdon DL, Stein BM (1979) Combined supratentorial and infratentorial exposure for low-lying aneurysms. Neurosurgery 4:422–426
24. Kayembe KNT, Sasahara M, Hazama F (1984) Cerebral aneurysms and variations in circle of Willis. Stroke 14:846–850
25. Kassel NF, Torner JC, Haley Jr EC, Jane JA, Adams HP, Kongable GL (1990) The international cooperative study on the timing of aneurysm surgery. Part 1: overall management results. J Neurosurg 73:18–36
26. Kawase T, Toya S, Shiobara R, Mine T (1985) Transpetrosal approach to aneurysms of the lower basilar artery. J Neurosurg 63:857–861
27. Kobayashi M, Kamiyama H (1999) Reconstruction of the basilar arterial system: methods of high flow bypass in posterior cranial fossa. Surg Cereb Stroke 27:270–276
28. Lie TA (1968) Congenital malformation of the carotid arteries inclusing the carotid–basilar anastomosis and carotid–vertebral anastomosis: Angiographic study and review of literature. Exp Med 35:44–49
29. Lawton MT, Hamilton MG, Morcos JJ, Spetzler RF (1996) Revascularization and aneurysm surgery: current techniques, indications, and outcome. Neurosurgery 38:83–94
30. Lawton MT, Daspit PD, Spetzler RF (1997) Technical aspects and recent trends in the management of large and giant midbasilar artery aneurysms. Neurosurgery 41:513–521
31. Miller CA, Hill SA, Hunt WE (1985) “De Novo” aneurysms A clinical review. Surg Neurol 24:173–180
32. Lempert TE, Malek AM, Halbach VV, Phatouros CC, Meyers PM, Dowd CF, Higashida RT (2000) Endovascular treatment of ruptured posterior circulation cerebral aneurysms: clinical and angiographic outcomes. Stroke 31:100–110
33. Mizoi K, Yoshimoto T, Takahashi A, Ogawa A (1994) Direct clipping of basilar trunk aneurysms using temporary balloon occlusion. J Neurosurg 80:230–236
34. Nichols DA, Brown RD Jr, Thielen KR, Meyers FB, Atkinson JLD, Piepgras DG (1997) Endovascular treatment of ruptured posterior circulation aneurysms using electrolytically detachable coils. J Neurosurg 87:374–380
35. Origitano TC, Anderson DE, Tarassori Y (1993) Skull base approaches to complex cerebral aneurysms. Surg Neurol 40:339–346
36. Peerless SJ, Hernesniemi JA, Gutman FR, Drake CG (1994) Early surgery for ruptured vertebrobasilar aneurysms. J Neurosurg 80:643–649
37. Peluso JPP, van Rooij WJ, Sluzewski M, Beute GN (2007) Aneurysms of the vertebrobasilar junction: Incidence, clinical presentation, and outcome of endovascular treatment. Am J Neuroradiol 28:1747–1751
38. Pierot L, Boulin A, Castaings L, Ray A, Moret J (1996) Selective occlusion of basilar artery aneurysms using controlled detachable coils: report of 35 cases. Neurosurgery 38:948–953
39. Redekop GJ, Durity FA, Woodhurst WB (1997) Management-related morbidity in unselected aneurysms of the upper basilar artery. J Neurosurg 87:836–842
40. Rhoton AL Jr (1980) Special article. Anatomy of saccular aneurysms. Surg Neurol 14:59–66
41. Rice BJ, Peerless SJ, Drake CG (1990) Surgical treatment of unruptured aneurysms of the posterior circulation. J Neurosurg 73:165–173
42. Rinne JK, Hernesniemi JA (1993) De novo aneurysms: special multiple intracranial aneurysms. Neurosugery 33:981–985
43. Roach MR, Scott S, Ferguson GG (1972) The hemodynamic importance of the geometry of bifurcations in the circle of Willis (glass model studies). Stroke 3:255–267
44. Samson D, Batjer HH, Kopitnik TA Jr (1999) Current results of the surgical management of aneurysms of the basilar apex. Neurosurgery 44:697–704
45. Seifert V, Stolke D (1996) Posterior transpetrosal approach to aneurysms of the basilar trunk and vertebrobasilar junction. J Neurosurg 85:373–379
46. Spetzler RF, Daspit CP, Pappas CTE (1992) The combined supra- and infratentorial approach for lesions of the petrous and clival region: experience with 46 cases. J Neurosurg 76:588–599
47. Steiger HJ, Medele R, Bruckmann H, Schroth G, Reulen HJ (1999) Interdisciplinary management results in 100 patients with ruptured and unruptured posterior circulation aneurysms. Acta Neurochir 141:359–366
48. Steinberg GK, Drake CG, Peerless SJ (1993) Deliberate basilar or vertebral artery occlusion in the treatment of intracranial aneurysms Immediate results and long-term outcome in 201 patients. J Neurosurg 79:161–173
49. Sugita K, Kobayashi S, Takemae T, Tada T, Tanaka Y (1987) Aneurysms of the basilar trunk. J Neurosurg 66:500–505
50. Sundt TM Jr, Piepgras DG, Marsh W (1986) Saphenous vein bypass grafts for giant aneurysms and intracranial occlusive disease. J Neurosurg 65:439–450
51. Takahashi M, Tamakawa Y, Kishikawa T, Kowada M (1973) Fenstration of the basilar artery. Radiology 107:79–82
52. Taki W, Nakahara I, Sakai N, Irie K, Murao K, Ohkata N, Tanaka M, Kikuchi H (1998) Large and giant middle to lower basilar

trunk aneurysms treated by surgically and interventional neuroradiological methods. Neurol Med Chir 38:826–835
53. Tanabe M, Yamasaki T, Hori T (1994) Subtemporal-transtentorial approach for high position vertebral artery—posterior inferior cerebellar artery aneurysm: A case report. Surg Cereb Stroke 22:5–8
54. Tanikawa R, Wada H, Ishizaki T, Izumi N, Fujita T, Hashimoto M, Kamiyama H (1998) Anterior temporal approach for basilar bifurcation aneurysms as a modified distal transsylvian approach. Surg Cereb Stroke 26:259–264
55. Thornton J, Bashir Q, Aletich VA, Debrun GM, Ausman JI, Charbel FT (2000) What percent of surgically clipped intracranial aneurysms have residual neck? Neurosurgery 46:1294–1300
56. Uda K, Murayama Y, Gobin YP, Duckwiler GR, Vinuela F (2001) Endovascular treatment of basilar artery trunk aneurysms with Guglielmi detachable coils: clinical experience with 41 aneurysms in 39 patients. J Neurosurg 95:624–632
57. Vinuela F, Duckwiler G, Mawad M (1997) Guglielmi detachable coil embolization of acute intracranial aneurysm: perioperative anatomical and clinical outcome in 403 patients. J Neurosurg 86:475–48
58. Yasargil MG, Antic J, Laciga R, Jain KK, Hodosh RM, Smith RD (1976) Microsurgical pterional approach to aneurysms of the basilar bifurcation. Surg Neurol 6:83–91

Comments

Kazuhiko Nozaki, Otsu, Japan

Aneurysms of basilar artery trunk are still difficult to obtain complete cure by open surgery, and recent advancements in endovascular surgery bring favorable outcomes in some selected patients. The authors summarized a relatively large series of basilar artery (BA) trunk saccular aneurysms from 22 patients (more than 11 mm in diameter in six cases) who underwent open surgery and/or endovascular surgery at their institute with relatively good clinical outcomes. Their treatment strategy has changed since 1996, after which endovascular surgery is the first choice. Adequate skull base techniques and preservation of perforators are mandatory for complete direct clipping of these lesions, and flow alteration techniques should be the last choice considering surgical morbidity and curability. Lateral type of aneurysms seems to be suitable for endovascular surgery in terms of curability, and further development in materials such as coils and stents may increase amenable cases for endovascular surgery. Although the authors discussed about the possible relationship between the incidences of vascular anomaly and aneurysms, the vascular anomaly itself does not necessarily mean the weakness and fragility of vascular walls. They also discussed about multiplicity and vascular wall fragility, but it is difficult to conclude that basilar trunk saccular aneurysms occur based on vascular fragility because of small sample size. Basilar artery trunk aneurysms are still challenging diseases, and surgical indication should be cautiously applied depending on location and morphology of aneurysms and patient's conditions.

Peter Vajkoczy, Berlin, Germany

The treatment of basilar artery trunk aneurysms still remains one of the major challenges in modern neurosurgery and interventionell neuroradiology. In the present study, Prof. Hori and his colleagues report on their experiences in treating 22 patients presenting with basilar artery trunk saccular aneurysms, most of them following rupture and SAH. Half of the patients have been treated by surgery applying skull approaches and advanced clipping strategies. The others have been treated by endovascular surgery. The authors have to be congratulated for their excellent clinical and morphological results. This applies both to surgical and endovascular treatment. Their study clearly demonstrates what modern subspecialized and interdisciplinary neurosurgery can provide excellent treatment results to patients presenting with these challenging lesions. The authors clearly state that today endovascular surgery should be the treatment modality no. 1 for saccular aneurysms along the basilar trunk, especially since the surgical approach by itself is often associated with significant comorbidities, and clipping of the aneurysm is most often limited by the narrow space. Therefore, complete obliteration of the aneurysm can often not be provided by surgery. In contrast, the endovascular approach is clearly the less invasive strategy, and particularly in this region along the basilar artery trunk, the rate of incomplete aneurysm obliteration is comparable to microsurgery. On the other hand, this study again demonstrates that, in case of basilar artery trunk aneurysms that are not well suited for endovascular therapy, microsurgery still remains an excellent therapeutic option.

Carl Muroi, Zurich, Switzerland

This study is about a single center experience in treating a rare type of aneurysm. The authors present good result in treating basilar artery trunk aneurysm by either surgical clipping and/or endovascular coiling. The authors report that the outcome was not affected by the choice of treatment, though it must be assumed that the clipping group is similar to a "historical control group" since after introduction of endovascular surgery the majority was rather coiled than clipped. Therefore, the follow-up period of the "clipping group" must be assumed to be longer than the "coiling group," which ranged from 9 to 72 months with a mean of 28.8 months. The incidence of recanalization and/or rebleeding after coiling might rise when the follow-up period becomes equal to the "clipping group." This circumstance makes the comparison of these groups and its interpretation a little bit difficult in my opinion.
