# Supplementary material for: Management strategies and outcomes of basilar trunk aneurysms: a systematic review and meta-analysis
Source: Langenbecks Arch Surg. 2026 Jan 20;411(1):71. doi: 10.1007/s00423-025-03959-3 (PMC12852281; doi:10.1007/s00423-025-03959-3)
Supplement: Supplementary file 1 — Supplementary file1 (ZIP 55327 KB) [file 423_2025_3959_MOESM1_ESM.zip › literature.zip!/meta/kalani2013.pdf]

Bypass and Flow Reduction for Complex Basilar and Vertebrobasilar Junction Aneurysms

M. Yashar S. Kalani, MD, PhD
Joseph M. Zabramski, MD
Peter Nakaji, MD
Robert F. Spetzler, MD

Division of Neurological Surgery, Barrow Neurological Institute, St. Joseph's Hospital and Medical Center, Phoenix, Arizona

Correspondence:
Robert F. Spetzler, MD,
c/o Neuroscience Publications,
Barrow Neurological Institute,
St. Joseph's Hospital and
Medical Center,
350 W Thomas Rd,
Phoenix, AZ 85013.
E-mail: Neuropub@dignityhealth.org

Received, September 10, 2012.
Accepted, December 25, 2012.
Published Online, January 17, 2013.

Copyright © 2013 by the
Congress of Neurological Surgeons

BACKGROUND: Giant aneurysms of the vertebral and basilar arteries are formidable lesions to treat.
OBJECTIVE: To evaluate the long-term outcomes of patients with vertebrobasilar aneurysms treated with extracranial-intracranial bypass and flow reduction.
METHODS: We retrospectively reviewed a prospective database of aneurysms cases treated between December 1993 and August 2011.
RESULTS: Eleven patients (8 male, 3 female) with 12 aneurysms were treated. There were 3 basilar apex aneurysms, 2 aneurysms of the basilar trunk, and 7 vertebrobasilar junction aneurysms. There were 5 saccular and 7 fusiform aneurysms. All patients underwent extracranial-intracranial bypass and vessel occlusion. Flow was reversed or reduced by complete (n = 6) or partial occlusion of the basilar artery (n = 3) or by occlusion of the vertebral arteries distal to the posterior inferior cerebellar artery (n = 3). Postoperatively (mean follow-up, 71.6 months; range, 4-228; median, 49 months), the bypass patency rate was 92.3% (12/13). The perioperative mortality rate for the initial treatment was 18.2% (2/11). In 4 cases, the aneurysms continued to grow and required further treatment; after re-treatment, 3 of these patients died. Of the initial 11 patients, 6 were treated successfully and 5 died. The mean preoperative modified Rankin Scale score was 2.1 (range, 1-3; median, 2). At last follow-up for all patients, the mean modified Rankin Scale score was 3.45 (range, 1-6; median, 3) and 2.5 (range, 1-4; median, 2.5) for the 6 long-term survivors.
CONCLUSION: Vertebrobasilar aneurysms are challenging lesions with limited microsurgical or endovascular options. Despite aggressive surgical treatment, the long-term outcome remains poor for most patients.

KEY WORDS: Aneurysm, Basilar artery, Complex, Extracranial-intracranial, Flow reduction, Giant, Vertebral artery

Neurosurgery 72:763–776, 2013 DOI: 10.1227/NEU.0b013e3182870703 www.neurosurgery-online.com

Giant, fusiform, and complex aneurysms of the basilar and vertebrobasilar junction pose major challenges for both microsurgical and endovascular treatment. These aneurysms tend to be partially calcified and thrombosed, to have perforators emanating from the aneurysm, to manifest with mass effect or embolic episodes, and to reside within a labyrinth of critical neurovascular structures. These features make direct surgical clipping or endovascular coiling challenging. Flow-diverting stents have been used successfully to treat some vertebral and basilar aneurysms.[1] However, outcomes of the challenging subset of fusiform aneurysms have been disappointing,[2] and complications associated with other aneurysms in the posterior circulation are also being reported.[3,4]

ABBREVIATIONS: EC-IC, extracranial-intracranial; **mRS,** modified Rankin Scale; **PCA,** posterior cerebellar artery; **PICA,** posterior inferior cerebellar artery; **SCA,** superior cerebellar artery; **STA,** superficial temporal artery

Historically, the treatment of choice for these aneurysms has been Hunterian ligation of the basilar artery or of one or both vertebral arteries.[5,6] Occlusion of the inflow arteries to the aneurysm redirects and decreases flow within the aneurysm. The intent is to induce gradual thrombosis of the aneurysm or at least to prevent further growth. Hunterian ligation is not without risks, and brainstem strokes caused by a mismatch in blood flow have been reported.[6] One method of augmenting flow to the basilar apex and brainstem is by performing an extracranial-intracranial (EC-IC)

Copyright © Congress of Neurological Surgeons. Unauthorized reproduction of this article is prohibited.

TABLE. Patient Characteristics

Case	Sex/ Age, y	Location	Aneurysm Type	Presentation and Previous Treatment	Preop mRS	BTO	Treatment	Bypass Type
1	M/43	VB	Giant fusiform	Brainstem TIA	3	Failed	Partial surgical occlusion of BA	STA-PCA
	48	VB	Giant fusiform	Brainstem compression	3		Coil/Onyx occlusion of BA	STA-SCA
2	M/54	VB	Giant fusiform	Brainstem compression, diplopia, dysarthria, weakness, gait instability, difficulty swallowing	2		Complete surgical occlusion of BA	STA-SCA
3	M/47	VB	Giant fusiform	Hemianesthesia, weakness, difficulty swallowing; CN 4 and 6 palsy	2	Failed	Surgical occlusion of RVA distal to PICA	STA-SCA
4	M/55	VB	Giant fusiform	Slurred speech, blurred vision, leg weakness	3		–	STA-SCA
5	F/51	Basilar	Giant fusiform	Memory difficulty, weakness, headaches, speech difficulty	3	Failed	Coil occlusion of BA	STA-SCA
6	M/58	VB	Giant fusiform	Hemiparesis, ataxia, dysarthria, diplopia; previous R VA coil occlusion	3		Surgical occlusion of BA with decompression of aneurysm content	STA-SCA
7	M/68	VB	Giant saccular	Headaches, dizziness, nausea and vomiting	1		Coil occlusion of VA and aneurysm	STA-SCA
8	F/32	Basilar trunk	Large saccular	Depression, decline in mentation	1	Passed	Surgical and coil occlusion of VA	STA-SCA
9	F/55	Basilar tip	Large saccular	Headaches and neck stiffness without SAH	1		Partial surgical occlusion of BA	STA-SCA
	55	Basilar tip	Large saccular	Progressive growth on angiogram	1		Complete surgical occlusion of BA	STA-SCA/STA-PCA
10	M/52	Basilar tip and trunk	Medium/large saccular	Previous SAH from midbasilar aneurysm treated with Neuroform stent; expressive aphasia, upper extremity weakness	3		Partial surgical occlusion of BA	STA-SCA
11	M/45	Basilar	Giant fusiform	Gait difficulty, dysarthria, left-sided weakness	1		Endovascular occlusion of BA and decompression of aneurysm content	STA-SCA

Case	Bypass Patency Postop	Angiographic Bypass F/U, month	Complications	LOS, d	F/U (months)	Postop mRS	Long-Term mRS	Other Aneurysms	Aneurysm at Last F/U
1	Yes	Patent at 26 mo; occluded at 60 mo postop	HCP	17	62	3			Increased
	Yes	-	CVA	33	–	6	6		Rehemorrhage while in hospital; died
2	Yes	–	HCP, EDH, CN 3,6,7 palsy, coronary artery bypass, MI	74	10	2	2		No imaging F/U
3	Yes	–	HCP	3	73	2	2		No imaging F/U
4	Yes	-	CVA, HCP, IPH	19	4	4	6		1 mo postop returned with brainstem infarct; ruptured basilar artery 4 mo postop; died
5	Yes	141		12	141	3	3	PCOM, ICA	Recurrence and endovascular re-treatment at 1, 2, 5, 6 y F/U; 12-y F/U no filling
6	Yes	–	EDH	–	12	4	4		No imaging F/U

(Continues)

Copyright © Congress of Neurological Surgeons. Unauthorized reproduction of this article is prohibited.

TABLE 1. *Continued*

Case	Bypass Patency Postop	Angiographic Bypass F/U, month	Complications	LOS, d	F/U (months)	Postop mRS	Long-Term mRS	Other Aneurysms	Aneurysm at Last F/U
7	Yes	–	CVA, HCP, SDH/EDH, postop cardiac and pulmonary arrests	15	–	6	6		Surgical death
8	Yes	132		15	132	1	6		Increased; died of SAH 13 y later during attempted coiling
9	No	–	Bypass thrombosis	6	18	1		ACOMM	increased
	Yes	Postop	Bypass thrombosis, CVA	25	–	6	6		Died 14 days after bypass of brainstem infarction.
10	Yes	Patent at 26 mo; slow flow at 36 mo postop		17	36	3	3		Stable flow within aneurysm
11	Yes	-	HCP	12	228	1	1		Minimal flow

HCP, hydrocephalus; VB, vertebrobasilar; CVA, cerebrovascular accident; EDH, extradural hemorrhage; MI, myocardial infarction; IPH, intraparenchymal hemorrhage; TIA, transient ischemic attack; PCOM, posterior communicating artery; ICA, internal carotid artery; BA, basilar artery; SDH, subdural hemorrhage; BTO, balloon test occlusion; F/U, follow up; R, right; VA, vertebral artery; Preop, preoperative; Postop, postoperative; mRS, modified Rankin Scale; SCA, superior cerebellar artery; STA, superficial temporal artery; PCA, posterior cerebellar artery; SAH, subarachnoid hemorrhage; ACOMM, anterior communicating artery.

arterial bypass before planned vessel occlusion. Although positive outcomes have been reported with this technique, scant data on the long-term outcome of such cases are available. We therefore reviewed the outcomes of our surgical treatment for this small but challenging group of aneurysms. Our treatment strategy for these patients consisted of EC-IC bypass combined with occlusion of the inflow vessel(s) to the aneurysm.

CLINICAL MATERIAL AND METHODS

Patient Population

Between 1993 and 2011, 11 patients (8 males, 3 females) with 12 symptomatic, complex or giant aneurysms of the basilar artery or vertebrobasilar junction were treated at Barrow Neurological Institute, St. Joseph's Hospital and Medical Center, Phoenix, Arizona (Table). Two of these patients have previously been reported.[7] The patients ranged in age from 32 to 68 years (mean, 50.7 years). All patients underwent surgery or combined surgery and endovascular treatment for their aneurysms. Pre- and postoperative neurologic function was evaluated by using the modified Rankin Scale (mRS).

Aneurysm Location and Characteristics

Giant aneurysms were defined as those with diameters greater than 2.5 cm as measured on computed tomographic (CT) or magnetic resonance imaging (MRI). Fusiform, serpentine, and saccular aneurysms were defined based on their angiographic morphology. Of the 12 aneurysms, 8 were giant and 4 were large. Three patients had multiple aneurysms. One patient had a large, symptomatic basilar trunk aneurysm and a medium basilar apex aneurysm. One patient had an anterior communicating aneurysm and a 1.2-cm basilar apex aneurysm. The third patient had an internal carotid artery terminus, a posterior communicating artery, and a 2.5-cm basilar apex aneurysm. There were 5 saccular and 7 fusiform aneurysms.

Clinical Presentation

One patient had a remote history of subarachnoid hemorrhage. One patient presented with symptoms of meningismus and headache, but a complete evaluation, including lumbar puncture, failed to provide evidence of hemorrhage. The remaining 9 patients had symptoms suggestive of mass effect (n = 5) or embolic episodes (n = 4). The most frequent symptoms included hemiparesis, ataxia, dysarthria, and cranial nerve deficits. In 2 cases, re-treatment was indicated. In 1 of these patients, routine follow-up imaging showed evidence of aneurysmal regrowth. The other patient had symptoms of brainstem compression. Initial and follow-up vascular imaging was performed by using conventional angiography or 3-dimensional CT angiography.

Treatment

All 11 patients underwent preoperative angiography. Balloon test occlusion data were available for 4 patients: 1 patient passed and 3 failed the test. The decision to perform a bypass was based on the degree of collateral circulation as judged on the basis of the results of balloon test occlusion, caliber of the posterior communicating arteries, and presence of a complete circle of Willis. Preoperatively, all patients were placed on aspirin (325 mg/day) before surgical intervention, and the dosage was continued postoperatively. Some patients had a short postoperative course of intravenous heparin to prevent sudden thrombosis of the aneurysm. Electroencephalographic monitoring was conducted intraoperatively.

Copyright © Congress of Neurological Surgeons. Unauthorized reproduction of this article is prohibited.

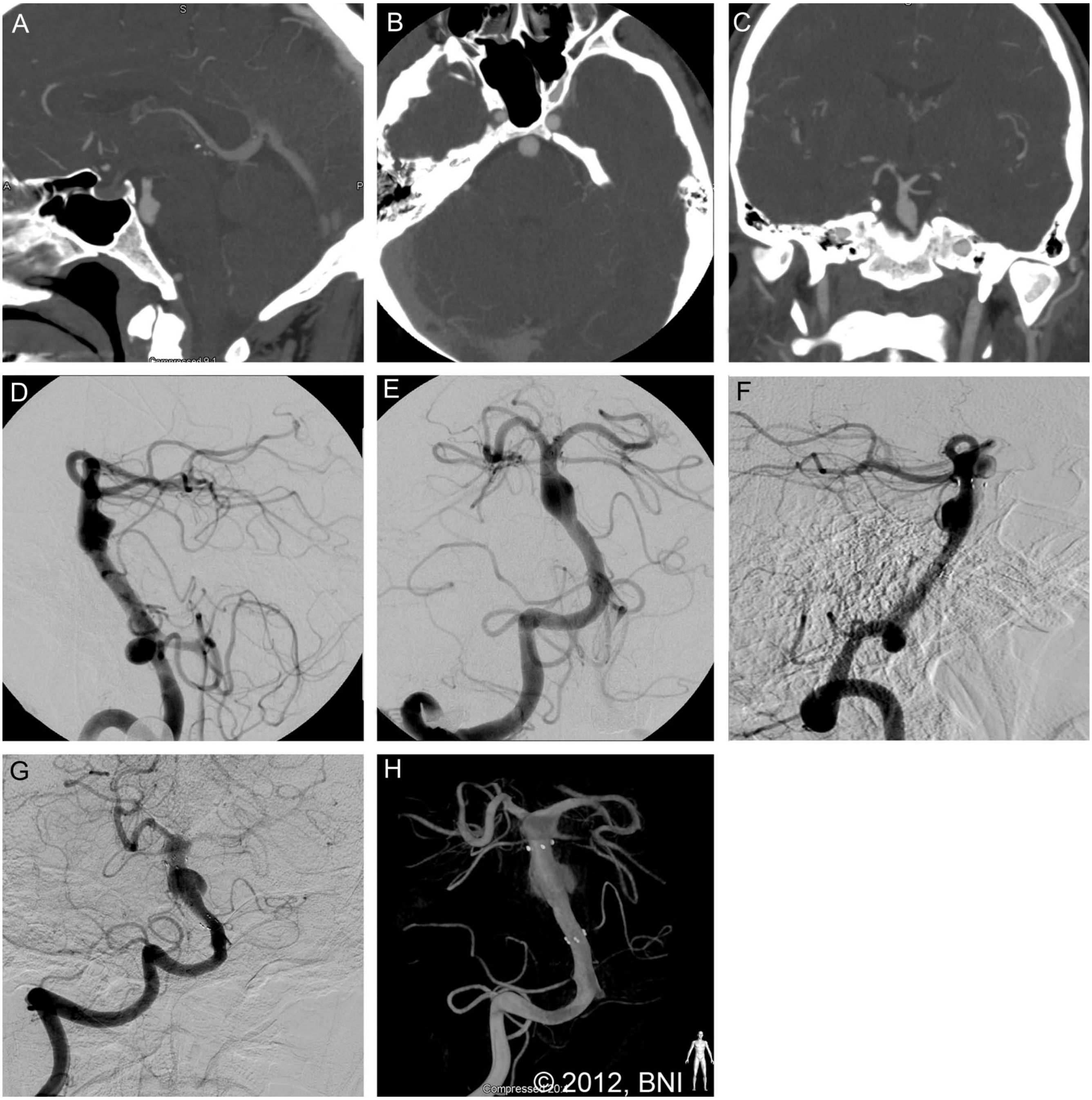

FIGURE 1. *Sagittal (**A**), axial (**B**), and coronal (**C**) CT angiograms demonstrated a basilar trunk aneurysm. The patient initially presented with subarachnoid hemorrhage. The basilar trunk aneurysm was stented. Post-stenting lateral (**D**) and anteroposterior (**E**) angiograms reveal the morphology of the aneurysm. One-year follow-up lateral (**F**) and anteroposterior (**G**) angiograms demonstrate growth of the basilar trunk aneurysm and a new basilar apex aneurysm. Three-dimensional reconstruction reveals the anatomy of the aneurysms (**H**).* Used with permission from Barrow Neurological Institute.

Copyright © Congress of Neurological Surgeons. Unauthorized reproduction of this article is prohibited.

EC-IC bypass procedures were performed after mild hypothermia was established, and barbiturate protection was provided as described previously.[8] Intraoperative evaluation of the bypass and treatment were confirmed with indocyanine green videoangiography or intraoperative angiography. Postoperative angiography was obtained to document baseline patency of the bypass and the extent of residual aneurysmal filling.

All patients underwent a full orbitozygomatic approach for the EC-IC bypass. Bypasses performed included superficial temporal artery-superior cerebellar artery (STA-SCA, n = 11), STA-posterior cerebellar artery (PCA, n = 1), and a novel double-barrel STA-SCA/STA-PCA bypass (n = 1). The STA was used as a donor artery for all bypasses.

After a successful EC-IC bypass was documented, the basilar or vertebral arteries were occluded proximally by clipping (n = 7) or endovascular technique (n = 4). A combined surgical-endovascular approach was used in 1 case. When the vertebral artery was occluded surgically, a far-lateral approach or variations thereof were used. Six patients underwent complete occlusion of the basilar artery, and 3 had partial occlusion of the basilar artery. Three patients underwent occlusion of both vertebral arteries distal to the posterior inferior cerebellar artery (PICA). In one case (case 4), vessel occlusion was planned in a staged fashion but delayed when the patient had a postoperative brainstem infarct. A month later, this patient returned to the hospital with a devastating hemorrhage from aneurysmal rupture and died. In 2 cases, the aneurysm was partially thrombectomized to reduce mass effect before the vessel was occluded. In 3 cases with basilar apex aneurysms, the basilar artery was only partially occluded to decrease blood flow.

RESULTS

The mean length of patients' initial hospitalization was 20.7 days (range, 3-74; median, 16 days). The mean length of long-term follow-up was 71.6 months (range, 4-228; median, 49 months). Long-term angiographic follow-up (>24 months) of the bypass was only available for 4 cases (Table).

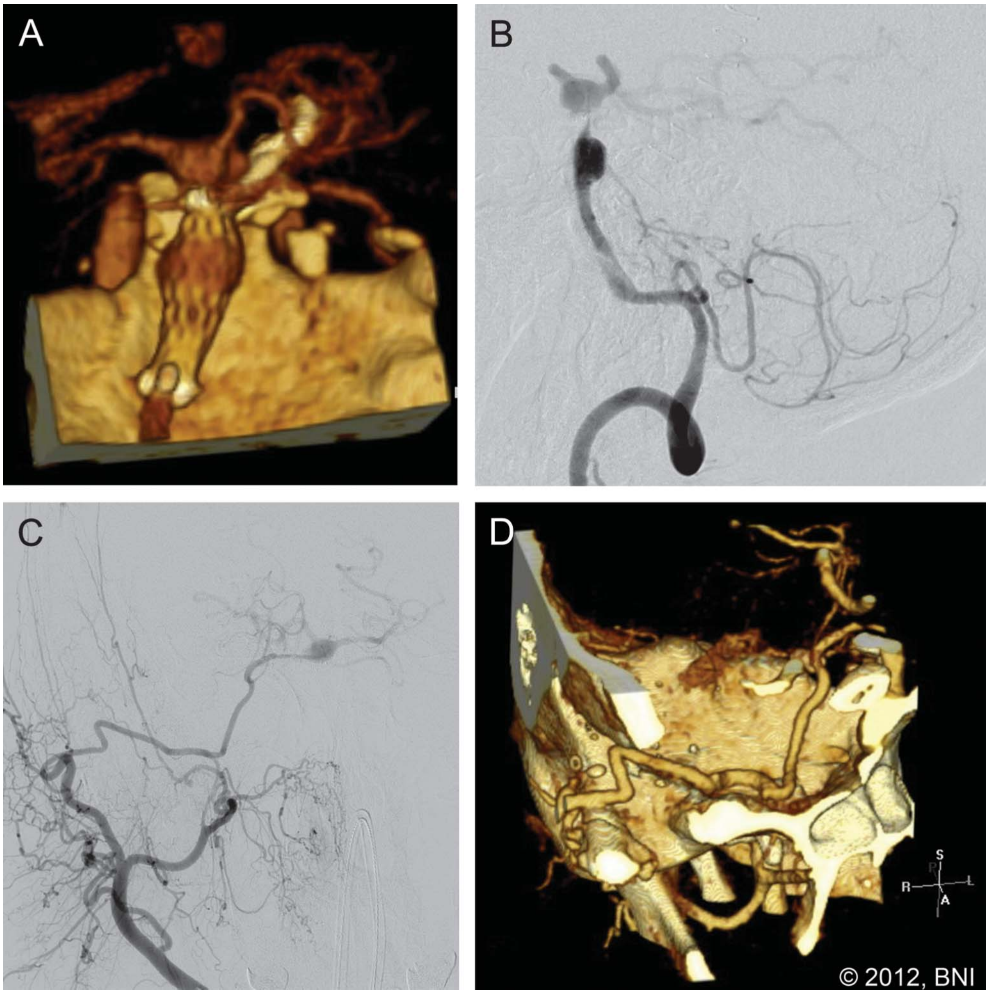

FIGURE 2. *Three-dimensional spin (**A**) and lateral angiograms (**B**) reveal partial occlusion of the basilar artery below the SCAs and above the stent. Anteroposterior (**C**) angiogram and CT angiogram (**D**) reveal a patent STA-SCA bypass. SCA, superior cerebellar artery; STA, superficial temporal artery.* Used with permission from Barrow Neurological Institute.

Copyright © Congress of Neurological Surgeons. Unauthorized reproduction of this article is prohibited.

Initial Surgical Outcomes

Postoperatively, 12 of 13 bypasses were patent (92.3%). There was a single case of asymptomatic immediate bypass thrombosis. The same patient (patient 9) had delayed postoperative occlusion of a second bypass during a repeat operation for the same aneurysm; she died of the resulting brainstem stroke.

One patient (case 7) died within 30 days of his initial treatment. A second patient (case 4) had an initial uneventful postoperative course but returned a month later with a brainstem infarct. Four months after his initial treatment, his aneurysm ruptured and he died. There were no cases of intraoperative aneurysmal rupture. Postoperatively, however, 4 cases of hemorrhage developed: 1 was intraparenchymal, 2 were epidural, and 1 was epidural and subdural.

Other complications associated with initial treatment included 6 cases of hydrocephalus and 2 infarcts (as stated, leading to death in case 7). Postoperatively, 2 patients had medical complications. One patient had a myocardial infarction and required cardiac bypass surgery, and 1 patient experienced a cardiopulmonary arrest and was not successfully resuscitated.

The mean mRS score was 2.1 (range, 1-3; median, 2) before surgery and 3.45 (range, 1-6; median, 3) after treatment. In 4 cases (cases 1, 5, 9, and 10), the aneurysms exhibited continued growth on follow-up imaging and required re-treatment.

Re-treatment Outcomes

Four patients required re-treatment, 3 of whom died. One patient (case 1) had a fair outcome after his first operation but returned with evidence of aneurysmal growth and brainstem compression. Re-treatment resulted in postoperative hemorrhage and death. After an uneventful first operation, 1 patient's (case 9) aneurysm continued to grow on follow-up angiography. After re-treatment, thrombosis of her bypass graft and a subsequent brainstem infarct led to her death. One patient (case 8) died 13 years after her initial treatment. On follow-up imaging, the patient's aneurysm had enlarged. She underwent endovascular coiling but died of intraoperative complications. The surviving patient (case 5) underwent multiple endovascular re-treatments without complications; after 12 years, follow-up angiography confirmed cure.

Excluding cases of hydrocephalus and asymptomatic bypass occlusion, surgical complications occurred in 6 of 13 operations (46.2%). Four complications were associated with initial treatment and 2 with re-treatment.

Long-term Outcome

The overall long-term outcome was heavily influenced by the high mortality rate (5 of 11 cases); the mRS score at last follow-up for all patients in this study was 3.45 (range, 1-6; median, 3). For the 6 long-term survivors, the mean mRS was 2.5 (range, 1-4; median, 2.5). Good long-term outcomes (mRS 0-2) were seen in 3 of 11 patients (27.3%), and moderate outcomes (mRS 3-4) were seen in an additional 3 patients. The remaining 5 patients died owing to disease progression or complications of treatment. Five patients were neurologically worse (by 1 mRS score or more) after treatment. The mean length of follow-up was 71.6 months (range, 4-228; median, 49 months).

ILLUSTRATIVE CASES

Case 10

A 52-year-old man with a history of a previous subarachnoid hemorrhage developed multiple episodes of expressive aphasia

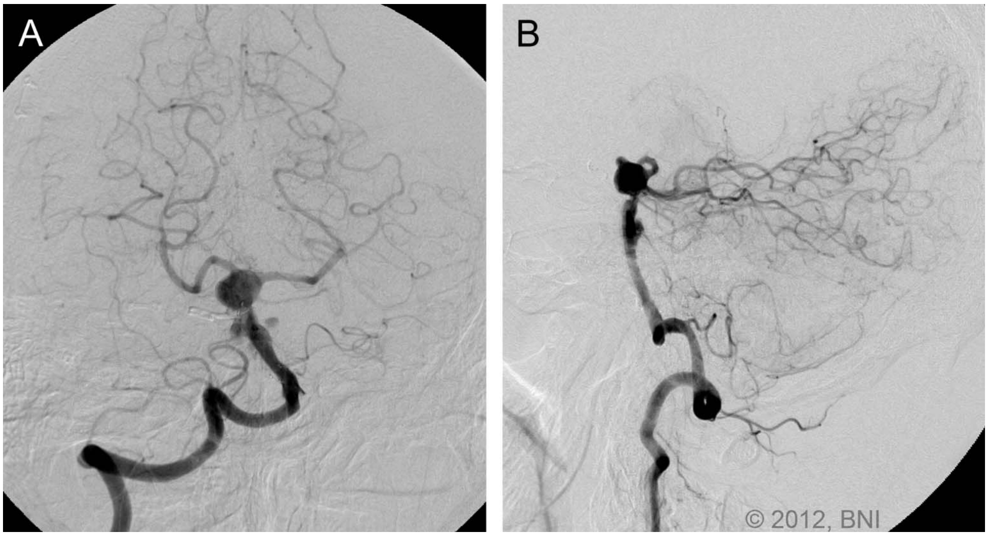

FIGURE 3. *At the patient's 3-year follow-up visit, anteroposterior (A) and lateral (B) angiograms show that the aneurysms appear relatively stable. At this point, the patient and treating team elected to follow the patient with serial scans rather than to pursue further treatment.* Used with permission from Barrow Neurological Institute.

Copyright © Congress of Neurological Surgeons. Unauthorized reproduction of this article is prohibited.

and upper extremity weakness. At the time of his initial hemorrhage, he was noted to have a midbasilar aneurysm (Figure 1A-C), which was stented by using a Neuroform stent (Figure 1D and E). At his 2-year follow-up examination, the midbasilar aneurysm had grown, and he also had a basilar apex aneurysm (Figure 1F and H).

The patient underwent an orbitozygomatic craniotomy to expose the basilar artery for partial clip occlusion of the artery below the SCA and above the stent (Figure 2A and B). Blood flow to the basilar apex was supplemented with an STA-SCA bypass. Neurologically, the patient was grossly intact after surgery, and imaging confirmed a patent bypass (Figure 2C-D).

At his 3-year follow-up (Figure 3A and B) examination, the aneurysm had remained relatively stable. The patient and treating team elected to follow the aneurysm with serial imaging and to pursue no further surgical or endovascular treatment.

Case 1

A previously healthy 43-year-old man presented to our clinical service with symptoms suggestive of brainstem transient ischemic attacks. Evaluation revealed a giant fusiform aneurysm of the vertebrobasilar artery exerting significant mass effect on the brainstem (Figure 4A and B).

The patient underwent an orbitozygomatic craniotomy to expose the aneurysm. The basilar artery was partially occluded by placing a clip below the SCAs on the basilar artery. To augment flow to the basilar apex, an STA-PCA bypass was performed. Postoperatively, the

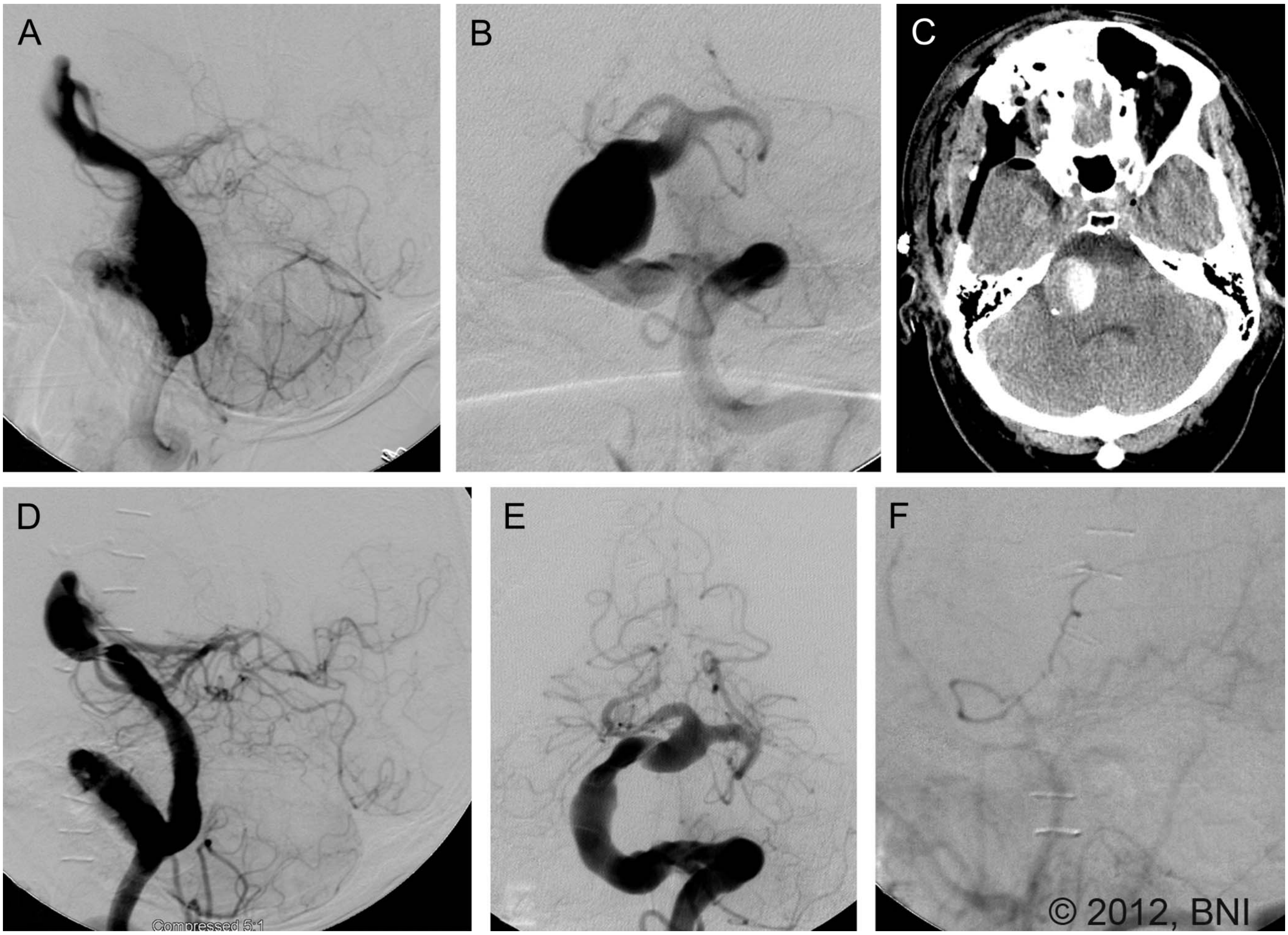

FIGURE 4. *Lateral (**A**) and anteroposterior (**B**) angiograms show a giant fusiform vertebrobasilar artery aneurysm involving the left vertebral artery and entire basilar trunk. Postoperative CT scan (**C**) demonstrates thrombosis of the aneurysm and significant mass effect on the brainstem. Postoperative lateral (**D**) and anteroposterior (**E**) angiograms show the placement of clip on the basilar artery below the SCAs. SCA, superior cerebellar artery. Postoperatively, the bypass was patent (**F**).* Used with permission from Barrow Neurological Institute.

Copyright © Congress of Neurological Surgeons. Unauthorized reproduction of this article is prohibited.

patient developed hydrocephalus from thrombosis of the aneurysm (Figure 4C-E). The bypass, however, was patent (Figure 4F).

After 5 years, the patient returned with symptoms of brainstem compression. Imaging revealed significant compression of the brainstem and further growth of the aneurysm (Figure 5). The basilar artery was partially occluded by the aneurysm clip.

The plan for re-treatment consisted of an STA-SCA bypass to augment flow to the brainstem, followed by endovascular occlusion of the basilar artery using a combination of coils and Onyx (ev3 Endovascular, Inc, Plymouth, Minnesota). The patient tolerated the procedure well. Postoperative imaging confirmed occlusion of the basilar artery and patency of the bypass (Figure 6A and B). Immediately after the operation, the patient was neurologically intact. In the intensive care unit, however, his condition deteriorated. Imaging showed subarachnoid and intraventricular hemorrhage (Figure 6D). The family elected to withdraw care.

Case 11

A previously healthy 45-year-old man presented to our service with symptoms suggestive of brainstem compression. CT (Figure 7A) and cerebral angiography (Figure 7B and C) revealed a giant, partially thrombosed, serpentine aneurysm of the basilar artery associated with significant compression of the brainstem.

The patient underwent an STA-SCA bypass with planned delayed endovascular occlusion of the portion of the basilar artery feeding the aneurysm. The STA-SCA bypass was performed uneventfully (Figure 7D and E). Postoperatively, the patient underwent cerebral angiography, which showed occlusion of the residual flow through the basilar artery aneurysm (Figure 7F-H). Given persistent symptoms of brainstem compression, the patient was returned to the operating room for decompression of the aneurysm (Figure 7I and J), which the patient tolerated well. After 19 years of follow-up, the patient is independent and working full-time.

DISCUSSION

Natural History and Treatment Options

Giant, complex basilar and vertebrobasilar junction aneurysms are uncommon but formidable surgical lesions.[8,9] Drake's results with the treatment of giant aneurysms suggest that their natural history is poor. If left untreated, almost 80% of such patients have

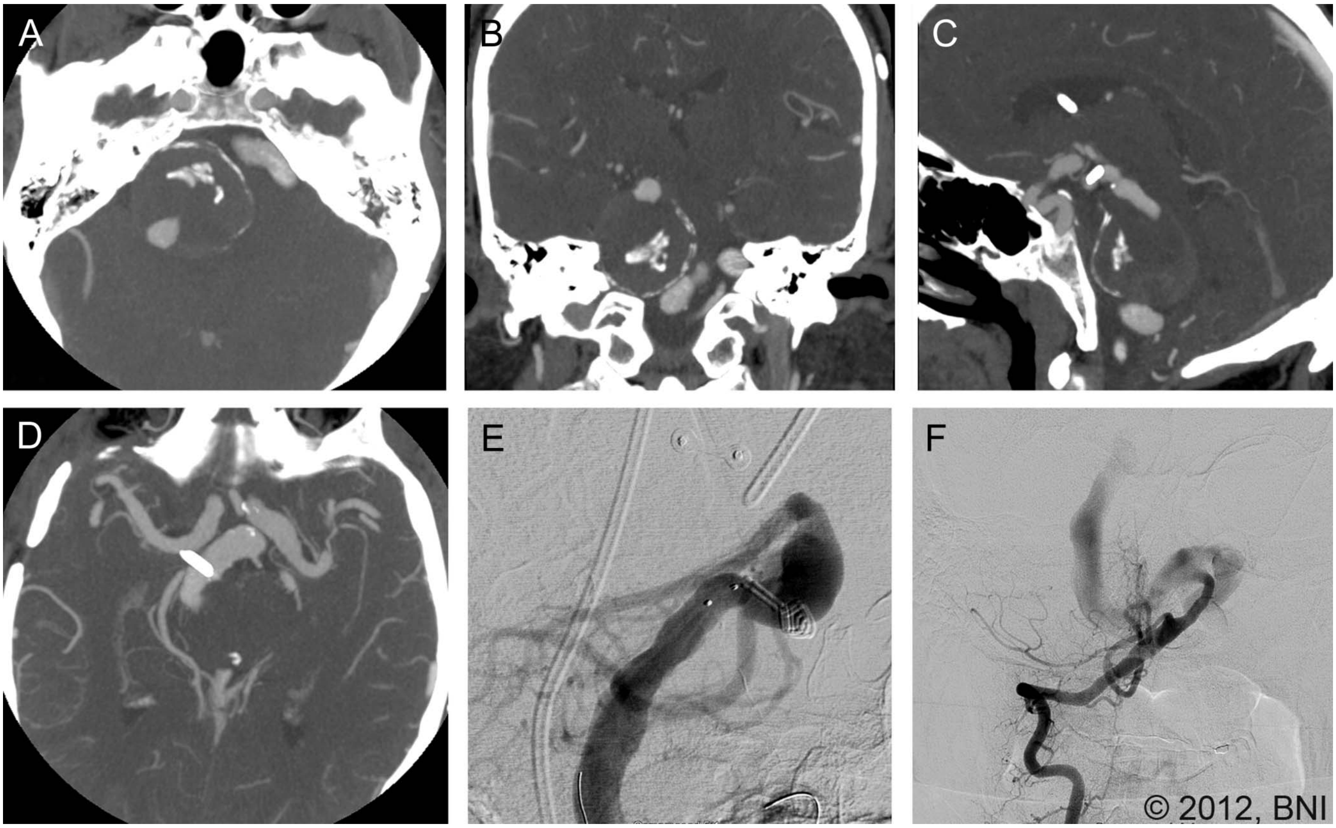

FIGURE 5. *Axial (**A**), coronal (**B**), and sagittal (**C**) CT scans demonstrate increased mass effect caused by the partially thrombosed, calcified aneurysm. CT angiogram (**D**) demonstrates partial occlusion of the basilar artery. Formal angiography confirmed the finding (**E** and **F**).* Used with permission from Barrow Neurological Institute.

Copyright © Congress of Neurological Surgeons. Unauthorized reproduction of this article is prohibited.

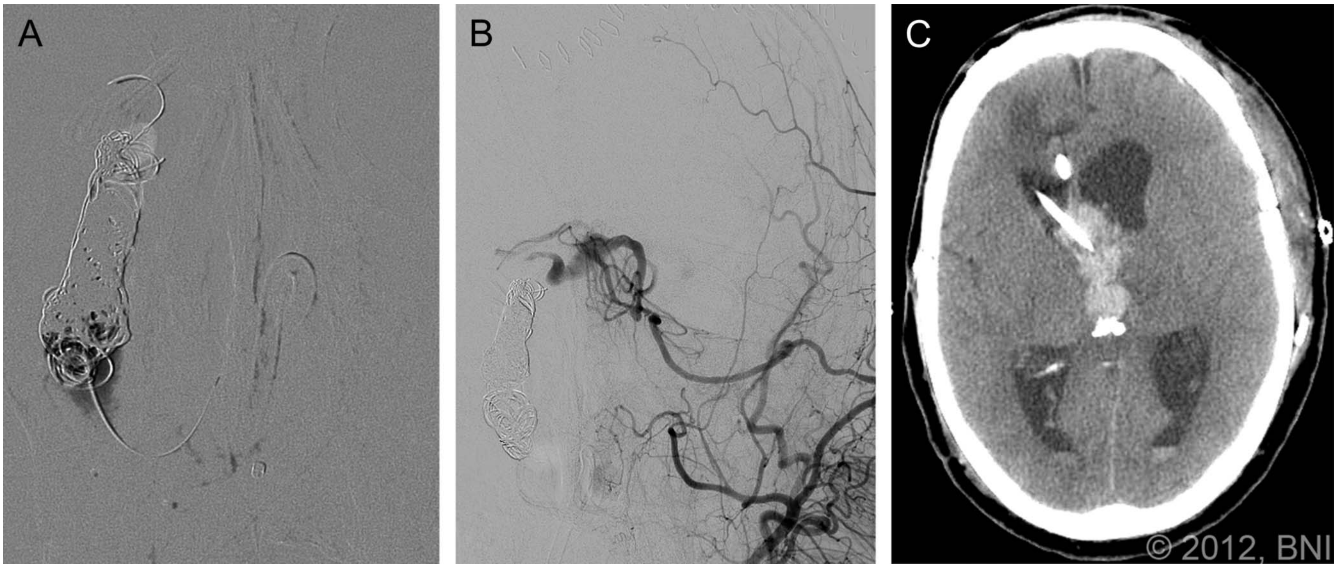

FIGURE 6. **A**, *anteroposterior angiograms demonstrate occlusion of the basilar artery after coil and Onyx embolization of the artery.* **B**, *oblique angiogram confirms patency of the STA-SCA bypass and occlusion of the basilar artery below the SCAs.* **C**, *postoperative CT scan obtained after the patient's condition deteriorated demonstrates subarachnoid and intraventricular hemorrhages. SCA, superior cerebellar artery; STA, superficial temporal artery.* Used with permission from Barrow Neurological Institute.

died of complications related to their aneurysm.[6,9] Neither standard treatment options such as clipping or clip reconstruction nor endovascular techniques have been successful in treating these aneurysms.[7] As initially advocated by Drake, Hunterian ligation can be performed as a salvage treatment for these lesions. However, occlusion of the basilar artery is associated with its own risks,[6] and inadequate collateral circulation can lead to devastating strokes.

Although endovascular therapies have been the predominant treatment modality for many posterior circulation aneurysms, large broad-neck aneurysms of the basilar apex (including those that incorporate major branches in the aneurysm dome), partially thrombosed aneurysms, and fusiform aneurysms of the vertebrobasilar junction may not be amenable to endovascular therapy.[10] After coil embolization, the recurrence rate of large, complex basilar apex aneurysms is about 40%, resulting in a significant rate of re-treatment. (The reader is referred to several excellent articles on this topic.[2,3,11-15]) The unpredictable flow dynamics at the basilar apex and the likely coil impaction that occurs as the aneurysm remodels frequently result in aneurysm recurrence after endovascular treatment. Furthermore, the initial enthusiasm for the use of flow-diverting stents in these aneurysms has been tempered by poor outcomes. In 1 report, 4 of 7 patients died, and 1 patient was severely disabled (overall poor outcome, 71.4%) after treatment with flow-diverting stents.[2] The poor outcomes with flow-diverting stents have been attributed to the partially thrombosed nature of the aneurysms, the need for anticoagulation which itself alters flow through the channels of the thrombosed aneurysm, and the unidirectional flow of blood into the aneurysm and their resultant growth.

Flow Reduction

When a large or complex posterior circulation aneurysm is not amenable to standard treatment options, proximal occlusion of the parent artery to alter blood flow in the aneurysm has been advocated as a safe and effective therapy.[5,6] The reduction in flow within the aneurysm provides an opportunity for the diseased vessel to thrombose gradually. The rich supply of perforators from the basilar artery, however, increases the risk of endovascular occlusion. Both microsurgical and endovascular occlusion must be performed with great caution to avoid compromising these perforating branches.[16,17] In patients with giant or complex basilar tip aneurysms, it is preferable to clip the basilar trunk below the exit of the SCAs if possible. If this option is infeasible, one or both vertebral arteries may be occluded. When both vertebral arteries must be occluded,[18] we advocate clip placement proximal to PICA on 1 vertebral artery and distal to PICA on the other. This strategy encourages flow in the proximal basilar artery and reduces the risk of acute basilar artery thrombosis. In the experience of the senior author (R.F.S.), this treatment modality is best suited for individuals who already have significant thrombosis of their aneurysms and recruitment to the territories normally fed by brainstem perforators. In these cases, flow reduction is less likely to lead to infarction of the brainstem.

Revascularization

Occlusion of the basilar artery can have devastating consequences if blood flow to the brainstem is inadequate. In such cases, a bypass to the posterior circulation may minimize the risk of

Copyright © Congress of Neurological Surgeons. Unauthorized reproduction of this article is prohibited.

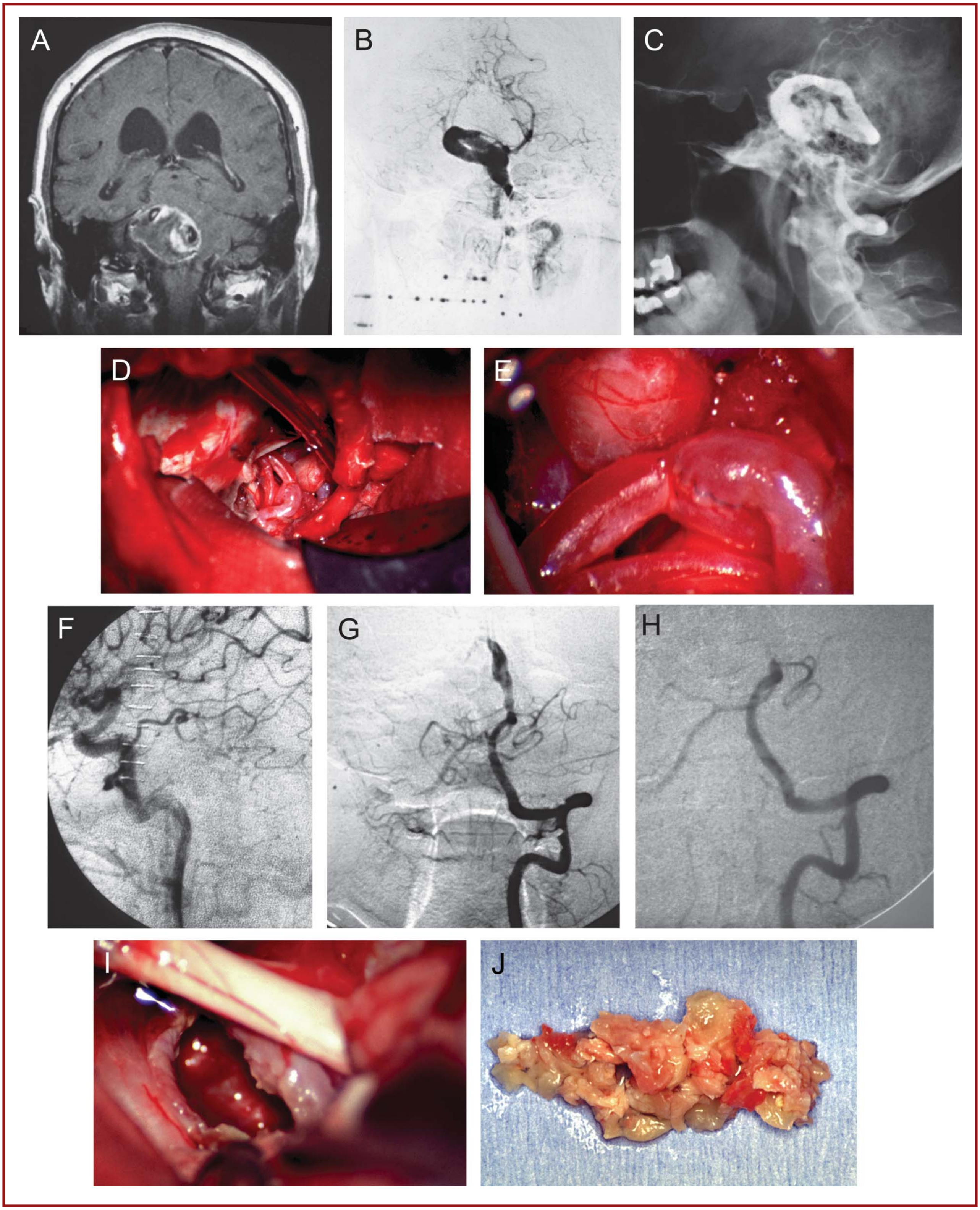

Copyright © Congress of Neurological Surgeons. Unauthorized reproduction of this article is prohibited.

FIGURE 7. **A**, *coronal CT reveals a giant, partially thrombosed aneurysm of the basilar artery with significant compression of the brainstem. Anteroposterior (***B***) and lateral (***C***) angiograms demonstrate the fusiform nature of the aneurysm. Intraoperative photographs (***D*** *and* ***E***) demonstrate the anastomosis between the STA and SCA used to revascularize the top of the brainstem. Postoperative lateral (***F***) and anteroposterior (***G*** *and* ***H***) angiograms demonstrate occlusion of the fusiform portion of the basilar artery. Intraoperative photographs (***I*** *and* ***J***) demonstrate decompression of the aneurysm contents causing persistent brainstem compression. SCA, superior cerebellar artery; STA, superficial temporal artery.* Used with permission from Spetzler RF, Koos WT, Richling B, and Lang J, eds. Color Atlas of Microneurosurgery. Vol 2. Cerebrovascular lesions (2e), New York: Thieme; 1997. Electronic rights courtesy of Barrow Neurological Institute.

neurologic complications.[8,19,20] Candidates for the recipient vessel include the SCA and PCA. The donor vessel used most often is the STA, but this vessel may not provide adequate perfusion in every case.[3]

EC-IC bypass to the SCA or PCA is technically challenging.[19] A surgical complication rate of 20% and mortality rate of 14% have been reported.[21] A bypass performed to prevent ischemia may lead to aneurysmal rupture by augmenting flow in excess of that needed to prevent ischemia. To treat these lesions, we combined occlusion of the inflow to the aneurysm at the basilar artery (n = 8) or distal to the PICA on the vertebral artery (n = 3) with an STA-SCA, STA-PCA or STA-SCA/STA-PCA bypass (Figure 8). A single bypass is typically adequate to revascularize the basilar apex, but, in rare cases (case 9), a double-barrel bypass may be necessary to ensure adequate perfusion of the brainstem.

Surgical Outcomes and Complications

Experience with the use of bypass and flow reversal or reduction for treating complex posterior circulation aneurysms is limited to case reports and small clinical series,[22-27] and long-term outcomes for this treatment option are unavailable. Indeed, as our experience demonstrates, despite initial treatment success, many patients return with progression of their disease and the need for further treatment followed by grim outcomes. Of our 4 patients who required further treatment for disease progression, 3 died. Steinberg noted that the mortality rate as a result of basilar occlusion in Drake's series was 24%.[28] The Stanford group has also reported a significant mortality rate (24%) associated with the treatment of fusiform aneurysms of the posterior circulation.[29]

When initial treatment and cases requiring further treatment in our series are combined, there were 4 cerebrovascular accidents, 6 cases of hydrocephalus, and multiple postoperative hemorrhages. The latter included 1 intraparenchymal hemorrhage, 2 epidural hemorrhages, and 1 combined subdural and epidural hemorrhage. The high incidence of postoperative extra-axial hemorrhages is likely related to the use of aspirin and heparin to prevent sudden postoperative thrombosis of aneurysms. Only 1 of 4 cerebrovascular accidents was associated with bypass occlusion. The remaining cases were likely embolic or thrombotic in origin. Hydrocephalus may simply be the result of aneurysmal thrombosis and the general inflammatory response associated with aneurysmal remodeling rather than a direct surgical complication. Nonetheless, it requires further surgical management and increases risks.

Functional Outcomes

As measured by the mRS, long-term outcome was heavily influenced by the mortality rate of 45%; however, in the 6 long-term survivors, the mean mRS was 2.5 (range, 1-4; median, 2.5). At the mean follow-up of 71.6 months, 3 of 11 patients were independent (mRS 0-2), and 3 were moderately to severely disabled (mRS 3-4). These outcomes indicate that treatment is associated with a high risk of morbidity and mortality.

Nonetheless, those who survive treatment have a better outcome than the natural history of untreated lesions. Recently, Miyamoto et al[23] used a similar strategy of flow reduction and bypass to treat basilar apex aneurysms. They noted stabilization or reduction of symptoms in 67% of their patients treated by using this strategy, whereas all patients who were treated conservatively had unfavorable outcomes. In another large study on the treatment of fusiform aneurysms of the posterior circulation, the rate of severe morbidity and mortality associated with treatment was 40%.[29] Based on an earlier experience from our institution with the surgical treatment of dolichoectatic and fusiform posterior circulation aneurysms, which included vertebral, PICA, and anterior inferior cerebellar artery aneurysms, only 65% of the patients had good outcomes.[7] Given the challenges with existing treatment strategies, conservative treatment of this patient population is an acceptable and appropriate option. Those patients that do survive the treatment, however, are likely to have acceptable long-term outcomes. Regardless of treatment, the high rates of recurrence and progression of disease in these patients mandate their long-term angiographic follow-up.

CONCLUSION

Despite initial successful treatment, the long-term outcomes of patients with complex and giant basilar and vertebrobasilar junction aneurysms treated via bypass and flow reversal or reduction remain guarded. Re-treatment(s) for aneurysm enlargement should be expected and is associated with a high risk of morbidity and mortality. In our experience, this treatment strategy is best suited for individuals who have generated collaterals to brainstem perforators, at times via channels within the thrombosed aneurysm itself. Given the poor natural history of giant, complex aneurysms of the posterior circulation and the lack of better alternatives, this surgical therapy may afford marginal improvement over the natural history of the untreated lesion. Given the high risk of recurrence or progression and aneurysmal growth, we recommend life-long angiographic follow-up of patients with these challenging lesions.

Copyright © Congress of Neurological Surgeons. Unauthorized reproduction of this article is prohibited.

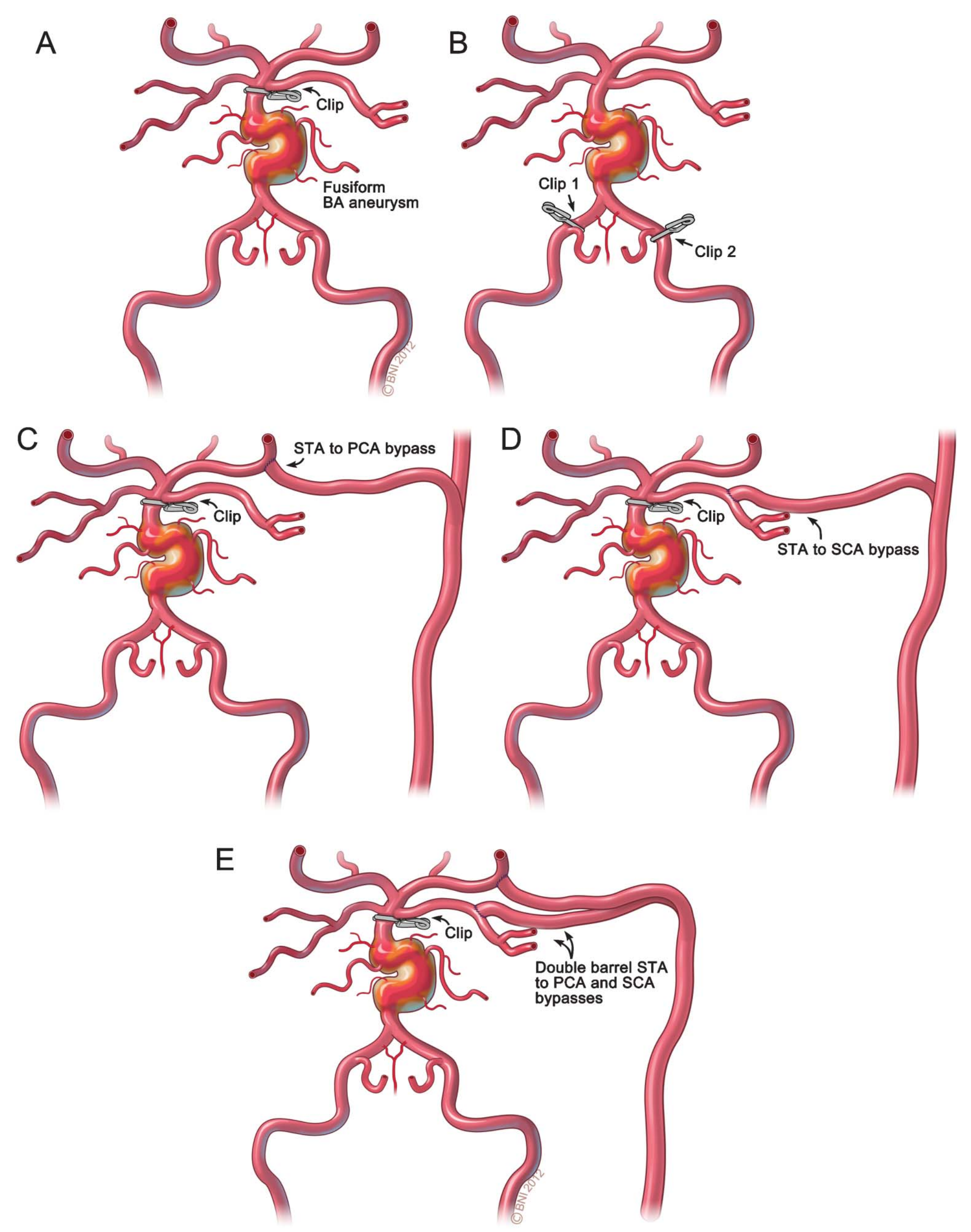

FIGURE 8. *Schematic of treatment strategy used for treating these complex aneurysms. Flow can be reduced by occlusion at the level of the vertebral artery (***A***) or basilar artery (***B***). In patients with giant or complex basilar tip aneurysms, it is preferable to clip the basilar trunk below the exit of the SCAs if possible. If this option is infeasible, one or both vertebral arteries may be occluded. When both vertebral arteries must be occluded, we advocate clip placement proximal to PICA on 1 vertebral artery and distal to PICA on the other. This strategy encourages flow in the proximal basilar artery and reduces the risk of acute basilar artery thrombosis. To revascularize the brainstem, various bypass options, including an STA-SCA (***C***), STA-PCA (***D***), or double-barrel STA-SCA/STA-PCA (***E***) bypass can be combined with options* **A** *or* **B**. *SCA, superior cerebellar artery; STA, superficial temporal artery; PCA, posterior cerebellar artery; BA, basilar artery.* Used with permission from Barrow Neurological Institute.

Copyright © Congress of Neurological Surgeons. Unauthorized reproduction of this article is prohibited.

Disclosure

The authors have no personal, financial, or institutional interest in any of the drugs, materials, or devices described in this article.

REFERENCES

1. Fiorella D, Kelly ME, Albuquerque FC, Nelson PK. Curative reconstruction of a giant midbasilar trunk aneurysm with the pipeline embolization device. *Neurosurgery*. 2009;64(2):212-217.
2. Siddiqui AH, Abla AA, Kan P, et al. Panacea or problem: flow diverters in the treatment of symptomatic large or giant fusiform vertebrobasilar aneurysms. *J Neurosurg.* 2012;116(6):1258-1266.
3. Klisch J, Turk A, Turner R, Woo HH, Fiorella D. Very late thrombosis of flow-diverting constructs after the treatment of large fusiform posterior circulation aneurysms. *AJNR Am J Neuroradiol.* 2011;32(4):627-632.
4. Fiorella D, Hsu D, Woo HH, Tarr RW, Nelson PK. Very late thrombosis of a pipeline embolization device construct: case report. *Neurosurgery*. 2010;67(3 suppl operative):onsE313-onsE314.
5. Kellner CP, Haque RM, Meyers PM, Lavine SD, Connolly ES Jr, Solomon RA. Complex basilar artery aneurysms treated using surgical basilar occlusion: a modern case series. Clinical article. *J Neurosurg.* 2011;115(2):319-327.
6. Steinberg GK, Drake CG, Peerless SJ. Deliberate basilar or vertebral artery occlusion in the treatment of intracranial aneurysms. Immediate results and long-term outcome in 201 patients. *J Neurosurg.* 1993;79(2):161-173.
7. Anson JA, Lawton MT, Spetzler RF. Characteristics and surgical treatment of dolichoectatic and fusiform aneurysms. *J Neurosurg.* 1996;84(2):185-193.
8. Lawton MT, Hamilton MG, Morcos JJ, Spetzler RF. Revascularization and aneurysm surgery: current techniques, indications, and outcome. *Neurosurgery*. 1996;38(1):83-92.
9. Drake CG, Peerless SJ. Giant fusiform intracranial aneurysms: review of 120 patients treated surgically from 1965 to 1992. *J Neurosurg.* 1997;87(2):141-162.
10. Krisht AF, Krayenbühl N, Sercl D, Bikmaz K, Kadri PA. Results of microsurgical clipping of 50 high complexity basilar apex aneurysms. *Neurosurgery*. 2007;60(2):242-250.
11. Bavinzski G, Killer M, Gruber A, Reinprecht A, Gross CE, Richling B. Treatment of basilar artery bifurcation aneurysms by using Guglielmi detachable coils: a 6-year experience. *J Neurosurg.* 1999;90(5):843-852.
12. Bavinzski G, Richling B, Gruber A, Killer M, Levy D. Endosaccular occlusion of basilar artery bifurcation aneurysms using electrically detachable coils. *Acta Neurochir (Wien).* 1995;134(3-4):184-189.
13. Henkes H, Fischer S, Mariushi W, et al. Angiographic and clinical results in 316 coil-treated basilar artery bifurcation aneurysms. *J Neurosurg.* 2005;103(6):990-999.
14. Lubicz B, Leclerc X, Gauvrit JY, Lejeune JP, Pruvo JP. Giant vertebrobasilar aneurysms: endovascular treatment and long-term follow-up. *Neurosurgery*. 2004;55(2):316-323.
15. Wakhloo AK, Mandell J, Gounis MJ, et al. Stent-assisted reconstructive endovascular repair of cranial fusiform atherosclerotic and dissecting aneurysms: long-term clinical and angiographic follow-up. *Stroke*. 2008;39(12):3288-3296.
16. Drake CG. Further experience with surgical treatment of aneurysm of the basilar artery. *J Neurosurg.* 1968;29(4):372-392.
17. Drake CG. The surgical treatment of aneurysms of the basilar artery. *J Neurosurg.* 1968;29(4):436-446.
18. Omahen DA, Findlay JM. A giant fusiform basilar aneurysm treated by bilateral vertebral artery occlusion. *J Clin Neurosci.* 2004;11(3):324-328.
19. Evans JJ, Sekhar LN, Rak R, Stimac D. Bypass grafting and revascularization in the management of posterior circulation aneurysms. *Neurosurgery*. 2004;55(5):1036-1049.
20. Sekhar LN, Natarajan SK, Ellenbogen RG, Ghodke B. Cerebral revascularization for ischemia, aneurysms, and cranial base tumors. *Neurosurgery*. 2008;62(6 suppl 3):1373-1408.
21. Hopkins LN, Budny JL. Complications of intracranial bypass for vertebrobasilar insufficiency. *J Neurosurg.* 1989;70(2):207-211.
22. Ramanathan D, Ciporen J, Ghodke B, Ellenbogen RG, Sekhar LN. Treatment of coil embolization failed recurrent giant basilar tip aneurysms with bypass and surgical occlusion. *J Neurointerv Surg.* 2010;2(3):237-241.
23. Miyamoto S, Funaki T, Iihara K, Takahashi JC. Successful obliteration and shrinkage of giant partially thrombosed basilar artery aneurysms through a tailored flow reduction strategy with bypass surgery. *J Neurosurg.* 2011;114(4):1028-1036.
24. Horie N, Kitagawa N, Morikawa M, et al. Giant thrombosed fusiform aneurysm at the basilar trunk successfully treated with endovascular coil occlusion following bypass surgery: a case report and review of the literature. *Neurol Res.* 2007;29(8):842-846.
25. Terasaka S, Itamoto K, Houkin K. Basilar trunk aneurysm surgically treated with anterior petrosectomy and external carotid artery-to-posterior cerebral artery bypass: technical note. *Neurosurgery*. 2002;51(4):1083-1087.
26. Ewald C, Kühne D, Hassler W. Giant basilar artery aneurysms encorporating the posterior cerebral artery: bypass surgery and coil occlusion: two case reports. *Neurol Med Chir (Tokyo).* 1998;(38 suppl):83-85.
27. Greene KA, Marciano FF, Hamilton MG, Herman JM, Rekate HL, Spetzler RF. Cardiopulmonary bypass, hypothermic circulatory arrest and barbiturate cerebral protection for the treatment of giant vertebrobasilar aneurysms in children. *Pediatr Neurosurg.* 1994;21(2):124-133.
28. O'Shaughnessy BA, Getch CC, Bendok BR, Parkinson RJ, Batjer HH. Progressive growth of a giant dolichoectatic vertebrobasilar artery aneurysm after complete Hunterian occlusion of the posterior circulation: case report. *Neurosurgery*. 2004;55(5):1223.
29. Coert BA, Chang SD, Do HM, Marks MP, Steinberg GK. Surgical and endovascular management of symptomatic posterior circulation fusiform aneurysms. *J Neurosurg.* 2007;106(5):855-865.

COMMENTS

The authors are to be commended on this report, treating 11 patients with 12 posterior fossa "complex" and giant aneurysms over an 18-year period. Patients who were not candidates for proximal vessel occlusion (either failed BTO, or were thought to have inadequate collateral circulation on angiography) were treated with an STA bypass to either the PCA or SCA. Six patients had complete, and 3 patients had partial occlusion of the basilar artery. Three patients had occlusion of the vertebral arteries. The bypass patency was 92%. Of 11 patients, 5 died.

We know that symptomatic posterior fossa aneurysms carry a grave prognosis. Patients can present with substantial neurological deficits from brainstem compression. They are problematically large, calcified, and may have a significant intraluminal thrombus. Perforator incorporation into the aneurysmal wall, and their subsequent occlusion plays a major part in the preoperative stroke presentation. Direct surgical clipping, with or without hypothermic cardiorespiratory arrest, carries a significant perioperative morbidity and mortality. From personal experience, I have also noted an increase in the operative risk when an aneurysm is calcified, opened, or an attempt is made at a thrombectomy.

Because of the small number of patients in this series, it is not possible to identify characteristics that predict aneurysmal growth, nor can we determine whether fusiform or saccular aneurysms have different prognoses. Do vertebrobasilar, basilar trunk ,or apex aneurysms behave in the same way? Could a high-flow EC-IC bypass have made a difference? How were patients with poor STAs treated? These questions remain to be answered.

Despite innovative surgical and endovascular options, we have not found a satisfactorily safe treatment. Nevertheless, and for the present time, I concur with the authors that an EC-IC bypass might be one of the safer ways to treat these formidable lesions.

Jafar Jewad Jafar
New York, New York

Giant vertebrobasilar aneurysms are fortunately uncommon lesions, so that even this world-renowned neurovascular unit has just 11 patients to report. It is probably safe to assume that the microsurgical strategies used here, namely partial or complete basilar artery occlusion above the aneurysm sac, or bilateral vertebral artery occlusion below, with uniform use of bypasses (all patients, regardless of test balloon occlusion

Copyright © Congress of Neurological Surgeons. Unauthorized reproduction of this article is prohibited.

results) are the product of not just their own thinking and results, but also born from the experience of others who have shared the podium on the subject at conferences and courses (including Dr Drake many years ago). Despite major efforts, the overall results are, as the authors emphasize, disappointingly poor, for a number of fairly obvious reasons, including large aneurysm sizes, their difficult location to access, and the underlying brainstem often partly supplied by perforators arising from the aneurysm body itself.

Given that, it is worth reminding ourselves that large aneurysms in the posterior circulation have a mixed etiological background (atherosclerotic, dissecting, dysplastic, developmental) and that, although they are not always readily distinguishable, they sometimes can be based on patient age, clinical presentation, sac morphology, wall thickness, aneurysm content, etc, and that the natural history varies accordingly. They do not all have a relentless and malignant clinical course. Aneurysms that present with compression, ischemia or maybe a combination of both do not always require intervention immediately, but, instead, medical management and follow-up, and they can remain stable for years. However, when patients survive a rupture from 1 of these aneurysms, or compressive symptoms are clearly progressive and disabling, we are forced to consider more aggressive intervention, and the discussion centers around endovascular, microsurgical, or perhaps combined approaches. As always, the vascular anatomy is critical for decision making, and a decent posterior communicating artery (making a difficult bypass unnecessary) combined with a partially or largely thrombosed aneurysm (already having gradually claimed the perforators, the brainstem having acquired collaterals) is maybe the best case scenario allowing for a single or staged proximal Hunterian ligation. This article is a very valuable resource when faced with such patients.

J Max Findlay
Edmonton, Canada

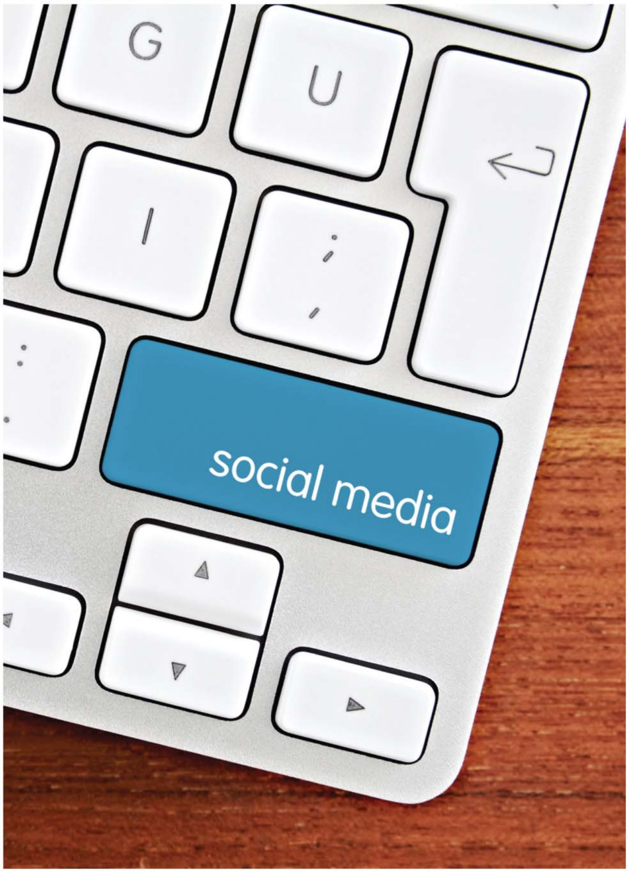

Like.

NEUROSURGERY® Social Media Channels.

For news and updates on recently-accepted content, follow NEUROSURGERY® on: FaceBook, Twitter, Google+, LinkedIn, Reddit or Digg.

Subscribe to the NEUROSURGERY Report weekly e-newsletter at *neurosurgerycns.wordpress.com*

For supplemental video content, visit the NEUROSURGERY® video channel on YouTube.

view with layar

NEUROSURGERY
THE REGISTER OF THE NEUROSURGICAL MEME

Copyright © Congress of Neurological Surgeons. Unauthorized reproduction of this article is prohibited.
