# Supplementary material for: Management strategies and outcomes of basilar trunk aneurysms: a systematic review and meta-analysis
Source: Langenbecks Arch Surg. 2026 Jan 20;411(1):71. doi: 10.1007/s00423-025-03959-3 (PMC12852281; doi:10.1007/s00423-025-03959-3)
Supplement: Supplementary file 1 — Supplementary file1 (ZIP 55327 KB) [file 423_2025_3959_MOESM1_ESM.zip › literature.zip!/meta/lawton2015.pdf]

# Bypass Surgery for the Treatment of Dolichoectatic Basilar Trunk Aneurysms: A Work in Progress

**Michael T. Lawton, MD***
**Adib A. Abla, MD***
**W. Caleb Rutledge, MD***
**Arnau Benet, MD***
**Zsolt Zador, MD***
**Vitaliy L. Rayz, PhD‡**
**David Saloner, PhD‡**
**Van V. Halbach, MD§**

Departments of *Neurological Surgery, ‡Radiology and Biomedical Imaging, and §Interventional Neuroradiology, University of California, San Francisco, San Francisco, California

**Correspondence:**
Michael T. Lawton, MD,
Department of Neurosurgery,
University of California,
San Francisco,
505 Parnassus Ave,
Room M780,
San Francisco, CA 94143.
E-mail: michael.lawton@ucsf.edu

**Received,** June 18, 2015.
**Accepted,** October 18, 2015.
**Published Online,** December 14, 2015.

Copyright © 2015 by the Congress of Neurological Surgeons.

**BACKGROUND:** The treatment of dolichoectatic basilar trunk aneurysms has been ineffectual or morbid due to nonsaccular morphology, deep location, and involvement of brainstem perforators. Treatment with bypass surgery has been advocated to eliminate malignant hemodynamics and to stabilize aneurysm growth.
**OBJECTIVE:** To validate that flow alteration with bypass and parent artery occlusion favorably impacts aneurysm progression.
**METHODS:** Surgical management evolved in 3 phases, each with different hemodynamic alterations.
**RESULTS:** During a 17-year period, 37 patients with dolichoectatic basilar trunk aneurysms were retrospectively identified, of whom 21 patients were observed, 12 treated immediately, and 4 selected for treatment after clinical progression. In phase 1, flow reversal was overly thrombogenic, despite heparin (N = 5, final mortality, 100%). In phase 2, flow reduction with intracranial-to-intracranial bypass was safer than flow reversal, but did not prevent progressive aneurysm enlargement (N = 3, final mortality 67%). In phase 3, distal clip occlusion of the basilar trunk aneurysm preserved anterograde flow in the aneurysm without rupture, but reduced flow threatened perforator patency, despite treatment with clopidogrel (N = 8, final mortality 62%).
**CONCLUSION:** Shifting treatment strategy for dolichoectatic basilar trunk aneurysms improved surgical (80% to 50%) and final mortalities (100% to 62%), with stabilization of aneurysms in the phase 3 survivors. Good outcomes are determined by perforator preservation and mitigating aneurysm thrombosis. Occlusion techniques with increased distal run-off seem to benefit perforators. The treatment of dolichoectatic basilar trunk aneurysms can advance through concentrated management in dedicated centers, concerted efforts to study morphology and hemodynamics with computational methods, and widespread collection of registry data.

**KEY WORDS:** Atherosclerosis, Basilar artery aneurysm, Basilar trunk, Bypass, Dolichoectasia, Giant aneurysm, Fusiform aneurysm, Revascularization

*Neurosurgery 79:83–99, 2016* DOI: 10.1227/NEU.0000000000001175 www.neurosurgery-online.com

*"Diseases desperate grown must by desperate appliance be relieved, or not at all."*
—Shakespeare, from *Hamlet*, Act IV, Scene 3

**ABBREVIATIONS: 4D PC-MRI,** time-resolved phase-contrast MRI; **AICA,** anterior inferior cerebellar artery; **CE-MRA,** high-resolution contrast-enhanced MR angiography; **EC-IC,** extracranial-to-intracranial bypass; **MCA,** middle cerebral artery; **MR,** magnetic resonance; **mRS,** modified Rankin Scale; **PCA,** posterior cerebral artery; **PICA,** posterior inferior cerebellar artery; **SCA,** superior cerebellar artery; **STA,** superficial temporal artery; **VA,** vertebral artery

There is no aneurysm more daunting than the dolichoectatic basilar trunk aneurysm. The pathogenesis of this aneurysm is not clear, but it seems to originate from circumferential atherosclerotic degeneration of the basilar artery, resulting in dilatation, elongation, and dolichoectasia. Progressive structural and morphological changes in the arterial walls are accompanied by hemodynamic changes, generating regions of abnormal flow velocity, wall shear stress, vorticity, turbulence, and stagnation, which in turn might exacerbate arterial degeneration through degradation, thrombus formation, and inflammation.[1,2] In addition to these structural, morphological,



Copyright © Congress of Neurological Surgeons. Unauthorized reproduction of this article is prohibited.

and hemodynamic changes, this disease may be fueled by anatomical variations, such as the absence of posterior communicating arteries and a competent circle of Willis, or by other diseases, such as arterial dissection or dysplasia. This pathogenesis can initiate a vicious cycle of clinical deterioration that results in aneurysm enlargement, brainstem and cranial nerve compression, thinning of the basilar arterial walls with aneurysmal rupture, and/or intraluminal thrombosis with perforator infarctions and embolic strokes. Patients may experience progressive neurological deficits from a growing mass or hydrocephalus, the sudden ictus of subarachnoid hemorrhage, or a stepwise decline from multiple strokes. Dolichoectatic basilar trunk aneurysms have a dismal natural history, with some estimates of 2-year survival rates as low as 20%.[3]

The treatment of these lesions has been either ineffectual or staggeringly morbid. Unlike saccular aneurysms with clippable necks, dolichoectatic aneurysms are unclippable and their location makes them poorly accessible surgically. Reconstruction with clips is possible with dolichoectatic aneurysms that are eccentric or involve only a portion of the arterial wall, but such lesions are rare, and it is difficult to exclude all abnormal tissue while preserving perforators. These lesions are often accessible endovascularly, but they are equally uncoilable and adjunctive stents or balloon remodeling techniques fail to improve their coilability. Hunterian ligation of one or both vertebral arteries (VA) or the proximal basilar artery was practiced by Charles Drake,[4] but this treatment depended on a sizable posterior communicating arteries (>2 mm diameter) and its long-term efficacy was poorly established. Some have performed arterial transposition to decompress the brainstem or cranial nerves,[5] but dolichoectatic basilar arteries are often immobile and, at best, this intervention offers only symptomatic relief temporarily. Advancements in endovascular devices have led to recent attempts to treat these aneurysms with flow diverters, but early results have been poor and have not matched the excellent results observed at other locations lacking perforators.[3,6-15] Failed efforts to treat this disease have led many to conclude that this problem is unsolvable and that these patients should be managed medically with aspirin, blood pressure control, and cholesterol-lowering agents, and possibly with ventriculoperitoneal shunts when hydrocephalus becomes symptomatic.

Treatment of dolichoectatic basilar trunk aneurysms with bypass surgery has been advocated as a means of eliminating malignant hemodynamics, redirecting flow away from areas of aneurysmal degeneration, and promoting intraluminal thrombosis, thereby stabilizing aneurysm growth and preventing hemorrhage.[16-18] The strategy consists of an extracranial-to-intracranial bypass (EC-IC), usually a superficial temporal artery (STA) bypass to either the superior cerebellar artery (SCA) or posterior cerebral artery (PCA), followed by Hunterian ligation of the VA or basilar trunk proximally. Published experiences with these treatments are limited to case reports and small series, each with small variations in strategy or technique.[16,17,19-32] These experiences are associated with significant morbidity and no clear solution has been demonstrated. In this report, we add our experience with dolichoectatic basilar trunk aneurysms to the literature. We embraced the concept that flow alteration by means of bypass and parent artery occlusion would favorably impact disease progression, and we sought to validate others' experiences with this bypass/Hunterian ligation strategy or improve upon it in an effort to find an answer to this disease.

## METHODS

### Patients

This study was approved by the Institutional Review Board at the University of California, San Francisco and conducted in compliance with Health Insurance Portability and Accountability Act regulations. Patients with basilar artery aneurysms were identified from a prospectively maintained database of the Vascular Neurosurgery Service, and those with dolichoectatic basilar trunk aneurysms were included. Dolichoectatic aneurysms were defined as fusiform, circumferential dilatations of the basilar trunk. Patients with aneurysms involving the basilar bifurcation, PCA, and SCA were excluded, as were those with aneurysms involving the vertebral arteries. Patients with saccular aneurysms on the basilar trunk, such as those originating from the anterior inferior cerebellar artery (AICA), vertebrobasilar junction, or basilar perforators, were also excluded. Excluded patients consisted of 9 patients with saccular basilar trunk aneurysms that were clipped microsurgically (mean diameter, 10 mm); 14 with AICA aneurysms (3 proximal AICA aneurysms and 11 distal aneurysms) that were clipped (n = 7) or trapped (n = 7); and 2 patients with basilar perforating artery aneurysms that were trapped. Four other surgical patients with basilar trunk dolichoectatic aneurysms were excluded: 3 patients who underwent partial thrombectomy and clip reconstruction, and 1 patient who underwent combined endonasal-transclival and retrosigmoid mobilization of the aneurysm for brainstem decompression.

Medical records, including preoperative and postoperative radiographic imaging, operative reports, intraoperative photographs, videos, hospital course, and outpatient visits, were reviewed retrospectively. Clinicians not directly involved in the patients' care (A.A.A. and W.C.R.) performed all outcome assessments using the modified Rankin Scale (mRS). Patient outcomes were reported using standard descriptive statistics.

### Management Algorithm

The diagnosis of dolichoectatic basilar trunk aneurysm was established with computed tomographic scans, magnetic resonance (MR) imaging, and digital subtraction angiography. Patients with mild or stable symptoms were managed conservatively, whereas those with severe or progressive symptoms were managed surgically (Figure 1). High-resolution contrast-enhanced MR angiography (CE-MRA) generated volumetric models to measure aneurysm growth during observation. In addition, time-resolved phase-contrast MRI (4D PC-MRI) measured aneurysm hemodynamics. Observed patients were imaged annually, and those with aneurysm growth, symptom progression, or rupture were considered for treatment. In contrast, patients presenting with severe or progressive symptoms were immediately considered for treatment. 4D PC-MRI was again used to measure aneurysm hemodynamics and to provide flow boundary conditions for numerical simulations of the altered hemodynamics associated with bypass options.[1,2] Simulations were used to guide the selection of bypasses.



Copyright © Congress of Neurological Surgeons. Unauthorized reproduction of this article is prohibited.

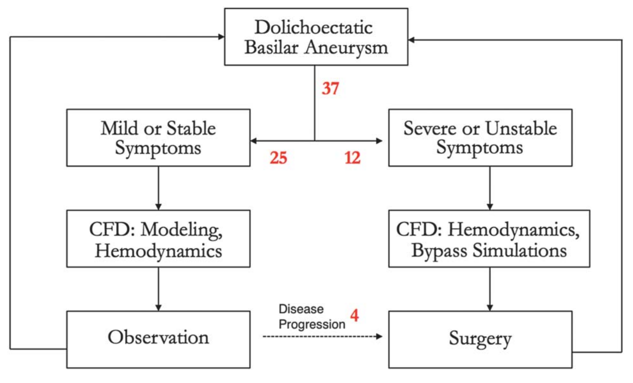


**FIGURE 1.** *Management algorithm for 37 patients with dolichoectatic basilar trunk aneurysms. CFD, computational fluid dynamics.*

Surgical management evolved in 3 distinct phases, each with different exposures, bypasses, methods of aneurysm occlusion, hemodynamic alterations, and postoperative managements (Figure 2 and Table 1). Surgical bypasses consisted of EC-IC bypasses (STA-SCA and STA-PCA bypasses in phase 1) or intracranial-to-intracranial bypasses using an interposition graft (VA-SCA in phase 2 and middle cerebral artery [MCA]-PCA bypasses in phase 3). When the STA was inadequate, the external carotid artery or other cervical donor arteries were used with a saphenous vein graft. For shorter intracranial-to-intracranial grafts, the radial artery was used preferentially in patients with a competent palmar arch on preoperative Allen testing. Bypasses were performed through orbitozygomatic craniotomies with either subtemporal (phase 1) or pretemporal and transsylvian approaches (phase 3), or through a combined far lateral and subtemporal craniotomy for the VA-SCA bypass (phase 2). The indication for bypass in all patients was the planned occlusion of the aneurysm's parent artery in the setting of diminutive or absent posterior communicating arteries. Aneurysm occlusion was performed in several ways: endovascularly in a second interventional stage, usually proximally in the vertebral arteries bilaterally (phase 1); surgically at the time of bypass, in the dominant vertebral artery (phase 2); or surgically at the time of bypass, at the distal aneurysm outflow, below the basilar apex and proximal to the SCAs (phase 3).

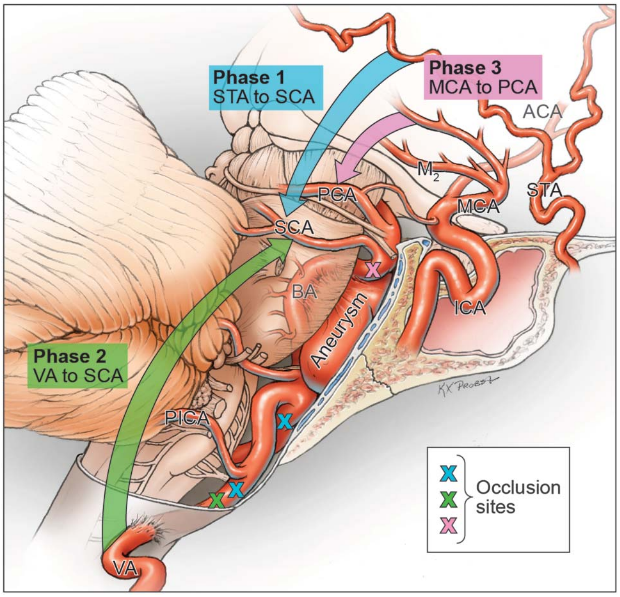


**FIGURE 2.** *Summary of bypasses used to treat dolichoectatic basilar trunk aneurysms and the evolution in surgical management from extracranial-intracranial bypasses (superficial temporal artery [STA] to superior cerebellar artery [SCA] or posterior cerebral artery [PCA], phase 1) to intracranial-intracranial bypasses with interposition grafts (V3 segment of vertebral artery [VA] to SCA, phase 2, and middle cerebral artery [MCA] to PCA, phase 3). ACA, anterior cerebral artery; BA, basilar artery; ICA, internal carotid artery.*

During the anastomosis, heparin solution was irrigated locally. Systemic heparin was not used. Bypasses were checked for patency with indocyanine green videoangiography and Doppler ultrasound. During clamp time, patients were maintained in barbiturate- or propofol-induced electroencephalogram burst suppression with mild elevation of the systolic arterial blood pressure. Postoperative angiography evaluated patency of the bypass and obliteration of the aneurysm and patients were maintained on acetylsalicylic acid 325 mg indefinitely. Endovascular Hunterian occlusions were typically performed 3 days after bypass surgery, and patients were routinely heparinized during and for 3 days after their endovascular procedure (phase 1). Clopidogrel (Plavix) was used later in the series as described below (phase 3).

## Computational Fluid Dynamics

Patient-specific computational fluid dynamic modeling was used to assess postoperative flows that would result from alternative surgical options. High-resolution CE-MRA produced patient-specific vascular geometries of the aneurysm and its proximal and distal vessels. The branches of the basilar trunk, including the AICAs and pontine perforators, could not be imaged because of the limitations in imaging resolution, and idealized geometries of the AICAs and perforators were added, according to modeling procedures described previously.[33] CE-MRA data were segmented using in-house software, and 3-dimensional surfaces corresponding to the luminal boundaries were obtained for each patient. The idealized geometries of the basilar trunk branches were then added using Geomagic Design software (3D Systems, Rock Hill, South Carolina).

Patient-specific inlet flow rates required for the flow computations were measured with through-plane PC-MRI technique, providing flow waveforms prescribing the inlet velocities through the cardiac cycle for each vertebral artery. Our previous studies[1] demonstrated that the flow rates ratio of the jets entering from the VA has a major effect on the flow field in the basilar trunk and, subsequently, in the aneurysm. In most of the patients, preoperative flow was also measured with 4D PC-MRI, providing time-resolved velocity field in 3 dimensions. Numerical flow simulations were conducted in the preoperative vertebrobasilar geometries and compared to in vivo PC-MRI measurements to ensure the accuracy of the computations. Postoperative flow simulations were then carried out for the alternative surgical options. Contrast agent transport was simulated by solving the advection diffusion equation along with the Navier-Stokes equations describing the flow. This numerical technique provided flow residence times and determined intra-aneurysmal regions of flow separation and stagnation, which were found to correlate with thrombus deposition.[2]



Copyright © Congress of Neurological Surgeons. Unauthorized reproduction of this article is prohibited.

**TABLE 1. Summary of Phases of Management of Dolichoectatic Basilar Trunk Aneurysms**[a]

| | Phase 1 | Phase 2 | Phase 3 |
|---|---|---|---|
| Exposure | OZ/subtemporal | Far lateral/subtemporal | OZ/pretemporal |
| Bypass | EC-IC: STA-SCA, STA-PCA | IC-IC: V3 VA-SCA | IC-IC: MCA-PCA |
| Aneurysm occlusion | Endovascular (proximal, staged) | Surgical (proximal, dominant VA) | Surgical (distal, basilar artery) |
| Basilar flow | Retrograde | Anterograde | Anterograde |
| Hemodynamic alteration | Reversal | Reduction (mild-moderate) | Reduction (severe) |
| Postoperative management | ASA, heparin | ASA | ASA, Plavix |

[a]ASA, acetylsalicylic acid; EC-IC, extracranial-to-intracranial bypass; IC-IC, intracranial-to-intracranial; MCA-PCA, middle cerebral artery to posterior cerebral artery; OZ, orbitozygomatic craniotomy; STA-PCA, superficial temporal artery to posterior cerebral artery; STA-SCA, superficial temporal artery to superior cerebellar artery; VA-SCA, vertebral artery-superior cerebellar artery.

# RESULTS

## Patients (Participants) and Baseline Characteristics

During a 17-year period from September 1997 to September 2014, 37 patients with dolichoectatic basilar trunk aneurysms were identified, of whom 25 had mild or stable symptoms and were observed (Figure 1). The average patient was 63 years old (range, 34-80) and there was a male predominance (16 men and 9 women). Patients presented with strokes, brainstem compression, or incidentally during workup for memory loss or headache. The mean mRS at presentation was 1.5 (range, 0-4). The mean aneurysm diameter was 1.7 cm (range, 0.4-5.0), including 7 giant aneurysms and 9 thrombotic aneurysms.

These patients were observed for a mean duration of 3.7 years (range, 3 months-11.7 years). During that time, 7 patients (28%) remained neurologically stable, and 11 patients (44%) deteriorated with new disabilities. Five patients (20%) died of the following causes: myocardial infarction (n = 1), complications from surgery (n = 1), and unknown (n = 3). Two patients (8%) were lost to follow-up. The mean mRS in the observation cohort at last follow-up was 2.8. Changes in mRS scores are shown (Figure 3). Four patients (16%) with deteriorating condition opted for surgical intervention.

Thirteen of the 25 observed patients were studied with CE-MRA. Initial mean luminal aneurysm volume was 851 $mm^3$ (range, 155-2231 $mm^3$). Volumetric growth curves demonstrated stable aneurysms in 10 patients and enlargement in 3 patients (Figure 4), who were later selected for surgery.

Sixteen surgical patients had a mean age of 59 years (range, 44-76 years) and a male predominance (12 men and 4 women). Presentations included subarachnoid hemorrhage in 3 patients (19%), brainstem compression in 10 patients (63%), cranial neuropathy in 5 patients (31%), stroke in 5 patients (31%), and hydrocephalus in 7 patients (44%). The mean mRS at presentation was 2.6 (range, 1-5). The mean aneurysm diameter was 2.7 cm (range, 1.5-5.0 cm), including 9 giant aneurysms and 9 thrombotic aneurysms.

## Main Results: Surgical Results, Phase 1

In the first management phase, patients were bypassed with an EC-IC bypass consisting of either STA-SCA (n = 3) or STA-PCA (n = 1) bypass (Table 1 and Figure 5). One patient with an inadequate STA required an ECA-SCA bypass with saphenous vein graft. Bypasses were performed through an orbitozygomatic craniotomy with subtemporal exposure of the recipient artery.

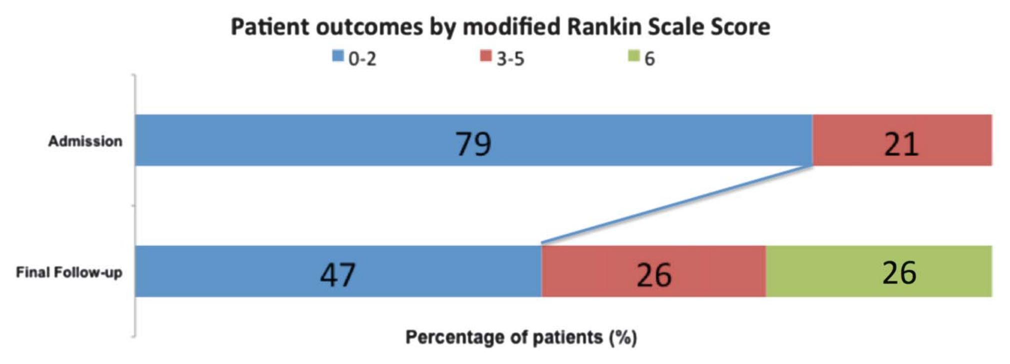


**FIGURE 3.** *Patient outcomes by modified Rankin Scale scores (mRS) in the 25 patients with dolichoectatic basilar trunk aneurysms that were managed conservatively initially. The mRS scores at final follow-up refer to those at the end of the observation period, including the preoperative mRS scores of the 4 patients who later went on to surgery. The postoperative mRS scores in these 4 patients are described later in the surgical results. The mRS scores of the 2 patients lost to follow-up were excluded.*



Copyright © Congress of Neurological Surgeons. Unauthorized reproduction of this article is prohibited.

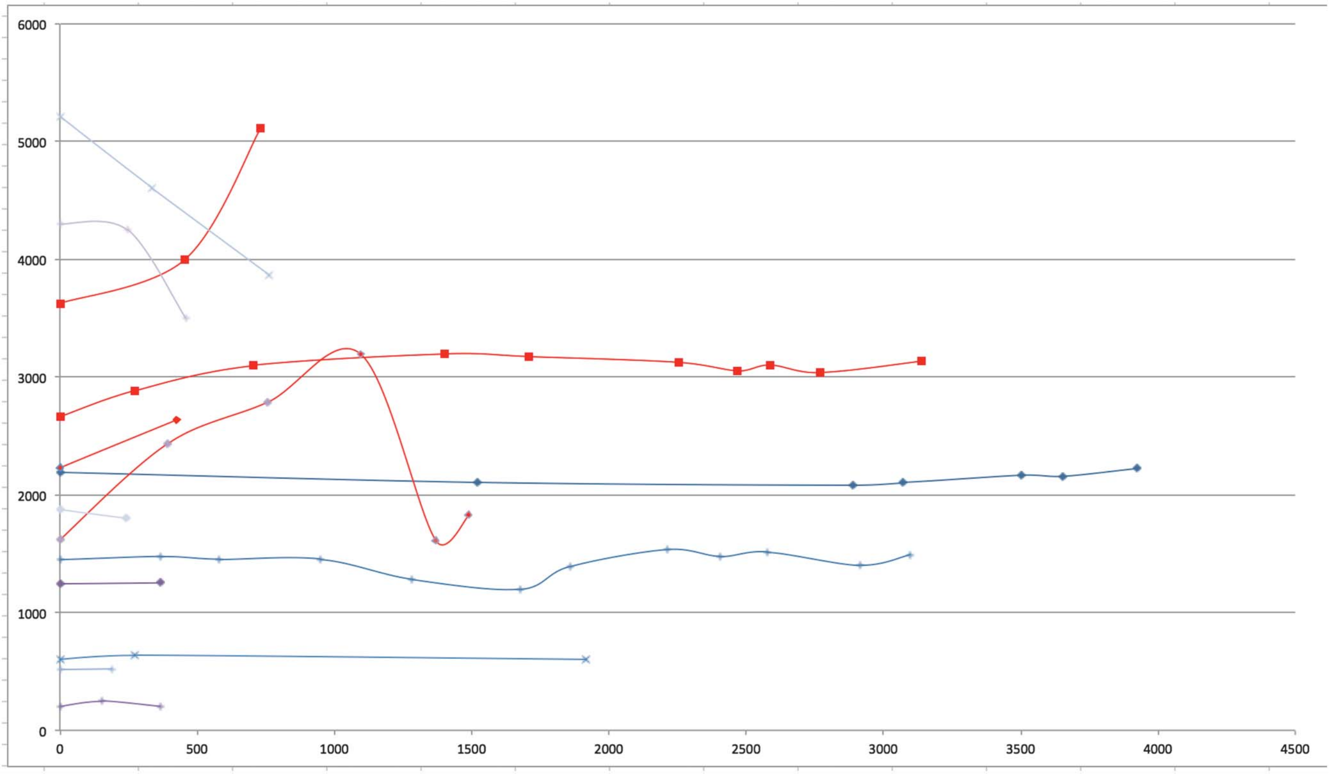


**FIGURE 4.** *Aneurysm growth curves for 13 patients with observed dolichoectatic basilar trunk aneurysms. The x-axis is observation time measured in days, and the y-axis is aneurysm volume measured in cubic mm. Decreases in volume correlated with new intra-aneurysmal thrombosis, with or without clinical stroke. Curves in red highlight patients with aneurysm enlargement, of whom 1 stabilized and 3 went on to surgical intervention.*

Surgery was limited to bypass only, with the intent to endovascularly coil occlude the vertebral arteries bilaterally, one proximal to the posterior inferior cerebellar artery (PICA) origin and the other distal to the PICA origin, in order to eliminate aneurysmal inflow, reverse blood flow in the basilar trunk, and use one PICA to draw or sump flow in this retrograde direction. All bypasses were performed successfully and were patent (Table 2). One patient who presented with subarachnoid hemorrhage had a fatal re-rupture the night after his surgery. The remaining 4 patients were coiled on the third postoperative day and heparinized to facilitate flow reversal. One patient experienced a fatal subarachnoid hemorrhage 2 days after tolerating her endovascular occlusion (Figure 6). Another developed a sizable subdural hematoma requiring evacuation, and although he recovered from deficits related to the SDH, his family later withdrew support. Two patients developed brainstem and cerebellar infarctions after their coiling procedures from basilar artery thrombosis and died.

In summary, although flow reversal with EC-IC bypass and staged endovascular occlusion of proximal inflow has been reported as a treatment option for dolichoectatic basilar trunk aneurysms, this hemodynamic alteration may be too thrombogenic, heparin may be too dangerous in the acute perioperative setting, and the temporal artery may be insufficient to fully revascularize the posterior circulation.

## Surgical Results, Phase 2

In the second management phase, patients were bypassed with an intracranial-to-intracranial bypass consisting of a VA-SCA bypass with an interposition graft (n = 3) (Figure 7). The dominant VA provided a donor site (proximal V3 segment) and was then occluded distally (distal V3 segment or proximal V4 segment) to reduce inflow to the basilar trunk aneurysm. One patient already had an occluded VA and the subclavian artery was used as the donor artery instead. This strategy was intended to maintain anterograde flow in the basilar trunk and reduce it with the combination of unilateral, dominant VA occlusion and increased distal flow to the basilar apex. The modified park bench position is used and the “hockey stick” incision of the far lateral approach is extended anterosuperiorly in front of the ear to incorporate the subtemporal exposure.

The first patient had a reduction in aneurysm luminal volume after the procedure with no neurological complications (Figure 8), but her aneurysm continued to grow with symptom progression. One year



Copyright © Congress of Neurological Surgeons. Unauthorized reproduction of this article is prohibited.

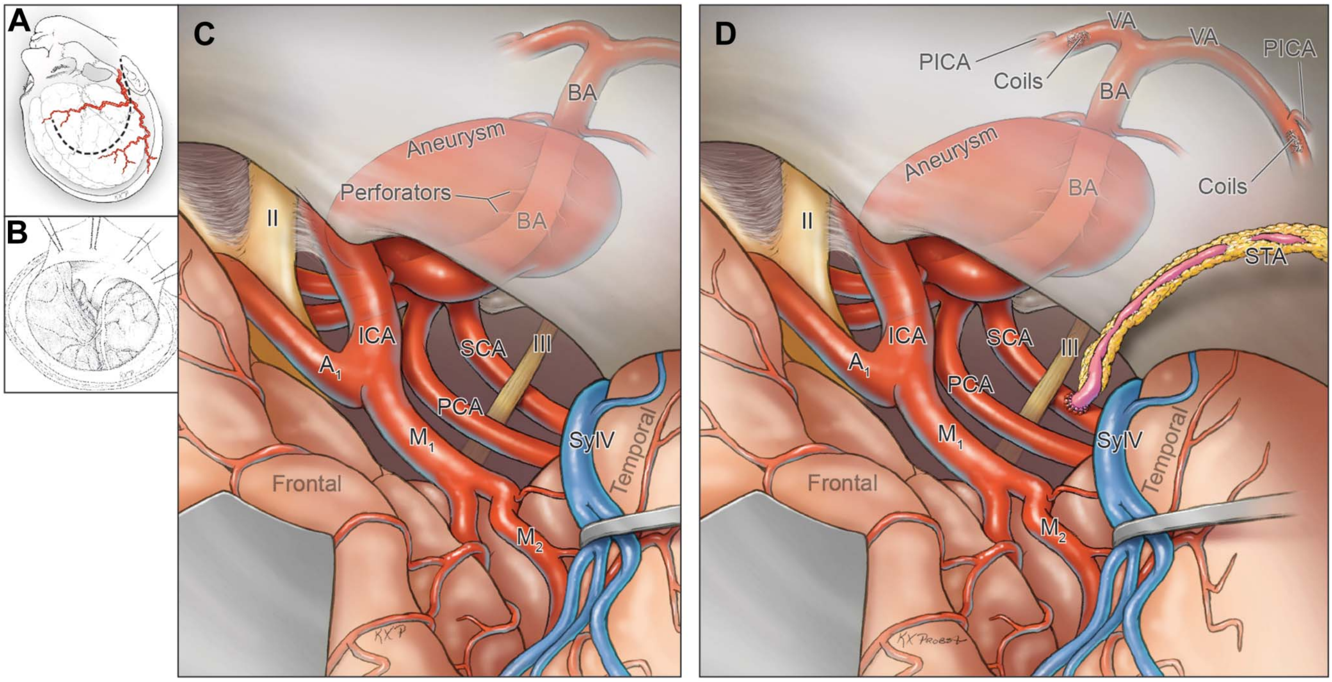


**FIGURE 5.** *Summary of phase 1 surgical management (STA to SCA or PCA bypass), with (***A***) harvest of donor STA, (***B***) exposure through an orbitozygomatic-pterional craniotomy, (***C***) exposure and access to recipient SCA or PCA subtemporally or pretemporally, (***D***) completion of the extracranial-to-intracranial bypass anastomosis (STA to SCA bypass shown), and endovascular occlusion of the vertebral arteries bilaterally in a separate procedural stage. II, optic nerve; III, oculomotor nerve; A1, anterior cerebral artery, A1 segment; BA, basilar artery; ICA, internal carotid artery; M1, middle cerebral artery, M1 segment; PCA, posterior cerebral artery; PICA, posterior inferior cerebellar artery; SCA, superior cerebellar artery; STA, superficial temporal artery; SylV, Sylvian vein; VA, vertebral artery.*

postoperatively, follow-up angiography showed the bypass filling only the recipient SCA territory distally. Her contralateral VA was sacrificed endovascularly, resulting in a fatal basilar artery thrombosis. The bypass in the second patient occluded postoperatively due to technical complications: an inability to access his radial artery in the park bench position; the use of an allograft saphenous vein graft instead; and a small, duplicated SCA used as the recipient artery. His bypass occlusion prevented any additional treatment and his aneurysm grew. He died from complications related to an aspiration pneumonia 1 year postoperatively, and experienced progressive deficits during that year. The third patient presented with a ruptured aneurysm involving the upper basilar trunk that was clip reconstructed after performing her bypass—the only clip reconstruction in the series. She recovered completely and is free of aneurysm recurrence 8 years after surgery. None of these patients was treated with heparin.

In summary, flow reduction seemed to be safer than flow reversal; except for the clip reconstructed aneurysm, the other

**TABLE 2.** **Summary of Results in Phases of Management of Dolichoectatic Basilar Trunk Aneurysms**[a]

| | Phase 1, N (%) | Phase 2, N (%) | Phase 3, N (%) | Total, N (%) |
|---|---|---|---|---|
| Treatment | Bypass/flow reversal | Bypass/flow reduction | Bypass/distal occlusion | |
| Patients | 5 | 3 | 8 | 16 |
| Bypass patency | 5 (100) | 2 (67) | 8 (100) | 15 (94) |
| Surgical Mortality | 4 (80) | 0 (0) | 4 (50) | 8 (50) |
| Final mortality | 5 (100) | 2 (67) | 5 (62) | 12 (75) |
| Survivors | 0 (0) | 1 (33) | 3 (38) | 4 (25) |
| Final mean mRS | 6 | 1.0 | 3.3 | 2.8 |
| F/U durations (yr) | NA | 8 | 4.5 | 5.4 |
| Aneurysm stabilization | Unknown | No | Yes | |

[a]F/U, follow-up; mRS, modified Rankin Scale score; NA, not applicable.



Copyright © Congress of Neurological Surgeons. Unauthorized reproduction of this article is prohibited.

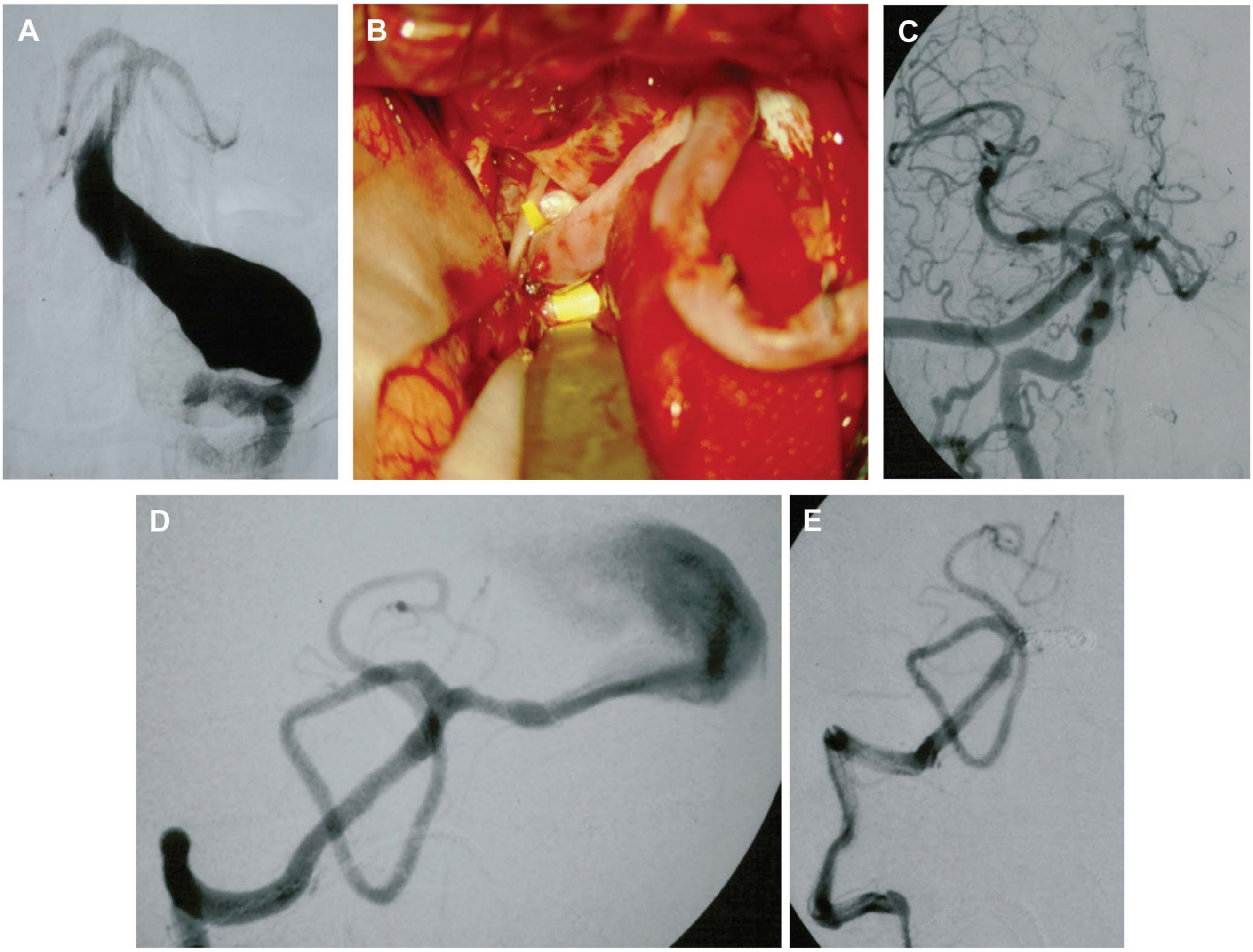


**FIGURE 6.** *Case example of phase 1 surgical management.* **A**, *this 60-year-old woman had progressive brainstem compressive symptoms from this dolichoectatic basilar trunk aneurysm (left vertebral arteries digital subtraction angiogram, anteroposterior view).* **B**, *a bypass from the right external carotid artery to the superior cerebellar artery with saphenous vein graft was performed, (***C***) revascularizing the basilar apex (right common carotid angiogram, anteroposterior view) and (***D*** and* **E***) enabling the vertebral arteries to be occluded endovascularly 3 days later while heparinized (right vertebral arteries angiogram, anteroposterior view, [***D***] before and [***E***] after coil occlusion distal to posterior inferior cerebellar artery). The patient tolerated her flow reversal for 2 days, and then experienced a fatal subarachnoid hemorrhage while on heparin.*

aneurysms still enlarged progressively and low demand on the bypass reduced its impact on distal basilar flow. The far lateral craniotomy accessed the posterior fossa for decompressive craniectomy and enabled simultaneous surgical occlusion of the dominant VA, which was not possible in phase 1 with the orbitozygomatic approach. The distal anastomosis to the SCA and the harvest of the bypass graft can be awkward in the park bench position, making this strategy imperfect.

### Surgical Results, Phase 3

In the third management phase, patients were bypassed with an intracranial-to-intracranial bypass consisting of an MCA-PCA bypass in all patients (n = 8), with radial artery used as the graft in 6 patients and saphenous vein in 2 patients. All bypasses were performed through an orbitozygomatic-transsylvian exposure of the recipient P2 segment of PCA (Figure 9). The deep anastomosis was performed first and the M2 MCA segment was used as the donor site for the second anastomosis. All bypasses were confirmed to be patent, after which aneurysm outflow was occluded distally with clips on the basilar trunk. Interestingly, indocyanine green demonstrated no bypass flow in 3 patients until demand was created by clip occlusion of the basilar artery. This strategy of MCA-PCA bypass and distal aneurysm occlusion maintained anterograde flow in the proximal basilar artery and aneurysm, albeit reduced; it split the posterior circulation into upper and lower halves, with the basilar



Copyright © Congress of Neurological Surgeons. Unauthorized reproduction of this article is prohibited.

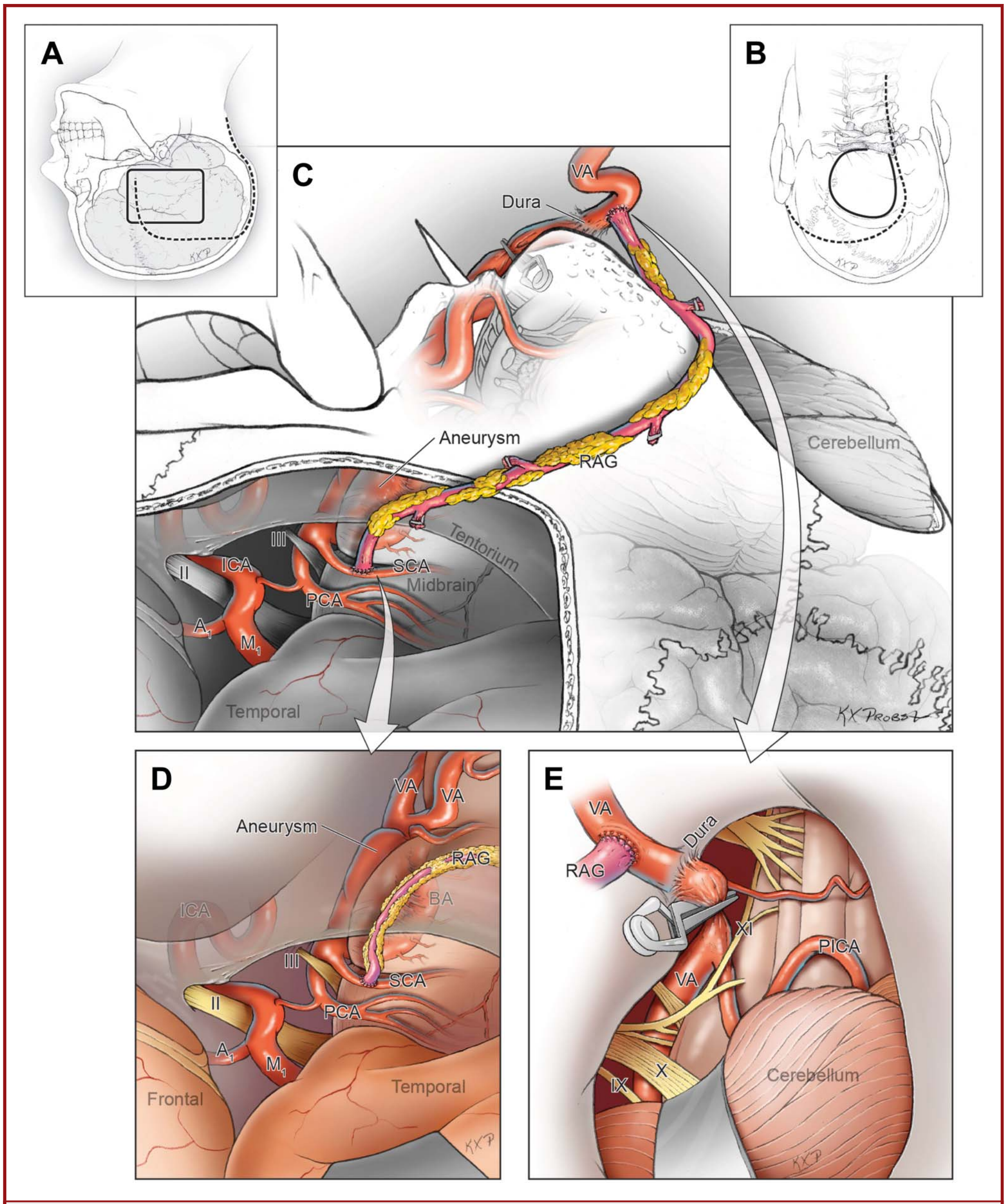


**FIGURE 7.** *Summary of phase 2 surgical management (VA to SCA bypass), with combined (**A**) temporal and (**B**) far lateral craniotomies through a single incision, (**C**) exposure of the donor V3 segment of VA and recipient SCA, (**D**) completion of the graft to SCA anastomosis, and (**E**) graft to VA anastomosis with clip occlusion of the dominant VA either extradurally (V3) or intradurally (V4). Suboccipital craniectomy and duraplasty creates space to accommodate cerebellar swelling. II, optic nerve; III, oculomotor nerve; IX, glossopharyngeal nerve; X, vagus nerve; XI, accessory nerve; A1, anterior cerebral artery, A1 segment; BA, basilar artery; ICA, internal carotid artery; M1, middle cerebral artery, M1 segment; PCA, posterior cerebral artery; PICA, posterior inferior cerebellar artery; RAG, radial artery graft; SCA, superior cerebellar artery; VA, vertebral artery.*

quadrifurcation supplied by a more robust bypass than the temporal artery bypasses; and it required a standard approach rather than an awkward combination approach.

Despite good technical execution in all of the first 4 patients, all experienced brainstem perforator infarcts postoperatively that were minor in 2 patients (Figure 10), resulting in a drop in mRS scores from 2 to 3 in both, and fatal in 2 patients. These results indicated that the MCA-PCA bypass adequately revascularized the distal basilar circulation, but the anterograde basilar flow was still not sufficient to preserve perforator patency.



Copyright © Congress of Neurological Surgeons. Unauthorized reproduction of this article is prohibited.

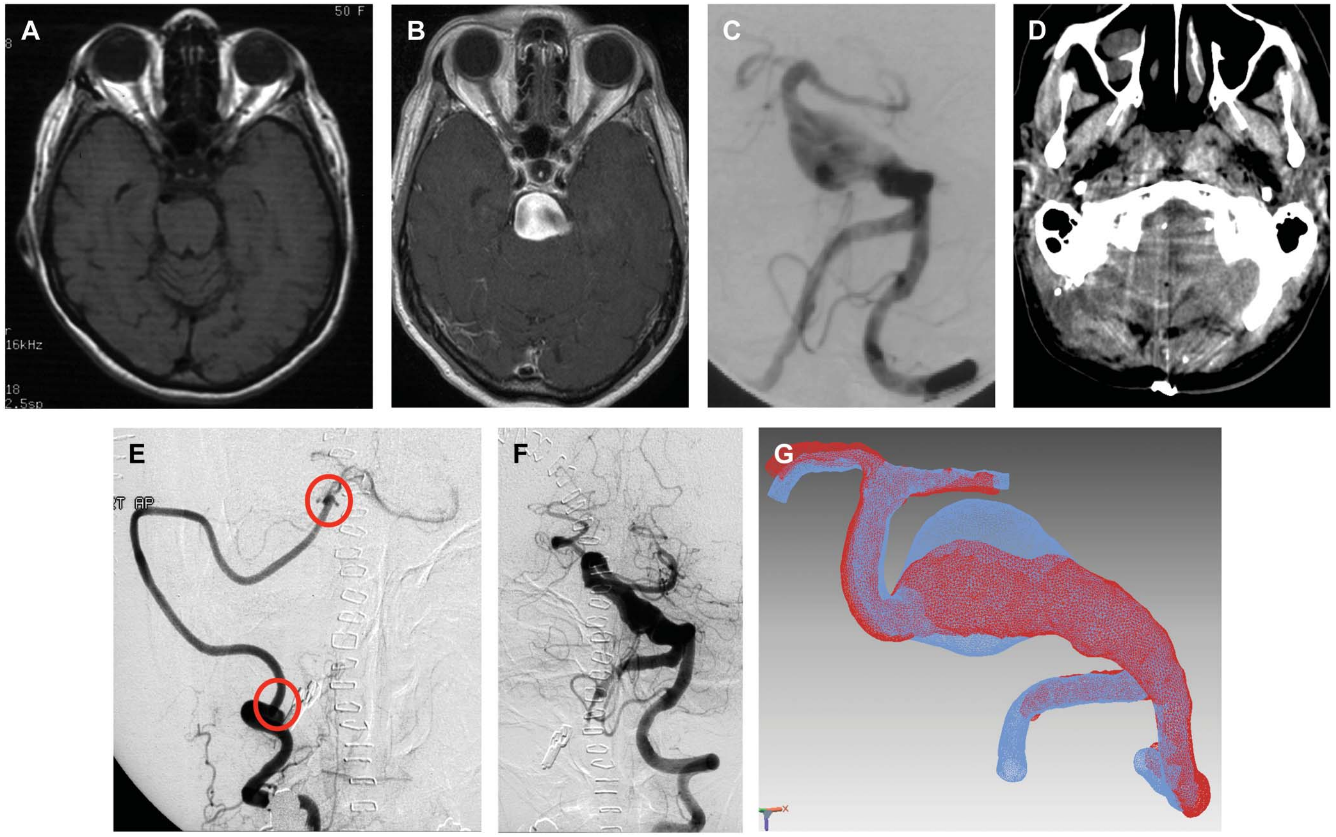


**FIGURE 8.** *Case example of phase 2 surgical management.* **A**, *this 51-year-old woman initially presented with mild symptoms from mild basilar dilatation and tortuosity (axial magnetic resonance imaging, T1-weighted image), and was observed for 3 years.* **B**, *during those years, her aneurysm grew and her symptoms became severe (axial magnetic resonance imaging, T1-weighted image with gadolinium; and [***C***] left vertebral artery digital subtraction angiogram, anteroposterior view).* **D**, *the combined subtemporal-far lateral approach enabled a wide suboccipital craniectomy and duraplasty for posterior fossa relaxation, as seen on axial postoperative computed tomographic scan.* **E**, *the postoperative angiogram (right vertebral arteries angiogram, anteroposterior view) demonstrated patency of the right V3 vertebral artery-superior cerebellar artery bypass, occlusion of the distal right vertebral artery, and filling of the basilar quadrification.* **F**, *the left vertebral artery, angiogram (anteroposterior view) demonstrated significant thrombosis of aneurysm lumen, (***G***) shown with superimposed luminal aneurysm volumes pre- (blue) and postoperatively (red) generated with high-resolution contrast-enhanced magnetic resonance angiography techniques. Although endovascular occlusion of the left vertebral arteries was intended postoperatively, it was deferred due to her excellent clinical and angiographic outcomes. Her symptoms progressed and the aneurysm enlarged 6 months later, prompting left vertebral artery, coiling that caused a fatal thrombosis of the basilar trunk.*

Therefore, clopidogrel (Plavix; Bristol-Myers Squibb, New York, New York) was added to the postoperative management regimen, with 75 mg administered immediately postoperatively and another 75 mg administered after confirming no hemorrhaging complications on CT scan. Clopidogrel (Plavix) was then maintained at 75 mg once daily.

With this clopidogrel (Plavix) regimen in the next 4 patients, 1 patient had no perforator infarcts and tolerated his surgery without complication; 1 patient experienced a minor brainstem perforator infarct resulting in a decrease in mRS score from 2 to 3; and 1 patient had fatal brainstem perforator infarcts. The final patient experienced an SCA territory infarction resulting from an attempt to clip occlude the distal basilar artery with the clip blades below one SCA to include it in the territory of the bypass and above the other to include it in the territory of the basilar artery. This "SCA cross-clipping" technique was intended to sump flow through the aneurysm to the SCA and thereby increase basilar artery blood flow to maintain perforator patency (Figure 11). Indocyanine green videoangiography demonstrated SCA patency intraoperatively, but it occluded later and the patient died.

In summary, MCA-PCA bypass created a "surgical posterior communicating artery" that robustly supplied the basilar quadrifurcation, but only after clip occlusion of the basilar artery. This bypass did not significantly diminish transaneurysmal flow by itself, based on indocyanine green videography. Distal clip occlusion of the basilar trunk aneurysm preserved anterograde flow in the aneurysm and did not precipitate its rupture, but the resulting flow reduction threatened perforator patency.



Copyright © Congress of Neurological Surgeons. Unauthorized reproduction of this article is prohibited.

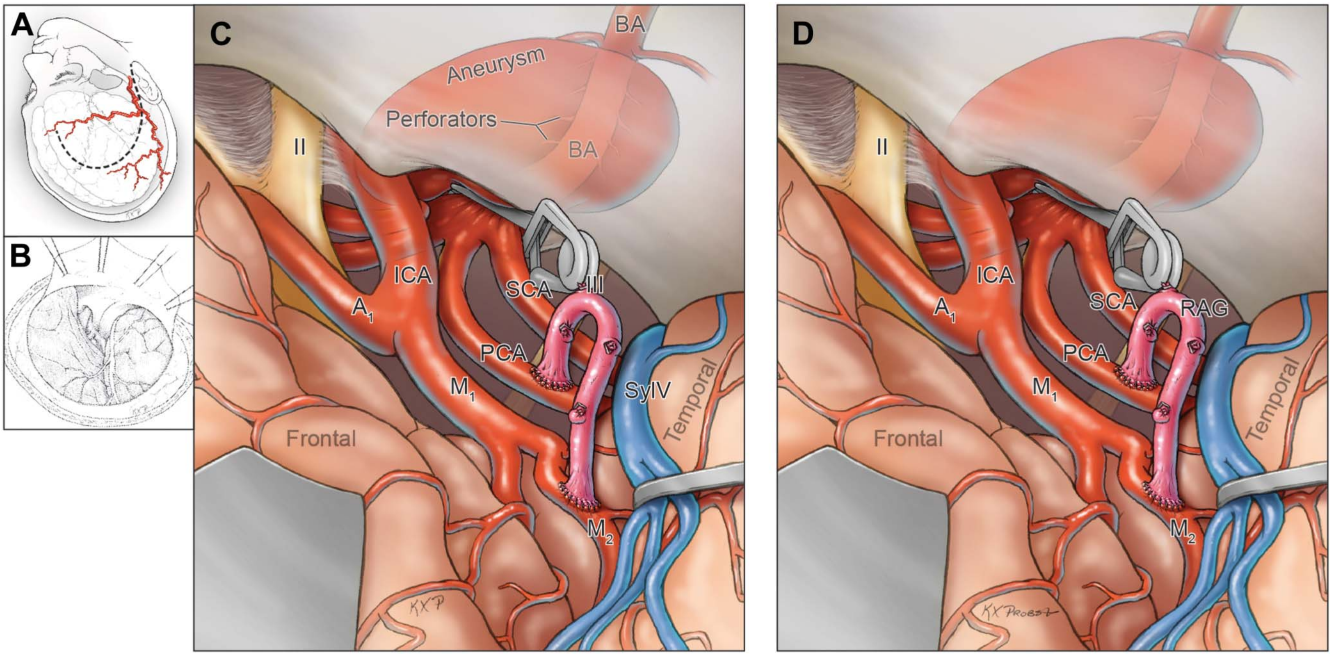


**FIGURE 9.** *Summary of phase 3 surgical management (middle cerebral artery to posterior cerebral artery bypass), with (***A** *and* **B***) exposure through an orbitozygomatic-pterional craniotomy, (***C***) exposure via transsylvian access to the recipient posterior cerebral artery pretemporally, (***D***) completion of 2 anastomoses to the posterior cerebral artery and middle cerebral artery, and distal clip occlusion of the aneurysm below the basilar quadrifurcation. II, optic nerve; III, oculomotor nerve; A1, anterior cerebral artery, A1 segment; BA, basilar artery; ICA, internal carotid artery; M1, middle cerebral artery, M1 segment; PICA, posterior inferior cerebellar artery; RAG, radial artery graft; SCA, superior cerebellar artery; SylV, Sylvian vein; VA, vertebral artery.*

Clopidogrel (Plavix) was tolerated postoperatively and an increased loading dose may be indicated. Surviving patients tended to have smaller aneurysms with smaller luminal volumes, suggesting that earlier intervention may impact outcome favorably. Surviving patients also tended to have aneurysms located at the upper basilar trunk (above AICA and the midpoint of clivus), suggesting that perforator complications from aneurysm thrombosis involving the midbrain are less devastating than those involving the pons. Cross-clipping the SCA was technically difficult to accomplish. The shifting treatment strategy for dolichoectatic basilar trunk aneurysms (Table 1) resulted in small improvements in surgical and final mortalities (Table 2), with stabilization of aneurysms in the phase 3 survivors.

## DISCUSSION

This report describes a large experience managing patients with dolichoectatic basilar trunk aneurysms—one of, if not the most, difficult aneurysms encountered in practice. Although we focused on the evaluation of bypass as part of an evolving treatment paradigm, our conservative management posture should be emphasized. Less than a third of all patients (12 of 37) were initially selected for treatment, favoring a period of clinical and radiographic surveillance instead. The natural history of our observed patients was more benign than expected, with only 44% experiencing neurological deterioration and 20% dying, not always from their aneurysms. Furthermore, only a quarter of patients followed with CE-MRA demonstrated significant enlargement. This natural history in our series was more favorable than predicted by other reports on giant aneurysms and dolichoectatic basilar trunk aneurysms.[3,13,14]

Reports in the literature suggest that the treatment of these aneurysms with EC-IC bypass surgery and aneurysm occlusion can be performed with high but perhaps acceptable morbidity (approximately 20%) and mortality (approximately 18%), as well as with reasonable efficacy, as determined by low rates of subsequent aneurysm growth.[16,17,19-32,34-38] Phase 1 of our experience can be viewed as a validation study, and our findings were not confirmatory. Our poor results cannot be attributed to technical complications with bypass (100% patency), leading us to conclude that reversal of flow in basilar trunk aneurysms is dangerously thrombogenic (Figure 12); the use of heparin postoperatively increases morbidity; and this strategy should be discouraged. Phase 2 demonstrated that flow reduction rather than reversal prevents catastrophic basilar thrombosis but fails to stabilize these aneurysms. Phase 2 also showed that a bypass to the posterior circulation distal to the aneurysm without the high demand created by an occlusive intervention fails to alter basilar



Copyright © Congress of Neurological Surgeons. Unauthorized reproduction of this article is prohibited.

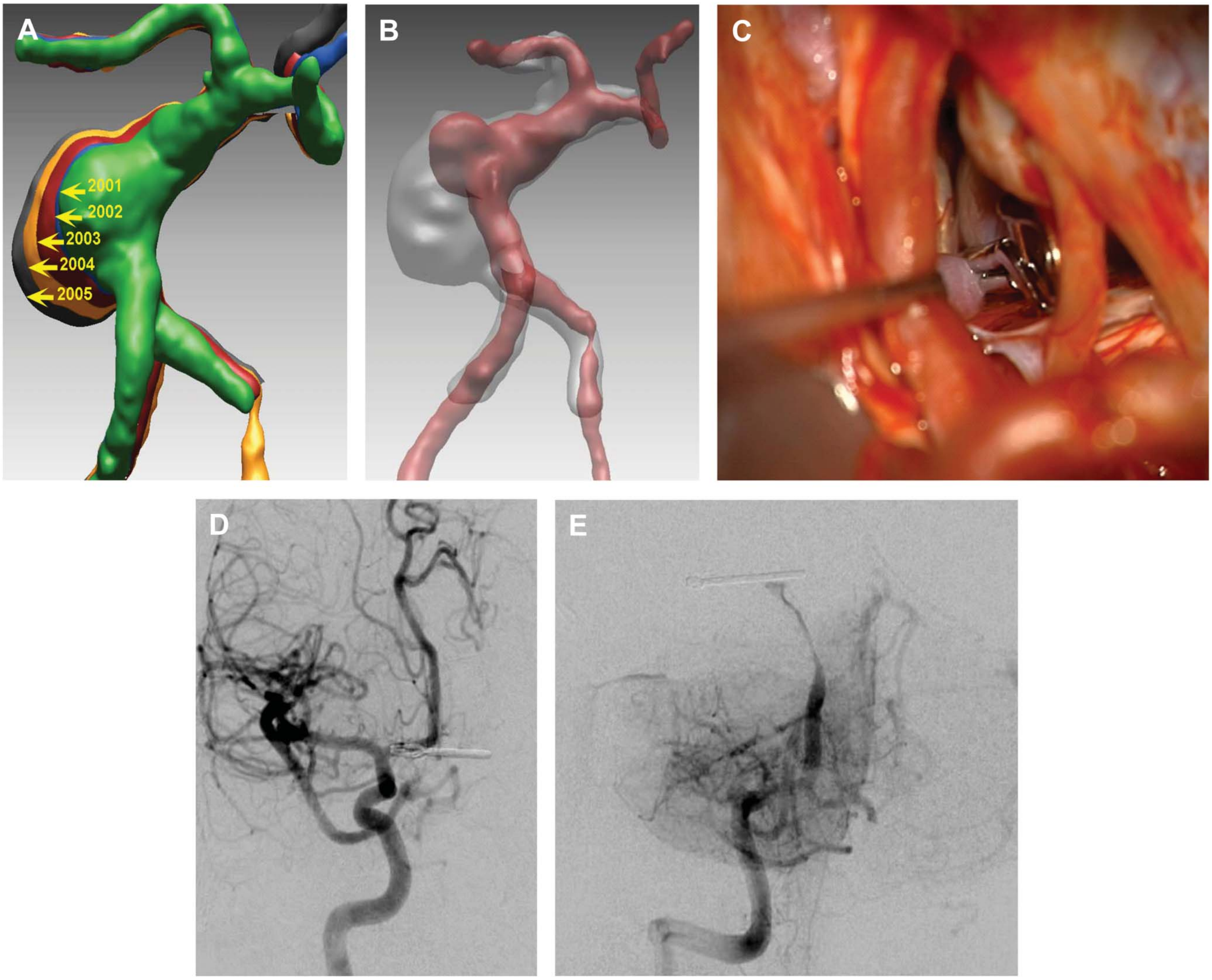


**FIGURE 10.** *Case example of phase 3 surgical management.* **A**, *the 44-year-old man was followed with high-resolution contrast-enhanced magnetic resonance angiography for 4 years with progressive aneurysm enlargement.* **B**, *he then had a pontine stroke from acute thrombosis of the inferior portion of the aneurysm lumen, as shown on superimposed pre- (gray) and post-stroke luminal aneurysm volumes.* **C**, *he underwent middle cerebral artery-posterior cerebral artery bypass and distal clip occlusion of the aneurysm.* **D**, *postoperative angiography showed good perfusion of the basilar quadrifurcation through the bypass (right internal carotid artery angiogram, anteroposterior view) and (***E***) thrombosis of the remaining aneurysm lumen with anterograde flow up to the clip (right vertebral artery angiogram, anteroposterior view). The patient experienced a minor pontine perforator infarct resulting in a decrease in modified Rankin Scale score from 2 to 3, but he has required no further treatment in the subsequent 7 years and lives with some minor assistance.*

hemodynamics and can lead to involution of the bypass. In phase 3, we expected the anterograde flow maintained by distal aneurysm occlusion to preserve blood flow in basilar perforators and were disappointed to find that patients still experienced postoperative brainstem infarcts (Figure 12). Overall, the results demonstrated that not just the right bypass determines good outcomes, but also perforator preservation and inhibition of aneurysm thrombosis after the flow-reducing intervention. The MCA-PCA bypass, with its intracranial location, robust flow, short length, and easier execution than other bypasses, is ideal and all were flawless in this series. However, the interplay between flow reduction and aneurysm thrombosis creates a dangerous therapeutic situation ranging from ineffective to catastrophic. The addition of antiplatelet agents seems to be an important addition to the management regimen and was tolerated better than expected, but perforator occlusion persisted and calls for further innovation, perhaps with a more aggressive loading dose.



Copyright © Congress of Neurological Surgeons. Unauthorized reproduction of this article is prohibited.

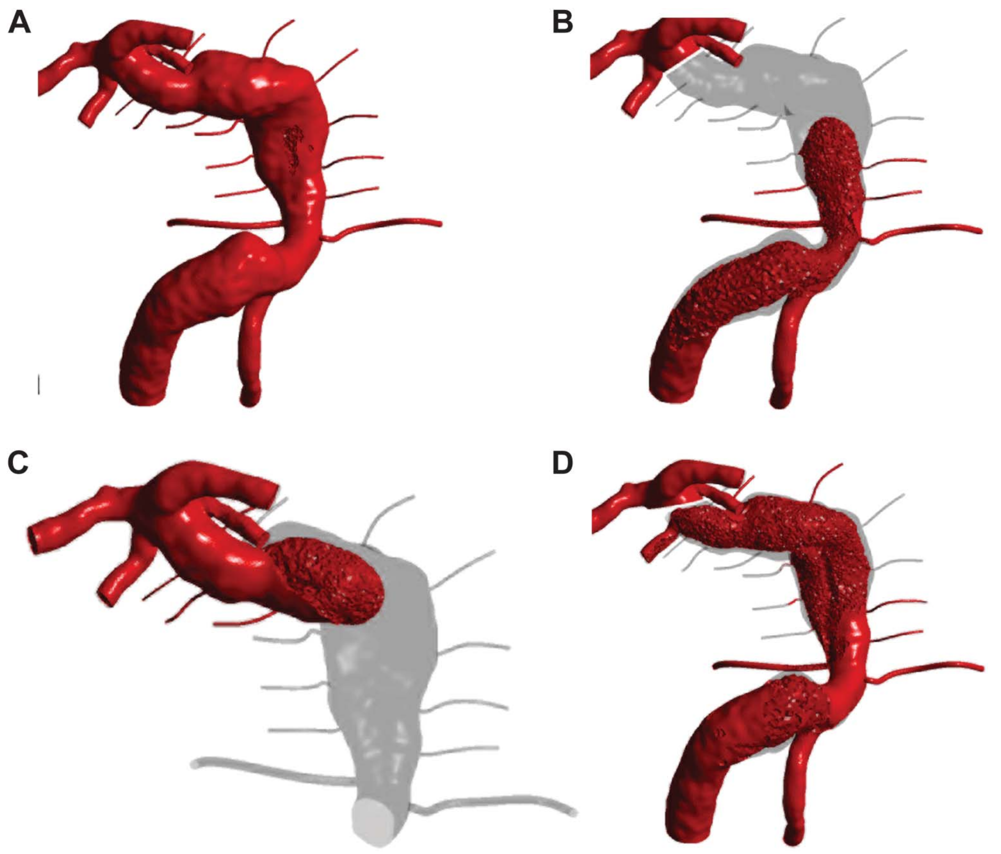


**FIGURE 11.** *Computational fluid dynamic simulations of treatment options for a dolichoectatic basilar trunk aneurysm.* **A**, *virtual contrast transport is shown in red, demonstrating the branch and perforator anatomy of this aneurysm preoperatively. After alternative treatments, contrast transport is shown in red 8 cycles after the start of virtual injection, whereas increased flow residence time is shown in gray.* **B**, *right middle cerebral artery-posterior cerebral artery bypass with distal aneurysm occlusion below the quadrifurcation, with a clip proximal to superior cerebellar arteries (option 1).* **C**, *right middle cerebral artery-posterior cerebral artery bypass with proximal aneurysm occlusion, with a clip on the basilar artery proximal to the anterior inferior cerebellar arteries (option 2).* **D**, *right middle cerebral artery-posterior cerebral artery bypass with superior cerebellar artery cross-clipping, with a clip on the distal basilar artery above one superior cerebellar artery to sump flow through the basilar artery and below the other superior cerebellar artery that is supplied by the bypass (option 3). Computational fluid dynamics simulations and computed velocities demonstrated slowed anterograde flow through the basilar trunk with distal occlusion and slowed retrograde flow through the basilar trunk with proximal occlusion, both with stagnation near the clip site, suggesting high risk of perforator occlusion. In contrast, superior cerebellar artery cross-clipping created an outlet for anterograde flow (run-off) that increased basilar trunk flow relative to option 1 and appeared more likely to maintain perfusion to pontine perforators.*

## The Next Phase

The reduction in surgical mortality from 80% in phase 1 to 50% in phase 3 represents some real progress, but our surgical efforts demonstrate that flow direction may not be the determinative factor. Flow above some threshold level, regardless of its direction, is needed to stave off complete thrombosis of the basilar lumen and occlusion of the perforators. Clip occlusion of the basilar artery, whether distal or proximal, eliminates basilar flow except what its branches can pull or sump either from the proximal basilar artery in the case of distal aneurysm occlusion or from the bypass in the case of proximal aneurysm occlusion. Small perforating branches are unable to sump enough flow for their preservation and are overwhelmed by stagnation of blood within a vast intra-aneurysmal space, leading to thrombosis. We have studied this process in the natural course of untreated giant aneurysms, observing spontaneous thrombosis in regions of low flow velocity, low wall shear stress, and long residence times. Extrapolating, therapeutic reduction of flow in the dolichoectatic basilar trunk aneurysm represents an extreme provocation of thrombosis that even the aggressive use of anti-platelets or anticoagulants may not be able to overcome.

One solution may be the creation of a distal run-off, or occluding the basilar artery but maintaining a major branch artery



Copyright © Congress of Neurological Surgeons. Unauthorized reproduction of this article is prohibited.

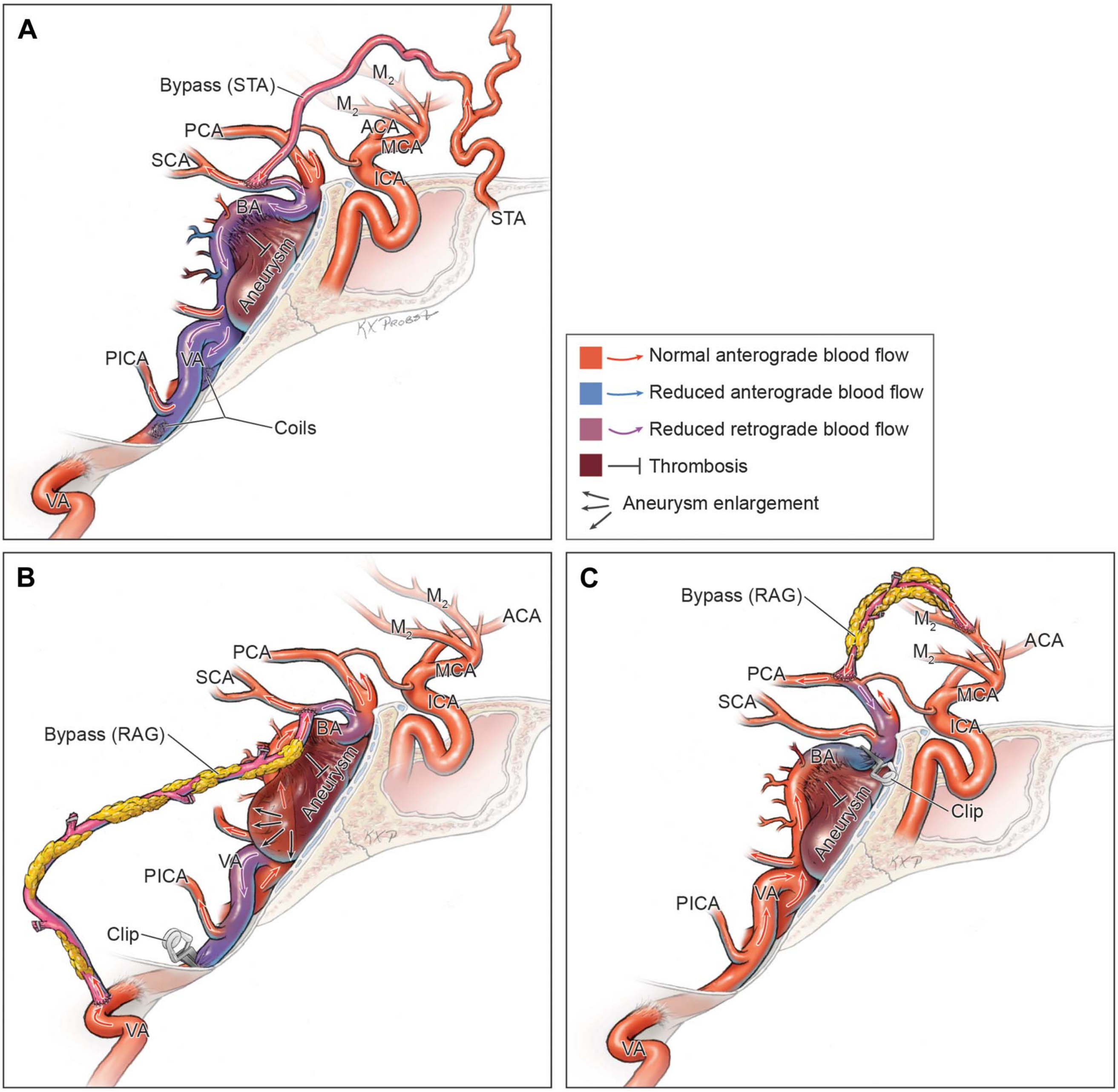


**FIGURE 12.** *Outcomes after bypass and flow alteration.* **A**, *STA-SCA bypass and flow reversal with bilateral vertebral artery coil occlusion resulted in aneurysm thrombosis and perforator occlusion (phase 1).* **B**, *VA-SCA bypass and flow reduction with unilateral vertebral artery clip occlusion preserved anterograde flow and precipitated some aneurysmal thrombosis but failed to stabilize the aneurysm (phase 2).* **C**, *MCA-PCA bypass and distal aneurysm clip occlusion also preserved anterograde flow but threatened distal basilar perforators in some patients (phase 3). BA, basilar artery; ICA, internal carotid artery; M1, middle cerebral artery, M1 segment; PICA, posterior inferior cerebellar artery; RAG, radial artery graft; SCA, superior cerebellar artery; STA, superficial temporal artery; VA, vertebral artery.*

that can sump flow through the aneurysm and nourish the perforators. Cross-coiling the PICAs in phase 1 is conceptually similar to distal run-off because one PICA sumps flow from the bypass. The SCA cross-clip is an example of this solution, but it is difficult to do in practice, as demonstrated by our last case in which the cross-clip occluded one of the SCA branches. An alternative might be cross-clipping the AICAs through a combined approach that exposes the basilar apex for the MCA-PCA bypass as well as



Copyright © Congress of Neurological Surgeons. Unauthorized reproduction of this article is prohibited.

the basilar trunk for proximal clip occlusion. Preliminary work in cadavers has demonstrated that an orbitozygomatic-extended retrosigmoid approach offers this exposure, but computational fluid dynamic simulations suggest that AICA has less sump effect than SCA due to its smaller caliber and the fact that flow is proportional to an artery's radius to the fourth power.

The pathology and hemodynamics of dolichoectatic basilar trunk aneurysms leave little margin for error in their surgical treatment. The confluence of 2 arteries into 1 and the dolichoectatic morphology of the aneurysm generate complex flows with high-velocity jets, variable shear stresses, stagnation, inflammation, and thrombosis. In this state, perforators frequently occlude spontaneously and patent perforators may already be struggling to survive. In this context, it is remarkable that any intervention upsetting the delicate balance between flow and thrombosis would work, but results in our survivors and in others' reports are dramatic.[16,26] The presence of intra-aneurysmal thrombus preoperatively is a feature shared by survivors, indicating that propagating thrombosis therapeutically may be safer than initiating it in an aneurysm that had none previously. Preexisting thrombus may be a nidus for additional thrombus that avoids branch origins, or it may have already occluded perforators that would have caused surgical morbidity. Aneurysms with lower initial luminal volumes tended to fare better as well, perhaps because the dead space within the aneurysm where flow stagnates after occlusion is smaller, reducing the load of new thrombus. These observations are anecdotal and, despite our efforts to quantitate the morphometrics and hemodynamics of these aneurysms, there are no measures to guide management. Even with sophisticated computational fluid dynamic, threshold levels for flow velocity, shear stress, and residence time are unknown, as are their effects on thrombus propagation.

### Endovascular Options

Stents or flow diverters were expected to solve the dolichoectatic basilar trunk aneurysm. Conceptually, an intra-aneurysmal conduit would eliminate malignant hemodynamics, preserve flow in branch arteries, and promote thrombosis outside the lumen, much like stent grafts used with aortic aneurysms. However, patients treated with the flow diverters have had complications related to perforator occlusion, just like surgical patients (Table 3).[3,6-15] Flow diverters did not actually divert flow unless multiple devices were deployed, resulting in occlusive coverage of the perforators and branch arteries originating from the trunk.[14] Flow diverters were initially too short to treat the entire lesion and necessitated overlapping devices.[14] In addition to perforator complications, the Pipeline device caused peri-procedural ruptures, even in aneurysms that had never ruptured before,[14] thought to be due to a "ball-valve" mechanism where blood forcibly escaped through the pores of the flow diverter during systole and was trapped extraluminally where it slowly expanded the aneurysm. This complication resulted in devastating ruptures (while patients were anti-coagulated or taking anti-platelet agents, or both).

With the Pipeline device now offered in longer sizes, some have begun treating dolichoectatic basilar trunk aneurysms with a single device to minimize perforator coverage and with intra-aneurysmal coiling around the flow diverter to obliterate the surrounding space.[39] Results with these technique modifications have not been reported. Endovascular technology is ever-evolving and new designs might offer a solution. We are intrigued by the possibility of parent artery occlusive devices that can be deployed endovascularly—an objective not typically pursued by industry but applicable in this setting. Occlusive devices might offer stand-alone therapies that reduce or redistribute flow to modify risk of hemorrhage or aneurysm growth, or they might be used in combination with a bypass procedure to obviate the need for clip occlusion. We are intrigued by the possibility of remodeling the basilar trunk with a flow diverter, allowing it time to endothelialize, and then debulking the aneurysm later to relieve mass effect symptoms. Many of these lesions present with brainstem compressive symptoms, and endovascular devices might enable safe decompressive thrombectomy, which is not currently part of existing interventions.

### Personal Perspective and Limitations

Although it is humbling to report a surgical series where half the patient died, we submit this work to stimulate discourse about the problem of the dolichoectatic basilar trunk aneurysm. These are rare cases, accounting for a fraction of 1% of a large microsurgical experience with nearly 4000 aneurysms, but they are painful and disturbing. Patients confront an incurable disease with a dismal prognosis, often with severe disabilities and high hopes for treatment. Neurosurgeons invest tremendous, and sometimes flawless, effort in the procedure, only to have an abrupt fatality that is difficult to explain, hard for families to accept, and visible to colleagues in the hospital. These defeats call into question the advisability and ethics of further efforts. While many clinicians have already given up on this disease and abandoned surgical options, our failures, perhaps more than our successes, have forced us to study this pathology in autopsy specimens and in the cadaver lab, stimulating insights, technical developments, and improvements. In our opinion, the treatment of dolichoectatic basilar trunk aneurysms will advance only through concentrated management in select few centers and concerted effort between neurosurgeons, neurologists, and interventional neuroradiologists to study the morphology and hemodynamics with sophisticated computational methods. Multicenter registry data must be collected on these patients. In the vascular neurosurgical subspecialty, this disease is akin to glioblastoma multiforme in the tumor neurosurgical subspecialty, in that the underlying biology is complex, cures are elusive, and the fight will involve unconventional teams of scientists. Therapeutic advancement with the dolichoectatic basilar aneurysm is hampered not only by its rarity, but also by a reluctance to intervene.

## CONCLUSION

In his first report on "Bleeding Aneurysms of the Basilar Artery" in 1960 in which he described results in 4 patients (3 basilar



Copyright © Congress of Neurological Surgeons. Unauthorized reproduction of this article is prohibited.

**TABLE 3. Published Results for the Endovascular Treatment of Dolichoectatic Basilar Trunk Aneurysms[a]**

| First Author | Last Author | Year, Journal | Total No. of Fusiform BA/VBJ Aneurysms | No. of Basilar Trunk Aneurysms | Vertebrobasilar Junction Aneurysms | Devices Used |
|---|---|---|---|---|---|---|
| Byrne J | Kamran M | 2010, *PLos One*[6] | 10 | 3 | 7 | SILK (Balt Extrusion, Montmorency, France) |
| Lubicz B | Leclerc X | 2010, *Stroke*[9] | 3 | 3 | 0 | SILK |
| Raphaeli G | Lubicz B | 2011, *Neurosurgery*[13] | 10 (16 purely vertebral artery aneurysms not included) | 8 | 2 | SILK, ENTERPRISE (Codman Neurovascular/Cordis Corp., Fremont, CA), LEO (Balt Extrusion), SOLITAIRE (Covidien/ev3 Neurovascular, Irvine, CA), COILS |
| Ertl L | Fesl G | 2014, *AJNR Am J Neuroradiol*[7] | 6 | 2 | 4 | PIPELINE, SILK, LEO |
| Monteith S | Jabbour P | 2014, *J Neurosurg*[11] | 7 | 4 | 3 | PIPELINE |
| Meckel S | Klisch J | 2013, *Neurosurgery*[10] | 6 | 0 | 6 | PIPELINE (n = 9), SILK (n = 1) |
| Siddiqui A | Levy E | 2012, *J Neurosurg*[14] | 7 | 7 | 0 | SILK (n = 1), PIPELINE (n = 6) |
| Van Oel L | Peluso J | 2013, *AJNR Am J Neuroradiol*[3] | 13 | NA | NA | Mix: Neuroform, Enterprise, LEO, SILK, and coiling |
| Fischer S | Henkes H | 2014, *Neurosurgery*[8] | 37 | 17 | 20 | PIPELINE |
| Toth G | Hui F | 2014, *J Neurointerv Surg*[15] | 2 | 2 | 0 | PIPELINE |
| Munich S | Lopes D | 2014, *J Neurosurg*[12] | 12 | NA | NA | PIPELINE |
| | | | | | | |
| | | | | | **Total BA trunk aneurysms 46** | |

| First Author | Deaths | Ischemic Complications | Hemorrhagic or Mass Effect Complications | Complications not Otherwise Specified | Angiographic Cures |
|---|---|---|---|---|---|
| Byrne J | 2 | 0 | 1 (mass effect from aneurysm thrombosis—led to death) | | NA |
| Lubicz B | 0 | 1 (branch occlusion) | 0 | | 1 |
| Raphaeli G | 3 | NA | NA | 1 treatment failure (died before treatment); 1 with transient hemiparesis at 2 weeks | 1 |
| Ertl L | 4 (2 additional with mRS 5) | 3 | 1—brainstem compression (after pneumonia) | 0 | 1 |
| Monteith S | 1 | 1 | 0 | 0 | 2 |
| Meckel S | 4 | 3 ischemic strokes | 1 mass effect; 1 hemorrhage | 1 flow diverter construct thrombosis | 2 |
| Siddiqui A | 4 | 5 | 1 (SAH) | NA | 2 |
| Van Oel L | 3 | 3 | 0 | 1—MI during anesthesia | 9 |
| Fischer S | NA | NA | NA | NA | 16 of 25 with DSA |
| Toth G | 1 | 2 strokes (1 died, 1 mRS of 5) | 0 | NA | 1 (secondary to construct thrombosis and occlusion) |
| Munich S | 1 | 2 strokes | 0 | 2 patients with new hemiparesis; 1 patient with new nystagmus/dizziness (ambulatory at 2 mo) | 9 |
| | **Deaths (in all aneurysms treated)** | **Ischemic Complications (in all aneurysms treated)** | **Hemorrhagic or Mass Effect Complications (in all aneurysms treated)** | **Complications not otherwise specified** | |
| | **23** | **20** | **5** | **7** | **38 of 123 total aneurysms cured** |

[a]BA, basilar artery; DSA, digital subtraction angiography; FD, flow diversion; MI, myocardial infarction; mRS, modified Rankin Scale; PIPELINE, pipeline embolization device (flow diverter); SAH, subarachnoid hemorrhage; SILK, silk flow diversion device; VBJ, vertebrobasilar junction.



Copyright © Congress of Neurological Surgeons. Unauthorized reproduction of this article is prohibited.

bifurcation aneurysm and 1 SCA aneurysm with 2 deaths),[40] Drake summarized: "the surgical treatment of these aneurysms will undoubtedly develop in some degree and it would appear that in certain instances in which expectancy of life is poor... a direct attack is justifiable." In 1968, he updated: "It is nice to be able to alter the gloomy statements of 2 years ago and encourage others with experience in the surgical treatment of aneurysms to operate on these lesions, with reason and caution, for they may not be as formidable as has been thought in the past."[41] The situation may be similar down the basilar trunk. There is a will among some neurosurgeons to fight this disease, and they need referrals, brave patients, supportive colleagues, and strong constitutions to persevere. It is difficult to say where this "work in progress" will lead, but it is, in our opinion, worth pursuing. There will certainly be more casualties along the way, but we hope to redeem ourselves and honor the patients who were harmed by applying the lessons we have learned and discovering a better solution for the dolichoectatic basilar trunk aneurysm, whatever it may be.

## Disclosures

Dr Saloner and his research work in this field are partly supported by a National Institutes of Health grant NS059944. Dr Rayz and his research work in this field are partly supported by a National Institutes of Health grant R01 HL115267. The authors have no personal, financial, or institutional interest in any of the drugs, materials, or devices described in this article.

## REFERENCES

1. Rayz VL, Boussel L, Lawton MT, et al. Numerical modeling of the flow in intracranial aneurysms: prediction of regions prone to thrombus formation. *Ann Biomed Eng.* 2008;36(11):1793-1804.
2. Rayz VL, Boussel L, Ge L, et al. Flow residence time and regions of intraluminal thrombus deposition in intracranial aneurysms. *Ann Biomed Eng.* 2010;38(10):3058-3069.
3. van Oel LI, van Rooij WJ, Sluzewski M, Beute GN, Lohle PN, Peluso JP. Reconstructive endovascular treatment of fusiform and dissecting basilar trunk aneurysms with flow diverters, stents, and coils. *AJNR Am J Neuroradiol.* 2013;34(3):589-595.
4. Drake CG. Ligation of the vertebral (unilateral or bilateral) or basilar artery in the treatment of large intracranial aneurysms. *J Neurosurg.* 1975;43(3):255-274.
5. Anson JA, Lawton MT, Spetzler RF. Characteristics and surgical treatment of dolichoectatic and fusiform aneurysms. *J Neurosurg.* 1996;84(2):185-193.
6. Byrne JV, Beltechi R, Yarnold JA, Birks J, Kamran M. Early experience in the treatment of intra-cranial aneurysms by endovascular flow diversion: a multicentre prospective study. *PLoS One.* 2010;5(9):e1212492.
7. Ertl L, Holtmannspotter M, Patzig M, Bruckmann H, Fesl G. Use of flow-diverting devices in fusiform vertebrobasilar giant aneurysms: a report on periprocedural course and long-term follow-up. *AJNR Am J Neuroradiol.* 2014;35(7):1346-1352.
8. Fischer S, Perez MA, Kurre W, Albes G, Bazner H, Henkes H. Pipeline embolization device for the treatment of intra- and extracranial fusiform and dissecting aneurysms: initial experience and long-term follow-up. *Neurosurgery.* 2014;75(4):364-374; discussion 374.
9. Lubicz B, Collignon L, Raphaeli G, et al. Flow-diverter stent for the endovascular treatment of intracranial aneurysms: a prospective study in 29 patients with 34 aneurysms. *Stroke.* 2010;41(10):2247-2253.
10. Meckel S, McAuliffe W, Fiorella D, et al. Endovascular treatment of complex aneurysms at the vertebrobasilar junction with flow-diverting stents: initial experience. *Neurosurgery.* 2013;73(3):386-394.
11. Monteith SJ, Tsimpas A, Dumont AS, et al. Endovascular treatment of fusiform cerebral aneurysms with the Pipeline Embolization Device. *J Neurosurg.* 2014;120(4):945-954.
12. Munich SA, Tan LA, Keigher KM, Chen M, Moftakhar R, Lopes DK. The Pipeline Embolization Device for the treatment of posterior circulation fusiform aneurysms: lessons learned at a single institution. *J Neurosurg.* 2014;121(5):1077-1084.
13. Raphaeli G, Collignon L, De Witte O, Lubicz B. Endovascular treatment of posterior circulation fusiform aneurysms: single-center experience in 31 patients. *Neurosurgery.* 2011;69(2):274-283.
14. Siddiqui AH, Abla AA, Kan P, et al. Panacea or problem: flow diverters in the treatment of symptomatic large or giant fusiform vertebrobasilar aneurysms. *J Neurosurg.* 2012;116(6):1258-1266.
15. Toth G, Bain M, Hussain MS, et al. Posterior circulation flow diversion: a single-center experience and literature review. *J Neurointerv Surg.* 2014;7(8):574-583.
16. Kalani MY, Zabramski JM, Nakaji P, Spetzler RF. Bypass and flow reduction for complex basilar and vertebrobasilar junction aneurysms. *Neurosurgery.* 2013;72(5):763-775; discussion 775-766.
17. Russell SM, Post N, Jafar JJ. Revascularizing the upper basilar circulation with saphenous vein grafts: operative technique and lessons learned. *Surg Neurol.* 2006;66(3):285-297.
18. Mai JC, Tariq F, Kim LJ, Sekhar LN. Flow diversion radial artery bypass graft coupled with terminal basilar artery occlusion for the treatment of complex basilar apex aneurysms: operative nuances. *Neurosurgery.* 2013;72(2 suppl operative):ons116-ons126; discussion ons126.
19. Evans JJ, Sekhar LN, Rak R, Stimac D. Bypass grafting and revascularization in the management of posterior circulation aneurysms. *Neurosurgery.* 2004;55(5):1036-1049.
20. Ewald CH, Kuhne D, Hassler WE. Bypass-surgery and coil-embolisation in the treatment of cerebral giant aneurysms. *Acta Neurochir (Wien).* 2000;142(7):731-737; discussion 737-738.
21. Gobble RM, Hoang H, Jafar J, Adelman M. Extracranial-intracranial bypass: resurrection of a nearly extinct operation. *J Vasc Surg.* 2012;56(5):1303-1307.
22. Horie N, Kitagawa N, Morikawa M, et al. Giant thrombosed fusiform aneurysm at the basilar trunk successfully treated with endovascular coil occlusion following bypass surgery: a case report and review of the literature. *Neurol Res.* 2007;29(8):842-846.
23. Jafar JJ, Russell SM, Woo HH. Treatment of giant intracranial aneurysms with saphenous vein extracranial-to-intracranial bypass grafting: indications, operative technique, and results in 29 patients. *Neurosurgery.* 2002;51(1):138-144; discussion 144-136.
24. Kaku Y, Funatsu N, Tsumimoto M, Yamashita K, Kokuzawa J. STA-MCA/STA-PCA bypass using short interposition vein graft. *Acta Neurochir Suppl.* 2014;119:79-82.
25. Kalani MY, Elhadi AM, Ramey W, et al. Revascularization and pediatric aneurysm surgery. *J Neurosurg Pediatr.* 2014;13(6):641-646.
26. Kalani MY, Zabramski JM, Nakaji P, Spetzler RF. Twenty-year follow-up of flow reversal and revascularization for a giant serpentine basilar artery aneurysm. *Neurosurgery.* 2014;10(suppl 3):E493-E497; discussion E497.
27. Kang HS, Oh CW, Han MH, Byun HS, Han DH. Treatment of a sequential giant fusiform aneurysm of the basilar trunk. *Korean J Radiol.* 2005;6(2):125-129.
28. Kim LJ, Tariq F, Sekhar LN. Pediatric bypasses for aneurysms and skull base tumors: short- and long-term outcomes. *J Neurosurg Pediatr.* 2013;11(5):533-542.
29. Shi X, Qian H, K CK, Zhang Y, Zhou Z, Sun Y. Bypass of the maxillary to proximal middle cerebral artery or proximal posterior cerebral artery with radial artery graft. *Acta Neurochir (Wien).* 2011;153(8):1649-1655; discussion 1655.
30. Shi X, Qian H, Singh KC, et al. Surgical management of vertebral and basilar artery aneurysms: a single center experience in 41 patients. *Acta Neurochir (Wien).* 2013;155(6):1087-1093.
31. Terasaka S, Itamoto K, Houkin K. Basilar trunk aneurysm surgically treated with anterior petrosectomy and external carotid artery-to-posterior cerebral artery bypass: technical note. *Neurosurgery.* 2002;51(4):1083-1087; discussion 1087-1088.
32. Tulleken CA, van der Zwan A, van Rooij WJ, Ramos LM. High-flow bypass using nonocclusive excimer laser-assisted end-to-side anastomosis of the external carotid artery to the P1 segment of the posterior cerebral artery via the sylvian route. Technical note. *J Neurosurg.* 1998;88(5):925-927.



Copyright © Congress of Neurological Surgeons. Unauthorized reproduction of this article is prohibited.

33. Rayz VL, Abla A, Boussel L, et al. Computational modeling of flow-altering surgeries in basilar aneurysms. *Ann Biomed Eng.* 2015;43(5):1210-1222.
34. Chwajol M, Munson TA, Alaraj A, Charbel FT, Aletich VA, Amin-Hanjani S. Extracranial carotid-vertebral bypass for endovascular access to complex posterior circulation aneurysms: a novel management approach. *Neurosurgery.* 2012;70(5): 1296-1303; discussion 12303-12304.
35. Jiang H, Ni W, Lei Y, Li Y, Gu Y. Combined extracranial-intracranial bypass surgery with stent-assisted coil embolization for moyamoya disease with a ruptured wide-necked basilar trunk aneurysm: a case report. *Turk Neurosurg.* 2015;25(1): 180-185.
36. Kakarla UK, Beres EJ, Ponce FA, et al. Microsurgical treatment of pediatric intracranial aneurysms: long-term angiographic and clinical outcomes. *Neurosurgery.* 2010;67(2):237-249; discussion 250.
37. Ponce FA, Albuquerque FC, McDougall CG, Han PP, Zabramski JM, Spetzler RF. Combined endovascular and microsurgical management of giant and complex unruptured aneurysms. *Neurosurg Focus.* 2004;17(5):E11.
38. Streefkerk HJ, Wolfs JF, Sorteberg W, Sorteberg AG, Tulleken CA. The ELANA technique: constructing a high flow bypass using a non-occlusive anastomosis on the ICA and a conventional anastomosis on the SCA in the treatment of a fusiform giant basilar trunk aneurysm. *Acta Neurochir (Wien).* 2004;146(9):1009-1019; discussion 1019.
39. Lin N, Brouillard AM, Krishna C, et al. Use of coils in conjunction with the pipeline embolization device for treatment of intracranial aneurysms. *Neurosurgery.* 2015;76(2):142-149.
40. Drake CG. Bleeding aneurysms of the basilar artery. Direct surgical management in four cases. *J Neurosurg.* 1961;18:230-238.
41. Drake CG. Further experience with surgical treatment of aneurysm of the basilar artery. *J Neurosurg.* 1968;29(4):372-392.

## COMMENTS

Dolichoectatic basilar trunk aneurysms are a true challenge for both neurosurgeons and interventionalists due to reasons well presented in the article. The natural history is known to be poor and the results after stenting and flow-diverting turned out somewhat disappointing after the initial promise and enthusiasm. Therefore, there is still need for surgical treatment: tailored bypasses and as the authors show preferring flow reduction than flow reversal. These difficult aneurysms often present with strokes and mass effect, but we still tend first to follow them and see what happens. According to the present series with a relatively long follow-up, only 44% of the patients experienced neurological deterioration and 20% died. This is better than in the previous series. In fact, larger series with a longer follow-up may show that the natural history of giant aneurysms in general may not be as bad as it has been thought earlier resembling that of a glioblastoma. However, when the patients are younger with a longer life-expectancy we must be able to offer therapy to prevent them to grow and thereby to reduce morbidity and mortality. The current article gives cookbook-type advice on how to plan the approach after imaging and flow measurements to compare alternative surgical options. This addresses the importance of a dedicated vascular center with enough cases to be able to perform the right kind of a bypass to preserve pertinent perforators not forgetting antiplatelets afterwards. It is amazing how the standard superficial temporal artery-middle cerebral artery bypass still stands the challenge of time as the workhorse in the field. We still wait for the breakthrough endovascular device to solve many of the problems in the treatment of giant and dolichoectatic aneurysms. I congratulate the authors for excellent and analytical work.

**Mika Niemelä**
*Helsinki, Finland*

Overall this is an exceptional article discussing the management and treatment strategies for dolichoectatic basilar trunk aneurysms. The data source with a prospectively collected database is appropriate for this uncommon disease, and the selection of pure basilar dolichoectasic basilar trunk aneurysms is to be commended. The review and evolution of the management techniques is logical and well described. Although routine imaging techniques of the aneurysms described is commonplace, the availability of computational fluid dynamic modeling is, arguably, sparse. As such, it bring challenges to adopting widespread use of these management strategies, even in larger cerebrovascular centers, less likely in current practice, although its value is certainly appreciated and highlighted here. It is likely that this will quickly evolve, as will the software itself, to a more accepted and available adjunct.

The technical descriptions are thorough, and often incorporate a hybrid approach of open and endovascular techniques that is to be emphasized. The challenges and importance of intracranial bypass has been discussed and debated by neurosurgeons since the extracranial-to-intracranial bypass study group,[1] but the availability of this skill set in the context of a complete cerebrovascular center should not be underemphasized, if for nothing else than a salvage option in these challenging cases. Lastly, the maintenance of the patency of perforators remains a challenge, and possibly an area of further focus from a pharmacologic approach, next-generation flow diversion, or possibly higher resolution computational fluid dynamic.

The morbidity and mortality of this series demonstrates improvement through the phases, and highlights the challenges of treating this disease. This article also suggests the importance of treatment of these lesions in high-volume centers, as they are indeed rare and outcomes will no doubt be improved. Lastly, the authors are to be commended on their humility with the presentation of their results. This represents a meaningful and important starting point for the management and treatment of this disease.

**Alexander Mason**
*Atlanta, Georgia*

1. The EC/IC Bypass Study Group. Failure of extracranial–intracranial arterial bypass to reduce the risk of ischemic stroke—results of an international randomized trial. *N Engl J Med.* 1985;313(19):1191-1200.



Copyright © Congress of Neurological Surgeons. Unauthorized reproduction of this article is prohibited.
