# Supplementary material for: Management strategies and outcomes of basilar trunk aneurysms: a systematic review and meta-analysis
Source: Langenbecks Arch Surg. 2026 Jan 20;411(1):71. doi: 10.1007/s00423-025-03959-3 (PMC12852281; doi:10.1007/s00423-025-03959-3)
Supplement: Supplementary file 1 — Supplementary file1 (ZIP 55327 KB) [file 423_2025_3959_MOESM1_ESM.zip › literature.zip!/meta/mu2014.pdf]

Clin Neuroradiol
DOI 10.1007/s00062-014-0369-4

ORIGINAL ARTICLE

Reconstructive Endovascular Treatment of Spontaneous Symptomatic Large or Giant Vertebrobasilar Dissecting Aneurysms: Clinical and Angiographic Outcomes

S. Mu · C. Li · X. Yang · Y. Wang · Y. Li · C. Jiang · Z. Wu

Received: 14 August 2014 / Accepted: 17 December 2014
© Springer-Verlag Berlin Heidelberg 2014

Abstract
Purpose To investigate the clinical and angiographic outcomes of spontaneous symptomatic large or giant vertebrobasilar dissecting aneurysms (VBDAs) following reconstructive endovascular treatment (EVT) with stent(s).
Methods We retrospectively identified 21 patients with spontaneous symptomatic large or giant VBDAs who had been treated with reconstructive EVT between September 2009 and September 2013 in our department. There were 20 men and 1 woman, with a mean age of 46.5 years (range: 17–67 years). Clinical and angiographic data were reviewed and evaluated.
Results Reconstructive EVT with sole stenting (SS, 10 cases) or stent-assisted coiling (SAC, 11 cases) technique was technically feasible in all cases. Angiographic follow-up results confirmed delayed thrombosis of the aneurysm sac in only two of the eight cases after SS, and recurrence of the aneurysm sac in six of the nine cases after SAC. Postoperative complications or poor neurologic outcomes occurred in seven cases (33.3 %). Three patients died after SS, including postoperative subarachnoid hemorrhage in one case and pulmonary embolism in one case. The other patient died soon after SAC, without radiologic result to confirm the causes of death. Other adverse events after treatment include ischemic complication in two cases, intramural hemorrhage in one case, aggravation of initial mass effect in one case. As to the other 14 cases, the post-procedural processes were uneventful and no complication was observed. The condition of them was improved in seven cases (33.3 %), and unchanged in seven cases (33.3 %) during followup.
Conclusions Reconstructive EVT with traditional intracranial stent(s) for the treatment of large and giant VBDAs carries a high failure rate and substantial risk of complications. The outcome of treating these difficult lesions with traditional intracranial stenting seems questionable and unpredictable.

Keywords Vertebrobasilar dissecting aneurysms · Endovascular treatment · Reconstructive · Stent · Giant · Large

C. Li, MS (✉) · S. Mu, MD · X. Yang, MD · Y. Wang, MD · Y. Li, MD · C. Jiang, MD · Z. Wu, MD
Beijing Neurosurgical Institute and Beijing Tiantan Hospital, Capital Medical University,
No. 6, Tiantan Xili,
Dongcheng District, Beijing 100050, People's Republic of China
e-mail: lichuanhui365@163.com

X. Yang, MD
e-mail: yang-xj@163.net

Introduction

Large and giant vertebrobasilar dissecting aneurysms (VBDAs) are rare lesions with very poor prognosis. Currently, appropriate management of these lesions remains controversial and lacks a standard protocol. If left untreated, approximately 80 % of patients with symptomatic large or giant VBDAs may be severely disabled or dead within 5 years of clinical presentation [1, 2]. Some cases might need aggressive surgical or endovascular treatment (EVT), especially when they presented with subarachnoid hemorrhage (SAH), progressive ischemia, or mass effect symptoms. Considering the morphological characteristics of these lesions and complicated anatomical structures in the posterior cranial fossa, surgical treatment is usually technically difficult and associated with high rates of morbidity

Published online: 25 December 2014

and mortality [3–5]. Hence, EVT has gradually become an important therapeutic option to manage these lesions [6–23].

The deconstructive EVT, such as internal coil trapping or proximal occlusion, is the best and most reliable technique for definitive obliteration of the dissecting aneurysm [6–8]. However, this treatment modality was not suitable and may be life threatening for patients without adequate collateral flow. Introduction of dedicated stent into the neurovascular system and ongoing technical refinement of intracranial stent characteristics have enabled the reconstructive therapy of the affected parent artery. And recently, reconstructive therapy using stents for VBDAs has been increasingly reported with promising results [9–23]. However, to the best of our knowledge, the literatures regarding reconstructive EVT for large or giant VBDAs are limited [9–13]. The aim of this study was to retrospectively investigate the clinical and angiographic outcomes of spontaneous symptomatic large and giant VBDAs after reconstructive EVT.

Materials and Methods

This retrospective study was approved by our hospital's institutional review board. Patients or their family members have given written informed consent to publish related materials.

Patients

Between September 2009 and September 2013, 21 consecutive patients with 21 spontaneous symptomatic large or giant VBDAs who underwent reconstructive EVT with sole stenting (SS) or stent-assisted coiling (SAC) technique in our department were enrolled in this study. Every patient underwent preoperative planar imaging examination, including computed tomography (CT) scanning or magnetic resonance (MR) imaging, and sometimes CT/MR angiography. Before treatment procedure, all patients underwent digital subtraction angiography (DSA) to confirm the diagnosis and to assist treatment decision making.

All patients met the inclusion criteria: (1) Clinical symptoms and/or signs relevant to the intracranial VBDAs, mainly including ischemic stroke, SAH or other presentations caused by the mass effect of the lesions; (2) Radiologic (CT/MR) and/or angiographic evidence (CT/MR angiography or conventional angiography) to diagnose VBDAs, including intimal flap, intramural hematoma, double-lumen, pearl-and-string sign, and retention of contrast medium in the involved segment of the parent vessel, configurational change of the lesion on serial imaging examination; (3) The aneurysm was large (defined as 12–25 mm in maximum diameter) or giant (>25 mm in maximum diameter). (4) The lesions that were treated with reconstructive EVT. Exclusion criteria included: (1) definitively traumatic or iatrogenic VBDAs, or lesions clearly related to vasculitis or fibromuscular dysplasia (because these lesions were thought to be another pathologic process and were not the focus of the present study); (2) incidentally found asymptomatic VBDAs (active intervention is imperative for patients with symptomatic VBDAs because of their poor natural history. As to asymptomatic VBDA, currently, appropriate management remains controversial and lacks a standard protocol. Therefore, in our study, we focused only on symptomatic VBDA because the indication of using EVT for treating them is definite.); (3) the lesions which were not "large" and "giant" according to the criteria mentioned above; (4) the lesions that were treated with deconstructive EVT.

The clinical and imaging characteristics of these 21 patients are presented in Table 1. There were 20 men and 1 woman, with ages ranging from 17 to 67 years (mean: 46.5 years). According to the clinical symptoms and/or signs and the MR/CT findings, clinical presentation were attributed to mass effect on the brain stem in 13 cases (case Nos. 2, 3, 5, 6, 7, 11, 12, 14, 16, 18, 19, 20, and 21), posterior circulation ischemic stroke in five cases (case Nos. 4, 9, 10, 13, and 17), SAH in one case (case No. 8), mass effect or posterior circulation ischemic stroke in one case (case No. 1), and uncertain in one case (case No. 15) who presented with agnogenic headache. Aneurysms were located at the vertebral artery (VA) in 1 case, the basilar artery (BA) in 10 cases, and the VA(s) and BA in 11 cases.

Treatment Protocol and Endovascular Techniques

In this series, indications for treatment of patients with VBDAs were discussed in a multidisciplinary team consisting of neurologists, neurosurgeons, and neuroradiologists. Every treatment was tailored to the individual patient on a case-by-case basis, considering various clinical and imaging parameters such as clinical presentation, patient age, the results of imaging (aneurysm size, type, and location), the availability of collateral flow, and patient preference. Reconstructive procedures with preservation of the parent artery were preferentially considered.

Patients with a history of acute SAH received a loading dosage of 300 mg of aspirin and 300 mg of clopidogrel 2 h prior to the procedures. The other patients received dual antiplatelet therapy including 100 mg of aspirin and 75 mg of clopidogrel from at least 3 days prior to the procedures. Dual antiplatelet therapy was maintained for 2 months postoperatively followed by aspirin monotherapy lasting indefinitely for the cases presenting with ischemic stroke; as to the other cases, succedent aspirin monotherapy lasted for 6 months. Some measures, including the antiplatelet effect test using thromboelastography and assessment of the CYP2C19 genotype or levels of P2Y12, were used to tailor

Table 1 Clinical and angiographic characteristics of 21 patients with large and giant vertebrobasilar dissecting aneurysms

Case No.	Sex	Age (years)	Initial mRS/ presentation	Aneurysm shape, site	EVT	Stents	Control angiogram	Complications	Angiographic follow-up (results/ modality/time)	Follow-up (mRS/time)
1	M	67	1/Ischemia, mass effect	Fusiform, middle–upper BA	SAC	EN × 2	PO	None	Recanalization/DSA/5 months	1/54 months
2	M	17	3/Mass effect	Dolichoectatic, middle BA,	SAC	EN × 2	PO	None	Partial thrombosis of the remnant aneurysmal sac/DSA/9 months	2/49 months
3	M	49	2/Mass effect	Fusiform, middle–lower BA, involving the VBJ	SS	SL × 3	No change	None	No change/DSA/5 months; enlargement of the dissecting sac/DSA/17 months, 23 months	2/24 months
4	M	55	1/Ischemia	Fusiform, middle–lower BA	SS	EN × 2	No change	None	Disappearance of double-lumen (DL)/DSA/5 months	0/48 months
5	M	29	1/Mass effect	Dolichoectatic, VBJ	SAC	EN × 1	STO	None	Recanalization/DSA/3 months	0/3 months
6	M	29	1/Mass effect	Fusiform, upper BA	SAC	EN × 1	STO	transient ischemic attack (TIA)	Recanalization/DSA/7 months	1/6 months
7	M	47	2/Mass effect	Dolichoectatic, VBJ,	SS	EN × 2	More retention	SAH	None	6/8 days
8	F	35	2/SAH	Fusiform, middle–upper BA	SAC	EN × 1	PO	None	Recanalization/DSA/2 months; Occlusion of the BA/DSA/16 months	0/36mons
9	M	39	5/Ischemia	Serpentine, left distal VA and middle–lower BA	SS	NF × 2 + WS × 1 + EN × 1	Blood flow improvement	Pulmonary embolism	None	4/2 months; 6/3 months
10	M	45	4/Ischemia	Fusiform, entire BA	SS	SL × 4	More retention	None	Thrombosis of the aneurysmal sac/ DSA/6 months	3/33 months
11	M	54	1/Mass effect	Fusiform, entire BA	SS	Leo × 1 + EN × 1	More retention	Intramural hemorrhage	Thrombosis of the aneurysmal sac/ DSA/6 months, 22 months	0/6 months; 1/24 months
12	M	46	0/Mass effect	Bilobulated, middle BA	SAC	SL × 1	STO	None	No change/CTA and MRA/1.5 months	1/28 months
13	M	44	2/Ischemia	Fusiform, lower BA and bilateral distal Vas	SS	SL × 4	No change	None	No change/DSA/11 months	2/26 months
14	M	66	1/Mass effect	Fusiform, middle BA	SAC	EN × 2	PO	Brain stem infarction	Total occlusion, occlusion of right AICA/DSA/6 months	4/15 months
15	M	55	1/Agnogenic headache	Serpentine, bilateral distal VA and middle–lower BA	SS	SL × 3	No change	None	Delayed retention/DSA/8 months	1/23 months
16	M	65	3/Mass effect	Dolichoectatic, left VA	SAC	EN × 1 + SL × 1	PO	None	None	3/22 months
17	M	20	2/Ischemia	Fusiform, entire BA	SAC	EN × 2	PO	None	Recanalization/DSA/7 months	1/53 months
18	M	64	2/Mass effect	Fusiform, lower BA and left distal VA	SS	SL × 3	No change	None	No change/DSA/6 months	2/20 months
19	M	54	3/Mass effect	Irregular, left VA	SAC	EN × 3	TO	Dyspnea, pneumonia	Recurrence/DSA/3 months	5/3 months
20	M	44	2/Mass effect	Dolichoectatic, lower BA	SAC	EN × 1 + SL × 1	PO	Died of SAH	None	6/17 h
21	M	52	2/Mass effect	Serpentine, middle–lower BA and left distal VA	SS	SL × 3	No change	None	No change/DSA/4 months	1/9 months

mRS modified Rankin score, *EVT* endovascular treatment, *M* male, *BA* basilar artery, *SAC* stent-assisted coiling, *EN* enterprise stent, *PO* partial occlusion, *DSA* digital subtraction angiography, *VBJ* vertebrobasilar junction, *SS* sole stenting, *SL* solitaire stent, *STO* subtotal occlusion, *SAH* subarachnoid hemorrhage, *F* female, *VA* vertebral artery, *NF* neuroform stent, *WS* wingspan stent, *TO* total occlusion, *CTA* computed tomography angiography, *MRA* magnetic resonance angiography, *AICA* anterior inferior cerebellar artery

each patient's antiplatelet strategy. Patients with insufficient response to aspirin or clopidogrel received another antiplatelet strategy, such as using the platelet glycoprotein IIb/IIIa receptor antagonist abciximab (Tirofiban), cilostazol, or ticagrelor as substitute.

All procedures were performed with the patients under general anesthesia. Anticoagulation during the treatment process included the intravenous bolus of 3000–5000 IU of heparin given at the beginning of each procedure and 1000 IU bolus administered intravenously thereafter every hour. Every coaxial catheter-flushing fluid was mixed with heparin at a concentration of 1000 IU of heparin per liter of saline. In all cases, vascular access to the lesions for reconstructive EVT was obtained via transfemoral approach using 6 Fr guiding catheter. First, diagnostic angiography and three-dimensional rotational angiography were performed. Following the diagnostic angiogram, the guiding catheter was navigated into VA, usually the preponderant one. In patients with two accessible VAs, a 5 or 6 Fr guiding catheter was placed in the contralateral VA for control angiograms and guidance of a second microcatheter if necessary.

SS or SAC technique was used in all cases. The choice of technique (SS or SAC) and the use of devices (e.g., stent type) were dependent on size and morphology of the aneurysm, the availability of devices, and anatomic geometry (e.g., the tortuosity of the parent vessel and whether the lesions involve vital perforators). For lesions involving the whole circumference of the parent vessel or vital perforators which supply the brainstem, or lacking a defined neck, SS was often preferred. However, this principle is not absolute. The final decision of treatment strategies was subjective, often at the discretion of the operator. The stents used in these cases include Solitaire AB (EV3), Enterprise (Codman cordis), Neuroform (Boston Scientific), Wingspan (Boston Scientific), and Leo (Balt). Under roadmapping, a microcatheter (Rebar 027, EV3; Prowler select plus, Codman cordis) or specialized delivery system (Vasco, Balt; Wingspan, Boston Scientific) was coaxially, by the use of a microguidewire (Silverspeed 14, EV3; Traxcess-14, Microvention; Transcend-14, Boston Scientific), navigated through the guiding catheter into the affected segment of the parent vessel to cover the lesions. For cases receiving SS, sometimes a single stent was not sufficient to alter intra-aneurysmal hemodynamics or completely cover the lesions, so subsequent overlapping stents or telescoping stents were deployed. As to the cases receiving SAC, stenting following coiling technique was used, as described before [15]. If available, modified coils, such as HydroCoils and fibered coil, were preferred. And multiple stents deployed in overlapping or telescoping fashion were also preferred, especially when the lesions were not completely occluded or in patients where long arterial segments needed to be bridged. In case of the lesions involving bilateral VAs, after deploying stents in the BA and unilateral VA, another stent was deployed via the mesh of the stent placed at the vertebrobasilar junction to cover the lesion on this side, either through the guiding catheter that was transferred from the contralateral VA or the guiding catheter placed previously.

When using SAC, we embolized the aneurysmal sac as much as possible in the condition that the vital perforators which supply the brainstem were not affected. However, because of the rich vital perforators originating from the vertebrobasilar system, total occlusion was often impossible. As to SS, retention of the contrast media in the aneurysmal sac or improvement of the blood flow in the parent vessel is an acceptable result, because this may be followed by a stimulation of thrombosis and reconstruction of the parent vessel. When there was still no retention after deploying three or more stents, we chose to finish the treatment and depend on the follow-up results to make further decision, because using too many stents in one stage might increase the risk of side branch or parent vessel occlusions due to in-stent thrombosis.

Follow-Up Protocol

Angiographic follow-up was generally recommended at 3–6 months after treatment, preferably using DSA. CT or MR angiography was regarded as an alternative for cases who refused to accept DSA as follow-up modality because of its invasiveness. Clinical follow-up information was acquired by means of outpatient visits and/or phone contact and evaluated according to modified Rankin scale. Endovascular retreatment was performed in three recanalized cases. The indications of retreatment were discussed in the multidisciplinary team and tailored to the individuals on a case-by-case basis, considering the clinical presentation when recurrence occurred and the extent of recurrence. Specifically, when dissecting aneurysm recanalized to be more than 20% of the original dissecting aneurysms compared with the control angiograms performed immediately after treatment, or the patient presented with obvious symptoms related to SAH, posterior circulation ischemia, or mass effect which were caused by the recanalized aneurysm, the patient was advised to accept retreatment.

Results

Reconstructive EVT with SS or SAC technique was technically feasible in all 21 cases. SS was used in 10 cases (case Nos. 3, 4, 7, 9, 10, 11, 13, 15, 18, and 21). Multiple stents deployed in overlapping (case Nos. 4, 7, and 11) or telescoping fashion (case Nos. 3, 9, 10, 13, 15, 18, and 21) was used in all of them. SAC was used in the other 11 cases (case Nos. 1, 2, 5, 6, 8, 12, 14, 16, 17, 19, and 20), with one stent

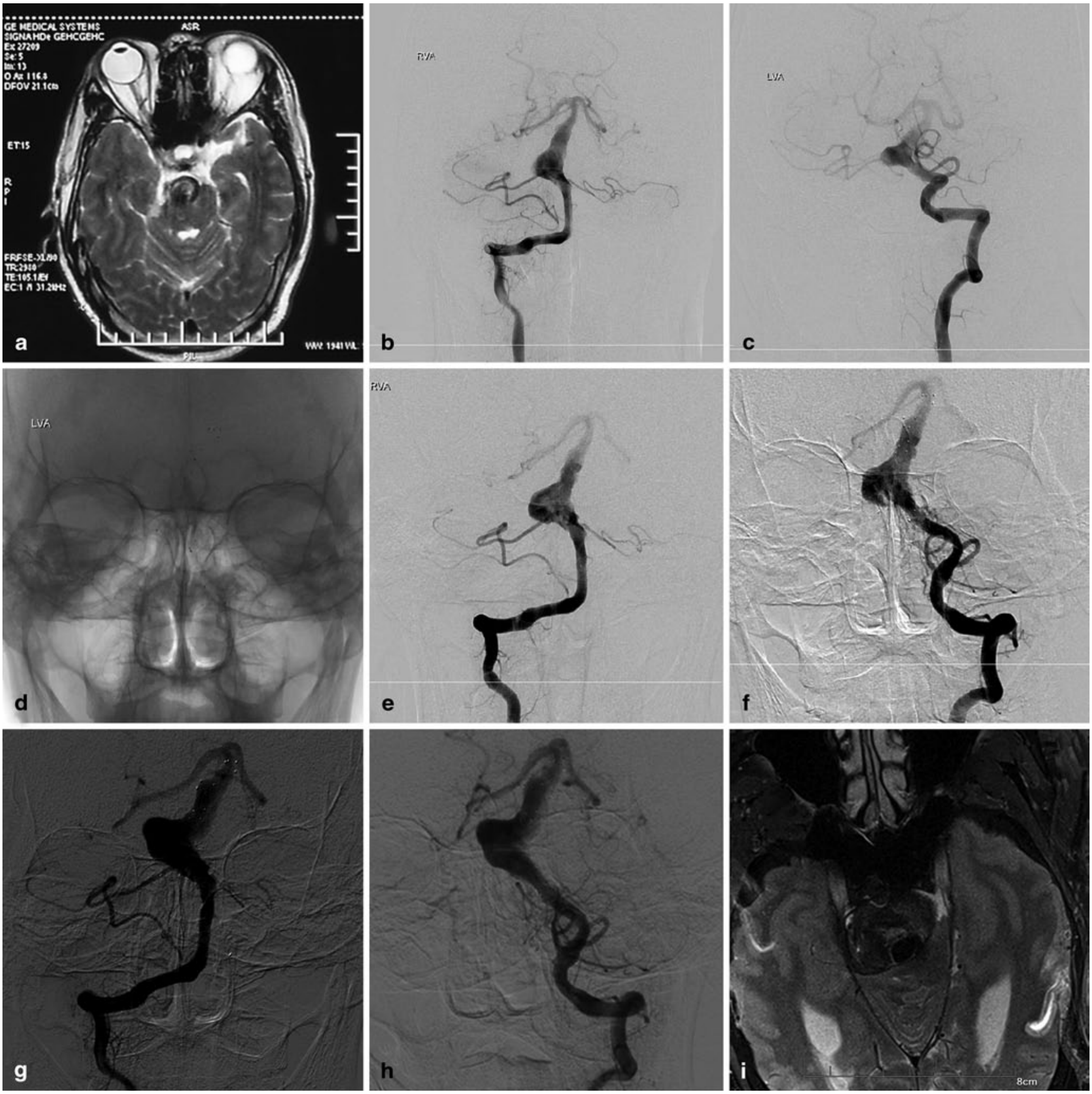

Fig. 1 A 49-year-old man presenting with dizziness, left weakness for 25 days (case 3). T2 weighted magnetic resonance (MR) imaging (**a**) and preoperative frontal view of bilateral vertebral angiograms (**b**, right; **c**, left) demonstrate a large fusiform dissecting aneurysm involving the middle–lower basilar artery (BA) and vertebrobasilar junction. **d** X-ray after placement of four telescoping stents. Note markers of the stents (**d**, *arrows*). Bilateral control vertebral angiograms performed immediately after stenting during procedure demonstrates no obvious hemodynamic change (figures not given). **e** and **f** Follow-up digital subtraction angiography (DSA) obtained 6 months later shows there is still no hemodynamic changes and no newly formed thrombosis in the aneurysm sac. **g** and **h** Follow-up angiograms obtained 17 months later show slight enlargement of the dissecting sac, which was confirmed by MR imaging (**i**). *Arrows* in **c**, **d**, **e**, and **f** show stent markers. The conditions of this patient was unchanged compared with the baseline condition after treatment (mRS = 2)

(case Nos. 5, 6, 8, and 12) or multiple stents (case Nos. 1, 2, 14, 16, 17, 19, and 20) deployed. Clinical and angiographic outcomes are summarized in Table 1. Representative cases are provided in Figs. 1–3.

Initial Angiographic Results

Immediately after SS or SAC, control angiography was performed in all 21 cases to evaluate post-procedural

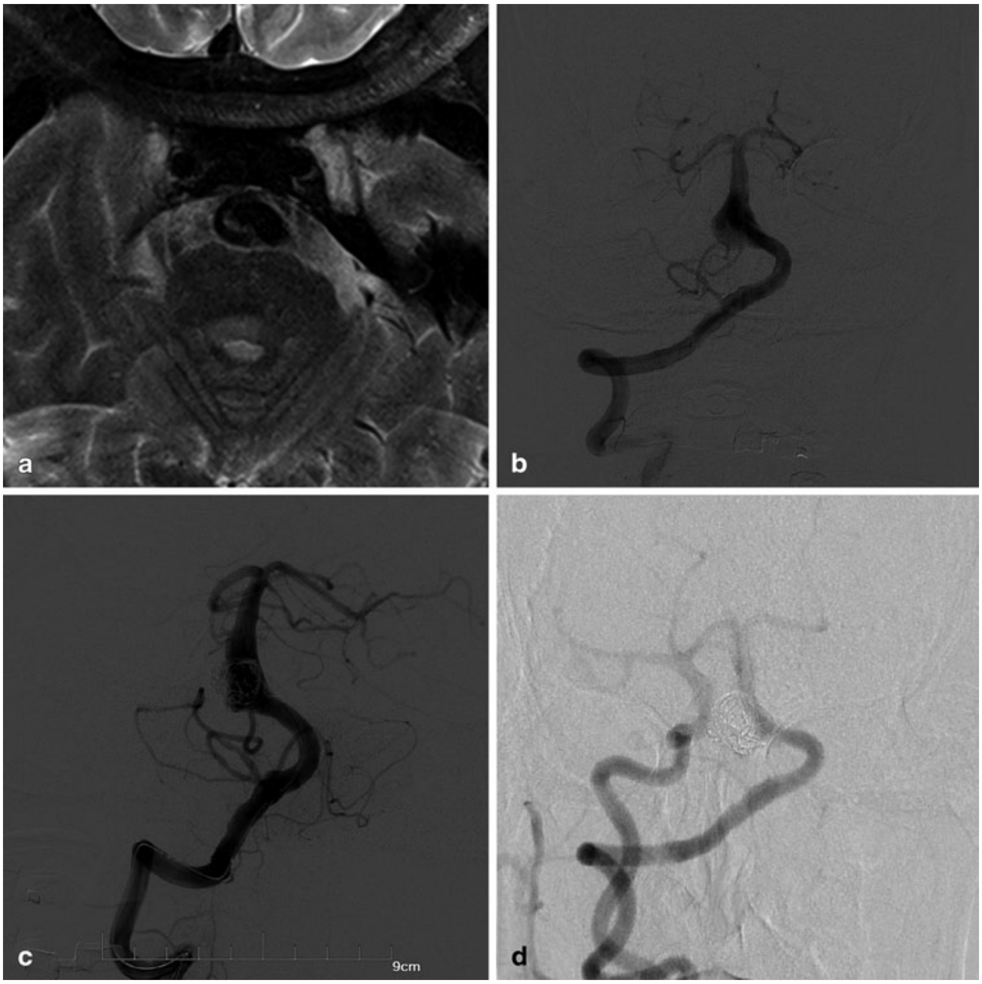

Fig. 2 A 66-year-old man (case 14) presenting with intermittent posterior headache and neck pain for 1 month. T2 weighted magnetic resonance (MR) imaging (**a**) and right vertebral angiograms (**b**) shows a partially thrombosed fusiform aneurysm of the basilar trunk. Note that the right anterior inferior cerebellar artery (AICA) originates from the lower part of the aneurysmal sac (**b**, *arrow*). Stent-assisted coiling was performed to partially occlude the aneurysm (**c**), with preservation of the right AICA (**c**, *arrow*). The patient developed right hemiplegia 2 months after embolization. **d** Follow-up digital subtraction angiography (DSA) obtained 6 months later confirmed delayed total thrombosis of the remnant aneurysmal sac. The patient still suffers from moderate disability at 15 months clinical follow-up (mRS = 4)

angiographic results. Immediately after SS or SAC, post-procedural control angiography showed total occlusion of the aneurysmal sac in 2 cases (9.5 %, case Nos. 6 and 19), subtotal occlusion in 2 cases (9.5 %, case Nos. 5 and 12), partial occlusion in 7 cases (33.3 %, case Nos. 1, 2, 8, 14, 16, 17, and 20), and unchanged perfusion or only hemodynamic change but no exclusion of the aneurysm sac in the other 10 cases (47.6 %) who were treated with SS.

Angiographic Follow-Up Results

Angiographic follow-up result was obtained in 17 cases, including 8 of the 10 cases undergoing SS (case Nos. 3, 4, 10, 11, 13, 15, 18, and 21) and 9 of the 11 cases undergoing SAC (cases Nos 1, 2, 5, 6, 8, 12, 14, 17, and 19). Angiographic results were as follows: total occlusion of the aneurysmal sac in 3 cases (17.6 %, case Nos. 10, 11, and 14), subtotal occlusion in 2 cases (11.8 %, case Nos. 2 and 12), recanalization of the occluded aneurysmal sac in 6 cases (35.3 %, case Nos. 1, 5, 6, 8, 17, and 19), no occlusion in 6 cases (35.3 %, case Nos. 3, 4, 13, 15, 18, and 21).

For case No. 8, subsequent follow-up angiograms obtained 16 months after the first DSA follow-up revealed delayed occlusion of the BA. No symptom related to the occluded BA was observed because the bilateral hypertrophied posterior communicating artery supplied the distal BA and its branches sufficiently. For case No. 3 (Fig. 1), subsequent follow-up angiograms obtained 17 and 23 months later show slight enlargement of the dissecting sac.

Complications and Clinical FollowUp

Postoperative complications or poor prognosis occurred in seven cases (33.3 %). Three patients (case Nos. 7, 9, and 20) died after treatment, and two of them (case Nos. 7 and 20) were considered to be related to procedure intervention. Case No. 7 presented with sudden headache, nausea, and vomiting 7 days after SS, and CT examination revealed a massive SAH. He died 5 days later. Case 20 presented with sudden unconsciousness, tachycardia, and breath arrest 3 h after SAC. His condition deteriorated so rapidly that he died 17 h after embolization. The exact reason why this patient died is uncertain because of the absence of radiologic result

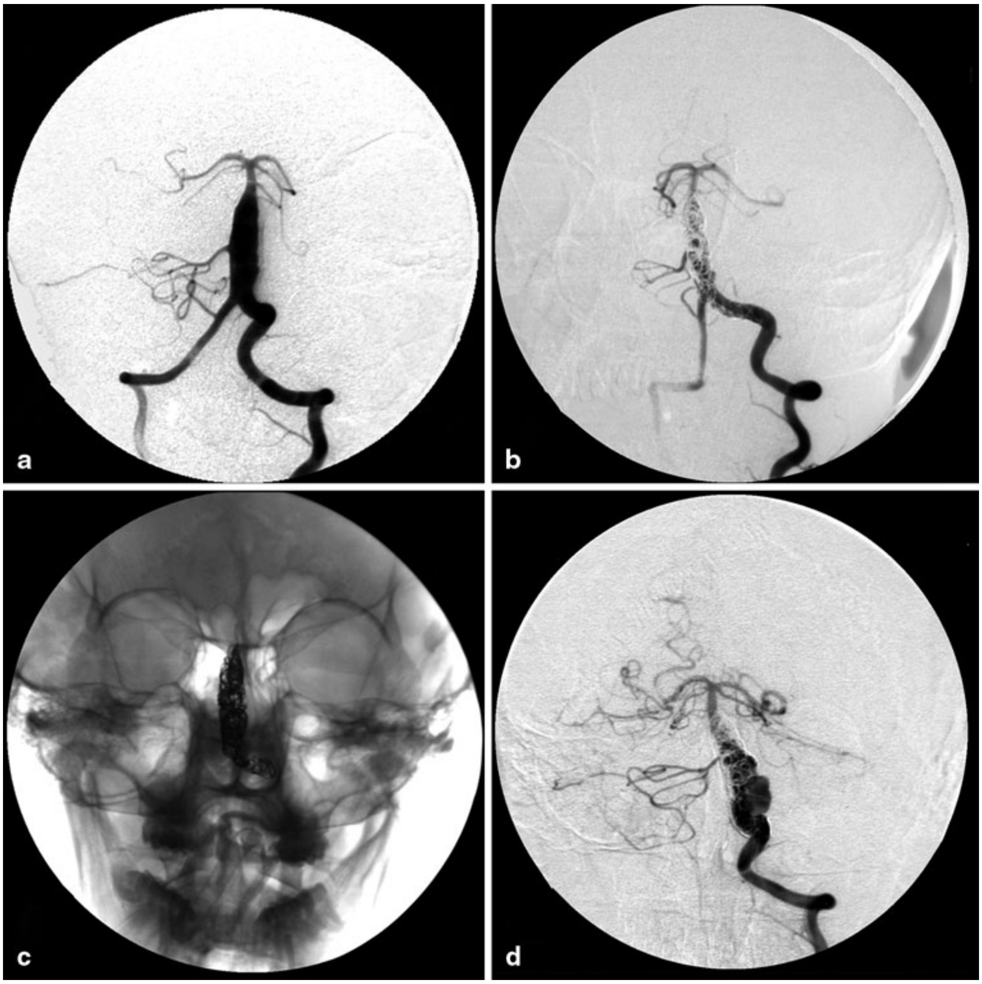

Fig. 3 A 20-year-old man (case 17) presenting with right limbs weakness, left facial paresis, and diplopia for 1.5 months. Preoperative magnetic resonance (MR) imaging shows a brain stem infarction (figure not given). Left vertebral angiograms (**a**) shows a fusiform aneurysm of the basilar trunk. Stent-assisted coiling with two overlapping stents was performed to partially occlude the aneurysm (**b**). The right AICA was preserved (**b**, *arrow*). **c** Frontal view of post-operative X-ray. **d** Follow-up DSA obtained 7 months later demonstrated recanalization of the fusiform aneurysm. The conditions of this patient was unchanged compared with the initial baseline condition when admitted for 12 months follow-up (mRS = 2). He was lost to follow-up after that

after SAC. The presumption is that the brainstem mass effect was aggravated, or in-stent thrombosis or SAH happened after SAC. Case No. 9, who presented with locked-in syndrome and was bed-ridden before procedure, unfortunately died of pulmonary embolism 3 months later although his condition had been gradually improving after treatment.

Complications of posterior ischemic stroke occurred in two cases (case Nos. 6 and 14). Case No. 6 presented with recurrent transient episode of right blurred vision 2 months after procedure, which gradually improved. No ischemia was detected on CT and MR imaging, hence, transient ischemic attack was considered to be the cause of blurred vision in this case. Case No. 14 presented with hemiplegia 2 months after SAC, and CT scan showed a brain stem infarction which was attributed to delayed occlusion of the right AICA that was previously preserved during procedure (Fig. 2). Both patients suffered from disability at last clinical follow-up (case No. 6, mRS = 2; case No. 14, mRS = 4).

For case No. 11, the symptom of right hemifacial spasm was improved gradually after SS had disappeared when he was admitted for 6 months DSA follow-up. However, the patient presented with right hemifacial anesthesia and anhidrosis 16 months after that, and MR imaging revealed an acute intramural hemorrhage. For case No. 19, the symptoms caused by mass effect were improved gradually in the first 2 months after SAC but aggravated after that. The patient presented with dyspnea with cough, and severe pneumonia when he was admitted for DSA follow-up at 3 months. Follow-up angiograms revealed recurrence of the lesion, and retreatment with internal trapping was performed to totally occlude the aneurysm.

As to the other cases, the post-procedural processes were uneventful and no complication was observed prior to patient discharge and during follow-up. The modified Rankin score (mRS) in seven cases (case Nos. 2, 4, 5, 8, 10, 17, and 21, 33.3 %) were improved during followup; the conditions of seven cases (case Nos. 1, 3, 12, 13, 15,16, and 18, 33.3 %) were unchanged compared with their baseline condition.

Retreatment

Endovascular retreatment was performed in three recanalized cases (case Nos. 5, 6, and 19) after SAC. The lesion

in case No. 5 was retreated with coiling via the mesh of the stent previously deployed, and was confirmed to be totally occluded by follow-up DSA 7 months after retreatment. This patient was asymptomatic when admitted for 35 months clinical follow-up. The recanalized lesion in case No. 6 was totally embolized with SAC. He presented with left weakness and numbness 9 months after retreatment, and CT examination confirmed pontine infarction which was attributable to delayed occlusion of the upper BA. This patient was still dependent in daily life (mRS = 2) at 14 months follow-up. For case No. 19, who was retreated with internal trapping technique, the angiographic follow-up was unavailable. The mass effect symptoms in this patient gradually improved after retreatment and the mRS was 2 at 15 months clinical follow-up.

Discussion

Large and giant VBDAs represent a small subset of intracranial aneurysms, and are often among the most ominous vascular lesions with an aggressive natural history [1, 2]. Despite the great advancements in microsurgery and EVT, the outcomes of patients with these lesions remain poor. Currently, reconstructive EVT with stents has been used as an important therapeutic option for vertebrobasilar dissections and dissecting aneurysms. However, apart from a few anecdotal cases, case series reports about using stents for the management of large and giant VBDAs were limited [9–13]. Here, we report the results of reconstructive EVT for management of these spontaneous symptomatic lesions.

Treatment Efficacy

Currently, appropriate treatment of the large and giant VBDAs is very challenging. Although measures such as multiple stents technique and use of modified coils were applied, the angiographic outcome after reconstructive EVT seems to be disappointing in this series. Total occlusion of the aneurysm was obtained in only 2 of the 10 cases treated with SAC, mainly because of the giant appearance, the irregular morphology and the wide neck or the long-affected parent vessel of these lesions. Additionally, these VBDAs nearly always involve important perforating arteries, and total occlusion of this subtype aneurysm will also occlude the perforating artery which may result in catastrophic ischemic complications. The recanalization rate in this series is high and some recanalized cases need retreatment, partly because only partial or subtotal occlusion of the lesions was obtained in most cases. In fact, even the lesions were densely packed and completely embolized as verified by angiogram, the true occlusion volume of the lesion was estimated to be about 30 % [24]. For cases receiving SS, no occlusion of the lesions was observed during procedure and only certain lesions responded to SS and benefited from this treatment during follow-up. For most cases, there was no change of the configuration of the lesions or hemodynamic remodeling in the control angiography during procedure or even in follow-up angiographic result. Some lesions (e.g. case No. 3, Fig. 1) even presented with delayed enlargement after a short-term stabilization. Dissecting aneurysms are likely the result of arterial dissections, which is physiologically active and dynamic compared with ordinary berry intracranial aneurysm [25–27]. For large and giant VBDAs, this “activeness” may be more active than other VBDAs, which may be the reason why these lesions grow chronically into “large or giant” [26]. This may be a reason why reconstructive EVT was disappointing for these lesions.

Many other issues needed to be discussed. First, whether this kind of treatment definitely improves the prognosis of patients with these large and giant VBDAs remains an unanswered issue. Improvement or stable condition of initial symptoms was observed in 14 cases, however, was the improvement of initial symptoms in the above cases caused by EVT, or that was the natural course of this disease, or that was caused by antiplatelet therapy in those who presented with initial ischemic stroke deserves to be discussed. Second, in most of these cases, the follow-up time is short, especially the angiographic follow-up. Whether this treatment modality improves the prognosis of patients with large and giant VBDAs in the long-term follow-up? It seems that traditional intracranial stent is oftentimes not enough for satisfactory management of these lesions. On the one hand, these lesions have a poor natural history with approximately 80 % of untreated patients dead or severely disabled [1, 2]. In view of this poor natural history, active treatment should be considered in these lesions. On the other hand, reconstructive EVT for these lesions carries substantial risk of complications, including ischemic stroke, rupture of the aneurysm, and aggravation of mass effect. Additionally, the recurrence or failure rate of this kind of treatment is high, with some patients requiring further treatment.

Reconstructive Modality Selection

Reconstructive strategies using stents for treatment of VBDAs includes SAC and SS. In most circumstance, complete embolization of the VBDAs with SAC is ideal because coiling decreases blood flow impingement into the fragile vessel wall of the lesion and reduces wall stress of the aneurysm sac, which is beneficial for thrombosis within the aneurysmal sac, thereby facilitating long-term healing of the lesions and decreasing the possibility of future bleeding compared with stent alone treatment (SAT). However, VBDAs are likely the result of arterial dissections. Because of the physiologically active characteristic of dissection,

these lesions frequently exhibit a fusiform, dolichoectatic, serpentine, or giant appearance due to the repeated spontaneous reparative process of intraluminal thrombosis and clot lysis, and often involve the whole circumference of the parent vessel and lack a defined neck [25–27].

Besides, some of these lesions involve vital perforators, the occlusion of which will lead to ischemic complications. Therefore, SAC is not feasible for some lesions because the coils may protrude into the parent artery or nearby large perforators, thus resulting in catastrophic outcome. Then, SS is an alternative. There is theoretical and experimental background supporting the use of SS technique for management of these lesions, such as hemodynamic remodeling, repair of the intimal flap, and facilitation of neo-endothelialization of the aneurysm neck or the injured parent vessel [16–21]. Multiple stents deployed in overlapping or telescoping technique is based on the theoretical mechanism of the less porous mesh causing greater intra-aneurysmal hemodynamic changes, also providing relatively stronger radial force for repair of the intimal flap, which is similar to that of the relatively novel flow-diverting stents (FDS). For the lesions which were not completely occluded with SAC and lesions involving long segment of the parent vessel which could not be completely covered with only one stent, multiple stents technique was preferred in our department. In fact, some reports have suggested that multiple stents technique could increase the total occlusion rate and benefit for long term of the lesions after treatment [22, 23]. Of course, we should note that deploying too many stents in one stage might increase the risk of side branch or parent vessel occlusions due to in-stent thrombosis. This issue supports the concept of a staged procedure with initial defensive implantation of stents followed by additional management dependent on the follow-up results, which allows the ability to decrease the risk of ischemic events, and appears to be a viable treatment option [28].

The dismal outcomes of standard microsurgical and EVT prompt us to develop a new treatment modality for these large and giant VBDAs. Some reports have demonstrated promising results of using FDS, such as the pipeline embolization device (ev3, Neurovascular, Irvine, CA, USA) and Silk (Balt Extrusion, Montmorency, France) for treatment of fusiform and dissecting aneurysms [9, 11, 28]. However, we have no direct experience of using FDS in the vertebrobasilar system. We expect more valuable data of using FDS for large and giant VBDAs, which may act as a breakthrough concept for satisfactory management of these daunting lesions.

Limitations

Several limitations of this study needs to be noted, including the retrospective nature, monocentric approach, the relatively small case series, and limited follow-up duration. The small case series consisting of only 21 patients does not allow statistically definitive conclusions. Nevertheless, considering the fact that large and giant VBDAs are rare and are among the most difficult neurovascular lesions to treat, the results of this study may be an important message to better understanding these lesions and provide the basis for further study.

In conclusion, our limited series suggest that reconstructive EVT with traditional intracranial stent(s) for the treatment of large and giant VBDAs carries a high failure rate and substantial risk of complications. The outcome of treating these difficult lesions with traditional intracranial stenting seems questionable and unpredictable. New treatment modality, such as reconstructive treatment using FDS, may be a breakthrough concept for management of these monster vascular lesions.

Acknowledgements This work was supported by the National Science Foundation of China (grant Nos. 81220108007, 81171079, 81371315, and 81471167) and Special Research Project for Capital Health Development (grant Nos. 2011-1015-04 and 2014-1-1071).

Conflicts of Interest None.

References

1. Steinberg GK, Drake CG, Peerless SJ. Deliberate basilar or vertebral artery occlusion in the treatment of intracranial aneurysms. Immediate results and long-term outcome in 201 patients. J Neurosurg. 1993;79:161–73.
2. Drake CG, Peerless SJ. Giant fusiform intracranial aneurysms: review of 120 patients treated surgically from 1965 to 1992. J Neurosurg. 1997;87:141–62.
3. Aziz KMA, van Loveren HR, Tew JM, Jr, Chicoine MR. The Kawase approach to retrosellar and upper clival basilar aneurysms. Neurosurgery. 1999;44:1225–36.
4. Lawton MT, Daspit PD, Spetzler RF. Technical aspects and recent trends in the management of large and giant midbasilar artery aneurysms. Neurosurgery. 1997;41:513–21.
5. Kalani MY, Zabramski JM, Nakaji P, Spetzler RF. Bypass and flow reduction for complex basilar and vertebrobasilar junction aneurysms. Neurosurgery. 2013;72:763–75.
6. Iihara K, Sakai N, Murao K, Sakai H, Higashi T, Kogure S, Takahashi JC, Nagata I. Dissecting aneurysms of the vertebral artery: a management strategy. J Neurosurg. 2002;97:259–67.
7. Rabinov JD, Hellinger FR, Morris PP, Ogilvy CS, Putman CM. Endovascular management of vertebrobasilar dissecting aneurysms. AJNR Am J Neuroradiol. 2003;24:1421–8.
8. Leibowitz R, Do HM, Marcellus ML, Chang SD, Steinberg GK, Marks MP. Parent vessel occlusion for vertebrobasilar fusiform and dissecting aneurysms. AJNR Am J Neuroradiol. 2003;24:902–7.
9. Meckel S, McAuliffe W, Fiorella D, Taschner CA, Phatouros C, Phillips TJ, Vasak P, Schumacher M, Klisch J. Endovascular treatment of complex aneurysms at the vertebrobasilar junction with flow-diverting stents: initial experience. Neurosurgery. 2013;73:386–94.
10. van Oel LI van Rooij WJ Sluzewski M Beute GN Lohle PN Peluso JP. Reconstructive endovascular treatment of fusiform and dissecting basilar trunk aneurysms with flow diverters, stents, and coils. AJNR Am J Neuroradiol. 2013;34:589–95.

11. Fiorella D, Kelly ME, Albuquerque FC, Nelson PK. Curative reconstruction of a giant midbasilar trunk aneurysm with the pipeline embolization device. Neurosurgery. 2009;64:212–7.
12. Siddiqui AH, Abla AA, Kan P, Dumont TM, Jahshan S, Britz GW, Hopkins LN, Levy EI. Panacea or problem: flow diverters in the treatment of symptomatic large or giant fusiform vertebrobasilar aneurysms. J Neurosurg. 2012;116:1258–66.
13. Ertl L, Holtmannspötter M, Patzig M, Brückmann H, Fesl G. Use of flow-diverting devices in fusiform vertebrobasilar giant aneurysms: a report on periprocedural course and long-term follow-up. AJNR Am J Neuroradiol. 2014;35:1346–52.
14. Phillips TJ, Wenderoth JD, Phatouros CC, Rice H, Singh TP, Devilliers L, Wycoco V, Meckel S, McAuliffe W. Safety of the pipeline embolization device in treatment of posterior circulation aneurysms. AJNR Am J Neuroradiol. 2012;33:1225–31.
15. Wang Y, Yang X, Youxiang L, Shiqing M, Chuhan J, Zhongxue W, Kocer N. Treatment of symptomatic fusiform aneurysm in basilar artery by stenting following coiling technique. Turk Neurosurg. 2014;24:44–7.
16. Fiorella D, Albuquerque FC, Deshmukh VR, Woo HH, Rasmussen PA, Masaryk TJ, McDougall CG. Endovascular reconstruction with the Neuroform stent as monotherapy for the treatment of uncoilable intradural pseudoaneurysm. Neurosurgery. 2006;59:291–300.
17. Geremia G, Brack T, Brennecke L, Haklin M, Falter R. Occlusion of experimentally created fusiform aneurysms with porous metallic stents. AJNR Am J Neuroradiol. 2000;21:739–45.
18. Rhee K, Han MH, Cha SH. Changes of flow characteristics by stenting in aneurysm models: influence of aneurysm geometry and stent porosity. Ann Biomed Eng. 2002;30:894–904.
19. Mizutani T, Kojima H, Asamoto S. Healing process for cerebral dissecting aneurysms presenting with subarachnoid hemorrhage. Neurosurgery. 2004;54:342–8.
20. Bai H, Masuda J, Sawa Y, Nakano S, Shirakura R, Shimazaki Y, Ogata J, Matsuda H. Neointima formation after vascular stent implantation. Spatial and chronological distribution of smooth muscle cell proliferation and phenotypic modulation. Arterioscler Thromb. 1994;14:1846–53.
21. Yoon WK, Kim YW, Kim SD, Park IS, Baik MW, Kim SR. Intravascular ultrasonography-guided stent angioplasty of an extracranial vertebral artery dissection. J Neurosurg. 2008;109:1113–8.
22. Zhao KJ, Zhao R, Huang QH, Xu Y, Hong B, Fang YB, Li Q, Yang PF, Liu JM, Zhao WY. The interaction between stent(s) implantation, PICA involvement, and immediate occlusion degree affect symptomatic intracranial spontaneous vertebral artery dissection aneurysm (sis-VADA) recurrence after reconstructive treatment with stent(s)-assisted coiling. Eur Radiol. 2014;24:2088–96.
23. Zhao KJ, Fang YB, Huang QH, Xu Y, Hong B, Li Q, Liu JM, Zhao WY, Deng BQ. Reconstructive treatment of ruptured intracranial spontaneous vertebral artery dissection aneurysms: long-term results and predictors of unfavorable outcomes. PLoS One. 2013;8:e67169.
24. Tamatani S, Ito Y, Abe H, Koike T, Takeuchi S, Tanaka R. Evaluation of the stability of aneurysms after embolization using detachable coils: correlation between stability of aneurysms and embolized volume of aneurysms. AJNR Am J Neuroradiol. 2002;23:762–7.
25. Mizutani T, Miki Y, Kojima H, Suzuki H. Proposed classification of nonatherosclerotic cerebral fusiform and dissecting aneurysms. Neurosurgery. 1999;45:253–60.
26. Mizutani T. A fatal, chronically growing basilar artery: a new type of dissecting aneurysm. J Neurosurg. 1996;84:962–71.
27. Schubiger O, Valavanis A, Wichmann W. Growth-mechanism of giant intracranial aneurysms; demonstration by CT and MR imaging. Neuroradiology. 1987;29:266–71.
28. Fischer S, Perez MA, Kurre W, Albes G, Bäzner H, Henkes H. Pipeline embolization device for the treatment of intra- and extracranial fusiform and dissecting aneurysms: initial experience and long-term follow-up. Neurosurgery. 2014;75:364–74.
