# Supplementary material for: Management strategies and outcomes of basilar trunk aneurysms: a systematic review and meta-analysis
Source: Langenbecks Arch Surg. 2026 Jan 20;411(1):71. doi: 10.1007/s00423-025-03959-3 (PMC12852281; doi:10.1007/s00423-025-03959-3)
Supplement: Supplementary file 1 — Supplementary file1 (ZIP 55327 KB) [file 423_2025_3959_MOESM1_ESM.zip › literature.zip!/meta/Nakatomi 2020.pdf]

# Giant Fusiform and Dolichoectatic Aneurysms of the Basilar Trunk and Vertebrobasilar Junction—Clinicopathological and Surgical Outcome

Hirofumi Nakatomi, MD, PhD[‡§*]
Satoshi Kiyofuji, MD[‡*]
Hideaki Ono, MD, PhD[‡]
Minoru Tanaka, MD, PhD[‡]
Hiroyasu Kamiyama, MD[¶]
Katsumi Takizawa, MD[||]
Hideaki Imai, MD, PhD[‡]
Nobuhito Saito, MD, PhD[‡]
Yoshiaki Shiokawa, MD, PhD[#]
Akio Morita, MD, PhD[**]
Kelly D. Flemming, MD[‡‡]
Michael J. Link, MD[§§]

[‡]Department of Neurosurgery, The University of Tokyo Hospital, Tokyo, Japan; [§]Division of Collaborative Laboratory for Medical Science of Neural Dynamics, RIKEN Center for Brain Science, Saitama, Japan; [¶]Department of Neurosurgery and Stroke, Teishinkai Hospital, Hokkaido, Japan; [||]Department of Neurosurgery, Japanese Red Cross Asahikawa Hospital, Hokkaido, Japan; [#]Department of Neurosurgery, Kyorin University, Tokyo, Japan; [**]Department of Neurosurgery, Nippon Medical University, Tokyo, Japan; [‡‡]Department of Neurology, Mayo Clinic, Rochester, Minnesota; [§§]Department of Neurologic Surgery, Mayo Clinic, Rochester, Minnesota

*Hirofumi Nakatomi and Satoshi Kiyofuji contributed equally to this work.

**Correspondence:**
Hirofumi Nakatomi, MD, PhD,
Department of Neurosurgery,
The University of Tokyo Hospital,
7-3-1 Hongo,
Tokyo 113-8655, Japan.
Email: hnakatomi-tky@umin.ac.jp

**Received,** September 15, 2019.
**Accepted,** May 24, 2020.

© Congress of Neurological Surgeons 2020.

This is an Open Access article distributed under the terms of the Creative Commons Attribution-NonCommercial-NoDerivs licence (http://creativecommons.org/licenses/by-nc-nd/4.0/), which permits non-commercial reproduction and distribution of the work, in any medium, provided the original work is not altered or transformed in any way, and that the work is properly cited. For commercial re-use, please contact journals.permissions@oup.com

**BACKGROUND:** Giant fusiform and dolichoectatic aneurysms of the basilar trunk and vertebrobasilar junction (BTVBJ-GFDA) are extremely difficult to treat.

**OBJECTIVE:** To evaluate factors influencing survival and outcome of BTVBJ-GFDA by performing a retrospective multicenter cohort study.

**METHODS:** A total of 32 patients with BTVBJ-GFDA were included in this study. Clinicopathological characteristics, treatment measures, and outcomes were collected from medical records and imaging studies. Autopsy and histological findings of the aneurysm and adjacent brain tissue were also obtained in 9 cases.

**RESULTS:** A total of 11 patients did not undergo surgery, of whom 10 died; 3 from progressive brainstem compression, 4 from subarachnoid hemorrhage, 2 from brainstem infarction, and 1 from associated atherosclerotic disease. The remaining 21 patients underwent a surgical treatment, consisting of immediately proximal parent artery occlusion, remotely proximal parent artery occlusion, clip reconstruction, and distal bypass and achieved significantly longer overall survival compared with those who received conservative therapy (adjusted hazard ratio 1.508, 95% CI 1.058-2.148, $P = .02$). Histological examination of the aneurysms demonstrated staged clots, open lumen, and intrathrombotic channels with endothelial lining. The patients younger than 45 yr of age showed statistically longer survival than those equal and older than 45 yr ($P = .03$).

**CONCLUSION:** Surgical intervention achieved greater survival than conservative management in BTVBJ-GFDA. Narrow ideal treatment window of the blood flow within the aneurysm to maintain sufficient but not excess supply should be targeted based on the hemodynamics of both the posterior communicating arteries and perforating vessel collaterals.

**KEY WORDS:** Basilar trunk, Dolichoectasia, Dolichoectatic aneurysms, Fusiform aneurysms, Megadolichobasilar anomaly, Vertebrobasilar junction

*Neurosurgery 0:1–14, 2020* DOI:10.1093/neuros/nyaa317 www.neurosurgery-online.com

Giant fusiform and dolichoectatic aneurysms of the basilar trunk and vertebrobasilar junction (BTVBJ-GFDA) are rare vascular anomalies with histological prevalence report of 0.17% to 5.8%.[1,2] Anatomic, hemodynamic, and biological factors are reported as the triggers for the dolichoectatic processes,[3] and prior studies demonstrated

**ABBREVIATIONS: BTVBJ-GFDA,** giant fusiform and dolichoectatic aneurysms of the basilar trunk and vertebrobasilar junction; **CI,** confidence interval; **CR,** clip reconstruction; **DB,** distal bypass; **IPO,** immediately proximal occlusion; **IU,** international unit; **MRI,** magnetic resonance imaging; **mRS,** modified Rankin Scale; **Pcom,** posterior communicating artery; **PVCC,** perforating vessel collateral circulation; **RPO,** remotely proximal occlusion; **SAH,** subarachnoid hemorrhage

Supplemental digital content is available for this article at www.neurosurgery-online.com.



Downloaded from https://academic.oup.com/neurosurgery/article-abstract/doi/10.1093/neuros/nyaa317/5880069 by guest on 06 August 2020

pathological resemblance of fusiform and dolichoectatic aneurysms,[4,5] but detailed developing mechanisms remain unknown. BTVBJ-GFDA manifests as clinical symptoms caused by thrombosis or mass effect compressing the brainstem, which usually progress to death if untreated.[6-11] Unfortunately, surgical treatment is extremely difficult because of the location, absence of discrete aneurysm neck, and presence of thrombosis, with limited reports of satisfactory outcomes.[6,7,9,11] In general, various combinations of occlusion of the parent arteries to reduce blood supply to the aneurysm with or without bypass construction to compensate by a nonphysiological route have been adopted, and recently, endovascular treatment with flow diverters has been performed.[12-14] Still, choosing the optimal treatment for individuals is complexed. The present study reviewed our surgical series of BTVBJ-GFDA to retrospectively analyze the treatment outcomes to elucidate the factors affecting outcomes.

# METHODS

## Patient Demographics

A total of 32 patients with BTVBJ-GFDA treated at 2 international institutions between April 1980 and September 2015 were included. Cerebral angiography and computed tomography were performed in all cases for diagnosis, whereas magnetic resonance imaging (MRI) was obtained in most cases. We defined GFDA as nonsaccular, fusiform, and dolichoectatic aneurysms, with giant GFDA having maximum diameter of 25 mm or larger and large GFDA of 15 mm or larger measured on axial, coronal, and sagittal sections. All patients were followed up with angiogram and MRI when available. Treatment planning involved both neurosurgeons and interventional neuroradiologists, including surgical, endovascular, or combinations of both approaches. This study was approved by the internal review board of each hospital. All the patients provided written consent for future publication in academic purposes in anonymous fashion at the time of treatment, and this report has implemented Strengthening the reporting of observational studies in epidemiology guideline.

Medical charts and imaging studies were reviewed to collect such data as patient age, systemic atherosclerotic risks, and related diseases (eg, hypertension, coronary artery disease, history of smoking, and abdominal aorta aneurysm),[8,15] and angiographical features including patency of the bilateral posterior communicating arteries (Pcoms). Treatment types were divided into 4 categories: immediately proximal parent artery occlusion (IPO) and remotely proximal parent artery occlusion (RPO: clips were applied more proximally than IPO), both intending flow reversal to facilitate aneurysm thrombosis, clip reconstruction (CR) to eliminate the flow within the aneurysm, and distal bypass with or without branch artery occlusion (DB) to achieve the flow reduction within the aneurysm (Figure 1). IPO and RPO also entailed bypass selectively depending on the results of preoperative angiogram and balloon occlusion test. Clinical outcome was assessed with modified Rankin Scale (mRS) at 1 mo after surgery and at the last follow-up. Favorable collateral was defined positive when bilateral large (more than 1 mm) Pcoms were demonstrated on angiographical collateral assessment with the balloon occlusion and/or Allcock test.

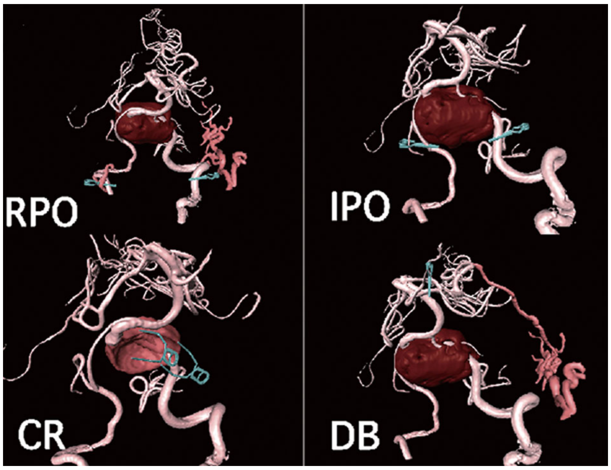


**FIGURE 1.** *Schemas of 4 different types of surgical treatments: remotely proximal parent artery occlusion (RPO), immediately proximal parent artery occlusion (IPO), clip reconstruction (CR), and distal bypass with or without branching artery occlusion (DB). Note that in RPO, collateral development extends from the external carotid system (arrowhead) or muscular branches of the extracranial vertebral artery (arrow) into the intracranial vertebral and basilar arteries.*

## Histopathological Analysis

In mortality cases, aneurysm tissues were obtained at autopsy and were histologically examined after fixation in 10% buffered formalin and embedded in paraffin. Tissue sections with 4-$\mu$m thickness were made and stained with hematoxylin and eosin stains. Immunohistochemical detection of factor VIII was performed using antiserum for factor VIII-related antigen (DAKO Corp, Carpinteria, California; polyclonal, dilution 1:1500) as the primary antibody.

## Statistical Analysis

Those mention above clinical, histological, and radiographical data were collected, whose frequencies were calculated. We performed a chi-square test or Fisher's exact test for categorical variables and the Kruskal-Wallis test for continuous variables. Overall survival was defined as the duration from the date of treatment until the date of death or the last follow-up. Overall survival was estimated using the Kaplan-Meier method, and comparison between age groups was performed with the log-rank test. Cox proportional hazard model was utilized to identify prognostic factors that might influence survival. Adjusted hazard ratios for death were calculated after adjusting for sex, age, aneurysm size, location, atherosclerotic risk, bilateral Pcom patency, and preoperative mRS. Univariate and multivariate Cox regression analyses were performed to assess factors related to survival. Statistical significance was set at $P < .05$. All data analyses used Dr SPSS II version 11.01 (IBM, Armonk, New York) for Windows.

# RESULTS

## Clinical Manifestations

The patient demographics of 32 patients (12 women and 20 men, mean age 56.8 yr) are summarized in Table 1. The

Downloaded from https://academic.oup.com/neurosurgery/article-abstract/doi/10.1093/neuros/nyaa317/5880069 by guest on 06 August 2020

**TABLE 1. Patient Demographics**

| Variable | All included patients | Surgically treated patients | Conservatively managed patients | *P* value, surgery vs conservative management |
|---|---|---|---|---|
| Number of patients | 32 | 21 | 11 | |
| Sex, female: male | 12: 20 | 9: 12 | 3: 8 | .54 |
| Age, ≥45 yr: < 45 yr | 27: 5 | 16: 5 | 11: 0 | .078 |
| Location, BAT: VBJ | 18: 14 | 11: 10 | 7: 4 | .54 |
| An size, mean ± SD (mm) | 27.62 ± 5.57 | 28.3 ± 6.44 | 26.4 ± 6.41 | .39 |
| Atherosclerosis risk, no: yes | 7: 25 | 5: 16 | 2: 9 | .71 |
| Bilateral Pcom patency, favorable: poor | 5: 27 | 5: 16 | 0: 11 | .078 |
| Preoperative mRS, 1: 2: 3: 4: 5 | 4: 2: 10: 9: 7 | 4: 1: 7: 6: 3 | 0: 1: 3: 3: 4 | .10 |
| Tx category, IPO: RPO: CR: DB: no Tx | 7: 6: 5: 3: 11 | 7: 6: 5: 3: 0 | 0: 0: 0: 0: 11 | |
| Construction of bypass, no: yes | 20: 12 | 20: 12 | 0: 0 | |
| Postoperative mRS[a], 1: 2: 3: 4: 5: 6 | 3: 2: 6: 8: 9: 4 | 3: 2: 6: 8: 9: 4 | NA | |
| Latest follow-up mRS, 1: 2: 3: 4: 5: 6 | 5: 1: 1: 1: 2: 22 | 5: 0: 1: 1: 2: 12 | 0: 1: 0: 0: 0: 10 | .11 |
| Follow-up from latest Tx, mean ± SD (mo) | 45.21 ± 53.60 | 56.1 ± 60.8 | 19.4 ± 26.7 | .10 |

An indicates aneurysm; BAT, basilar trunk; CR, clip reconstruction; DB, distal bypass; IPO, immediate proximal occlusion; mRS, modified Rankin Scale; Pcom, posterior communicating artery; RPO, remote proximal occlusion; Tx, treatment; VBJ, vertebrobasilar junction.
[a]For untreated patients, mRS was obtained at 3 mo after the initial diagnosis.

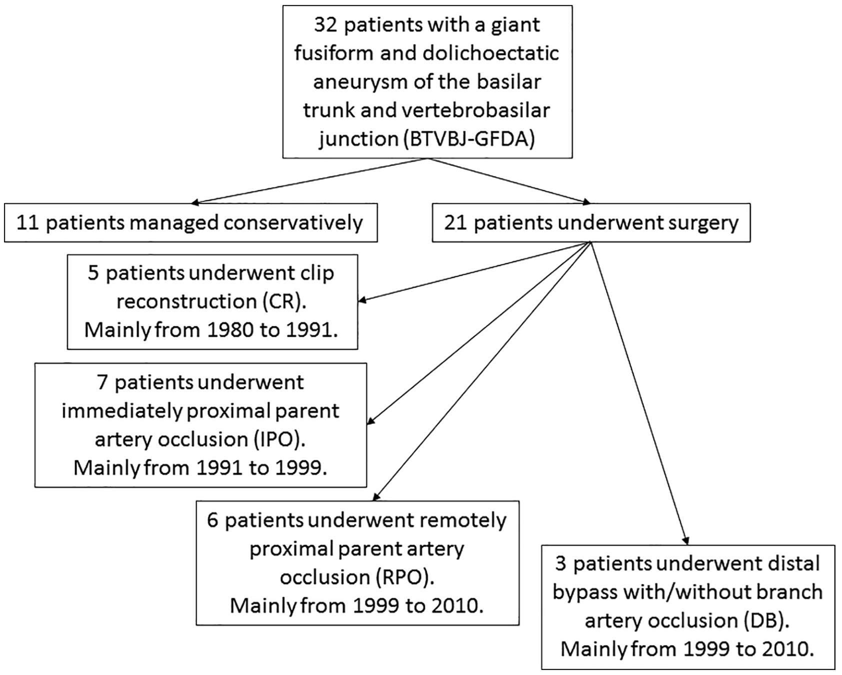


**FIGURE 2.** *Patient flowchart of included patients.*

follow-up period ranged from 1 d to 15 yr (mean 45.2 mo). Aneurysms were located in the proximal BT in 18 cases and at the VBJ in 14 cases. A total of 29 patients had a giant aneurysm and 3 had a large aneurysm as the mean size was 27.6 mm. All cases were symptomatic: ischemic strokes in 10 cases, subarachnoid hemorrhage (SAH) in 2, and brainstem compression in 20. A patient flow chart is demonstrated in Figure 2.

Downloaded from https://academic.oup.com/neurosurgery/article-abstract/doi/10.1093/neuros/nyaa317/5880069 by guest on 06 August 2020

## Conservative Treatment vs Surgical Treatment

Of the 32 patients, 11 underwent conservative medical treatment without any surgical intervention. Of these 11 patients, 10 died; 3 from progressive brainstem dysfunction caused by continued growth of the aneurysm (mean at 12 mo from initial presentation), 4 from SAH (mean at 21.6 mo), 2 from brainstem infarction (mean at 68 mo), and 1 from acute renal failure at 6 mo. One patient remained moderately disabled for 5 mo and was lost to follow-up. The overall morbidity and mortality rates of conservative treatment were 90.9% and 90.9%, respectively.

Surgical treatments were performed in 21 patients. In the early postoperative period, morbidity, and mortality rates were 47.6% and 14.3%, respectively. At the last follow-up (mean 56.6 mo), the overall morbidity and mortality rates of all surgical interventions were 71.4% and 57.1%, respectively. Multivariate logistic regression analysis showed that patients with surgical intervention had significantly longer overall survival than those conservatively managed (adjusted hazard ratio 1.508, 95% CI 1.058-2.148, $P = .02$). Kaplan-Meier analyses showed that the median survival time was 72.0 mo (range 0.0-151.9 months) for patients who underwent surgery, which was significantly longer than that of 9.0 mo (range 3.2-14.8 mo) for patients with conservative management (Figure 3A).

## Difference of Clinical and Radiological Characteristics and Outcomes by Age

We performed a subgroup analysis divided by age, younger patient group (younger than 45 yr of age, n = 5, mean age 26.2 yr) and elderly patient group (equal or older than 45 yr, n = 27, mean age 62.5 yr) (Table 2). Kaplan-Meier plots and the log-rank test showed that the median survival time of the elderly group was 11.0 mo (95% CI 0.00-23.25 mo), whereas that of the younger group was unreached ($P = .03$; Figure 3B). Albeit without statistical significance, the younger group tended to have favorable hemodynamic collaterals from the bilateral Pcoms than the elderly group (40% vs 11.1%, $P = .073$). A total of 4 of the 5 patients in the younger group were free from any atherosclerosis risk factors or diseases, whereas 24 of the 27 patients in the elderly group had at least one of them (88.9% vs 20%, $P = .004$).

## Treatment Options and Outcomes

The patient demographics divided by the treatment types are summarized in Table 3. Kaplan-Meier analyses by the surgical treatments are shown in Figure 3C. Median survival time showed no significant difference between the treatment types. Treatment selection evolved with our accumulated experience: CR was mainly performed during the initial period from 1980 to 1991, IPO was primarily selected from 1991 to 1999, RPO from 1999 to 2010, and DB was exclusively selected from 2010 to 2015.

Staged IPO was performed in 7 patients. A total of 3000 international units (IU) of heparin was administered intraoperatively. Postoperatively, continuous intravenous administration of 10 000 IU of heparin per day was maintained for 2 wk and then gradually

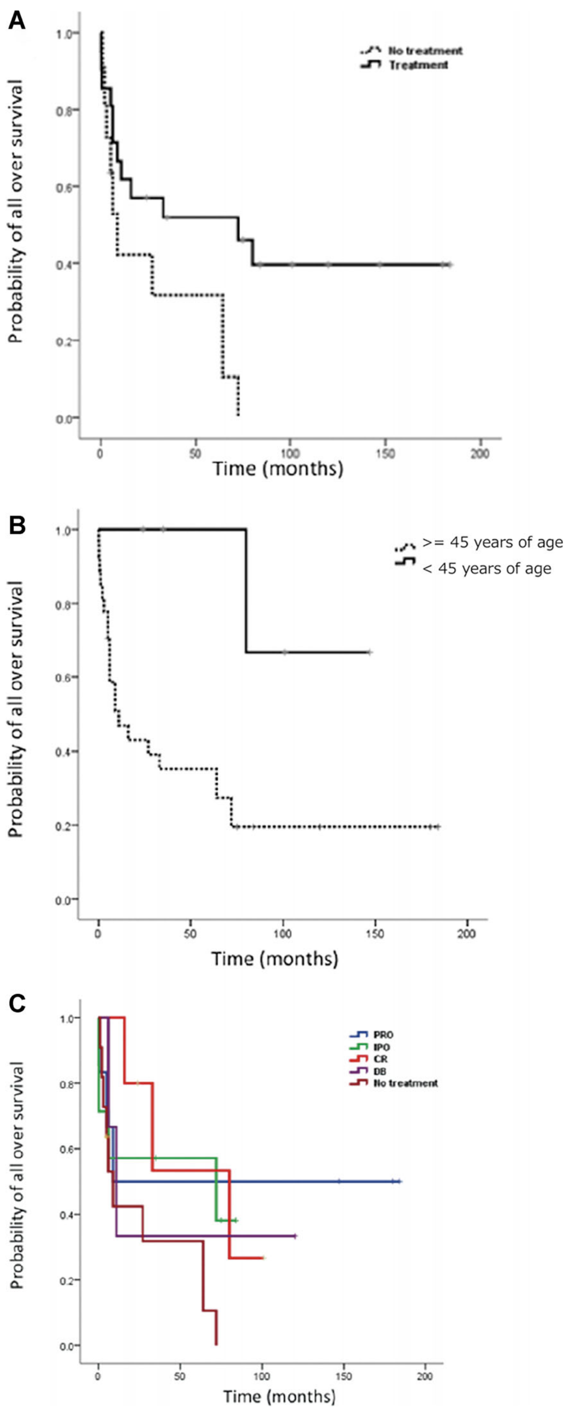


**FIGURE 3.** **A**, *Kaplan-Meier estimation of overall survival according to conservative vs surgical treatment in patients with giant fusiform and dolichoectatic aneurysms of the basilar trunk and vertebrobasilar junction (BTVBJ-GFDA).* **B**, *Kaplan-Meier estimation of overall survival according to age group, younger than 45 yr of age and older in patients with BTVBJ-GFDA.* **C**, *Kaplan-Meier estimation of survival according to surgical treatment types in patients with BTVBJ-GFDA.*

Downloaded from https://academic.oup.com/neurosurgery/article-abstract/doi/10.1093/neuros/nyaa317/5880069 by guest on 06 August 2020

**TABLE 2. Comparison of Patient Characteristics and Outcomes by Age**

| Variable | <45 yr | ≥45 yr | *P* value |
|---|---|---|---|
| Number of patients | 5 | 27 | |
| Sex, male: female | 2:3 | 18:9 | .26 |
| Treatment, treatment: no treatment | 5: 0 | 16: 11 | .14 |
| Location, BAT: VBJ | 1: 4 | 17: 10 | .14 |
| An size (mm), mean ± SD | 28.00 ± 7.58 | 27.56 ± 5.68 | .88 |
| Atherosclerosis risk, no: yes | 4:1 | 3: 24 | .004 |
| Bilateral Pcom patency, favorable: poor | 2: 3 | 3: 24 | .073 |
| Preoperative mRS, 1: 2: 3: 4: 5 | 0: 0: 3: 2: 0 | 4: 2: 7: 7: 7 | .37 |
| Tx category, IRO: RPO: CR: DB: No Tx | 1: 1: 3: 0: 0 | 6: 5 2: 3: 11 | .039 |
| Construction of bypass, no: yes | 4: 1 | 16: 11 | .63 |
| Postoperative mRS[a], 1: 2: 3: 4: 5: 6 | 2: 0: 1: 1: 1: 0 | 0: 3: 5: 7: 8: 4 | .031 |
| Latest follow-up mRS, 1: 2: 3: 4: 5: 6 | 3: 0: 0: 1: 0: 1 | 2: 1: 1: 0: 2: 21 | .008 |
| Follow-up from latest Tx (mo), mean ± SD | 77.40 ± 50.14 | 39.25 ± 52.93 | .15 |

An indicates aneurysm; BAT, basilar trunk; CR, clip reconstruction; DB, distal bypass; IPO, immediate proximal occlusion; mRS, modified Rankin Scale; Pcom, posterior communicating artery; RPO, remote proximal occlusion; Tx, treatment; VBJ, vertebrobasilar junction.
[a]For untreated patients, mRS was obtained at 3 mo after the initial diagnosis.

**TABLE 3. Comparison of Clinical Demographics by Treatment Type**

| | Treatment | | | | |
|---|---|---|---|---|---|
| Variable | IPO | RPO | CR | DB | *P* value |
| Number of patients | 7 | 6 | 5 | 3 | |
| Sex, female: male | 2: 5 | 2: 4 | 3: 2 | 2: 1 | .55 |
| Age, (years), ≥45 yr: < 45 yr | 6: 1 | 5: 1 | 2: 3 | 3: 0 | .17 |
| Location, BAT: VBJ | 3: 4 | 3: 3 | 2: 3 | 3: 0 | .35 |
| An size (mm), mean ± SD | 28.29 ± 7.89 | 29.67 ± 6.98 | 27.00 ± 6.71 | 27.67 ± 2.52 | .93 |
| Atherosclerosis risk, no: yes | 2: 5 | 1: 5 | 2: 3 | 0: 3 | .59 |
| Bilateral Pcom patency, favorable: poor | 1:6 | 2: 4 | 2:3 | 0:3 | .51 |
| Preoperative mRS, 1: 2: 3: 4: 5 | 2: 0: 2: 2: 1 | 2: 0: 2: 1: 1 | 0: 0: 2: 2: 1 | 0: 1: 1: 1: 0 | .64 |
| Construction of bypass, no: yes | 1: 6 | 3: 3 | 5: 0 | 0: 3 | .010 |
| Postoperative mRS, 1: 2: 3: 4: 5: 6 | 1: 0: 0: 2: 2: 2 | 1: 2: 1: 1: 1: 0 | 0: 0: 1: 2: 2: 0 | 0: 0: 1: 1: 0: 1 | .56 |
| Postoperative angiographic findings | AD6, PBR6 | AD4, CD5, PBR3 | AD5 | AD0, PB3 | |
| Latest follow-up mRS, 1: 2: 3: 4: 5: 6 | 1[a]: 0: 0: 0: 2: 4 | 3[b]: 0: 0: 0: 0: 3 | 1[a]: 0: 0: 1: 0: 3 | 0: 0: 1: 0: 0: 2 | .19 |
| Follow-up from latest Tx (mo), mean ± SD | 38.90 ± 37.72 | 87.58 ± 91.59 | 50.80 ± 37.48 | 45.67 ± 60.37 | .54 |

AD, aneurysm disappeared; An, aneurysm; BAT, basilar artery trunk; CD, collateral development via either external carotid artery branches or vertebral artery muscular branches; CR, clip reconstruction; DB, distal bypass; IPO, immediately proximal occlusion; mRS, modified Rankin Scale; PB, patent bypass; PBR, patent bypass retrograde filling to distal basilar artery; Pcom, posterior communicating artery; RPO, remotely proximal occlusion; Tx, treatment; VBJ, vertebrobasilar junction.
[a]One patient with IPO and one patient with CR attained mRS 1 at the latest follow-up, both aged younger than 45 yr (20 and 27).
[b]Three patients with RPO attained mRS 1 at the latest follow-up, 2 patients aged older than 45 yr (51and 57) and 1 aged 21 yr.

switched to oral antiplatelet therapy with aspirin 100 mg daily or cilostazol 100 mg twice per day over a week, which was maintained for 1 to 3 mo. Two patients attained the same mRS in the early postoperative period as preoperatively (Figure 4A-4D). In contrast, 5 patients suffered devastating progressive brainstem infarction (Figures 5A-5D and 6A and 6B). Two of these 5 patients died. One patient with BT aneurysm with favorable Pcom collateral flows ended up with unilateral occlusion because of patient refusal. This aneurysm did not disappear at postoperative angiogram (Table 3). The early postoperative morbidity and mortality rates were 71.4% and 28.6%, respectively. At the last follow-up (mean 38.90 mo), 1 patient remained in mRS 1, 2 in mRS 5, and 4 had died (mRS 6). The overall morbidity and mortality rates of IPO were 85.7% and 57.1%, respectively.

Staged RPO, either surgical or endovascular, was performed in 6 patients (Figures 7 and 8, and **Video**, **Supplemental Digital**

Downloaded from https://academic.oup.com/neurosurgery/article-abstract/doi/10.1093/neuros/nyaa317/5880069 by guest on 06 August 2020

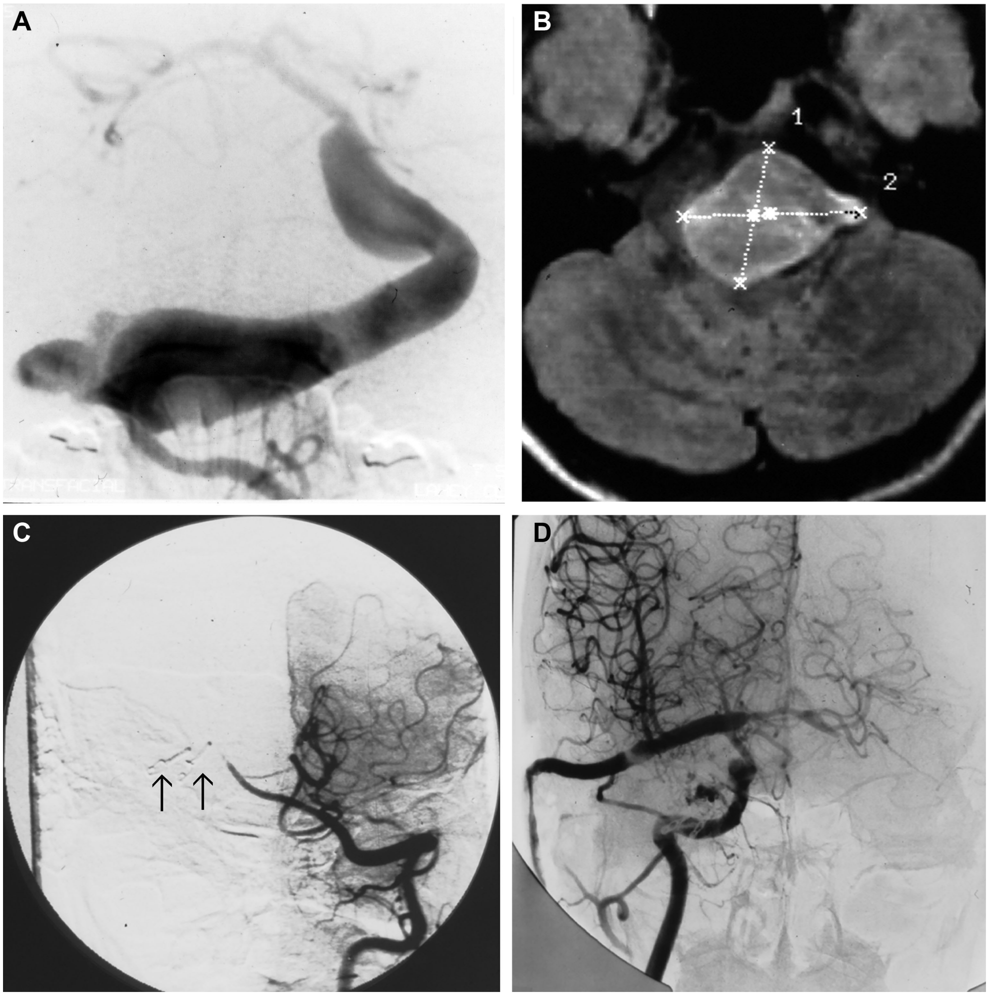


**FIGURE 4.** *Imaging studies of a 20-yr-old woman with a giant/large fusiform and dolichoectatic aneurysms of the basilar trunk and vertebrobasilar junction (BTVBJ-GFDA) presenting with brainstem compression syndrome. She underwent immediate proximal parent artery occlusion (IPO) treatment with high-flow saphenous vein graft bypass from the external carotid artery (ECA) to the posterior cerebral artery (PCA).* **A**, *Preoperative left vertebral angiogram demonstrated BTVBJ-GFDA.* **B**, *Preoperative T1-weighted MR image demonstrated significant brainstem compression by the aneurysm.* **C**, *Postoperative left vertebral artery angiograms showed disappearance of the BTVBJ-GFDA. The arrows are pointing at clips used for IPO.* **D**, *Postoperative right common carotid artery angiogram demonstrated patent ECA to PCA bypass.*

**Content**). Anticoagulation treatment was administered intraoperatively, maintained for 7 d, and then tapered gradually for each vertebral artery occlusion. After the bypass construction, antiplatelet therapy with prostaglandin E1 infusion was initiated immediately, and this was switched to oral cilostazol on postoperative day 7, and then continued for 1 mo. Four patients attained the same mRS as the preoperative status (Figure 7). Among other 2 patients, one patient initially presented with deterioration of consciousness due to pontine hemorrhage, and remained in the vegetative state postoperatively. The other patient suffered temporary deterioration due to brainstem ischemia (mRS 3 immediately after surgery), then recovered to mRS1 at the last follow-up at 180 mo (Figure 8). The aforementioned 4 patients without postoperative neurological decline all showed remarkable aneurysm shrinkage over the follow-up period (Figure 9). Of the 6 patients, 5 developed collateral flow from either or both of the vertebral artery muscular branches and the external carotid artery branches into the basilar artery (Figure 9C). Two patients with BT aneurysm ended up with unilateral occlusion due to severe clinical condition and patient refusal, respectively. These 2 aneurysms did not disappear (Table 3). The early postoperative morbidity and mortality rates of RPO were 33% and 0%, respectively. At the



Downloaded from https://academic.oup.com/neurosurgery/article-abstract/doi/10.1093/neuros/nyaa317/5880069 by guest on 06 August 2020

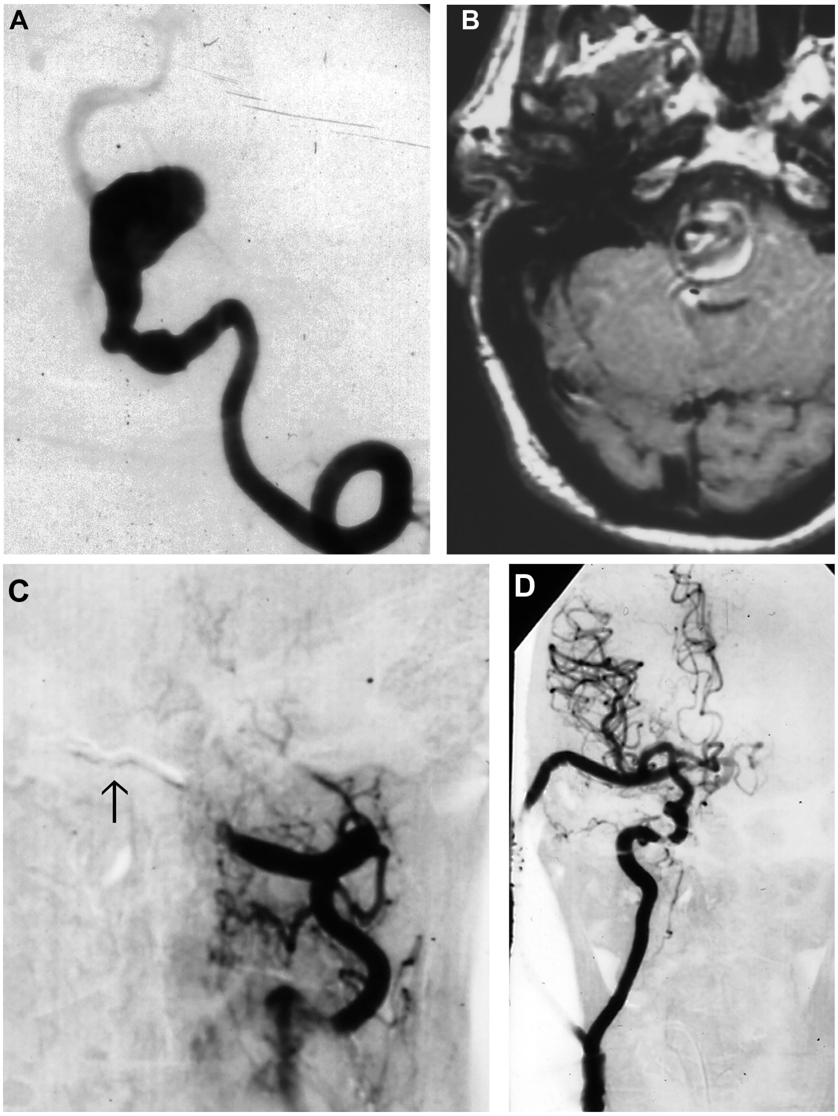


**FIGURE 5.** *Imaging studies of another case of IPO. A 63-yr-old man presented with brainstem compression syndrome caused by BTVBJ-GFDA. He underwent the IPO with high-flow bypass (ECA-saphenous vein graft-PCA).* **A**, *Preoperative left vertebral angiogram revealing BTVBJ-GFDA.* **B**, *Preoperative T1-weighted MR image demonstrated significant brainstem compression by the aneurysm.* **C**, *Postoperative left vertebral artery angiograms showed disappearance of the BTVBJ-GFDA.* **D**, *An arrow is pointing at clips used for IPO. Postoperative right common carotid artery angiogram demonstrated patent ECA to PCA bypass. ECA, external carotid artery; PCA, posterior cerebral artery.*

last follow-up (mean 87.58 mo), 3 patients achieved mRS 1, and 2 died of undetermined causes. Overall morbidity and mortality rates were 50% and 50%, respectively.

CR was performed in 5 patients. Immediately after surgery, 2 patients attained the same mRS as the preoperative condition, and 3 developed severe brainstem infarction. The early postoperative morbidity and mortality rates were 60% and 0%, respectively. At the last follow-up (mean 50.80 mo), 3 patients died after 16, 33, and 80 mo. One patient attained good neurological recovery to mRS 1 over 3 yr. The morbidity and mortality rates at the last follow-up were 80% and 60%, respectively.

DB was performed in 3 patients. Both anticoagulation and antiplatelet treatments were administered as the same protocol as RPO. Immediately after surgery, 2 of the 3 patients attained the

Downloaded from https://academic.oup.com/neurosurgery/article-abstract/doi/10.1093/neuros/nyaa317/5880069 by guest on 06 August 2020

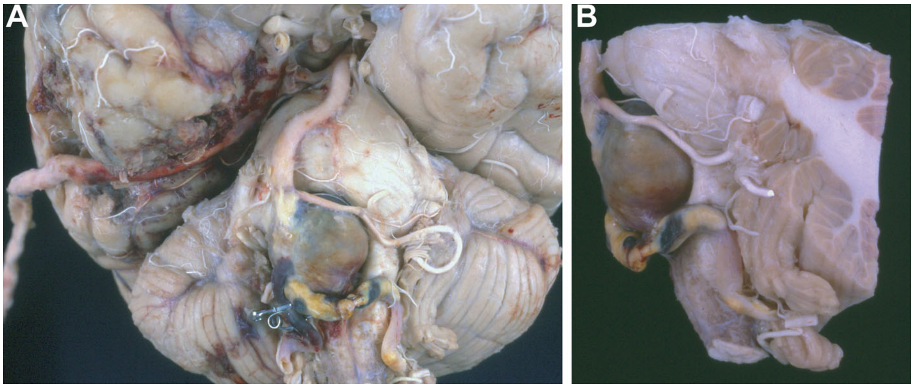


**FIGURE 6.** *Postmortem examination photographs of a case of Figure 3 (IPO and high-flow bypass). Although the bypass was patent in the examination* **A**, *massive brainstem infarction was also noted* **B**.

same mRS as the preoperative status. One patient who underwent high-flow bypass developed fatal SAH on the day after surgery and died. The early postoperative morbidity and mortality rates were 33.3% and 33.3%, respectively. One patient attained mRS 3 at 120 mo, whereas one patient who underwent DB with remote unilateral vertebral artery occlusion subsequently developed SAH and died 6 mo later. At the latest follow-up (mean 45.67 mo), morbidity and mortality rates were 66.7% and 66.7%, respectively. Both 2 patients who died after DB were older than 45 yr and did not have favorable Pcom flow collaterals but did have atherosclerotic risk factors. All 5 patients who had favorable Pcom collateral flows underwent surgery (RPO in 2, IPO in 1, and DB in 2) but did not require bypass construction. Contrarily, 12 of 17 patients who underwent surgery with poor Pcom collaterals (70.6%) required bypass construction.

Transition of mRS in all patients is demonstrated in Figure 10A, whereas that of 21 surgically treated patients in Figures 10B, and 11 conservatively managed patients in Figure 10C.

### Pathological Findings

Histological examination of 2 aneurysms obtained from 2 patients (case 1: Figures 5 and 6; case 2: Figure 11A-11C) demonstrated prominent neovascularization in both the thickened intima and intrathrombotic organized staged clots. The normal intracranial arterial wall contained no neovascularization or thickened intima and intrathrombotic organized staged clots (Figure 11D and 11E). The perforating vessels located in the adjacent brainstem parenchyma and the recanalizing vessels within the thickened adventitia (vasa vasorum) were aligned continuously, apparently maintaining their patency (Figure 11F and 11G). This patent lumen was further supported by the factor VIII-positive endothelial lining, without occluding organized clots.

## DISCUSSION

### Factors Affecting the Natural History of BTVBJ-GFDA

A major problem in treating BTVBJ-GFDA is that the lesion often extends to the entire circumference of the vessel wall and involves vital perforating arteries arising from the affected vessel.[13] Occlusion of the aneurysm by clipping or coils carries a high risk of sacrificing those critical perforating vessels. Nevertheless, these aneurysms have been reported of an extremely poor natural history,[6-11] indeed, as demonstrated in our series with a median survival of 9 mo. Of our 11 patients, 10 patients without surgical treatment died. Therefore, any procedures which could improve this otherwise devastating natural prognosis should be considered. Both hemorrhage caused by excess of blood flow and ischemia due to poor blood flow can occur and be fatal, so surgical treatment of BTVBJ-GFDA should achieve adequate hemodynamic status by targeting the narrow treatment window. Two procedures constitute the core keys to achieve this delicate goal, occlusion of the parent arteries and bypass construction. Appropriate combination of these 2 procedures to match the specific hemodynamic status in individuals should provide the best chance of improving the poor clinical course of this disease. In our series, 4 types of surgical treatments were performed in 21 cases, achieving the median survival of 72 mo. Our study suggests that surgical treatment is an essential factor to reverse the dismal prognosis.

### Surgical Treatment vs Conservative Management

Admittedly, selection bias regarding decision making toward surgery cannot be neglected in our limited cohort. As shown in Table 1, those patients who underwent surgery tended to be younger and to present with less severe condition (better mRS) than those managed conservatively ($P = .078$ and 0.10, respectively). Our present study was unable to demonstrate superiority

Downloaded from https://academic.oup.com/neurosurgery/article-abstract/doi/10.1093/neuros/nyaa317/5880069 by guest on 06 August 2020

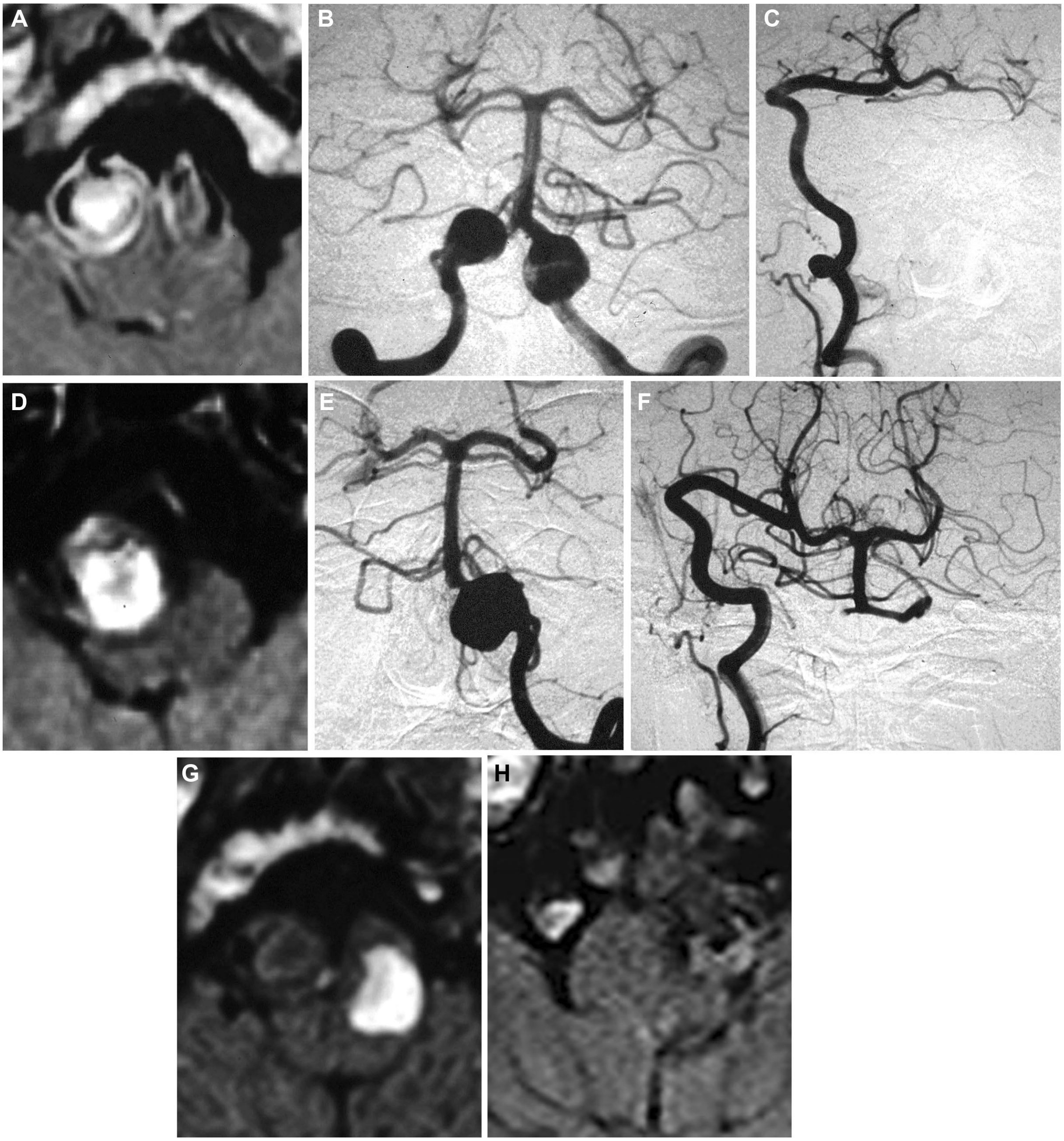


**FIGURE 7.** *Imaging studies of a case of remote proximal parent artery occlusion (RPO) with high-flow radial artery graft bypass from the extracranial vertebral artery (VA) to the posterior cerebral artery (PCA) (see* ***Video, Supplemental Digital Content****, first case). A 57-yr-old man presented with brainstem compression syndrome caused by bilateral large fusiform and dolichoectatic aneurysms of the vertebrobasilar junction (VBJ-BLFDA).* **A***, Preoperative T1-weighted MR image with gadolinium demonstrated significant brainstem compression by the aneurysm.* **B***, Preoperative bilateral vertebral angiogram revealing VBJ-BLFDA.* **C***, Postoperative angiogram showing disappearance of the right VA aneurysm and patent extracranial VA to PCA bypass.* **D***, Postoperative T1-weighted MR image showing complete thrombosis of the right VA aneurysm. Follow-up angiogram revealed slight enlargement of the left VA aneurysm* **E***, so was occluded with a balloon at the C1-2 level 9 mo after initial treatment* **F***.* **G***, Postendovascular occlusion MR image revealed successful left VA aneurysm thrombosis.* **H***, Recent T1-weighted MR image showed stable aneurysms on both sides at 184 mo after the treatment.*

Downloaded from https://academic.oup.com/neurosurgery/article-abstract/doi/10.1093/neuros/nyaa317/5880069 by guest on 06 August 2020

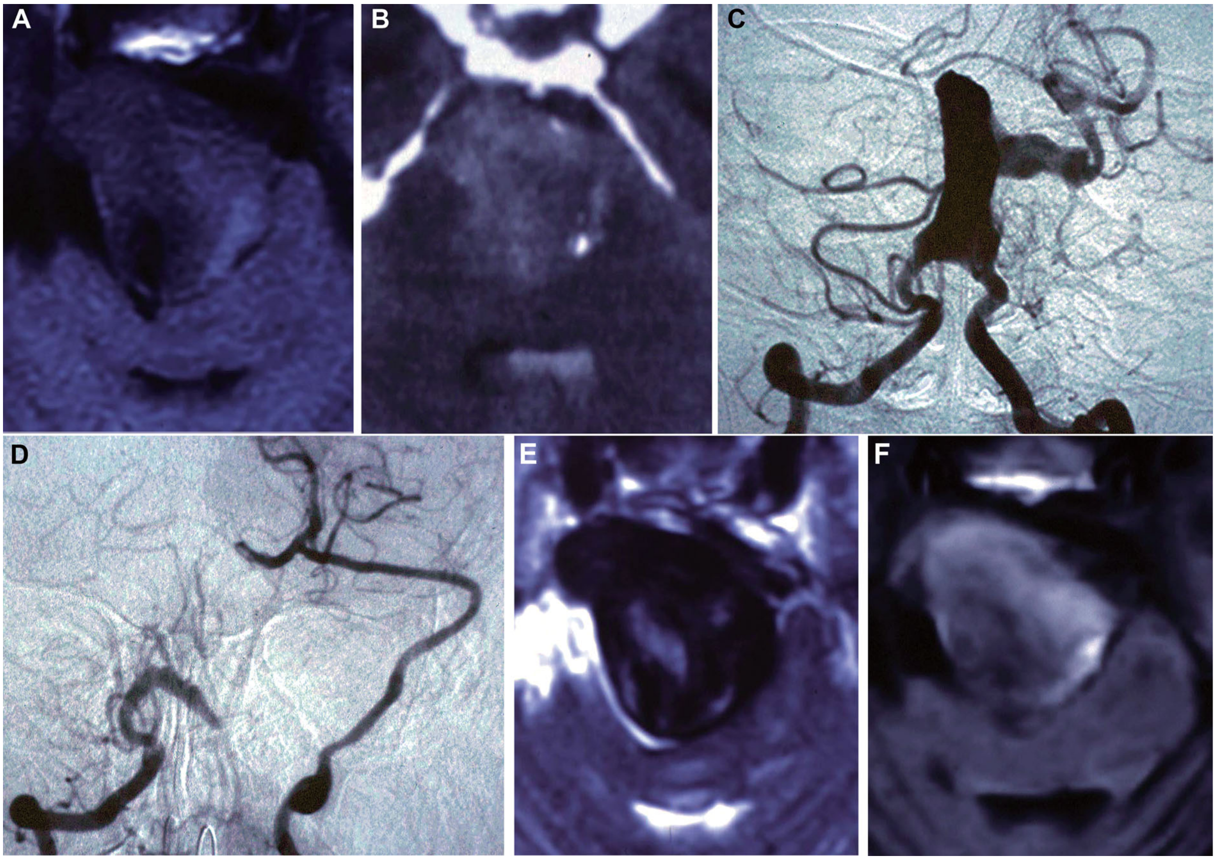


**FIGURE 8.** *Imaging studies of another case of staged remote proximal parent artery occlusion (RPO) and high-flow radial artery graft bypass from the extracranial VA to the posterior cerebral artery (PCA) (see **Video, Supplemental Digital Content**, second case). A 52-yr-old woman presented with subarachnoid hemorrhage (SAH) caused by rupture of a giant fusiform and dolichoectatic aneurysm of the basilar trunk (BT-GFDA).* **A**, *MR image taken 5 mo prior demonstrated a mid-basilar partially thrombosed giant fusiform aneurysm with small intramural hemorrhage.* **B**, *Computed tomography (CT) at admission demonstrated SAH with hematoma in the fourth ventricle.* **C**, *Cerebral angiogram showed a mid-basilar, partially thrombosed giant dolichoectatic aneurysm.* **D**, *Postoperative angiogram showed disappearance of the BT-GFDA and patent extracranial VA to PCA bypass.* **E**, *Postoperative T2-weighted MR image showed complete thrombosis of the aneurysm with slight pontine ischemia.* **F**, *Follow-up MR image 1 yr after the treatment revealed successful aneurysm thrombosis and shrinkage. She recovered well and no rebleeding occurred during the 180-mo follow-up period.*

of surgery over conservative management regarding neurological outcome despite this potential patient characteristics' advantages, which could emphasize the complexity of the disease: only 5 patients among 21 patients who underwent surgery (23.8%) achieved mRS 1.

## Parent Artery Occlusion Immediately Proximal or Remotely Proximal to the Aneurysm

Only 1 patient among 6 who underwent RPO developed brainstem infarction and suffered early postoperative neurological deterioration, but recovered remarkably at the last follow-up. The other 5 patients tolerated the procedure well with early morbidity in 1 patient. In contrast, 5 of the 7 patients treated with IPO suffered brainstem infarction, and 4 of the 7 patients died. From our limited case series, RPO seemed more favorable than IPO. Our hypothesis is because of the dramatic pathological change from dolichoectasia, manipulation or occlusion just proximal to the lesion carries a risk of occluding the nearby brainstem perforators. Exceptionally, one patient who underwent IPO of bilateral vertebral arteries had good outcome; however, this was a young patient (20-yr-old female) with no atherosclerotic risk and had large unilateral Pcom: this patient was in extremely fortunate condition. IPO might be more likely to be tolerated if good collateral circulation can be expected after parental artery occlusion.

## Two Types of Collateral Blood Flow: Pcom and Perforating Vessels

Close histological examination of the autopsy cases showed rich microvascular networks on the surfaces of the aneurysms,

Downloaded from https://academic.oup.com/neurosurgery/article-abstract/doi/10.1093/neuros/nyaa317/5880069 by guest on 06 August 2020

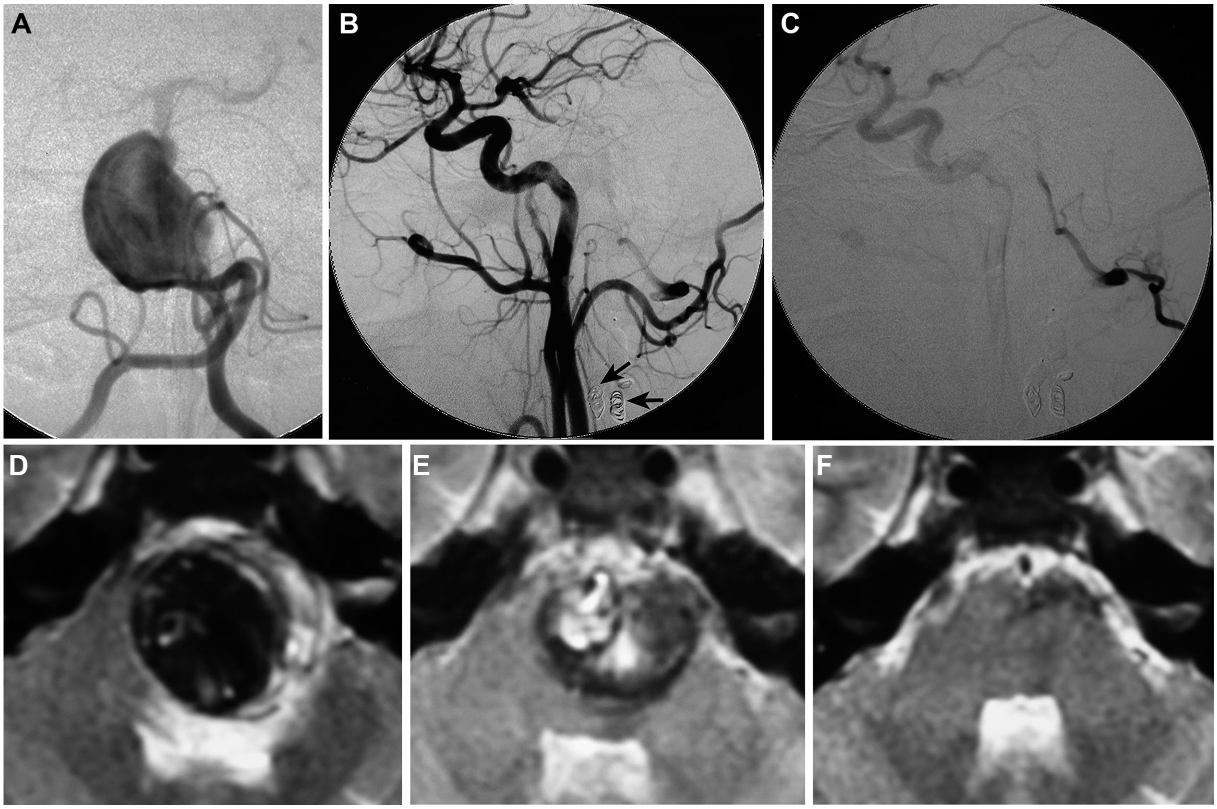


**FIGURE 9.** *Imaging studies of another case of remote proximal parent artery occlusion (RPO) (endovascular coil embolization of bilateral vertebral arteries) without bypass. A 23-yr-old man presented with brainstem compression syndrome caused by giant fusiform and dolichoectatic aneurysms of the basilar trunk (BT-GFDA).* **A**, *Preoperative angiogram demonstrated BT-GFDA.* **B**, *Postoperative right carotid artery angiogram demonstrated sufficient collateral flow from the right posterior communicating artery (Pcom). Coils are shown in arrows.* **C**, *Evolution of collateral flow from the right occipital artery to the vertebral artery as well as to the mid-basilar artery through the muscular branches was noted in the postoperative angiogram. T2-weighted MR images 24 d* **D**, *3 mo* **E**, *and 12 mo* **F** *after RPO showed consecutive shrinkage and disappearance of the BT-GFDA. The patient has remained free from neurological deficits for 147 mo.*

which apparently were connected to the vessels in the brainstem, as we previously reported.[16] Such vessels may form collateral circulation at another level besides Pcom, namely perforating vessel collateral circulation (PVCC). The poor outcomes after IPO may have been caused by injuring PVCC. Indeed, a recent study of surgical outcome in dolichoectatic BT aneurysms in 37 patients concluded that good outcomes are determined by perforator preservation and mitigating aneurysm thrombosis.[16] However, evaluation of PVCC requires indirect parameters because direct evaluation is very guarded with currently available imaging studies. Given that atherosclerotic small artery disease is probably the most prominent risk for perforating vessel occlusion, atherosclerosis risk factors can be a reasonable parameter for evaluation of PVCC. In our series, 24 of the 27 patients in the elderly group had some atherosclerosis risks (88.9%) and, indeed, less satisfactory survival. Contrarily, only one patient in the younger patient group had atherosclerosis risk factors (20%, $P = .004$). Furthermore, there was a tendency that younger patients had more favorable Pcom collateral flows ($P = .073$). This suggests younger patients with healthier vasculature and arterial plasticity, and good Pcom collateral flows that enable the circle of Willis to habituate to new established hemodynamic flow out of BTVBJ-GFDA could enjoy good outcome. Additionally, both 2 patients who died after DB were older than 45 yr of age and did not have favorable Pcom collateral flows but did have atherosclerotic risk factors, all of which could contribute to lack of arterial plasticity. This can potentially guide surgical treatment options: in younger patients with good Pcom collateral flow and without atherosclerosis risk factors, IPO can be selected if technically more approachable. On the other hand, in elderly patients or patients with multiple atherosclerosis risk factors, RPO might be a safer option to minimize risk of perforator injury, as insufficient arterial plasticity is suggested. Furthermore, requirement of bypass construction should also be considered in patients with

Downloaded from https://academic.oup.com/neurosurgery/article-abstract/doi/10.1093/neuros/nyaa317/5880069 by guest on 06 August 2020

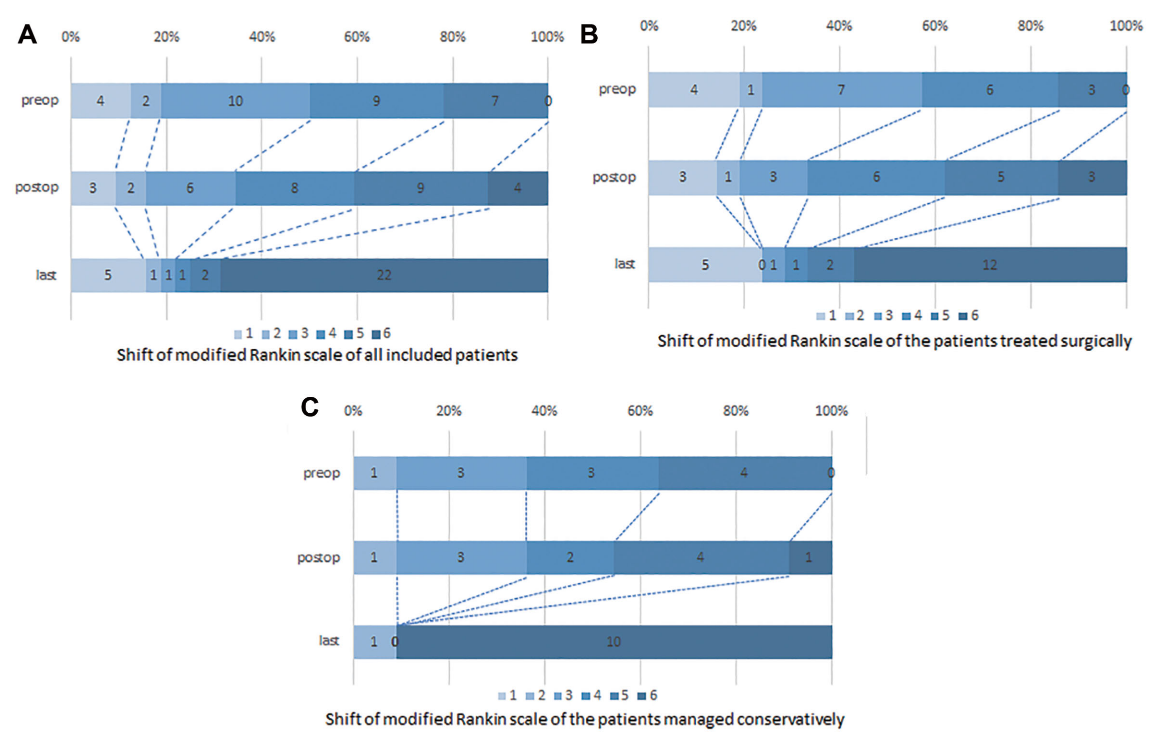


**FIGURE 10.** *Shifts of modified Rankin Scale (mRS) of included patients (***A***, all included patients,* **B***, surgically treated patients, and* **C***, conservatively managed patients).*

poor Pcom collateral flows, as in our cohort, 70.6% of those patients required bypass construction in surgery.

## Limitations

One limitation of this study is scarcity of treatment options. Recently, flow diverter treatment for vertebrobasilar fusiform aneurysms has been performed,[12-14] which can be another treatment option. One major problem regarding flow diverter treatment in BTVBJ-GFDA is lack of long-term follow-up. Recent published meta-analysis in 2018 included 13 studies worldwide; however, the longest mean follow-up period among these studies was 25.2 mo. In their meta-analysis, small size (≤10 mm) was the prognostic factor for good neurological outcome, and avoidance of perforator injury was reported as a key for achieving good neurological outcome.[14] Patients with a relatively small fusiform/dolichoectatic aneurysm might benefit from flow diverter treatment. However, the concern remains as we try to save the perforators, because this inevitably saves the vasa vasorum as well, which is also believed to be a contributing factor for aneurysm growth. Large fusiform/dolichoectatic aneurysms with severe symptomatology, on the other hand, may require thoughtful consideration of more drastic approach including surgery, which comes with a risk with high mortality rate (57.1%). Second, we cannot exclude selection bias in our limited cohorts: 10 of the 11 patients who were managed conservatively presented with moderate/severe disability (mRS ≥ 3), and we might have included more severe patients eccentrically than those with benign symptomatology (eg, incidentally found BTVBJ-GFDA). Furthermore, functional outcome of surgery in our limited cohort was still dismal, with just 28.1% achieving mRS 1. External validity should be carefully implemented from our study in decision-making process of intervention, especially when comparing to the natural history of BTVBJ-GFDA. One possible explanation is our series included already grown BTVBJ-GFDA, because the inclusion criteria was ≥15 mm, as Mangrum et al[17] demonstrated that mortality rate of patients who have grown vertebrobasilar fusiform or dolichoectatic aneurysms was 5.7 times higher than those without aneurysm growth. Third, because of the long study period, and severe conditions in many patients, access to the follow-up imaging studies was very limited. Indeed, 4 patients in 22 surgically treated patients (18.2%) did not undergo MRI because they died very soon after surgery (1-15 d). Likewise, long-term follow-up MRI was accessible in only 3 patients (Figures 5-7). Further case accumulation and sharing of treatment experience is crucial for investigation of this validity.

## CONCLUSION

BTVBJ-GFDA has a devastating natural history, but surgery can offer longer survival. Patients younger than 45 yr of age



Downloaded from https://academic.oup.com/neurosurgery/article-abstract/doi/10.1093/neuros/nyaa317/5880069 by guest on 06 August 2020

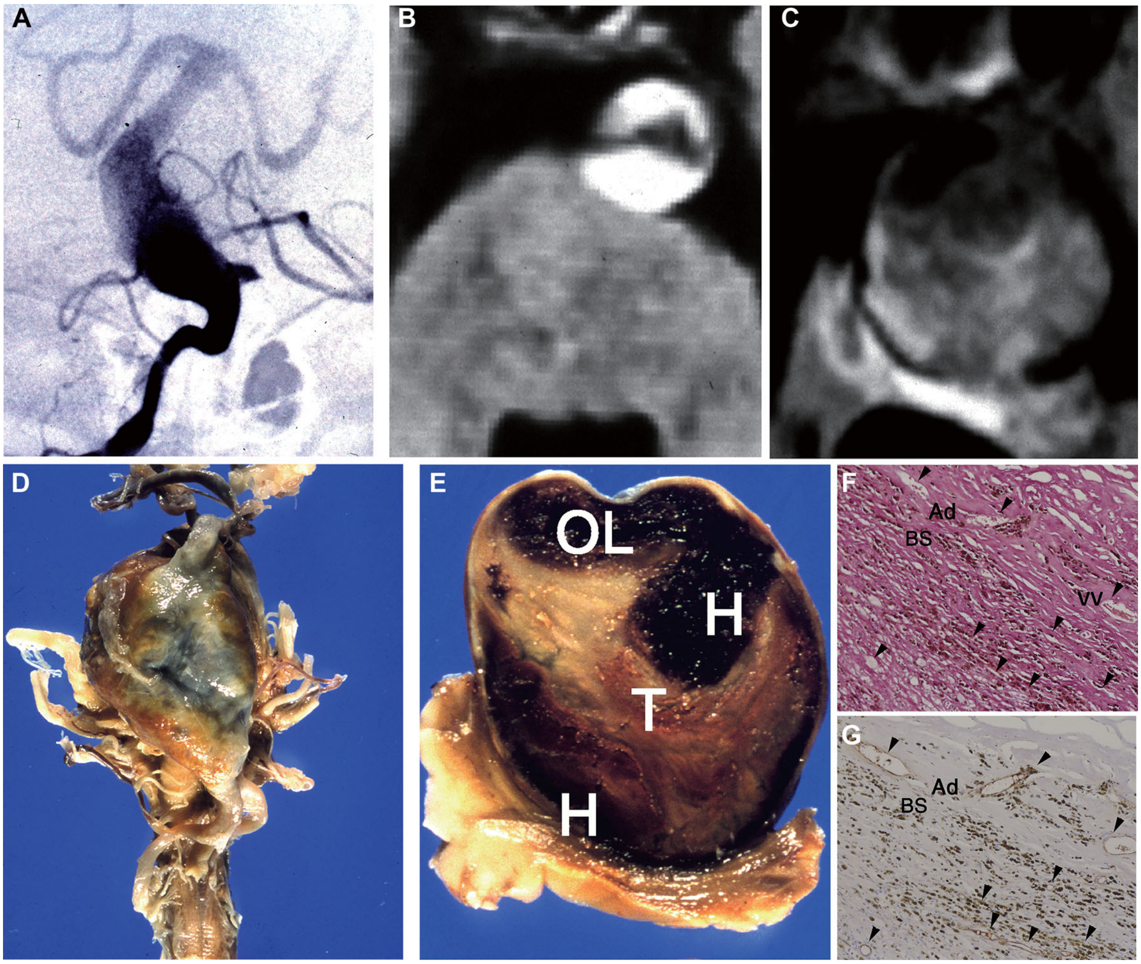


**FIGURE 11.** *A mortality case with conservative management. A 66-yr-old man presented with "locked-in" syndrome caused by a large fusiform aneurysm of the basilar trunk.* **A**, *Right vertebral artery angiogram demonstrating dolichoectatic basilar artery. Bilateral anterior inferior cerebellar arteries are coming off from this lesion.* **B**, *Magnetic resonance angiogram (MRA) demonstrated a tortuous, enlarged basilar artery with a diameter of 1.5 cm with intramural hemorrhage within the aneurysm wall.* **C**, *Follow-up MR image 4 yr later revealed progressive aneurysm growth exceeding 4 cm.* **D**, *Five years later, he suddenly developed hypotensive shock and deceased. Postmortem examination revealed a giant aneurysm arising from the middle one-third of the basilar artery.* **E**, *Coronal section of the aneurysm showed an open lumen (OL), flap-like tissue (arrow) containing numerous vascular channels, staged laminated thrombus (T), and thick aneurysm wall. The staged thrombus contains hemorrhage (H) and new clots.* **F** *and* **G**, *Pathological specimen with hematoxylin and eosin stain* **F** *and factor VIII stain* **G** *with original magnification x40. Perforating vessels located in the surrounding brainstem parenchyma (BS) and recanalizing vessels (VV, vasa vasorum) within the thickened adventitia (Ad) are aligned continuously, apparently maintaining patency (arrowheads), and lined with a layer of endothelial cells positively stained with antibody for factor VIII-related antigen.*

enjoyed longer survival in our limited series. In surgery, a narrow treatment window of hemodynamic conditions within the aneurysm to maintain sufficient but not excess blood supply should be targeted based on the hemodynamics of both the Pcoms and perforating vessel collaterals.

## Disclosures

This work was supported by a Ministry of Health, Labor and Welfare Scientific Research Grant on rare and intractable diseases to Dr Nakatomi (No. 2015-38) from the Ministry of Health, Labor and Welfare of Japan. The authors have no personal, financial, or institutional interest in any of the drugs, materials, or devices described in this article.

## REFERENCES

1. Resta M, Gentile MA, Di Cuonzo F, Vinjau E, Brindicci D, Carella A. Clinical-angiographic correlations in 132 patients with megadolichovertebrobasilar anomaly. *Neuroradiology*. 1984;26(3):213-216.

Downloaded from https://academic.oup.com/neurosurgery/article-abstract/doi/10.1093/neuros/nyaa317/5880069 by guest on 06 August 2020

2. Yu YL, Moseley IF, Pullicino P, McDonald WI. The clinical picture of ectasia of the intracerebral arteries. *J Neurol Neurosurg Psychiatry*. 1982;45(1):29-36.
3. Pico F, Labreuche J, Amarenco P. Pathophysiology, presentation, prognosis, and management of intracranial arterial dolichoectasia. *Lancet Neurol*. 2015;14(8):833-845.
4. Nakatomi H, Segawa H, Kurata A, et al. Clinicopathological study of intracranial fusiform and dolichoectatic aneurysms: insight on the mechanism of growth. *Stroke*. 2000;31(4):896-900.
5. Ono H, Nakatomi H, Tsutsumi K, et al. Symptomatic recurrence of intracranial arterial dissections: follow-up study of 143 consecutive cases and pathological investigation. *Stroke*. 2013;44(1):126-131.
6. Anson JA, Lawton MT, Spetzler RF. Characteristics and surgical treatment of dolichoectatic and fusiform aneurysms. *J Neurosurg*. 1996;84(2):185-193.
7. Drake CG, Peerless SJ. Giant fusiform intracranial aneurysms: review of 120 patients treated surgically from 1965 to 1992. *J Neurosurg*. 1997;87(2):141-162.
8. Flemming KD, Wiebers DO, Brown RD Jr, et al. The natural history of radiographically defined vertebrobasilar nonsaccular intracranial aneurysms. *Cerebrovasc Dis*. 2005;20(4):270-279.
9. Haddad GF, Haddad FS. Cerebral giant serpentine aneurysm: case report and review of the literature. *Neurosurgery*. 1988;23(1):92-97.
10. Nasr DM, Brinjikji W, Rouchaud A, Kadirvel R, Flemming KD, Kallmes DF. Imaging characteristics of growing and ruptured vertebrobasilar non-saccular and dolichoectatic aneurysms. *Stroke*. 2016;47(1):106-112.
11. Tomasello F, Albanese V, Cioffi FA. Giant serpentine aneurysms: a separate entity. *Surg Neurol*. 1979;12(5):429-432.
12. Cohen JE, Gomori JM, Moscovici S, Itshayek E. Successful endovascular treatment of a growing megadolichoectasic vertebrobasilar artery aneurysm by flow diversion using the "diverter-in-stent" technique. *J Clin Neurosci*. 2012;19(1):166-170.
13. Tan LA, Moftakhar R, Lopes DK. Treatment of a ruptured vertebrobasilar fusiform aneurysm using pipeline embolization device. *J Cerebrovasc Endovasc Neurosurg*. 2013;15(1):30-33.
14. Kiyofuji S, Graffeo CS, Perry A, et al. Meta-analysis of treatment outcomes of posterior circulation non-saccular aneurysms by flow diverters. *J Neurointerv Surg*. 2018;10(5):493-499.
15. Pico F, Labreuche J, Cohen A, Touboul PJ, Amarenco P, GENIC Investigators. Intracranial arterial dolichoectasia is associated with enlarged descending thoracic aorta. *Neurology*. 2004;63(11):2016-2021.
16. Lawton MT, Abla AA, Rutledge WC, et al. Bypass surgery for the treatment of dolichoectatic basilar trunk aneurysms: a work in progress. *Neurosurgery*. 2016;79(1):83-99.
17. Mangrum WI, Huston J 3rd, Link MJ, et al. Enlarging vertebrobasilar nonsaccular intracranial aneurysms: frequency, predictors, and clinical outcome of growth. *J Neurosurg*. 2005;102(1):72-79.

***Supplemental digital content** is available for this article at www.neurosurgery-online.com.*

**Supplemental Digital Content. Video.** (5 min, 357 MB) Surgical procedure of 2 cases of Figures 5 and 6: staged remote proximal parent artery occlusion (RPO) and high-flow radial artery graft bypass from the extracranial VA to the posterior cerebral artery (PCA).

## COMMENT

Giant fusiform and dolichoectatic aneurysms of the basilar trunk and vertebrobasilarjunction (BTVBJ-GFDA) are the 'glioblastomas of neurovascular surgery' often with very poor natural history. Diffuse anomaly of whole vertebrobasilar (VBA) system makes often the neurosurgeon unarmed - sudden spontaneous or induced changes of hemodynamics may cause the anomaly to rupture or thrombose leading to sudden death. This is a nice paper but conclusions drawn on these 21 selected cases should be cautious. As the authors have concluded even some blood flow through the dolichoectacy may be enough to extend the survival of the patients. This was achieved by modification of hemodynamics from the aneurysm toward the Pcoms - from posterior circulation to anterior circulation. The main message is that the outcome is highly patient-depended and related to vascular plasticity and tolerance to the newly established hemodynamic stress. Revascularization procedures via deep corridors in the eloquent posterior fossa are quite complex and risky, too. The best results are achieved when induced changes are slow enough to give possibilities for Pcoms and collaterals to grow in timely fashion without changing dramatically blood flow inside of the dolichoectasy and critical perforators to the brainstem. This may also be achieved in the not so far away future by a slowly closing 'Helsinki clip'.[1]

**Behnam Rezai Jahromi**
**Mika Niemelä**
*Helsinki, Finland*

1. Rezai Jahromi B, Hirvelä V, Sarpaneva S, et al. A device for applying external pressure on a surface of an anatomical object. 4/4/2019, "FI Patent PCT/FI2017/050,690" WO 2019/06 3871 Al.

Downloaded from https://academic.oup.com/neurosurgery/article-abstract/doi/10.1093/neuros/nyaa317/5880069 by guest on 06 August 2020
