# Supplementary material for: Management strategies and outcomes of basilar trunk aneurysms: a systematic review and meta-analysis
Source: Langenbecks Arch Surg. 2026 Jan 20;411(1):71. doi: 10.1007/s00423-025-03959-3 (PMC12852281; doi:10.1007/s00423-025-03959-3)
Supplement: Supplementary file 1 — Supplementary file1 (ZIP 55327 KB) [file 423_2025_3959_MOESM1_ESM.zip › literature.zip!/meta/Ota 2018.pdf]

Accepted Manuscript

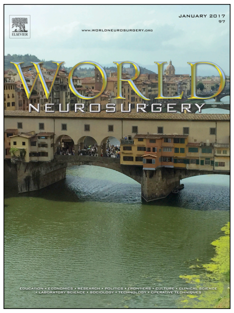

Evaluation of Microsurgery for Managing Giant or Complex Cerebral Aneurysms: A Retrospective Study

Nakao Ota, M.D, Hidetoshi Matsukawa, M.D., Ph.D, Kosumo Noda, M.D, Hirotaka Sato, M.D, Yuto Hatano, M.D, Atsumu Hashimoto, M.D, Takanori Miyazaki, M.D, Tomomasa Kondo, M.D, Yu Kinoshita, M.D, Norihiro Saito, M.D., Ph.D, Hiroyasu Kamiyama, M.D, Sadahisa Tokuda, M.D, Kyousuke Kamada, M.D., Ph.D, Rokuya Tanikawa, M.D

PII: S1878-8750(18)30723-X

DOI: 10.1016/j.wneu.2018.04.007

Reference: WNEU 7843

To appear in: *World Neurosurgery*

Received Date: 6 February 2018

Revised Date: 31 March 2018

Accepted Date: 2 April 2018

Please cite this article as: Ota N, Matsukawa H, Noda K, Sato H, Hatano Y, Hashimoto A, Miyazaki T, Kondo T, Kinoshita Y, Saito N, Kamiyama H, Tokuda S, Kamada K, Tanikawa R, Evaluation of Microsurgery for Managing Giant or Complex Cerebral Aneurysms: A Retrospective Study, *World Neurosurgery* (2018), doi: 10.1016/j.wneu.2018.04.007.

This is a PDF file of an unedited manuscript that has been accepted for publication. As a service to our customers we are providing this early version of the manuscript. The manuscript will undergo copyediting, typesetting, and review of the resulting proof before it is published in its final form. Please note that during the production process errors may be discovered which could affect the content, and all legal disclaimers that apply to the journal pertain.

ACCEPTED MANUSCRIPT

Evaluation of Microsurgery for Managing Giant or Complex Cerebral Aneurysms: A Retrospective Study

Nakao Ota[1], M.D., Hidetoshi Matsukawa[1], M.D., Ph.D., Kosumo Noda[1], M.D., Hirotaka Sato[1], M.D., Yuto Hatano[1], M.D., Atsumu Hashimoto[1], M.D., Takanori Miyazaki[1], M.D., Tomomasa Kondo[1], M.D., Yu Kinoshita[1], M.D., Norihiro Saito[1], M.D., Ph.D., Hiroyasu Kamiyama[1], M.D., Sadahisa Tokuda[1], M.D., Kyousuke Kamada[2], M.D., Ph.D., and Rokuya Tanikawa[1], M.D.

Affiliations:

[1] Stroke Center, Department of Neurosurgery, Sapporo Teishinkai Hospital

3-1, Higashi 1, Kita 33, Higashi-ku, Sapporo, Hokkaido 065-0033, Japan

[2] Department of Neurosurgery, Asahikawa Medical University, Asahikawa, Japan

Details of previous presentations: None.

Financial and material support: None.

Acknowledgements: None.

Corresponding author: Nakao Ota

Sapporo Teishinkai Hospital

3-1, Higashi 1, Kita 33, Higashi-ku, Sapporo, Hokkaido 065-0033, Japan

Tel: +81-11-712-1131

ACCEPTED MANUSCRIPT

ACCEPTED MANUSCRIPT

E-mail: nakao1980@gmail.com

Post-publication corresponding author: Rokuya Tanikawa

Sapporo Teishinkai Hospital

3-1, Higashi 1, Kita 33, Higashi-ku, Sapporo, Hokkaido 065-0033, Japan

Tel: +81-11-712-1131

E-mail: taniroku@gmail.com

Keywords: basilar artery aneurysm, bypass surgery, complex aneurysm, dolichoectatic basilar aneurysm, giant aneurysm

Abbreviations: ACA, anterior cerebral artery; BA, basilar artery; BTO, balloon test occlusion; CTA, computed tomography angiography; EC, external carotid artery; ICA, internal carotid artery; MCA, middle cerebral artery; mRS, modified Rankin scale; ORs, odds ratios; PCA, posterior cerebral artery; PICA, posterior inferior cerebellar artery; SAH, subarachnoid hemorrhage; STA, superficial temporal artery; VA, vertebral artery; IRB, institutional review board; IC, internal carotid

ACCEPTED MANUSCRIPT

ACCEPTED MANUSCRIPT

Evaluation of Microsurgery for Managing Giant or Complex Cerebral Aneurysms: A Retrospective Study

Abstract

Objects

Surgical or endovascular treatment for giant or complex aneurysms is challenging. To evaluate clinical outcomes and factors affecting the prognosis of giant or complex aneurysms, and to better establish the role of microsurgery in the management strategy.

Methods

One hundred fifty-nine patients with surgically treated complex aneurysms were included. Thirty-two (20.1%) patients had giant aneurysms (≥25 mm); 57 (35.8%) had large aneurysms (≥15 mm). Poor outcome was defined as modified Rankin scale scores 3-6.

Results

The mean aneurysm size was 17.0 mm (range, 1.6-47.5 mm). One hundred sixteen aneurysms (80.0%) were in the anterior circulation; 43 (27.0%) were in the posterior circulation. One hundred thirty-eight (86.8%) aneurysms were completely occluded without residual aneurysms. Nineteen (11.9%) had minor aneurysm remnants; 2 (1.3%) had incomplete occlusion. Two patients (1.3%) with giant basilar artery (BA) trunk aneurysms experienced rupture of the treated aneurysm and died. Bypass surgery was combined with microsurgery in 148 patients (93.1%). Perforating artery infarction was observed postoperatively in 42 (26.4%) patients, and poor outcome was observed in 29 (18.2%). Male sex (p=0.016; adjusted odds ratio [OR]: 4.524 [1.949-10.500]), perforating artery infarction (p<0.001; adjusted OR: 13.625 [5.329-34.837]), and BA aneurysm location (p=0.003;

adjusted OR: 56.333 [6.830-464.657]) were significantly related to poor outcome. The aneurysm size (p=0.017; adjusted OR: 1.064 [1.021-1.107]), C1 aneurysm location (p=0.042; adjusted OR: 2.591 [0.986-6.811]), and BA aneurysm location (p=0.033; adjusted OR: 12.956 [3.197-52.505]) were significantly related to perforating artery infarction.

Conclusion

Microsurgery with bypass is effective for many different complex aneurysms, except BA aneurysms.

Running Title: Microsurgery for Giant/Complex Aneurysms

Introduction

The natural history of giant aneurysms is remarkably poor. The reported 5-year incidence rates of rupture were 40% for aneurysms in anterior circulation and 50% for aneurysms in posterior circulation.[1] Giant aneurysms are associated with a higher rate of mortality and rupture, and surgical treatment is challenging. Reportedly, mortality rates of giant aneurysms, whether ruptured and unruptured, were 6-22%.[2-4] Even if the aneurysm is less than 25 mm in diameter, some factors, such as thrombosis, calcification of the aneurysm, previous treatment, location of the aneurysm, and absence of collateral circulation, make it difficult to perform surgical treatment. An aneurysm that has these aforementioned factors is defined as a complex aneurysm.[5] Although surgical or endovascular treatment for giant or complex intracranial aneurysms is still challenging, the treatment strategies are partially established for aneurysms in certain locations, and good results have been reported.[2-4, 6-8] However, results for aneurysms in other locations, especially complex aneurysms in the posterior

ACCEPTED MANUSCRIPT

ACCEPTED MANUSCRIPT

circulation, are still unacceptable.[9]

In the present study, we aimed to evaluate the clinical outcomes and factors affecting the prognosis of surgically treated complex intracranial aneurysms, and to better establish the role of microsurgery in the management strategy.

Methods

Patients

This study was approved by the institutional review board (IRB) (approval number 2017-34), and the IRB approved a waiver for the requirement of patients' informed consent.

A complex aneurysm was defined as an aneurysm with (1) a diameter ≥25 mm; (2) previous treatment (surgically or endovascularly); (3) branches arising from the aneurysm itself, including an unclippable aneurysm such as a blister, fusiform, or dolichoectasic configuration; (4) an intraluminal thrombus, and (5) severe unclippable calcification. The presence or absence of collateral circulation was not assessed because the balloon test occlusion is not reliable for predicting postoperative infarction at some locations.[10] Hence, in the case that an aneurysm was not feasible for neck clipping, bypass was performed. Indications for the bypass are described later herein.

From April 2012 to December 2016, 159 complex aneurysms (159 patients) were treated microsurgically by the senior author (R.T.) and his team. Of these, 32 patients (20.1%) had giant aneurysms (≥25 mm), and 57 (35.8%) had large aneurysms (≥15 mm). One hundred eleven (69.8%) women and 48 (30.2%) men with a mean age of 59.92 years (range, 11-89 years) were included.

Aneurysm morphology

Dolichoectasic aneurysms were defined as fusiform, circumferential dilatations of the basilar trunk. Fusiform aneurysms were defined as neither saccular nor dolichoectasic aneurysms.

Indications for bypass

The indications for performing bypass were as follows. 1) Planned parent artery occlusion: when the anterograde blood flow was occluded, bypass was performed without balloon test occlusion (BTO). 2) Pressure monitoring: when the high-flow bypass, such as external carotid artery (EC) to M2 bypass or V3 to posterior cerebral artery (PCA) bypass, was performed, pressure monitoring was performed via the superficial temporal artery (STA)-middle cerebral artery (MCA) bypass or STA-superior cerebellar artery bypass. 3) Protective bypass, preoperative and intraoperative findings of the aneurysm cannot indicate whether clipping is possible, or cannot predict the temporally occlusion time. In such cases, bypass was performed before repairing the aneurysm to secure the distal blood flow. 4) Reconstruction of the cortical branch: in patients in whom some cortical branches became trapped after repairing the aneurysm, the cortical branches were reconstructed.

Surgical management

Direct aneurysm occlusion with conventional clipping with or without bypass was the primary treatment strategy for saccular clippable aneurysms. For fusiform aneurysms, angioplastic clipping with preservation of the anterograde blood flow was considered if possible. Indirect aneurysm occlusion was selected for unclippable aneurysms that required clipping of the parent artery (proximal or distal occlusion, or trapping) with bypass or cervical internal carotid artery (ICA) ligation with bypass. One exception was vertebral artery aneurysm. In case of unilateral non-clippable vertebral artery aneurysms, simple parent artery occlusion without bypass was considered.

ACCEPTED MANUSCRIPT

Outcomes

Aneurysm occlusion was classified using postoperative computed tomography angiography (CTA) as complete (no residual aneurysm, trapping, and proximal or distal parent artery occlusion with no filling into aneurysm), residual (small neck remnant and proximal or distal parent artery occlusion with partially filling into aneurysm by reverse blood flow by 6 months postoperatively), or incomplete (major residual or proximal or distal parent artery occlusion with filling into the aneurysm of more than one-third of the previous aneurysm by 6 months postoperatively). Bypass patency was also assessed by CTA immediately postoperatively.

A neurological and general medical evaluation was performed by a neurosurgeon. Modified Rankin scale (mRS) scores were measured at discharge and at 6 months postoperatively based on medical charts or telephone interviews. We defined death or poor outcome as an mRS score of 3-6. If the preoperative mRS score was 3 or worse, postoperative worsening of the mRS score was also defined as a poor outcome.

Statistical analysis

Variables are expressed as a mean±standard deviation, median (interquartile range), or number of patients (%), as appropriate. For continuous variables, we first used the Shapiro-Wilk test to determine if the distributions were normal. If they were normal, we analyzed the data using the t-test, but otherwise we used the Mann-Whitney U-test. The chi-square test or Fisher exact test was used to analyze categorical variables, as appropriate.

Any differences that were statistically significant in univariate analysis or considered clinically meaningful were further analyzed with multivariate logistic regression adjustment.

Odds ratios (ORs) in multivariate analysis reflect the risk of developing poor outcome or

ACCEPTED MANUSCRIPT

ACCEPTED MANUSCRIPT

perforating artery infarctions. The goodness of fit of the regression model was confirmed by demonstrating a nonsignificant p value in the Hosmer-Lemeshow test.

Data were analyzed with IBM SPSS Statistics for Mac (version 24.0, IBM Corp., Armonk, NY, USA). Statistical significance was defined as $p<0.05$.

Results

Characteristics of the aneurysms

The mean aneurysm size was 17.0 mm (range, 1.6-47.5 mm). Forty-two patients (26.4%) had thrombosed aneurysms. Twenty-two (13.8%) patients were previously treated. Six patients (3.8%) presented with a subarachnoid hemorrhage (SAH). The Hunt & Kosnik grades were as follows: grade III, 1 patient; grade IV, 2 patients; and grade V, 3 patients.

Of 159 aneurysms, 116 (80.0%) were location in the anterior circulation, and 43 (27.0%) were located in the posterior circulation. Among 116 anterior circulation aneurysms, 26 (16.0%) were located in the C1 segment of the ICA, 31 (19.5%) were located in the C2 segment of the ICA, 34 (21.4%) were located in the extradural ICA (C3-C7), 18 (11.3%) were located in the MCA, and 7 (4.4%) were located in the anterior cerebral artery (ACA). Among 43 posterior circulation aneurysms, 14 (8.8%) were located in the basilar artery (BA), 17 (10.7%) in the vertebral artery, 7 (4.4%) in the PCA, and 5 (3.1%) in the posterior inferior cerebellar artery (PICA).

One hundred one aneurysms (63.5%) had a fusiform shape, and 49 (30.8%) were saccular in shape; 4 patients (2.5%) had dolichoectasic aneurysms, and 5 (3.1%) had acute dissecting aneurysms.

Aneurysm outcomes

ACCEPTED MANUSCRIPT

Of 159 aneurysms, direct neck clipping was performed in 46 (28.9%). The other 113 (71.1%) aneurysms were treated with an indirect procedure, such as proximal clipping (21 [13.2%]), internal carotid (IC) ligation (40 [25.2%]), and trapping (56 [35.2%]) (Table 1).

One hundred thirty-eight (86.8%) aneurysms were completely occluded with no residual aneurysm. Nineteen aneurysms (11.9%) had minor aneurysm remnants, and 2 aneurysms (1.3%) had incomplete occlusion (these giant BA trunk aneurysms were treated via proximal clipping with bypass; the patients experienced rupture of the treated aneurysm 1 week and 1 month postoperatively and died). Three patients (1.9%) had a retreatment after the first operation; among these patients, 2 had a cavernous portion giant aneurysm that was treated with cervical IC ligation and EC-M2 bypass. There were small aneurysm remnants via retrograde flow from the EC-M2 bypass. During conservation, trigeminal neuralgia or diplopia appeared. Hence, aneurysm trapping was then performed by clipping proximal to the ophthalmic artery. Subsequently, the mass effect disappeared. Another case of re-treatment was a patient with a P1-P2 fusiform aneurysm treated by performing aneurysmal dome clipping and securing anterograde blood flow because a small perforating artery arose from just proximal to the aneurysm. Six months postoperatively, aneurysm recurrence was observed, so trapping with STA-P2 bypass was performed.

Bypass surgery was combined with microsurgery in 148 (93.1%) patients. Bypass patency immediately after the operation was observed in 147 (99.3%) patients. Bypass patency was maintained in 138 of 139 patients at the 6-month postoperative follow-up.

Patient outcomes

Table 1 shows the mRS scores at 6 months postoperatively. A poor outcome was observed in 29 (18.2%) patients, including 2 with an mRS score of 6.

ACCEPTED MANUSCRIPT

Perforating artery infarction, including asymptomatic infarction, was observed postoperatively in 42 (26.4%) patients. Twelve (7.5%) cases of cortical infarction were also observed. The relationship between perforating artery infarction and the aneurysm location is shown in Table 2.

Factors related to a poor outcome

Table 2 shows the factors related to a poor outcome in univariate analysis. Age, sex, aneurysm size, aneurysm location (C2, C3-C7, and BA), SAH, perforating artery infarction, cortical infarction, previous treatment, proximal clipping, and cervical IC ligation were significantly correlated with a poor outcome.

Multivariate logistic regression analysis showed that male sex (p=0.016; adjusted OR: 4.524 [1.949-10.500]), perforating artery infarction (p<0.001; adjusted OR: 13.625 [5.329-34.837]), and BA aneurysm location (p=0.003; adjusted OR: 56.333 [6.830-464.657]) were significantly related to a poor outcome (Table 3).

Factors related to perforating artery infarction

Table 2 shows the factors related to perforating artery infarction in univariate analysis. The aneurysm size, SAH, contusion, diabetes mellitus, cervical IC ligation, and aneurysm location (C3-C7, BA, and vertebral artery [VA]) were significantly correlated with perforating artery infarction.

Multivariate logistic regression analysis showed that the aneurysm size (p=0.017; adjusted OR: 1.064 [1.021-1.107]), C1 aneurysm location (p=0.042; adjusted OR: 2.591 [0.986-6.811]), and BA aneurysm location (p=0.033; adjusted OR: 12.956 [3.197-52.505]) were significantly associated with perforating artery infarction (Table 4).

Treatment and outcomes of patients with BA aneurysms

ACCEPTED MANUSCRIPT

Of 14 cases of BA aneurysms (Table 5), 9 were saccular, 4 were dolichoectasic, and 1 was fusiform shaped. Neck clipping was performed in 6 patients, proximal occlusion was performed in 7 patients, and trapping was performed in 1 patient. Perforating artery infarction was observed in 11 patients (78.6%), and venous infarction was observed in 1 patient (7.1%). A poor outcome was observed in 12 patients (85.7%).

Discussion

Summary of the present study's findings

In the present study, a good outcome was observed in 81.8% of patients, and the complete occlusion rate was 86.8%. However, the prognostic factors of a poor outcome were perforating artery infarction and BA aneurysms. The rate of perforating artery infarction of BA aneurysms was 78.6%. These results showed that microsurgical treatment for complex cerebral aneurysms is effective for most aneurysms, including VA aneurysms but excluding BA aneurysms.

Although the complexity of an aneurysm partially depends on the patient, Hanel and Spetzlar reported that complex aneurysms depend on the following characteristics:[5] (1) a diameter >25 mm; (2) location; (3) previous treatments; and (4) presence or absence of collateral circulation, (5) intraluminal thrombus, and (6) calcification of the aneurysmal wall. The present study showed that the complexity of an aneurysm is dependent on its size, previous treatment, especially previous coiling, and aneurysm location. The reason why our study did not indicate the presence or absence of an intraluminal thrombus or calcification as a factor for determining an aneurysm's complexity was because of our surgical strategy. We routinely perform bypass if a patient's anterograde circulation is obliterated; the presence of collateral

circulation is not considered an indication for bypass. Additionally, in patients with a luminal thrombus or calcification, aneurysmectomy and thrombectomy were performed to make it easy to dissect the aneurysm from surrounding structures and repair it. However, this strategy cannot be used for BA aneurysms.

Surgical strategy for complex aneurysms

The bypass surgery and skull base technique, a surgical strategy for complex aneurysms at some locations, such as the MCA,[11, 12] ACA,[8, 13] PCA,[6] VA,[7] and extradural ICA,[14] have good results. The standard treatment strategy for aneurysms in these aforementioned locations is the bypass technique. We prefer to use the protective bypass strategy, wherein the bypass should be made distal to the aneurysm before repairing the aneurysm. Protective bypass enables the surgeon to secure distal blood flow during the approach to the aneurysm as well as during additional revascularization, which is crucial when treating complex aneurysms. To approach complex aneurysms, trapping is sometimes needed to identify the surrounding structure for thrombectomy and during complex revascularization. Thus, the occlusion time cannot be predicted, and severe ischemia may occur while repairing the aneurysm and reconstructing vessels; however, this can effectively be avoided by using the protective bypass strategy. Establishing protective bypasses before approaching the aneurysm can relieve the surgeon's concerns about distal blood flow during trapping of the aneurysm.

Even if major cortical arteries are secured by bypasses, the possibility of perforating artery infarction remains. The reason for the good outcomes observed for most aneurysm locations, except BA aneurysms, is that perforating artery infarction does not always become a severe disability, and long-term rehabilitation can improve patients' condition. In addition, preservation of the perforating artery is much easier than that of the BA location, because the

ACCEPTED MANUSCRIPT

lesions are relatively shallow, making it easier to preserve the perforating arteries.

Surgical treatment for complex BA aneurysms

Surgical or endovascular treatment for giant or complex BA aneurysms is challenging.[1, 3, 9, 15-19] For the saccular BA aneurysms, Krisht et al.[18] reported that the reasons of complexity for BA aneurysms are as follows: 1) the limited space leads to difficult maneuverability around the aneurysm; 2) the brainstem and thalamus have limited tolerance to prolonged temporary occlusion; 3) surgeons have limited knowledge of the collateral blood flow system, and 4) dissectability of the perforators from the wall of the aneurysm is limited. Hence, we chose to treat our patients by flow alternation if neck clipping was not feasible, such as bypass with parent artery occlusion. However, the outcomes are unacceptable because of infarction of the perforating artery and rupture postoperatively (Figures 1 and 2). Lawton et al.[9] reported their surgical outcome for dolichoectasic BA aneurysms after treating them with flow alteration and bypass. The results during the study period improving by changing the bypass strategy and occlusion site, but they were not good. Perforating artery infarction is one of the main reasons for a poor outcome, and it indicates that bypass surgery cannot directly improve outcomes. Endovascular treatment, such as a flow-diverting stent, also has been reported to be associated with poor outcomes for this lesion, although excellent results with this treatment have been observed at other locations lacking perforators.[15-17] In their large series, Drake et al. described the treatment of BA trunk fusiform aneurysms by various surgical methods such as wrapping, single VA occlusion, bilateral VA occlusion, creation of severe BA stenosis by using a tourniquet, and lower basilar occlusion and trapping. Each surgical procedure showed good and poor outcomes, including postoperative rupture of the previous intact aneurysm. The factors related to poor outcomes were brain stem ischemia and

postoperative rupture, which were the same as in our study. Drake et al.'s series suggests that some surgical flow reduction approaches can stabilize several aneurysms, but others can cause rupture after the surgical intervention. The problem is that it is unknown which vessel should be occluded, as it depends on each case. We need to establish a strategy based on the logic of computational flow dynamics, not on the inventiveness of neurosurgeons.[20] Postoperative intraluminal flow should be predicted by preoperative simulation and computational flow dynamics,[21] and patient-oriented flow alteration with bypass surgery should be performed, which may improve outcomes.

Limitation

This study was retrospective and nonrandomized. Although the sample size was enough to discuss complex or giant aneurysms, the numbers of aneurysms at each location were small.

Conclusions

Microsurgical treatment with bypass for complex aneurysms is effective for many different aneurysms, except BA aneurysms. For treating BA aneurysms, an additional treatment strategy such as preoperative computational flow dynamics simulation is needed.

References

1. Wiebers DO, Whisnant JP, Huston J, 3rd, et al. Unruptured intracranial aneurysms: natural history, clinical outcome, and risks of surgical and endovascular treatment. *Lancet.* Jul 12 2003;362(9378):103-110.
2. Gewirtz RJ, Awad IA. Giant aneurysms of the anterior circle of Willis: management outcome of open microsurgical treatment. *Surg Neurol.* May 1996;45(5):409-420; discussion 420-401.
3. Lawton MT, Spetzler RF. Surgical management of giant intracranial

ACCEPTED MANUSCRIPT

aneurysms: experience with 171 patients. *Clin Neurosurg.* 1995;42:245-266.

4. Piepgras DG, Khurana VG, Whisnant JP. Ruptured giant intracranial aneurysms. Part II. A retrospective analysis of timing and outcome of surgical treatment. *J Neurosurg.* Mar 1998;88(3):430-435.

5. Hanel RA, Spetzler RF. Surgical treatment of complex intracranial aneurysms. *Neurosurgery.* Jun 2008;62(6 Suppl 3):1289-1297; discussion 1297-1289.

6. Ota N, Goehre F, Miyazaki T, et al. Bypass revascularization applied to the posterior cerebral artery. *World Neurosurg.* Sep 16 2016.

7. Ota N, Tanikawa R, Eda H, et al. Radical treatment for bilateral vertebral artery dissecting aneurysms by reconstruction of the vertebral artery. *J Neurosurg.* Oct 2016;125(4):953-963.

8. Ota N, Tanikawa R, Miyama M, et al. Surgical strategy for complex anterior cerebral artery aneurysms: retrospective case series and literature review. *World Neurosurg.* Nov 5 2015.

9. Lawton MT, Abla AA, Rutledge WC, et al. Bypass Surgery for the Treatment of Dolichoectatic Basilar Trunk Aneurysms: A Work in Progress. *Neurosurgery.* Jul 2016;79(1):83-99.

10. Jayakumar PN, Desai S, Srikanth SG, Ravishankar S, Kovoor JM. Relevance of occlusion test in endovascular coiling of posterior cerebral artery (p2 segment) aneurysms. *Interv Neuroradiol.* Sep 30 2004;10(3):235-248.

11. Nakajima H, Kamiyama H, Nakamura T, Takizawa K, Tokugawa J, Ohata K. Direct surgical treatment of giant middle cerebral artery aneurysms using microvascular reconstruction techniques. *Neurol Med Chir (Tokyo).* 2012;52(2):56-61.

12. Tayebi Meybodi A, Huang W, Benet A, Kola O, Lawton MT. Bypass surgery for complex middle cerebral artery aneurysms: an algorithmic approach to revascularization. *J Neurosurg.* Sep 2017;127(3):463-479.

13. Abla AA, Lawton MT. Anterior cerebral artery bypass for complex aneurysms: an experience with intracranial-intracranial reconstruction and review of bypass options. *J Neurosurg.* Jun 2014;120(6):1364-1377.

14. Ishishita Y, Tanikawa R, Noda K, et al. Universal extracranial-intracranial graft bypass for large or giant internal carotid aneurysms: techniques and

results in 38 consecutive patients. *World Neurosurg.* Jul-Aug 2014;82(1-2):130-139.

15. Meckel S, McAuliffe W, Fiorella D, et al. Endovascular treatment of complex aneurysms at the vertebrobasilar junction with flow-diverting stents: initial experience. *Neurosurgery.* Sep 2013;73(3):386-394.

16. Toth G, Bain M, Hussain MS, et al. Posterior circulation flow diversion: a single-center experience and literature review. *J Neurointerv Surg.* Aug 2015;7(8):574-583.

17. van Oel LI, van Rooij WJ, Sluzewski M, Beute GN, Lohle PN, Peluso JP. Reconstructive endovascular treatment of fusiform and dissecting basilar trunk aneurysms with flow diverters, stents, and coils. *AJNR Am J Neuroradiol.* Mar 2013;34(3):589-595.

18. Krisht AF, Kadri PA. Surgical clipping of complex basilar apex aneurysms: a strategy for successful outcome using the pretemporal transzygomatic transcavernous approach. *Neurosurgery.* Apr 2005;56(2 Suppl):261-273; discussion 261-273.

19. Drake CG, Peerless SJ. Giant fusiform intracranial aneurysms: review of 120 patients treated surgically from 1965 to 1992. *J Neurosurg.* Aug 1997;87(2):141-162.

20. Alnaes MS, Mardal KA, Bakke S, Sorteberg A. Computational fluid dynamics evaluation of flow reversal treatment of giant basilar tip aneurysm. *Interv Neuroradiol.* Oct 2015;21(5):586-591.

21. Miura Y, Ishida F, Kamei Y, et al. A Case of Vertebral Artery Fusiform Aneurysm Treated by Flow Alteration: Successful Prediction of Therapeutic Effects Using Computational Fluid Dynamics. *NMC Case Rep J.* Oct 2017;4(4):107-110.

Figure Legends

Figure 1. Case 7: Flow reduction with V3-saphenous vein graft (SVG)-P2, superficial temporal artery (STA)-superior cerebellar artery (SCA), and STA-anterior inferior cerebellar artery (AICA) bypass

A 70-year-old man had progressive ataxia and difficulty walking. For the purpose of flow

ACCEPTED MANUSCRIPT

alteration, left V4 clipping with V3-SVG-P2, STA-SCA, and STA-AICA bypass was performed. The right side of the vertebral artery (VA) was secured to maintain the right side posterior inferior cerebellar artery flow. Postoperatively, however, infarction of the right side of the cerebellum and pons was observed, and the patient developed quadriparesis. Seven days postoperatively, he suddenly deteriorated; the computed tomography (CT) scan showed subarachnoid hemorrhage (SAH) due to the treated aneurysm and he died.

A: Preoperative computed tomography angiogram revealing a dolichoectasic aneurysm in the lower basilar artery trunk.

B: Postoperative computed tomography angiogram showing good filling of the V3-P2, STA-SCA, and STA-AICA bypass. The left side of the VA is occluded by clipping. Most of the aneurysm is thrombosed immediately after the operation.

C: Postoperative T2-weighted magnetic resonance imaging scan showing infarction of the right side of the medulla and cerebellum.

D: One week postoperatively, the CT scan shows SAH around the thrombosed aneurysm.

Figure 2. Case 13: Proximal ligation with bypass

A left side basilar artery (BA)-superior cerebellar artery (SCA) saccular aneurysm was incidentally identified 67-year-old man, and endovascular coiling was performed at another hospital. However, aneurysm recurrence was observed, and he was referred to our institution. Proximal BA ligation with superficial temporal artery (STA)-posterior cerebral artery (PCA) bypass and STA-SCA bypass was planned. Intraoperative findings showed a large perforating artery to the brainstem just proximal to the ligated site. We have found that these perforators

ACCEPTED MANUSCRIPT

will become occluded due to delayed thrombosis. Hence, SCA transposition (i.e., cut the SCA and BA proximal to the ligation site to the SCA and perform end-to-side anastomosis) was performed. Postoperative imaging showed good filling of the STA-PCA and transposed SCA. However, infarction of the brainstem was observed, and the patient was discharged with a modified Rankin scale score of 5.

A: Preoperative computed tomography angiogram showing the BA-SCA aneurysm with coil compaction.

B: T2-weighted magnetic resonance imaging scan showing the aneurysm compressing the cerebral peduncle.

C: Intraoperative view and schema of this treatment. First, STA-PCA and STA-SCA bypass was performed. Then, to make the blood flow out of anterograde, STA transposition was performed. Finally, proximal ligation was performed between the transposed SCA and PCA.

D: Postoperative computed tomography angiogram showing good filling of the STA-PCA bypass. The BA trunk is also filling just proximal to the ligated site and flowing out to the transposed SCA.

E, F: Postoperative T2-weighted and diffusion-weighted magnetic resonance imaging scans showing infarction of the midbrain.

ACCEPTED MANUSCRIPT

ACCEPTED MANUSCRIPT

	No.(%), n=159
Sex	
Female	111 (69.8%)
Male	48 (30.2%)
Age	
Mean	59.92(11- 89)
SAH	6 (3.8%)
Hunt & Kosnik Gr	III:1, IV:2, V:3
WFNS Gr	II:1, IV:1, V:4
Thrombosed	42 (26.4%)
Previously treated	22 (13.8%)
Previously clipping	16 (10.1%)
Previously Coiling	6 (3.8%)
Shape	
Saccular	49 (30.8%)
Fusiform	101 (63.5%)
Doclicoectasic	4 (2.5%)
Dissecting	5 (3.1%)
Aneurysm location	
C1	26 (16.4%)
C2	31 (19.5%)
C3-C7	34 (21.4%)
MCA	18 (11.3%)
ACA	7 (4.4%)
BA	14 (8.8%)
VA	17 (10.7%)
PCA	7 (4.4%)
PICA	5 (3.1%)
Treatment	
Bypass surgery	148 (93.1%)
Neck clipping	46 (28.9%)
Proximal clipping	21 (13.2%)

IC ligation	40 (25.2%)
Distal clipping	0
Trapping	56 (35.2%)
Outcome of Aneurysm	
Complete occlusion	138 (86.8%)
Minor remnant	19 (11.9%)
Incomplete	2 (1.3%)
Rupture after operation	2 (1.3%)
Re-treatment after operation	3 (1.9%)
Outcome	
mRS 0	97 (61.0%)
mRS 1	27 (17.0%)
mRS 2	15 (9.4%)
mRS 3	5 (3.1%)
mRS 4	1 (0.6%)
mRS 5	12 (7.5%)
mRS 6	2 (1.3%)
Poor outcome	29 (18.2%)
Bypass patency immediately after operation	147 (99.3%)
Bypass patency 6 months after operation	138 occlusion 1
Perforating artery infarction	42 (26.4%)
Cortical infarction	12 (7.5%)

Table 1. Patients' baseline characteristics

C1: C1 segment of internal carotid artery, C2: C2 segment of internal carotid artery, C3-C7: C3 to C7 segment of internal carotid artery, MCA: middle cerebral artery, ACA:

ACCEPTED MANUSCRIPT

ACCEPTED MANUSCRIPT

anterior cerebral artery, BA: Basilar artery, VA: vertebral artery, PCA: posterior cerebral artery, PICA: posterior inferior cerebellar artery, mRS: modified Rankin Scale score

ACCEPTED MANUSCRIPT

ACCEPTED MANUSCRIPT

	No.(%), n=159	Poor outcome n(%)		P value	Perforating artery infarction n(%)		P value
		Good (n=130)	Poor (n=29)		No (n=117)	Yes (n=42)	
Sex*				P=0.001			P=0.366
Female	111 (69.8%)	99 (76.2)	12 (41.4)		84 (71.8)	27 (64.3)	
Male	48 (30.2%)	31 (23.8)	17 (58.6)		33 (28.2)	15 (35.7)	
Age (Yrs) *				P=0.064			P=0.144
Mean	59.9 ± 13.9	59.0 ± 14.1	64.2 ± 12.7		59.0 ± 14.1	62.6 ± 13.2	
Size (mm) *				P=0.001			P= 0.007
Mean	17.0 ± 8.8	15.9 ± 8.3	22.0 ± 10.1		15.7 ± 8.0	20.8 ± 10.5	
SAH‡	6 (3.8%)	2 (1.5)	4 (66.6)	P=0.011	2 (33.3)	4 (66.6)	P=0.042
Hunt & Kosnik Gr	III:1, IV:2, V:3						
WFNS Gr	II:1, IV:1, V:4						
Perforating artery infarction‡	42 (26.4%)	21(16.2)	21 (72.4)	P<0.001			
Cortical infarction‡	12 (7.5%)	7 (5.4)	5 (17.2)	P=0.045	7 (6.0)	5 (11.9)	P=0.304
Venous infarction‡	3	1 (0.7)	2 (6.9)	P=0.086	2 (1.7)	1 (2.4)	P=1.000
Contusion‡	11	9 (6.9)	2 (6.9)	P= 1.000	11 (9.4)	0	P= 0.038
Thrombosed‡	42 (26.4%)	30 (23.1)	12 (41.4)	P=0.061	28 (23.9)	14 (33.3)	P= 0.307
Previously treated‡	22 (13.8%)	15 (11.5)	7 (24.1)	P=0.039	14 (12.0)	8 (19.0)	P =0.199
Previously clipping‡	16 (10.1%)	12 (9.2)	4 (13.8)	P=0.495	11 (9.4)	5 (11.9)	P =0.765
Previously Coiling‡	6 (3.8%)	3 (2.3)	3 (10.3)	P=0.075	3 (2.6)	3 (7.1)	P =0.189
DM‡	5	3 (2.3)	2 (6.9)	P= 0.207	1 (0.9)	4 (9.5)	P= 0.016
Smoking‡	62	50 (38.5)	12 (41.4)	P=0.835	43 (36.8)	19 (45.2)	P=0.363
Hypertension‡	58	46 (35.4)	12 (41.4)	P = 0.391	42 (35.9)	16 (38.1)	P= 0.707
Hyperlipidemia‡	50	38 (29.2)	12 (41.4)	P= 0.173	37 (31.6)	13 (31.0)	P=1.000
Shape†				P=0.743			P=0.671
Saccular	49 (30.8%)	37 (28.5)	12 (41.4)		31 (26.5)	18 (42.9)	
Fusiform	101 (63.5%)	90 (69.2)	11 (37.9)		83 (70.9)	18 (42.9)	
Doclicoectasic	4 (2.5%)	0	4 (13.8)		0	4 (9.5)	
Dissecting	5 (3.1%)	3 (2.3)	2 (6.9)		3 (2.6)	2 (4.8)	
Aneurysm location†				P= 0.004			P=0.767
C1‡	26 (16.4%)	21 (16.2)	5 (17.2)	P=1.000	15 (12.8)	11 (26.2)	P=0.054

ACCEPTED MANUSCRIPT

ACCEPTED MANUSCRIPT

C2‡	31 (19.5%)	30 (23.1)	1 (3.5)	P=0.017	26 (22.2)	5 (11.9)	P=0.178
C3-C7‡	34 (21.4%)	34 (26.2)	0	P=0.001	30 (25.6)	4 (9.5)	P=0.030
MCA‡	18 (11.3%)	14 (10.8)	4 (13.8)	P=0.745	12 (10.3)	6 (14.3)	P=0.571
ACA‡	7 (4.4%)	6 (4.6)	1 (3.5)	P=1.000	5 (4.3)	2 (4.8)	P=1.000
BA‡	14 (8.8%)	1 (0.7)	13 (44.8)	P<0.001	3 (2.6)	11 (26.2)	P<0.001
VA‡	17 (10.7%)	14 (10.7)	3 (10.3)	P=1.000	16 (13.7)	1 (2.4)	P=0.044
PCA‡	7 (4.4%)	6 (4.6)	1 (3.5)	P=1.000	5 (4.3)	2 (4.8)	P=1.000
PICA‡	5 (3.1%)	4 (3.1)	1 (3.5)	P=1.000	5 (4.3)	0	P=0.326
Treatment							
Bypass surgery	148 (93.1%)	121 (93.1)	27 (93.1)		107 (91.5)	41 (97.6)	
Neck clipping‡	46 (28.9%)	35 (26.9)	11 (37.9)	P=0.261	31 (26.5)	15 (35.7)	P=0.321
Proximal clipping‡	21 (13.2%)	12 (9.2)	9 (31.0)	P=0.004	12 (10.3)	9 (21.4)	P=0.107
IC ligation‡	40 (25.2%)	40 (30.8)	0	P<0.001	35 (29.9)	5 (11.9)	P=0.023
Trapping‡	56 (35.2%)	47 (36.2)	9 (31.0)	P=0.672	43 (36.8)	13 (30.1)	P=0.574

Table 2. Factors related to poor outcome and perforating artery infarction in univariate analysis.

SAH: subarachnoid hemorrhage, DM: diabetes mellitus, C1: C1 segment of internal carotid artery, C2: C2 segment of internal carotid artery, C3-C7: C3 to C7 segment of internal carotid artery, MCA: middle cerebral artery, ACA: anterior cerebral artery, BA: Basilar artery, VA: vertebral artery, PCA: posterior cerebral artery, PICA: posterior inferior cerebellar artery, IC: internal carotid artery

*T test, †Mann- Whitney U test, ‡Fishers exact test

ACCEPTED MANUSCRIPT

ACCEPTED MANUSCRIPT

	Partial deviation coefficient	Significant probability (p)	Crude Odds ratio	Adjusted Odds ratio	95% CI lowest	95%CI Highest
Male Sex	1.656	0.016	4.524(1.949-10.500)	5.238	1.357	20.217
Perforating artery infarction	2.483	0.001>	13.625(5.329-34.837)	11.978	3.247	44.190
BA location	3.321	0.003	56.333 (6.830-464.657)	27.687	3.140	244.089

Table 3. Factors related to poor outcome in multivariate logistic regression analysis

Model chai square test $p<0.001$, Hosmer and Lemeshow test 0.935, Percentage of correct classification 91.7%

CI: confidential interval, BA: basilar artery

ACCEPTED MANUSCRIPT

	Partial deviation coefficient	Significant probability (p)	Crude Odds ratio	Adjusted Odds ratio	95% CI lowest	95%CI Highest
Size	0.063	0.017	1.064(1.021 -1.107)	1.065	1.011	1.121
C3-C7	-1.382	0.061	0.471(0.143 -1.549)	0.251	0.059	1.066
C1 location	1.131	0.042	2.591(0.986 -6.811)	3.100	1.044	9.209
BA location	1.691	0.033	12.956(3.197-52.505)	5.425	1.142	25.769

Table 4. Factors related to perforating artery infarction in multivariate logistic regression analysis

Model chai square test p<0.001, Hosmer and Lemeshow test 0.866, Percentage of correct classification 83.1%

ACCEPTED MANUSCRIPT

ACCEPTED MANUSCRIPT

Case. No.	Age/ Sex	Shape/ Size (mm)	SAH	Location	Surgical treatment	Bypass	6m mRS	Rupture after operation	infarction
1	75/F	Saccular /24.00	-	BA-top	proximal clipping	STA-PCA, STA-SCA	5	-	Perforating artery
2	65/M	Saccular /19.00	-	BA-SCA	Neck clipping	STA-PCA, STA-SCA	5	-	Not identified on MRI
3	61/M	Dolicoectasic /19.00	-	BA	Proximal clipping	STA-SCA, OA-AICA	5	-	Venous infarction
4	65/M	Saccular /27.10	-	BA -SCA	Neck clipping	STA-SCA	5	-	Perforating artery
5	75/M	Saccular /26.00	-	BA-SCA	Neck clipping	None	2	-	None
6	76/M	Saccular /19.50	+	BA -SCA	Neck clipping	STA-SCA	5	-	Perforating artery
7	70/M	Dolicoectasic /30.60	-	BA	Proximal clipping	V3-SVG-P2, STA-SCA, STA-AICA	6	Rupture after operation and died	Perforating artery

ACCEPTED MANUSCRIPT

ACCEPTED MANUSCRIPT

8	77/F	Saccular /39.00	-	BA-SCA	Proximal clipping	STA-SCA, STA-PCA, SCA proximal clipping	3	-	Perforating artery
9	67/F	Saccular /33.00	-	BA-top	Proximal clipping	STA-PCA, STA-SCA	5	-	Perforating artery
10	70/F	Fusiform /8.00	-	BA	Neck clipping	Clipping with OA-AICA bypass	0	-	Perforating artery
11	73/M	Dolicoectasic /27.30	-	BA	Trapping	OA-AICA, Ⅴ3-RA-P2	5	-	Perforating artery
12	66/F	Dolicoectasic /41.78	-	BA	Proximal clipping	STA-SCA and STA-PCA	6	Rupture 1 months after operation and died	Perforating artery
13	67/M	22.00	-	BA-SCA	Proximal clipping	STA-PCA, SCA transposition	4	-	Perforating artery
14	79/F	16.40	+	BA-top	Neck clipping	None	5	-	Perforating artery

ACCEPTED MANUSCRIPT

ACCEPTED MANUSCRIPT

Table 5. Surgical treatment and outcome of basilar artery aneurysms

ACCEPTED MANUSCRIPT

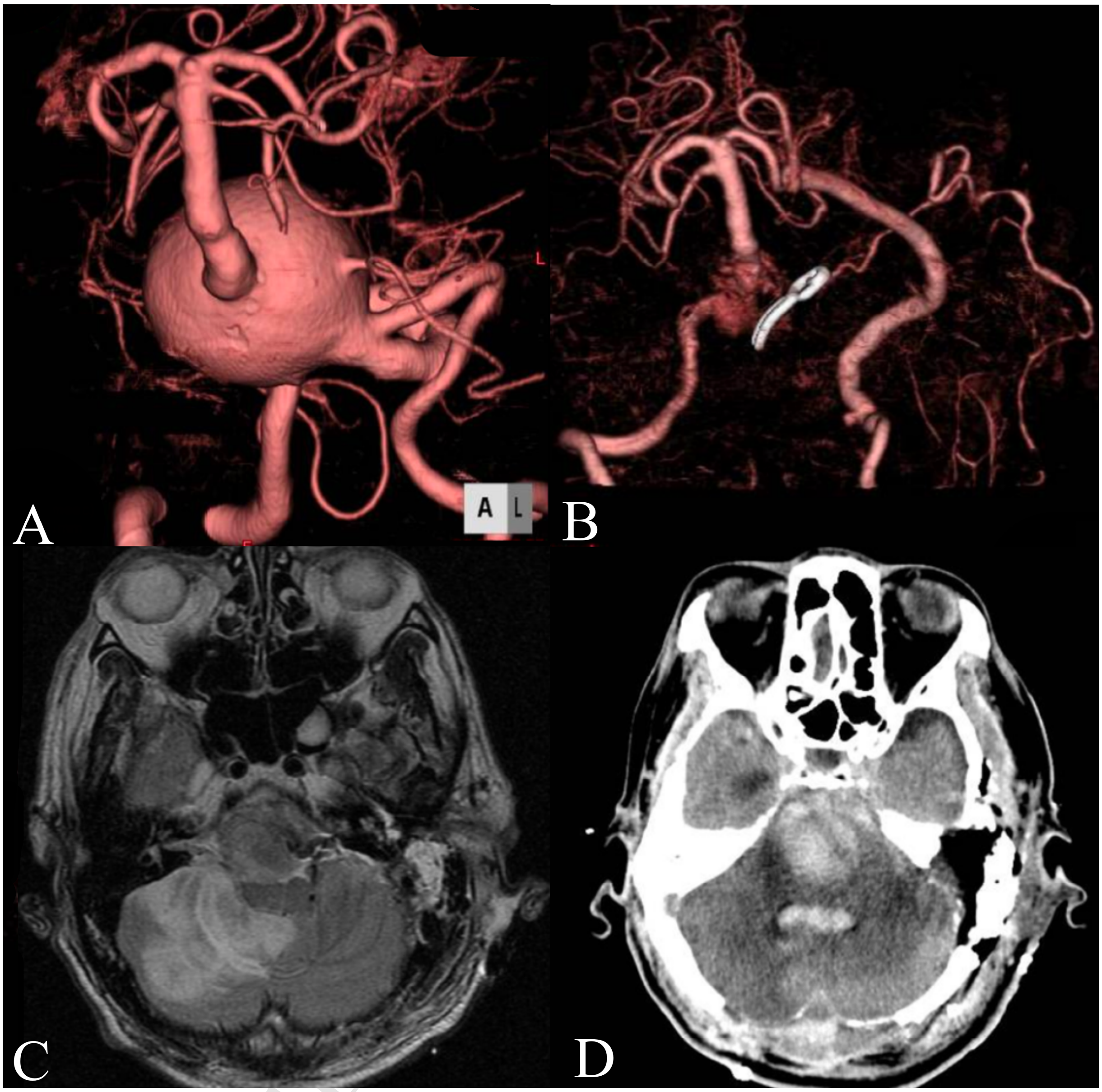

ACCEPTED MANUSCRIPT

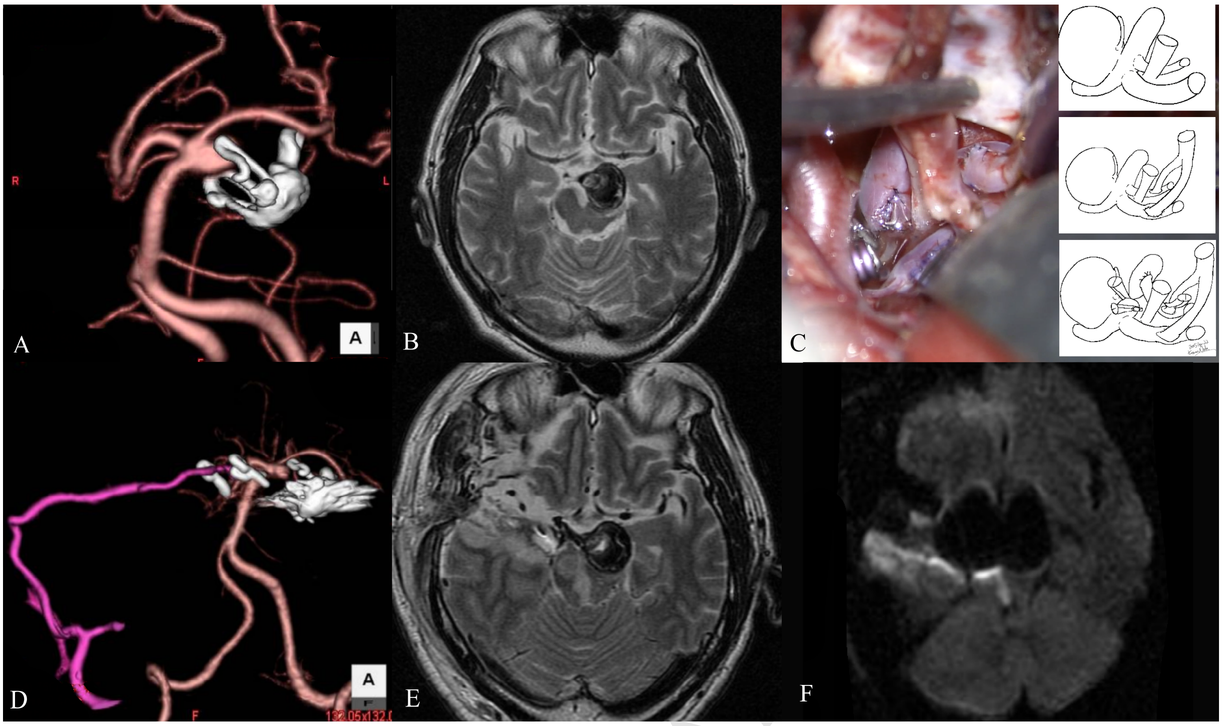

ACCEPTED MANUSCRIPT

ACCEPTED MANUSCRIPT

Highlights

One hundred fifty-nine patients with surgically treated complex aneurysms were included.

One hundred thirty-eight (86.8%) aneurysms were completely occluded without residual aneurysms.

Perforating artery infarction was observed postoperatively in 42 (26.4%) patients

poor outcome was observed in 29 (18.2%)

Male sex, perforating artery infarction and BA aneurysm location were significantly related to poor outcome

The aneurysm size, C1 aneurysm and BA aneurysm location were significantly related to perforating artery infarction.

ACCEPTED MANUSCRIPT
