# Supplementary material for: Management strategies and outcomes of basilar trunk aneurysms: a systematic review and meta-analysis
Source: Langenbecks Arch Surg. 2026 Jan 20;411(1):71. doi: 10.1007/s00423-025-03959-3 (PMC12852281; doi:10.1007/s00423-025-03959-3)

ENDOVASCULAR COIL EMBOLIZATION OF RUPTURED AND UNRUPTURED POSTERIOR CIRCULATION ANEURYSMS: REVIEW OF A 10-YEAR EXPERIENCE

Aditya S. Pandey, M.D.
Department of Neurosurgery,
Thomas Jefferson University Hospital,
Philadelphia, Pennsylvania

Christopher Koebbe, M.D.
Department of Neurosurgery,
Thomas Jefferson University Hospital,
Philadelphia, Pennsylvania

Robert H. Rosenwasser, M.D.
Department of Neurosurgery,
Thomas Jefferson University Hospital,
Philadelphia, Pennsylvania

Erol Veznedaroglu, M.D.
Department of Neurosurgery,
Thomas Jefferson University Hospital,
Philadelphia, Pennsylvania

Reprint requests:
Erol Veznedaroglu, M.D.,
Department of Neurosurgery,
Thomas Jefferson University Hospital,
909 Walnut Street,
Philadelphia, PA 19107.
Email: veznedaroglu@yahoo.com

Received, December 27, 2005.
Accepted, January 25, 2007.

OBJECTIVE: Treatment of posterior circulation aneurysms poses a great technical challenge for the practicing neurosurgeon. The advent of endovascular techniques has made such treatment more feasible. We report our experience with the endovascular management of ruptured and unruptured posterior circulation aneurysms during the past 10 years.

METHODS: A retrospective analysis was performed on all patients with posterior circulation aneurysms undergoing endovascular treatment at Jefferson Hospital for Neuroscience between July 1995 and December 2005. This yielded 275 patients (67 men and 208 women). The degree of aneurysm occlusion was determined by the operating endovascular neurosurgeon at the time of the procedure. Successful embolization was defined as greater than 95% occlusion of the dome without any coil prolapsing into the parent vessel. Clinical outcome was evaluated using the modified Glasgow Outcome Scale. Clinical follow-up data was obtained for 262 patients (95.3%); the follow-up period ranged from 1 to 94 months (mean, 31.8 mo for procedures performed before 2004 and 13.3 mo for procedures performed during 2004 and 2005). Angiographic follow-up data was obtained for 224 patients (84.8%) for periods ranging from 6 to 94 months (mean, 31.3 mo for procedures performed before 2004 and 13.7 mo for procedures performed during 2004 and 2005).

RESULTS: Based on the Hunt and Hess grading scale, the patient population included 106 patients (38.5%) with unruptured aneurysms, 43 patients (15.6%) with Grade I aneurysms, 16 patients (5.8%) with Grade II aneurysms, 56 patients (20.5%) with Grade III aneurysms, and 54 patients (19.6%) with Grade IV aneurysms. The locations of the posterior circulation aneurysms included 189 (68.7%) in the basilar apex or posterior cerebral artery, 23 (8.4%) in the basilar trunk/anterior inferior cerebellar artery, 22 (8%) in the superior cerebellar artery, and 41 (14.9%) in the vertebral artery or posterior inferior cerebellar artery. Of the 275 patients, 208 (76%) were women and 67 (24%) were men. The mean age at the time of treatment was 53.9 years (range, 7–90 yr). Of all patients treated, 237 patients (87.8%) had successful embolization (>95% occlusion of the dome). On angiographic follow-up, 55 patients (24.5%) developed recanalization of at least 5%. Retreatment was required in 11 patients (4.9%; 0.01%/patient yr) and rehemorrhage occurred in three patients (1.1%; 0.003%/patient yr). Clinical follow-up was graded using the modified Glasgow Outcome Scale (mGOS) and revealed 229 patients (87.4%) in the mGOS I category, 12 patients (4.6%) in the mGOS II category, eight patients (3%) in the mGOS III category, two patients (0.8%) in the mGOS IV category, and 11 patients (4.2%) were deceased (mGOS V). Clinically significant vasospasm requiring angioplasty occurred in 11 patients (6.5%) with subarachnoid hemorrhage, and 120 patients (71%) with subarachnoid hemorrhage required ventricular shunts. Complications causing clinical morbidity occurred in 14 patients (5.1%) and ranged from postoperative ischemia to recurrent subarachnoid hemorrhage. Of all clinical factors evaluated, Hunt and Hess grade was the strongest predictor of good clinical outcome ($P < 0.0001$).

CONCLUSION: Endovascular coil embolization of posterior circulation aneurysms is an effective treatment in the short term but is associated with recurrence, which requires close surveillance, possible retreatment, and can, albeit very rarely, lead to rehemorrhage. Future technological advancements such as the development of biologically active coils will be essential in the permanent obliteration of aneurysms.

KEY WORDS: Balloon assisted, Cerebral aneurysm, Coil embolization, Endovascular, Posterior circulation, Stent, Subarachnoid hemorrhage, Thromboembolism, Vasospasm

Neurosurgery 60:626–637, 2007 10.1227/01.NEU.0000255433.47044.8F www.neurosurgery-online.com

The treatment of posterior circulation aneurysms poses a great technical challenge for the practicing neurosurgeon (2, 13, 15, 18, 21). With the advent of endovascular techniques, such treatment is much more feasible and without the inherent risks associated with microsurgical procedures (14, 15, 30, 31). Endovascular embolization of cerebral aneurysms using platinum microcoils is a treatment made possible by the parallel evolution of several technologies, particularly computerized imaging and flexible, hydrophilic microcatheterization systems (1, 4, 8, 16, 25, 30, 31). The development of the detachable type of microcoil by Guglielmi et al. (16) took advantage of the newest developments in intravascular navigation to enable the percutaneous endovascular embolization of cerebral aneurysms. The first percutaneous coil embolization of an aneurysm in a living human using the Guglielmi detachable coil was reported in 1991, followed by Food and Drug Administration approval in 1995.

Technological advancements have also had a profound impact on the success of modern microneurosurgery. Craniotomy with clip ligation remains the preferred treatment for certain types of cerebral aneurysms, with operative morbidity and mortality approaching 3% in experienced hands for nongiant lesions of the anterior circulation (24). The outcomes for surgically treated patients are closely correlated with the presence and clinical grade of subarachnoid hemorrhage (SAH), as well as with the age and premorbid medical condition of the patient (23, 36). Although the operative treatment of posterior circulation aneurysms carries a higher morbidity, the technical and clinical success of this form of treatment remains high (35). For cases posing relatively high medical and surgical risk, the alternative treatment modality of endovascular coil embolization may be useful. This is particularly true for posterior circulation aneurysms, which are generally more difficult to treat surgically and carry a higher operative morbidity and mortality (15, 26).

Neurosurgeons in our Cerebrovascular Division who are trained in both microsurgical clip ligation and endovascular coil embolization use a multimodality treatment strategy to treat cerebral aneurysms. We report our 10-year experience of treating posterior circulation aneurysms with coil embolization in 275 patients harboring ruptured and unruptured aneurysms. To our knowledge, this represents the largest single institution series of posterior circulation aneurysms treated using coil embolization to date (3, 14, 15, 27, 33, 34).

PATIENTS AND METHODS

A retrospective analysis officially approved by the Thomas Jefferson University Institutional Review Board was performed on all patients with posterior circulation aneurysms undergoing endovascular treatment at The Jefferson Hospital for Neuroscience between July 1995 and December 2005. This review included operative reports, angiographic studies, magnetic resonance imaging scans, computed tomographic scans, outpatient charts, and telephone follow-up evaluations. The analysis yielded 275 patients; 208 were women and 67 were men. The Hunt and Hess (HH) grades were present for all patients. The degree of aneurysm occlusion was determined by the operating endovascular neurosurgeon at the time of the procedure.

Clinical outcome was evaluated using the modified Glasgow Outcome Scale (mGOS) and was scored as follows: I, minor deficits but not hindering normal activity; II, independent but with moderate disability; III, severely disabled and dependent on others for activities of daily living; IV, persistent vegetative state; and V, deceased. Clinical follow-up data was obtained for 262 patients (95.3%) for a period ranging from 1 to 94 months (mean, 31.8 mo for procedures performed before 2004 and 13.3 mo for procedures performed during 2004 and 2005). Angiographic follow-up data was obtained for 224 patients (84.8%) for periods ranging from 6 to 94 months (mean, 31.3 mo for procedures performed before 2004 and 13.7 mo for procedures performed during 2004 and 2005).

Statistical Analysis

Statistical analyses were performed using the commercial package JMP IN 5.1 (SAS Institute, Cary, NC). For categorical variables, contingency table analyses returned χ^2 values for the statistical significance of relationships between variables. For continuous variables (e.g., patient age and aneurysm dome size), analysis of variance was performed to test for statistical differences between categories, and Tukey's pair-wise comparison was used to identify which pairs of categories had statistically significant relationships. A P value of less than 0.05 was considered statistically significant. Retreatment rates and rehemorrhage rates are reported per patient year and were calculated by dividing the percent of patients with rehemorrhage or retreatment by the cumulative number of years of follow-up.

Patient Selection

The angioarchitecture of each aneurysm was the main influencing factor in patient selection. Particular attention was paid to the tortuosity of arteries that would require microcatheter navigation to gain access to the lesion. In general, the aneurysm with a relatively narrow inflow region (i.e., the neck) was considered a more suitable lesion for coil embolization than one with a larger neck. No HH Grade V patients were considered for treatment unless improvement occurred with ventricular decompression or other stabilizing maneuvers.

Aneurysms with large necks were made suitable for coil embolization using a series of technological advancements that progressed from balloon remodeling to the current stent technology (5, 11, 17, 22). In addition, inflow zones that initially appeared wide on digital subtraction angiography were actually found to be narrow on three-dimensional rotational angiography and lent themselves to coiling. The addition of three-dimensional rotational angiography imaging capabilities has reduced the degree of uncertainty of preoperative diagnostic angiography.

Technical Procedure

Coil Embolization and Angiographic Follow-up

Coil embolization procedures were performed with the patient under general endotracheal anesthesia in the Interventional

Neuroradiology Suite. All SAH patients with an HH grade of III or higher were monitored with ventriculostomy catheters, central venous catheters, and radial arterial lines to better optimize the medical management of vasospasm. During the endovascular procedure, patients were monitored with hemodynamic parameters as well as continuous electroencephalography, somatosensory evoked potentials, and brainstem auditory evoked responses.

The essential characteristic and therapeutic goal of the coil procedure is the induction of thrombosis within the aneurysm by the deployment of platinum microcoils. General cerebral angiographic and embolization techniques were used that have been outlined in other publications (5, 6, 22). For patients with SAH, heparinization was started once the dome of the aneurysm was protected. For unruptured aneurysms, heparinization was started at the beginning of the procedure. The activated clotting time is used as the index of the degree of anticoagulation and is considered satisfactory when it is two times the baseline. The end point of the intervention is complete angiographic elimination of the aneurysm fundus in multiple angiographic views, with full anticoagulation. Postoperatively, heparinization is maintained for 24 hours; in certain circumstances, heparinization is followed by dextran-40 for an additional 24 hours. In cases in which less than 95% occlusion is achieved, it is thought that cessation of anticoagulation therapy would lead to complete thrombosis of the aneurysm. This is exemplified by the following case.

Figure 1 demonstrates a 36-year-old woman with an unruptured dysplastic basilar trunk (BT) aneurysm projecting anteriorly (*Fig. 1, A* and *B*). This was treated with endovascular occlusion, achieving approximately 95% closure of the aneurysm (*Fig. 2, A* and *B*). Postoperatively, she remained neurologically intact and was discharged home on postoperative Day 2. She returned 7 months later for a follow-up angiogram, which revealed complete occlusion and, thus, the 5% residual at the end of the treatment had thrombosed (*Fig. 3, A* and *B*).

Follow-up selective cerebral angiography is performed at 6-month intervals for 2 years, after which noninvasive imaging (magnetic resonance imaging/magnetic resonance angiography and computed tomographic angiography) is used. The duration of follow-up imaging is determined on a case-by-case basis, with age being a prominent consideration. For a patient of advanced age, for example, it is less imperative to aggressively pursue follow-up angiography, in view of the relative risk for the older patient. Similarly, ambiguous or worrisome angiographic or radiological features at the time of or after treatment prompts earlier or more frequent angiographic follow-up examination. This is exemplified by the following case.

A 48-year-old woman with a 4- to 6-month history of intermittent vertigo, tinnitus, and intermittent ataxia underwent cerebral angiography, which confirmed a left posterior inferior cerebellar artery (PICA) aneurysm (*Fig. 4, A* and *B*). Subsequently, the aneurysm was treated with endovascular embolization, achieving 100% occlusion of the aneurysm (*Fig. 5, A* and *B*). Two days later, she was discharged home.

She returned for her 6-month follow-up angiogram, which demonstrated continued 100% occlusion (*Fig. 6, A* and *B*).

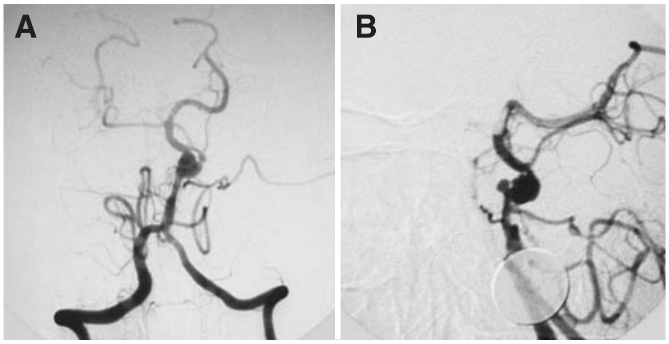

FIGURE 1. *Anteroposterior (A) and lateral (B) angiograms of a vertebral artery injection pre-embolization.*

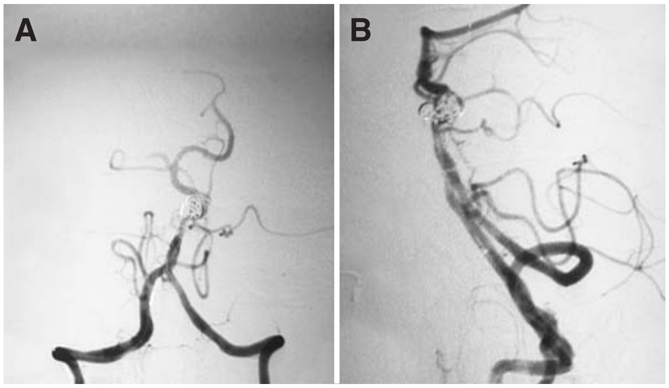

FIGURE 2. *Anteroposterior (A) and lateral (B) angiograms of a vertebral artery injection postembolization.*

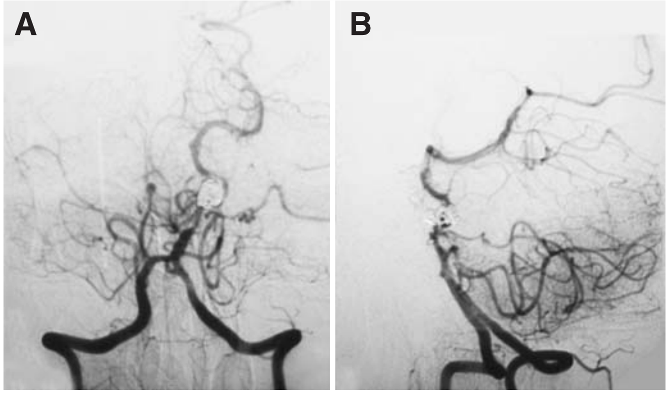

FIGURE 3. *Anteroposterior (A) and lateral (B) angiograms of a vertebral artery injection at the time of the 7-month follow-up examination.*

However, follow-up magnetic resonance imaging and magnetic resonance angiography at approximately 22 months was suspicious for recanalization. Therefore, she returned for a 23-month follow-up angiogram, which again revealed 100% occlusion of the aneurysm (*Fig. 7, A* and *B*).

Management of Vasospasm and Hydrocephalus

SAH patients with high transcranial Doppler velocities and neurological deficits not attributable to any definable causes

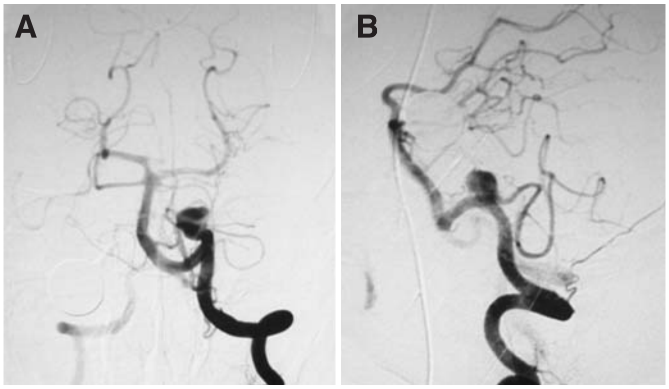

FIGURE 4. *Anteroposterior (A) and lateral (B) angiograms of a vertebral artery injection pre-embolization.*

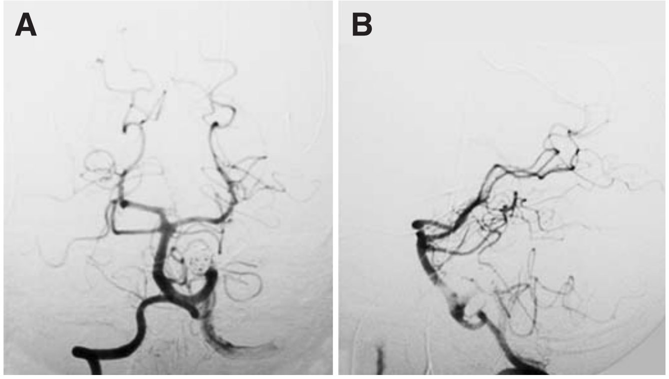

FIGURE 5. *Anteroposterior (A) and lateral (B) angiograms of a vertebral artery injection postembolization.*

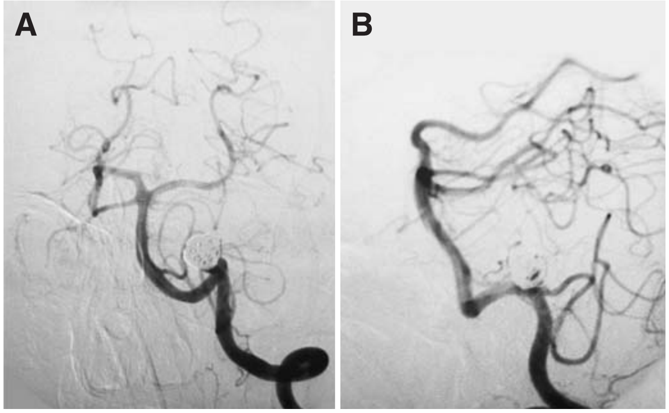

FIGURE 6. *Anteroposterior (A) and lateral (B) angiograms of a vertebral artery injection at the time of the 6-month follow-up examination.*

were started on prophylactic medical treatment, hypertension, hypervolemia, and hemodilution, for vasospasm (20). Those patients whose deficits did not improve with maximal medical management were evaluated with a head computed tomographic scan. Patients without a definable stroke underwent emergent cerebral angioplasty. In addition, all patients with

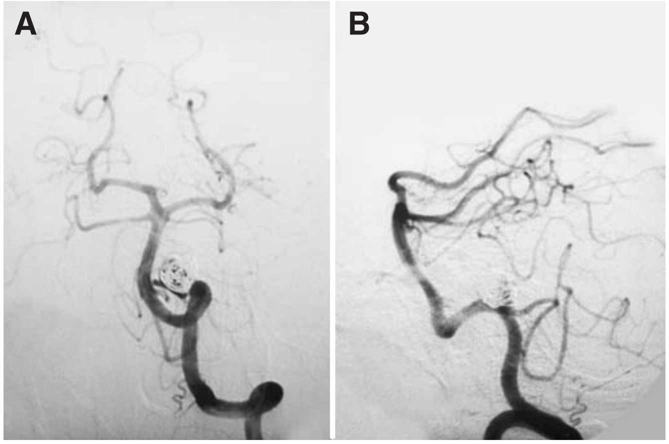

FIGURE 7. *Anteroposterior (A) and lateral (B) angiograms of a vertebral artery injection at the time of the 23-month follow-up examination.*

hydrocephalus and those with HH grades of III to V were managed with a ventriculostomy. After these patients were treated and were neurologically stable, they underwent a ventricular weaning protocol to determine whether or not they needed a permanent ventricular shunt.

RESULTS

Demographics, Aneurysmal Dome Size, and Clinical Presentation

Of 3227 patients with cerebral aneurysms treated by the Cerebrovascular Division between July 1995 and December 2005, 1877 patients (58.2%) were treated by coil embolization and 1350 patients (41.8%) were treated by craniotomy (usually clip ligation). Patients undergoing endovascular treatment for posterior circulation aneurysms (n = 275) accounted for 8.5% of the total aneurysm patient population and 14.7% of all aneurysms embolized at Jefferson Hospital for Neuroscience. Pertinent results presented within this article are summarized in *Table 1*.

The prevalence of posterior circulation aneurysms according to location was as follows: 189 cases (68.7%) of basilar apex (BA) aneurysms, 23 cases (8.4%) of BT aneurysms, 22 cases (8%) of superior cerebellar artery (SCA) aneurysms, and 41 cases (14.9%) of vertebral artery (Vert)/PICA aneurysms. Of all posterior circulation aneurysm patients, the mean age was 53.9 years (range, 7–90 yr); however, the mean age varied with aneurysm location: 55.6 years for BA cases, 56.2 years for SCA cases, 47 years for BT cases, and 49 years for Vert/PICA cases. The mean age of patients harboring BA and SCA aneurysms was significantly older than patients harboring BT and Vert/PICA aneurysms ($P = 0.0007$). The mean dome sizes as measured by the operating neurosurgeon were as follows: 7.7 mm for all cases, 8 mm for BA cases, 6.9 mm for SCA cases, 6.2 mm for BT cases, and 7.9 mm for Vert/PICA cases. Dome size as a function of location did not vary significantly ($P = 0.4077$).

Elective patients were a significant proportion of the cases included and were distributed as follows: 106 (38.5%) of all

TABLE 1. Data summary of patients harboring posterior circulation aneurysms[a]

	Total cases	BA	SCA	BT	Vert/PICA
No. of patients	275	189	22	23	41
HH Grade 0 Patients (elective)	38.5%	38.1%	27.3%	43.5%	43.9%
HH Grade I or II patients	21.5%	22.2%	18.2%	13%	24.4%
HH Grade III or IV patients	40%	39.7%	54.5%	43.5%	31.7%
Mean age (yr)	53.9	55.6	56.2	47	49
Mean dome size (mm)	7.7	8	6.9	6.2	7.9
Achieving 95–100% embolization	87.8%	89%	81%	91%	82.9%
Achieving mGOS I	87.4%	89.5%	76.2%	87%	84.6%
>10% recurrence	19.6%	11.4%	29.4%	5.9%	14.7%
Retreatment rate	4.9%	4.9%	4.8%	0%	4.9%
Retreatment rate in patients with <90% initial embolization	60%	100%	0%	0%	50%
Rehemorrhage rate	1.1%	0.06%	4.8%	0%	2.4%
Requiring angioplasty	6.5%	6.8%	6.3%	7.7%	4.3%
Requiring VPS	71%	71%	50%	69.2%	47.8%
Complications	5.8%	5.3%	4.5%	4.3%	4.8%

[a] BA, basilar apex; SCA, superior cerebellar artery; BT, basilar trunk; Vert/PICA, vertebral artery or posterior inferior cerebellar artery; HH, Hunt and Hess; mGOS, modified Glasgow Outcome Scale; VPS, ventriculoperitoneal shunt.

patients included in this study, 72 (38.1%) patients in the BA group, six (27%) patients in the SCA group, 10 (43.5%) patients in the BT group, and 18 (43.7%) patients in the Vert/PICA group. The differences in the distribution of elective patients according to location did not reach statistical significance ($P = 0.5910$). Of SAH patients, HH Grades I and II were present in the following proportions of patients according to location: 59 (21.4%) of all patients included in this study, 42 (22.2%) patients in the BA group, four (18%) patients in the SCA group, three (13%) patients in the BT group, and 10 (24.4%) patients in the Vert/PICA group. Lower HH grade (Grades III and IV) patients were present as follows: 110 (40.1%) of all patients included in the study, 75 (39.6%) patients in the BA group, 12 (55%) patients in the SCA group, 10 (43.4%) patients in the BT group, and 13 (31.7%) patients in the Vert/PICA group. As was true with elective patients, differences in the incidence of lower or higher HH grade patients according to location was not statistically significant ($P = 0.5102$).

Successful Embolization and the Use of Stent Technology

The degree of aneurysmal dome occlusion was determined by the operating neurosurgeon and was obtained from the dictated operative reports. Successful embolization was deemed as 95 to 100% occlusion of the dome. Patients with 95 to 100% occlusion were distributed as follows: 237 (87.8%) of all patients included in the present study, 166 (89%) patients in the BA group, 17 (81%) patients in the SCA group, 20 (91%) patients in the BT group, and 34 (82.9%) patients in the Vert/PICA group.

Aneurysmal dome occlusions of 90 to 95% were considered successful but incomplete, and its prevalence was as follows: 28 (10%) of all patients in the study, 18 (9.7%) patients in the BA group, four (19%) patients in the SCA group, one (4.5%) patient in the BT group, and five (12.2%) patients in the Vert/PICA group. Dome occlusions of less than 90% were considered protective but incomplete and were present as follows: five (1.8%) patients in this study, two (1.1%) patients in the BA group, no patients in the SCA group, one (4.5%) patient in the BT group, and two (4.9%) patients in the Vert/PICA group. There were no significant relationships between the degree of occlusion and location of aneurysm ($P = 0.4152$)

Those aneurysms with a dome-to-neck ratio that did not favor endovascular therapy were made more suitable for the procedure with the use of a Neuroform stent (Boston Scientific/Target, Fremont, CA) (7). The relative incidences of stent requirement according to location were as follows: 38 cases (13.8%) in the entire series, 23 cases (12.2%) in the BA group, two cases (9.1%) in the SCA group, six cases (26.1%) in the BT group, and seven cases (17%) in the Vert/PICA group. Although a greater proportion of patients with BT aneurysms required stent-assisted coiling, the differences in the rate of stent use according to location was not significant ($P = 0.2651$).

Clinical Outcome

Clinical outcome using mGOS was present for 262 (95.3%) of the 275 patients. The duration of follow-up was 31.8 months for cases performed before 2004 and 13.4 months for cases performed during 2004 and 2005. Independent functioning (mGOS I and II) was achieved by the following proportions of patients: 241 (92%) patients in the study, 169 (93.4%) patients in the BA group, 16 (76%) patients in the SCA group, 20 (95%) patients in the BT group, and 36 (87.8%) patients in the Vert/PICA group. The prevalence of poor outcome (mGOS III and IV) was as follows: 10 (3.8%) patients in the study, five (2.8%) patients in the

BA group, two (9%) patients in the SCA group, one (5%) patient in the BT group, and two (4.9%) patients in the Vert/PICA group. Eleven patients (4.9%) were deceased (mGOS V) and were distributed according to location as follows: seven (3.9%) in the BA group, three (15%) in the SCA group, none in the BT group, and one (2.4%) in the Vert/PICA group. Outcome as a function of location did not vary significantly ($P = 0.1486$).

Clinical outcome was also analyzed according to clinical presentation of the patient (elective versus SAH patients). Comparing elective versus SAH patients, those achieving independence (mGOS I or II) were as follows: 95.4 and 70% of all elective and SAH cases, respectively; 98.6 and 82.9% of all elective and SAH BA cases, respectively; 100 and 58% of all elective and SAH SCA cases, respectively; 88.9 and 66.6% of all elective and SAH BT cases, respectively; and 94 and 73.9% of all elective and SAH Vert/PICA cases, respectively. Patients in the elective group did significantly better than patients in the SAH group ($P = 0.0163$).

Angiographic Follow-up

Angiographic follow-up data was obtained for 224 (84.8%) of the 264 surviving patients. The mean duration of follow-up was 31.3 months for cases performed before 2004 and was 31.3 and 13.7 months for cases performed during 2004 and 2005, respectively. Recurrence was divided into three categories: less than 5%, clinically insignificant and requiring only conservative management; 5 to 10%, significant and requiring continued angiographic evaluation; and greater than 10%, clinically significant and raising the possibility of retreatment. When evaluated according to location, significant recurrence (>5%) was present in the following proportion of patients: 55 patients (24.5%) in this series, 37 patients (23.7%) in the BA group, six patients (35.3%) in the SCA group, three (16.1%) patients in the BT group, and nine patients (26.5%) in the Vert/PICA group. Although the recurrence rate varied as a function of location, the differences did not reach significance ($P = 0.3070$).

Recurrence was also evaluated as a function of initial degree of aneurysmal occlusion. Those patients who had greater than 95% occlusion (complete) on initial coiling were compared with those who had less than 90% occlusion (incomplete). The prevalence of clinically significant recurrence (>5%) within these two groups is as follows: 15.2 and 54.5% of all complete and incomplete cases presented in this series, respectively; 11.4 and 55.5% in the complete and incomplete BA cases, respectively; 23.5 and 50% in the complete and incomplete SCA cases, respectively; 13.3 and 50% in the complete and incomplete BT cases, respectively; and 18.8 and 43% in the complete and incomplete Vert/PICA cases, respectively. As evident, clinically significant recurrence was more common in incompletely coiled aneurysms compared with completely occluded aneurysms ($P < 0.0001$).

Retreatment

Whether elective or presenting with SAH, patients who were initially treated at the Jefferson Hospital for Neuroscience and then required recoiling or clip ligation of the initially treated aneurysm comprised the retreatment group. The retreatment rate according to location was as follows: 4.9% of all patients in this series, 4.9% of all patients in the BA group, 4.8% of all pateints in the SCA group, 0% of patients in the BT group, and 4.9% of patients in the Vert/PICA group. The rate of recoiling as a function of location did not vary significantly ($P = 0.6506$). The overall retreatment rate per year of follow-up study was 0.01%.

Rehemorrhage Rate

Patients were included in the rehemorrhage category if they presented with aneurysmal SAH from a previously coiled posterior circulation aneurysm. The prevalence of rehemorrhage as a function of location was as follows: 1.1% of all patients reported in this series, 0.06% of all BA patients, 4.8% of all SCA patients, 0% of all BT patients, and 2.4% of all patients in the Vert/PICA group. Although the rates varied according to location, differences were not statistically significant ($P = 0.6070$). Of the three patients who presented with recurrent SAH attributable to the previously coiled aneurysm, two had initially presented with SAH, whereas the other patient was an elective patient who had significant recurrence. All of the rehemorrhage patients had greater than 10% recurrence at a mean angiographic follow-up period of 11 months. The overall rehemorrhage rate per year of follow-up study is 0.003%.

Clinically Significant Vasospasm Requiring Cerebral Angioplasty

Those SAH patients who developed clinically significant vasospasm refractory to medical therapy were included in this group. The proportion of patients requiring cerebral angioplasty as a function of location was as follows: 6.5% of all patients in this series, 6.8% of all patients in the BA group, 6.3% of all patients in the SCA group, 7.7% of all patients in the BT group, and 4.3% of all patients in the Vert/PICA group. The incidence of vasospasm varied according to location; however, there were no significant differences ($P = 0.9306$).

Incidence of Ventricular Shunts

Included in this group were SAH patients who failed ventricular weaning and required permanent placement of a ventricular shunt. The prevalence of ventricular shunts in the SAH patient population as a function of location was as follows: 71% of all SAH cases in this series, 71% of SAH patients in the BA group, 50% of SAH patients in the SCA group, 69.2% of SAH patients in the BT group, and 47.8% of all patients in the Vert/PICA group. The difference in the incidence of shunt requirement as a function of aneurysmal location was not significant ($P = 0.8497$).

Complications

Complications related to treatment were organized temporally into early, late, and delayed. Early complications were those that occurred within 48 hours of treatment, and included six patients (2.2%) with intraoperative rupture, one patient (0.4%) with clinically significant catheter-induced vasospasm, and five patients (1.8%) with clinically insignificant prolapse of

coils within the parent vessel. The overall immediate complication rate was 3.6%. Of the six patients experiencing intraoperative rupture, five achieved independence and one died. The patient who experienced catheter-induced vasospasm also attained mGOS I. Patients with prolapsed coils did not experience any thromboembolic events or other morbidities related to the coiling procedure. Early complications and clinical outcomes are summarized in *Table 2*.

Late complications (*Table 3*) were those that occurred 48 hours after treatment but before the patient was discharged; these included six patients (2.2%) with cerebral ischemia. Of these six patients, four had deterioration in clinical functioning caused by the ischemic event. Delayed complications (*Table 4*) were those that occurred after discharge and included those patients who required retreatment or presented with a rehemorrhage. These included eight patients (2.9%) who required retreatment and three patients (1.1%) who experienced rehemorrhage. The overall delayed complication rate was 4%. When evaluating complications led to morbidity, complication rates as a function of location were as follows: 5.1% of all patients in this series, 4.8% of BA patients, 4.5% of SCA patients, 4.3% of BT patients, and 9.8% of Vert/PICA patients. There was no significant relationship between the complication rate and location of aneurysm ($P = 0.6416$). Our overall complication rate of 5.1% correlates well with other series reporting complication rates between 3 and 9.5% (27, 28, 32).

DISCUSSION

Demographics and Clinical Presentation

Endovascular treatment for ruptured and unruptured posterior circulation aneurysms has been known to be efficacious; however, most series to date have not commented on the long-term durability. In a retrospective analysis covering 10 years and representing one of the largest series of its kind, we report our angiographic and clinical outcomes of patients undergoing endovascular treatment of ruptured and unruptured posterior circulation aneurysms at the Jefferson Hospital for Neuroscience.

Our posterior circulation patient population represented nearly 10% of all aneurysm patients treated at the Jefferson Hospital for Neuroscience. Posterior circulation aneurysms were further divided by location and were assigned to either the BA group, the SCA group, the BT group, or the Vert/PICA group. As has been previously documented by other series, the most common location for posterior circulation aneurysms was the BA, which represented approximately 69% of our series. Our demographic characteristics of a mean age of 53.9 years and a male-to-female patient ratio of 1:2 is representative of other similar series (27, 28). Of note, patients in the BA and SCA groups (mean age, 55.6 and 56.2 yr, respectively) were significantly older than patients in the BT and Vert/PICA groups (47 and 49 yr, respectively) ($P = 0.0007$). This could indicate that patients harboring Vert/PICA or BT aneurysms become symptomatic at a younger

TABLE 2. Early complications and outcome[a]

Age (yr)/sex	HH Grade	Aneurysmal location	Complication	Treatment	Outcome (mGOS)
37/F	I	BA	Catheter-induced spasm	Cerebral angioplasty	I
57/F	0	BA	Intraop rupture	Ventric; pentobarb	I
50/M	I	BA	Intraop rupture	Ventric; pentobarb	I
38/F	0	BT	Intraop rupture	Ventric; pentobarb	I
34/F	II	PICA	Intraop rupture	Ventric; pentobarb	I
43/F	I	BA	Intraop rupture	Ventric; pentobarb	II
90/F	III	BA	Intraop rupture	Ventric; pentobarb	V

[a] HH, Hunt and Hess; mGOS, modified Glasgow Outcome Scale; BA, basilar apex; intraop, intraoperative; ventric, ventriculostomy; pentobarb, pentobarbital; BT, basilar trunk; PICA, posterior inferior cerebellar artery.

TABLE 3. Late complications and outcome[a]

Age (yr)/sex	HH Grade	Aneurysmal location	Complication	Treatment	Outcome (mGOS)
62/F	0	BA	Ischemia: cerebral peduncle	Anticoagulation	I
70/F	I	PICA	Ischemia: cerebellum	Anticoagulation	I
47/F	0	BA	Ischemia: occipital lobe	Anticoagulation	I
58/F	0	BA	Ischemia: occipital lobe	Anticoagulation	I
58/F	IV	BA	Ischemia: temporal lobe	Anticoagulation	II
62/F	0	BA	Ischemia: cerebral peduncle	Anticoagulation	III

[a] HH, Hunt and Hess; mGOS, modified Glasgow Outcome Scale; BA, basilar apex; PICA, posterior inferior cerebellar artery.

TABLE 4. Delayed complications and outcome[a]

Age (yr)/sex	HH Grade	Aneurysmal location	Complication	Treatment	Outcome (mGOS)
63/M	III	BA	Rehemorrhage	Recoiled	I
58/F	III	SCA	Rehemorrhage	Recoiled	V
62/M	II	Vert/PICA	Rehemorrhage	Clipped	V

[a] HH, Hunt and Hess; mGOS, modified Glasgow Outcome Scale; BA, basilar apex; SCA, superior cerebellar artery; Vert/PICA, vertebral artery or posterior inferior cerebellar artery.

age. Nonetheless, the difference in age according to location was only significant when evaluating elective patients (P = 0.0193), and the relationship was lost when comparing SAH patients (P = 0.0930). Usually, patients with asymptomatic aneurysms are discovered via evaluation of other conditions, such as headache, transient ischemic attack, stroke, family history, or traumatic head injury. It would be interesting to evaluate the incidence of these conditions among patients with Vert/PICA and BT aneurysms compared with patients with BA and SCA aneurysms.

Elective patients comprised nearly 40% of all cases within this series and the incidence was not significantly different when compared as a function of location (P = 0.5910). Patients with benign (HH Grade I or II) or poor (HH Grade III or IV) SAH presentations represented approximately 20 and 40% of the cases, respectively (21). HH grade presentation, when evaluated as a function of location, did not show significant differences (P = 0.4956) (21).

Clinical Outcome

In a review by Lozier et al. (28) comparing 21 different studies related to endovascular management of posterior circulation aneurysms, 70 to 91% of patients achieved independence. This correlates well with our own series because we report 85% of patients achieving independence (mGOS I or II). The fraction of patients achieving independence did not vary with location (P = 0.1898); however, there was a significant relationship between HH grade and clinical outcome (P = 0.0015). This was evident because 95.4% of elective patients achieved functional independence, whereas only 70% of SAH patients achieved functional independence.

Nearly 4% of the patients in this series were in the poor outcome category (mGOS III or IV). The incidence of poor outcome as a function of location did not vary significantly (P = 0.1898). Eleven patients (4.9%) died and the incidence did not vary significantly according to location (P = 0.1486). Mortality rates within other series have ranged from 5 to 11% and are correlated with presenting HH grade (28).

Multiple clinical factors (age, sex, location, aneurysmal size, presence of shunt, initial occlusion, and coil type) were evaluated for their clinical significance in predicting outcome (*Table 5*). The only significant predictors of good clinical outcome (mGOS I or II) were presenting HH grade (P [lt] 0.0001) and the absence of shunt (P = 0.0015). Although presenting HH grade is well documented as a predictor of clinical outcome, the absence of a shunt should portend a better outcome because ventricular drains are placed in patients with higher HH grades.

TABLE 5. Predictors of clinical outcome[a]

Variable	Standard error	P value
Age	0.5009	0.4826
Sex	0.3278	0.2417
Location	0.4803	0.4936
Size	0.2969	0.9657
HH Grade	0.4129	0.0001
Coil type	0.3655	0.7140
Absence of shunt	0.3018	0.0015

[a] HH, Hunt and Hess.

Angiographic Outcome: Initial Occlusion and Recurrence Intimately Related

Aneurysmal architecture plays a significant role in the success of endovascular therapy because those aneurysms with a narrow inflow zone are more favorable for coiling. The mean dome size of our cases was 7.7 mm (range, 3–25 mm) and there were no significant relationships between dome size and location or dome size and presenting HH grade (P = 0.4077 and 0.4566, respectively). Aneurysms with wide necks were made more suitable for coiling with the aid of Neuroform stents. The overall rate of stent use was 13.8% and was most commonly used with BT aneurysms (26.1% of cases.) This is to be expected because most BT aneurysms have wide necks and, thus, require stent-assisted coiling for stabilization of the coil mass and prevention of coil prolapse into the parent vessel. However, no significant relationships were derived between the incidence of stent use as a function of location or between the use of stent and clinical outcome (P = 0.2651 and 0.5797, respectively).

The majority (87.8%) of patients in our series were successfully coiled, as represented by greater than 95% occlusion (complete occlusion) of the aneurysmal dome; only 1.8% of the patients had less than 90% occlusion (incomplete occlusion) of the dome. The degree of initial occlusion was not related to aneurysmal location and did not correlate with the clinical outcome of patients (P = 0.4152 and 0.5500, respectively). Nonetheless, there was a strong relationship between the degree

of initial occlusion and subsequent recurrence. Clinically significant recurrence (>5%) was observed in 15.2% of patients with complete occlusion and was observed in 54.5% of those who had incomplete occlusion ($P < 0.0001$). This relationship is clinically evident because incompletely occluded aneurysms continue to have the presence of flow, which prevents thrombus organization and intra-aneurysmal scarring, factors that would prevent recurrence. The degree of recurrence was not related to aneurysmal location or clinical outcome ($P = 0.3070$).

Incidence of Shunts

Those patients with hydrocephalus or high HH grades (Grades III–V) are managed with a ventricular drain. Over time, the ventricular drain is weaned and those who are unable to tolerate the weaning process receive ventricular shunts. The overall shunt rate among the SAH patients within this series was approximately 71% and did not differ significantly when the incidence was compared in relation with location. We are unable to compare this data with other series because, to our knowledge, the rate of ventricular shunts is not reported in other published studies of posterior circulation aneurysms. Nonetheless, when comparing with the prevalence of shunts in our own series of anterior circulation aneurysms (26.5%, unpublished data), shunts were much more common in the posterior circulation patient population as compared with the anterior circulation patient population ($P < 0.0001$). Dorai et al. (10) support our findings because they report location (posterior circulation) as a risk factor of needing a shunt in patients with aneurysmal SAH (10, 19, 37). This most likely relates to the fact that most posterior circulation aneurysms present with the presence of blood within the basal cisterns and the fourth ventricle, leading to obstructive or communicating hydrocephalus and, therefore, the need for ventricular drainage. Future studies relating Fisher grade and the need for ventricular shunts will be essential in deciphering whether or not a greater proportion of patients harboring posterior circulation aneurysms present with higher Fisher grades.

Medically Refractory Vasospasm

Cerebral vasospasm is the leading cause of morbidity among surviving SAH patients. Those patients with vasospasm who fail medical management can benefit from emergent cerebral angioplasty, with the only exclusion criteria being the presence of an infarct on imaging studies (unpublished data). The incidence of medically refractory vasospasm requiring cerebral angioplasty was approximately 6.5% and did not vary significantly according to location ($P = 0.9306$). The lower incidence of clinically significant vasospasm in patients harboring posterior circulation aneurysms has been previously reported by Hirashima et al. (19). A possible hypothesis is that there is greater collateral flow within the posterior fossa, thus, even significant vasospasm would not lead to symptomatology (19). In addition, it will be important to evaluate the Fisher grade of patients to correlate differences in Fisher grade when comparing anterior and posterior circulation patients.

Complications

Complications attributable to endovascular therapy varied from the clinically silent prolapse of coils and asymptomatic ischemic events, to the clinically devastating intraoperative aneurysmal rupture, large posterior circulation infarcts, and recurrent hemorrhages from previously treated aneurysms.

Coil Prolapse

Those patients with prolapsed coil fragments within the parent vessel (n = 5, 1.8% of the patients in the series) were continued on heparin therapy for 24 hours and then subsequently switched to Plavix (Bristol-Myers Squibb/Sanofi Pharmaceuticals, New York, NY) for 6 weeks. The goal was to provide anticoagulation until the protruding coil would have undergone endothelialization. All of these cases were asymptomatic and did not lead to permanent morbidity or mortality.

Thromboembolic Events

Thromboembolic events are a well-documented complication of endovascular therapy because thrombogenic coils are introduced into the host's circulatory system. Previously published series have indicated rates of thromboembolism as approximately 1 to 6% (28). In our series, six patients (2.2%) experienced ischemic events related to the coiling procedure. Four (66.7%) of the six patients had clinical changes associated with the stroke, ranging from hemiparesis and ataxia to visual field defects. Once the possibility of ischemia related to the coiling procedure is raised, the patient is emergently imaged to exclude hemorrhage as a cause of the clinical changes. If there are no contraindications, the patient is reheparinized for 24 to 48 hours and subsequently switched to Plavix for 6 to 8 weeks. Once again, the goal is to allow for endothelialization across the neck of the aneurysm, thus, excluding the thrombogenic coils from the parent vessel circulation and, therefore, reducing the risk of thromboembolism.

Intraoperative Rupture

Intraoperative rupture is a serious risk during endovascular and/or microsurgical therapy of cerebral aneurysms, leading to devastating morbidity and mortality. During endovascular therapy, intraoperative rupture can lead to irreversible cerebral damage caused by the immense rise in intracranial pressure. This is not the scenario in microsurgical therapy, in which the brain is open to the atmosphere and, thus, aneurysmal rupture does not affect the intracranial pressure. When extravasation is suspected during a coiling procedure, the coiling procedure continues to allow for protection of the dome while the patient is administered protamine to reverse heparinization, and/or platelet transfusion to reverse the effects of antiplatelet therapeutics. In addition, the patient is immediately placed under burst suppression with the use of pentobarbital. Subsequently, the patient is emergently imaged to evaluate the presence of intracerebral hematomas and/or hydrocephalus. A ventriculostomy is placed to monitor cerebral pressure because the patient will be in burst suppression. When intraoperative rup-

TABLE 6. Endovascular treatment of posterior circulation aneurysms: Analysis of previously published series[a]

Series (ref. no.)	Patients	Completely occluded	≤95% occlusion	Recanalization	Outcome: excellent/good
Pandey (current study)	275	87.8% (≥95% occlusion)	12.2% (<95% occlusion)	24.5%	87.4%
Eskridge and Song (14)	150	75% (>90% occlusion)	NA	NA	75%
Lempert et al. (27)	112	54% (99–100% occlusion)	6% (<90% occlusion)	22.4%	83%
UCSF	107	54% (99–100% occlusion)	22%	22.4%	83%
Bavinzski et al. (3)	45	54%	13%	NA	69%
Pierot et al. (33)	35	62%	38%	14%	91%
Raymond and Roy (34)	31	42%	6%	23%	61%
Gruber et al. (15)	21	66% (>95%)	24%	19%	90%

[a] NA, not applicable; UCSF, University of California, San Francisco.

ture occurs in an elective patient, the patient is treated as an SAH patient.

When comparing multiple series on endovascular therapy of posterior circulation aneurysms, Lozier et al. (28) quote an intraoperative rupture rate of approximately 2 to 3% (9, 28). In our series, intraoperative rupture occurred in six patients (2.2%), of whom one patient was an elective patient and the others were SAH patients. Five (83.3%) of these six patients achieved independence on clinical follow-up. The other patient had presented with a HH Grade III SAH, did not subsequently recover, and had withdrawal of supportive measures according to the wishes of the family. Although intraoperative rupture prolonged hospitalization time, it did not prevent our patients from achieving independence in majority of the cases (83.3%).

Rehemorrhage from Previously Coiled Aneurysms

Endovascular therapy, although feasible with acceptable risks, poses a great challenge regarding its long-term durability. Recurrence, the measure of durability, leads to further angiographic evaluation and treatment, thus, incurring risk to the patient and a cost to society. More importantly, the clinical significance of recurrence can be evaluated with its relationship with rehemorrhage.

We had three patients who presented with recurrent SAH. Two of these patients were previous SAH patients, and the other was an elective patient with significant recurrence. Recurrence played a significant role in rehemorrhage because all of these patients had greater than 10% recurrence at a mean angiographic follow-up period of 11 months. Thus, of the 44 patients who had greater than 10% recurrence, three (6.8%) went on to present with recurrent SAH. Among the patients with greater than 10% recurrence, there was a follow-up time of 108.25 years; thus, the yearly rehemorrhage rate among patients with greater than 10% recurrence is 0.063%. Initial occlusion was not related to recurrent SAH because only one of the three rehemorrhage patients had less than 90% occlusion at baseline. In addition, clinical outcome among the rehemorrhage patients was grave because two of the three patients died. This was correlated with the fact that the rehemorrhage patients had poor HH presentation because two of the three patients were categorized as HH Grade IV. As exemplified, recurrence within our series is a predictor of recurrent SAH and, thus, intimately related to patient morbidity and mortality.

CONCLUSION

Although this series is large with a long follow-up period, it is limited by its retrospective methodology. Although the ultimate goal of aneurysm treatment is permanent obliteration of the aneurysm, this goal is not always achieved by endovascular therapy, as evident in nearly 15% of our cases. A protective effect of treatment that does not result in complete elimination may result from partial coil embolization, despite the measurable risk of regrowth and rupture of aneurysmal remnants (9, 12, 27, 28). This protective effect of partial embolization is also supported by the results of the International Subarachnoid Hemorrhage Trial (29).

Although endovascular therapy leads to approximately 90% of patients achieving independence (mGOS I or II), the durability of the treatment remains a question (*Table 6*). With nearly a 3-year follow-up period, we report a 24.5% incidence of clinically significant recurrence (>5%). Our data suggest that recurrence is related to initial embolization because patients with less than 90% occlusion go on to develop clinically significant recurrence in nearly 60% of the cases. In addition, all of the recurrent hemorrhages occurred in patients with greater than 10% recurrence. Although endovascular therapy is an essential modality of treatment for patients harboring aneurysms, the main challenge continues to be unacceptable rates of recurrence and the associated risks. We look forward to the development of new bioactive coil technology toward the goal of permanent occlusion of aneurysms and the elimination of recurrences and all associated risks (15, 26).

REFERENCES

1. Arat A, Islak C, Saatci I, Kocer N, Cekirge S: Endovascular parent artery occlusion in large-giant or fusiform distal posterior cerebral artery aneurysms. **Neuroradiology** 44:700–705, 2002.
2. Batjer HH, Samson DS: Causes of morbidity and mortality from surgery of aneurysms of the distal basilar artery. **Neurosurgery** 25:904–916, 1989.

3. Bavinzski G, Richling B, Gruber A, Killer M, Levy D: Endosaccular occlusion of basilar artery bifurcation aneurysms using electrically detachable coils. **Acta Neurochir (Wien)** 134:184–189, 1995.
4. Bendok BR, Przybylo JH, Parkinson R, Hu Y, Awad IA, Batjer HH: Neuroendovascular interventions for intracranial posterior circulation disease via the transradial approach: Technical case report. **Neurosurgery** 56:E626, 2005.
5. Benitez RP, Silva MT, Klem J, Veznedaroglu E, Rosenwasser RH: Endovascular occlusion of wide-necked aneurysms with a new intracranial microstent (Neuroform) and detachable coils. **Neurosurgery** 54:1359–1368, 2004.
6. Birknes JK, Hwang SK, Pandey AS, Cockroft K, Dyer AM, Benitez RP, Veznedaroglu E, Rosenwasser RH: Feasibility and limitations of endovascular coil embolization of anterior communicating artery aneurysms: Morphological considerations. **Neurosurgery** 59:43–52, 2006.
7. Cockroft KM, Marks MP, Steinberg GK: Planned direct dual-modality treatment of complex broad-necked intracranial aneurysms: Four technical case reports. **Neurosurgery** 46:226–231, 2000.
8. Czepko R, Libionka W, Turski T, Uhl H, Pietraszko W: Mechanically detachable coils in the treatment of posterior circulation aneurysms: A comparison with surgical repair [in Polish]. **Przegl Lek** 62:24–28, 2005.
9. David CA, Vishteh AG, Spetzler RF, Lemole M, Lawton MT, Partovi S: Late angiographic follow-up review of surgically treated aneurysms. **J Neurosurg** 91:396–401, 1999.
10. Dorai Z, Hynan LS, Kopitnik TA, Samson D: Factors related to hydrocephalus after aneurysmal subarachnoid hemorrhage. **Neurosurgery** 52:763–771, 2003.
11. dos Santos Souza MP, Agid R, Willinsky RA, Cusimano M, Montanera W, Wallace MC, terBrugge KG, Marotta TR: Microstent-assisted coiling for wide-necked intracranial aneurysms. **Can J Neurol Sci** 32:71–81, 2005.
12. Drake CG, Friedman AH, Peerless SJ: Failed aneurysm surgery. Reoperation in 115 cases. **J Neurosurg** 61:848–856, 1984.
13. Drake CG, Peerless SJ: Giant fusiform intracranial aneurysms: Review of 120 patients treated surgically from 1965 to 1992. **J Neurosurg** 87:141–162, 1997.
14. Eskridge JM, Song JK: Endovascular embolization of 150 basilar tip aneurysms with Guglielmi detachable coils: Results of the Food and Drug Administration multicenter clinical trial. **J Neurosurg** 89:81–86, 1998.
15. Gruber DP, Zimmerman GA, Tomsick TA, van Loveren HR, Link MJ, Tew JM Jr: A comparison between endovascular and surgical management of basilar artery apex aneurysms. **J Neurosurg** 90:868–874, 1999.
16. Guglielmi G, Viñuela F, Dion J, Duckwiler G: Electrothrombosis of saccular aneurysms via endovascular approach. Part 2: Preliminary clinical experience. **J Neurosurg** 75:8–14, 1991.
17. Higashida RT, Smith W, Gress D, Urwin R, Dowd CF, Balousek PA, Halbach VV: Intravascular stent and endovascular coil placement for a ruptured fusiform aneurysm of the basilar artery. Case report and review of the literature. **J Neurosurg** 87:944–949, 1997.
18. Hillman J, Säveland H, Jakobsson KE, Edner G, Zygmunt S, Fridriksson S, Brandt L: Overall management outcome of ruptured posterior fossa aneurysms. **J Neurosurg** 85:33–38, 1996.
19. Hirashima Y, Kurimoto M, Hori E, Origasa H, Endo S: Lower incidence of symptomatic vasospasm after subarachnoid hemorrhage owing to ruptured vertebrobasilar aneurysms. **Neurosurgery** 57:1110–1116, 2005.
20. Hoh BL, Topcuoglu MA, Singhal AB, Pryor JC, Rabinov JD, Rordorf GA, Carter BS, Ogilvy CS: Effect of clipping, craniotomy, or intravascular coiling on cerebral vasospasm and patient outcome after aneurysmal subarachnoid hemorrhage. **Neurosurgery** 55:779–789, 2004.
21. Hunt WE, Hess RM: Surgical risk as related to time of intervention in the repair of intracranial aneurysms. **J Neurosurg** 28:14–20, 1968.
22. Jabbour P, Koebbe C, Veznedaroglu E, Benitez RP, Rosenwasser R: Stent-assisted coil placement for unruptured cerebral aneurysms. **Neurosurg Focus** 17:E10, 2004.
23. Kassell NF, Torner JC, Haley EC Jr, Jane JA, Adams HP, Kongable GL: The International Cooperative Study on the Timing of Aneurysm Surgery. Part 1: Overall management results. **J Neurosurg** 73:18–36, 1990.
24. Kassell NF, Torner JC, Jane JA, Haley EC Jr, Adams HP: The International Cooperative Study on the Timing of Aneurysm Surgery. Part 2: Surgical results. **J Neurosurg** 73:37–47, 1990.
25. Klein GE, Szolar DH, Leber KA, Karaic R, Hausegger KA: Basilar tip aneurysm: Endovascular treatment with Guglielmi detachable coils—Midterm results. **Radiology** 205:191–196, 1997.
26. Leber KA, Klein GE, Trummer M, Eder HG: Intracranial aneurysms: A review of endovascular and surgical treatment in 248 patients. **Minim Invasive Neurosurg** 41:81–85, 1998.
27. Lempert TE, Malek AM, Halbach VV, Phatouros CC, Meyers PM, Dowd CF, Higashida RT: Endovascular treatment of ruptured posterior circulation cerebral aneurysms. Clinical and angiographic outcomes. **Stroke** 31:100–110, 2000.
28. Lozier AP, Connolly ES Jr, Lavine SD, Solomon RA: Guglielmi detachable coil embolization of posterior circulation aneurysms: A systematic review of the literature. **Stroke** 33:2509–2518, 2002.
29. Molyneux AJ, Kerr RS, Yu LM, Clarke M, Sneade M, Yarnold JA, Sandercock P; International Subarachnoid Aneurysm Trial (ISAT) Collaborative Group: International subarachnoid aneurysm trial (ISAT) of neurosurgical clipping versus endovascular coiling in 2143 patients with ruptured intracranial aneurysms: A randomised comparison of effects on survival, dependency, seizures, rebleeding, subgroups, and aneurysm occlusion. **Lancet** 366:809–817, 2005.
30. Mordasini P, Schroth G, Guzman R, Barth A, Seiler RW, Remonda L: Endovascular treatment of posterior circulation cerebral aneurysms by using Guglielmi detachable coils: A 10-year single-center experience with special regard to technical development. **AJNR Am J Neuroradiol** 26:1732–1738, 2005.
31. Nichols DA, Brown RD Jr, Thielen KR, Meyer FB, Atkinson JL, Piepgras DG: Endovascular treatment of ruptured posterior circulation aneurysms using electrolytically detachable coils. **J Neurosurg** 87:374–380, 1997.
32. Ogilvy CS, Hoh BL, Singer RJ, Putman CM: Clinical and radiographic outcome in the management of posterior circulation aneurysms by use of direct surgical or endovascular techniques. **Neurosurgery** 51:14–22, 2002.
33. Pierot L, Boulin A, Castaings L, Rey A, Moret J: Selective occlusion of basilar artery aneurysms using controlled detachable coils: Report of 35 cases. **Neurosurgery** 38:948–954, 1996.
34. Raymond J, Roy D: Safety and efficacy of endovascular treatment of acutely ruptured aneurysms. **Neurosurgery** 41:1235–1246, 1997.
35. Samson D, Batjer HH, Kopitnik TA Jr: Current results of the surgical management of aneurysms of the basilar apex. **Neurosurgery** 44:697–704, 1999.
36. Schievink WI, Wijdicks EF, Piepgras DG, Chu CP, O'Fallon WM, Whisnant JP: The poor prognosis of ruptured intracranial aneurysms of the posterior circulation. **J Neurosurg** 82:791–795, 1995.
37. Varelas P, Helms A, Sinson G, Spanaki M, Hacein-Bey L: Clipping or coiling of ruptured cerebral aneurysms and shunt-dependent hydrocephalus. **Neurocrit Care** 4:223–228, 2006.

Acknowledgments

We thank Mitchell Maltenfort, Ph.D., for his expertise in statistical analysis and Summeet Vadera, B.S., and Michael Brown, B.S., for their assistance in collecting charts.

COMMENTS

The authors are to be congratulated on the excellent outcomes achieved in their 10 year experience with coiling of posterior circulation aneurysms. This retrospective, single-center series shows that the endovascular treatment can be performed at relatively low risk and that, in the short term, rerupture or new rupture is uncommon and certainly far less than the natural history for ruptured aneurysms. This series included approximately 60% ruptured cases; the percentage of patients with Glasgow Outcome Scale scores of I and II was almost 80%. The coiling was not perfect, with 20% having residual neck remnants and about 20% with some recanalization on follow-up angiography. However, for the treatment philosophy of stabilizing the dome, protecting from recurrent hemorrhage, and minimizing risk, they have achieved success. The authors also describe the improvement in aneurysm occlusion rates with the patients later in the series (13%

recanalization in patients treated between 2003 and 2005). We look forward to a follow-up study on this series of patients to see if these early benefits will be sustained in the mid- and long term.

Gary Duckwiler
Los Angeles, California

This experienced endovascular group reports a large of series of posterior circulation aneurysms treated with coil embolization. I have two concerns regarding this work. The first is the inclusion of patients with both ruptured and unruptured aneurysms. Certainly, the recurrence rate would be skewed in these comparative populations. One would expect a higher recurrence rate in patients experiencing previous subarachnoid hemorrhage. Secondly, the authors relate their 10-year experience and include cases in which they deployed the Neuroform stent (Boston Scientific/Target, Fremont, CA). The Neuroform stent was not available during the entire course of this study. In all likelihood, the use of the Neuroform stent has expanded the treatment options for patients with posterior circulation aneurysms. Nonetheless, the authors have reported a large series of patients in whom they performed rigorous clinical and statistical assessments. Of particular note is the relatively high rate of aneurysm recurrence and the low rates of vasospasm and aneurysmal rerupture. Despite the relatively high recurrence rate, the low incidence of rerupture suggests that endovascular therapy may be the treatment of choice for such lesions. The authors are to be commended for their analysis and excellent clinical results.

Felipe C. Albuquerque
Phoenix, Arizona

This is a well written report and a welcome addition to the literature from a highly respected and skilled neurovascular team. We agree with the authors that endovascular treatment is a reasonable alternative to microsurgery in select patients with posterior circulation aneurysms. This study is notable for the large number of patients and the excellent acute results with regards to aneurysm occlusion rates, which compare favorably with other published endovascular reports. Although the clinical outcomes (79% Glsgow Outcome Scale I and II) compare favorably with microsurgery outcomes for basilar apex aneurysm surgery, it should be noted that only 65% of the patients in this series had basilar apex aneurysms, which are the most challenging microsurgically, and 14% had posterior inferior cerebellar artery aneurysms, which are less challenging microsurgically. The study's limitations include the relatively short clinical and angiographic follow-up period. What remains unanswered by the report is the impact of recanalization and incomplete aneurysm treatment on long-term outcome. It is our hope that longer-term angiographic outcomes (5 and 10 yr) will shed light on the long-term efficacy of the endovascular approach to aneurysm treatment. We congratulate the authors on carefully tracking their clinical data.

Joseph G. Adel
H. Hunt Batjer
Bernard R. Bendok
Chicago, Illinois

This series represents a large population of posterior circulation aneurysms treated by endovascular means. Just as endovascular technique has evolved, the reporting of endovascular results must follow. We know that coiling is safe and effective in the short term, particularly for posterior circulation aneurysms. The burden of large endovascular aneurysm series now is to prove long-term efficacy. The mean follow-up duration in this report was 31.8 months for patients before 2004 and 13.7 months for patients undergoing treatment between 2004 and 2005. To that end, the series represents a valuable contribution for the treatment of posterior circulation aneurysms. Although this series does not add new information to the literature, it further confirms the value of endovascular treatment of posterior circulation aneurysms at a center at which there is no bias as to treatment choice.

Ramachandra P. Tummala
L. Nelson Hopkins
Buffalo, New York

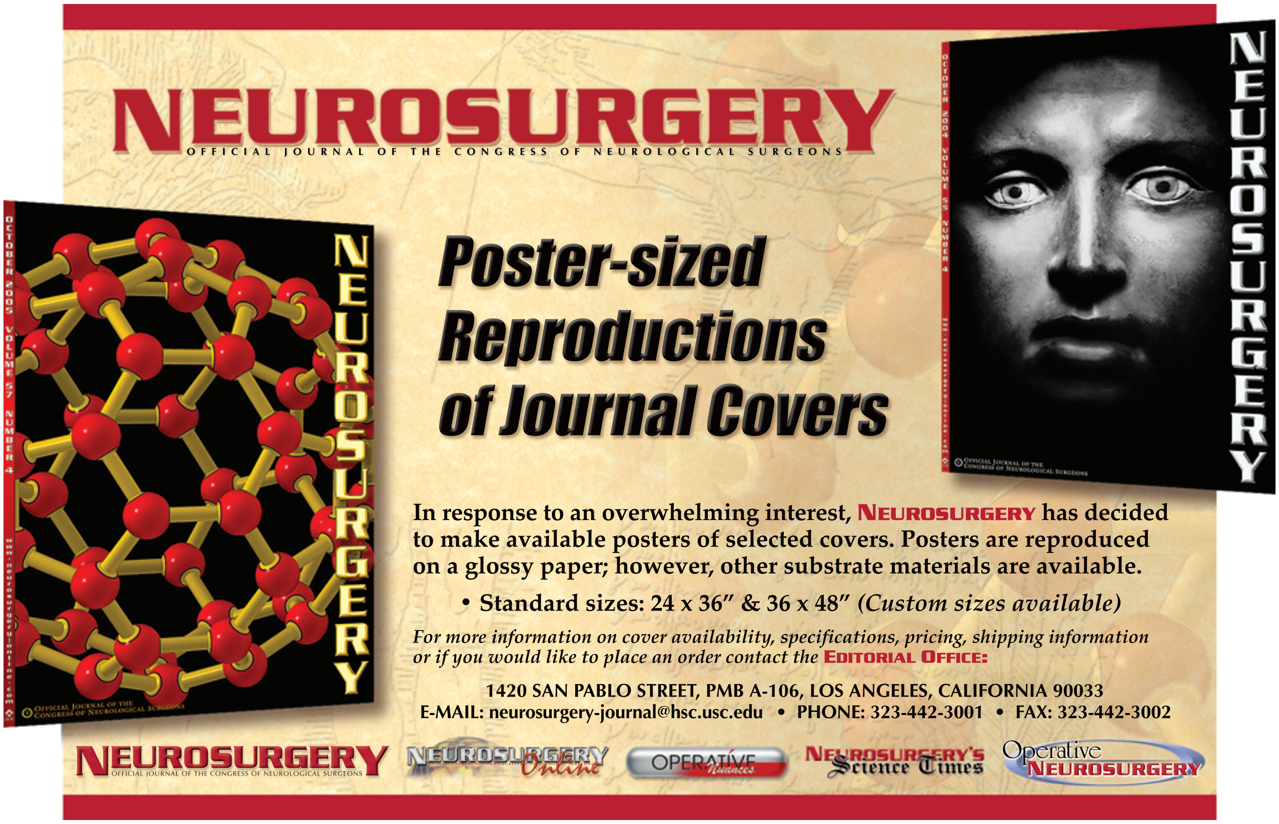

Supplement: Supplementary file 1 — Supplementary file1 (ZIP 55327 KB) [file 423_2025_3959_MOESM1_ESM.zip › literature.zip!/meta/pandey2007.pdf]
