# Supplementary material for: Management strategies and outcomes of basilar trunk aneurysms: a systematic review and meta-analysis
Source: Langenbecks Arch Surg. 2026 Jan 20;411(1):71. doi: 10.1007/s00423-025-03959-3 (PMC12852281; doi:10.1007/s00423-025-03959-3)
Supplement: Supplementary file 1 — Supplementary file1 (ZIP 55327 KB) [file 423_2025_3959_MOESM1_ESM.zip › literature.zip!/meta/peerless1994.pdf]

J Neurosurg 80:643–649, 1994

Early surgery for ruptured vertebrobasilar aneurysms

SYDNEY J. PEERLESS, M.D., F.R.C.S.(C), JUHA A. HERNESNIEMI, M.D., FREDERICK B. GUTMAN, M.D., AND CHARLES G. DRAKE, M.D., F.R.C.S.(C)

Department of Neurological Surgery, University of Miami, Miami, Florida; Department of Neurosurgery, University Hospital of Kuopio, Kuopio, Finland; and Division of Neurosurgery, University of Western Ontario, London, Ontario, Canada

The authors present a series of 1767 patients with aneurysms of the vertebrobasilar circulation, most of whom were operated on 14 days or more following their last subarachnoid hemorrhage (SAH). Since 1970, 206 patients with vertebrobasilar aneurysms have been surgically treated within 7 days after their last SAH (day of SAH = Day 0). Of patients with a good preoperative grade (Botterell Grade 1 or 2), a good or excellent outcome was obtained in 80% during the first postsurgical month, irrespective of the timing of surgery. All except one of the Grade 5 patients died, and 70% of the Grade 4 patients were significantly disabled or dead. The overall operative mortality rate was the same whether surgery took place in the 1st week after SAH or was delayed. The frequency of rupture of the aneurysm during early surgery was not higher than during late surgery. Thirteen percent of patients developed a delayed ischemic neurological deficit as a consequence of reactive arterial narrowing (vasospasm). The authors recommend early surgery for patients with a good preoperative grade, whose aneurysm does not present a particular technical difficulty because of size, configuration, or location, and occasionally in patients whose lives appear to be in jeopardy because of recurrent hemorrhage.

KEY WORDS • **vertebrobasilar aneurysm** • **subarachnoid hemorrhage** • **timing of surgery** • **management mortality**

THE timing of surgery to obliterate ruptured intracranial aneurysms remains controversial.[1–8,10–28] A recent controlled study suggested a slight benefit associated with early surgery; however, in that series posterior circulation aneurysms were excluded from analysis.[17] There is little recorded evidence of an advantage or disadvantage relating to early surgery for those uncommon aneurysms.[2,4,10,12,16,23,27,28] The International Cooperative Study on the Timing of Aneurysm Surgery[13,14] found that the number of vertebrobasilar aneurysms reported was too small to form a basis for conclusions. In a recent study, high management mortality and morbidity rates were reported, even with a policy for early surgery, although the results were excellent with no surgical mortality in good-grade cases.[10]

The relative rarity of aneurysms of the vertebrobasilar circulation prevents most neurosurgeons from gaining substantial experience with them, and therefore a policy of delayed surgical treatment is most common.[10,13,14,17] It is unlikely that the experience at London, Ontario,[4–7,18–20] with 1767 vertebrobasilar aneurysms will be duplicated or that a controlled study on the timing of surgery of vertebrobasilar aneurysms, even multicenter-based, is possible. Therefore, we believe it is valuable to report our experience with 206 patients whose vertebrobasilar aneurysms were operated on in the 1st week after subarachnoid hemorrhage (SAH).

Clinical Material and Methods

From 1959 to 1992, a total of 1767 patients underwent vertebrobasilar aneurysm surgery at our institution. Because of our referral pattern, most of these patients were operated on 14 days or more following their last SAH and after the immediate effects of the hemorrhage had passed (Table 1). Since 1970, 206 patients with ruptured vertebrobasilar aneurysms were operated

TABLE 1
Summary of 1767 patients operated on for vertebrobasilar aneurysms

Time & Type of Surgery	No. of Cases
operated on 1959–1969	64
operated on 1970–1992	1703
unruptured or remote (> 1 yr) rupture	457
early surgery (0–7 days)	206
late surgery (8–365 days)	1040

TABLE 2

*Baseline characteristics and timing of surgery in 1246 patients with ruptured vertebrobasilar aneurysms**

Variable	Timing of Surgery	
	Early Surgery (0–7 days)	Late Surgery (8–365 days)
no. of cases	206	1040
mean preop grade†	1.76	1.46
females	143 (69.4%)	664 (63.8%)
age (yrs) range	8–80	4–77
mean	48.0	46.5
multiple bleeds (%)	40.8	24.5
multiple aneurysms (%)	27.7	24.0
repeat aneurysm surgery (%)	3.4	7.5
arteriovenous malformation (%)	1.5	3.0
large & giant aneurysms (%)	41.8	38.5
aneurysm location		
basilar bifurcation	113 (54.9%)	555 (53.4%)
basilar SCA	29 (14.1%)	155 (14.9%)
basilar trunk	9 (4.4%)	76 (7.0%)
vertebrobasilar junction	14 (6.8%)	65 (6.2%)
vertebral artery	31 (15.1%)	141 (13.6%)
posterior cerebral artery	10 (4.9%)	48 (4.6%)

* Day 0 = day of subarachnoid hemorrhage. SCA = superior cerebellar artery.

† Classification according to Botterell, *et al.*[2]

on within 7 days following their last SAH (the day of SAH = Day 0); 96 of these cases were the subject of an earlier report.[20]

The 457 patients with unruptured aneurysms or aneurysms that had ruptured more than 1 year before surgery were excluded from the series. We considered it proper, however, to include three patients who underwent acute endovascular surgery (one with an excellent outcome and two who died), as acute open surgery was considered in these cases. The baseline characteristics of the remaining 1246 patients are shown in Table 2, in which the 206 early-surgery patients are compared with the 1040 patients operated on more than 7 days following their last SAH during the same time period. In the late-surgery group, one-third of the patients were operated on in the 2nd week after their last SAH and one-third in the 3rd week.

The aneurysms arose at the usual sites from vertebrobasilar or posterior cerebral arteries, with the basilar bifurcation being the most common. The distribution of vertebrobasilar aneurysms in the early-surgery group was very similar overall to our larger series (Tables 2 and 3).

Aneurysm size was classified in the same manner as in previous reports: small (< 12 mm in diameter), large (13–24 mm), and giant (> 25 mm).[4–7,18–20] Nearly two-thirds of the additional (multiple) aneurysms were treated in the same, earlier, or subsequent operations. Analysis of the computerized tomography (CT) findings has been reported earlier, and similar patterns were found in subsequent patients.[19] The preoperative classification of Botterell, *et al.*,[2] was used to grade the patients' surgical risk. Patient recovery was categorized as "excellent" only if they were neurologically normal

TABLE 3

*Outcome related to aneurysm site in 206 patients with early operation**

Site of Aneurysm	Excellent	Good	Poor	Dead	Totals
basilar bif	76	17	11	9 (8.0%)	113
basilar SCA	17	6	3	3 (10.3%)	29
basilar trunk	3	2	0	4 (44.4%)	9
VB junction	8	4	1	1 (7.1%)	14
vertebral artery	19	7	3	2 (6.5%)	31
PCA	6	3	0	1 (10.0%)	10
totals	129	39	18	20 (9.7%)	206

* Bif = bifurcation; SCA = superior cerebellar artery; VB = vertebrobasilar; PCA = posterior cerebral artery. For definition of outcome categories see text.

TABLE 4

*Outcome as related to preoperative grade in 206 patients with early operation**

Grade	Excellent	Good	Poor	Dead	Totals
1	85	12	6	4 (3.7%)	107
2	34	17	6	5 (8.1%)	62
3	9	7	2	4 (18.2%)	22
4	0	3	4	3 (30.0%)	10
5	1	0	0	4 (80.0%)	5
totals	129	39	18	20 (9.7%)	206

* Preoperative grade according to the classification of Botterell, *et al.*[2] For definition of outcome categories see text.

on repeat examination (Table 3). Patients with detectable neurological abnormalities who returned to work and to an unrestricted lifestyle were classified as "good." Patients with detectable neurological dysfunction who were unable to work or required some level of assistance in their daily living were categorized as "poor."

Results

Pre- or Intraoperative Factors

The overall results are related to grade at operation in Table 4. All except one (treated endovascularly) of the Grade 5 patients died, and 70% of the Grade 4 patients were ultimately significantly disabled or dead. A good or excellent outcome was obtained in 82% of the Grade 1 and 2 patients, irrespective of the timing of surgery. Curiously, the worst results occurred in patients who underwent surgery on Day 2. The operative mortality rate was the same for all grades of patients whether they underwent surgery in the 1st week or the surgery was delayed.

Contrary to our earlier experience, patients with large aneurysms had a similar mortality rate to patients with giant sacs (Table 5). Slightly better results were obtained in younger patients (< 50 years). None of the eight patients over 70 years of age died, but three had

TABLE 5

*Outcome as related to aneurysm size in 206 patients with early operation**

Aneurysm Size	Excellent	Good	Poor	Dead	Totals
small (< 12 mm)	79	21	12	8 (6.7%)	120
large (12–25 mm)	39	13	3	9 (14.1%)	64
giant (> 25 mm)	11	5	3	3 (13.6%)	22
totals	129	39	18	20 (9.7%)	206

* For definition of outcome categories see text.

TABLE 6

*Outcome as related to age in 206 patients with early operation**

Age (yrs)	Excellent	Good	Poor	Dead	Totals
0–9	1	0	0	0	1
10–19	2	0	0	0	2
20–29	9	2	0	1 (8.3%)	12
30–39	29	6	1	3 (7.7%)	39
40–49	42	8	5	3 (5.2%)	58
50–59	27	12	2	7 (14.6%)	48
60–69	16	9	7	6 (15.8%)	38
≥ 70	3	2	3	0	8
totals	129	39	18	20 (9.7%)	206

* For definition of outcome categories see text.

TABLE 8

*Operative and intraoperative factors and timing of surgery in 1246 patients with ruptured vertebrobasilar aneurysms**

Variable	Timing of Surgery: Early Surgery (0–7 days)	Timing of Surgery: Late Surgery (8–365 days)
no. of cases	206	1040
temporary clipping	40.8%	19.4%
use of hypotension (< 70 mm Hg)	32.5%	60.9%
intraop aneurysm rupture	13.6%	13.2%
inadvertent major vessel occlusion	1.5%	4.3%
perforator injury	5.4%	9.8%
cranial nerve injury	0.5%	1.8%
reoperation for postop hematoma	4.4%	2.9%
reclipping	1.9%	4.4%
shunt operation	8.7%	10.8%
symptomatic spasm	15.0%	6.9%
spasm contributing to death	3.4%	1.1%

* Day 0 = the day of subarachnoid hemorrhage.

a poor result (Table 6). The number of patients with multiple hemorrhages was high in the early-surgery group (41%); more than one bleed was one of the most compelling reasons to repair the aneurysm early. Nevertheless, 29% of these individuals with recent hemorrhage had a poor outcome as compared to 12% of patients with a single bleed. Communicating hydrocephalus, requiring cerebrospinal fluid diversion, was seen as frequently in the early- as in the late-surgery group. Surprisingly, the frequency of intraoperative rupture of the aneurysm was not different in the early- and late-surgery groups; however, as one might expect, the outcome for patients who suffered inadvertent intraoperative aneurysm rupture was poor (29%) as compared to those whose aneurysm did not rupture (17%).

Postoperative follow-up angiography was performed in all patients. Total obliteration was seen in 87.4% of the aneurysms; in 5.8% a small remnant of neck was seen but considered unimportant. In the remaining 14 patients (6.8%), some part of the aneurysmal body or fundus was visualized (Table 7). Reoperation and repositioning of the clip was carried out in four patients. Proximal vessel occlusion was performed when it proved to be impossible to directly clip the neck of the aneurysm in 19 patients (four patients with tourniquet).

Delayed ischemic neurological deficit as a consequence of documented vasospasm or reactive arterial narrowing was recognized in 13% of the patients. The onset of these ischemic symptoms ranged between 3 and 14 days following the last SAH.

A comparison of intra- and postoperative baseline characteristics in the 1246 patients with ruptured vertebrobasilar artery aneurysms can be seen in Table 8. The results of treatment in the early-surgery group can be seen in relation to operative method (Table 9) and time of operation within the 1st week post-SAH (Table 10).

Analysis of Aneurysms by Site

Basilar Bifurcation Aneurysms. The largest single group of aneurysms was located at the basilar bifurcation in 113 patients (Tables 3 and 11). Recurrent hemorrhage occurred in 41 of the patients. All aneurysms were saccular, 38 were large (one poor result and five deaths) and eight were giant (one poor result). Thirty-

TABLE 7

Operative method and completeness of aneurysm treatment in 206 patients with early operation

Operative Method	Total Obliteration	Residual Neck	Residual Fundus	No Obliteration	Totals
clip	162 (91.5%)	12 (6.8%)	3 (1.7%)	0	177 (85.9%)
silk ligature	1 (50.0%)	0	1 (50.0%)	0	2 (1.0%)
Hunterian ligation	13 (68.4%)	0	5 (26.4%)	1 (5.3%)	19 (9.2%)
trapping	4 (100.0%)	0	0	0	4 (1.9%)
endovascular	0	0	3 (100.0%)	0	3 (1.5%)
hematoma removed	0	0	0	1 (100.0%)	1 (0.5%)
totals	180 (87.4%)	12 (5.8%)	12 (5.8%)	2 (1.0%)	206 (100.0%)

six patients with basilar bifurcation aneurysms had additional aneurysms, seven of these were multiple vertebrobasilar aneurysms. Sixty-six patients were classified preoperatively as Grade 1, with five poor outcomes and two deaths; 31 were Grade 2, with three poor outcomes and three deaths; 11 were Grade 3 with two poor results and two deaths; and three were Grade 4 with one poor result and one death. One of two Grade 5 patients made a good recovery, the other died.

Seventeen patients underwent surgery on the day of hemorrhage or the following day, 36 on Days 2 or 3, 42 on Days 4 to 6, and 19 on Day 7. Of all intraoperative deaths, one-half occurred in the patients who underwent surgery on Days 2 to 3. In 15 patients with multiple aneurysms, a frontotemporal approach was used to clip aneurysms on the carotid circulation. Two patients received endovascular treatment; the remaining 95 were operated on via the subtemporal approach. A temporal lobe hematoma was removed from one Grade 5 patient. Division of the tentorium was necessary to gain exposure of a low-lying aneurysmal neck in 20 patients. Temporary clipping of the basilar artery was used in one-half of the cases. The neck of the aneurysm was directly clipped in 106 patients (Table 11). Direct clipping of the neck of the aneurysm was not possible in four patients because of size, rigid atherosclerotic neck, or important major vessels thought to be in jeopardy from the clip. These patients were treated with upper basilar artery occlusion (three with the tourniquet).

Complications due to technical factors were encountered in 28 patients (two major vessel occlusions, seven incomplete clipping, 12 intraoperative ruptures, and 10 perforator injuries) and accounted for six poor results and five deaths. Delayed ischemic deficit secondary to vasospasm occurred in 17 patients. In three patients, the primary cause of death was the impact of the initial or subsequent hemorrhage. The recovery of several patients was hampered by both technical complications and vasospasm. Five patients died due to vasospasm and surgical complications. Tragically, two deaths were related to postoperative clots. One patient died on Day 3 from rupture of a previously intact middle cerebral artery aneurysm.

Basilar Superior Cerebellar Artery Aneurysms. In 29 patients with aneurysms at the basilar superior cerebellar artery (Table 3), there were 10 large aneurysms (with one poor outcome and two deaths) and five giant aneurysms (one poor outcome and one death). In three Grade 3 patients there was one death, and one Grade 4 patient had a poor outcome. The pterional approach was used in four cases, subtemporal craniotomy in five, and tic-craniectomy in 20. Two cases had basilar artery occlusion, and one distal aneurysm was trapped with an excellent result. Temporary clipping of the basilar artery was used in one-third of the patients. Six patients had intraoperative aneurysm rupture. Four aneurysms were incompletely obliterated. There were no deaths related to vasospasm in this group. One patient who was recovering after evacuation of a small postoperative subdural hematoma died from the rupture of the residual one-third of the giant sac proximal to the clip. The other patient with postoperative subdural clot remained in poor condition. In two cases, death was related to intraoperative complications (aneurysm rupture and major vessel occlusion).

Basilar Trunk Aneurysms. Of nine patients with aneurysms of the basilar trunk (Table 3), two arose at the origin of the anterior inferior cerebellar artery and the remaining seven were related to small branches of the basilar artery. Both patients with anterior inferior cerebellar artery aneurysms made excellent recoveries. In four patients with giant aneurysms, two had good outcomes and two died. Two Grade 5 patients were deeply unconscious following their first hemorrhage and both died. Of two Grade 3 patients, one died and one had a good outcome. Six aneurysms were approached subtemporally (two with additional transmastoid exposure), two suboccipitally, and one with an endovascular technique alone. Four patients had basilar artery occlusion (one with a tourniquet and three using a clip). The aneurysm was totally occluded except in those treated with a tourniquet and an endovascular technique.

Vertebrobasilar Junction Aneurysms. In 11 of 14 patients with aneurysms arising at the vertebrobasilar junction, a fenestration of the basilar artery associated with the aneurysm was noted. One patient was classified preoperatively as Grade 3 and three were Grade 4. The necks of all aneurysms except a single giant one, in which vertebral ligation with a Selverstone clamp obliterated the main part of the fundus, were ligated with a clip. Ten aneurysms were completely obliterated and three almost completely. The one patient with a giant aneurysm had an excellent outcome. The only death occurred in a patient who sustained four hemorrhages and was classified as Grade 4 at the time of operation. This patient developed vasospasm 2 days after the last hemorrhage and died of widespread cerebral infarction.

Vertebral Aneurysms. Of 31 patients with vertebral aneurysms (Table 3), 37 were saccular and four were dissecting aneurysms. Three aneurysms were large, and two were giant. This group included the greatest number of patients with multiple hemorrhages (65%). Six patients were treated with proximal vertebral ligation, including all of the dissecting aneurysms. The necks of all other aneurysms were clipped. All aneurysms except two were totally occluded: one had a small remaining neck and one giant aneurysm treated by proximal vertebral ligation filled partially from the contralateral vertebral artery. Fourteen patients had postoperative low cranial nerve dysfunction: in six patients these disabling palsies were persistent. One death occurred due to repeat hemorrhage and one due in part to vasospasm (this patient also had a subdural hematoma). Seven patients developed hydrocephalus and required a shunt, accounting for one-third of the shunts in the whole early-surgery group. Three large and two giant aneurysms were operated on without mortality, but one patient with a giant aneurysm had a poor outcome with severe hemiparesis. Of three patients pre-

TABLE 9

*Operative method and outcome in 206 patients with early operation of vertebrobasilar aneurysms**

Operative Method	Excellent	Good	Poor	Dead	Totals
clip	116 (65.6%)	33 (18.6%)	17 (9.6%)	11 (6.2%)	177 (85.9%)
silk ligature	1 (50.0%)	0	0	1 (50.0%)	2 (1.0%)
Hunterian ligation	8 (42.1%)	6 (31.6%)	1 (5.3%)	4 (21.1%)	19 (9.2%)
trapping	3 (75.0%)	0	0	1 (25.0%)	4 (1.9%)
endovascular	1 (33.3%)	0	0	2 (66.7%)	3 (1.5%)
hematoma removed	0	0	0	1 (100.0%)	1 (0.5%)
totals	129	39	18	20 (9.7%)	206

* For definition of outcome categories see text.

TABLE 10

*Outcome as related to timing of operation in 206 patients with early operation on vertebrobasilar aneurysms**

Timing of Surgery	Excellent	Good	Poor	Dead	Totals
Day 0–1	20 (54.1%)	6 (16.2%)	5 (13.5%)	6 (16.2%)	37 (18.0%)
Day 2–3	34 (57.6%)	10 (16.9%)	8 (13.6%)	7 (11.9%)	59 (28.6%)
Day 4–6	55 (70.5%)	17 (21.8%)	3 (3.8%)	3 (3.8%)	78 (37.9%)
Day 7	20 (62.5%)	6 (18.8%)	2 (6.3%)	4 (12.5%)	32 (15.5%)
totals	129	39	18	20 (9.7%)	206

* For definition of outcome categories see text.

TABLE 11

*Operative method and outcome in 113 patients with early operation of basilar bifurcation aneurysms**

Operative Method	Excellent	Good	Poor	Dead	Totals
clip	73 (68.9%)	16 (15.1%)	11 (10.4%)	6 (5.7%)	106 (93.8%)
Hunterian ligation	2 (50.0%)	1 (25.0%)	0	1 (25.0%)	4 (3.5%)
endovascular	1 (50.0%)	0	0	1 (50.0%)	2 (1.8%)
hematoma removed	0	0	0	1 (100.0%)	1 (0.9%)
totals	76 (67.3%)	17 (15.0%)	11 (9.7%)	9 (8.0%)	113 (100.0%)

* For definition of outcome categories see text.

operatively in Grade 4, one died and one remained severely disabled. The remaining three poor results were seen in Grade 2 patients: one with the giant aneurysm with proximal vertebral artery ligation already mentioned, one 70-year-old woman with a huge clot from hemorrhage into a Dandy-Walker cyst requiring permanent tracheostomy who remained disabled with ataxia and a feeding jejunostomy, and a 68-year-old woman with perioperative aneurysm rupture and postoperative vasospasm; she died from a pulmonary embolus $2\frac{1}{2}$ months after operation and 2 days after discharge.

Posterior Cerebral Artery Aneurysms. Posterior cerebral artery aneurysms were found in 10 patients (Table 3). Six had clip occlusion of the aneurysm, one had proximal posterior cerebral artery occlusion, and one aneurysm was trapped. One patient died; the others had excellent or good results. The subtemporal approach was used in eight patients (three by small tic-craniectomies). The frontotemporal approach was used to expose an aneurysm located high above the posterior clinoid aneurysms of the P_1 segment and one located at the P_1–P_2 segments, associated with a middle cerebral artery aneurysm. The only death occurred in a patient with a large aneurysm and who was classified preoperatively as Grade 5.

Discussion

Early surgery for posterior circulation aneurysms was attempted by the senior author in some of the earliest cases. Two of the first four patients died, one from cardiac arrest following poorly controlled hypotension and hypothermia.[4–6] Since that time, and after operating on more than 1750 patients with aneurysms arising from the vertebral and basilar arteries in our unit, the pre- and postoperative care, the techniques of neuroradiology, anesthesia, and surgery, and the recognition and management of complications of SAH have become well established and quite routine.[4–7,18–20,24] Overall, more than 80% of these patients have achieved excellent or good results, and the surgical mortality for the whole group is 6.5%. Even so, we continue to be concerned by a combined morbidity and mortality rate

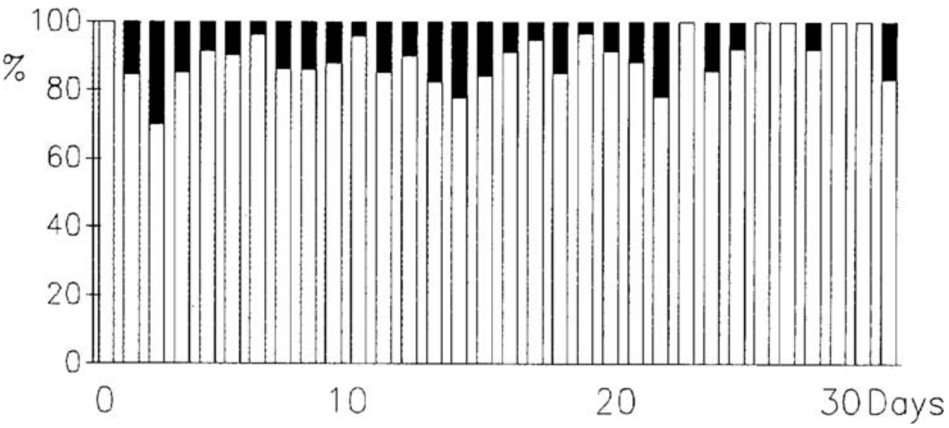

FIG. 1. Bar graph showing the outcome of surgery in the first 32 days after subarachnoid hemorrhage in 868 good-grade (Botterell, *et al.*,[2] Grades 1 and 2) patients with vertebrobasilar aneurysms. *Closed bars* = poor outcome, including death; *open bars* = excellent or good outcome.

of 15% and are continually haunted by the deterioration and often death of patients because of recurrent hemorrhage occurring before the aneurysm can be secured. Second or third hemorrhage almost always resulted in a worse grade and a worse outcome. The posterior fossa aneurysms seem to rebleed easily and more of these were fatal (48% to 83%): one point in favor of acute surgery.[10,26]

As a result of the rarity of these aneurysms, combined with limited surgical experience and technical difficulties in treating them, there has been a general trend, supported by our previous publications, to delay surgery for those aneurysms arising from the posterior circulation.[1,7,11,12,16,23,28] We have compared the results of early operation in patients with aneurysms arising from the anterior circulation to those in patients with aneurysms arising from the posterior circulation.[20] The results are equally good in patients graded 1 or 2 at the time of surgery and equally poor in those graded 3 or 4 according to the Botterell scale.[2] Thus, in our hands, the condition of the patient going into the operating room would appear to be the most powerful determinant of outcome.

It has been suggested that vasospasm is less common following rupture of aneurysms of the posterior circulation. It has also been suggested that early operation may increase the incidence of vasospasm by some unknown effect of the surgical manipulation or anesthetic carried out at a time when the vessel's reactivity is most vulnerable. Conversely, it has been hoped that the incidence of vasospasm might be reduced by the evacuation of clot and bloody cerebrospinal fluid at the time of surgical intervention. Our experience does not support any of these notions. The frequency of vasospasm was significantly lower in the late-surgery group. In most of these cases, however, surgery was performed outside the usual time limits of occurrence of clinical spasm (Fig. 1). The most important predictor of vasospasm has been the CT documentation of subarachnoid clot.[9,20]

Isolation of the thin sac of an aneurysm by surgical obliteration of the neck to prevent rebleeding is the ultimate goal in the operative treatment of intracranial aneurysms. If the sac is of large or giant size, the technical complexity of the surgical repair is markedly increased. Neck clipping is always difficult and may be impossible. The proximal or Hunterian ligation of the parent artery in these circumstances is an effective and usually safe alternative method of treatment in those patients coming electively to surgery.[4–7,19,20,24] Proximal vessel ligation was attempted in 18 patients in the acute stage, with five deaths. These disappointing results in a few patients with complex lesions have made us cautious about attempting proximal vessel ligation in the week immediately following acute SAH.

In our hands, early surgery for vertebrobasilar aneurysms yielded results similar to early surgery for aneurysms of the anterior circulation, and they are not dissimilar to the results achieved in delayed operation of vertebrobasilar aneurysms in our unit. It is clear that early surgery, with the aneurysm appropriately clipped, will dramatically reduce the risk of rebleeding. Vasospasm is no more common, no less amenable to therapy, and no more severe in patients who have been operated on early than in those receiving delayed surgical treatment. Volume expansion and elevation of blood pressure are safer after the aneurysm has been surgically obliterated. Although early surgery more frequently presents a brain that is more swollen and tight with a confining exposure, the use of lumbar spinal fluid drainage, controlled ventilation, and osmotic diuretics has made exposure possible. Dissection of the aneurysm itself with removal of surrounding fresh clot, identification of the neck, and separation of perforators, although somewhat more tedious in the acute case, is not significantly more difficult or more hazardous. The early or late timing of surgery did not cause intraoperative aneurysm rupture to be more frequent or more difficult to control.

We recommend early surgery in those patients who

are given a good preoperative grade (Botterell Grade 1 to 2 or a Hunt and Hess Grade I to III[2,11]), whose aneurysm does not present a particular technical difficulty because of size, configuration, or location, and occasionally in patients whose lives appear to be in jeopardy because of recurrent hemorrhage. However, review of this series makes us hesitant to recommend early surgery for aneurysms arising from the basilar trunk.

The most important factor in the timing of surgery in a patient with a good preoperative grade is the availability of an experienced, alert surgical team. It is well shown by this series that results improve with experience. Patients with basilar bifurcation aneurysms, once held to be the most difficult aneurysms to treat, had results comparable to those achieved in patients with aneurysms at other locations, whether in the anterior or posterior circulation. Centralizing the care of these challenging lesions in a few experienced hands with adequate surgical experience may be a tool for improved patient outcome. In spite of improvements in surgical techniques, we are far away from ideal results. Because there are now means to detect aneurysms before they rupture, there is progress toward the best timing of aneurysm surgery: operation before rupture. This will improve the management results more than any technical innovation.

References

1. Batjer HH, Samson DS: Causes of morbidity and mortality from surgery of aneurysms of the distal basilar artery. **Neurosurgery 25:**904–916, 1989
2. Botterell EH, Lougheed WM, Scott JW, et al: Hypothermia, and interruption of carotid, or carotid and vertebral circulation, in the surgical management of intracranial aneurysms. **J Neurosurg 13:**1–42, 1956
3. Dandy WE: **Intracranial Arterial Aneurysms.** Ithaca, NY: Comstock, 1944
4. Drake CG: Bleeding aneurysms of the basilar artery. Direct surgical management in four cases. **J Neurosurg 18:** 230–238, 1961
5. Drake CG: Further experience with surgical treatment of aneurysms of the basilar artery. **J Neurosurg 29:**372–392, 1968
6. Drake CG: Surgical treatment of ruptured aneurysms of the basilar artery. Experience with 14 cases. **J Neurosurg 23:** 457–473, 1965
7. Drake CG: The treatment of aneurysms of the posterior circulation. **Clin Neurosurg 26:**96–144, 1979
8. Falconer MA: Surgical treatment of spontaneous intracranial haemorrhage. **Br Med J 1:**790–792, 1958
9. Fisher CM, Kistler JP, Davis JM: Relation of cerebral vasospasm to subarachnoid hemorrhage visualized by computerized tomography scanning. **Neurosurgery 6:**1–9, 1980
10. Hernesniemi J, Vapalahti M, Niskanen M, et al: Management outcome for vertebrobasilar artery aneurysms by early surgery. **Neurosurgery 31:**857–862, 1992
11. Hunt WE, Hess RM: Surgical risk as related to time of intervention in the repair of intracranial aneurysms. **J Neurosurg 28:**14–20, 1968
12. Jamieson KG: Aneurysms of the vertebrobasilar system. Surgical intervention in 19 cases. **J Neurosurg 21:**781–797, 1964
13. Kassell NF, Torner JC, Haley EC Jr, et al: The International Cooperative Study of the Timing of Aneurysm Surgery. Part 1: Overall management results. **J Neurosurg 73:**81–36, 1990
14. Kassell NF, Torner JC, Jane JA, et al: The International Cooperative Study on the Timing of Aneurysm Surgery. Part 2: Surgical results. **J Neurosurg 73:**37–47, 1990
15. Logue V: Posterior fossa aneurysms. **Clin Neurosurg 11:** 183–207, 1964
16. MacFarlane MR, McAllister VL, Whitby DJ, et al: Posterior circulation aneurysms. Results of direct operations. **Surg Neurol 20:**399–413, 1983
17. Öhman J, Heiskanen O: Timing of operation for ruptured supratentorial aneurysms: a prospective randomized study. **J Neurosurg 70:**55–60, 1989
18. Peerless SJ: Pre- and post-operative management of cerebral aneurysms. **Clin Neurosurg 26:**209–231, 1979
19. Peerless SJ, Drake CG: Posterior circulation aneurysms, in Wilkins RH, Rengachary SS (eds): **Neurosurgery.** New York: McGraw-Hill, 1985, pp 1422–1437
20. Peerless SJ, Nemoto S, Drake CG: Acute surgery for ruptured posterior circulation aneurysms. **Adv Tech Stand Neurosurg 15:**115–129, 1987
21. Poppen JL: Vascular surgery of the posterior fossa. **Clin Neurosurg 6:**198–209, 1959
22. Schwartz HG: Arterial aneurysm of the posterior fossa. **J Neurosurg 5:**312–316, 1949
23. Sugita K, Kobayashi S, Shintani A, et al: Microneurosurgery for aneurysms of the basilar artery. **J Neurosurg 51:** 615–620, 1979
24. Steinberg GK, Drake CG, Peerless SJ: Deliberate basilar or vertebral artery occlusion in the treatment of intracranial aneurysms. Immediate results and long-term outcome in 201 patients. **J Neurosurg 79:**161–173, 1993
25. Tönnis W: Zur Behandlung Intrakraniellar Aneurysmen. **Langenbecks Arch Klin Chir 189:**474–476, 1937
26. Troupp H: The natural history of aneurysms of the basilar bifurcation. **Acta Neurol Scand 47:**350–356, 1971
27. Yaşargil MG: **Microneurosurgery. II. Clinical Considerations, Surgery of the Intracranial Aneurysms and Results.** Stuttgart: Georg Thieme Verlag, 1986, pp 232–295
28. Yaşargil MG, Antic J, Lagica R, et al: Microneurosurgical pterional approach to aneurysms of the basilar bifurcation. **Surg Neurol 6:**83–91, 1976

Manuscript received June 11, 1993.

Accepted in final form August 9, 1993.

Address for Dr. Hernesniemi: Department of Neurosurgery, University Hospital of Kuopio, Kuopio, Finland.

Address for Dr. Drake: Division of Neurosurgery, University of Western Ontario, London, Ontario, Canada.

Address reprint requests to: Sydney J. Peerless, M.D., F.R.C.S.(C), Department of Neurological Surgery, 1501 N.W. 9th Avenue, Miami, Florida 33136.
