# Supplementary material for: Management strategies and outcomes of basilar trunk aneurysms: a systematic review and meta-analysis
Source: Langenbecks Arch Surg. 2026 Jan 20;411(1):71. doi: 10.1007/s00423-025-03959-3 (PMC12852281; doi:10.1007/s00423-025-03959-3)
Supplement: Supplementary file 1 — Supplementary file1 (ZIP 55327 KB) [file 423_2025_3959_MOESM1_ESM.zip › literature.zip!/meta/qu2009.pdf]

Clinical Outcomes of Basilar Artery Aneurysms

S. QU*, X. LV**, Z. WU**

* The affiliated Hospital to Changchun University of Chinese Medicine; Changchun, China
** Beijing Neurosurgical Institute, Beijing Tiantan Hospital, Capital Medical University; Beijing, China

Key words: basilar artery, intracranial aneurysm, endovascular embolization

SUMMARY – *To evaluate the effects of endovascular treatments, we retrospectively analyzed a consecutive series of patients with basilar artery aneurysms (BAAs) treated endovascularly. During a seven year period, 43 patients (mean age 42.2 years, male/female ratio 21:22) with BAAs were treated mainly with endovascular techniques at Beijing Tiantan Hospital. Pretreatment clinical grades were determined using the Hunt-Hess scale. Outcome was evaluated using the Glasgow Outcome Scale scores (GOS) during a mean follow-up period of 21.1 months (range, 1 to 72 months). Forty-three patients had 44 BAAs, one MCA aneurysm and one P1 aneurysm. Endovascular treatment was technically feasible in 40 patients. One aneurysm thrombosed spontaneously after initial angiography. Four (9.3%) patients died periprocedurally. Immediate postprocedural angiograms in 44 BAAs showed that complete occlusion was achieved in 33 BAAs, subtotal occlusion in one and incomplete occlusion in seven. Follow-up angiographic results in 30 patients confirmed complete occlusion of 27 aneurysms, subtotal occlusion in one, and incomplete occlusion in two. Two patients with vertebrobasilar atheromatous fusiform aneurysms were treated with antiplatelet medications. Long-term outcome was good (GOS Score 4 or 5) in 39 patients (90.7%) and fatal (GOS Score 1) in four (9.3%). Favorable overall long-term outcome can be achieved in patients with BA apex aneurysms, and in 78.6% of those with BA trunk aneurysms when using endovascular techniques. Endovascular coil embolization of BAAs is an effective treatment in the long-term. Patients with vertebrobasilar atheromatous fusiform aneurysms can be treated with antiplatelet medications.*

Introduction

Aneurysms of the basilar artery (BA) are rare and have a poorer prognosis than that of typical saccular aneurysms [5,7,9,10,15,19]. One literature report documented a five year incidence of severe morbidity and mortality of 80% [7]. Anatomically, the BA is located in the depth of the valley surrounded by the bilateral petrous bones. Surgical access to this area is hampered by a dense collection of vital cranial nerves and perforating arteries to the brain stem [4,5,11-14]. Challenges include difficulty with the surgical approach, the frequent occurrence of broad-necked, fusiform or giant aneurysms, and the incorporation of the branches of the BA. As a result, the rarity and unique morphological features of BA aneurysms make it difficult to tailor the treatment strategy individually.

However, an endovascular approach to this area is comparatively easy because of the simple access to the BA [1,5,16]. The objective of this retrospective analysis was to evaluate endovascular strategies and the results of endovascular management, and review the relevant literature.

Clinical Material and Methods

The clinical data for this study were obtained from a retrospective analysis of 43 patients with 44 BAAs who underwent endovascular treatment at Beijing Tiantan Hospital, Capital Medical University between 2000 and 2007. There were 22 women (51.2%) and 21 men (48.8%), ranging from 10 to 71 years with a mean age of 42.2 years. Twenty-seven patients presented with SAH. The patients presenting without SAH had headaches (n=9), symptoms of mass effect and ischemia (n=7). The aneurysms were located on the basilar artery: basi-

lar apex (n=26); basilar trunk (n=14); and vertebrobasilar artery (n=3).

All procedures were performed or supervised by the senior author (ZW). All endovascular procedures were performed in the neurointerventional suite via the transfemoral arterial route. All endovascular surgeries were performed under general anesthesia. All the procedures were performed under full systemic heparinization to maintain the activated clotting time around twice the base value. In this series, Guglielmi detachable coil (GDC, Boston Scientific, Fremont, CA, USA/Orbit, Cordis, Cal, USA/Microvention, Microvention, Aliso Viejo, CA, USA) was mainly used to fill the aneurysm. The detachable coils were delivered under road-mapping guidance, and the aneurysm was packed as densely as possible. Forty patients underwent endovascular treatment and two patients with atheromatous fusiform aneurysm were treated with 100 mg aspirin by mouth daily. Four patients underwent endovascular parent vessel occlusions without bypass procedure. If a stent was placed, the patient was medicated with 300 mg/d aspirin and 75 mg/d clopidogrel three days before procedure and 300 mg/d aspirin after one month.

Postoperative angiography was classified as follows: complete occlusion (Co), subtotal (Su), or incomplete (In). Results of follow-up angiography were also classified in the same way, and clinical outcomes were evaluated according to Glasgow Outcome Scale scores. The preoperative neurological function was evaluated according to the Hunt-Hess (H&H) grade.

Aneurysm Location

Twenty-six aneurysms (60.5%) were located at the BA apex, three (7%) were at the upper BA trunk, four (9.3%) at the middle BA trunk and seven at the lower BA trunk or vertebrobasilar junction. The vertebal and basilar arteries were involved in three patients.

Hunt and Hess Grade

The neurological condition in patients presenting with symptoms was related to mass effect or ischemia such as TIA, stroke, cranial nerve dysfunction. Based on the Hunt and Hess grading scale, the patient population included 27 patients (62.9%) with ruptured aneurysms, 18 patients (41.9%) with Grade I aneurysms, six (14%) with Grade II aneurysms and three (7%). In the other 16 (37.1%) patients (presenting without SAH), symptoms were attributed to brainstem compression in four (9.3%), TIA or stroke in three (7%) patients. Nine patients (20.9%) presented with headache but no neurological deficits.

Table 1 Definition of Hunt and Hess unruptured aneurysm subclassifications

Hunt & Hess Grade.	*Definition*
O1	independent & able to work
O2	independent but unable to work
O3	dependent & unable to work

Results

The aneurysms were small in 25 (diameter <11 mm), large in 13 (11 to 25 mm), and giant in five (>25 mm). Thirty-five (79.5%) aneurysms were saccular, nine (20.5%) aneurysms were fusiform including three atheromatous fusiform aneurysms. Three cases were associated with multiple aneurysms. All cases are listed in Table 1. Endovascular treatment was technically feasible in 39 cases: 27 ruptured aneurysms and 12 unruptured aneurysms, including three aneurysms associated with three incidentally discovered aneurysms. Stent was used in 13 cases and a balloon assisted technique was used in three cases to prevent the coils from protruding into the parent artery. In 33 (75%) out of 44 BAAs, CO was achieved, while Su was found in one (2.3%) aneurysm, and In remained in seven (15.9%). Follow-up angiography was obtained in 30 surviving cases: there was CO in 27 aneurysms, Su in one aneurysm and In in two aneurysms. After a mean follow-up duration of 21.1 months (range, 1-72 months), outcome in 39 patients (90.7%) was reflected by a GOS score of 4 or 5, in four (9.3%) by a score of 1.

Summary of Deficits

Neurological status worsened within 30 days of treatment in seven (16.3%) out of 43 cases, resulting in death in four. Neurological status in two patients manifested as new or worsening of disequilibrium, weakness, numbness and dysarthria. Neurological deficits were attributed to ischemia in two patients and progression of brainstem compression in two. In one case of lower BA trunk fusiform aneurysm, thrombosis occurred after stent placement and caused brain stem infarction after the procedure regardless of oral administration of an antiplatelet agent.

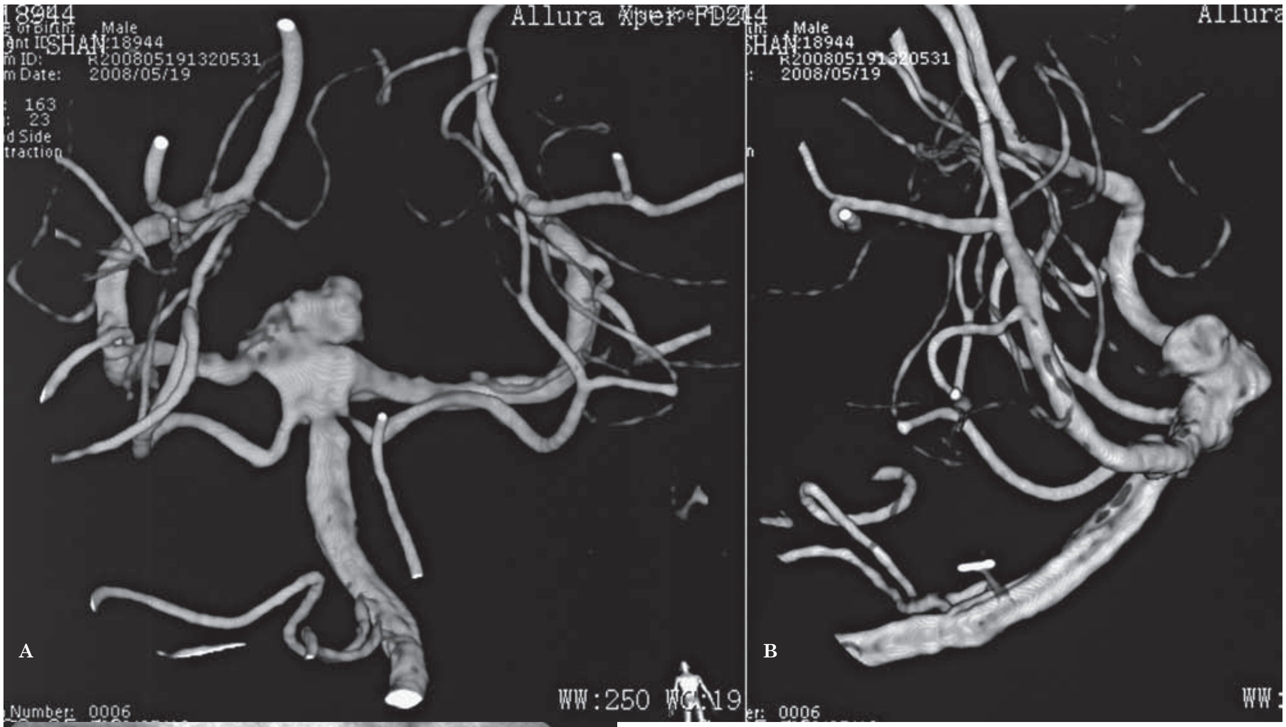

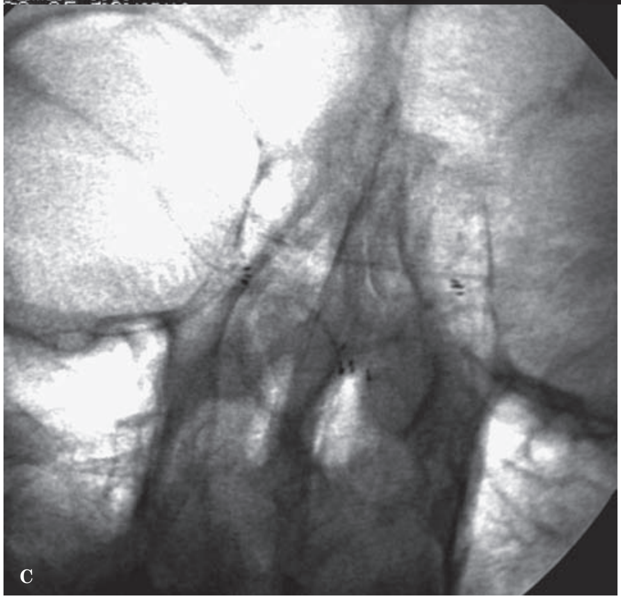

Figure 1 A 57-year-old man presented with a wide-necked basilar artery apex aneurysm and bilateral PcoAs were small. He denied of surgical clipping. After discussion of the treatment alternatives in detail, a Y-stent reconstruction technique was thought to offer the best chance for treatment of this aneurysm. A) 3-D reconstructions of the left vertebral injection, AP (left) and lateral (right), showing the aneurysm. B) Fluoroscopic image shows Y-stent-supported reconstruction of the basilar apex. C) Postprocedure angiography shows coil occlusion of the aneurysm. Six days after the treatment, the patient experienced transient internuclear ophthalmoplegia and neurological examinination was intact at 2-month follow-up.

Summary of Deaths

Recurrent SAH was confirmed to be the cause of death in three patients (occurring after endovascular treatment), whereas increasing mass effect or brainstem ischemia was the cause of death in one patient (at one month after treatment). In all of these patients there was residual aneurysm filling in two patients after treatment.

Discussion

Basilar artery aneurysms (BAAs) are considered to comprise less than 4% of intracranial aneurysms [8,9,12,15,17,18,21]. These aneurysms have a poorer prognosis than aneurysms at other locations [7,12]. One group documented a five year incidence of severe morbidity and mortality of 80% [12]. Several neurosurgical approaches have been used to access the BA as described by many

Table 2 Data of 26 patients with basilar apex aneurysms

Patient No	*Age/ Sex*	*HH Grade*	*Sizes of aneurysms(mm)*	*Treatment*	*Angio-graphic outcome*	*Follow-up*	*Outcome (GOS)*
1.	45/M	I	4×6, saccular	Remodeling(R, P1) Hyperglide(4×20)	Co	3-mo	5
2.	51/F	I	20×20, saccular	Coiling	Co	11-mo	5
3.	44/M	I	5×9, saccular	Coiling	Co	3-mo	5
4.	38/F	I	4×7, saccular	Coiling	Co	10-mo	5
5.	39/M	I	10×10, saccular	Coiling	Co	3-mo	5
6.	57/M	I	6×7, saccular	Coiling	Co	6-mo	5
7.	38/M	I	6×6, saccular	Remodeling(R,P1) Hyperglide(4×20)	Co	9-mo	5
8.	57/M	I	5×6, saccular	Y-stent technique 3×20,3×15	Sub	3-mo	5
9.	51/F	I	4×5, saccular	Stent(R,P1) 3.5×20	Co	4-mo	5
10.	43/M	I	7×7, saccular	Remodeling(R, P1) Hyperglide(4×20)	Co	54-mo	5
11.	41/F	II	4×8, saccular	Coiling	Co	53-mo	5
12.	42/M	I	5×6, saccular	Stent(L,P1) 3.0×20	Co	48-mo	5
13.	34/M	I	4×3, saccular	Coiling	Co	5-mo	5
14.	49/F	II	20×25, saccular	Stent(L,P1) 3.5×20	Co	21-mo	5
15.	10/F	O1	7×10, saccular	Stent(R,P1) 3.5×20	Co	10-mo	5
16.	62/F	O1	15×9, saccular	Stent(R,P1) 3.5×20	Co	18-mo	5
17.	53/F	O1	17×17, saccular	Coiling	Co	35-mo	5
18.	50/F	O2	20×30, saccular	Coiling	Co	33-mo	5
19.	50/M	II	7×6, saccular	Coiling	Co	15-mo	5
20.	46/F	I	30×30, saccular	Coiling	Co	72-mo	4
21.	29/M	II	15×15, saccular	Coiling	In	8-mo	5
22.	29/M	II	15×15, saccular	Coiling	In	8-mo	5
23.	71/M	O3	10×10, saccular	Coiling	In	12-mo	3
24.	26/F	O2	16×15, saccular	Coiling	Co	6-mo	5
25.	51/F	I	4×5, saccular	Stent(R,P1) 3.5×20	Co	60-mo	5
26	10/F	O1	5×5, saccular	Stent(L,P1)	Co	11-mo	5

Note – HH Grade, Hunt-Hess grade; M, male; F, female; R, right; GOS, Glasgow outcome score, mo, months; Co, complete; In, incomplete; Sub, subtotal

authors, but these approaches have been successful only in the hands of a limited number of skilled neurosurgeons [4,13,17-19,21,23,27]. On the other hand, endovascular access to the BA is not difficult in general [10-12,19,22,24,30]. Endovascular neurosurgery has offered a striking therapeutic benefit because endovascular access is not affected by complex arteries of the BA trunk such as

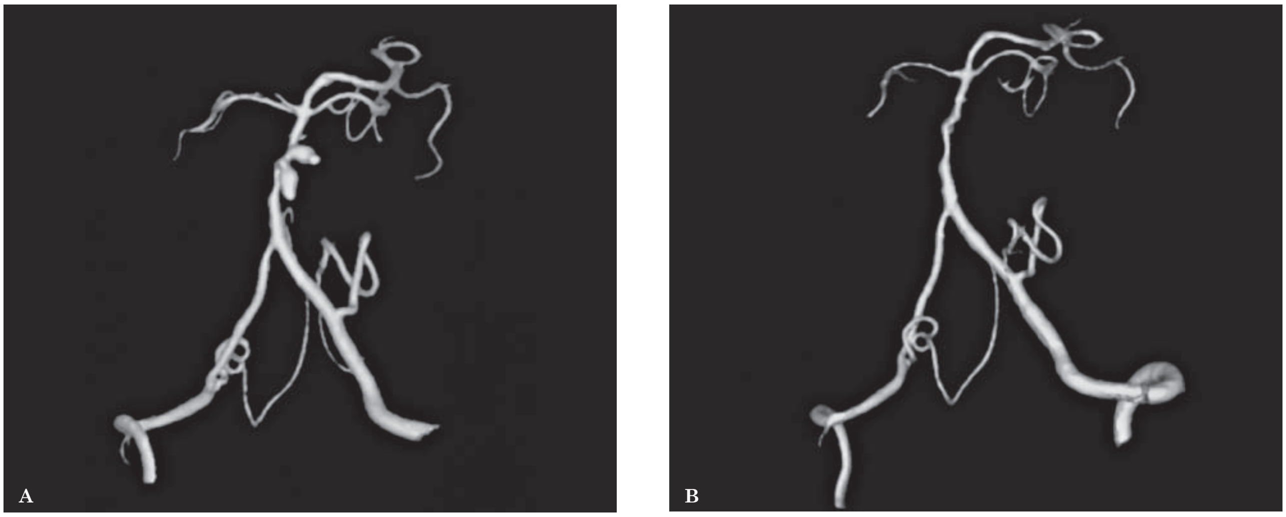

Figure 2 A 33-year-old man who presented with SAH was found to have two aneurysms at the middle BA. A) 3-D reconstruction of the left vertebral injection, AP, shows the 2 aneurysms. B) 6-month follow-up angiogram revealed that the aneurysms were no longer filled.

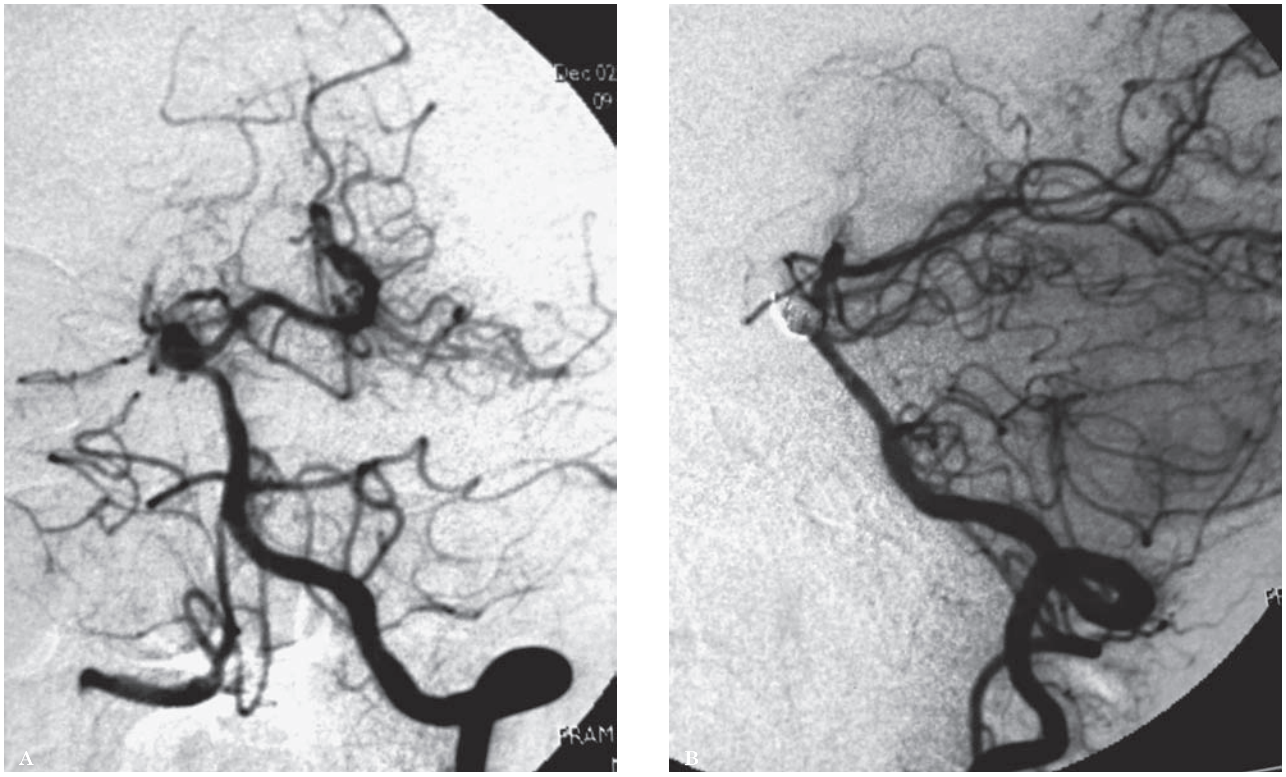

Figure 3 A 52-year-old woman who presented with SAH and was found to harbor a saccular aneurysm at the middle BA as shown on left VA angiogram (AP [A]). The aneurysm was completely obliterated by placing coils (lateral [B]). This patient died 15 hours after procedure of an SAH (GOS score of 1).

many perforating arteries and cranial nerves, which may often cause the problems in a surgical approach. Since the advent of endovascular techniques, we have selected endovascular surgery as the first choice of treatment for BAAs with anticipated surgical difficulties. There was no procedure-related mortality in this series, compared with rates ranging from 0% to

Table 3 Data of 14 patients with basilar trunk aneurysms

Patient No.	Age/ Sex	HH Grade	Sizes of aneurysms(mm)	Loca-tion	Treatment	Angiographic outcome	Follow-up	Outcome
1.	18/F	I	50×60, Saccular	Mid BA	Stent alone (Driver)	In	18days	1
2.	10/M	II	34×45, Fusiform	Lower BA	Stent alone (Driver)	In	21days	1
3.	39/F	O1	14×19, Saccular	VBJ	Stent-coil (Neuroform)	Co	4-mo	5
4.	18/M	O1	12×10, Saccular	VBJ	Coil	Co	56-mo	5
5.	51/F	O1	10×10, Saccular	Upper BA	Coil	Co	8-mo	5
6.	52/F	I	6×7, Saccular	Mid BA	Coil	Co	15-h	1
7.	22/M	O1	21×15, Fusiform	VBJ	PAO, LVA	In	4-mo	5
8.	44/F	I	9×7, Fusiform	VBJ	Stent-coil (AVE)	Co	70-mo	5
9.	46/F	O2	25×15, Fusiform	VBJ	PAO	In	72-mo	5
10.	33/M	I	3×5, Saccular 3×5, Saccular	Mid BA	Coil	Co	6-mo	5
11.	31/F	III	20×30, Fusiform	Upper BA	PAO	Co	1-mo	4
12.	50/M	III	7×8, Saccular	Lower BA	Coil	Co	5-mo	5
13.	68/F	III	7×12, Saccular	Mid BA	Stent-coil	Co	8-mo	5
14.	36/F	O3	14×10, Fusiform	Upper BA	Thrombosed spontaneously		9-mo	5

Note – Mid BA, middle basilar artery; VBJ, vertebrobasilar junction; PAO, parent artery occlusion.

Table 4 Data of three patients with atheromatous fusiform aneurysm

Patient No.	Age/ Sex	HH Grade	Sizes of aneurysms (mm)	History of hypertension (years)	Treatment	Follow-up	Outcome (GOS)
1.	54/M	O2	6, Fusiform	18	Aspirin	24-mo	4
2.	67/M	O1	8, Fusiform	13	Aspirin	36-mo	5
3.	59/M	O3	10, Fusiform	10	PAO, bi VA	1-mo	1

3.8% in other series of posterior circulation (9,26). A thromboembolic complication occurred in one of our patients despite oral administration of an antiplatelet agent. As in other reported series, thromboembolic events seem to be the most common complication, and special attention must be paid to platelet aggregation in cases treated with stent placement [1,6,25,29]. Although detachable coils have provided an alternative approach to treatment, they have their own difficulties in the treatment of giant and fusiform aneurysms of the BA [5,11,24,30,31]. Clot formation in the sac does not mean the aneurysm is completely cured, particularly with giant aneurysms or aneurysms with a wide neck in which the main blood stream impinges upon the orifice of the aneurysm and may disturb endothelialization of the aneurysm orifice [12]. Subsequent bleeding was observed in three patients, two BA trunk giant aneurysms were treated with stenting without coiling and one was coiled completely on angiogram. The rebleeding rate of coil embolization in this series was 2.3% as good as that reported in other endovascular series, ranging from 0% to 3.7% [9,12,16]. Endovascular stenting without coiling has been used successfully for the treatment of atherosclerotic lesions, pseudo-aneurysms, fusiform aneurysms, arterial dissections and thrombosis of the cerebral vasculature [15,16,19,26]. Fiorella et Al

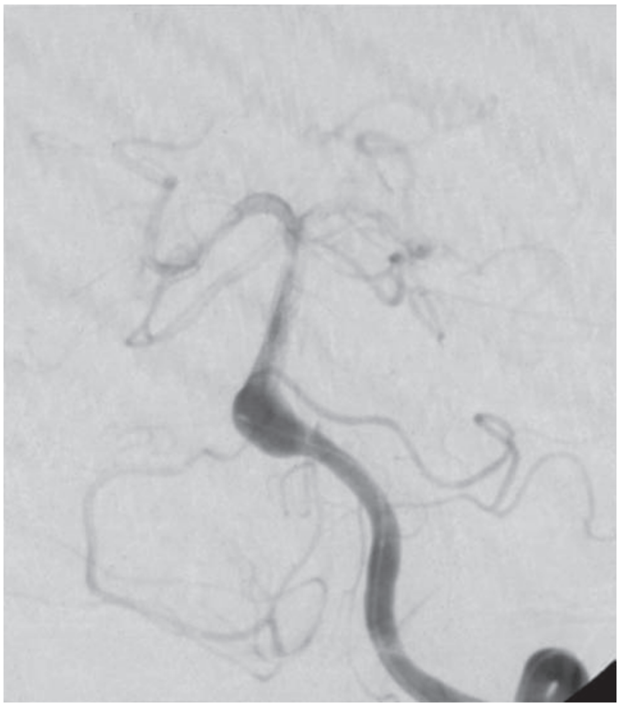

A

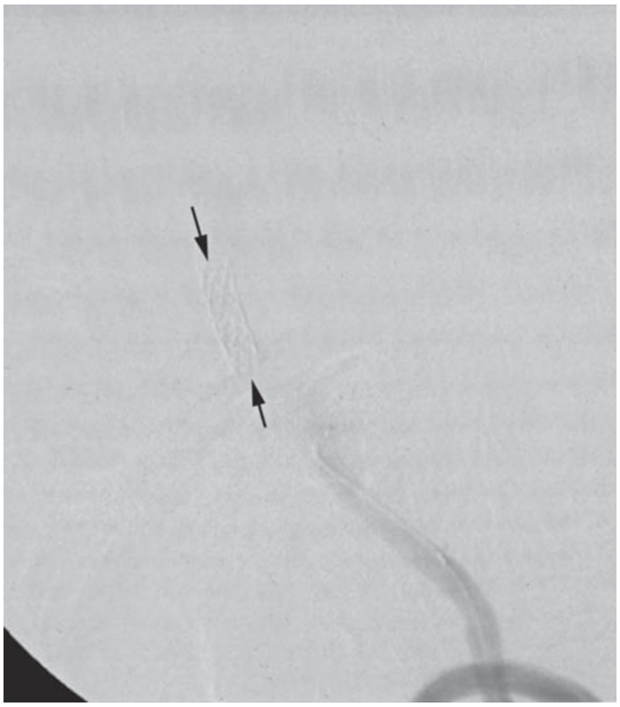

B

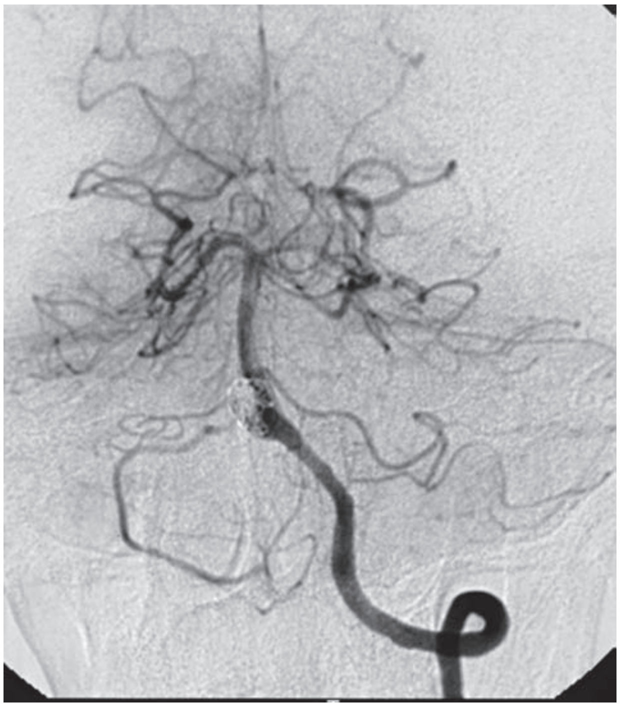

C

Figure 4 A 44-year-old presented with SAH and cerebral angiography revealed a fusiform aneurysm at the junction of vertebrobasilar artery. A-C) The aneurysm was stent-coiled (AVE, arrows) and nearly complete obliteration was achieved. This patient did well after treatment and at 70-month follow up.

described patients with fusiform and dissecting aneurysms in whom endovascular stenting alone, without coiling, led to aneurysm thrombosis [8]. However, two patients with giant BA trunk aneurysms treated with stenting alone died of rebleeding within one month after the procedure. Stent-assisted coiling is typically reserved for those cases deemed surgically high-risk in conjunction with a wide-necked aneurysm not amenable to traditional coil embolization alone. The technique of placing a second stent inside a previously deployed stent has been described in the basilar artery by other authors [1,6,25,29]. The Neuroform stent system is highly flexible and is large enough to allow the passage of a second stent system through the cells of the first. Thus, the stent construction allows for ready creation of a layered Y'd stent configuration at the BA apex. Important considerations when creating the Y'd configuration at the basilar bifurcation include the use of a 20 and 15-mm stent. During placement of the first Y'd stent system, migration of the first stent may occur during placement of the second stent, although we did not encounter it in our patient. Thus, we place the 20-mm stent first to allow movement and maintain the construction. Giant and fusiform aneurysms of the BA are still challenging for neurosurgeons.

Three cases of basilar trunk endovascular occlusion have been reported in cases of giant and fusiform aneurysms of the BA with good outcomes [30]. We present one patient with an upper BA fusiform aneurysm treated successfully with endovascular occlusion of the aneurysm and basilar trunk. In determining the safety of BA occlusion, the most important factor to be taken into account is the presence and size of the PCoAs [11,24,30]. Therefore, before occlusion of the BA during therapy is considered, documentation of the presence and size of the PCoAs is essential. Steinberg et Al reported the surgical outcome of 201 patients undergoing an attempted Hunterian ligation without any revascularization in which a successful long-term outcome was achieved in 75% of patients. They reported that the size of the posterior communicating artery was a good predictor of tolerance to BA occlusion. The presence of an adequate caliber of the posterior communicating artery allowed retrograde filling of the BA. In this series, we encountered three patients with vertebrobasilar atheromatous fusiform aneurysms (figure 5). All of them had history of hypertension lasting more than ten years. Two patients were treated with aspirin and one with GOS 5 at three year follow-up and one with GOS 4 because of hypogeusia at two year follow-up. One patient treated with bilateral vertebral artery occlusion eventually died of BA thrombosis one month after the procedure. A series of vertebrobasilar aneurysms treated by parent vessel occlusion, reported delayed neurological complications in 45% of the cases and the majority of these were found to be fatal [11,24,30]. These data may assist in guiding medical or endovascular therapy and will improve counseling of patients with vertebrobasilar atheromatous fusiform aneurysm. The immediate risk of endovascular treatment needs to be weighed against the predicted annual likelihood of morbidity and mortality from this disease in patients with risk factors commonly seen in the stroke population. Recently, warfarin use was more strongly associated with reduced mortality, which may suggest a prophylactic role for warfarin in vertebrobasilar atheromatous fusiform aneurysm [31]. Whether prophylactic warfarin therapy would be beneficial in preventing strokes in asymptomatic patients needs to be better evaluated with larger prospective studies. The benefit of warfarin use will have to outweigh the potential risk of subarachnoid hemorrhage from vertebrobasilar dolichoectasia rupture and other systemic bleeding complications

Clinical Outcome after Treatment

Given the generally poor natural history of untreated BAAs, we consider our overall long-term good outcome (GOS Score 4-5) of 100% for BA apex aneurysms and 78.6% for BA trunk aneurysms to reflect favorable clinical results. Reported results for BA and VBJ aneurysms were less favorable: good outcome (GOS 4-5) in 39% and death in 44% [5,9,11,14,23,25,29,30]. In our series, 9.3% (4 out of 43) of periprocedural deaths in our series were caused by SAH and ischemia. Good results were reported in 72% of the 46 BA cases managed surgically in Steinberg et Al's study (24% mortality rate) (28), whereas only 26% of the patients were reported to make a good recovery (mortality rate 53%) in a report by Amin-Hanjani et Al [2]. Yoshimoto et Al reported favorable outcomes in four (40%) out of ten patients with BA fusiform aneurysms, of whom four presented with SAH [32]. Two of the four patients who presented with SAH underwent endovascular treatment. One of the two patients died and the other had a good recovery. In our present series 40 (93%) of the 43 patients with BAAs were treated endovascularly: seven (90.7%) fared well at long-term follow-up, and four died (9.3%). Hunterian proximal ligation or endovascular parent vessel occlusion of VBJ aneurysms has resulted in virtually complete thrombosis in 46 to 84% of patients in previous studies [20,28] and 60% in the present series. Incomplete thrombosis was associated with a poor subsequent natural history due to rebleeding and increased mass effect or brainstem ischemia in our study and prior reports [20,28]. When a fusiform aneurysm is located in the basilar trunk, its treatment is quite challenging. The main treatment strategy in a large surgical series of 32 cases of giant basilar trunk aneurysms of the fusiform type was proximal basilar artery occlusion or trapping, resulting in seven cases of mortality and five cases of morbidity [5]. Another series reported three mortalities among eight cases of basilar artery fusiform aneurysm [3]. The placement of a flexible stent, followed by coil embolization may be a legitimate treatment option in eccentrically bulging, broad-neck aneurysms. Stent placement has the advantage of allowing the maintenance of the parent arterial lumen, but clinical use of this technique is limited by the difficulty associated with the endovascular navigation of stents into the cerebral vasculature. This treatment modality using stents appears infeasible in cases such as our own with

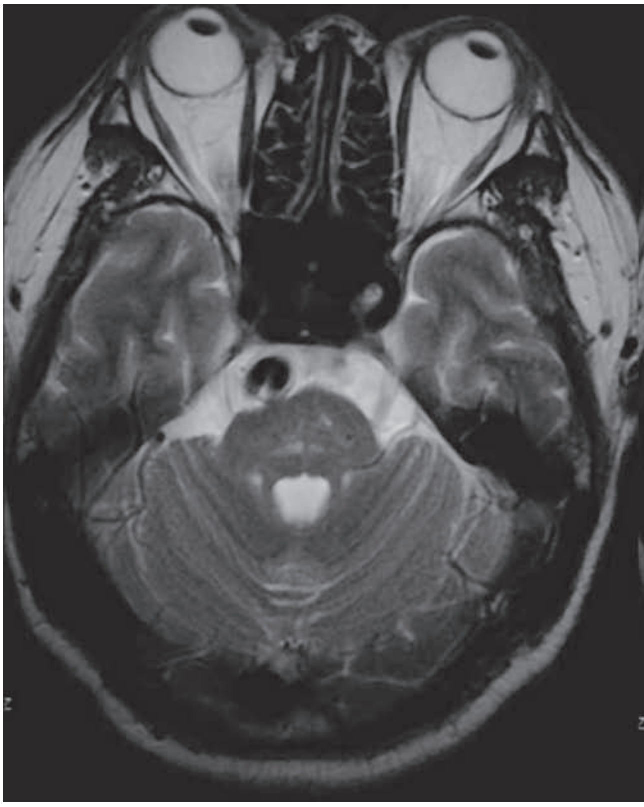

A

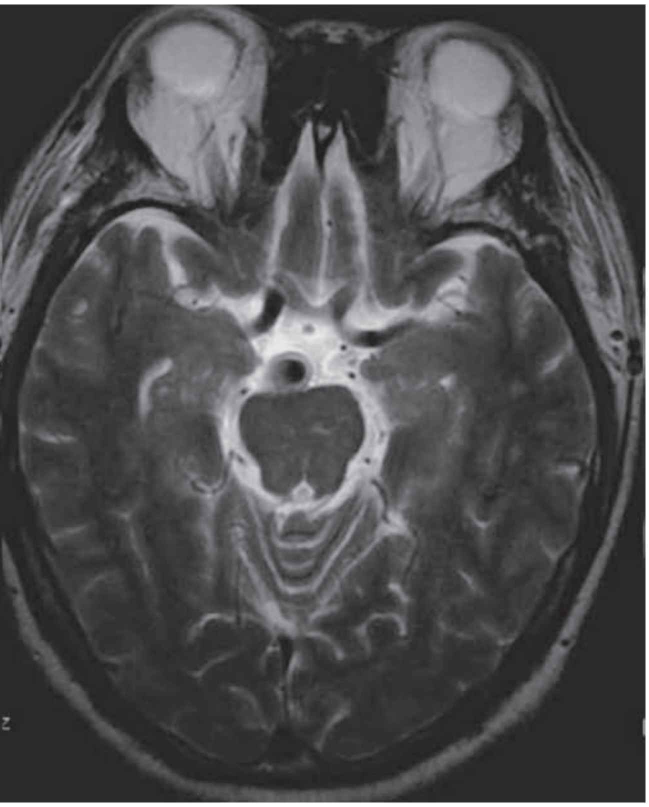

B

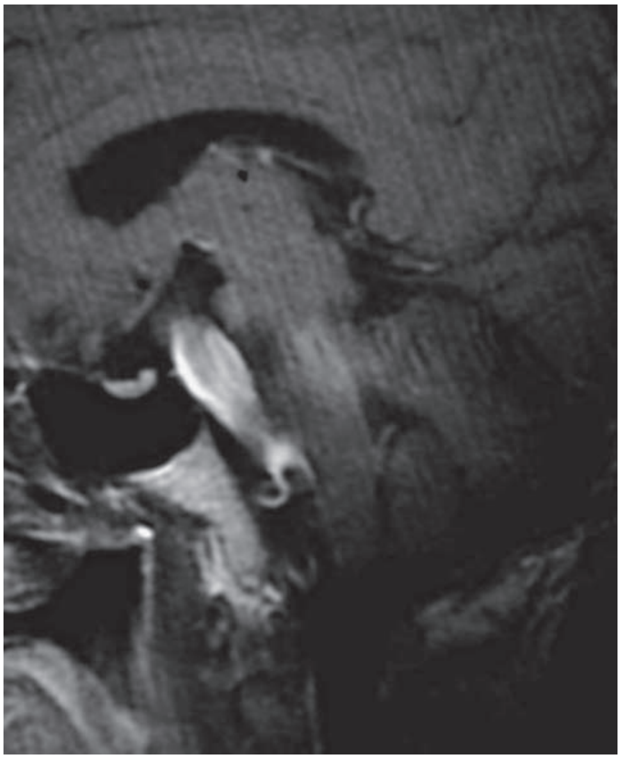

C

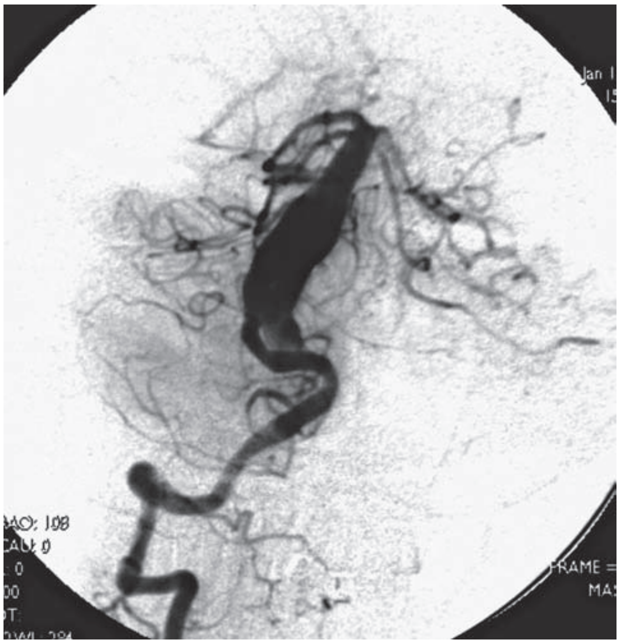

D

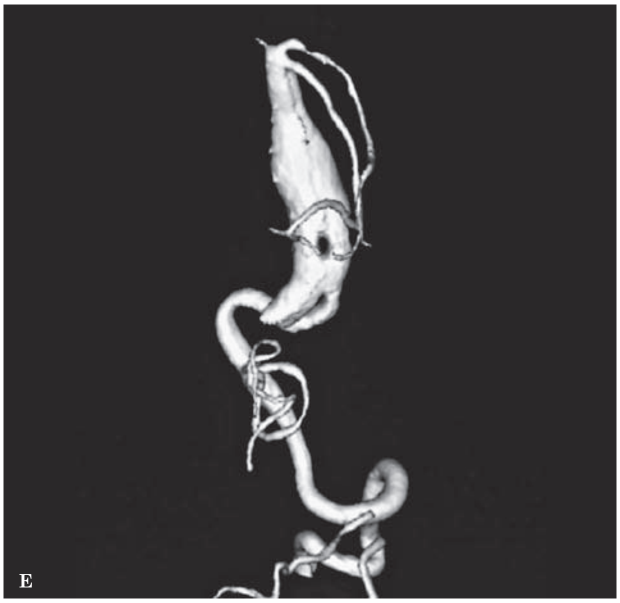

Figure 5 Magnetic resonance (MR) images and angiograms acquired in a 54-year-old man who presented with four limbs numbness. The MR images (axial T2-weighted (A) and sagittal T1-weighted (B)) reveal a 15-mm atheromatous fusiform aneurysm of the left VA reaching to the BA and causing the brainstem infarction (arrow). C,D) show the atheromatous fusiform aneurysm involving the fenestrated BA. This patient treated with antiplatelet and anti-hypertension medications. At 24 months his numbness resolved but developed hypogeusia, and his outcome was designated a GOS score of 4.

extremely tortuous arteries. With regard to a good clinical outcome in our study as opposed to poorer outcome in previous reports, we think that the development of endovascular technique skills has produced better results. Another important factor for good outcome in our series may be that the majority of pediatric patients were in a good state on admission.

Conclusions

Technical advances and growing experience with endovascular techniques have resulted in an increased percentage of patients undergoing endovascular therapies. Favorable overall long-term outcome can be achieved in patients with BA apex aneurysms, 78.6% of those with BA trunk aneurysms when using endovascular techniques. Treatment of fusiform aneurysms resulted in immediate worsening of the neurological condition in 16.3% of patients, but overall long-term results were good in 90.3% of patients with 9.3% of these patients dying of SAH or brainstem compression/ischemia. To manage of BAAs, endovascular surgery should be considered as the first choice.

Advances in new device technology will allow us to provide better clinical and anatomical outcomes for such challenging lesions.

References

1 Akpek S, Morsi H, Benndorf G et Al: Reconstruction of the Basilar Tip with T Stent Configuration for Treatment of a Wide-Neck Aneurysm. Journal of Vascular and Interventional Radiology 15: 1024-1026, 2004.
2 Amin-Hanjani S, Ogilvy CS. Buonanno FS et Al: Treatment of dissecting basilar artery aneurysm by flow reversal. Acta Neurochir (Wien) 139: 44-51, 1997.
3 Aoki N, Sakai T: Rebleeding from intracranial dissecting aneurysm in the vertebral artery. Stroke 21: 1628-1631, 1990.
4 Bendok BR, Getch CC, Parkinson R et Al: Extended lateral transsylvian approach for basilar bifurcation aneurysms. Neurosurgery 55: 174-178, 2004.
5 Buckle C, Rabadi MH: Bilateral pontine infarction secondary to basilar trunk saccular aneurysm. Arch Neurol 63: 1498-1499, 2006.
6 Chow MM, Woo HH, Masaryk TJ et Al: A novel endovascular treatment of a wide-necked basilar apex aneurysm by using a Y-configuration, double-stent technique. Am J Neuroradiol 25: 509-512, 2004.
7 Clarke M: Systematic review of reviews of risk factors for intracranial aneurysms. Neuroradiology 50: 653-664, 2008.
8 Fiorella D, Albuquerque FC, Deshmuck VR et Al: Endovascular reconstruction with the Neuroform stent as monotherapy for the treatment of uncoilable intradural pseudoaneurysms. Neurosurgery 59: 291-300, 2006.
9 Gruber DP, Zimmerman GA, Tomsick TA et Al: A comparison between endovascular and surgical management of basilar artery apex aneurysms. J Neurosurg 90: 868-874,1999.
10 Hassan T, Ezura M, Takahashi A: Treatment of giant fusiform aneurysms of the basilar trunk with intra-aneurysmal and basilar artery coil embolization. Surg Neurol 62: 455-462, 2004.
11 Henkes H, Liebig T, Reinartz J et Al: Endovascular occlusion of the basilar artery for the treatment of dissecting and dysplastic fusiform aneurysms. Nervenarzt 77: 192-196, 2006.
12 Higa T, Ujiie H, Kato K et Al: Basilar artery trunk saccular aneurysms: morphological characteristics and management. Neurosurg Rev DOI 10.1007/s10143-008-0163-3
13 Hsu FP, Clatterbuck RE, Spetzler RF: Orbitozygomatic approach to basilar apex aneurysms. Neurosurgery 56: 172-177, 2005.
14 Hua SE, Gluckman TJ, Batjer HH: Middle cerebral artery occlusion after pterional approach to basilar bifurcation aneurysm: technical case report. Neurosurgery 39: 1050-1054,1996.
15 Jabbour R, Khalifeh R, Al Kutoubi A et Al: Dissecting aneurysm of the basilar trunk in a young man. Arch Neurol 60: 1016-1018, 2003.
16 Jahromi BS, Mocco J, Bang JA et Al: Clinical and angiographic outcome after endovascular management of giant intracranial aneurysms. Neurosurgery 63: 662-675, 2008.
17 Krisht AF, Kadri PA: Surgical clipping of complex basilar apex aneurysms: a strategy for successful outcome using the pretemporal transzygomatic transcavernous approach. Neurosurgery 56: 261-273, 2005.
18 Krisht AF, Krayenbühl N, Sercl D et Al: Results of microsurgical clipping of 50 high complexity basilar apex aneurysms. Neurosurgery 60: 242-252, 2007.
19 Lawton MT, Daspit CP, Spetzler, RF: Technical aspects and recent trends in the management of large and giant midbasilar artery aneurysms. Neurosurgery 41: 513-521, 1997.
20 Leibowitz R, Do HM, Marcellus ML et Al: Parent vessel occlusion for vertebrobasilar fusiform and dissecting aneurysms. Am J Neuroradiol 24: 902-907, 2003.
21 Lozier AP, Kim GH, Sciacca RR et Al: Microsurgical treatment of basilar apex aneurysms: perioperative and long-term clinical outcome. Neurosurgery 54: 286-299, 2004.
22 Lv X, Li Y, Liu A, Wu Z: Endovascular management of multiple cerebral aneurysms in acute subarachnoid hemorrhage associated with fenestrated basilar artery: a case report and review of the literature. Neuroradiol J 21: 137-142, 2008.
23 Mack WJ, Ducruet AF, Angevine PD et Al: Deep hypothermic circulatory arrest for complex cerebral aneurysms: lessons learned. Neurosurgery 62: 1311-1323, 2008.
24 Omahen DA, Findlay JM: A giant fusiform basilar aneurysm treated by bilateral vertebral artery occlusion. J Clin Neurosci 11: 324-328, 2004.
25 Perez-Arjona E, Fessler RD: Basilar artery to bilateral posterior cerebral artery 'Y stenting' for endovascular reconstruction of wide-necked basilar apex aneurysms: report of three cases. Neurol Res 26: 276-281, 2004.
26 Redekop GJ, Durity FA, Woodhurst WB: Management-related morbidity in unselected aneurysms of the upper basilar artery. J Neurosurg 87: 836-842,1997.
27 Russell SM, Nelson PK, Jafar JJ: Neurological deterioration after coil embolization of a giant basilar apex aneurysm with resolution following parent artery clip ligation. Case report and review of the literature. J Neurosurg 97: 705-708, 2002.
28 Steinberg GK, Drake CG, Peerless SJ: Deliberate basilar or vertebral artery occlusion in the treatment of intracranial aneurysms. Immediate results and long-term outcome in 201 patients. J Neurosurg 79: 161-173, 1993.
29 Thorell WE, Chow MM, Woo HH et Al: Y-configured dual intracranial stent-assisted coil embolization for the treatment of wide-necked basilar tip aneurysms. Neurosurgery 56: 1035-1040, 2005.
30 Wenderoth JD, Khangure MS, Phatouros CC et Al: Basilar trunk occlusion during endovascular treatment of giant and fusiform aneurysms of the basilar artery. Am J Neuroradiol 24: 1226-1229, 2003.
31 Wolfe T, Ubogu EE, Fernandes-Filho JA et Al: Predictors of clinical outcome and mortality in vertebrobasilar dolichoectasia diagnosed by magnetic resonance angiography. Journal of stroke and cerebrovascular diseases 17: 388-393, 2008.
32 Yoshimoto Y, Hoya K, Tanaka Y et Al: Basilar artery dissection. J Neurosurg 102: 476-481, 2005.

Xianli Lv, MD
Beijing Neurosurgical Institute
Beijing Tiantan Hospital
Capital Medical University
6, Tiantan Xili, Chongwen
Beijing, 100050 P.R.China
Tel.: 86-10-67098850
Fax: 86-10-95105105
E-mail: lvxianli000@163.com
