# Supplementary material for: Management strategies and outcomes of basilar trunk aneurysms: a systematic review and meta-analysis
Source: Langenbecks Arch Surg. 2026 Jan 20;411(1):71. doi: 10.1007/s00423-025-03959-3 (PMC12852281; doi:10.1007/s00423-025-03959-3)
Supplement: Supplementary file 1 — Supplementary file1 (ZIP 55327 KB) [file 423_2025_3959_MOESM1_ESM.zip › literature.zip!/meta/saliou 2015.pdf]

Original Contribution

Natural History and Management of Basilar Trunk Artery Aneurysms

Guillaume Saliou, MD, PhD; Raphael H. Sacho, MD; Sarah Power, MD, PhD; Alex Kostynskyy, MD; Robert A. Willinsky, MD; Michael Tymianski, MD, PhD; Karel G. terBrugge, MD, PhD; Sapna Rawal, MD; Timo Krings, MD, PhD

Background and Purpose—Basilar trunk aneurysms (BTAs), defined as aneurysms distal to the basilar origin and proximal to the origin of the superior cerebellar artery, are rare and challenging to manage. We describe the natural history and management in a consecutive series of BTAs.

Methods—Between 2000 and 2013, 2522 patients with 3238 aneurysms were referred to our institution for aneurysm management. A retrospective review of this database was conducted to identify all patients with BTAs.

Results—In total, 52 patients had a BTA. Mean age was 56 (SD±18) years. Median clinical follow-up was 33 (interquartile range, 8–86) months, and imaging follow-up was 26 (interquartile range, 2–80.5) months. BTAs were classified into 4 causal subtypes: acute dissecting aneurysms, segmental fusiform ectasia, mural bleeding ectasia, and saccular aneurysms. Multiple aneurysms were more frequently noticed among the 13 saccular aneurysms when compared with overall population (P=0.021). There was preponderance of segmental ectasia or mural bleeding ectasia (P=0.045) in patients presenting with transit ischemic attack/stroke or mass effect. Six patients with segmental and 4 with mural bleeding ectasia demonstrated increasing size of their aneurysm, with 2 having subarachnoid hemorrhage caused by aneurysm rupture. None of the fusiform aneurysms that remained stable bled.

Conclusions—BTAs natural histories may differ depending on subtype of aneurysm. Saccular aneurysms likely represent an underlying predisposition to aneurysm development because more than half of these cases were associated with multiple intracranial aneurysms. Intervention should be considered in segmental ectasia and chronic dissecting aneurysms, which demonstrate increase in size over time as there is an increased risk of subarachnoid hemorrhage. **(*Stroke*. 2015;46:00-00. DOI: 10.1161/STROKEAHA.114.006909.)**

American Heart Association | American Stroke Association

Key Words: dissecting aneurysm ■ fusiform aneurysm ■ saccular aneurysm

Stroke

Basilar trunk artery is defined as the arterial segment from the origin of the basilar artery up to the origin of the superior cerebellar artery. Therefore, by definition, basilar tip and superior cerebellar artery aneurysms are excluded from the basilar trunk aneurysms (BTAs). BTAs are rare[1,2] and pose significant challenges to the treating physician: their cause is diverse, their natural history is poorly understood, and their treatment is challenging. Their 5-year morbidity–mortality is high, described as 80%,[3] and rupture of BTA is associated with a poor prognosis and high mortality rate within the first 48 hours.[4] Acute and chronic dissecting aneurysms of the basilar trunk are known to have severe morbidity and 20% to 30% mortality.[5] The higher frequency of giant fusiform aneurysms in the basilar trunk compared with the anterior circulation may reflect segmental vulnerability of this arterial system as previously proposed.[6] Management is challenging because of difficult surgical access, frequent occurrence of a broad-neck, and the incorporation of the perforating branches of the BA within the aneurysm. Treatment decisions are often complicated by an unpredictable clinical course and controversies on treatment strategy. Besides case reports, few large series have been published in the pertinent literature on management of these aneurysms.[1–3,7–9] A better understanding of both the incidence and the natural history of these aneurysms may help to improve management strategies. Therefore, we describe herewith a single-center experience of the epidemiology, natural history, and management in 52 consecutive BTAs.

Materials and Methods

Patient Population

Between January 2000 and September 2013, 2522 patients were referred to our institution for the management of a cerebral aneurysm (58.3% unruptured and 41.7% ruptured; Figure 1). There were a total

Received August 5, 2014; final revision received December 26, 2014; accepted January 21, 2015.
From the Department of Neuroradiology, Hôpital Bicêtre, Le Kremlin Bicetre, France (G.S.); and Division of Neuroradiology, Department of Medical Imaging (G.S., R.H.S., S.P., A.K., R.A.W., K.G.t., S.R., T.K.) and Division of Neurosurgery (M.T.), Toronto Western Hospital, Toronto, Ontario, Canada.
Guest Editor for this article was Louis Caplan, MD.
Correspondence to Guillaume Saliou, MD, PhD, Service de Neuroradiologie, Hôpital Bicêtre, 78 Rue du Général Leclerc, 94275 Le Kremlin Bicetre, France. E-mail guillaume.saliou@bct.aphp.fr
© 2015 American Heart Association, Inc.

of 3238 aneurysms in the overall population. A total of 3100 aneurysms were sacculars and 138 were fusiforms. A percentage of 23 patients had ≥2 aneurysms. The mean age was 55.6 years (SD, 14.5; range, 0–96 years). A percentage of 28 population was men. A retrospective medical chart and imaging review of a prospectively collected neurovascular database were conducted after approval by our local Research Ethics Board to identify all patients with BTAs. Moreover, to exclude the possibility that patients may have been missed from this consecutive, prospectively collected database, we also performed a key word search from all reports of intracranial vascular imaging performed during the same time period at our institution (computed tomographic angiography [CTA]/MR angiography/catheter angiogram). All charts and their imaging features (head CTA, MRI, and cerebral angiography) of patients with identified posterior cerebral circulation aneurysm were reviewed by the first author.

BTAs were defined as aneurysms located distal to the origin of the basilar artery and proximal to the origin of the superior cerebellar artery. Basilar tip aneurysms (n=169) and superior cerebellar artery aneurysms (n=49) were excluded. Patients with aneurysms located on distal branches arising from the basilar artery (n=2) and arteriovenous malformation–related aneurysms (n=4) were excluded from the study. Patients with acute hemorrhagic or ischemic basilar artery dissection without evidence of an aneurysmal dilatation were also excluded (n=2). Patients with dolichoectatic fusiform aneurysms that involved either the vertebral artery (n=5) or the basilar tip (n=1) were included.

BTAs were selected and classified on review of head MRA, CTA, and digital subtraction angiography images based on previous publications[10–12] into 4 causal subtypes (Figure 2): acute dissecting aneurysms, segmental fusiform ectasia, mural bleeding ectasia, and saccular aneurysms. Acute dissecting aneurysms (Mizutani type 1) were defined as aneurysms that were not located at a bifurcation and that demonstrated T1 hyperintense signal on MR indicating acute mural hematoma, with or without associated subarachnoid hemorrhage (SAH). The acute dissecting aneurysms demonstrated focal narrowing preceding the arterial dilatation. Segmental ectasia (Mizutani type 2) was defined as fusiform dilatation of the lumen without intra- or extraluminal thrombus formation. Chronic mural bleeding ectasia was defined as a chronic type of intramural bleeding because of either chronic dissection or bleeding from vasa-vasorum in underlying atherosclerotic vessels (Mizutani type 3 or previously coined dolichoectatic chronic fusiform dissecting aneurysm and atherosclerotic fusiform aneurysms) or not. Saccular aneurysm included saccular aneurysms arising from the main arterial trunk (Mizutani type 4) and saccular bifurcation aneurysms (eg, at local nonfused segments, or the anterior inferior cerebellar artery).

Additional imaging features that were recorded included morphological aneurysm subtypes (saccular versus fusiform), aneurysm size, change in aneurysm size over imaging follow-up, and exact aneurysm location.

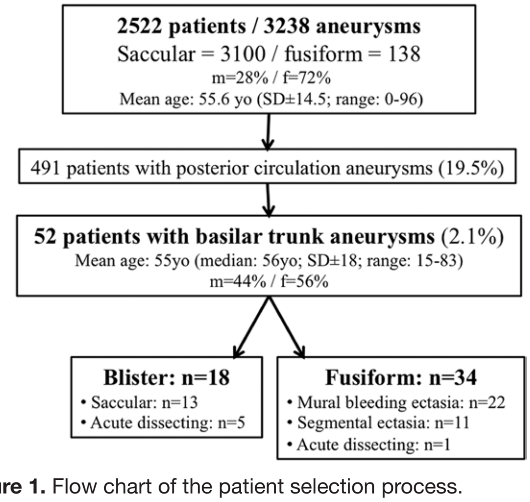

Figure 1. Flow chart of the patient selection process.

Clinical Features

Initial presentation was classified as incidental, SAH, or focal neurological deficit related to the aneurysm. Epidemiological data, risk factors, pertinent medication, and associated comorbidities were recorded including age, sex, family history, multiple aneurysms, high blood pressure, smoking, high lipid level, statin, and antiplatelet/anticoagulation medication. All therapeutic procedures (endovascular or surgical) were retrospectively analyzed.

Clinical follow-up was assessed by the modified Rankin Scale for all patients. For the subgroup of patients who had a SAH, the World Federation of Neurosurgery Society Scale was evaluated to assess initial outcome and the Glasgow Outcome Scale for the late outcome.

Statistical Analysis

Descriptive statistics were performed to summarize the distribution of clinical features across BTA types (Table 1). The distribution of categorical variables was described by frequencies and percentages, continuous and normally distributed variables by mean and SD, and continuous and non-normally distributed variables by medians and interquartile ranges. Univariate statistics were performed to assess for associations between clinical features and BTA types. These were performed via ANOVA (comparison of continuous variables with normal distribution), and χ^2 or Fisher exact tests (comparison of categorical variables). The Shapiro–Wilk test was used to test for normality. A P value <0.05 was considered statistically significant. Given the small size of the sample, it was not possible to create a valid model to perform multivariable analyses.

Results

Among the 2522 patients, 491 (19.5%) patients had ≥1 brain aneurysm in the posterior circulation (53.6% unruptured and 47.3% ruptured), and there were 52 patients (2.1%) in our population with aneurysms of the basilar trunk (Figure 3).

Among the 52 selected patients, 12 (23%) had multiple intracranial aneurysms. Three patients had a second aneurysm located in the basilar tip (n=2) or superior cerebellar artery (n=1) and 9 patients had ≥1 aneurysm located at other intracranial sites. The mean age was 56 (SD±18) years. We had a median clinical follow-up of 33 (interquartile range [IQR], 8–86) months and median imaging follow-up of 26 (IQR, 2–80.5) months. There were 29 (56%) women and 23 (44%) men.

Aneurysm Type and Outcome

For each causal type of aneurysm, the initial presentation, vascular risk factors, and outcomes are summarized in Table 1. A definite diagnostic of acute dissecting aneurysm with identification of an acute vessel wall hematoma was available in all but 1 patient in whom no vessel wall hematoma was identified, but major changes of the aneurysm (increase of size) occurred after 2 weeks on repeated imaging. A definite diagnostic of mural bleeding ectasia was available for all patients. Vessel wall thickening with clot deposition was identified on CTA (4 patients), MRA (2 patients), or combination of ≥2 examinations between MRI, CTA, and digital subtraction angiography (16 patients). Patients diagnosed with segmental ectasia were examined by CTA (3 patients), MRA (3 patients), or both (5 patients). For those patients, no vessel wall thickening or clot deposition was identified.

The median maximal diameter was 5 (IQR, 3–9) mm for saccular aneurysms, 8 (IQR, 5–10) mm for acute dissecting aneurysms, 10 (IQR, 7.5–14.5) mm for segmental ectasia, and 15 (IQR, 11–21) mm for mural bleeding ectasia. None of segmental ectasia and mural bleeding ectasia had SAH at

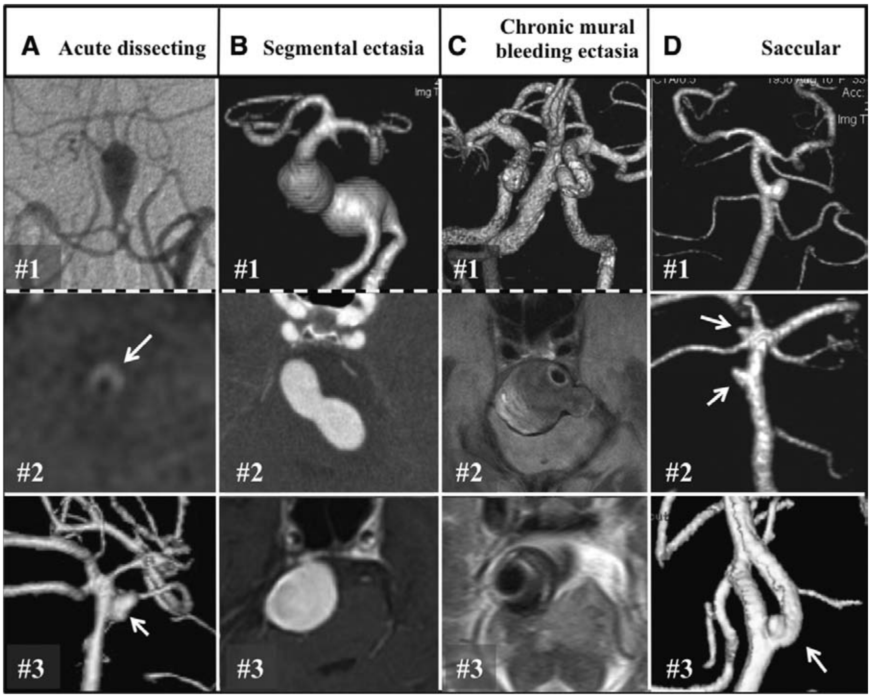

Figure 2. Examples in classification of basilar trunk aneurysms. For **A**–**C**, 1 and 2 are in the same patient. Acute dissecting aneurysm (**A**) had acute symptoms and wall hematoma (2: arrow, axial T1SE-weighted sequence). Segmental ectasia (**B**) without any clot/dissection seen in the wall. Chronic dissecting aneurysms (**C**), with multiple layers of dissection/thrombus in the wall. Saccular aneurysm (**D**) branching (1 and 3) or nonbranching (2).

onset. Both together were mainly incidental (64%). However, 4 patients with segmental ectasia (36%) presented with stroke and none of them with compressive symptoms. Moreover, 6 patients (27%) with mural bleeding ectasia presented with stroke and 2 (9%) with isolated compressive symptoms. Both stroke and isolated compressive symptoms at onset (neurological deficit) were more frequently seen in mural bleeding ectasia and segmental ectasia (P=0.045). Conversely, acute dissecting aneurysms mainly presented with SAH at onset (83%, n=5) or with ischemic stroke (17%, n=1). None of them was incidental. Finally, saccular aneurysms were mainly incidental (62%) or with SAH (38%). There was a preponderance of acute dissecting or saccular aneurysms (P<0.001) in patients with SAH at onset.

Among the 13 saccular aneurysms, 7 were located in a branching segment (6 in an anteroinferior cerebellar artery

Table 1. Clinical Data at Onset and Last Imaging Outcome According to the Type of Aneurysm

Type of Aneurysm	Acute Dissecting	Segmental Ectasia	Mural Bleeding Ectasia	Saccular	*P* Value
n	6	11	22	13	…
Mean age, y (±SD)	44 (±23)	56 (±18)	61 (±18)	53 (±12)	…
Median FU (IQR)	11 (1–54)	33 (10–69)	24 (1–75)	84 (11–83)	…
Men (%)	2 (33)	6 (55)	13 (59)	2 (15)	0.062
SAH	5 (83%)	0	0	5 (38%)	<0.001
Neurological deficit	1 (17%)	4 (36%)	8 (36%)	0	0.045
Stroke	1	4	6	0	0.09
Compressive symptoms	1	0	2	0	NS
Incidental	0	7 (64%)	14 (64%)	8 (62%)	…
Multiple aneurysms	0	2 (18%)	3 (14%)	7 (54%)	0.021
≥2 risk factors*	1 (17%)	5 (45%)	12 (54%)	5 (38%)	NS
Follow-up					
Increase	0	6 (67%)†	4 (25%)‡	0§	…
Rupture	0	1 (9%)	1 (4%)	0	…
Stroke	0	0	3 (14%)	0	…
Compressive symptoms	0	0	0	0	…

Deficit related to the aneurysm among ischemic stroke, transit ischemic attack, or mass effect (only 2 patients, 1 among segmental ectasia and 1 among mural bleeding ectasia had isolated mass effect). FU indicates follow-up; IQR, interquartile range; SAH, subarachnoid hemorrhage.

*≥2 risks factors among high blood pressure, smoking, diabetes mellitus, and high cholesterol level.

†Data not available in 2 patients.

‡Data not available in 6 patients.

§Data not available in 3 patients.

American Heart Association | American Stroke Association
Stroke

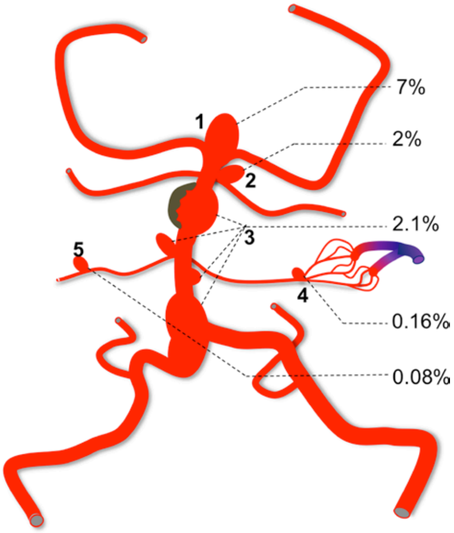

Figure 3. Epidemiological considerations in overall basilar artery aneurysms among a database of 2425 patients. (1) Basilar tip (n=169), (2) superior cerebellar artery (n=49), (3) basilar trunk (n=52, present study), (4) arteriovenous malformation–related (n=4), and (5) distal branch (n=2).

and 1 in a trigeminal artery), 2 were located on an unfused basilar artery, and 3 were located in a nonbranching segment. For these patients, multiple aneurysms were noticed in 54% (n=7; *P*=0.021), which is significantly larger when compared with the overall multiplicity rare of our database (20%).

During follow-up, 10 patients (6 with segmental ectasia and 4 with mural bleeding ectasia) presented with an increase in the size of their aneurysm (Tables 1 and 2). Among them, 2 patients had a SAH because of aneurysm rupture and died (1 segmental ectasia: maximal diameter increase from 17 to 20 mm in 15 months of follow-up and 1 mural bleeding ectasia: maximal diameter increase from 15 to 17 mm in 27-month period) and 1 patient (mural bleeding ectasia: maximal diameter increase from 11 to 38 mm in 101-month follow-up period) died from an ischemic brain stem stroke 2 days after surgical treatment with a bypass (Table 3, patient no 34). An increase in size of aneurysm seemed to be associated to a higher incidence of bleeding (*P* value not applicable) because only 2 aneurysms who increased their size ruptured and none of those stable ruptured (Table 2). Two patients with mural bleeding ectasia had spontaneous ischemic stroke attributed to the aneurysm without any increase in aneurysm size. One of them died and the other had a good recovery (modified Rankin Scale 1 at 3 months). If under appropriate antiplatelet or anticoagulant therapy, stroke recurrence occurred in 16% of mural bleeding ectasia and no stroke recurrence occurred in patients harboring segmental ectasia.

None of the saccular aneurysms that were not treated or before treatment increased in size or had a new episode of bleeding. Among the 10 fusiform aneurysms that increased in size over time (Table 2), the median increase was 1 mm/y. They had a higher frequency of vascular risk factors (80% versus 33% in stable aneurysms; *P* value not applicable), were more frequently men (90% versus 53% in stable aneurysms; *P* value not applicable), and involved more frequently segmental ectasia (60%) than mural bleeding ectasia (40%; *P* value not applicable).

Treatment Considerations

Eighteen patients underwent treatment (ie, endovascular procedure or surgery) for their aneurysm (Table 3). Among the 10 patients who had a SAH at onset, 8 were treated and 2 died before treatment. Three of them had surgical clipping (2 patients) or trapping (1patient). Four patients had aneurysm coiling, 1 patient a stent assisted coiling, and 1 patient had a flow diverter placement. None of the patients who survived experienced a treatment complication. Two treated patients (coiling alone) had a neck recurrence, which did not require any further treatment. Five chronic unruptured dissecting aneurysms were treated. Three of them presented with ischemic symptoms related to the aneurysm and were treated by an open surgical approach. Two of them who underwent flow reversal and posterior circulation bypass died of surgical complications. The single patient who had an isolated posterior circulation bypass had a good outcome. Two patients who presented with an incidental finding of a chronic dissecting aneurysm were treated as well (1 patient endovascular flow reversal alone and 1 patient flow reversal and posterior circulation surgical bypass). Both of these patients had a good outcome.

Discussion

The percentage of BTAs among the overall aneurysm locations (2.1%) highlights that these types of aneurysms are exceptionally rare.

Saccular Aneurysms

Our findings are consistent with previous reports on saccular aneurysms studies that basilar aneurysms occur more

Table 2. Size Changes in Mural Bleeding and Segmental Ectasia Aneurysms Over Time and Outcome

Size Over Time	n	Median Imaging (m) FU (IQR)	Mural Bleeding Ectasia	Segmental Ectasia	Men (%)	≥2 Risk Factors*	Statin	Mean Dose Statin, mg	% Antiplatelet or Anticoagulation	FU: SAH	FU: Ischemia
Stable	15	15 (8–88)	12 (80%)	3 (20%)	8 (53%)	6 (40%)	9 (60%)†	21	67	0	2
Increase	10	41 (28–96)	4 (40%)	6 (60%)	9 (90%)	9 (90%)	7 (78%)†	60	100†	2	1‡
Unknown	8	NA	6 (75%)	2 (25%)	2 (25%)	2 (25%)	0	0	43†	0	0

FU indicates follow-up; IQR, interquartile range; and SAH, subarachnoid hemorrhage.
*≥2 risk factors among high blood pressure, high lipid level, diabetes mellitus, and smoking.
†Data not available for 1 patient.
‡One patient died 2 days after surgery (bypass) from brain stem infarction.

Table 3. Endovascular/Surgical Aneurysm Treatment and Outcome

Patient Number	Sex	Age, y	Type of Aneurysm	Symptoms at Onset	Score at Onset	Treatment	Treatment-Related Complication	Recurrence	Score at Last Outcome	Mean FU Months
2	M	16	Acute dissecting	SAH	WFNS 2	Coils+stent	Stent misplaced	No	GOS 5	22
3	F	59	Saccular	Incidental	mRS 0	Stent+coils	None	No	mRS 0	62
4	F	48	Saccular	Incidental	mRS 0	Clipping	None	No	mRS 0	61
5	F	53	Chronic dissecting	Incidental	mRS 1	Flow reversal	None	No	mRS 0	2
6	F	45	Saccular	Incidental	mRS 0	Clipping	None	Yes*	mRS 0	112
8	F	46	Acute dissecting	SAH	WFNS 1	Coils	None	Yes*	GOS 5	18
17	M	59	Saccular	SAH	Unknown	Coiling	None	Yes*	GOS 5	109
21	F	71	Acute dissecting	SAH	mRS 1	FD alone	None	No	mRS 0	4
26	M	55	Chronic dissecting	Brain stem stroke	mRS 4	Elana bypass	Died: brain stem infarction	No	mRS 6	104
28	F	52	Chronic dissecting	Incidental	mRS 0	STA-PCA bypass+parent vessel occlusion	None	No	mRS 1	79
32	F	37	Saccular	SAH	WFNS 2	Clipping	None	No	GOS 4	89
34	M	52	Chronic dissecting	Brain stem stroke	mRS 3	Flow reversal and PCA bypass	Died: posterior fossa hematoma	NA	mRS 6	88
40	F	66	Saccular	SAH	WFNS 4	Trapping	None	No	GOS 1	0
41	F	33	Saccular	SAH	WFNS 1	Clipping	None	No	GOS 4	107
42	M	47	Saccular	Incidental	mRS 0	Coils+stent	None	No	mRS 0	104
46	F	51	Saccular	SAH	WFNS 1	Coils	None	No	GOS 1	84
51	M	18	Acute dissecting	Brain stem stroke	mRS 4	Parent vessel occlusion	None	No	mRS 4	75
52	F	30	Chronic dissecting	Diplopia	mRS 1	Bypass	None	No	mRS 2	42

F indicates female; FD, flow diverter stent; FU, follow-up; GOS, Glasgow Outcome Scale; M, male; mRS, modified Rankin Scale; SAH, subarachnoid hemorrhage; STA-PCA bypass, superficial temporal artery-posterior cerebral artery bypass; and WFNS, World Federation of Neurosurgery Society Scale.

*No retreatment required.

American Heart Association | American Stroke Association
Stroke

commonly in young female patients.[8] We found that 54% of the patients with saccular aneurysms harbored other intracranial aneurysms, which is higher than the usual frequency described at between 10% and 30% of patients.[14–16] Within this group, 2 patients had a confirmed family history of cerebral aneurysms and a strongly suspected family history for a third patient. None of them had an obvious genetic vascular disease indentified (although no specific testing was performed). All those considerations support the fact that there could be an underlying cerebrovascular vulnerability to form aneurysms in patients with saccular aneurysms in this rare location.

Acute Dissecting Aneurysms

Acute dissecting aneurysms have a high risk of rebleeding in the acute phase estimated to be between 43% and 86% in unsecured dissecting aneurysms or blood blister-like aneurysms,[10,17,18] with more than half of the rebleeds occurring in the first 24 hours after the initial SAH.[10] Among our 6 patients, 5 presented with SAH and 1 with a brain stem stroke. Two of them died before treatment from complications of the initial bleed. Three patients had a good outcome with modified Rankin Scale of 0 at 1 year, and the patient who had a stroke had a bad clinical outcome (modified Rankin Scale=4 at 1 year). The 4 surviving patients were successfully treated endovascularly with selective coiling (n=1), stent assisted coiling (n=1) of the dissecting aneurysm, parent vessel artery occlusion (n=1), or flow diverter placement through the dissecting artery (n=1). None of them rebled after treatment, and no procedural complications occurred. Nevertheless, controversies exist on the management of those aneurysms (deconstructive versus reconstructive). Parent artery occlusion, trapping or flow reversal are effective to prevent rebleeding with a relatively low rate of complications[17,19]; however, reconstruction of the parent artery with stent or flow diverters can also be considered in selective cases in the sight of recent publications.[9,18,20]

Segmental Ectasia

An important finding of the present series is that segmental fusiform ectasia seem to be as prone as chronic dissecting aneurysms to grow over time. These aneurysms have traditionally been considered to follow a benign course without clinical or imaging progression.[10] In the present study, 4 of 11 patients increased their aneurysm size over time over a mean imaging follow-up of 48 months (median, 22; SD±61; range, 0–175 months). A mechanism has previously been described for progression of chronic fusiform aneurysms is internal elastic lamina fragmentation.[21] At presentation, 4 patients had transit ischemic attacks or strokes attributed to the aneurysm although no thrombus formation or dissection was identified on imaging. The mechanism could be perforating arteries

injury because of an ongoing dissecting phenomenon. No treatment (endovascular or surgery) was performed in any of these patients. Secondary stroke prevention was successfully performed by antiplatelet therapy for the patients who had ischemic symptoms at onset, and none of these patients had an ischemic stroke on follow-up. On the contrary, 1 patient bled from his aneurysm during the follow-up and died.

Mural Bleeding Ectasia

One main difference between atherosclerotic and fusiform nonatherosclerotic aneurysms that has been previously described is the initial step of the disease: lipid deposition in atherosclerotic aneurysms and internal elastic lamina fragmentation in fusiform nonatherosclerotic aneurysms.[21] However, 58% of all patients with fusiform aneurysms in our study had atherosclerotic findings in their extracranial vasculature. Moreover, 54% of mural bleeding ectasia had ≥2 cardiovascular risk factors (including hypertension, smoking, hypercholesterolemia, or diabetes mellitus) and 27% (n=6) had history of coronary artery disease. Because of the frequency of associated atherosclerotic disease in the overall population of fusiform aneurysms,[8] this makes true atherosclerotic aneurysms difficult to differ from nonatherosclerotic ones without any histopathologic examinations. This would suggest that aneurysms with intramural bleeding, like the so-called dolichoectatic dissecting aneurysms (type 3 from Mizutani classification), are probably similar to atherosclerotic aneurysms in term of outcome and ischemic risk. They probably evolve and grow in similar ways with intramural bleeding (either mural dissection or bleeding from vasa-vasorum) as previously proposed,[11] which is why we do not further subdifferentiate these aneurysms. They have been shown to be associated with paramedian pontine infarction,[5] which is supported in the present series as 30% of mural bleeding ectasia presented with an ischemic stroke (TIA or stroke) and 2 of them had an ischemic stroke during the follow-up.

This study has the usual limitations of retrospective studies. Moreover, no clinical or imaging follow-up was respectively available for 1 (2%) and 10 (20%) patients who could have biased our findings.

Conclusions

BTAs are rare. We have shown using a causal classification based on imaging that the natural history may differ depending on the subtype of aneurysm. There also seem to be unique sex differences within the subtypes with saccular and acute dissecting aneurysms having a female preponderance and atherosclerotic/fusiform aneurysms being more common in men. Saccular aneurysms seem stable over time and are likely to represent an underlying predisposition to aneurysm development given that more than half of these cases are associated with multiple intracranial aneurysms. Intervention should be considered in segmental ectasia and chronic dissecting aneurysms, which demonstrate an increase in size over time, especially in men with significant vascular risk factors because of the increased risk of SAH.

Sources of Funding

Dr Saliou has been funded by the French Society of Radiology for this research.

Disclosures

None.

References

1. Zhang J, Zhang R, Wu Z, Lv X, Liu B. Results of endovascular management for mid-basilar artery aneurysms. *Interv Neuroradiol*. 2010;16:249–254.
2. Pandey AS, Koebbe C, Rosenwasser RH, Veznedaroglu E. Endovascular coil embolization of ruptured and unruptured posterior circulation aneurysms: review of a 10-year experience. *Neurosurgery*. 2007;60:626–36; discussion 636–7.
3. Higa T, Ujiie H, Kato K, Kamiyama H, Hori T. Basilar artery trunk saccular aneurysms: morphological characteristics and management. *Neurosurg Rev*. 2009;32:181–91; discussion 191. doi: 10.1007/s10143-008-0163-3.
4. Schievink WI, Wijdicks EF, Piepgras DG, Chu CP, O'Fallon WM, Whisnant JP. The poor prognosis of ruptured intracranial aneurysms of the posterior circulation. *J Neurosurg*. 1995;82:791–795. doi: 10.3171/jns.1995.82.5.0791.
5. Kwon HM, Kim JH, Lim JS, Park JH, Lee SH, Lee YS. Basilar artery dolichoectasia is associated with paramedian pontine infarction. *Cerebrovasc Dis*. 2009;27:114–118. doi: 10.1159/000177917.
6. Drake CG, Peerless SJ. Giant fusiform intracranial aneurysms: review of 120 patients treated surgically from 1965 to 1992. *J Neurosurg*. 1997;87:141–162. doi: 10.3171/jns.1997.87.2.0141.
7. Chung J, Park H, Lim YC, Hyun DK, Shin YS. Endovascular treatment of basilar artery trunk aneurysms. *Acta Neurochir (Wien)*. 2011;153:2137–2145. doi: 10.1007/s00701-011-1117-z.
8. Nijensohn DE, Saez RJ, Reagan TJ. Clinical significance of basilar artery aneurysms. *Neurology*. 1974;24:301–305.
9. Raphaeli G, Collignon L, De Witte O, Lubicz B. Endovascular treatment of posterior circulation fusiform aneurysms: single-center experience in 31 patients. *Neurosurgery*. 2011;69:274–283. doi: 10.1227/NEU.0b013e31821723f2.
10. Mizutani T, Miki Y, Kojima H, Suzuki H. Proposed classification of nonatherosclerotic cerebral fusiform and dissecting aneurysms. *Neurosurgery*. 1999;45:253–259; discussion 259.
11. Krings T, Piske RL, Lasjaunias PL. Intracranial arterial aneurysm vasculopathies: targeting the outer vessel wall. *Neuroradiology*. 2005;47:931–937. doi: 10.1007/s00234-005-1438-9.
12. Krings T, Alvarez H, Reinacher P, Ozanne A, Baccin CE, Gandolfo C, et al. Growth and rupture mechanism of partially thrombosed aneurysms. *Interv Neuroradiol*. 2007;13:117–126.
13. Bland JM, Altman DG. Multiple significance tests: the Bonferroni method. *BMJ*. 1995;310:170.
14. Schievink WI. Intracranial aneurysms. *N Engl J Med*. 1997;336:28–40. doi: 10.1056/NEJM199701023360106.
15. Brisman JL, Song JK, Newell DW. Cerebral aneurysms. *N Engl J Med*. 2006;355:928–939. doi: 10.1056/NEJMra052760.
16. Bijlenga P, Ebeling C, Jaegersberg M, Summers P, Rogers A, Waterworth A, et al; @neurIST Investigators. Risk of rupture of small anterior communicating artery aneurysms is similar to posterior circulation aneurysms. *Stroke*. 2013;44:3018–3026. doi: 10.1161/STROKEAHA.113.001667.
17. Zhao WY, Krings T, Alvarez H, Ozanne A, Holmin S, Lasjaunias P. Management of spontaneous haemorrhagic intracranial vertebrobasilar dissection: review of 21 consecutive cases. *Acta Neurochir (Wien)*. 2007;149:585–96; discussion 596. doi: 10.1007/s00701-007-1161-x.
18. Gonzalez AM, Narata AP, Yilmaz H, Bijlenga P, Radovanovic I, Schaller K, et al. Blood blister-like aneurysms: single center experience and systematic literature review. *Eur J Radiol*. 2014;83:197–205. doi: 10.1016/j.ejrad.2013.09.017.
19. Matouk CC, Kaderali Z, terBrugge KG, Willinsky RA. Long-term clinical and imaging follow-up of complex intracranial aneurysms treated by endovascular parent vessel occlusion. *AJNR Am J Neuroradiol*. 2012;33:1991–1997. doi: 10.3174/ajnr.A3079.
20. Cruz JP, O'Kelly C, Kelly M, Wong JH, Alshaya W, Martin A, et al. Pipeline embolization device in aneurysmal subarachnoid hemorrhage. *AJNR Am J Neuroradiol*. 2013;34:271–276. doi: 10.3174/ajnr.A3380.
21. Nakatomi H, Segawa H, Kurata A, Shiokawa Y, Nagata K, Kamiyama H, et al. Clinicopathological study of intracranial fusiform and dolichoectatic aneurysms: insight on the mechanism of growth. *Stroke*. 2000;31:896–900.

American Heart Association | American Stroke Association
Stroke
