# Supplementary material for: Management strategies and outcomes of basilar trunk aneurysms: a systematic review and meta-analysis
Source: Langenbecks Arch Surg. 2026 Jan 20;411(1):71. doi: 10.1007/s00423-025-03959-3 (PMC12852281; doi:10.1007/s00423-025-03959-3)
Supplement: Supplementary file 1 — Supplementary file1 (ZIP 55327 KB) [file 423_2025_3959_MOESM1_ESM.zip › literature.zip!/meta/Seifert 2001.pdf]

Acta Neurochir (Wien) (2001) 143: 343–349

Acta Neurochirurgica
© Springer-Verlag 2001
Printed in Austria

Management-Related Morbidity and Mortality in Unselected Aneurysms of the Basilar Trunk and Vertebrobasilar Junction

V. Seifert[1], **A. Raabe**[1], and **D. Stolke**[2]

[1] Department of Neurosurgery, Johann Wolfgang Goethe University, Frankfurt/Main, Germany
[2] Department of Neurosurgery, University of Essen, Germany

Summary

Objects. To analyze the management-related morbidity and mortality in unselected aneurysms of the basilar trunk and vertebrobasilar junction. The secondary objective was to investigate the factors associated with favourable or unfavourable surgical outcome in order to define subgroups for surgical and endovascular treatment.

Methods. 24 consecutive patients with aneurysms of the basilar trunk and vertebrobasilar junction were included in this study. They comprised 2.7% of all aneurysms treated during the study period between 1990 and 1997. 22 patients presented with acute subarachnoid hemorrhage (SAH) and 2 patients with symptoms of brainstem compresssion. All patients were managed using a standard protocol including surgery at the earliest possible moment, aggressive tripe-H therapy in patients with symptomatic vasospasm and mandatory follow-up angiography. 23 patients underwent surgical clipping and one patient endovascular coiling of the aneurysm. 12 patients had an excellent outcome. 6 patients had a good outcome, resulting in a total of satisfactory outcomes in 18 patients (75%). 4 patients (17%) had moderate to severe deficits. Two patients died (8%). Both patients had fusiform basilar trunk aneurysms. Good or excellent outcome was observed in 7 of 8 patients with aneurysms of the vertebrobasilar junction, 13 of 14 patients with moderate or minor SAH or without SAH (Fisher grade 0 to 2) and all patients with small sized aneurysm (n = 6). Factors mostly associated with poor outcome or death after surgical treatment were aneurysm location at the basilar trunk, large aneurysm size or fusiforme aneurysm type and severe SAH.

Conclusions. Location, aneurysm size and the severity of SAH may help to predict the subgroup which highly benefits from surgical clipping of these rare vascular lesions.

Keywords: Aneurysm; basilar trunk; vertebrobasilar junction; clipping; outcome.

Introduction

Aneurysms of the basilar trunk and vertebrobasilar junction are rare. In the two largest published single institution aneurysm series they comprise only 1.8 percent of all intracranial aneurysms, or 15% to 17% of those aneurysms located along the posterior circulation [7, 28]. It is obvious, that the number of aneurysms located in this region seen by neurosurgeons is to a large extent dependent on the referral pattern of a highly specialized neurovascular institution. Consequently, many centers, even with high neurovascular expertise will only see a limited number of these treacherous aneurysms per year. Although many publications have appeared dealing with surgical aspects and problems of basilar trunk and vertebrobasilar junction aneurysms, to the best of our knowledge no article has detailed the outcome results of a consecutive unselected series of these aneurysms using a standardized treatment protocol.

Clinical Material and Methods

Patient Population

Between January 1990 and December 1997 24 consecutive patients with aneurysms of the basilar trunk or vertebrobasilar junction (VB-Junction) were treated. A subgroup of these patients has already been presented elsewhere [21]. During this period 884 patients with aneurysmal subarachnoid hemorrhage were admitted to our institution. A total of 104 aneurysms, which represented 11.8% of all aneurysms in our series, were located along the posterior circulation. Thus, the 24 basilar trunk and VB-junction aneurysms comprise 23% of all posterior circulation aneurysms and 2.7% of all aneurysms seen during this period. The patient group consisted of 14 male and 10 female patients with an age distribution ranging from 19 to 68 years (mean 49 years). Patients with SAH were graded according to the HUNT and HESS scale. The amount of subarachnoid blood seen on the initial CT-scan was graded according to Fisher's classification. According to the definition of SUGITA, aneurysms of the basilar trunk were defined as those lesions originating on the portion of the basilar artery between the junction of the vertebral arteries and the branching point of the superior cerebellar artery [26]. A lesion was defined as a VB-junction aneurysm when its origin was either directly located at the VB junction, or the VB-junction was incorporated into

Table 1. *Characteristics of the 24 Patients with Aneurysms of the Basilar Trunk or the Vertebrobasilar Junction*

No.	Pat	Sex	Age	Aneurysm location	Size	H + H grade	Fisher grade	Surgical approach	Outcome
1.	D. D.	f	55	basilar trunk	large	III	III	pterional/transzygomatic	poor
2.	M. S.	f	47	basilar trunk	small	I	II	comb. transpetrosal	excellent
3.	I. W.	f	64	basilar trunk	large	II	III	fronto-orbito-zygomatic	good
4.	D. D.	m	40	basilar trunk	large	II	II	comb. transpetrosal	excellent
5.	F. D.	m	57	VB-junction	small	II	I	far lateral	excellent
6.	J. R.	m	42	VB-junction	giant	[1]	0	far lateral	good
7.	D. J.	m	42	basilar trunk	fusiforme	IV	III	comb. transpetrosal	dead
8.	V. V.	f	19	basilar trunk	small	III	II	comb. transpetrosal	excellent
9.	W. S.	m	66	basilar trunk	large	II	III	comb. transpetrosal	poor
10.	P. W.	f	46	VB-junction	large	II[2]	II	comb. transpetrosal	excellent
11.	H. B.	m	56	basilar trunk	giant	[3]	0	comb. transpetrosal	excellent
12.	A. P.	m	39	VB-junction	large	I	II	far lateral	excellent
13.	A. T.	m	68	VB-junction	large	III	III	far lateral	poor
14.	S. V.	m	30	basilar trunk	large	II	II	comb. transpetrosal	poor
15.	W. P.	m	54	basilar trunk	fusiforme	IV	III	ventriculostomy	dead
16.	K. L.	m	42	VB-junction	small	II	I	comb. transpetrosal	excellent
17.	X. T.	f	25	basilar trunk	large	III	II	comb. transpetrosal	good
18.	T. K.	m	47	VB-junction	small	I	II	far lateral	excellent
19.	D. F.	f	44	basilar trunk	large	III	III	comb. transpetrosal	good
20.	G. M.	f	45	basilar trunk	small	III	II	comb. transpetrosal	excellent
21.	M. P.	m	64	VB-junction	large	II	III	far lateral	good
22.	S. G.	f	49	basilar trunk	large	I	I	comb. transpetrosal	good
23.	F. X.	m	21	basilar trunk	large	IV	III	GDC	excellent
24.	F. D.	f	41	basilar trunk	fusiforme	II	III	comb. transpetrosal	excellent

[1] Giant completetly thrombosed and partially calcified aneurysm of the VB-junction, with clinical signs of brain stem compression and lower cranial nerve dysfunction.
[2] Asymptomatic VB-junction aneurysm diagnosed incidentally during panangiography after SAH from an right-sided internal carotid artery aneurysm.
[3] Giant partially thrombosed aneurysm of the midbasilar trunk, presenting as an intrapontine mass.

the aneurysm. Aneurysms were classified as small (<1 cm), large (1–2.5 cm), giant (>2.5 cm) and fusiform. Information on all surviving patients was collected by either direct examination during follow-up, or telephone information from their treating physicians at a minimum interval of 12 months post-treatment. All patients were graded according to the following grading scale: patients had an excellent outcome when they were able to return to their premorbid activities and full-employment. Patients were graded as having a good outcome, when they had minor neurological deficits, mainly from cranial nerve palsy but were capable of returning to the majority of their daily activities. Patients with moderate or severe neurological deficits that made them partially or completely dependent on others without the possibility of returning to their pretreatment activities were graded as poor result.

Treatment

In 22 patients SAH was confirmed by computerized tomography. A ventriculostomy with temporary ventricular drainage was performed in those patients presenting with posthemorrhagic hydrocephalus. In all patients with SAH the presence of an aneurysm was confirmed by digital subtraction angiography. In selected patients an additional 3D-CT-angiography was performed to obtain additional information. Nimodipine was used routinely in all patients. All patients were kept normotensive and euvolaemic before surgery or endovascular treatment. After aneurysm clipping or coil placement moderate prophylactic hypertensive hypervolaemic haemodilution was used. Clinically symptomatic vasospasm was treated utilizing aggressive therapeutic hypertensive hypervolaemic haemodilution. Surgery was performed as early as possible taking into consideration that no patient presented in clinical grade V according to Hunt and Hess. In general, surgery was performed under normotension and normothermia. During the later part of this series, patients in whom intra-operative temporary clipping of parent vessels was anticipated, surgery was performed under moderate hypothermia (33 °C).

In those cases with direct aneurysm clipping, surgery was performed by the senior author (VS) by using one of the three following surgical approaches. In those patients with high positioned aneurysms of the basilar trunk an orbitozygomatic craniotomy with a pterional approach was used. For aneurysms of the middle and lower basilar trunk and in selected aneurysms of the V-B-junction, a combined supra-infratententorial transpetrosal approach was made. Surgery in this group was performed routinely in the semisitting or lounging position. In all of these patients surgery was performed under ventricular drainage. Parts of these cases have already been presented previously. The majority of V-B-junction aneurysms were approached using a far lateral paracondylar approach, however as already mentioned, selected cases of these aneurysms were treated using the transpetrosal approach. Temporary vessel occlusion was applied according to the intra-operative situation. Maximum time of temporary clipping of the parent artery was usually limited to 5

Table 2. *Ruptured Aneurysms of the Basilar Trunk and the Vertebrobasilar Junction. Location and Size at Presentation of 24 Patients*

Location	Small (<1 cm)	Large (1–2.5 cm)	Giant (>2.5 cm)	Fusiform
Basilar trunk	3	9	1	3
Vertebrobasilar junction	3	4	1	0
Total (n = 24)	6	13	2	3

minutes. Endovascular treatment was used in one patient presenting in poor medical condition with a large basilar trunk aneurysm. Postoperative control-angiography was scheduled routinely to confirm complete occlusion of the aneurysm.

Results

Clinical Presentation

Of the 22 patients presenting with clinical signs of SAH at the time of admission, 13 were in grade I or II, 6 were in grade III and 3 were in grade IV according to Hunt and Hess. The time from subarachnoid hemorrhage to clinical admission was up to 72 hours in 15 patients and longer than that period up to 28 days in the remaining patients. The amount of blood distribution in the basal cisterns according to Fisher's classification was grade 1 in 3 patients, grade 2 in 9 patients and grade 3 in 10 patients. In none of the patients relevant additional intraparenchymal hemorrhage was present. On admission, 8 patients had accompanying posthemorrhagic hydrocephalus which led to immediate placement of a ventricular drainage. However, ventricular catheterization was employed deliberately during the peri-operative period in an additional 10 patients to allow for intra-operative release of CSF. During the postoperative follow-up period only 4 patients required placement of a permanent ventriculoperitoneal shunt system.

Table 3. *Fisher Grading in Patients with SAH from Ruptured Aneurysms of the Basilar Trunk and the Vertebrobasilar Junction*

Location	Grade 0	Grade 1	Grade 2	Grade 3
Basilar trunk	1	1	6	8
Vertebrobasilar junction	1	2	3	2
Total (n = 24)	2	3	9	10

Aneurysm Treatment

With regard to type and size of aneurysm, there were 21 saccular aneurysms of which 6 were small, 13 were large and 2 were giant. All three fusiforme aneurysms were large. 16 aneurysms were located along the basilar trunk as defined earlier, and 8 aneurysms were located at the VB-junction.

Two patients with giant aneurysms of the basilar trunk and VB-junction respectively presented with progressive clinical signs of brain stem compression and cranial nerve dysfunction. One asymptomatic aneurysm of the VB-junction was diagnosed during panangiography for subarachnoid hemorrhage resulting from a carotid artery aneurysm.

Of the aneurysms of the basilar trunk, one was clipped using a pterional approach with section of the zygoma and in another patient a combined orbitozygomatic approach was used. 12 aneurysms of the basilar trunk including one giant aneurysm and 2 aneurysms of the V-B-junction, including one calcified giant aneurysm were treated using a combined supra-infratententorial transpetrosal approach. 6 patients with V-B-junction aneurysms were approached using a far lateral paracondylar approach. Again 4 patients were treated in the semisitting position. In 2 patients surgery was performed in the three-quarter prone position. One patient was treated by endovascular coil embolization, and one patient with a fusiform aneurysm of the basilar trunk was treated with ventriculostomy alone. Postoperative angiography was performed in 22 patients at an intervall of 6 days to 5 weeks after definitive aneurysm treatment. Complete aneurysm occlusion was confirmed in 20 (91%) patients. In one patient with a fusiform aneurysm incorporating a part of the VB-junction a small remnant of the aneurysm was not incorporated in the clip. The patient could be convinced to undergo a control angiogram 3 years after surgery, which demonstrated the aneurysm rest to be unchanged. In the patient with the giant calcified VB-junction aneurysm, in

Table 4. *Ruptured Aneurysms of the Basilar Trunk and the Vertebrobasilar Junction. Location and Outcome of the 24 Patients*

Location	Excellent outcome	Good outcome	Poor outcome	Death
Basilar trunk	7	4	3	2
Vertebrobasilar junction	5	2	1	0
Total (n = 24)	12	6	4	2

whom the tumor-like aneurysm sac had been resected during surgery, control MRI at 3, 12 and 28 months postsurgery showed adequate decompression of the previously compressed brain stem. The only patient with coil embolization showed complete occlusion of the aneurysm at postoperative angiography.

Outcome

The outcome of all 24 patients at twelve months according to the above described grading scale was as follows: 12 patients had an excellent outcome. 6 patients had a good outcome, resulting in a total of satisfactory outcomes in 18 patients (75%). 4 patients (17%) had moderate to severe deficits. Two patients died (8%). Both patients had fusiform basilar trunk aneurysms.

The first patient had been admitted in grade IV HUNT and HESS. Angiography showed a fusiform aneurysm pointing to the left side between the AICA and the VB-junction. Within two days the patient had two recurrent hemorrhages with consecutive clinical deterioration. A ventricular drain was placed, and it was decided to delay definitive surgery. However, on the next day, there was again fresh blood in the drainage system, evidence of a third recurrent bleed. Due to his young age it was now decided to proceed to aneurysm clipping in order to prevent further hemorrhages. After transpetrosal exposure, the highly fragile fusiform aneurysm ruptured during an attempt of clip positioning. Despite deep hypotension, the leaking part of the aneurysm could not be secured satisfactorily, and finally a clip had to be place over the rupture point incorporating parts of the basilar artery. Although the patient woke up and started obeying to commands, he deteriorated two days later and died from progressive brain stem infarction. The second patient was admitted in grade IV after a severe SAH. A ventricular drain was placed. Angiography revealed a broad based fusiform midbasilar aneurysm incorporating the AICA origin on both sides. Within four days he suffered two catastrophic repeated hemorrhages from which he finally died.

Discussion

The first case of a successfully surgically treated basilar trunk aneurysm was operated on by DRAKE in 1959 and presented in 1995 among his series of 1767 aneurysms of the basilar and vertebral artery collected from his London, Ontario experience. Among this outstanding collection of vertebrobasilar aneurysms, 14.7% were located along the basilar trunk and VB-junction. It is obvious, that such a series will never be collected again and that, especially in the case of basilar trunk and VB-junction aneurysms, it reflects the experience of a highly specialized neurovascular center with world wide patient referral. Of this patient group, 76% had an excellent or good outcome, 11% had a poor outcome and 13% were dead. These numbers are even more impressive when it is taken into consideration, that 37.6% of the basilar trunk and VB-junction aneurysms were giant in size. Among Yasargil's series of 144 vertebrobasilar aneurysms, presented in 1984, 25 (17.4%) were located along the basilar trunk and VB-junction of which 7 (28%) were giant in size [28]. 5 patients (20%) had a poor outcome or died and 80% made a good or an excellent recovery.

With regard to the neurosurgical treatment of basilar trunk and VB-junction aneurysms all of the recently published papers on this exciting subgroup of aneurysms have exclusively dealt with surgery related problems, such as operative approaches, technical adjuncts such as intra-operative temporary vessel occlusion or deep hypothermia. Although these technical aspects of the surgical treatment are extremely important for the neurosurgeon being confronted with aneurysms in this location, the management related morbidity and mortality in an unselected series of basilar trunk and VB-junction aneurysms have not been presented so far.

Surgical Treatment

Aneurysms of the basilar trunk and the vertebrobasilar junction were historically classified among the most challenging vascular lesions for surgical treatment. The limited exposure of the highly vulnerable structures such as the aneurysm, the brain stem and the numerous perforators arising from the parent artery obtained by the classical lateral suboccipital or subtemporal approach often resulted in a high rate of surgical complications. In recent years a variety of different approaches were developed to increase exposure of this region, which are often referred to as skull base techniques [1, 2, 6, 8, 9, 11, 12, 16, 17, 20, 22–24]. Although many of these techniques may be used for these lesions, we preferred for the majority of our cases the combined supra-infratentorial transpetrosal retro-

labyrinthine approach for more distal lesions or the far lateral approach for more proximal lesions. The combined supra-infratentorial presigmoid transpetrosal approach was developed from a variety of neurosurgical and otological approaches. It was mainly introduced in the 70s and further refined in the following years [1, 5, 9–11, 14, 15, 20]. Our technique of posterior retrolabyrinthine transpetrosal approach has been described elsewhere [21]. The far lateral approach has gradually evolved over many years focussing on removal of the lateral foramen magnum to the occipital condyle by Heros [12], removal of the posterior condyle and the lateral mass of C1 by Spetzler [25] and removal of the jugular tubercle by Seeger [3]. These techniques offer many advantages and, if necessary, may be combined to further increase exposure. In two patients with high aneurysms of the distal basilar trunk an orbitozygomatic pterional approach was used.

Endovascular Treatment

With the advent of improved technology patients are increasingly scheduled for primary endovascular treatment. Endovascular techniques for occluding basilar trunk or vertebrobasilar aneurysms consist mainly in Guglielmi detachable coils (GDC) placement or rarely balloon occlusion of a defined segment of the parent artery. Although there are case reports about the successful occlusion of these aneurysms, we are not aware of a study detailing the results of this treatment in a series of consecutive patients. Therefore and because of the obvious missing long term results using the improved GDC technique, there is currently no evidence that in patients with low surgical risk endovascular treatment may achieve similar results as surgical clipping. However, in patients with high surgical risks such as large aneurysms in this location and poor medical condition, endovascular treatment may currently offer useful treatment option. Although GDC treatment appeared to be more successful in small saccular aneurysms (complete occlusion rate 58%) compared to large or giant aneurysms (complete occlusion rate 35% and 50% respectively), or small neck aneurysms (complete occlusion rate 71%) compared to wide neck aneurysms (complete occlusion rate 31%), the use of new techniques such as balloon-assisted coiling or stent protection of the parent artery may increase the rate of successful treatment of these problematic vascular lesions [13, 18, 19, 27]. Unfortunately, the unique anatomical and functional vascular situation of the basilar trunk may limit some of the techniques otherwise useful during endovascular treatment such as the placement of stents or balloons. In the series of 6 patients with aneurysms of the vertebrobasilar junction published by Collice *et al.* four were scheduled for endovascular treatment with GDC [4]. However, in three patients correct obliteration could not be achieved and consequently these patients underwent surgery. Hence, for many of the most challenging aneurysms at present surgical treatment may still remain the only option to improve morbitidy and mortality resulting from the natural history of these lesions.

Data Analysis

Although the numbers are insufficient to provide statistical analysis of the data, there were differences between aneurysms of the basilar trunk and the vertebrobasilar junction in our series. Aneurysms of the basilar trunk were twice as common as those arising from the vertebrobasilar junction. However, they were associated with 100% (2/2) of all deaths, 75% (3/4) of all poor outcomes and accordingly 83% (5/6) of all unfavorable outcomes in our cohort of 24 consecutive patients. Moreover, of the 6 patients with unfavorable outcome (poor outcome or death) all patients had either a fusiform or a large aneurysm and 5/6 patients had massive SAH classified as Fisher grade 3. Thus, factors mostly associated with poor outcome or death after surgical treatment were aneurysm location on the basilar trunk, large aneurysm size or fusiform aneurysm type and severe SAH Fisher grade 3. On the other hand good or excellent outcome was observed in 7/8 patients with aneurysms of the vertebrobasilar junction, 13/14 patients with moderate or minor SAH or without SAH (Fisher grade 0 to 2) and all patients with a small size of the aneurysm (6/6).

Consequently, endovascular treatment should be considered as the first choice in patients presenting with massive SAH and large basilar trunk aneurysms and those fusiform aneurysms amenable to endovascular treatment.

The bottom line of our data however is that modern surgical treatment of small aneurysms of the basilar trunk or the vertebrobasilar junction in patients with mild or moderate SAH is highly successful despite the critical location.

Conclusion

Aneurysms arising from the basilar trunk or the vertebrobasilar junction require interdisciplinary treatment. In patients presenting with minor or moderate SAH and small aneurysms, regardless of the location, our first choice would be surgical clipping of the aneurysm. In these patients surgical treatment is successful as demonstrated by the high numbers of good or excellent outcome (93% in our series) and the low rates of surgical morbidity and mortality when using modern skull base techniques, which are essential for achieving these results. Endovascular treatment should be considered as the first choice in patients presenting with massive SAH and large basilar trunk aneurysms and those fusiform aneurysms amenable to endovascular treatment.

References

1. Al-Mefty O, Fox JL, Smith RR (1988) Petrosal approach for petroclival meningiomas. Neurosurgery 22: 510–517
2. Archer DJ, Young S, Uttley D (1987) Basilar aneurysms: a new transclival approach via maxillotomy. J Neurosurg 67: 54–58
3. Bertalanffy H, Seeger W (1991) The dorsolateral, suboccipital, transcondylar approach to the lower clivus and anterior portion of the craniocervical junction. Neurosurgery 29: 815–821
4. Collice M, Arena O, D'Aliberti G *et al* (1997) Aneurysms of the vertebro-basilar junction area: preliminary experience in endovascular and surgical management. Acta Neurochir (Wien) 139: 124–133
5. Day JD, Fukushima T, Giannotta SL (1997) Cranial base approaches to posterior circulation aneurysms. J Neurosurg 87: 544–554
6. Dolenc VV, Skrap M, Sustersic J *et al* (1987) A transcavernous-transsellar approach to the basilar tip aneurysms. Br J Neurosurg 1: 251–259
7. Drake CG, Peerless SJ, Hernesniemi JA (1996) Surgery of vertebrobasilar aneurysms, London, Ontario experience on 1767 patients. Springer, Wien New York
8. Giannotta SL, Maceri DR (1988) Retrolabyrinthine transsigmoid approach to basilar trunk and vertebrobasilar artery junction aneurysms. Technical note. J Neurosurg 69: 461–466
9. Hakuba A, Nishimura S, Inoue Y (1985) Transpetrosal-transtentorial approach and its application in the therapy of retrochiasmatic craniopharyngiomas. Surg Neurol 24: 405–415
10. Hakuba A, Nishimura S, Tanaka K *et al* (1977) Clivus meningioma: six cases of total removal. Neurol Med Chir (Tokyo) 17: 63–77
11. Hashi K, Nin K, Shimotake K (1982) Transpetrosal combined supra-infratentorial approach for midline vertebro-basilar aneurysms. Mod Neurosurg 1: 442–448
12. Heros RC (1986) Lateral suboccipital approach for vertebral and vertebrobasilar artery lesions. J Neurosurg 64: 559–562
13. Higashida RT, Smith W, Gress D *et al* (1997) Intravascular stent and endovascular coil placement for a ruptured fusiform aneurysm of the basilar artery. Case report and review of the literature. J Neurosurg 87: 944–949
14. House WF, De la Cruz A, Hitselberger WE (1978) Surgery of the skull base: transcochlear approach to the petrous apex and clivus. Otolaryngology 86: ORL-9
15. House WF, Hitselberger WE (1976) The transcochlear approach to the skull base. Arch Otolaryngol 102: 334–342
16. Kasdon DL, Stein BM (1979) Combined supratentorial and infratentorial exposure for low-lying basilar aneurysms. Neurosurgery 4: 422–426
17. Kawase T, Toya S, Shiobara R *et al* (1985) Transpetrosal approach for aneurysms of the lower basilar artery. J Neurosurg 63: 857–861
18. Moret J, Cognard C, Weill A *et al* (1997) [Reconstruction technic in the treatment of wide-neck intracranial aneurysms. Long-term angiographic and clinical results. Apropos of 56 cases]. J Neuroradiol 24: 30–44
19. Nelson PK (1998) Neurointerventional management of intracranial aneurysms. Neurosurg Clin N Am 9: 879–895
20. Samii M, Ammirati M (1988) The combined supra-infratentorial pre-sigmoid sinus avenue to the petro-clival region. Surgical technique and clinical applications. Acta Neurochir (Wien) 95: 6–12
21. Seifert V, Stolke D (1996) Posterior transpetrosal approach to aneurysms of the basilar trunk and vertebrobasilar junction [see comments]. J Neurosurg 85: 373–379
22. Sen CN, Sekhar LN (1990) An extreme lateral approach to intradural lesions of the cervical spine and foramen magnum [see comments]. Neurosurgery 27: 197–204
23. Solomon RA, Stein BM (1988) Surgical approaches to aneurysms of the vertebral and basilar arteries. Neurosurgery 23: 203–208
24. Spetzler RF, Daspit CP, Pappas CT (1992) The combined supra- and infratentorial approach for lesions of the petrous and clival regions: experience with 46 cases. J Neurosurg 76: 588–599
25. Spetzler RF, Grahm TW (1990) The far-lateral approach to the inferior clivus and the upper cervical region: technical note. BNI Quarterly 6: 35–38
26. Sugita K, Kobayashi S, Takemae T *et al* (1987) Aneurysms of the basilar artery trunk. J Neurosurg 66: 500–505
27. Vinuela F, Duckwiler G, Mawad M (1997) Guglielmi detachable coil embolization of acute intracranial aneurysm: perioperative anatomical and clinical outcome in 403 patients. J Neurosurg 86: 475–482
28. Yasargil MG (1984) Microneurosurgery vol 2. Clinical considerations, surgery of the intracranial aneurysms and results. Thieme, Stuttgart

Comments

This article describes a single center's experience treating a small series of basilar trunk and vertebrobasilar junction aneurysms. However, the authors have achieved results that are comparable with those of Drake [1], which is unquestionably the gold standard. It is worth noting that in Drake's formidable series, over a third of the aneurysms were classified as being giant.

There are multiple issues in the management of these complex aneurysms. First is the selection of patients. This is primarily influenced by the age, underlying medical condition, and the clinical grade of the patient at presentation after subarachnoid hemorrhage [2]. The second issue is the surgical access. The definitive paper by Day *et al.* summarizes the skull base approaches to these aneurysms according to their location [3]. Seifert *et al.* discuss the three most widely used approaches. The third issue is the choice of treatment. The development of endovascular treatment and the exponential

improvement of the technique and the technicians performing these procedures have opened the way for the alternative treatment of basilar aneurysms. Indeed, Lempert *et al.* [4], using endovascular treatment of posterior circulation aneurysms, achieved 2.8% overall mortality and 83% good/excellent outcomes, comparing favorably with the published surgical results. Understandably, this technique carries, in addition to comparable complications of vasospasm and rebleeding, the inherent risks of effects specific to intravascular coiling, namely, thrombosis, coil unraveling, encroachment of the parent artery, and vascular rupture. In our experience, of 20 patients who have undergone surgical clipping of basilar apex aneurysms, 15 (75%) had a good outcome, defined as a Glasgow outcome score of 4 or 5. Of 21 patients who have undergone endovascular treatment with Guglielmi detachable coils, 90% experienced a good outcome [5]. Thus, our results are better with coiling than with clipping. However, it should be noted that these results represent our experience with basilar apex aneurysms, in contrast to the basilar trunk and vertebrobasilar junction aneurysms treated and discussed by Seifert *et al.*

Giant wide neck basilar trunk aneurysms continue to be a challenge for surgical treatment, and are rarely approachable by current intravascular techniques. Patients with complex aneurysms should be treated in centers with the necessary surgical and endovascular experience to offer the most acceptable outcome.

References

1. Drake CG, Peerless SJ, Hernesniemi J (1996) Surgery of vertebral basilar aneurysms. Springer, Berlin Heidelberg New York Tokyo
2. Gruber DP, Zimmerman GA, Tomsick TA, van Loveren HR, Link MJ, Tew JM Jr (1999) A comparison between endovascular and surgical management of basilar apex aneurysms. J Neurosurg 90: 868–874
3. Day JD, Fukushima T, Giannotta SL (1997) Cranial base approaches to posterior circulation aneurysms. J Neurosurg 87: 544–554
4. Lempert *et al* (2000) Endovascular treatment of ruptured posterior circulation cerebral aneurysms. Stroke 31: 100–110
5. Gruber DP, Zimmerman GA, Tomsick TA, van Loveren HR, Link MJ, Tew JM Jr (1999) A comparison between endovascular and surgical management of basilar artery apex aneurysms. J Neurosurg 90: 868–874

Ali Nadn – Sepahi – J. Tew

Seifert *et al.* in this well-written report present their retrospective experience in the management of 24 consecutive basilar trunk and vertebrobasilar junction aneurysms. The development of microsurgical techniques combined with thorough understanding of perforator anatomy have led to good results in these otherwise formidable lesions. More recent advancements in the use of skull base approaches have also led to improved access to posterior circulation aneurysms. For basilar trunk and vertebrobasilar junction aneurysms, the petrosal and the transcondylar avenues may allow in selected situations additional manoeuvrability. Of significant importance in this region is possible injury to the ninth and tenth cranial nerves leading to disabling morbidity. In selected cases, endovascular coiling of these aneurysms ought to be considered.

We commend the authors for their excellent results in the management of these complex and often formidable aneurysms.

M. G. Yasargil – S. T. Abdulrauf

Correspondence: Volker Seifert M.D., Ph.D., Department of Neurosurgery, Johann Wolfgang Goethe University, Frankfurt/Main, Schleusenweg 2-16, 60528 Frankfurt am Main, Germany.

Copyright of Acta Neurochirurgica is the property of Kluwer Academic Publishing / Academic and its content may not be copied or emailed to multiple sites or posted to a listserv without the copyright holder's express written permission. However, users may print, download, or email articles for individual use.
