# Supplementary material for: Management strategies and outcomes of basilar trunk aneurysms: a systematic review and meta-analysis
Source: Langenbecks Arch Surg. 2026 Jan 20;411(1):71. doi: 10.1007/s00423-025-03959-3 (PMC12852281; doi:10.1007/s00423-025-03959-3)
Supplement: Supplementary file 1 — Supplementary file1 (ZIP 55327 KB) [file 423_2025_3959_MOESM1_ESM.zip › literature.zip!/meta/Sim 2022.pdf]

J NeuroIntervent Surg: first published as 10.1136/neurintsurg-2021-017698 on 27 July 2021. Downloaded from http://jnis.bmj.com/ on September 11, 2021 at lane medical library. Protected by copyright.

Case series

Basilar artery trunk aneurysm: clinical and angiographic outcomes of endovascular treatment

Sook Young Sim,[1] Joonho Chung,[2] Jai Ho Choi,[3] Myeong Jin Kim,[4] Yong Sam Shin,[3] Yong Cheol Lim[5]

[1]Department of Neurosurgery, Inje University Ilsan Paik Hospital, Goyang, Korea (the Republic of)
[2]Department of Neurosurgery, Yonsei University College of Medicine, Seodaemun-gu, Korea (the Republic of)
[3]Department of Neurosurgery, Catholic University of Korea School of Medicine, Seoul, Seoul, Korea (the Republic of)
[4]Department of Neurosurgery, Gachon University Gil Medical Center, Incheon, Incheon, Korea (the Republic of)
[5]Department of Neurosurgery, Ajou University School of Medicine and Graduate School of Medicine, Suwon, Gyeonggi-do, Korea (the Republic of)

Correspondence to
Dr Yong Cheol Lim, Department of Neurosurgery, Ajou University School of Medicine and Graduate School of Medicine, Suwon, Gyeonggi-do 443-721, Korea (the Republic of); nsyclim@gmail.com and Dr Joonho Chung, Department of Neurosurgery, Severance Hospital, Yonsei University College of Medicine, Seoul, Republic of Korea; ns.joonho.chung@gmail.com

JC and YCL contributed equally.

Received 26 April 2021
Accepted 22 June 2021

Check for updates

© Author(s) (or their employer(s)) 2021. No commercial re-use. See rights and permissions. Published by BMJ.

To cite: Sim SY, Chung J, Choi JH, *et al*. *J NeuroIntervent Surg* Epub ahead of print: [*please include* Day Month Year]. doi:10.1136/neurintsurg-2021-017698

ABSTRACT

Background Basilar artery (BA) trunk aneurysms are rare, and the clinical characteristics and outcomes of endovascular treatment (EVT) remain unclear. The purpose of this study was to report clinical and angiographic outcomes of BA trunk aneurysm treated with EVT and to analyze risk factors for unfavorable outcomes.

Methods From October 2004 to December 2020, a total of 40 patients with BA trunk aneurysms underwent EVT. Clinical characteristics and outcomes were evaluated retrospectively from a prospectively collected database. Of the 40 enrolled patients, nine were treated by coiling without stents, 17 were treated by stent-assisted coiling, six by stent only, five by flow diverters, and three by vertebral artery occlusion.

Results In total, 27 (67.5%) patients had subarachnoid hemorrhage as an initial presentation, and 20 (50.0%) had large/giant aneurysms. Procedure-related complications occurred in five patients (12.5%); favorable clinical outcome was achieved in 27 patients (67.5%); and six patients (15.0%) died. Favorable angiographic outcome was achieved in 26 (83.9%) of 31 patients who underwent follow-up angiography. Poor initial Hunt-and-Hess grade (OR 7.67, 95% CI 1.55 to 37.80; p=0.018) was the only independent risk factor for unfavorable clinical outcome. Large/giant aneurysm (OR 8.14, 95% CI 1.88 to 27.46; p=0.047) and long lesion (OR 14.25, 95% CI 1.48 to 69.80; p=0.013) were independent risk factors for unfavorable angiographic outcomes during follow-up.

Conclusions EVT might be a feasible option for this rare disease entity. Unfavorable angiographic outcome might be expected in a large/giant aneurysm or a long lesion. It can be difficult to treat BA trunk aneurysms by EVT, needing multiple procedures or various techniques due to diverse clinical and angiographic features.

INTRODUCTION

Basilar artery (BA) trunk aneurysms are rare, representing only 1% of all intracranial aneurysms.[1–3] Large (10–24 mm) and giant (≥25 mm) BA trunk aneurysms that show a mass effect on the brain stem and cranial nerves have a tendency to progressively increase in size, with a high likelihood of rupture or even fatality.[1 3–6] Another characteristic of BA trunk aneurysm is that fusiform and dissecting aneurysms are frequently present in addition to saccular aneurysms. However, because they are so rare, the natural history and clinical characteristics of BA trunk aneurysms are not well clarified.

With the remarkable progress of endovascular treatment (EVT), it is now the first-line treatment for posterior circulation aneurysm; nevertheless, it can be difficult to apply to BA trunk aneurysms. Among various endovascular techniques, optimal EVT for patients harboring a BA trunk aneurysm is controversial. Additionally, no risk factor analysis for unfavorable clinical or angiographic outcome has been conducted in a large series of patients. Therefore, the purpose of this study was to report clinical and angiographic outcomes of BA trunk aneurysm treated with EVT and to analyze risk factors for unfavorable outcomes.

METHODS

Patient selection and characteristics

This retrospective study was approved by our institutional review board and informed consent was waived. Between October 2004 and December 2020, we had a prospectively collected database containing a series of 4187 aneurysms treated using EVT. Among them, a total of 40 consecutive patients (male/female ratio 19:21, mean age 50.5 years, range 13–82 years) with BA trunk aneurysms treated with EVT were included in this study. BA trunk aneurysm was defined as an aneurysm that arose between the vertebrobasilar junction and the origin of the superior cerebellar artery (figure 1). Presentations at admission were subarachnoid hemorrhage (27 patients, 67.5%), mass effect (seven patients, 17.5%), and incidental finding (six patients, 15%). In ruptured cases (n=27), initial Hunt and Hess grade (HHG) was 2 in 10 patients, 3 in seven, 4 in nine, and 5 in one. Fisher grade (FG) was 1 in one, 2 in four, 3 in 16, and 4 in six patients. Preoperative pontine hemorrhage, pontine infarction, and hydrocephalus were noticed in one (2.5%), two (5.0%), and 13 (32.5%) patients, respectively. One patient had preoperative rebleeding. Vascular risk factors including hypertension, diabetes mellitus, history of smoking, and dyslipidemia were observed in 16 (40.0%), six (15.0%), eight (20.0%), and seven (17.5%) patients, respectively (table 1). Clinical poor-grade patients were defined as those with an initial HHG of 4 or 5. Acute hydrocephalus was diagnosed on CT scans.

Aneurysm characteristics

Morphological aneurysm characteristics were determined using digital subtraction angiography with three-dimensional rotational imaging. Aneurysm size was measured as the maximal diameter

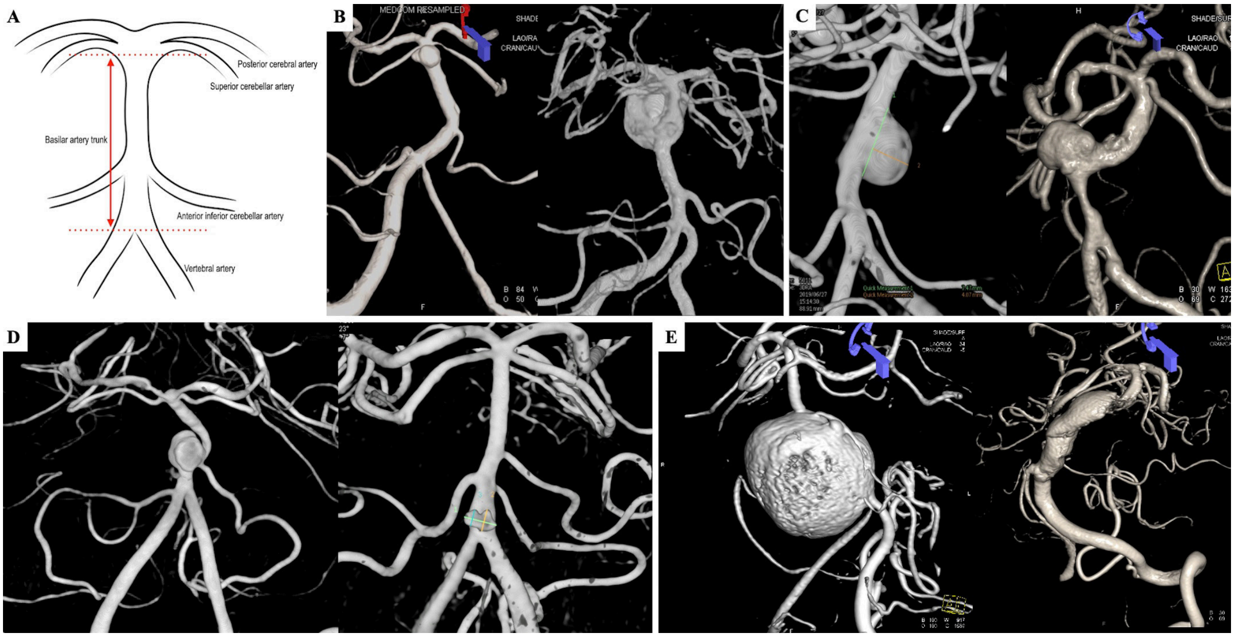

Figure 1 Basilar artery (BA) trunk aneurysms. (A) BA trunk aneurysm was defined as an aneurysm that arose between the vertebrobasilar junction and the origin of the superior cerebellar artery. Involved segments of BA were defined as (B) upper-third (n=12), (C) middle-third (n=13), and (D) lower-third (n=11). (E) A long lesion was defined as an aneurysm involving more than two segments (n=4). (B) Left: a ruptured aneurysm, treated by stent-assisted coiling (SAC). (B) Right: a previously treated ruptured aneurysm from the BA fenestration, re-treated by a flow diverter. (C) Left: a symptomatic unruptured aneurysm, treated by SAC. (C) Right: a ruptured aneurysm, treated by SAC. (D) Left: an unruptured aneurysm, treated by SAC. (D) Right: a ruptured aneurysm, treated by coiling without stents. (E) Left: a ruptured aneurysm, treated by bilateral vertebral artery occlusion. (E) Right: an unruptured aneurysm, treated by flow diverters.

of the aneurysm and categorized as small (<10 mm), large (10–24 mm), and giant (≥25 mm). The mean aneurysm length and width were 15.6 mm (range 0.5–68 mm) and 10.6 mm (range 0.5–43 mm), respectively. There were 20 small, 11 large, and nine giant aneurysms. A wide-neck aneurysm was defined either as neck diameter ≥4 mm or dome-to-neck ratio <2. There were 36 aneurysms with wide necks and only four with small necks (mean neck diameter 6.6 mm, range 0.5–27 mm).

Aneurysmal configuration was classified into saccular and fusiform aneurysms. A fusiform aneurysm was defined as a symmetrical or tortuous dilation involving the circumference of the arterial wall, whereas a saccular aneurysm was defined as an eccentric dilation of the main lumen by a variable-sized neck to an extraluminal pouch.[3] There were 29 saccular and 11 fusiform aneurysms. According to the etiology of aneurysm formation, there were 15 dissecting aneurysms and five blister-like aneurysms. Six aneurysms (15.0%) originated from BA fenestration, and three patients (7.5%) had multiple aneurysms. The involved segments of the BA were defined as upper-third (n=12), middle-third (n=13), and lower-third (n=11) segments (figure 1). A long lesion was defined as an aneurysm involving more than two segments (n=4).

Endovascular treatment

Treatment decisions were made by consensus among the neurosurgeons and neurointerventionalists. Generally, we treated saccular aneurysms by simple coiling or stent-assisted coiling. For dissecting aneurysms or fusiform aneurysms, we performed multiple stenting with/without coils or flow diverters. In our country, additional coil insertion along with a flow diverter is not allowed. For large/giant saccular aneurysms, we prefer the reconstructive techniques, such as stent-assisted coiling, or flow diverters, and seldom perform parent artery occlusion. All EVTs were performed under general anesthesia. In unruptured cases, dual antiplatelet agents (daily dose of aspirin 100 mg and clopidogrel 75 mg) were given at least 5 days before and continued at least 3 to 6 months after the procedure if a stent was deployed. Subsequently, a single antiplatelet agent (usually aspirin 100 mg) was administered for at least 1 year. Patients received a bolus of 3000 to 5000 IU heparin just after positioning of the guide catheter, followed by 1000 IU/hour heparin to maintain the activated clotting time above 250 s. In ruptured cases, heparin was not injected intravenously or subcutaneously, but was mixed into the saline flushes during the procedure; thus, the activated clotting time was not measured during the procedure. If a stent was to be used, 300 mg aspirin and 300 mg clopidogrel were loaded via the nasogastric tube before stent deployment. Details of the procedural techniques have been previously reported.[7 8]

In total, 47 endovascular procedures were performed in 40 patients. Initial treatment included coiling without stents (n=9), stent-assisted coiling (SAC; n=17), stent only (n=6), flow diverters (n=5), and vertebral artery occlusion (n=3). Among the 23 patients in whom stents were used, 12 received a single stent, while double stenting was performed in eight patients and triple stenting in three.

Assessment of clinical and angiographic outcomes

Follow-up angiography was performed in 31 of the 40 patients between 1 and 72 months (mean 18.9 months) after the procedure, and clinical follow-up was performed for 3 to 84 months (mean 26.3 months) in 34 of the 40 patients. (It was not performed in six patients due to death.) Clinical outcome was assessed based on the Glasgow Outcome Scale (GOS) at discharge and at final clinical follow-up. A favorable clinical outcome was defined as a GOS score of 4 or 5, and an unfavorable clinical outcome was defined as a GOS score of 1, 2 or

J NeuroIntervent Surg: first published as 10.1136/neurintsurg-2021-017698 on 27 July 2021. Downloaded from http://jnis.bmj.com/ on September 11, 2021 at lane medical library. Protected by copyright.

Table 1 Baseline characteristics and outcomes

Variables	n=40
Age (years)	
<50	19 (47.5)
≥50	21 (52.5)
Female	21 (52.5)
Hypertension	16 (40.0)
Diabetes mellitus	6 (15.0)
Smoking	8 (20.0)
Dyslipidemia	7 (17.5)
Initial presentation	
Subarachnoid hemorrhage	27 (67.5)
Mass effect	7 (17.5)
Incidental finding	6 (15.0)
Hunt and Hess grade	**n=27**
Good (1, 2 or 3)	17 (62.9)
Poor (4 or 5)	10 (37.0)
Fisher grade	**n=27**
Good (1 or 2)	5 (18.5)
Poor (3 or 4)	22 (81.5)
Preoperative comorbidity	
Pontine infarction	2 (5.0)
Pontine hemorrhage	1 (2.5)
Hydrocephalus	13 (32.5)
Aneurysm size	
Small (<10 mm)	20 (50.0)
Large (10–24 mm)	11 (27.5)
Giant (≥25 mm)	9 (22.5)
Wide neck	36 (90.0)
Aneurysm configuration	
Saccular	29 (72.5)
Fusiform	11 (27.5)
Specific condition	
Acute dissection	15 (37.5)
Blister-like	5 (12.5)
Basilar artery fenestration	6 (15.0)
Multiple aneurysms	3 (7.5)
Involved segment of basilar artery	
Upper 1/3	12 (30.0)
Middle 1/3	13 (32.5)
Lower 1/3	11 (27.5)
Long lesion	4 (10.0)
Clinical outcome at discharge	
Favorable (GOS 4 or 5)	27 (67.5)
Unfavorable (GOS 1, 2 or 3)	13 (32.5)
Immediate post-procedural angiographic outcome	
Complete occlusion	24 (60.0)
Neck remnant	6 (15.0)
Incomplete occlusion	10 (20.0)
Follow-up clinical outcome	
Favorable (GOS 4 or 5)	26 (65.0)
Unfavorable (GOS 1, 2 or 3)	14 (35.0)

Continued

Table 1 Continued

Variables	n=40
Follow-up angiographic outcome	**n=31**
Complete occlusion	22 (71.0)
Neck remnant	4 (12.9)
Incomplete occlusion	5 (16.1)

Values are presented as number (%).
GOS, Glasgow Outcome Scale.

3 (a GOS score of 1 was indicative of death). The angiographic results were evaluated by two independent investigators and categorized as complete occlusion, neck remnant (>90% occlusion with small neck remnant), or incomplete occlusion (filling of the aneurysm lumen). A favorable angiographic outcome was defined as complete occlusion or neck remnant, and an unfavorable angiographic outcome was defined as incomplete occlusion.

Statistical analysis

Patient factors included demographic data (age, sex, hypertension, diabetes, smoking, dyslipidemia), initial presentation, HHG, FG, and preprocedural comorbidity (intraparenchymal hemorrhage, infarction, hydrocephalus, and rebleeding). Aneurysm factors were maximal diameter and aneurysmal neck size, aneurysm configuration, and BA segment involvement. Procedural factors were analyzed as treatment methods (reconstructive vs deconstructive treatment), number of devices (single stent vs multiple stents), and type of devices (conventional intracranial stent vs flow diverter).

A biostatistician was consulted for all statistical analyses, which were performed using IBM SPSS Statistics version 26.0 (IBM, Armonk, NY). Univariate logistic regression analysis was performed to determine independent risk factors for unfavorable clinical outcome and unfavorable angiographic outcome at follow-up. Multiple logistic regression analyses were performed on variables with an unadjusted effect and p value <0.10 on simple logistic regression analysis. A p value <0.05 at a 95% confidence interval (95% CI) was considered statistically significant.

RESULTS

Of the 40 patients, immediate postprocedural angiographic outcomes showed complete occlusion in 24 (60.0%), neck remnant in six (15.0%), and incomplete occlusion in 10 (20.0%). Seven patients (17.5%) needed additional treatment due to recanalization (n=6) or initial incomplete occlusion (n=1). Of the seven additional procedures, complete occlusion was achieved in six patients.

Procedure-related complications occurred in five patients (12.5%) including cerebral infarction in two patients, new onset cranial neuropathy due to mass effect in two patients, and aggravation of pre-existing cranial neuropathy in one patient. After initial treatment, three patients who had been treated with stent only, flow diverter, or SAC experienced rebleeding at 3 days, 9 months, and 12 months, respectively.

At discharge, 27 patients (67.5%) achieved favorable clinical outcomes, and the other 13 (32.5%) showed unfavorable clinical outcomes including four deaths. GOS at discharge was grade 5 in 17 patients, grade 4 in 10, grade 3 in three, grade 2 in six, and grade 1 in four. During the mean follow-up period of 26.3 months, 26 patients (65.0%) achieved favorable clinical outcomes (table 1). GOS grades at follow-up were grade 5 in 21 patients, grade 4 in 5, grade 3 in four, grade 2 in four, and grade

J NeuroIntervent Surg: first published as 10.1136/neurintsurg-2021-017698 on 27 July 2021. Downloaded from http://jnis.bmj.com/ on September 11, 2021 at lane medical library. Protected by copyright.

J NeuroIntervent Surg: first published as 10.1136/neurintsurg-2021-017698 on 27 July 2021. Downloaded from http://jnis.bmj.com/ on September 11, 2021 at lane medical library. Protected by copyright.

Table 2 Risk factors for unfavorable clinical outcomes in patients with basilar artery trunk aneurysms

	Clinical outcomes (n=40)		Unadjusted*	Adjusted*	
Variables	Unfavorable (n=13)	Favorable (n=27)	P value	OR (95% CI)	P value
Age ≥50 years	9 (69.2)	12 (44.4)	0.27	2.10 (0.55 to 8.00)	0.34
Female	7 (53.8)	14 (51.9)	1.00	0.89 (0.23 to 3.42)	0.89
Hypertension	8 (61.5)	8 (29.6)	0.07	3.92 (0.48 to 22.46)	0.12
Diabetes	2 (15.4)	4 (14.8)	1.00		
Smoking	3 (23.1)	5 (18.5)	0.95		
Dyslipidemia	1 (7.7)	6 (22.2)	0.85		
Ruptured aneurysm	10 (76.9)	17 (63.0)	0.72		
Poor initial HHG (4 or 5)	7 (53.8)	3 (11.1)	0.009	7.67 (1.55 to 37.80)	0.018
Large/giant aneurysm	7 (53.8)	13 (48.1)	0.84		
Wide neck	12 (92.3)	24 (88.9)	0.96		
Long lesion	1 (7.7)	3 (11.1)	0.93		
Fusiform aneurysm	6 (46.2)	5 (18.5)	0.09	3.15 (0.75 to 13.29)	0.11
Dissection	5 (38.5)	10 (37.0)	1.00		
Blister-like aneurysm	3 (23.1)	2 (7.4)	0.32		
Aneurysm arising from BA fenestration	0 (0.0)	6 (22.2)	0.14		
Pontine infarction	1 (7.7)	1 (3.7)	0.77		
Pontine hemorrhage	1 (7.7)	0 (0.0)	0.61		
Hydrocephalus	7 (53.8)	6 (22.2)	0.08	2.91 (0.63 to 9.08)	0.22
Rebleeding before EVT	1 (7.7)	0 (0.0)	0.61		
Deconstructive treatment	2 (15.4)	1 (3.7)	0.52		
Multiple use of stent	5 (38.5)	6 (22.2)	0.45		
Flow diversion	2 (15.4)	3 (11.1)	0.89		

*Logistic regression analysis.
BA, basilar artery; EVT, endovascular treatment; HHG, Hunt and Hess grade.

1 in two. The overall mortality rate was 15% (6/40). Causes of mortality were brain stem compression in three patients, initial poor grade subarachnoid hemorrhage in two patients, and pneumonia in one patient.

Follow-up angiography was performed in 31 patients (77.5%) during the follow-up period of 18.9 months (table 1). Complete occlusion was achieved in 22 patients (71.0%), neck remnant in four (12.9%), and incomplete occlusion in five (16.1%). Overall, favorable follow-up angiographic outcomes (complete occlusion or neck remnant) were achieved in 26 patients (83.9%).

Hypertension, poor initial HHG, fusiform aneurysm, and hydrocephalus, with p values <0.10 on simple logistic regression analysis, were included in the multiple logistic regression analysis with age and sex adjustment to determine risk factors for unfavorable clinical outcomes. Poor initial HHG (odds ratio (OR) 7.67, 95% CI 1.55 to 37.80; p=0.018) remained the only independent risk factor for unfavorable clinical outcome (table 2). Age, hypertension, large/giant aneurysm, long lesion, and flow diversion, with p value <0.10 on simple logistic regression analysis, were included in the multiple logistic regression analysis with age and sex adjustment to determine risk factors for unfavorable angiographic outcomes at follow-up. Large/giant aneurysm (OR 8.14, 95% CI 1.88 to 27.46; p=0.047) and long lesion (OR 14.25, 95% CI 1.48 to 69.80; p=0.013) remained independent risk factors for unfavorable angiographic outcomes at follow-up (table 3).

DISCUSSION

The present study reports long-term clinical and angiographic follow-up results of EVT in 40 patients with 40 BA trunk aneurysms and analyzed risk factors for unfavorable outcomes. Favorable clinical and angiographic outcomes were achieved in 65.0% (mean 26.3 months) and 83.9% (mean 18.9 months) of patients, respectively. EVT of large/giant aneurysms or aneurysms with a long lesion of the BA trunk might show unfavorable angiographic outcomes.

Aneurysms of the BA are classified into four subgroups in surgical series as follows: (1) BA bifurcation aneurysms; (2) BA–superior cerebellar artery aneurysms; (3) BA trunk aneurysms; and (4) vertebrobasilar junction aneurysms. However, there is an inconsistency in clinical series whether BA–superior cerebellar artery aneurysms and vertebrobasilar junction aneurysms are included as BA trunk aneurysms. To clarify the true clinical characteristics of BA trunk aneurysms, this study defined aneurysms arising at the non-branching site of the BA trunk as 'pure BA trunk aneurysms'.

BA trunk aneurysms have two distinct characteristic features such as so-called lateral aneurysm[9] and fusiform aneurysms. In general, lateral aneurysms tend to have saccular features with various sizes of the aneurysmal neck.[9] Fusiform aneurysms consist of chronic dolichoectatic segmental dilatation with a stretched and fragmented internal elastic lamina, and dissecting aneurysms involve widespread disruption of the elastic lamina.[10] Conversely, BA dissecting aneurysms are somewhat unique

Table 3 Risk factors for unfavorable angiographic outcomes in patients with basilar artery trunk aneurysms

	Angiographic outcomes (n=31)		Unadjusted*	Adjusted*	
Variables	Unfavorable (n=5)	Favorable (n=27)	P value	OR (95% CI)	P value
Age ≥50 years	4 (80.0)	11 (40.7)	0.03	2.66 (0.85 to 11.30)	0.08
Female	2 (40.0)	4 (14.8)	0.29	1.93 (0.63 to 7.43)	0.31
Hypertension	3 (60.0)	8 (29.6)	0.07	4.95 (0.89 to 19.49)	0.11
Diabetes	1 (20.0)	4 (14.8)	0.91		
Smoking	2 (40.0)	5 (18.5)	0.39		
Dyslipidemia	2 (40.0)	4 (14.8)	0.29		
Ruptured aneurysm	2 (40.0)	18 (66.7)	0.18		
Poor initial HHG (4 or 5)	0 (0.0)	4 (14.8)	0.59		
Large/giant aneurysm	4 (80.0)	11 (40.7)	0.03	8.14 (1.88 to 27.46)	0.047
Wide neck	3 (60.0)	15 (55.6)	0.98		
Long lesion	2 (40.0)	0 (0.0)	0.02	14.25 (1.48 to 69.80)	0.013
Fusiform aneurysm	2 (40.0)	5 (18.5)	0.39		
Dissection	2 (40.0)	9 (33.3)	0.84		
Blister-like aneurysm	0 (0.0)	3 (11.1)	0.65		
Aneurysm arising from BA fenestration	0 (0.0)	6 (22.2)	0.35		
Deconstructive treatment	0 (0.0)	2 (7.4)	0.81		
Multiple use of stent	2 (40.0)	6 (22.2)	0.50		
Flow diversion	2 (40.0)	2 (7.4)	0.049	2.28 (0.79 to 11.88)	0.06

*Logistic regression analysis.
BA, basilar artery; EVT, endovascular treatment; HHG, Hunt and Hess grade.

in their mechanism of development. BA dissecting aneurysms accounted for 1.0% of all cases of subarachnoid hemorrhage, and 10.5% of posterior circulation aneurysms.[11] The size of the BA trunk aneurysms tends to be variable, with previous clinical studies reporting a range from 3 mm to 45 mm in maximal diameter.[3 10 12–14] In the present study, aneurysmal size varied strikingly, from 0.5 mm to 68 mm. Large/giant aneurysms comprised 50.0% of the cases, fusiform aneurysms 27.5%, and wide-necked aneurysms 90.0%. The variable size of the aneurysms contributed to variations in aneurysm morphology: 12.5% of the BA aneurysms were blister-like, and 37.5% were dissecting aneurysms. Various sizes and morphologies, such as fusiform or dissecting aneurysms, might have associations with procedure-related complications and unfavorable angiographic outcomes.

The key point for successful treatment of a BA trunk aneurysm is complete occlusion with preservation of all involved arteries. Technical success rate and good clinical outcome have been reported in 93.8% and 78.6% of patients, respectively.[12] Immediate angiographic results have been reported as 31.4–73.3% complete occlusion[12 13] and 26.7% incomplete occlusion.[12] Notably, the incomplete occlusion rate in cases of aneurysm rupture is high, at 66.7–80.0%.[8 12 13] The overall recanalization rates of EVT of BA trunk aneurysms have been reported as 20.9–33.3%.[2 12] In one clinical series of 35 BA trunk aneurysms treated with EVT, 42.9% of the aneurysms with immediate incomplete occlusion resulted in recanalization on follow-up angiography, in contrast to 0% of those with initial complete occlusion.[13] Pandey *et al* reported a significant difference in recanalization rates between complete and incomplete occlusion at 16.2% and 50%, respectively.[2] In the present study, however, initial angiographic outcomes did not affect follow-up angiographic outcomes. We identified two cases of complete occlusion and two cases of neck remnant at immediate post-procedure that developed into incomplete occlusion at follow-up. Also, five cases of incomplete occlusion at immediate post-procedure resolved to complete occlusion in four patients and neck remnant in one patient at follow-up. The reason for the high recurrence rates might be that most BA trunk aneurysms are large or giant size with wide neck or fusiform configuration. Moreover, in the present study, a long lesion involving more than two segments of the BA trunk was an additional risk factor for unfavorable angiographic outcome.

No difference was noted in clinical or angiographic outcomes among the various treatment techniques. Specific techniques or devices were assumed to not be critical factors affecting the angiographic outcomes, but long lesions involving the BA trunk, which need to be treated with multiple stents or a flow diverter, might be difficult to treat initially. If technically possible, total occlusion of the aneurysm sac with a coil seems to be the ideal treatment method. However, coil compaction or recanalization of the aneurysm can raise concerns, particularly in large/giant aneurysms.[4] In cases of dissection or too small aneurysms involving the perforator, stenting (single stent, multiple overlapping or telescoping stents) is another option.[8 12] Multiple overlapping stents can accelerate aneurysm thrombosis by reducing stent porosity to result in further disruption of inflow in the aneurysm.[14 15] In addition, stent placement provides a physiologic scaffolding for neointimal growth over the aneurysmal ostium.[4] Flow diverters can be applied to large/giant fusiform aneurysms.[4 16] The flow diversion technique is a valuable option for treating lesions that are not amenable to conventional clipping or coiling and can achieve complete aneurysm occlusion without superselection of a microcatheter or delivery of coils through the aneurysm. The technique also maintains patency of perforating vessels by a pressure gradient.[4] It remains to be determined whether or not the remaining aneurysm should be filled with coils.[10] Larger prospective studies are necessary to evaluate clinical and angiographic outcomes of BA trunk aneurysms based on treatment modality.

J NeuroIntervent Surg: first published as 10.1136/neurintsurg-2021-017698 on 27 July 2021. Downloaded from http://jnis.bmj.com/ on September 11, 2021 at lane medical library. Protected by copyright.

This study has several limitations. First, all five medical institutes are tertiary referral centers, so subjects included in this study, who may be referred due to difficult-to-treat BA aneurysms, have lesions that are more complicated. Second, even though this study dealt with the largest reported series of BA trunk aneurysms to date, the number of cases was small for deriving statistical power. In statistics, the one-in-10 rule is based on two simulation studies and a rule of thumb for how many predictor parameters can be estimated from data when doing logistic and Cox models.[17–19] The rule states that one predictive variable can be studied for every 10 events. Additionally, there are rules requiring 20 or more predictor variables, indicating the need for shrinkage of regression coefficients.[18 20] Currently, our statistical model had less than seven subjects per predictive variable and needs validation in large data or trials with large sample sizes. However, another study shows that the one-in-10 rule may be too conservative as a general recommendation and that five to nine events per predictor can be enough, depending on the research question.[21] Despite the rarity of BA trunk aneurysms, we tried our best to evaluate risk factors for EVT with small sample sizes as far as possible in the present study. Finally, because of the rarity of BA trunk aneurysms, the data collection period was 16 years. There has been a considerable trend change in treatment of large/giant or fusiform aneurysms in posterior circulation during that time; therefore, the therapeutic approach for some of the early subjects might be different from the recent treatment trends.

CONCLUSIONS

BA trunk aneurysms have diverse clinical and angiographic features and are difficult to treat, requiring various techniques and multiple procedures. One feasible option for this rare disease entity is EVT. Favorable clinical and angiographic outcomes were achieved in 65.0% and 83.9% of patients, respectively. Unfavorable angiographic outcome might be expected in large/giant aneurysms or long lesions. Further studies with large sample sizes are needed to better understand how EVT can be used to treat BA trunk aneurysms successfully.

Contributors Substantial contribution to the conception or design of the work: JC and YCL. Acquisition, analysis, or interpretation of the data for the work: SYS, JC, JHC, MJK, YSS, and YCL. Drafting the work or revising it critically for important intellectual content: SYS, JC, JHC, MJK, YSS, and YCL. Final approval of the version to be published: SYS, JC, JHC, MJK, YSS, and YCL. Agreement to be accountable for all aspects of the work in ensuring that questions related to the accuracy or integrity of any part of the work are appropriately investigated and resolved: SYS, JC, JHC, MJK, YSS, and YCL.

Funding This work was supported by (a Grant from Research Year of Inje University) grant number [20 170 109].

Competing interests None declared.

Patient consent for publication Not required.

Provenance and peer review Not commissioned; externally peer reviewed.

ORCID iD
Joonho Chung http://orcid.org/0000-0003-2745-446X

REFERENCES

1 Nakatomi H, Kiyofuji S, Ono H, *et al*. Giant fusiform and dolichoectatic aneurysms of the basilar trunk and vertebrobasilar junction—clinicopathological and surgical outcome. *Neurosurgery* 2020;88:82–95.
2 Pandey AS, Koebbe C, Rosenwasser RH, *et al*. Endovascular coil embolization of ruptured and unruptured posterior circulation aneurysms: review of a 10-year experience. *Neurosurgery* 2007;60:626–36.
3 Saliou G, Sacho RH, Power S, *et al*. Natural history and management of basilar trunk artery aneurysms. *Stroke* 2015;46:948–53.
4 Fiorella D, Kelly ME, Albuquerque FC, *et al*. Curative reconstruction of a giant midbasilar trunk aneurysm with the Pipeline embolization device. *Neurosurgery* 2009;64:212–7.
5 Hassan T, Ezura M, Takahashi A. Treatment of giant fusiform aneurysms of the basilar trunk with intra-aneurysmal and basilar artery coil embolization. *Surg Neurol* 2004;62:455–62.
6 Lawton MT, Daspit CP, Spetzler RF. Technical aspects and recent trends in the management of large and giant midbasilar artery aneurysms. *Neurosurgery* 1997;41:513–20.
7 Cho DY, Kim B-S, Choi JH, *et al*. The fate of unruptured intracranial vertebrobasilar dissecting aneurysm with brain stem compression according to different treatment modalities. *AJNR Am J Neuroradiol* 2019;40:1924–31.
8 Chung J, Park H, Lim YC, *et al*. Endovascular treatment of basilar artery trunk aneurysms. *Acta Neurochir* 2011;153:2137–45.
9 Higa T, Ujiie H, Kato K, *et al*. Endovascular treatment of basilar trunk saccular aneurysms. *Neuroradiol J* 2011;24:687–92.
10 van Oel LI, van Rooij WJ, Sluzewski M, *et al*. Reconstructive endovascular treatment of fusiform and dissecting basilar trunk aneurysms with flow diverters, stents, and coils. *AJNR Am J Neuroradiol* 2013;34:589–95.
11 Ruecker M, Furtner M, Knoflach M, *et al*. Basilar artery dissection: series of 12 consecutive cases and review of the literature. *Cerebrovasc Dis* 2010;30:267–76.
12 Cho K-C, Jeon P, Kim BM, *et al*. Saccular or dissecting aneurysms involving the basilar trunk: endovascular treatment and clinical outcome. *Neurol Res* 2019;41:671–7.
13 Uda K, Murayama Y, Gobin YP, *et al*. Endovascular treatment of basilar artery trunk aneurysms with Guglielmi detachable coils: clinical experience with 41 aneurysms in 39 patients. *J Neurosurg* 2001;95:624–32.
14 Wang Q, Leng B, Song D, *et al*. Fusiform aneurysms of the vertebrobasilar arterial trunk: choice of endovascular methods and therapeutic efficacy. *Acta Neurochir* 2010;152:1467–76.
15 Kim BM, Suh SH, Park SI, *et al*. Management and clinical outcome of acute basilar artery dissection. *AJNR Am J Neuroradiol* 2008;29:1937–41.
16 Siddiqui AH, Abla AA, Kan P, *et al*. Panacea or problem: flow diverters in the treatment of symptomatic large or giant fusiform vertebrobasilar aneurysms. *J Neurosurg* 2012;116:1258–66.
17 Concato J, Peduzzi P, Holford TR, *et al*. Importance of events per independent variable in proportional hazards analysis. I. background, goals, and general strategy. *J Clin Epidemiol* 1995;48:1495–501.
18 Harrell FE, Lee KL, Mark DB. Multivariable prognostic models: issues in developing models, evaluating assumptions and adequacy, and measuring and reducing errors. *Stat Med* 1996;15:361–87.
19 Peduzzi P, Concato J, Kemper E, *et al*. A simulation study of the number of events per variable in logistic regression analysis. *J Clin Epidemiol* 1996;49:1373–9.
20 Steyerberg EW, Eijkemans MJ, Harrell FE, *et al*. Prognostic modelling with logistic regression analysis: a comparison of selection and estimation methods in small data sets. *Stat Med* 2000;19:1059–79.
21 Vittinghoff E, McCulloch CE. Relaxing the rule of ten events per variable in logistic and COX regression. *Am J Epidemiol* 2007;165:710–8.

J NeuroIntervent Surg: first published as 10.1136/neurintsurg-2021-017698 on 27 July 2021. Downloaded from http://jnis.bmj.com/ on September 11, 2021 at lane medical library. Protected by copyright.
