# Supplementary material for: Management strategies and outcomes of basilar trunk aneurysms: a systematic review and meta-analysis
Source: Langenbecks Arch Surg. 2026 Jan 20;411(1):71. doi: 10.1007/s00423-025-03959-3 (PMC12852281; doi:10.1007/s00423-025-03959-3)
Supplement: Supplementary file 1 — Supplementary file1 (ZIP 55327 KB) [file 423_2025_3959_MOESM1_ESM.zip › literature.zip!/meta/tjahjadi 2016.pdf]

Accepted Manuscript

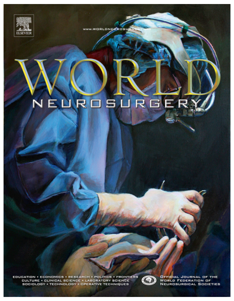

Presigmoid Approach to Vertebrobasilar Artery Aneurysms: A Series of 31 Patients and Review of the Literature

Mardjono Tjahjadi, MD, Mika Niemelä, MD PhD, Juri Kivelev, MD PhD, Joseph Serrone, MD, Hidetsugu Maekawa, MD, Behnam Rezai Jahromi, MB, Oleg Kerro, MD, Ahmad Hafez, MD, Hanna Lehto, MD, Riku Kivisaari, MD PhD, Juha Hernesniemi, MD PhD

PII: S1878-8750(16)30262-5

DOI: 10.1016/j.wneu.2016.05.001

Reference: WNEU 4062

To appear in: *World Neurosurgery*

Received Date: 2 April 2016

Accepted Date: 2 May 2016

Please cite this article as: Tjahjadi M, Niemelä M, Kivelev J, Serrone J, Maekawa H, Jahromi BR, Kerro O, Hafez A, Lehto H, Kivisaari R, Hernesniemi J, Presigmoid Approach to Vertebrobasilar Artery Aneurysms: A Series of 31 Patients and Review of the Literature, *World Neurosurgery* (2016), doi: 10.1016/j.wneu.2016.05.001.

This is a PDF file of an unedited manuscript that has been accepted for publication. As a service to our customers we are providing this early version of the manuscript. The manuscript will undergo copyediting, typesetting, and review of the resulting proof before it is published in its final form. Please note that during the production process errors may be discovered which could affect the content, and all legal disclaimers that apply to the journal pertain.

ACCEPTED MANUSCRIPT

PRESIGMOID APPROACH TO VERTEBROBASILAR ARTERY ANEURYSMS: A SERIES OF 31 PATIENTS AND REVIEW OF THE LITERATURE

Mardjono Tjahjadi MD , Mika Niemelä MD PhD, Juri Kivelev MD PhD, Joseph Serrone MD, Hidetsugu Maekawa MD , Behnam Rezai Jahromi MB, Oleg Kerro MD, Ahmad Hafez MD, Hanna Lehto MD, Riku Kivisaari MD PhD, Juha Hernesniemi MD PhD

Department of Neurosurgery, University of Helsinki and Helsinki University Hospital **-** *Finland*

ABSTRACT

Objective : For treating vertebrobasilar artery aneurysms, the presigmoid approach can be used when circumstances require more operative exposure. High morbidity and mortality rates on these cases have been reported. In this study, we describe our modified presigmoid approach for vertebrobasilar artery aneurysms and our clinical results.

Methods : This series includes patients with vertebrobasilar aneurysms clipped via the modified presigmoid approach at the Department of Neurosurgery at Helsinki University Hospital from 1998-2014. Data were collected from the operating record books, patients' medical records, and a radiological database server.

Results : Thirty-three presigmoid procedures of 31 patients were performed to treat 34 aneurysms(14 ruptured, 20 unruptured). The aneurysms had a mean distance from posterior clinoid process (PCP) to the aneurysm neck of 12.2 mm (range 0 – 26.6 mm). Favorable outcome was achieved in 21 patients (64%). In unruptured and good-grade patients, favorable outcome was achieved in 74% of patients, while in poor-grade patients favorable outcome was only achieved in 36% of patients. Complete or near complete occlusion was achieved in 79%. Larger aneurysms, fusiform morphology, and anterior dome projection had lower occlusion rates.

Conclusion: We have described our experiences of utilizing the presigmoid approach to treat vertebrobasilar aneurysms. The clinical and radiographic results are acceptable given the

complex location and configuration of the treated aneurysms. Unfavorable outcomes are related to the poor admission Hunt-Hess grade, aneurysm's morphology, and aneurysm's size.

Keywords : Vertebrobasilar aneurysm ; Presigmoid approach

PRESIGMOID APPROACH TO VERTEBROBASILAR ARTERY ANEURYSMS: A SERIES OF 31 PATIENTS AND REVIEW OF THE LITERATURE

Mardjono Tjahjadi MD , Mika Niemelä MD PhD, Juri Kivelev MD PhD, Joseph Serrone MD, Hidetsugu Maekawa MD , Behnam Rezai Jahromi MB, Oleg Kerro MD, Ahmad Hafez MD, Hanna Lehto MD, Riku Kivisaari MD PhD, Juha Hernesniemi MD PhD

Department of Neurosurgery, University of Helsinki and Helsinki University Hospital - Finland

INTRODUCTION

The presigmoid approach (also referred to as the posterior transpetrosal approach or combined supra-infratentorial approach) is considered one of the most complex procedures in microneurosurgery. This approach was originally introduced as a surgical route to treat the ventral brainstem and clival tumors but it has also been applied to posterior circulation aneurysms that were not feasible for standard pterional, subtemporal or retrosigmoid approaches. [1] In the early years of skull base surgery, this procedure was performed through the infratentorial corridor with sacrificing the sigmoid sinus and labyrinth. This approach resulted in hearing loss and complications related to venous hypertension [2, 3]. Hakuba later introduced his modification of a combined partial labyrinthectomy and petrous apicectomy to treat retrochiasmatic craniopharyngiomas and clival meningiomas. This technique avoided sacrifice of the sigmoid sinus with improved hearing outcomes and less complications related to venous insufficiency. [4, 5]

Recently, many less invasive variations of the presigmoid approach have been described including retrolabyrinthine, transcrusal, minimum combined transpetrosal, and partial supero-posterior petrosectomy.[6-12] However, the presigmoid approach still carries a relatively high

ACCEPTED MANUSCRIPT

morbidity (62%) and mortality rate (9%). [10, 11] After the publication of the ISAT and ISUIA trials demonstrating competitive results of endovascular therapy for aneurysm treatment, endovascular therapy has become more common in general but especially for treating posterior circulation aneurysms. [13-15] Nonetheless, not all vertebrobasilar aneurysms can be treated well by endovascular means. Some drawbacks of the endovascular treatment of cerebral aneurysms in either clinical or radiological evaluation have been reported.[16-19] In certain cases, microsurgery still has a role in treating posterior circulation aneurysms.

In this study, we aim to review our experience in utilizing a modified presigmoid approach to treat vertebrobasilar aneurysms and to correlate the pre-operative clinical and radiological characteristics to patient outcomes.

MATERIALS AND METHODS

This retrospective review includes a consecutive series of vertebrobasilar aneurysm patients treated by our modified presigmoid approach at the Department of Neurosurgery at Helsinki University Central Hospital (catchment area of 1.8 million inhabitants) between 1998-2014. Data was collected from the operating record books, patients' medical records, and a radiological database server.

Patient data

Our review included 3492 patients with 4677 intracranial aneurysms. From this population, 601 aneurysms were located in the vertebrobasilar system. A microsurgical procedure was performed for 421 aneurysms, endovascular therapy in 66 aneurysms, and conservative management in 114. The cases managed conservatively were mainly due to poor clinical condition at presentation for ruptured cases or small aneurysm size (< 3 mm) in unruptured cases indicating a lower risk for rupture.

Clinical data

Preoperative clinical condition was assessed using the Hunt-Hess score.[20] The outcome after surgery was assessed using the modified Rankin Scale with classification into three categories: favorable if the condition is similar to the pre-operative condition or with minor

ACCEPTED MANUSCRIPT

neurologic signs that did not disturb their life activities including walking satisfactorily (GOS 4 – 5); unfavorable if the condition is worse postoperatively with moderate to severe neurologic deficit (GOS 2 – 3), and death (GOS 1).

Radiological Measurement

Radiological data for the study was obtained from the available images of computed tomography angiography (CTA) and digital subtraction angiography (DSA). Radiological images were analyzed both in 2D and 3D using the GE Centricity RA 600 software and the IMPAX software version 5.3. The aneurysm characteristics for statistical analysis included the size of aneurysm dome and neck, vertical distance from the aneurysm neck to the PCP, the lateralization of the aneurysm neck or parent artery with respect to the clivus, direction of aneurysm dome projection, and post-operative angiographic occlusion of the aneurysm. Patients with incomplete radiological data were excluded from this study. Aneurysm size was categorized as: very small < 3 mm, small 3-6 mm, medium 7-13 mm, large 14-24 mm, or giant ≥ 25mm. The radiologic evaluation of aneurysm occlusion was performed on the first post-operative day and divided into three categories; complete occlusion, near complete occlusion (any signs of neck remnant), and incomplete occlusion (fundus remnant).

OPERATIVE TECHNIQUE AND METHODS (Figure-1)

Although the details of the presigmoid approach have been described elsewhere, below we review our modifications of this approach.[21]

Patient Position and Cerebrospinal Fluid Drainage

The patient is placed in the park bench position with the mastoid process as the highest point. The upper shoulder is carefully retracted caudally and backward with tape to increase the surgeon's working space. Lumbar cerebrospinal fluid (CSF) drainage (50 ml) helps to provide a slack brain before the dural opening and this action facilitates the subtemporal route to the interpeduncular cistern.

Skin Incision And Craniotomy

The one-layer horseshoe incision forming musculocutaneous flap is performed from a

point about 1 cm anterior and superior of the root of the zygoma, arching posterosuperiorly 2-3 cm over the ear and stopping 2 cm posterior to the occipitomastoid suture. This myocutaneous flap is retracted fronto-caudally and fixated with several Sugita spring-hook retractors to expose the temporal and occipital-retrosigmoid area. The craniotomy is made to expose the dura of the middle fossa and lateral suboccipital area. An extradural dissection is performed to expose the subtemporal dural and the upper part of sinodural angle.

Temporal Bone Drilling

Drilling is performed under high magnification of the operating microscope. The base of squamous part of temporal bone is drilled first to support an adequate supratentorial surgical corridor with minimal retraction of the temporal lobe. Drilling the posterosuperior mastoid part of the temporal bone increases the exposure of the sinodural angle, which has been exposed after the craniotomy. This angle or the presigmoid dura is exposed just as necessary and drilling stops at the level of anthrum without compromising the structures of the middle or inner ear. During this procedure, gradual and cautious detachment of the dura mater from the inner surface of the petrous-mastoid temporal bone is important to prevent the damage of the sigmoid sinus. The transverse and sigmoid sinuses are left intact. The postoperative result of the temporal bone drilling can be seen in Figure-2.

Dural Opening and cutting of the tentorium

The dura mater is opened in a J-shape that involves the subtemporal and presigmoid areas. The first opening is a horizontal incision of subtemporal dura mater that extends posteriorly towards the sinodural angle. The second opening is the vertical incision of the presigmoid dura mater. These two dural openings are connected by dividing the superior petrosal sinus. The tentorium is cut to the incisura just posterior to the insertion of the fourth nerve into the tentorial edge. The mobilized anterior flap of the tentorium is folded anteriorly and fixed to the anterior tentorium with small aneurysm clips (Figure-1).

Closing

The closure for this approach is of utmost importance to avoid post-operative CSF leakage and

ACCEPTED MANUSCRIPT

infection. Dura mater is closed in a watertight fashion and if necessary reinforced by dural sealants. Any open mastoid air-cells are identified and sealed with a pedicle of the temporal muscle flap. This flap is very good at promoting the granulation between the muscle and the air-cells mucosa that will close the defect. Bone, soft tissue, and skin closure should be performed in layers under high magnification of the operating microscope.

RESULTS

From 1998 – 2014, the senior author (J.H.) performed 36 presigmoid approaches to operate on 37 vertebrobasilar aneurysms in 34 patients. Three patients were excluded because of incomplete radiological data for the analysis leaving 31 patients with 33 procedures on 34 vertebrobasilar aneurysms. Two of the patients came from outside Finland but could be followed up post-operatively. `

General characteristics of the patients

The male:female ratio was 1:1 (16 males and 17 females) with a mean age of 47.9 years (range 28 – 68 years). Three of 31 patients had two basilar artery aneurysms. Two of these patients required bilateral approaches because of the different origin and projections of the basilar artery aneurysms. Twenty aneurysms (59 %) were ruptured with a median Hunt-Hess score being 3 (Table 1). The mean duration of surgery from skin incision to skin closure was 189 minutes (70 – 257 minutes).

General Aneurysm Characteristics

Aneurysm Morphology

The aneurysms were saccular in 29 cases (85%), fusiform in four cases (12%), and dissecting in one case (3%). The mean aneurysm dome size was 9.8 mm (range from 2.6 mm to

ACCEPTED MANUSCRIPT

38.4 mm). The mean aneurysm neck size for saccular and dissecting aneurysms was 5.5 mm with a mean dome-to-neck ratio of 1.6. The majority of aneurysms were small and medium-sized accounting for 17 (35%) and 10 (29%) cases, respectively (Table 1).

Aneurysm Location

The most common site of the aneurysm was the basilar trunk, accounting for 14 aneurysms (41%). In decreasing order of frequency, the remaining aneurysms were located at the basilar bifurcation, basilar-SCA, basilar-AICA, VBJ, and distal vertebral artery (Table 2 and Figure 3). The mean distance from PCP to proximal neck of the aneurysm was 12.2 mm (range 0 – 26.6 mm). The lateralization of the aneurysm proximal neck was as follows: 26 midline aneurysms (76%), 5 right-sided (15%), and 3 left-sided (9%). The presigmoid approach was performed from the right side in 24 procedures (73%) and from the left side in 9 procedures (27%). Aneurysms at the midline were approached from the right side in 21 patients (81%) from the left side in 5 patients (19%).

Radiographic Outcome

A complete or near-complete aneurysm occlusion was achieved in 27 cases (79 %). The mean aneurysm size for aneurysms with complete occlusion, neck remnant, and fundus remnant was 7.8 mm, 9.9 mm, and 16.7 mm, respectively. Several other factors were also found to predict fundus remnants including fusiform morphology (75%), vertebrobasilar junction location (50%), and anterior dome projection (50%). (Table 3).

Clinical Outcome

Clinical outcome was evaluated with a mean follow-up of 6.2 months (range 1 – 30 months). The median GOS in this series was 4. Postoperative CSF leak was seen in 2 cases (6%), one of them also developed a post-operative wound infection. The CSF leaks were transient and healed with spinal drainage and antibiotics. Favorable outcome was achieved in 21 patients (64%). Favorable outcome was more common in unruptured cases (71%) compared to

ACCEPTED MANUSCRIPT

ruptured cases (58%). Unfavorable outcome occurred in 7 patients (36 %) and 5 patients died (15%). (Table 2) Four of the mortalities (80%) had poor Hunt-Hess grade on admission. The only unruptured case that had a fatal outcome was an Ehlers-Danlos patient with a medium-sized basilar trunk aneurysm. This patient experienced an intraoperative rupture because the arterial wall was extremely fragile. The situation was successfully managed, Indocyanine green (ICG) angiography showed a well contrast filling of basilar artery and perforators, but severe basilar artery vasoconstriction occurred post-operatively with subsequent post-operative brainstem stroke resulting in death.

Post-operative permanent cranial nerves deficit were seen in 5 patients (16 %) (Table 4). Three patients developed both cranial nerve VII and VIII palsy, 1 patient developed an abducens palsy, and 1 patient developed cranial nerve III and IV palsy. Transient cranial nerves deficit occurred in 13 (39%) patients. Six of them (46%) had multiple cranial nerves deficits. Diplopia associated with abducens nerve palsy was the most common transient post-operative cranial nerve deficit and this was seen in 10 patients (32%). Hemiparesis occurred in 8 patients with seven (88%) patients recovering to normal motor function.

DISCUSSION

The presigmoid approach for treating vertoebrobasilar aneurysms is utilized in a very selective manner.[22, 23] A current literature review of the posterior transpetrosal approach to aneurysms in the posterior circulation found only 34 patients published in the last 20 years.[10] Our series is comprised of 33 procedures in 31 patients making it the largest series to date; moreover all the consecutive cases were done by a single surgeon (J.H.) in a single center. No patient was lost to follow-up.

In the present series, we demonstrated a simplified classic posterior petrosal / presigmoid approach with a tailored petrosal bone drilling. This approach modification was invented by the senior author (JH). In most cases, we do not need to drill the petrosal bone extensively and usually stop before reaching the semicircular canals. In order for this more limited bony removal to still provide the needed exposure, cooperation with the

ACCEPTED MANUSCRIPT

neuroanesthesist to maintain a slack brain and the utilization of very high magnification during surgery is required.[24] With this strategy, surgical times are limited to approximately three hours. Only 2 (6%) patients developed post-operative CSF leakage, which was treated without surgical intervention. These findings are interesting since the literature reports a CSF leak rate of 21%. [10] We believe the technique of a tailored bone drilling and closing the opened air-cells with a temporalis muscle pedicle are keys to the prevention for CSF leakage.

Until now, many surgical approaches have been proposed to reach retroclival basilar artery aneurysms including anterolateral, lateral, or posterolateral routes.[6, 22, 25, 26] The anterolateral route (e.g. pterional, orbitozygomatic, pretemporal, and their modifications) gives a relatively shorter working distance to reach the region.[27] The main disadvantage of this approach is the limited access to the lower segment of the basilar artery, even after the removal of the PCP and petrous apex.[28-31] The lateral route consists of subtemporal approach and its extensions.[32-35] This approach for basilar aneurysms was introduced and popularized by Charles Drake and continues to be used commonly by the senior author (J.H.).[33, 36] Additional removal of the petrosal apex, which was popularized by Kawase et al., allows surgeons to reach basilar aneurysms up to 18 mm below the sellar floor.[34, 37]

The posterolateral route (e.g. presigmoid, retrosigmoid, and their modifications) gives a relatively longer working distance, but it exposes lesions at almost all segments of the basilar artery. The presigmoid approach with opening of the dura over the petrous temporal bone, subtemporal dura, and sectioning of the tentorium creates a supratentorial and infratentorial corridor for exposure of the vertebrobasilar system.[1, 3, 4, 12] The disadvantage of the presigmoid approach is its complexity resulting in a longer operative duration and higher risks to injure venous structures, cranial nerves, and the vestibulocochlear system. In some specific situations where the patient has a petrosal or sphenopetrosal venous system, sacrifice of the superior petrosal sinus (SPS) and cutting the tentorium is not safe. Hafez et al. recommended to free the SPS from its groove until the Meckel cave and cut the tentorium below the SPS.[38] In our experience, if the patient has such venous system group, the senior author (J.H.) will work from

supratentorial and infratentorial corridor with cutting only the edge of the tentorium to increase the exposure.

Previous literature emphasizes the level of the aneurysm neck relative to the bony structures (such as the PCP or floor of the sellae turcica) for selection of surgical approaches.[26, 37] Aziz et al. proposed utilizing the presigmoid approach if the aneurysm is located more than 18 mm below the floor of the sellae turcica.[37] In the present series, the aneurysms treated with a presigmoid approach had an average distance being 12.2 mm below the PCP placing most aneurysms well above the cut-off point proposed by Aziz et al. We prefer to use the distance of aneurysms from the PCP to determine surgical approaches since this structure is encountered in surgery whereas the floor of the sellae turcica is only radiographically identified.

Surgical Outcomes and Complications

Even when taking into account the unselected nature of the series the results are comparable to the existing literature (Table 4).[10] The incidence of permanent postoperative cranial nerve injury and hemiparesis occurred in 16 % and 3.2 %, respectively. The death rates were highly related to the poor Hunt-Hess scale on admission.[10] Additionally, we believed a learning curve allowed for lower morbidity at the end of the series.

Clip occlusion rates of the aneurysms were evaluated by post-operative CTA and/or DSA. For saccular aneurysms, the rate for complete occlusion was 71% (non-Titanium clips cause artefact making full estimation of neck remnants very challenging). This finding is consistent with the finding of Kivisaari, et al. in 2004 who reported a 74% rate of occlusion for vertebrobasilar saccular aneurysms operated by the senior author (J.H.). They also considered the aneurysm location, small gap, and size of the aneurysms as reasons for the post-clipping aneurysm remnants, which were consistent to our findings.[39]

Endovascular and Microsurgery Perspective

With endovascular therapy using natural routes one can avoid extensive skull-base approaches and cranial nerve deficits, which are the down-side of microsurgical approaches.

ACCEPTED MANUSCRIPT

However, endovascular procedures also carry risks of complications in cases of vertebrobasilar aneurysms. Even with the rapid development of its technology, complications with endovascular approaches include thrombo-embolic complications, aneurysm recanalization, intra-procedural hemorrhage, in-stent stenosis, jailed perforators, and formation of pseudoaneurysms have been reported and long-term follow-up of the patients is lacking.[16, 18, 19, 40-42] Recent study on flow-diverting stents for posterior circulation aneurysms showed a mortality rate of 12.3 % and permanent neurologic deficit of 11% with mean follow-up time of 9.5 months.[43] For more complex cases such fusiform vertebrobasilar aneurysms, the procedural morbidity and mortality after endovascular treatment has been reported at 29% and 57%, respectively. [44] Indeed, the role of microneurosurgery in many vertebrobasilar aneurysm cases is still essential. Not only because of the technical means, but also because of the reality that many people in the world cannot access high-priced endovascular procedures. In order to maintain a high level performance in microneurosurgery, it should be done in high volume centers. Therefore, centralization of the service is the best strategy to achieve acceptable outcomes.

CONCLUSION

We have described our experiences of utilizing a modified presigmoid approach to treat vertebrobasilar aneurysms by a single very experienced neurosurgeon. The overall results are still acceptable when accounting for the complex location and configuration of the aneurysm. Unfavorable patient's outcomes was related to the poor pre-admission Hunt-Hess grade, aneurysm morphology, and aneurysm size.

Special thanks to Jörgen Svensson, MA from Helsinki University Language Center

ACCEPTED MANUSCRIPT

References

1. Grossi PM, Nonaka Y, Watanabe K, Fukushima T. The history of the combined supra- and infratentorial approach to the petroclival region. *Neurosurgical focus.* Aug 2012;33(2):E8.

2. Hitselberger WE, House WF. A combined approach to the cerebellopontine angle. A suboccipital-petrosal approach. *Archives of otolaryngology.* Sep 1966;84(3):267-285.

3. Morrison AW, King TT. Experiences with a translabyrinthine-transtentorial approach to the cerebellopontine angle. Technical note. *Journal of neurosurgery.* Mar 1973;38(3):382-390.

4. Hakuba A, Nishimura S, Inoue Y. Transpetrosal-transtentorial approach and its application in the therapy of retrochiasmatic craniopharyngiomas. *Surgical neurology.* Oct 1985;24(4):405-415.

5. Hakuba A, Nishimura S, Jang BJ. A combined retroauricular and preauricular transpetrosal-transtentorial approach to clivus meningiomas. *Surgical neurology.* Aug 1988;30(2):108-116.

6. Day JD, Fukushima T, Giannotta SL. Cranial base approaches to posterior circulation aneurysms. *Journal of neurosurgery.* Oct 1997;87(4):544-554.

7. Kunihiro N, Goto T, Ishibashi K, Ohata K. Surgical outcomes of the minimum anterior and posterior combined transpetrosal approach for resection of retrochiasmatic craniopharyngiomas with complicated conditions. *Journal of neurosurgery.* Jan 2014;120(1):1-11.

8. Lawton MT, Daspit CP, Spetzler RF. Presigmoid approaches to skull base lesions. *Advances and technical standards in neurosurgery.* 1997;23:189-204.

9. Sincoff EH, McMenomey SO, Delashaw JB, Jr. Posterior transpetrosal approach: less is more. *Neurosurgery.* Feb 2007;60(2 Suppl 1):ONS53-58; discussion ONS58-59.

10. Gross BA, Tavanaiepour D, Du R, Al-Mefty O, Dunn IF. Petrosal approaches to posterior circulation aneurysms. *Neurosurgical focus.* Aug 2012;33(2):E9.

11. Spetzler RF, Daspit CP, Pappas CT. The combined supra- and infratentorial approach for lesions of the petrous and clival regions: experience with 46 cases. *Journal of neurosurgery.* Apr 1992;76(4):588-599.

12. Gross BA, Tavanaiepour D, Du R, Al-Mefty O, Dunn IF. Evolution of the posterior petrosal approach. *Neurosurgical focus.* Aug 2012;33(2):E7.

13. Molyneux A, Kerr R, Stratton I, et al. International Subarachnoid Aneurysm Trial (ISAT) of neurosurgical clipping versus endovascular coiling in 2143 patients with ruptured intracranial aneurysms: a randomised trial. *Lancet.* Oct 26 2002;360(9342):1267-1274.

14. Wiebers DO, Whisnant JP, Huston J, 3rd, et al. Unruptured intracranial aneurysms: natural history, clinical outcome, and risks of surgical and endovascular treatment. *Lancet.* Jul 12 2003;362(9378):103-110.

ACCEPTED MANUSCRIPT

ACCEPTED MANUSCRIPT

15. Smith GA, Dagostino P, Maltenfort MG, Dumont AS, Ratliff JK. Geographic variation and regional trends in adoption of endovascular techniques for cerebral aneurysms. *Journal of neurosurgery*. Jun 2011;114(6):1768-1777.

16. Wakhloo AK, Mandell J, Gounis MJ, et al. Stent-assisted reconstructive endovascular repair of cranial fusiform atherosclerotic and dissecting aneurysms: long-term clinical and angiographic follow-up. *Stroke; a journal of cerebral circulation*. Dec 2008;39(12):3288-3296.

17. Sekhar LN, Tariq F, Morton RP, et al. Basilar tip aneurysms: a microsurgical and endovascular contemporary series of 100 patients. *Neurosurgery*. Feb 2013;72(2):284-298; discussion 298-289.

18. Siddiqui AH, Kan P, Abla AA, Hopkins LN, Levy EI. Complications after treatment with pipeline embolization for giant distal intracranial aneurysms with or without coil embolization. *Neurosurgery*. Aug 2012;71(2):E509-513; discussion E513.

19. Pandey AS, Koebbe C, Rosenwasser RH, Veznedaroglu E. Endovascular coil embolization of ruptured and unruptured posterior circulation aneurysms: review of a 10-year experience. *Neurosurgery*. Apr 2007;60(4):626-636; discussion 636-627.

20. Hunt WE, Hess RM. Surgical risk as related to time of intervention in the repair of intracranial aneurysms. *Journal of neurosurgery*. Jan 1968;28(1):14-20.

21. Martin Lehecka AL, Juha Hernesniemi. Common Approaches. In: Martin Lehecka AL, Juha Hernesniemi, ed. *Helsinki Microneurosurgery*. Vol 1. Helsinki: Druckerei Hohl GmbH & Co. KG; 2011:160 - 169.

22. Gonzalez LF, Amin-Hanjani S, Bambakidis NC, Spetzler RF. Skull base approaches to the basilar artery. *Neurosurgical focus*. Aug 15 2005;19(2):E3.

23. Drake CG, Peerless, S.J., Hernesniemi, J.A. *Surgery of Vertebrobasilar Aneurysm London, Ontario Experience on 1767 Patients*. New York: Springer-Verlag; 1996.

24. Randell T, Niemela M, Kytta J, et al. Principles of neuroanesthesia in aneurysmal subarachnoid hemorrhage: The Helsinki experience. *Surgical neurology*. Oct 2006;66(4):382-388; discussion 388.

25. Nader Sanai AM, Robert F. Spetzler. Surgical Management of Midbasilar and Lower Basilar Aneurysms. In: Quiñones-Hinojosa A, ed. *Schmidek*. Vol 1. 6th ed. Philadelphia: Saunders; 2012:925 - 936.

26. Abdel Aziz KM. Basilar Bifurcation Aneurysms: Strategies for surgical approach selection. *Neurosurgery Quarterly*. 2007;17(2):101-112.

27. Villavicencio AT, Gray L, Leveque JC, Fukushima T, Kureshi S, Friedman AH. Utility of three-dimensional computed tomographic angiography for assessment of relationships between the vertebrobasilar system and the cranial base. *Neurosurgery*. Feb 2001;48(2):318-326; discussion 326-317.

28. Bendok BR, Getch CC, Parkinson R, O'Shaughnessy BA, Batjer HH. Extended lateral transsylvian approach for basilar bifurcation aneurysms. *Neurosurgery*. Jul 2004;55(1):174-178; discussion 178.

29. Dolenc VV, Skrap M, Sustersic J, Skrbec M, Morina A. A transcavernous-transsellar approach to the basilar tip aneurysms. *Br J Neurosurg*. 1987;1(2):251-259.

ACCEPTED MANUSCRIPT

30. Figueiredo EG, Paiva WS, Gomes M, Castro Flores JA, Wen HT, Teixeira MJ. Modified abbreviated transcavernous approach to basilar artery aneurysms--case report. *Surgical neurology*. Jan 2009;71(1):25-29.

31. Gonzalez LF, Crawford NR, Horgan MA, Deshmukh P, Zabramski JM, Spetzler RF. Working area and angle of attack in three cranial base approaches: pterional, orbitozygomatic, and maxillary extension of the orbitozygomatic approach. *Neurosurgery*. Mar 2002;50(3):550-555; discussion 555-557.

32. Bendok BR, Rahme RJ, Aoun SG, et al. Enhancement of the subtemporal approach by partial posterosuperior petrosectomy with hearing preservation. *Neurosurgery*. Jun 2014;10 Suppl 2:191-199; discussion 199.

33. Hernesniemi J, Ishii K, Niemela M, Kivipelto L, Fujiki M, Shen H. Subtemporal approach to basilar bifurcation aneurysms: advanced technique and clinical experience. *Acta neurochirurgica. Supplement.* 2005;94:31-38.

34. Kawase T, Toya S, Shiobara R, Mine T. Transpetrosal approach for aneurysms of the lower basilar artery. *Journal of neurosurgery*. Dec 1985;63(6):857-861.

35. McLaughlin N, Martin NA. Extended subtemporal transtentorial approach to the anterior incisural space and upper clival region: experience with posterior circulation aneurysms. *Neurosurgery*. Mar 2014;10 Suppl 1:15-23; discussion 23-14.

36. Drake CG. The surgical treatment of aneurysms of the basilar artery. *Journal of neurosurgery*. Oct 1968;29(4):436-446.

37. Aziz KM, van Loveren HR, Tew JM, Jr., Chicoine MR. The Kawase approach to retrosellar and upper clival basilar aneurysms. *Neurosurgery*. Jun 1999;44(6):1225-1234; discussion 1234-1226.

38. Hafez A, Nader R, Al-Mefty O. Preservation of the superior petrosal sinus during the petrosal approach. *Journal of neurosurgery*. May 2011;114(5):1294-1298.

39. Kivisaari RP, Porras M, Ohman J, Siironen J, Ishii K, Hernesniemi J. Routine cerebral angiography after surgery for saccular aneurysms: is it worth it? *Neurosurgery*. Nov 2004;55(5):1015-1024.

40. Carneiro A, Rane N, Kuker W, Cellerini M, Corkill R, Byrne JV. Volume changes of extremely large and giant intracranial aneurysms after treatment with flow diverter stents. *Neuroradiology*. Jan 2014;56(1):51-58.

41. Kulcsar Z, Ernemann U, Wetzel SG, et al. High-profile flow diverter (silk) implantation in the basilar artery: efficacy in the treatment of aneurysms and the role of the perforators. *Stroke; a journal of cerebral circulation.* Aug 2010;41(8):1690-1696.

42. Nagashima H, Kobayashi S, Tanaka Y, Hongo K. Endovascular therapy versus surgical clipping for basilar artery bifurcation aneurysm: retrospective analysis of 117 cases. *Journal of clinical neuroscience : official journal of the Neurosurgical Society of Australasia.* Jun 2004;11(5):475-479.

43. Toth G, Bain M, Hussain MS, et al. Posterior circulation flow diversion: a single-center experience and literature review. *Journal of neurointerventional surgery*. Jul 1 2014.

44. Siddiqui AH, Abla AA, Kan P, et al. Panacea or problem: flow diverters in the treatment of symptomatic large or giant fusiform vertebrobasilar aneurysms. *Journal of neurosurgery*. Jun 2012;116(6):1258-1266.

ACCEPTED MANUSCRIPT

ACCEPTED MANUSCRIPT

Table 1. Clinical characteristic of 33 procedures utilizing presigmoid approach in our series

**Description**	**Value**
Mean age (years)	47.9 (28 – 68)
Sex	
Male	16 (49%)
Female	17 (51%)
Pre-operative Hunt-Hess PreOp	
Grade 0	14 (43%)
Grade 1	6 (18%)
Grade 2	3 (9%)
Grade 3	6 (18%)
Grade 4	3 (9%)
Grade 5	1 (3%)
Operation duration (skin to skin) (minutes)	189 (70 – 257)
Approach side	
Right side	24(73%)
Left side	9 (27%)
Post-operative Outcome	
Favorable	21 (64%)
Unfavorable	7 (21%)
Dead	5 (15%)

ACCEPTED MANUSCRIPT

ACCEPTED MANUSCRIPT

Table 2. Patient's general outcome according to the clinical and radiologic factors

Description	Favorable	Unfavorable	Dead	Total
Age (n = 33)	47.5 (30 – 68)	47.6 (28 – 62)	50 (36 – 65)	
Hunt-Hess (n = 33)				
Good grade (0 – 2)	17 (74%)	5 (22%)	1 (4%)	23 (68%)
Poor grade (3 – 5)	4 (36%)	3 (28%)	4 (36%)	10 (32%)
Aneurysm Morphology (n = 34)				
Saccular	19 (66%)	7 (24%)	3 (10%)	29 (85%)
Fusiform	2 (50%)	1 (25%)	1 (25%)	1 (3%)
Dissecting	0 (0%)	0 (0%)	1 (100%)	4 (12%)
Neck Size (n = 30)	4.3 (1.7 – 12.8)	8.2 (2.4 – 20.4)	5.4 (4.8 – 6.1)	
Mean Dome size (n = 34)	8.9 (2.6 – 38.4)	14.8 (5.5 – 28.6)	5.8 (2.6 – 7.3)	
Very small (< 3mm)	4 (80%)	0 (0%)	1 (20%)	5 (15%)
Small (3 – 7 mm)	9 (75%)	1 (8%)	2 (17%)	12 (35%)
Medium (7 – 14 mm)	5 (50%)	3 (30%)	2 (20%)	10 (29%)
Large (14 – 24 mm)	2 (50%)	2 (50%)	0 (0%)	4 (12%)
Giant (> 24 mm)	1 (33%)	2 (67%)		3 (9%)
Distance from the tip of posterior clinoid process to the proximal aneurysm distance	13 (0 – 26.6)	12 (5.6 – 26.4)	8.6 (4.4 – 15.1)	
Aneurysm Origin (n = 34)				
Basilar bifurcation	6 (55%)	3 (27%)	2 (18%)	11 (32%)
Basilar-SCA	2 (50%)	2 (50%)	0 (0%)	4 (12%)
Basilar Trunk	9 (64%)	2 (14%)	3 (21%)	14 (41%)
Basilar-AICA	2 (100%)	0 (0%)	0 (0%)	2 (6%)
VBJ	1 (50%)	1 (50%)	0 (0%)	2 (6%)
Distal vertebral	1 (100%)	0 (0%)	0 (0%)	1 (3%)
Dome Projection (n=34)				
Superior	3 (43%)	2 (29%)	2 (29%)	7 (20%)
Posterior	4 (57%)	3 (43%)	0 (0%)	7 (20%)
Anterior	3 (50%)	2 (33%)	1 (17%)	6 (18%)
Right	7 (70%)	1 (10%)	2 (20%)	10 (30%)
Left	4 (100%)	0 (0%)	0 (0%)	4 (12%)

ACCEPTED MANUSCRIPT

ACCEPTED MANUSCRIPT

Table 3. The aneurysm occlusion rate characteristics

Description	Complete	Near Complete	Incomplete
Aneurysm presentation			
Unruptured	6 (43%)	3 (21%)	5 (36%)
Ruptured	17 (85%)	1 (5%)	2 (10%)
Aneurysm Morphology			
Saccular	21 (72%)	4 (14%)	4 (14%)
Fusiform	1 (25%)	0 (0%)	3 (75%)
Dissecting	1 (100%)	0 (0%)	0 (0%)
PCP to aneurysm distance	11.4 (0 – 26.6)	12.9 (8.2 – 16.8)	15.4 (0 -26.4)
Neck size§	5.0 (1.7 – 20.4)	7.1 (4.7 – 12.8)	5.9 (2.6 – 9.5)
Aneurysm Origin			
Basilar bifurcation	7 (64%)	2 (18%)	2 (18%)
Basilar-SCA	4 (100%)	0 (0%)	0 (0%)
Basilar Trunk	8 (57%)	2 (14%)	4 (29%)
Basilar-AICA	2 (100%)	0 (0%)	0 (0%)
VBJ	1 (50%)	0 (0%)	1 (50%)
Distal vertebral	1 (100%)	0 (0%)	0 (0%)
Dome Size	7.8 (2.6 – 28.6)	9.9 (7 – 13.4)	16.7 (6.4 – 38.4)
Very small (< 3mm)	5 (100%)	0 (0%)	0 (0%)
Small (3 – 7 mm)	11 (92%)	0 (0%)	1 (8%)
Medium (7 – 14mm)	4 (40%)	4 (40%)	2 (20%)
Large (14 – 24 mm)	2 (50%)	0 (0%)	2 (50%)
Giant (> 24 mm)	1 (33%)	0 (0%)	2 (67%)
Dome Projection			
Superior	6 (86%)	0 (0%)	1 (14%)
Posterior	4 (57%)	1 (14%)	2 (28%)
Anterior	3 (50%)	0 (0%)	3 (50%)
Right	7 (70%)	2 (20%)	1 (10%)
Left	3 (75%)	1 (25%)	0 (0%)

§ = fusiform aneurysms were not included in this analysis.

ACCEPTED MANUSCRIPT

Table 4. The multi-authors review of presigmoid approach experience for vertebrobasilar aneurysm
(Adapted from Gross, et al[10])

Authors/year	Cases No	CSF leak	Permanent CN deficit	Mortality
Salomon / 1991	1	0	0	0
Spetzler / 1992	2	1 (50%)	0	0
King / 1993	4	0	1 (25%)	0
Oritigano / 1993	2	0	1(50%)	0
Collice / 1997	5	0	1(20%)	1 (20%)
Day / 1997	1	0	0	0
Arai / 1998	1	0	1(100%)	0
Motoyama / 2000	1	0	0	0
Seifert / 2003	15	6 (40%)	1 (7%)	1 (7%)
Evans / 2004	1	0	0	1 (100%)
Hamel / 2005	1	1 (100%)	0	0
Bambakidis / 2009	1	1	0	0
Higa / 2009	1	0	0	0
Hernesniemi / 2014	33	2 (6%)	5 (16%)	5 (15%)

ACCEPTED MANUSCRIPT

ACCEPTED MANUSCRIPT

Figure Legends

Figure 1. Steps of the presigmoid approach to clip a basilar tip aneurysm. A. Skin incision marking. B. The exposure after the craniotomy and temporal bone drilling. C. Opening the duramater and cutting the SPS. D. The tentorium is cut toward its edge with preservation of the fourth nerve E. Aneurysm exposure with its surrounding neurovascular anatomy. F. Proximal temporary clip of Basilar artery G.Distal temporary clip of left Pcom. H. Neck clip application. I. Definitive tandem clip application.
(SS = sigmoid sinus ; SDA = sinodural angle ; SPS : Superior Petrosal Sinus ; III = occulomotor nerve ; IV = trochlear nerve ; An = aneurysm ; Lt P1 : left posterior cerebral artery segment 1 ; Lt P2 : left posterior cerebral artery segment 2 ; Lt SCA : left superior cerebellar artery ; Lt PCom = left posterior communicating artery ; BA = Basilar artery; TLD = Temporal lobe dura)

Figure 2. The postoperative CT bone reconstruction revealed the area of craniotomy and temporal bone drilling. The minimal bone drilling allows the surgery to be faster and safer (yellow arrows). Red arrow : the inner ear structures that left intact.

Figure 3. The location of basilar artery aneurysms those were clipped using our modified presigmoid approach.

Figure 4. A1-A2. Shows the pre and post operative result of complete clipping of a large proximal basilar trunk aneurysm ; B1-B2. Shows the pre- and postoperative result of the neck residual of the medium-sized basilar tip aneurysm, some part of neck remnant is still seen on the post operative angiogram (yellow arrow). C1-C2. Shows the pre- and post operative image of both Basilar-SCA and fenestrated vertebrobasilar junction aneurysm. The Basilar – SCA aneurysm is completely clipped, but there is a small neck and fundus remnant of the fenestrated vertebrobasilar junction aneurysm (yellow arrow)

ACCEPTED MANUSCRIPT

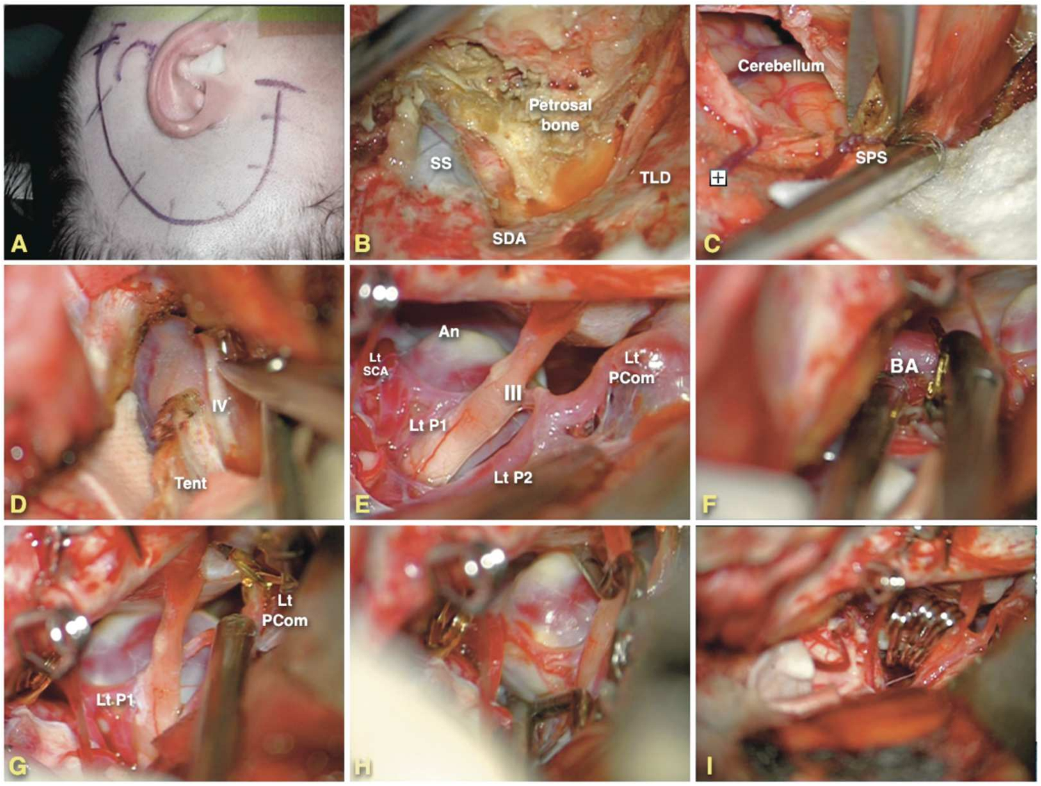

ACCEPTED MANUSCRIPT

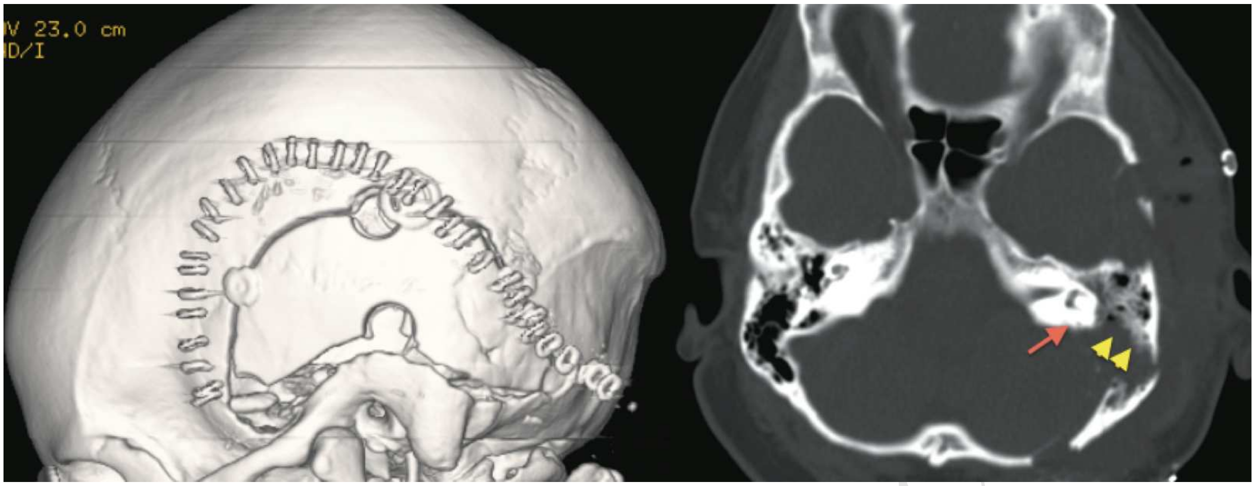

ACCEPTED MANUSCRIPT

ACCEPTED MANUSCRIPT

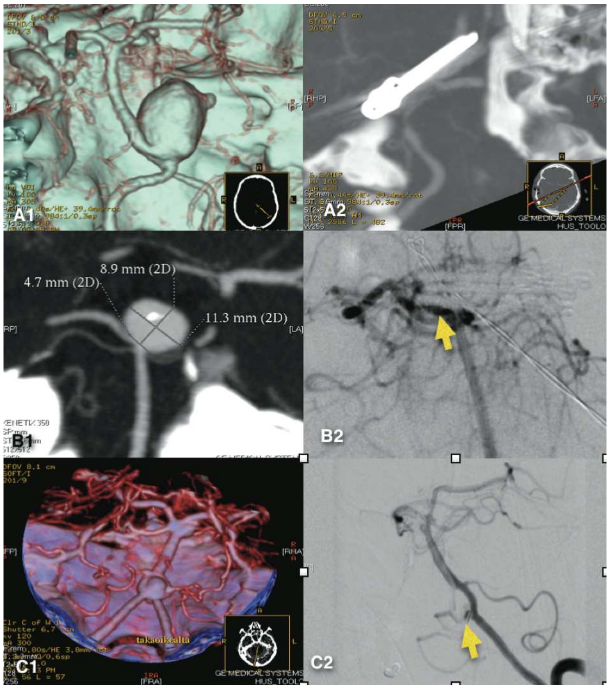

ACCEPTED MANUSCRIPT

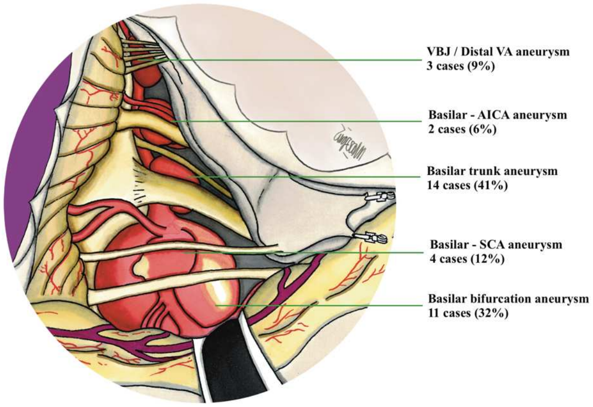

ACCEPTED MANUSCRIPT

ACCEPTED MANUSCRIPT

Key Highlights from Manuscript

- One of the biggest series of a single surgeon experience utilizing presigmoid approach for vertebrobasilar aneurysm clipping from 1998 – 2014 was studied.
- Simplified presigmoid surgical technique modified by the senior author (J.H.) was described
- The most common site aneurysms were basilar trunk (42%). Other aneurysm sites in this study were low-lying basilar bifurcation, Basilar-SCA, Basilar-AICA, Vertebrobasilar junction, and distal vertebral artery.
- Postoperative clinical and radiological outcome were discussed in this manuscript.
- Unfavorable patient's outcomes were related to the poor pre-admission Hunt-Hess grade, aneurysm morphology, and aneurysm size.

ACCEPTED MANUSCRIPT

ACCEPTED MANUSCRIPT

Abbreviations :

AICA : Anterior Inferior Cerebellar Artery
BA : Basilar Artery
CSF : Cerebro-Spinal Fluid
CTA : Computed Tomography Angiography
DSA : Digital Substraction Angiography
ISAT : International Subarachnoid Aneurysm Trial
ISUIA : International Study of Unruptured Intracranial Aneurysms
GOS : Glasgow Outcome Score
PCP : Posterior Clinoid Process
P1 : First segment of Posterior Cerebral Artery
P2 : Second segment of Posterior Cerebral Artery
PCom : Posterior Communicating Artery
SCA : Superior Cerebellar Artery
SDA : Sinodural Angle
SPS : Superior Petrosal Sinus
Tent : Tentorium
TLD : Temporal Lobe Duramater
VA : Vertebral Artery
VBJ : Vertebro-Basilar Junction

ACCEPTED MANUSCRIPT
