# Supplementary material for: Management strategies and outcomes of basilar trunk aneurysms: a systematic review and meta-analysis
Source: Langenbecks Arch Surg. 2026 Jan 20;411(1):71. doi: 10.1007/s00423-025-03959-3 (PMC12852281; doi:10.1007/s00423-025-03959-3)
Supplement: Supplementary file 1 — Supplementary file1 (ZIP 55327 KB) [file 423_2025_3959_MOESM1_ESM.zip › literature.zip!/meta/uda 2001.pdf]

J Neurosurg 95:624–632, 2001

Endovascular treatment of basilar artery trunk aneurysms with Guglielmi detachable coils: clinical experience with 41 aneurysms in 39 patients

KEN UDA, M.D., YUICHI MURAYAMA, M.D., Y. PIERRE GOBIN, M.D., GARY R. DUCKWILER, M.D., AND FERNANDO VIÑUELA, M.D.

Division of Interventional Neuroradiology, University of California at Los Angeles School of Medicine, California

Object. The authors present a retrospective analysis of their clinical experience in the endovascular treatment of basilar artery (BA) trunk aneurysms with Guglielmi detachable coils (GDCs).

Methods. Between April 1990 and June 1999, 41 BA trunk aneurysms were treated in 39 patients by inserting GDCs. Twenty-seven patients presented with subarachnoid hemorrhage, six had intracranial mass effect, and in six patients the aneurysms were found incidentally. Eighteen lesions were BA trunk aneurysms, 13 were BA–superior cerebellar artery aneurysms, four were BA–anterior inferior cerebellar artery aneurysms, and six were vertebrobasilar junction aneurysms. Thirty-five patients (89.7%) had excellent or good clinical outcomes; procedural morbidity and mortality rates were 2.6% each. Thirty-six aneurysms were selectively occluded while preserving the parent artery, and in five cases the parent artery was occluded along with the aneurysm. Immediate angiographic studies revealed complete or nearly complete occlusion in 35 aneurysms (85.4%). Follow-up angiograms were obtained in 29 patients with 31 aneurysms; the mean follow-up period was 17 months. No recanalization was observed in the eight completely occluded aneurysms. In 19 lesions with small neck remnants, seven (36.8%) had further thrombosis, three (15.8%) remained anatomically unchanged, and nine (47.3%) had recanalization caused by coil compaction. In one patient (2.6%) the aneurysm rebled 8 years after the initial embolization.

Conclusions. In this clinical series the authors show that the GDC placement procedure is valuable in the therapeutic management of BA trunk aneurysms. The endovascular catheterization of these lesions tends to be relatively simple, in contrast with more complex neurosurgical approaches. Endosaccular obliteration of these aneurysms also decreases the possibility of unwanted occlusion of perforating arteries to the brainstem.

KEY WORDS • basilar artery • aneurysm • basilar artery trunk aneurysm • embolization • Guglielmi detachable coil

BASILAR artery trunk aneurysms are located in a sort of "no man's land."[46] Because of their deep location in front of the brainstem and their intimate relationship to perforating vessels and cranial nerves, the morbidity and mortality rates associated with their exposure during surgical procedures used to be daunting.[43] Recent advances in neuroanesthesiology and neuroprotection modalities, neurosurgical instrumentation, and the development of innovative skull base approaches for exposure of the ventral brainstem area have led to significantly better surgical results in patients with BA trunk aneurysms.[1,7,11,22,27,32,35] Nevertheless, patients presenting in poor clinical condition, those of advanced age, or those with large or giant lesions that are surgically difficult to treat are considered poor candidates for microsurgical intervention.[17,18] In selected neurovascular treatment centers, endovascular procedures have been accepted as an alternative form of therapy.[2,5,15,25,40,42] Thus, GDCs have been incorporated into the neuroendovascular armamentarium for the treatment of aneurysms.[14] The safety and efficacy of this endovascular methodology are still being defined, as shown by the increasing number of reports related to this procedure in the neurosurgical literature.[2,4,5,12–15,25,30,40,43,51,52] We present a series of 39 patients with 41 BA trunk aneurysms that were treated with GDCs at the UCLA Medical Center.

Clinical Material and Methods

Patient Population

Between April 1990 and June 1999, 41 BA trunk aneurysms were treated in 39 patients by using GDC technology at the UCLA Medical Center. There were 29 women and 10 men, whose ages ranged from 28 to 76 years (mean 51 years). The locations of the 41 aneurysms were as follows: 18 pure BA trunk aneurysms, including nine lesions in a BA fenestration (Figs. 1 and 2); 13 BA–SCA aneurysms; four BA–AICA aneurysms; and six VBJ aneurysms (Fig. 3). Basilar artery bifurcation and VA aneurysms were excluded from this analysis.

Abbreviations used in this paper: AICA = anterior inferior cerebellar artery; BA = basilar artery; GDC = Guglielmi detachable coil; PCoA = posterior communicating artery; SAH = subarachnoid hemorrhage; SCA = superior cerebellar artery; UCLA = University of California at Los Angeles; VA = vertebral artery; VBJ = vertebrobasilar junction.

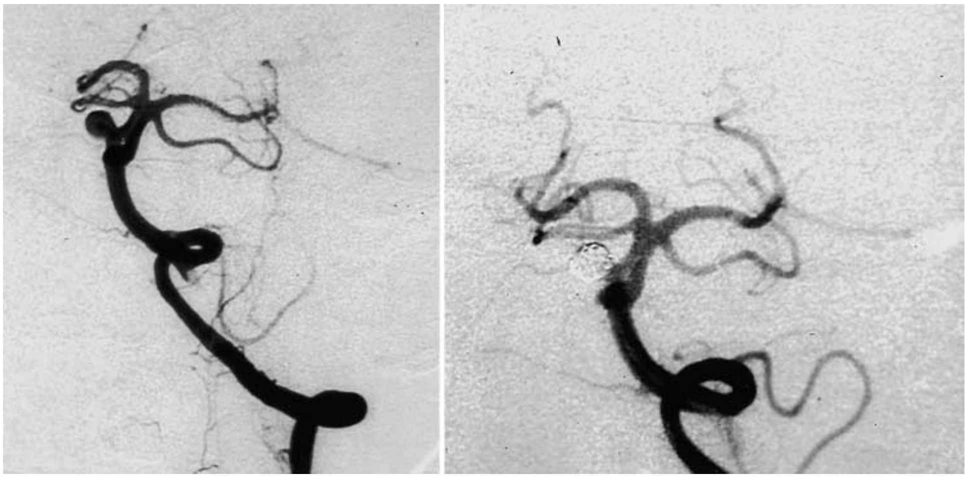

FIG. 1. Case 3. *Left:* Left VA angiogram demonstrating a ruptured BA trunk aneurysm arising from a limb of the BA fenestration in a 75-year-old woman presenting with Hunt and Hess Grade II SAH. *Right:* Postembolization angiogram demonstrating a nearly complete aneurysm occlusion; the patient remained neurologically intact.

Clinical Presentation

As shown in Table 1, 27 patients presented with SAH and six suffered from intracranial mass effect. The aneurysms were incidentally discovered in six patients, they were associated with arteriovenous malformations in two, and were associated with coexisting aneurysms with mass effect in two.

Sixteen patients with SAH were classified according to the Hunt and Hess scale,[20] three patients were Grade I, four were Grade II, eight were Grade III, and one was Grade IV. Eleven patients were treated during the nonacute phase of SAH (defined as 2 weeks or more posthemorrhage).

Anatomical Characteristics of the Aneurysms

Based on angiographic data obtained at the time of embolization, the aneurysms were classified by their size and neck width. Twenty-eight lesions were small (diameter $<$ 11 mm), seven were large (11–25 mm), and six were giant ($>$ 25 mm). Fifteen aneurysms had small necks ($<$ 4 mm), 22 had wide necks ($\geq$ 4 mm), and four were fusiform. In three patients with giant aneurysms, an intraaneurysmal thrombus was identified on magnetic resonance images.

Indications for Endovascular Treatment

Therapeutic alternatives were discussed among the members of the neurosurgical and neurointerventional teams. The indications for endovascular treatment included the following: 1) anticipated surgical difficulty because of aneurysm size or location in 26 patients (66.7%); 2) attempted surgical clipping in eight patients (20.5%); 3) poor neurological status in one patient (2.6%); and 4) poor medical condition in four patients (10.2%).

The GDC Procedure

The details of the GDC procedure have already been published in the literature.[14,15,37,52] In our series, the same basic method was applied in the GDC embolization of BA trunk aneurysms. In the last 2 years, we began to use softer and smaller GDCs, which allowed denser packing of the aneurysm, particularly of its neck and inflow zone. In three patients with wide-necked aneurysms, the balloon-assisted method[28] and three-dimensional GDCs were used. When selective occlusion of the aneurysm sac was not possible, concomitant occlusion of the aneurysm and the parent artery was performed. This procedure was performed only after the patient tolerated temporary balloon test occlusion of the parent artery.[12]

Anatomical and Clinical Results

All aneurysms were evaluated angiographically immediately after treatment. Follow-up angiograms were obtained 6 months postembolization if the lesions had been completely or nearly completely occluded. A 3-month angiographic follow-up examination was recommended in patients with incompletely embolized aneurysms. In some patients follow-up angiographic studies were performed 12 months or more postembolization.

The anatomical results were classified as follows: complete occlusion, neck remnant, and incomplete occlusion.[37] Complete occlusion designated aneurysms in which no residual neck was detected on any angiographic projection, neck remnant referred to lesions with residual filling of part of the neck of the aneurysm, and incomplete occlusion was defined as the presence of contrast agent filling the body and/or dome of the aneurysm because of incomplete GDC packing. The same classifications were used to evaluate follow-up angiograms.

The changes observed on follow-up angiograms were classified as further thrombosis, which was defined as a decrease in the amount of contrast agent filling the aneurysm; unchanged, defined as a similar degree of aneurysm occlusion in multiple projections; and recanalization, defined as an increase in the amount of contrast material filling the aneurysm compared with the immediate postembolization angiographic results.[16]

Clinical follow up was obtained at office appointments or by telephone interviews. Clinical outcome was graded

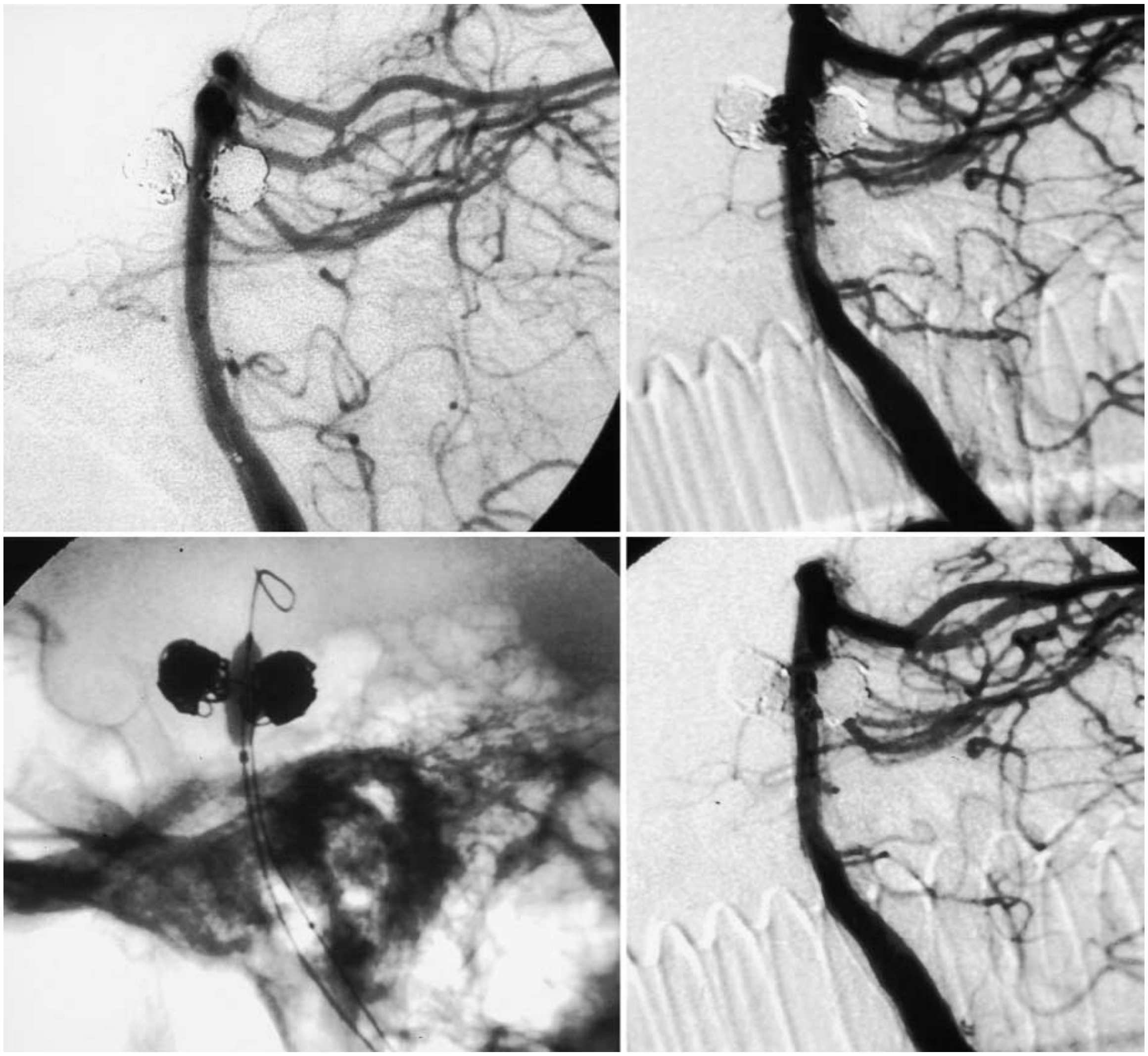

FIG. 2. Case 5. *Upper Left:* Postembolization VA angiogram demonstrating successful occlusion of two small, saccular, "kissing" aneurysms on the BA trunk. *Upper Right:* Follow-up angiogram obtained 29 months post-GDC embolization revealing recanalization of the neck of the ventral aneurysm. *Lower Left:* Angiogram demonstrating microballoon catheter being advanced to the neck of the aneurysm and inflated during deposition of additional GDCs. *Lower Right:* Postembolization VA angiogram in which the balloon-assisted method was used, demonstrating nearly complete occlusion of the aneurysm with preservation of the lumen of the BA.

according to a modified Glasgow Outcome Scale,[21] as follows: excellent (neurologically intact); good (mild hemiparesis, cranial nerve palsy, or other deficit that does not interfere with daily functioning or work); fair (significant hemiparesis, aphasia, confusion, or other deficit that interferes with daily activities or prevents a return to work); and poor (coma or severe neurological deficit rendering the patient dependent on family or nursing staff).

Results

Angiographic Results

Thirty-six aneurysms were selectively occluded using GDCs, and in five patients (Cases 4, 15, 35, 36, and 37), concomitant occlusions of the aneurysm and parent artery were performed. Three of these five lesions were VBJ aneurysms. A giant BA trunk aneurysm (Case 4) and a giant VBJ aneurysm (Case 37) were treated with bilateral VA occlusions in the patients' necks.

Thirteen aneurysms (31.7%) were completely occluded, 22 (53.7%) had a neck remnant, and six (14.6%) were incompletely occluded. Five (35.7%) of 14 small aneurysms with small necks were completely obliterated, whereas in eight (57.1%) of 14 there was a neck remnant. Seven (50%) of 14 small aneurysms with wide necks were completely occluded, whereas in six (42.9%) of these lesions there was a neck remnant. A complete occlusion was achieved in one (14.3%) of seven large aneurysms, whereas in none of the giant aneurysms was complete occlusion achieved.

Follow-up angiograms were obtained in 29 patients with a total of 31 aneurysms, and the mean angiographic follow-up period was 17 months (range 3–98 months).

Long-term follow-up angiograms could not be collected in 10 patients: one had died, two had a poor clinical outcome, one refused cerebral angiography, and six have since been scheduled for follow-up testing.

No recanalization was observed in the eight completely occluded aneurysms. In the 19 aneurysms with small neck remnants, seven (36.8%) demonstrated further thrombosis, three (15.8%) remained anatomically unchanged, and in nine (47.3%) we observed recanalization caused by coil compaction into the body of the aneurysm. In the three incompletely occluded aneurysms with contrast agent filling in the body of the lesion, one (33.3%) treated with bilateral VA occlusion revealed further thrombosis, one (33.3%) remained anatomically unchanged, and in one (33.3%) recanalization was observed.

Repeated Embolization

In 39 patients with 41 aneurysms, 53 endovascular procedures (mean 1.3 per lesion) were performed. Thirty-three of the 41 aneurysms were successfully treated in a single session, whereas eight required multiple procedures: six lesions required two, and the other two required four endovascular interventions.

Postembolization Surgical Procedures

Postembolization surgical clipping was performed in two lesions (4.9%). One patient (Case 21) had a residual BA–SCA aneurysm, and another (Case 30) had a residual BA–AICA aneurysm; both lesions were successfully clipped.

Postembolization Hemorrhage

In one patient (Case 10) the lesion rebled 8 years after the initial endovascular treatment. This patient was a 58-year-old woman who had presented with an acute SAH in 1990. Cerebral angiographic studies revealed a large BA trunk aneurysm arising from a lower BA fenestration, for which the patient underwent embolization. Two additional endovascular procedures were performed in 1991 and 1994, and he suffered a repeated SAH in 1998. Although the fourth GDC embolization was uneventful, the patient died of pneumonia.

Procedural Morbidity and Clinical Outcome

Four technical complications were observed: one patient (Case 6) died of aneurysm perforation (2.6%), and three patients (Cases 9, 36, and 37) had ischemic complications (7.7%), including two thromboembolic and one hemodynamic events.

The patient in Case 9 had an aneurysm of a BA fenestration and another one in the right PCoA; both lesions were occluded using GDCs. After embolization, the patient experienced a right cerebral ischemic event, most likely the result of an embolus from the right PCoA aneurysm; this patient's symptoms totally resolved within 12 hours. The patient in Case 36 experienced a transient diplopia caused by clot formation at the neck of the aneurysm. The patient in Case 37 had a giant VBJ aneurysm, and he underwent endovascular treatment consisting of bilateral VA occlusion. He suffered a permanent, mild hemiparesis after embolization, although heparin was ad-

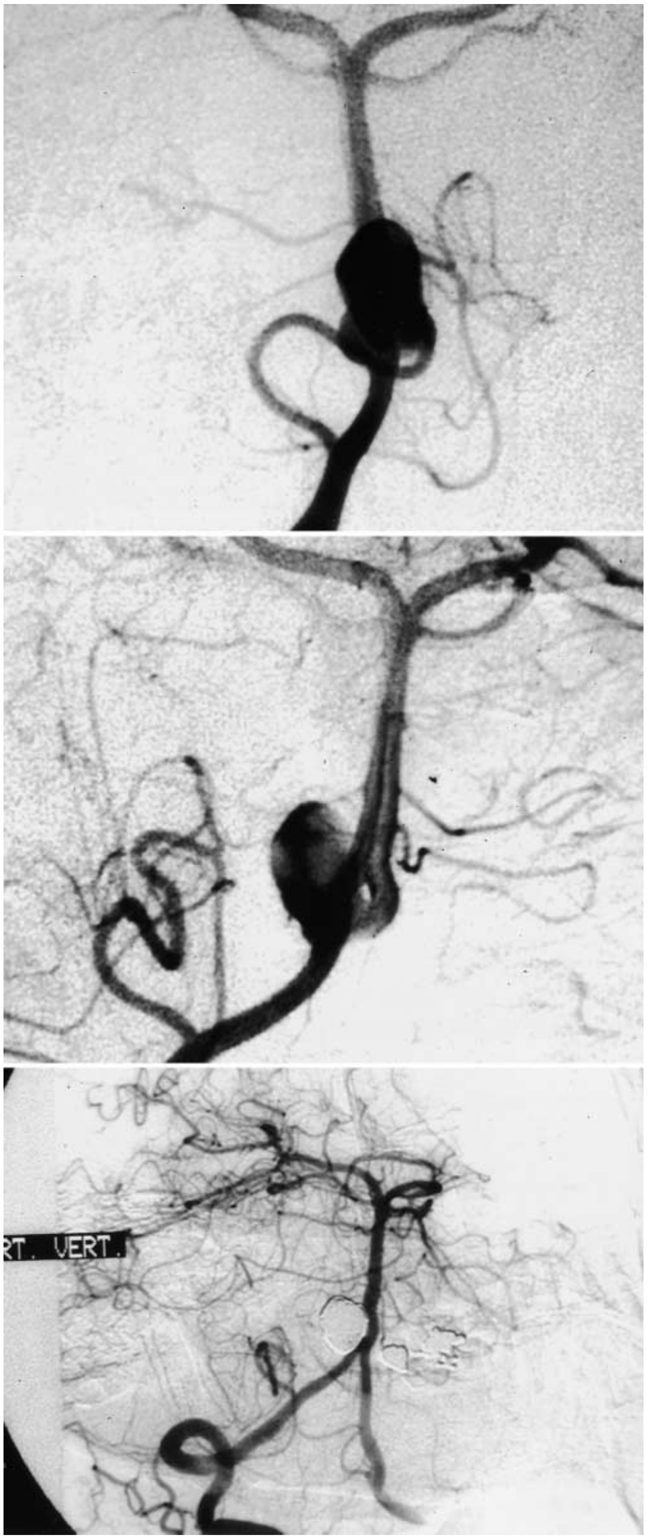

FIG. 3. Case 39. *Upper:* Anteroposterior view of a left VA angiogram demonstrating lower BA aneurysm totally superimposed on a BA. *Center:* Anteroposterior oblique view clearly demonstrating a BA trunk aneurysm arising from a lower BA fenestration. Complete occlusion of the aneurysm was achieved using the balloon-assisted method. *Lower:* Follow-up angiogram obtained 1 year postembolization demonstrating persistent aneurysm occlusion.

TABLE 1

*Clinical characteristics of 39 patients in whom 41 BA trunk aneurysms were identified**

Case No.	Age (yrs), Sex	Presentation	Lesion Characteristics: Location	Size	Neck	Indication for GDCs	Initial Results	Change on FU Angiograms†	No. of Embols	Result of Re-embol	Clinical FU (mos)	Angiographic FU (mos)	Results of Last Angiogram	Outcome
1	29, M	nonacute SAH	trunk	large	small	failed clipping	comp		1	ND	ND	ND	comp	excellent
2	63, F	incidental	trunk fen	small	small	antic surg diff	NR	unchanged	1	ND	7	7	NR	excellent
3	75, F	SAH (II)	trunk fen	small	small	antic surg diff	NR	unchanged	1	ND	13	11	NR	excellent
4	28, F	SAH (I)	trunk	giant	fusi	antic surg diff	incomp (bilat VA oc)	thrombosis	1	ND	17	14	comp	excellent
5	50, F	nonacute SAH	trunk; kissing	small	small	poor condition	NR	recanal	2	NR	29	29	NR	excellent
			trunk; kissing	small	small		NR	thrombosis	1	ND	29	29	comp	excellent
6	53, F	mass effect	trunk	giant	fusi	antic surg diff	incomp		1	ND	ND	ND	incomp	death
7	51, M	SAH (III)	trunk	small	small	antic surg diff	comp	unchanged	1	ND	14	14	comp	excellent
8	39, F	SAH (III)	trunk	small	small	antic surg diff	incomp	unchanged	1	ND	12	12	incomp	excellent
9	41, F	nonacute SAH	trunk fen	small	wide	antic surg diff	NR	thrombosis	1	ND	48	6	comp	excellent
10	58, F	SAH (II)	trunk fen	large	wide	poor condition	NR	recanal	4	NR	99	98	NR	death
11	42, F	nonacute SAH	trunk fen	small	wide	failed clipping	comp	unchanged	1	ND	55	18	comp	excellent
			trunk fen	small	small		NR	thrombosis	1	ND	55	15	comp	excellent
12	31, F	SAH (II)	trunk fen	large	wide	antic surg diff	NR	recanal	2	NR	6	3	comp	excellent
13	62, F	SAH (III)	trunk fen	small	wide	antic surg diff	NR	thrombosis	1	ND	24	24	comp	excellent
14	61, F	incidental	trunk fen	small	wide	antic surg diff	comp	unchanged	1	ND	12	12	comp	excellent
15	38, F	mass effect	trunk	GT	fusi	antic surg diff	incomp	recanal	2	NR (rt VA oc)	55	12	NR	excellent
16	52, F	nonacute SAH	trunk	small	small	antic surg diff	NR	thrombosis	1	ND	49	20	comp	excellent
17	40, F	incidental	BA–SCA	large	wide	antic surg diff	NR		1	ND	ND	ND	NR	excellent
18	42, F	incidental	BA–SCA	small	wide	antic surg diff	NR	thrombosis	1	ND	34	18	NR	excellent
19	72, F	SAH (III)	BA–SCA	small	small	poor condition	comp	unchanged	1	ND	6	6	comp	good (improved)
20	63, M	SAH (III)	BA–SCA	GT	wide	failed clipping	NR		1	ND	ND	ND	NR	fair (improved)
21‡	36, F	SAH (III)	BA–SCA	small	small	poor condition	NR	recanal	1	ND	6	6	comp	excellent
22	63, F	mass effect	BA–SCA	GT	wide	antic surg diff	NR	recanal	2	NR	26	26	NR	excellent
23	73, M	SAH (I)	BA–SCA	small	wide	antic surg diff	NR	unchanged	1	ND	6	6	NR	excellent
24	57, M	incidental	BA–SCA	small	wide	antic surg diff	comp		1	ND	ND	ND	comp	good (unchanged)
25	70, F	nonacute SAH	BA–SCA	small	wide	failed clipping	NR	recanal	2	NR	90	7	NR	excellent
26	55, F	SAH (I)	BA–SCA	small	small	antic surg diff	comp		1	ND	ND	ND	comp	excellent
27	37, F	mass effect	BA–SCA	small	small	antic surg diff	NR	thrombosis	1	ND	16	16	comp	excellent
28	65, F	SAH (III)	BA–SCA	small	wide	antic surg diff	comp	unchanged	1	ND	17	17	comp	excellent
29	68, F	nonacute SAH	BA–SCA	small	small	antic surg diff	comp	unchanged	1	ND	24	24	comp	excellent
30‡	55, F	SAH (IV)	BA–AICA	small	wide	poor grade	NR	recanal	1	ND	6	6	comp	excellent
31	42, M	SAH (II)	BA–AICA	large	wide	antic surg diff	NR	recanal	4	comp	56	29	comp	excellent
32	55, M	SAH (III)	BA–AICA	small	wide	failed clipping	comp	unchanged	1	ND	9	9	comp	good (improved)
33	41, M	incidental	BA–AICA	small	wide	antic surg diff	comp	unchanged	1	ND	14	14	comp	excellent
34	35, F	nonacute SAH	VBJ	large	fusi	failed clipping	NR	recanal	1	ND	24	12	NR	excellent
35	62, F	nonacute SAH	VBJ	small	wide	failed clipping	incomp		2	comp (rt VA oc)	1	ND	comp	death
36	39, M	nonacute SAH	VBJ	small	wide	failed clipping	comp (rt VA oc)		1	ND	30	ND	comp	excellent
37	76, M	mass effect	VBJ	giant	wide	antic surg diff	incomp (bilat VA oc)		1	ND	ND	ND	incomp	good
38	61, F	mass effect	VBJ	large	wide	antic surg diff	NR		1	ND	ND	ND	NR	good
39	53, F	nonacute SAH	VBJ	small	small	antic surg diff	comp	unchanged	1	ND	12	12	comp	excellent

* Roman numerals in parentheses denote Hunt and Hess grades. Four patients experienced complications: an aneurysm rupture in Case 6; thromboembolic events in Cases 9 and 36; and a hemodynamic event in Case 37. Abbreviations: antic surg diff = anticipated surgical difficulty; comp = complete; embol = embolization; fen = fenestration; FU = follow up; fusi = fusiform; GT = giant aneurysm with intraaneurysmal thrombus; incomp = incomplete; kissing = mirror-image BA trunk aneurysms; ND = not done; NR = neck remnant; oc = occlusion; recanal = recanalization.

† Follow-up angiograms were not obtained for the following reasons: poor medical condition (Cases 20 and 35); refusal (Case 36); and follow-up angiography planned (Cases 1, 17, 24, 26, 37, and 38).

‡ Patient underwent postembolization surgical clipping.

ministered and his blood pressure was maintained between 150 to 160 mm Hg. The mortality and permanent morbidity rates directly related to the aforementioned interventions both were 2.6%. The mean clinical follow-up period was 21 months (range 0–99 months).

Thirty-five patients (89.7%) had excellent or good clinical outcomes. Three patients died: one (Case 6) as a result of aneurysm perforation, and the other two of respiratory complications. One of the two (Case 35) had a ruptured bilobulated VBJ aneurysm that was treated at another institution. This patient developed impaired swallowing, which resulted in aspiration pneumonia, after left VA clipping. Although embolization of the aneurysm and the right VA was performed without complications, the patient died 1 month after embolization. The other patient (Case 10) died of pneumonia after suffering rerupture of the aneurysm.

Clinical Outcome of Ruptured Aneurysms

All patients with ruptured aneurysms were treated as soon as possible after admission at the UCLA Medical Center. An excellent clinical outcome was achieved in 22 (81.5%) of 27 patients with ruptured aneurysms. Twelve (75%) of 16 patients treated within 14 days after SAH had excellent clinical outcomes. In Case 19, a 72-year-old woman with a ruptured BA–SCA aneurysm (Hunt and Hess Grade III) gradually improved after successful embolization and had a good clinical outcome. In Case 32, a 55-year-old man with a ruptured BA–AICA aneurysm (Grade III) was treated successfully with the balloon-assisted procedure. His abducent nerve palsy related to a previous surgical attempt also improved. In Case 20, a 63-year-old man with a ruptured giant BA–SCA aneurysm (Grade III) underwent an unsuccessful surgical exploration of the lesion at another institution. This patient developed postsurgical right-sided hemiparesis. His outcome was just fair because of this right-sided hemiparesis, although his symptoms improved after uneventful embolization of the aneurysm.

No aneurysm rebleeding occurred during the perioperative period; however, a large BA trunk aneurysm reruptured 8 years after the initial treatment. Ten of 11 patients treated in the nonacute phase after SAH had excellent outcomes and one died (Case 35; described earlier).

Clinical Outcome of Unruptured Aneurysms

One patient (Case 6) who presented with mass effect died of aneurysm perforation during the procedure. In three of the remaining five patients who presented with mass effect (Case 15, hearing loss and gait problem; Cases 22 and 27, oculomotor nerve palsy) their symptoms resolved completely after embolization of the aneurysms. Clinical follow up in the other patients (Cases 37 and 38) was not done. The six patients whose aneurysms were incidentally discovered were treated uneventfully.

Discussion

Aneurysms of the BA have been classified into four subgroups in surgical series[42,47] as follows: 1) BA bifurcation aneurysms; 2) BA–SCA aneurysms; 3) BA trunk aneurysms; and 4) VBJ aneurysms. Basilar artery trunk lesions are relatively uncommon and comprise only 1 to 2% of all intracranial anuerysms.[32] Of 566 aneurysms treated in all areas of the brain, we included in this paper 41 (7.2%) that originated from the region of the VBJ to the region of the SCA, because there are no major technical differences among those aneurysms in regard to endovascular treatment, and they all are related to important perforating arteries to the brainstem. Basilar tip and VA aneurysms were excluded from this retrospective study.

Development of a Surgical Approach for BA Trunk Aneurysms

Surgical access to BA trunk aneurysms has been hampered by the structure of the petrous bone, which blocks the direct surgical approach to these lesions.[1,24,27,46] Drake[8] recommended a subtemporal transtentorial approach for BA trunk aneurysms located above the middle third of the clivus, and a suboccipital approach for the ones below that level. Recent advances in skull base surgery have made possible the approach to these anatomically difficult regions and have dramatically reduced the morbidity and mortality rates associated with surgery. One of these, the transoral and transfacial transclival approach,[41] offers a direct angle of visualization. A variety of modified approaches that lessen the degree of retraction of the neural structures have been reported, including the transpetrosal approach,[24] the retrolabyrinthine transsigmoidal approach,[11] and the combined supratentorial and infratentorial approach.[22,47] Intraoperative digital subtraction angiography may help to establish with certainty that aneurysm clip placement is perfect in the tight confines of the prepontine space.[33] Intraoperative temporary balloon occlusion of the BA[35] and deep hypothermia with circulatory arrest[27] have been described as methods facilitating clipping of giant BA trunk aneurysms. The modern approaches for these regional aneurysms have been successful only in the hands of a limited number of skilled neurosurgeons.

Advantages of the Endovascular Approach

In the early days of endovascular methods for the therapeutic management of BA trunk aneurysms, there was a limited role for the interventional neuroradiologist, who could contribute only with balloon occlusion of one or both VAs in cases of large or giant BA trunk aneurysms. The presence of well-developed PCoAs allowed retrograde filling of the BA and aneurysm, producing hemodynamic changes and thrombosis of giant aneurysms in a limited number of cases.[10,48]

The endovascular treatment of intracranial aneurysms with the GDC system was introduced in the clinical setting in 1991.[14] Endovascular navigation through the vertebrobasilar system tends to be simple, and safe catheterization of a BA trunk aneurysm may be performed in most cases. The presence of two well-developed VAs offers two arterial avenues through which to reach the aneurysm. In general, most BA trunk aneurysms are lateral wall lesions. The balloon-assisted procedure[28] or the placement of a microstent across the neck of a large aneurysm[26] may be more controllable than in terminal aneurysms such as BA bifurcation lesions. The ability to work within the lesion, reaching the neck of the aneurysm from inside, decreases

the chances of compromising the ostium of a perforating artery to the brainstem. These perforating arteries may be accidentally occluded by the GDC system if there is unwanted coil migration into the parent artery or clot progression in the neck of the aneurysm. In our clinical series the rate of complications related to unwanted occlusion of a perforating artery was 2.6%.

Guglielmi detachable coils are very useful for treating aneurysms arising from a BA fenestration. It is mandatory to obtain an angiographic view that demonstrates both limbs of the fenestration to perform a safe delivery of the GDC. In a limited number of patients, the balloon-assisted method may help to anchor the microcatheter in place while preserving the lumen of the parent artery.

Limitations of GDC Treatment

One limitation may be the anatomical characteristics of the VA. The VA is smaller than the internal carotid artery, its origin in the subclavian artery may be compromised by an atheromatous plaque, and the VA may have a steep tortuosity in the neck and skull base. Placement of a guiding catheter through an atheromatous VA may cause ischemic events or dissection of the artery, although in our series this complication was not seen.

Vertebrobasilar junction aneurysms originate at the union of both VAs with the BA. This unique morphological and hemodynamic situation may create a very difficult technical challenge for the interventional neuroradiologist. An aneurysm in this location is filled from both VAs and there is a relatively high incidence of recanalization. In this series, three of five VBJ aneurysms required permanent occlusion of one or both VAs. Two of three of the ischemic complications occurred in VBJ aneurysms, in which the lumen of perforating arteries to the medulla oblongata may be compromised, or, more rarely, the ostium of the anterior spinal artery.

Clinical Outcomes

Posterior circulation aneurysms have a well-known association with high morbidity rates, often with a fatal outcome.[17,18,42] In a population-based study[45] performed in Rochester, Minnesota, survival rates for posterior circulation aneurysms were considerably lower than the rates for anterior circulation aneurysms. In the international cooperative study,[23] a 31.2% mortality rate was reported in 266 vertebrobasilar aneurysms. The surgical treatment-related morbidity/mortality rate for BA trunk aneurysms was estimated to be between 11% and 36%.[27,46,47,49] We achieved excellent and good clinical results in 89.7% of our patients, with 2.6% rates for both procedural morbidity and mortality; the overall mortality rate was 7.7%. Our 5.2% treatment-related morbidity/mortality rate and the 7.7% overall mortality rate compare favorably with other surgical series.

Aneurysm perforation was documented in one (2.4%) of 41 aneurysms in this series. In a systematic review that included a MEDLINE search, we found that aneurysm perforation occurred in 30 (2.4%) of 1256 patients treated with GDCs.[4] Certainly, uncontrollable hemorrhaging during endovascular procedures results in a much greater risk of acute increased intracranial pressure and intraparenchymal extension of the bleeding. Intraoperative aneurysm rupture occurred at rates between 13% and 20% in surgical series.[17,18,42] Intraoperative vertebrobasilar artery aneurysm rupture is a greater disaster and is always difficult to handle because there is minimal space in the surgical field. The outcome in patients who suffered inadvertent intraoperative aneurysm rupture was more often poor (29%) compared with those whose aneurysm did not rupture (17%).[42]

Ischemic complications were observed in three patients (7.7%), including two thromboembolic and one hemodynamic events. Viñuela, et al.,[52] reported cerebral clot embolization in 10 (2.5%) of 403 patients with ruptured aneurysms. This complication was seen more commonly within 48 hours after the procedure, and particularly in wide-necked aneurysms with a small residual neck. The orifice of intracranial aneurysms may remain unsecured after endosaccular GDC treatment;[3,19,31,36] this phenomenon appears to be related to progressive thrombosis of the residual neck extending beyond the aneurysm into the parent artery, and migration of a fresh clot intracranially. This complication may also be observed after surgical clipping of an aneurysm. It may be observed more frequently in large aneurysms with wide, calcified necks that require multiple clips.

Morphological Evaluation

Complete isolation of the aneurysm from the circulatory system is the obvious goal of both open and endovascular surgery. In this series, 13 aneurysms (31.7%) were initially completely occluded. The final angiograms demonstrated that 25 aneurysms (61%) were completely occluded. The complete occlusion rate was estimated to be 53.8% in a systematic review.[4] Three fusiform aneurysms (excluding Case 6, in which aneurysm perforation resulted in death) were treated by embolization of aneurysms through one or both VAs; parent artery occlusion is an alternative treatment for unclippable aneurysms.[10,48] Basilar artery trunk aneurysms treated by balloon occlusion of one or both VAs were completely obliterated in 50% of cases.[10] Gobin, et al.,[12] have shown that large and giant fusiform intracranial aneurysms can be successfully treated by occlusion of the lesion and the parent artery with GDCs. In surgical series, complete aneurysm occlusion rates of vertebrobasilar artery aneurysms were reported to be between 34% and 91%.[35,42,46,48] This relatively broad range can be explained by differences in aneurysm size and morphological composition.

There are very few published reports in which the occurrence of aneurysm rests after surgical clipping is detailed.[6,9,29] In large surgical series the incidence of postsurgical rests has been reported as being approximately 4%.[9,29,32] David, et al.,[6] also reported that late angiographic follow-up review revealed two recurrent aneurysms (1.5%) of 135 lesions that had been clipped without leaving neck remnants. They suggested that it is the fragility of the vascular wall along the clip edge or microscopic rests of aneurysm neck which, when subjected to the original hemodynamic stresses, cause the aneurysm to regrow. In contrast, the natural history of neck remnants in aneurysms treated with GDCs has not been fully evaluated. Our series indicated a relatively high recanalization rate (47.3%) in the aneurysms with neck remnants. Hayaka-

wa, et al.,[16] reported long-term angiographic results of neck remnant in aneurysms managed by endovascular GDC treatment, as follows: in small aneurysms with small necks, six (50%) developed progressive thrombosis, eight (33%) were unchanged, and four (14%) showed recanalization. The recanalization rates of large and giant aneurysms with neck remnants were 87% and 90%, respectively. An accurate comparison of the anatomical results between the surgical and endovascular approaches remains difficult because angiographic follow up is not always available in the surgical literature. Recently, in a prospective randomized trial, anatomical and clinical outcome for surgical and GDC treatments were compared.[51] In posterior circulation aneurysms, the primary angiographic results were significantly better in the endovascularly treated group.

Aneurysm Hemorrhage After GDC Treatment

In one patient with an aneurysm neck remnant, the lesion rebled 8 years after the initial treatment. This patient had a large BA trunk aneurysm arising from a lower BA fenestration and refused to undergo postembolization angiographic follow up. The cerebral angiogram obtained after the second hemorrhagic episode revealed recanalization of the neck and body of the aneurysm. A fourth embolization in the early phase of recanalization could have averted this hemorrhagic complication. Overall, the 2.6% hemorrhage rate after GDC treatment in our series compares favorably with the 3.7% rebleeding rate from aneurysm neck remnants in published surgical series.[9] Tsutsumi, et al.,[50] reported that six (1.4%) of 425 patients suffered recurrent SAH even after complete obliteration of cerebral aneurysms by surgical clipping.

The GDC technology represents a promising one for endovascular therapy of cerebral aneurysms, and particularly of BA trunk aneurysms. The addition of soft GDCs, three-dimensional GDCs, balloon-assisted technology,[28] and microstents[26] has improved the final anatomical results, and the morbidity and mortality rates are low. These technical improvements, however, may not be the fundamental solution to the problems with aneurysm therapy. We need to seal the mural defect of the neck of an aneurysm by creating and supporting neo-intimal and neo-endothelial growth. Biologically active GDCs have shown faster neo-intimal and neo-endothelial proliferation and migration at the neck of experimentally induced aneurysms in swine.[38,39] Endovascular therapeutic devices, including GDCs, will undergo further modifications, which will provide a more stable and complete aneurysm occlusion, even in wide-necked lesions.[38,39,44]

Conclusions

Current GDC technology appears to be an effective and safe alternative treatment for patients with BA trunk aneurysms. We achieved excellent and good clinical results in 89.7% of our patients, with 2.6% rates for both procedural morbidity and mortality. The issue of GDC instability with concomitant aneurysm recanalization is of considerable clinical importance, however.[34] In the future, microcoils will undergo further biological modifications that will provide a faster, more stable, and more complete aneurysm occlusion, even in wide-necked lesions. Further developments in the manufacture of dedicated neurointerventional intracranial microstents will also contribute to better anatomical and clinical outcomes in the treatment of complex aneurysms.

References

1. Aziz KMA, van Loveren HR, Tew JM Jr, et al: The Kawase approach to retrosellar and upper clival basilar aneurysms. **Neurosurgery 44:**1225–1236, 1999
2. Bavinzski G, Killer M, Gruber A, et al: Treatment of basilar artery bifurcation aneurysms by using Guglielmi detachable coils: a 6-year experience. **J Neurosurg 90:**843–852, 1999
3. Bavinzski G, Talazoglu V, Killer M, et al: Gross and microscopic histopathological findings in aneurysms of the human brain treated with Guglielmi detachable coils. **J Neurosurg 91:** 284–293, 1999
4. Brilstra EH, Rinkel GJE, van der Graaf Y, et al: Treatment of intracranial aneurysms by embolization with coils: a systematic review. **Stroke 30:**470–476, 1999
5. Byrne JV, Sohn MJ, Molyneux AJ, et al: Five–year experience in using coil embolization for ruptured intracranial aneurysms: outcomes and incidence of late rebleeding. **J Neurosurg 90:** 656–663, 1999
6. David CA, Vishteh AG, Spetzler RF, et al: Late angiographic follow-up review of surgically treated aneurysms. **J Neurosurg 91:**396–401, 1999
7. Day JD, Fukushima T, Giannotta SL: Cranial base approaches to posterior circulation aneurysms. **J Neurosurg 87:**544–554, 1997
8. Drake CG: The treatment of aneurysms of the posterior circulation. **Clin Neurosurg 26:**96–144, 1979
9. Feuerberg I, Lindquist C, Lindqvist M, et al: Natural history of postoperative aneurysm rests. **J Neurosurg 66:**30–34, 1987
10. Fox AJ, Viñuela F, Pelz DM, et al: Use of detachable balloons for proximal artery occlusion in the treatment of unclippable cerebral aneurysms. **J Neurosurg 66:**40–46, 1987
11. Giannotta SL, Maceri DR: Retrolabyrinthine transsigmoid approach to basilar trunk and vertebrobasilar artery junction aneurysms. Technical note. **J Neurosurg 69:**461–466, 1988
12. Gobin YP, Viñuela F, Gurian JH, et al: Treatment of large and giant fusiform intracranial aneurysms with Guglielmi detachable coils. **J Neurosurg 84:**55–62, 1996
13. Gruber A, Killer M, Bavinzski G, et al: Clinical and angiographic results of endosaccular coiling treatment of giant and very large intracranial aneurysms: a 7-year, single-center experience. **Neurosurgery 45:**793–804, 1999
14. Guglielmi G, Viñuela F, Dion J, et al: Electrothrombosis of saccular aneurysms via endovascular approach. Part 2: Preliminary clinical experience. **J Neurosurg 75:**8–14, 1991
15. Guglielmi G, Viñuela F, Duckwiler G, et al: Endovascular treatment of posterior circulation aneurysms by electrothrombosis using electrically detachable coils. **J Neurosurg 77:**515–524, 1992
16. Hayakawa M, Murayama Y, Duckwiler GR, et al: Natural history of the neck remnant of the cerebral aneurysm treated with the Guglielmi detachable coil system. **J Neurosurg 93:** 561–568, 2000
17. Hernesniemi J, Vapalahti M, Niskanen M, et al: Management outcome for vertebrobasilar artery aneurysms by early surgery. **Neurosurgery 31:**857–862, 1992
18. Hillman J, Saveland H, Jakobsson KE, et al: Overall management outcome of ruptured posterior fossa aneurysms. **J Neurosurg 85:**33–38, 1996
19. Horowitz MB, Purdy PD, Burns D, et al: Scanning electron microscopic findings in a basilar tip aneurysm embolized with Guglielmi detachable coils **AJNR 18:**688–690, 1997
20. Hunt WE, Hess RM: Surgical risk as related to time of inter-

vention in the repair of intracranial aneurysms. **J Neurosurg 28:**14–20, 1968
21. Jennett B, Bond M: Assessment of outcome after severe brain damage. A practical scale. **Lancet 1:**480–484, 1975
22. Kasdon DL, Stein BM: Combined supratentorial and infratentorial exposure for low-lying basilar aneurysm. **Neurosurgery 4:**422–426, 1979
23. Kassell NF, Torner JC, Haley EA Jr, et al: The International Cooperative Study on the Timing of Aneurysm Surgery. Part 1: Overall management results. **J Neurosurg 73:**18–36, 1990
24. Kawase T, Toya S, Shiobara R, et al: Transpetrosal approach for aneurysms of lower basilar artery. **J Neurosurg 63:**857–861, 1985
25. Kuether TA, Nesbit GM, Barnwell SL: Clinical and angiographic outcomes, with treatment data, for patients with cerebral aneurysms treated with Guglielmi detachable coils: a single-center experience. **Neurosurgey 43:**1016–1025, 1998
26. Lanzino G, Wakhloo, AK, Fessler RD, et al: Efficacy and current limitations of intravascular stents for intracranial internal carotid, vertebral, and basilar artery aneurysms. **J Neurosurg 91:**538–546, 1999
27. Lawton MT, Daspit CP, Spetzler RF: Technical aspects and recent trends in the management of large and giant midbasilar artery aneurysms. **Neurosurgery 41:**513–521, 1997
28. Lefkowitz MA, Gobin YP, Akiba Y, et al: Balloon-assisted Guglielmi detachable coiling of wide-necked aneurysms: Part II—clinical results. **Neurosurgery 45:**531–538, 1999
29. Lin T, Fox AJ, Drake CG: Regrowth of aneurysm sacs from residual neck following aneurysm clipping. **J Neurosurg 70:** 556–560, 1989
30. Malisch TW, Guglielmi G, Viñuela F, et al: Intracranial aneurysms treated with the Guglielmi detachable coil: midterm clinical results in a consecutive series of 100 patients. **J Neurosurg 87:**176–183, 1997
31. Manabe H, Fujita S, Hatayama T, et al: Rerupture of coil-embolized aneurysm during long-term observation. Case report. **J Neurosurg 88:**1096–1098, 1998
32. Martin NA: Basilar trunk aneurysm: surgical techniques, in Batjer HH (ed): **Cerebrovascular Disease.** Philadelphia: Lippincott-Raven, 1997, pp 1043–1053
33. Martin NA, Bentson J, Viñuela F, et al: Intraoperative digital subtraction angiography and the surgical treatment of intracranial aneurysms and vascular malformations. **J Neurosurg 73:** 526–533, 1990
34. Mericle RA, Wakhloo AK, Lopes DK, et al: Delayed aneurysm regrowth and recanalization after Guglielmi detachable coil treatment. Case report. **J Neurosurg 89:**142–145, 1998
35. Mizoi K, Yoshimoto T, Takahashi A, et al: Direct clipping of basilar trunk aneurysms using temporary balloon occlusion. **J Neurosurg 80:**230–236, 1994
36. Molyneux AJ, Ellison DW, Morris J, et al: Histological findings in giant aneurysms treated with Guglielmi detachable coils. Report of two cases with autopsy correlation. **J Neurosurg 83:** 129–132, 1996
37. Murayama Y, Viñuela F, Duckwiler GR, et al: Embolization of incidental cerebral aneurysms by using the Guglielmi detachable coil system. **J Neurosurg 90:**207–214, 1999
38. Murayama Y, Viñuela F, Suzuki Y, et al: Development of biologically active GDC for the treatment of cerebral aneurysms. Part II: an experimental study in a swine aneurysm model. **AJNR 20:**1992–1999, 1999
39. Murayama Y, Viñuela F, Suzuki Y, et al: Ion implantation and protein coating of detachable coils for endovascular treatment of cerebral aneurysms: concepts and preliminary results in swine models. **Neurosurgery 40:**1233–1244, 1997
40. Nichols DA, Brown RD Jr, Thielen KR, et al: Endovascular treatment of ruptured posterior circulation aneurysms using electrolytically detachable coils. **J Neurosurg 87:**374–380, 1997
41. Oglivy CS, Barker FG II, Joseph MP, et al: Transfacial transclival approach for midline posterior circulation aneurysms. **Neurosurgery 39:**736–742, 1996
42. Peerless SJ, Hernesniemi JA, Gutman FB, et al: Early surgery for ruptured vertebrobasilar aneurysms. **J Neurosurg 80:** 643–649, 1994
43. Pierot L, Boulin A, Castaings L, et al: Selective occlusion of basilar artery aneurysms using controlled detachable coils: report of 35 cases. **Neurosurgery 38:**948–954, 1996
44. Raymond J, Desfaits AC, Roy D: Fibrinogen and vascular smooth muscle cell grafts promote healing of experimental aneurysms treated by embolization. **Stroke 30:**1657–1664, 1999
45. Schievink WI, Wijdicks EFM, Piepgras DG, et al: The poor prognosis of ruptured intracranial aneurysms of the posterior circulation. **J Neurosurg 82:**791–795, 1995
46. Seifert V: Direct surgery of basilar trunk and vertebrobasilar junction aneurysms via the combined transpetrosal approach. **Neurol Med Chir 38 (Suppl):**86–92, 1998
47. Solomon RA, Stein BM: Surgical approaches to aneurysms of the vertebral and basilar arteries. **Neurosurgery 23:**203–208, 1988
48. Steinberg GK, Drake CG, Peerless SJ: Deliberate basilar or vertebral artery occlusion in the treatment of intracranial aneurysms. Immediate results and long-term outcome in 201 patients. **J Neurosurg 79:**161–173, 1993
49. Sugita K, Kobayashi S, Takemae T, et al: Aneurysms of the basilar artery trunk. **J Neurosurg 66:**500–505, 1987
50. Tsutsumi K, Ueki K, Usui M, et al: Risk of recurrent subarachnoid hemorrhage after complete obliteration of cerebral aneurysms. **Stroke 29:**2511–2513, 1998
51. Vanninen R, Koivisto T, Hernesniemi, et al: Ruptured intracranial aneurysms: acute endovascular treatment with electrolytically detachable coils—a prospective randomized study. **Radiology 211:**325–336, 1999
52. Viñuela F, Duckwiler G, Mawad M: Guglielmi detachable coil embolization of acute intracranial aneurysm: perioperative anatomical and clinical outcome in 403 patients. **J Neurosurg 86:** 475–482, 1997

Manuscript received July 31, 2000.

Accepted in final form June 26, 2001.

Address reprint requests to: Fernando Viñuela, M.D., Division of Interventional Neuroradiology, Department of Radiological Sciences, University of California at Los Angeles School of Medicine and Medical Center, 10833 Le Conte Avenue, Los Angeles, California 90024. email: fvinuela@mednet.ucla.edu.
