# Supplementary material for: Management strategies and outcomes of basilar trunk aneurysms: a systematic review and meta-analysis
Source: Langenbecks Arch Surg. 2026 Jan 20;411(1):71. doi: 10.1007/s00423-025-03959-3 (PMC12852281; doi:10.1007/s00423-025-03959-3)
Supplement: Supplementary file 1 — Supplementary file1 (ZIP 55327 KB) [file 423_2025_3959_MOESM1_ESM.zip › literature.zip!/meta/vanoel2013.pdf]

Reconstructive Endovascular Treatment of Fusiform and Dissecting Basilar Trunk Aneurysms with Flow Diverters, Stents, and Coils

L.I. van Oel, W.J. van Rooij, M. Sluzewski, G.N. Beute, P.N.M. Lohle, and J.P.P. Peluso

ABSTRACT

BACKGROUND AND PURPOSE: Patients with fusiform basilar trunk aneurysms have a poor prognosis. Reconstructive endovascular therapy is possible with modern devices. We describe the clinical presentation, radiologic features, and clinical outcome of 13 patients with fusiform basilar trunk aneurysms treated with flow diverters, stents, and coils.

MATERIALS AND METHODS: Of the 13 patients, 7 were men and 6 were women with a mean age of 59.7 years. Clinical presentation was SAH in 3 patients, mass effect on the brain stem in 4 patients, vertebral artery dissection in 1 patient, and the aneurysm was an incidental finding in 5 patients. Mean aneurysm size was 21 mm. All except 1 were large or giant aneurysms. Nine aneurysms were partially thrombosed.

RESULTS: Stents were used in all 13 patients, in 2 patients with additional flow diverters and in 11 patients with additional coils. In 4 patients, 1 vertebral artery was subsequently occluded with coils to decrease flow into the aneurysm. Of 13 patients, 9 had a good outcome with adequate aneurysm occlusion and stable size on follow-up of 6–72 months. One of 3 patients who presented with SAH died of a rebleed 1 month later. One other patient died soon after treatment of in-stent thrombosis, and another patient became mute after treatment. In 2 of 3 patients who presented with symptoms of mass effect, there was improvement at a follow-up of 6–24 months.

CONCLUSIONS: Reconstructive endovascular therapy of fusiform and dissecting basilar trunk aneurysms is feasible but carries substantial risks. The safety and effectiveness in relation to natural history has not yet been elucidated.

ABBREVIATIONS: 3DRA = 3D rotational angiography; GOS = Glasgow Outcome Score

Aneurysms of the basilar trunk are rare. They may be fusiform or saccular (sidewall). Fusiform aneurysms can be classified as segmental ectasia with a stretched and fragmented internal elastic lamina without intraluminal thrombus or as dissecting aneurysms with widespread disruption of the elastic lamina, thickened intima, and extensive intraluminal thrombus. Basilar trunk aneurysms most commonly present with SAH or mass effect due to brain stem compression and sometimes with ischemic stroke by dissection-induced occlusion of cerebellar or perforating arteries or by thromboembolism. When basilar trunk aneurysms are symptomatic, prognosis is poor. Ruptured dissecting basilar aneurysms are prone to rebleeding, with a high mortality. Basilar aneurysms that present with symptoms of mass effect on the brain stem and cranial nerves have a tendency to progressively increase in size with ultimately fatal mass effect.[1-5]

Modern endovascular techniques allow reconstructive therapy for fusiform basilar trunk aneurysms.[6-14] We describe the clinical presentation, radiologic features, and clinical outcome of 13 patients with basilar trunk aneurysms who were treated with flow diverters, stents, and coils.

MATERIALS AND METHODS

Patients

Between August 2005 and July 2011, thirteen patients with dissecting, fusiform, or dolichoectatic basilar trunk aneurysms were treated with reconstructive endovascular techniques by using coils, stents, and flow diverters. Patient and treatment characteristics are summarized in the On-line Table. There were 7 men and 6 women, with a mean age of 59.7 years (median, 61 years; range, 33–70 years). Clinical presentation was SAH in 3 patients, mass effect on the brain stem in 4 patients, posterior inferior cerebellar artery infarction following distal vertebral dissection in 1 patient; and in 5 patients, the aneurysm was an incidental finding on imaging studies for unrelated symptoms. Mean aneurysm size on cross-sectional imaging was 21 mm (median, 20 mm; range, 9–30

Received March 28, 2012; accepted after revision May 29.

From the Departments of Radiology (L.I.v.O., W.J.v.R., M.S., P.N.M.L., J.P.P.P.) and Neurosurgery (G.N.B.), St. Elisabeth Ziekenhuis, Tilburg, the Netherlands.

Please address correspondence to W.J. van Rooij, MD, Department of Radiology, St. Elisabeth Ziekenhuis, Hilvarenbeekseweg 60, 5022 GC Tilburg, the Netherlands; e-mail: radiol@eztilburg

Indicates article with supplemental on-line table.

http://dx.doi.org/10.3174/ajnr.A3255

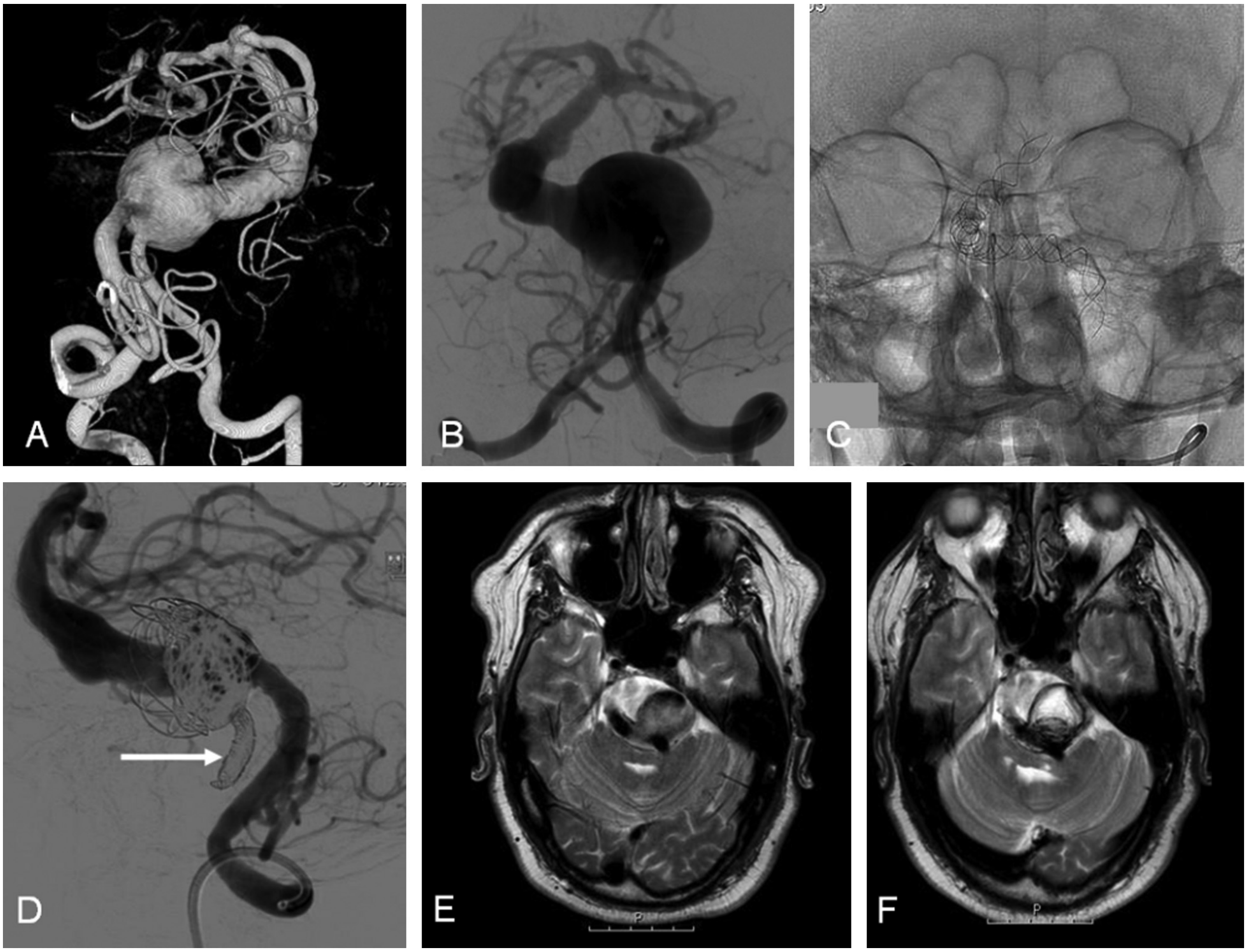

FIG 1. A 48-year-old man with intermittent dysphasia and dysarthria (patient 6). *A* and *B*, 3D and conventional frontal vertebral angiograms show a giant dolichoectatic fusiform aneurysm of the basilar artery. *C*, Two telescopically placed LEO stents are used as a scaffold for the flow diverter and coils. *D*, Lateral vertebral angiogram after a telescopically placed Silk flow diverter, coiling of the aneurysm lumen, and distal coil occlusion of the right vertebral artery (*arrow*). *E*, MR image before treatment shows a partially thrombosed aneurysm of the basilar artery with brain stem compression. *F*, MR imaging follow-up at 3 months demonstrates the thrombosed aneurysm with unchanged size.

mm). Of 13 aneurysms, 11 were large or giant and 9 were partially thrombosed.

Endovascular Treatment

Indications for treatment of patients with symptomatic and incidental basilar trunk aneurysms were discussed in a joint meeting with neurologists, neurosurgeons, and neuroradiologists. Treatment was tailored to the individual patient, accounting for various clinical and imaging parameters such as clinical presentation, patient age, the results of (serial) imaging, the presence of comorbidity, and patient preference. The choice of technique and the use of devices (stents, flow diverters, and coils) were dependent on anatomic geometry, size and length of the aneurysmal lumen, and the availability of devices. In the beginning of the study period, flow diverters were not yet available in our hospital. We preferred placing coils in the aneurysmal lumen after stent placement; when 2 good-caliber vertebral arteries were present, we preferred distal occlusion of 1 to decrease the flow into the aneurysm.

Endovascular treatment was performed with the patient under general anesthesia and with systemic heparinization on a biplane angiographic unit (Allura Neuro; Philips Healthcare, Best, the Netherlands) equipped with 3DRA.

Patients were preloaded with clopidogrel and aspirin. Angiography and 3DRA were performed through a single or bilateral vertebral artery contrast injection. Stable access to the basilar artery for stent placement was established with a 90-cm 6F introducer sheath (Destination; Terumo, Leuven, Belgium) positioned in the subclavian artery followed by insertion of a flexible 6F introducer catheter (Fargo; Balt, Montmorency, France) high in the vertebral artery, preferably in the V3 segment. In patients with 2 accessible vertebral arteries, a 5F introducer catheter was placed in the contralateral artery for control angiography and guidance of a second microcatheter if needed. From 3DRA images, the length and diameter of the stent were calculated by using standard machine software. For small stent sizes up to 4.5-mm vessel diameter and 32-mm length, an Enterprise stent was used (Codman, Raynham, Massachusetts), and for larger stent sizes, a LEO stent was used (Balt). Stents were telescopically placed in patients where long arterial segments needed to be bridged. In 2 patients, LEO stents were used as scaffolds to prevent shortening of telescopi-

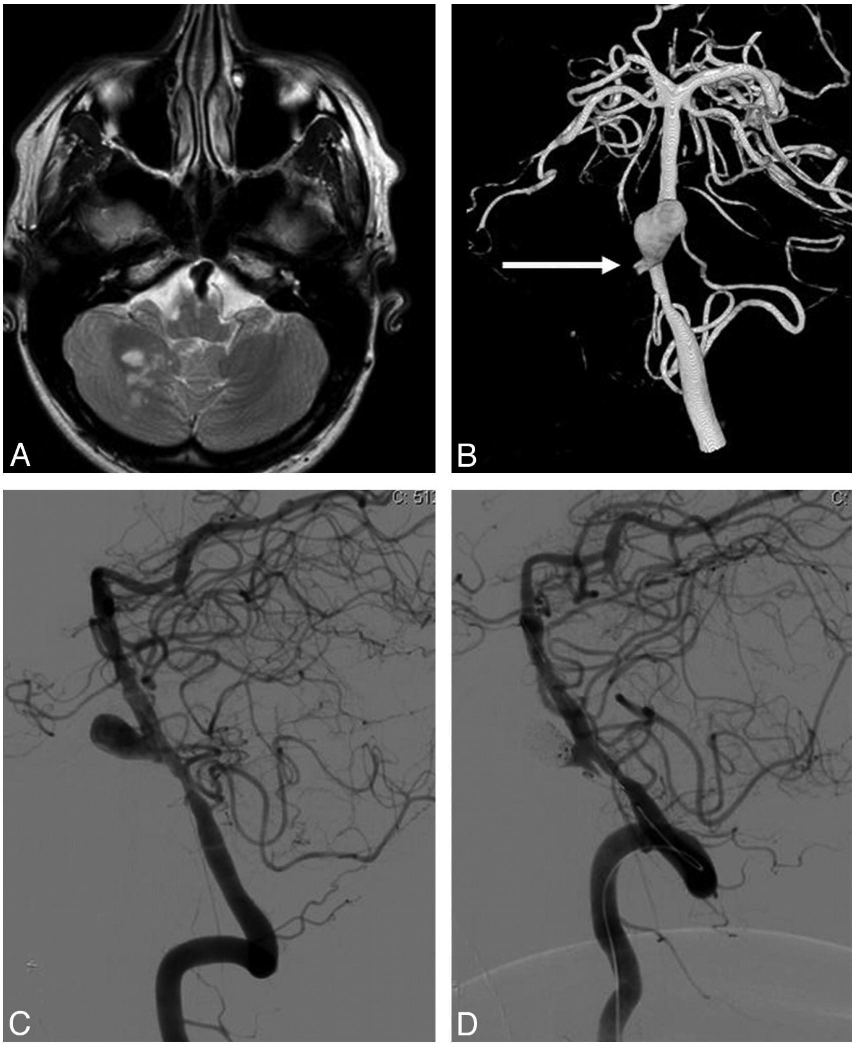

FIG 2. A 33-year-old man with a dissection and occlusion of the right vertebral artery, resulting in a PICA infarction (patient 8). *A*, MR image shows partial right PICA infarction and a proximal basilar dissecting aneurysm. *B*, 3D angiogram in a frontal view demonstrates a large dissecting proximal basilar aneurysm with occlusion of the distal right vertebral artery (*arrow* indicates the stump) and narrowing of the distal right vertebral artery. *C* and *D*, Lateral view of a left vertebral angiogram before (*C*) and after (*D*) stent placement and coiling of the dissecting aneurysm.

cally placed Silk flow diverters (Balt). After stent placement, additional coils (Axium; ev3, Irvine, California) were inserted in the aneurysmal lumen in 12 of the 13 aneurysms, either via a microcatheter through the stent struts or via a previously jailed microcatheter. To further decrease the flow in the aneurysm in an attempt to promote luminal thrombosis, in 4 of 13 patients, we finally occluded 1 of the 2 vertebral arteries with coils in the V4 segment just proximal to the vertebral junction.

After treatment, follow-up MR imaging or angiography was scheduled at 6–12 weeks.

RESULTS

The clinical and radiologic results are summarized in the On-line Table. In all 13 patients, it was technically feasible to place the stents, flow diverters, and coils as intended.

Complications of treatment occurred in 4 patients. Patient 3 (Fig 1) had a hemiparesis immediately after treatment and a brain stem infarction on MR imaging. Patient 4 experienced a myocardial infarction during general anesthesia, resulting in cardiac decompensation for which hospitalization of 4 weeks was necessary. Patient 7 did not wake up from general anesthesia, and repeat angiography demonstrated complete basilar thrombosis. Mechanical thrombectomy was successful, but the patient was brain dead and died the next day. Patient 10, who had a concomitant disseminated bile duct carcinoma, appeared mute following treatment. This condition remained until death from pulmonary embolism 3 weeks later.

Of 13 patients, 9 (69%) had a good functional outcome (GOS 1–2) at a median follow-up of 12 months (mean, 18; range, 6–72 months). One patient (patient 2, 8%) who presented with SAH in poor clinical condition is dependent in a nursing home after 24 months. Three patients (23%) died shortly after treatment: Patient 3 died 4 weeks after treatment of a recurrent SAH despite adequate occlusion of the aneurysm with coils; patient 7 died directly after treatment from in-stent basilar thrombosis; and patient 10, who was mute since treatment, died 3 weeks later of a pulmonary embolism.

Of 3 surviving patients who presented with symptoms of mass effect on the brain stem, 2 improved neurologically and 1 was unchanged.

Representative Cases

Case 1, Patient 6. A 48-year-old man presented with intermittent dysphasia and dysarthria (Fig 1). On MR imaging, a large and partially thrombosed basilar trunk aneurysm with brain stem compression was apparent. Angiography showed a giant fusiform dolichoectatic proximal basilar aneurysm. The aneurysm was treated with a Silk flow diverter telescopically placed in 2 overlapping LEO stents. The aneurysmal lumen was occluded with coils, and finally, the right vertebral artery was occluded with coils to decrease inflow in the basilar system. MR imaging follow-up at 3 months showed complete thrombosis of the aneurysm with unchanged aneurysm size. Clinically, there was no improvement of the brain stem symptoms.

Case 2, Patient 8. A 33-year-old man presented with a dissection of the right vertebral artery, resulting in a PICA infarction, an occlusion of the V4 segment of the right vertebral artery, and a large dissecting lower basilar aneurysm (Fig 2). Because the distal part of the left vertebral artery was narrowed, apparently the dissection extended over the vertebral junction into the opposite left V4 segment. The dissecting basilar aneurysm was completely occluded with coils after stent placement over the aneurysm neck

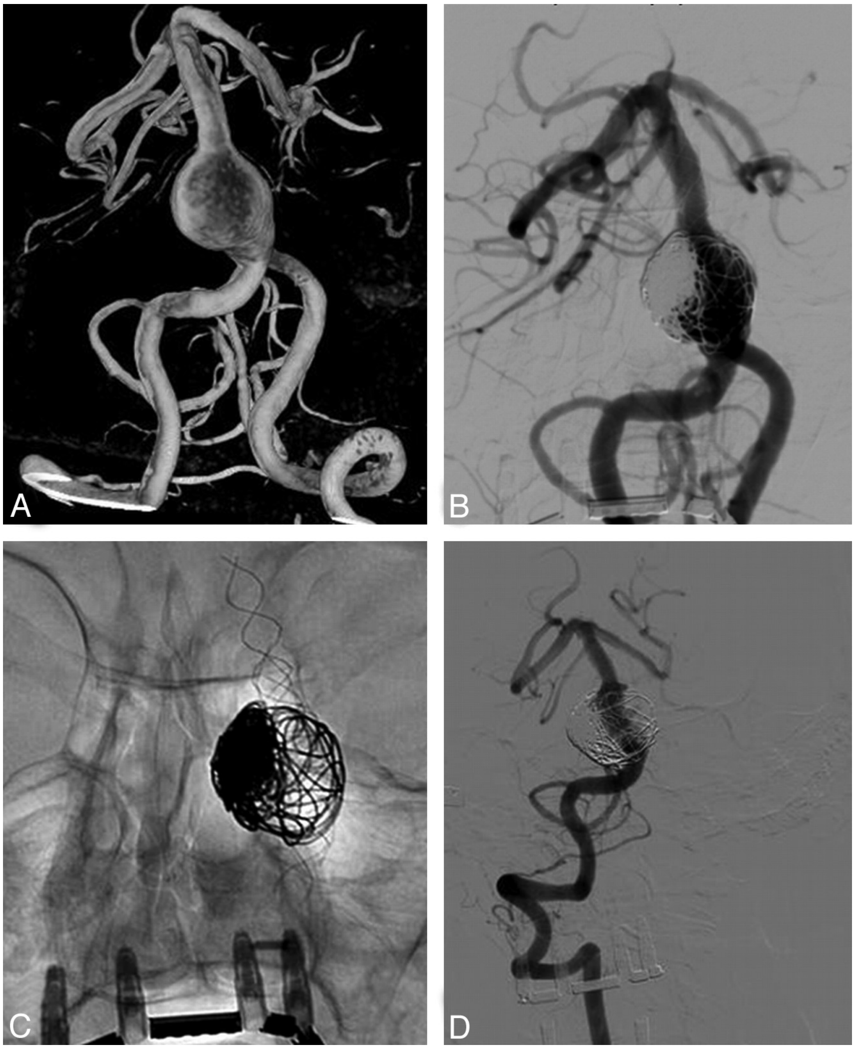

FIG 3. A 65-year-old woman with an incidentally found partially thrombosed fusiform basilar trunk aneurysm (patient 12). *A*, 3D angiogram shows a giant dolichoectatic fusiform aneurysm of the basilar trunk. *B* and *C*, Angiography after 3 telescopically placed LEO stents and coiling of the aneurysm lumen. *D*, Follow-up angiogram at 12 months shows complete occlusion of the fusiform aneurysm.

and the dissected and narrowed V4 segment of the left vertebral artery. Follow-up angiography at 14 months showed stable complete occlusion of the aneurysm. The patient made an uneventful clinical recovery.

Case 3, Patient 12. A 65-year-old woman had a transient ischemic attack; on CT, a partially thrombosed fusiform basilar trunk aneurysm was found incidentally (Fig 3). Angiography showed a large proximal fusiform basilar aneurysm in an ectatic vertebrobasilar system. The aneurysm was bridged with 3 telescopically placed LEO stents from the distal basilar artery into the distal right vertebral artery. Subsequently, the aneurysmal lumen was partially occluded with coils. Follow-up angiography at 6 months and 1 year showed progressive thrombosis of the aneurysm. Aneurysm size was unchanged at MR imaging. Clinically, she was asymptomatic.

Case 4, Patient 3. A 70 -year-old woman presented with an SAH of Hunt and Hess grade III. CT angiography showed a giant aneurysm on the basilar artery (Fig 4). Angiography confirmed the presence of a giant aneurysm on an ectatic and elongated basilar trunk, just distal to a proximal basilar fenestration. Three days later, a LEO stent was placed via the right vertebral artery in the basilar trunk across the aneurysmal neck. Subsequently, 1130 cm of coils was inserted in the aneurysmal lumen, and the aneurysm appeared adequately occluded. Several hours later, the patient developed a right-sided hemiplegia. MR imaging the next day showed a brain stem infarction and a small cerebellar infarction on the right side. The patient gradually neurologically improved and was discharged home 2 weeks later. Four weeks after treatment, she was found comatose at home, and a CT scan revealed a recurrent SAH. She died the next day.

Case 5, Patient 7. A 64-year-old man presented with abducens paresis. MR imaging showed a large dolichoectatic partially thrombosed proximal basilar aneurysm (Fig 5). Angiography demonstrated the aneurysmal lumen and irregular and ectatic distal vertebral arteries. After telescopic placement of 2 LEO stents from the distal basilar artery into the right vertebral artery, a Silk flow diverter was inserted, the aneurysm lumen was filled with coils, and the left vertebral artery was occluded with a detachable balloon in the V4 segment. After embolization, the patient did not wake up from general anesthesia, and repeated right vertebral angiography showed in-stent thrombosis in the V4 segment. Mechanical thrombectomy was performed resulting in recanalization of the basilar artery, but with persistent occlusion of the right posterior cerebral artery. The clinical condition did not improve, and brain death was established. The patient died the same day.

DISCUSSION

We found that with modern endovascular techniques and devices, reconstructive treatment of large and giant dolichoectatic and dissecting basilar trunk aneurysms is feasible. Conventional stents with sufficiently large diameters can be placed telescopically to negotiate long dilated and ectatic vessel segments and to serve as a scaffold for additional telescopically inserted flow diverters and/or coils in the remaining aneurysmal lumen.

Whether this kind of treatment improves the prognosis of patients with large fusiform basilar aneurysms remains an unanswered question. On the one hand, fusiform basilar artery aneurysms are associated with a high rate of mortality and disability. If left untreated, progressive brain stem compression or subarachnoid hemorrhage may occur with 2-year survival rates as low as 20%.[1-5] With SAH, the chance of rebleed is high and almost in-

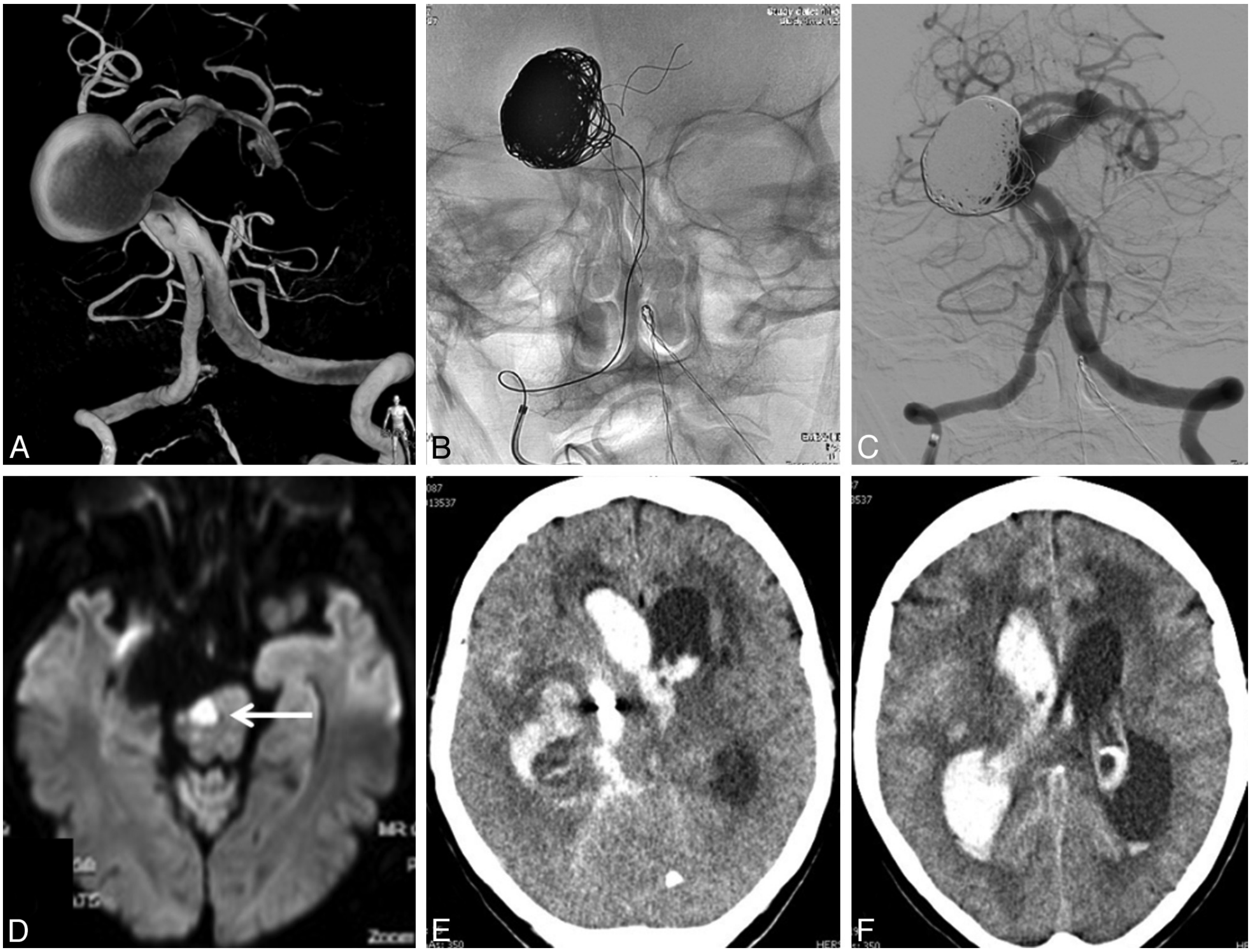

FIG 4. A 70-year-old woman presenting with SAH (patient 3). *A*, 3D angiography shows a giant dolichoectatic aneurysm of the basilar trunk with a proximal fenestration. *B* and *C*, Adequate coil occlusion of the aneurysm after stent placement. *D*, Diffusion MR imaging the next day performed for sudden hemiparesis shows a brain stem infarction. *E* and *F*, Massive recurrent SAH 1 month after endovascular treatment.

variably fatal.[4,5,15,16] In view of this poor natural history, treatment should be considered in symptomatic patients. Conventional surgical treatment consists of Hunterian arterial occlusion of the proximal basilar trunk or 1 or both vertebral arteries in patients with sufficient collateral circulation over the posterior communicating arteries. Other surgical methods consist of aneurysm wrapping, bleb clipping, or bypass surgery preceding vertebral or basilar occlusion.[1,16,17]

In a report on 201 patients with predominantly giant posterior circulation aneurysms, treatment by surgical occlusion of the parent artery had good result in 68%, but 25% died. Outcome varied depending on the patient's condition on admission and the site of the aneurysm.[16] In recent years, surgical parent vessel occlusion has been largely replaced by endovascular techniques to occlude the vessels with detachable balloons or coils. With endovascular treatment, collateral circulation over the posterior communicating arteries can be tested in the awake patient before definitive occlusion. The few anecdotal reports and small case series on the treatment of giant vertebrobasilar aneurysms with balloon occlusion of afferent arteries vary widely with regard to patient selection and aneurysm characteristics. All authors agree to the importance of the size of the posterior communicating arteries to provide sufficient collateral flow to the territory of the occluded vessels. Little is known about the long-term treatment effect in respect to mass effect on the brain stem or prevention of primary or recurrent subarachnoid hemorrhage.[15,18,19]

With the recent introduction of easy-to-place stents and flow diverters, reconstructive (instead of deconstructive) endovascular treatment is now possible in most patients with giant fusiform vertebrobasilar aneurysms. Although technically challenging, feasibility is high. Some technical issues are not yet clarified. Should the remaining aneurysm lumen after stent or flow diverter placement be filled with coils? Is an additional flow diverter placed in a conventional stent necessary or is a stent alone sufficient to induce thrombosis of the remaining part of the aneurysm? Is additional unilateral vertebral occlusion after stent placement necessary to decrease the flow and hence promote thrombosis of the aneurysm? If yes, should treatment be performed in a single session or staged?[20,21]

Our results in a limited series of 13 patients do not answer these questions. Of 13 patients, 9 had a good outcome with adequate aneurysm occlusion and stable aneurysm size on follow-up of 6–72 months. One of 3 patients who presented with subarachnoid hemorrhage died of a rebleed despite angiographically adequate occlusion of the aneurysm. One other patient died soon after treatment of in-stent thrombosis, and another patient be-

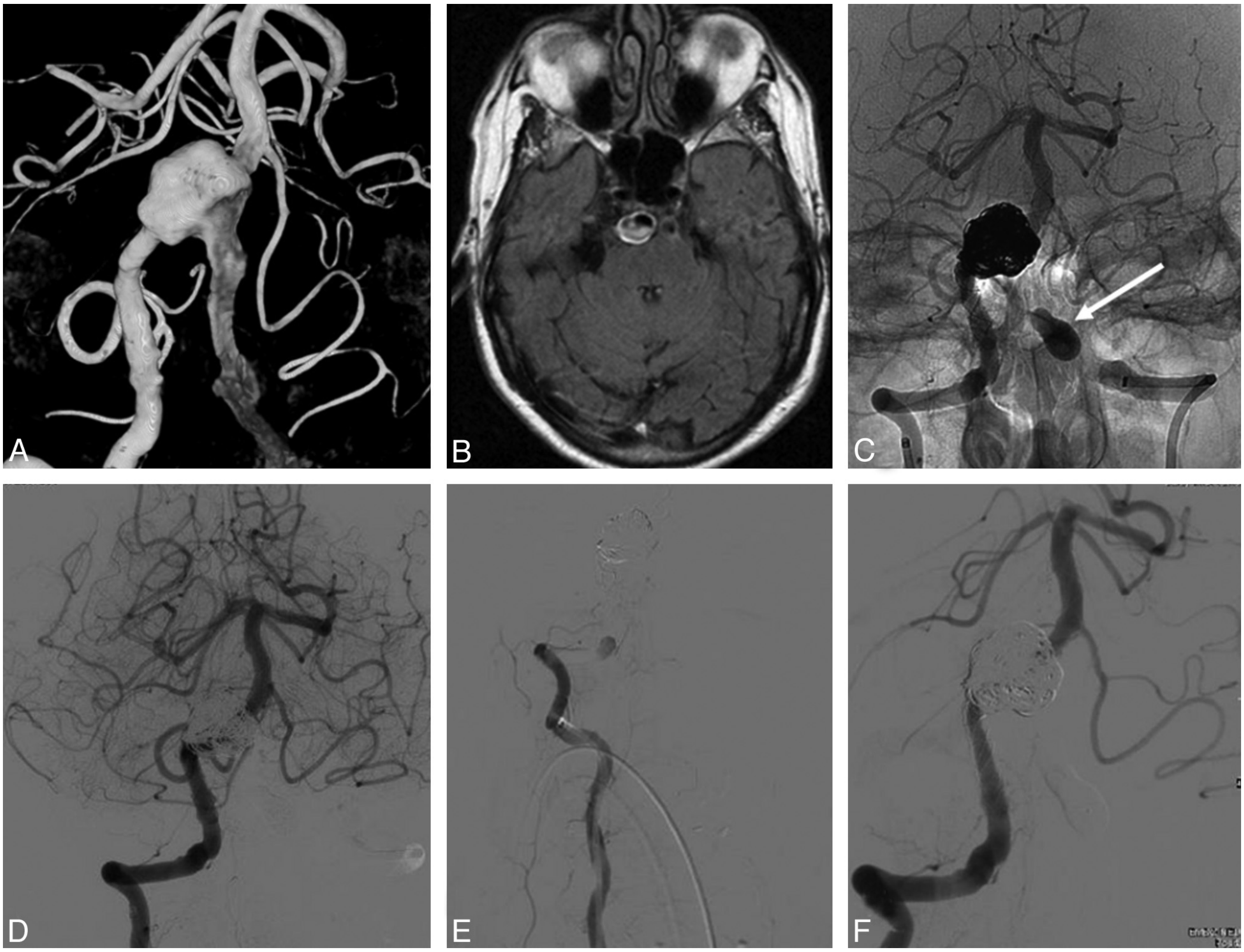

FIG 5. A 64-year-old man with abducens paresis (patient 7). *A* and *B*, 3D angiography and MR imaging demonstrate a large dolichoectatic partially thrombosed basilar trunk aneurysm. Note irregular and ectatic distal vertebral arteries. *C* and *D*, Right vertebral angiography after telescopic placement of 2 LEO stents and a Silk flow diverter, coiling of the aneurysm lumen, and balloon occlusion of the left vertebral artery in the V4 segment (*arrow* in *C*). *E*, After embolization, the patient did not wake up from general anesthesia. Repeated right vertebral angiography shows in-stent thrombosis in the V4 segment. *F*, Recanalization of the basilar artery after mechanical thrombectomy but with persistent occlusion of the right posterior cerebral artery. The patient died the same day.

came mute after treatment. In 2 of 3 patients who presented with signs of mass effect, symptoms improved at a follow-up of 6–24 months. Of 5 patients with incidentally discovered aneurysms, 4 had a good outcome after treatment and 1 patient with a disseminated carcinoma died eventually of pulmonary embolism. Our results are largely in concordance with the few scattered reports published so far on this type of treatment. Most patients do well, but serious complications are not uncommon.[6-14,20,21] In a recent report of 7 patients (6 symptomatic, 1 incidental) with fusiform vertebrobasilar artery aneurysms treated with flow diverters, 4 patients died, 1 was severely disabled, and only 2 did well.[14]

Long-term effects or benefits of reconstructive endovascular treatment are not yet completely understood. So far, the complication rate of reconstructive treatment of fusiform vertebrobasilar artery aneurysms seems particularly high in symptomatic patients, while the outcome of treatment in patients with incidentally discovered aneurysms appears rather good. These findings would suggest that treatment should be offered in asymptomatic patients, especially when serial imaging indicates growth of the fusiform aneurysm. In patients presenting with (progressive) mass effect on the brain stem, treatment may be offered despite the risk of complications because the natural history is very poor. In patients presenting with subarachnoid hemorrhage, treatment is indicated in view of the high chance of recurrent hemorrhage.

CONCLUSIONS

Patients with large fusiform dolichoectatic and dissecting aneurysms of the basilar trunk are clinically challenging. The natural history is poor, especially when the aneurysm is symptomatic by mass effect or SAH. Therefore, reconstructive endovascular treatment may be offered in both symptomatic and asymptomatic patients, despite the substantial procedural risk and uncertain clinical benefit in the long term. More data are needed to elucidate the many clinical dilemmas involved in this patient group.

Disclosures: Willem Jan van Rooij—*UNRELATED*: *Grants/Grants Pending*: Vertebroplasty for Painful Acute Osteoporotic Vertebral Fractures IV.* *Money paid to the institution.

REFERENCES

1. Mizutani T, Miki Y, Kojima H, et al. **Proposed classification of nonartherosclerotic cerebral fusiform and dissecting aneurysms.** *Neurosurgery* 1999;45:253–59
2. Drake CG, Peerless SJ. **Giant fusiform intracranial aneurysms: review of 120 patients treated surgically from 1965–1992**. *J Neurosurg* 1997;87:141–62
3. Rinkel GJ, Djibuti M, Algra A, et al. **Prevalence and risk of rupture of intracranial aneurysms—a systematic review.** *Stroke* 1998;29:251–56
4. Schievink WI, Wijdicks EF, Piepgras DG, et al. **The poor prognosis of ruptured intracranial aneurysms of the posterior circulation.** *J Neurosurg* 1995;82–791-95
5. **Unruptured intracranial aneurysms: risk of rupture and risks of surgical intervention—the International Study of Unruptured Intracranial Aneurysms Investigators.** *N Engl J Med* 1998;339:1725–33
6. Lubicz B, Collignon L, Raphaeli G, et al. **Flow-diverter stent for the endovascular treatment of intracranial aneurysms: a prospective study in 29 patients with 34 aneurysms.** *Stroke* 2010;41:2247–53
7. Lylyk P, Miranda C, Ceratto R, et al. **Curative endovascular reconstruction of cerebral aneurysms with the Pipeline embolization device: the Buenos Aires experience.** *Neurosurgery* 2009;64:632–43, discussion 642–43, quiz N6
8. Szikora I, Berentei Z, Kulcsar Z, et al. **Treatment of intracranial aneurysms by functional reconstruction of the parent artery: the Budapest experience with the Pipeline embolization device.** *AJNR Am J Neuroradiol* 2010;31:1139–47
9. Kulcsár Z, Ernemann U, Wetzel SG, et al. **High-profile flow-diverter (Silk) implantation in the basilar artery: efficacy in the treatment of aneurysms and the role of the perforators.** *Stroke* 2010;41:1690–96
10. Lubicz B, Collignon L, Raphaeli G, et al. **Pipeline flow-diverter stent for endovascular treatment of intracranial aneurysms: preliminary experience in 20 patients with 27 aneurysms.** *World Neurosurg* 2011;76:114–19
11. Fischer S, Vajda Z, Aguilar Perez M, et al. **Pipeline embolization device (PED) for neurovascular reconstruction: initial experience in the treatment of 101 intracranial aneurysms and dissections.** *Neuroradiology* 2012;54:369–82
12. Wakhloo AK, Mandell J, Gounis MJ, et al. **Stent-assisted reconstructive endovascular repair of cranial fusiform atherosclerotic and dissecting aneurysms: long-term clinical and angiographic follow-up.** *Stroke* 2008;39:3288–96
13. Cohen JE, Gomori JM, Moscovici S, et al. **Successful endovascular treatment of a growing megadolichoectasic vertebrobasilar artery aneurysm by flow diversion using the "diverter-in-stent" technique.** *J Clin Neurosci* 2012;19:166–70
14. Siddiqui AH, Abla AA, Kan P, et al. **Panacea or problem: flow diverters in the treatment of symptomatic large or giant fusiform vertebrobasilar aneurysms.** *J Neurosurg* 2012;116:1258–66
15. Sluzewski M, Brilstra EH, van Rooij WJ, et al. **Bilateral vertebral artery balloon occlusion for giant vertebrobasilar aneurysms.** *Neuroradiology* 2001;43:336–41
16. Steinberg GK, Drake CG, Peerless SJ. **Deliberate basilar or vertebral artery occlusion in the treatment of intracranial aneurysms: immediate results and long-term outcome in 201 patients.** *J Neurosurg* 1993;79: 161–73
17. Tulleken CA, van der Zwan A, van Rooij WJ, et al. **High-flow bypass using nonocclusive excimer laser-assisted end-to-side anastomosis of the external carotid artery to the P1 segment of the posterior cerebral artery via the sylvian route: technical note.** *J Neurosurg* 1998;88:925–27
18. Lubicz B, Leclerc X, Gauvrit JY, et al. **Giant vertebrobasilar aneurysms: endovascular treatment and long-term follow-up.** *Neurosurgery* 2004;55:316–23
19. van Rooij WJ, Sluzewski M, Menovsky T, et al. **Coiling of saccular basilar trunk aneurysms.** *Neuroradiology* 2003;45:19–21
20. Cebral JR, Mut F, Raschi M, et al. **Aneurysm rupture following treatment with flow-diverting stents: computational hemodynamics analysis of treatment**. *AJNR Am J Neuroradiol* 2011;32:27–33
21. van Rooij WJ, Sluzewski M. **Perforator infarction after placement of a Pipeline flow-diverting stent for an unruptured A1 aneurysm.** *AJNR Am J Neuroradiol* 2010;31:E43–44
22. Peluso JP, van Rooij WJ, Sluzewski M. **Rapidly growing basilar dissecting aneurysm**. *BMJ Case Rep* 2009;2009:bcr2007121533
