# Supplementary material for: Management strategies and outcomes of basilar trunk aneurysms: a systematic review and meta-analysis
Source: Langenbecks Arch Surg. 2026 Jan 20;411(1):71. doi: 10.1007/s00423-025-03959-3 (PMC12852281; doi:10.1007/s00423-025-03959-3)
Supplement: Supplementary file 1 — Supplementary file1 (ZIP 55327 KB) [file 423_2025_3959_MOESM1_ESM.zip › literature.zip!/meta/Wallace 2019.pdf]

Check for updates

Pipeline Embolization of Vertebrobasilar Aneurysms—A Multicenter Case Series

Adam N. Wallace[1,2], Thomas P. Madaelil[3], Mudassar Kamran[4], Timothy R. Miller[5], Josser E. Delgado Almandoz[1], Jonathan A. Grossberg[6], Akash P. Kansagra[4,7,8], Dheeraj Gandhi[5], Yasha Kayan[1], C. Michael Cawley[6], Christopher J. Moran[4,7], Gaurav Jindal[9,10], Travis CreveCoeur[7], Brian M. Howard[6], DeWitte T. Cross[4,7], Matthew J. Kole[10], Anil K. Roy[6], Jacques E. Dion[3], Joshua W. Osbun[7,4,8]

■ BACKGROUND: **The Pipeline Embolization Device (PED) has been increasingly used for the treatment of posterior circulation aneurysms. The purpose of the present study was to examine the clinical and angiographic outcomes of patients with vertebrobasilar aneurysms treated with the PED.**

■ METHODS: **We performed a retrospective review of vertebrobasilar aneurysms treated with the PED at 4 high-volume neurovascular centers. Patient, aneurysm, and procedural data were collected, including perioperative and delayed complications. Aneurysm occlusion on follow-up imaging studies was defined as complete (100%), near-complete (>90%), or incomplete (<90%) occlusion.**

■ RESULTS: **The cohort included 35 patients with 37 vertebrobasilar aneurysms who underwent 36 treatment sessions. Of the 35 patients, 10 were men (29%), and the mean patient age was 54.1 years (range, 32—75). Eight patients (23%) underwent urgent treatment because of a ruptured aneurysm ($n = 6$), brainstem perforator stroke ($n = 1$), or post-traumatic pseudoaneurysm ($n = 1$). Of the 37 aneurysms, 22 arose from the vertebral artery (59%) and 15 from the basilar artery (41%). Also, 19 were saccular aneurysms (51%), with a mean size of 7.7 mm (range, 1.7—38.0); 17 were fusiform aneurysms (46%), with a mean size of 11.0 mm (range, 4.3—34); and 1 was a 2.9-mm blister aneurysm. The overall procedural complication rate was 14% (5 of 36), including 3 neurologically symptomatic complications. At a mean follow-up period of 14 months (range, 3—59), 24 of 34 aneurysms (71%) were completely occluded and 29 of 34 (85%) were completely or near-completely occluded.**

■ CONCLUSION: **Our results show that Pipeline embolization of vertebrobasilar aneurysms is associated with acceptable occlusion and complication rates.**

INTRODUCTION

The Pipeline Embolization Device (PED) has greatly expanded the range of aneurysms amenable to endovascular treatment, including those with wide necks that incorporate branch vessels. The reported occlusion rates for anterior circulation aneurysms treated with the PED have typically been >85%,[1-7] with morbidity rates of <7%.[7-9] Prompted by this success, the PED has been increasingly used for the off-label treatment of posterior circulation aneurysms, with mixed results.[10-12] The purpose of the present study was to examine the procedural, clinical, and angiographic outcomes of patients with vertebrobasilar aneurysms treated with the PED.

Key words
- Aneurysm
- Pipeline embolization
- Posterior circulation
- Vertebrobasilar

Abbreviations and Acronyms
CI: Confidence interval
DAPT: Dual antiplatelet therapy
DSA: Digital subtraction angiography
HR: Hazard ratio
mRS: modified Rankin scale
PCA: Posterior cerebral artery
PED: Pipeline Embolization Device
PICA: Posterior inferior cerebellar artery
PRU: P2Y12 reaction unit
SAH: Subarachnoid hemorrhage
VA: Vertebral artery

From the [1]Division of Neurointerventional Radiology, Neuroscience Institute, Abbott Northwestern Hospital, Minneapolis, Minnesota; [2]Department of Radiology, University of Iowa, Iowa City, Iowa; [3]Department of Radiology, Emory University, Atlanta, Georgia; [4]Mallinckrodt Institute of Radiology, Washington University, St. Louis, Missouri; [5]Department of Radiology, University of Maryland, Baltimore, Maryland; [6]Department of Neurosurgery, Emory University, Atlanta, Georgia; Departments of [7]Neurosurgery, and [8]Neurology, Washington University, St. Louis, Missouri; and Departments of [9]Neurology, and [10]Neurosurgery, University of Maryland, Baltimore, Maryland, USA

To whom correspondence should be addressed: Adam N. Wallace, M.D. [E-mail: adam.wallace@crlmed.com]

Citation: World Neurosurg. (2019) 124:e460-e469.
https://doi.org/10.1016/j.wneu.2018.12.116

*Journal homepage: **www.journals.elsevier.com/world-neurosurgery***

*Available online: **www.sciencedirect.com***

1878-8750/$ - see front matter © 2019 Elsevier Inc. All rights reserved.

METHODS

Data Collection

Retrospective review of the neurointerventional databases of four high-volume neurovascular centers was approved by respective institutional review boards to identify patients with vertebrobasilar aneurysms that were treated with the PED. Each participating institution performs >100 endovascular aneurysm treatments annually and has deployed >200 PEDs. The patients were selected for PED treatment only when other techniques (e.g., stent-assisted coiling) were considered to be of greater risk and after a detailed discussion with the patient regarding the off-label nature of PED treatment for posterior circulation aneurysms. Demographic, clinical, and angiographic data were collected from electronic medical records. Procedural details, including the devices used, were collected from the operative reports. Procedure-related complications were prospectively documented in each institution's database, including thromboembolic events, intracranial hemorrhage, arterial puncture complications, and events related to dual antiplatelet therapy (DAPT). Preoperative digital subtraction angiography (DSA) was reviewed to determine the morphological characteristics of the treated aneurysms, including the largest diameter in any dimension, location, shape, and incorporated branch vessels.

Antiplatelet Regimens

All patients received peri- and post-procedural DAPT and underwent platelet function testing in accordance with individual institution protocols. Patients 1–3 and 5–8 received DAPT for 10–14 days before their elective treatments. The clopidogrel response was assessed using platelet aggregometry 4–7 days before and on the day of the procedure. Clopidogrel-resistant patients were switched to prasugrel or ticagrelor. Patients 9, 11, 12, and 15–17 received DAPT for 3 weeks before elective treatment, and the clopidogrel response was assessed using VerifyNow (Accumetrics, San Diego, California, USA) 10 days and 1 day before the procedure. The clopidogrel doses were adjusted to achieve a goal of 60–200 P2Y12 reaction units (PRUs), and clopidogrel hyporesponders were switched to prasugrel. Patients 19–27 started DAPT 17 days before elective treatment. The clopidogrel response was assessed using VerifyNow (Accumetrics) testing 10 days and 1 day before the procedure, and the doses were adjusted to achieve a target of 60–240 PRUs. Clopidogrel-resistant patients were switched to ticagrelor. Patients 28, 29, and 31–35 received DAPT for 4 weeks before elective treatment. VerifyNow (Accumetrics) testing was performed 2 weeks before treatment, and clopidogrel was adjusted to achieve a goal of 100–200 PRUs. The clopidogrel-resistant patients were switched to ticagrelor. The remaining patients underwent urgent treatment for ruptured aneurysms within 48 hours of presentation and received a loading dose of aspirin and a P2Y12 inhibitor (clopidogrel or ticagrelor) before treatment. DAPT was continued for ≥9 months after treatment for all patients.

Procedure Characteristics

The procedure characteristics are summarized in Table 1. Triaxial access was used for 29 of 36 procedures (81%). The sheaths used for triaxial access included a 6F or 7F Shuttle (Cook Medical, Bloomington, Indiana, USA) in 19 of 36 treatment sessions (53%); a 6F or 7F Arrow (Teleflex Medical, Research Triangle Park, North Carolina, USA) in 4 (11%); a Neuron Max (Penumbra, Inc., Alameda, California, USA) in 5 (14%); or a 6F Envoy (DePuy Synthes, Raynham, Massachusetts, USA) in 1 treatment session (3%). The intermediate catheters included a 0.058-in. Navien (Medtronic, Minneapolis, Minnesota, USA) in 18 of 36 treatment sessions (50%), a 0.058-in. ReFlex (Covidien, Mansfield, Massachusetts, USA) in 4 (11%), a 5F Benchmark (Penumbra, Inc.) in 1 (3%), an 0.044-in. DAC (digital access catheter) (Stryker, Kalamazoo, Michigan, USA) in 4 (11%), a 5F Sofia (MicroVention, Aliso Viejo, California, USA) in 1 (3%), and a 0.045-in. Phenom Plus (Medtronic) in 1 treatment session (3%). The remaining cases were performed using only a 6F Benchmark in 5 (14%), a 0.053-in. Neuron (Penumbra, Inc.) in 1 (4%), or a 0.044-in. DAC in 1 (4%) for proximal support. The PED was delivered through a Marksman microcatheter (Medtronic) in 32 of 36 treatment session (89%) or a 0.027-in. Phenom microcatheter (Medtronic) in 4 (11%). Of the 37 aneurysms, 34 (92%) were treated with a single PED and 3 required treatment with 2–4 PEDs. The original PED was used in 14 of the 36 procedures (39%) and the Flex iteration in 22 (61%). The PEDs ranged from 2.75 to 5 mm in diameter and from 10 to 35 mm in length. Of the 37 aneurysms, 5 (14%) were adjunctively coiled at the time of Pipeline embolization.

Clinical and Angiographic Outcome Assessment

Clinical outcomes were assessed using the modified Rankin scale (mRS). Post-treatment surveillance angiography protocols varied by institution. All patients underwent follow-up DSA at 3–6 months after treatment. Longer term follow-up imaging studies were performed using DSA, magnetic resonance angiography, or computed tomography angiography at the discretion of the treating physician. Angiographic outcomes were categorized as complete (100%), near-complete (> 90%), or incomplete (< 90%) occlusion. The follow-up angiographic images were also reviewed for intra-PED stenosis and patency of the branch vessels covered by the PED.

RESULTS

Patient and Aneurysm Characteristics

During the study period, 35 patients with 37 vertebrobasilar aneurysms underwent 36 treatment sessions with the PED. The pertinent demographic, clinical, and aneurysm characteristics of the cohort are summarized in Table 2. Of the 35 patients, 10 were men (29%) and 25 were women (71%). Their mean age was 54.1 years (range, 32–75), and 17 (49%) were previous or current smokers. Aneurysms were incidentally found in 25 of the 35 patients (71%), and 2 patients (patients 33 and 35) were treated for recurrent, previously ruptured aneurysms that were asymptomatic at the time of Pipeline embolization (6%). Of the remaining 8 patients, 6 (17%) presented with subarachnoid hemorrhage (SAH), 1 (2.9%) presented with stroke related to thrombosis of a brainstem perforator incorporated into the aneurysm (patient 20), and 1 patient (2.9%) presented with a gunshot wound to the neck (patient 34). The clinical

Table 1. Procedure Characteristics of Pipeline Embolization of Vertebrobasilar Aneurysms

Pt No.	Procedure No.	Aneurysm No.	Proximal Guide Catheter/Sheath	Intermediate Catheter	PED Delivery Catheter	PEDs (*n*)	PED Iteration	PED Size (mm)	Coils Used	Complications
1	1	1	6F Shuttle	0.058-in. Navien	Marksman	1	Flex	2.75 × 14	No	No
2	2	2	7F Arrow	0.058-in. ReFlex	Marksman	1	Original	4.5 × 35	Yes	No
3	3	3	053 Neuron	None	Marksman	1	Original	3.2 × 20	No	No
4	4	4	6F Arrow	071 Benchmark	Marksman	1	Original	4.5 × 16	No	No
5	5	5	6F Arrow	0.044-in. DAC	Marksman	1	Flex	2.75 × 18	No	No
6	6	6	6F Arrow	0.058-in. Navien	Marksman	1	Original	3.5 × 20	No	IPH
7	7	7	6F Shuttle	0.058-in. Navien	Marksman	1	Flex	2.75 × 16	No	No
8	8	8	6F Arrow	0.058-in. Navien	Marksman	1	Flex	4 × 25	No	No
9	9	9	6F Shuttle	0.058-in. ReFlex	Marksman	1	Original	4.5 × 20	No	No
10	10	10	7F Shuttle	0.058-in. Navien	Marksman	1	Original	3.5 × 25	No	Fractured PED delivery wire
10	11	10	7F Shuttle	0.058-in. Navien	Marksman	1	Original	4.25 × 16	No	No
11	12	11*	7F Shuttle	0.058-in. Navien	Marksman	1	Original	3 × 16	No	No
11	12	12*								
12	13	13	6F Shuttle	0.058-in. Navien	Marksman	1	Flex	3.75 × 16	No	No
13	14	14	6F Shuttle	0.058-in. Navien	Marksman	1	Original	3 × 18	No	No
14	15	15	7F Shuttle	0.058-in. Navien	Marksman	1	Original	3.5 × 18	Yes	No
15	16	16	6F Shuttle	5F Sofia	Marksman	1	Flex	4.75 × 20	No	Stroke
16	17	17	6F Shuttle	0.058-in. Navien	Marksman	1	Flex	6.5 × 16	No	No
17	18	18	6F Shuttle	0.058-in. Navien	Marksman	3	Flex	5 × 25, 5 × 35, 5 × 35	No	No
18	19	19	6F Benchmark	None	Marksman	1	Flex	3.5 × 10	No	No
19	20	20	6F Shuttle	0.058-in. ReFlex	Marksman	1	Flex	3 × 14	No	No
20	21	21	Neuron Max	0.058-in. Navien	Marksman	3	Original	5 × 25, 5 × 30, 5 × 35	No	No
21	22	22	Neuron Max	0.058-in. Navien	Marksman	1	Flex	3.5 × 18	No	No
22	23	23†	Neuron Max	0.058-in. Navien	Phenom 27	2	Flex	5 × 25, 5 × 35	No	Stroke
22	23	24†				1	Flex	5 × 25	No	No
23	24	25	6F Benchmark	None	Marksman	1	Flex	3 × 10	No	No
24	25	26	6F Benchmark	None	Marksman	1	Flex	3.25 × 10	No	No
25	26	27	Neuron Max	0.058-in. Navien	Marksman	1	Flex	3 × 10	No	No
26	27	28	6F Benchmark	None	Phenom 27	1	Flex	3 × 14	Yes	No
27	28	29	6F Benchmark	None	Marksman	1	Original	3.5 × 10	No	No
28	29	30	Neuron Max	Phenom Plus	Phenom 27	1	Flex	4.0 × 20	No	No
29	30	31	6F Shuttle	0.058-in. Navien	Phenom 27	1	Flex	3.5 × 25	No	No
30	31	32	6F Shuttle	0.044-in. DAC	Marksman	1	Flex	4 × 20	Yes	No
31	32	33	None	0.044-in. DAC	Marksman	1	Flex	3.75 × 25	Yes	No
32	33	34	6F Shuttle	0.044-in. DAC	Marksman	1	Flex	4.5 × 20	No	No
33	34	35	6F Envoy	0.044-in. DAC	Marksman	1	Flex	3 × 16	No	No

Continues

Table 1. Continued

Pt No.	Procedure No.	Aneurysm No.	Proximal Guide Catheter/Sheath	Intermediate Catheter	PED Delivery Catheter	PEDs (*n*)	PED Iteration	PED Size (mm)	Coils Used	Complications
34	35	36	6F Shuttle	0.058-in. Navien	Marksman	1	Original	3.25 × 30	No	No
35	36	37	6F Shuttle	0.058-in. ReFlex	Marksman	1	Original	3.75 × 14	No	No

Pt. No., patient number; PED, Pipeline Embolization Device; IPH, intraparenchymal hemorrhage.
*Aneurysms 11 and 12 were both covered by a single device.
†Aneurysms 23 and 24 were treated with separate PEDs using the same catheter assembly.

characteristics of the patients who presented with SAH are listed in Table 3.

Of the 37 aneurysms, 13 (35%) were located in the basilar trunk, 2 (5%) in the basilar tip, 4 (11%) in the vertebral artery (VA) proximal to the posterior inferior cerebellar artery (PICA), 10 (24%) in VA at the PICA origin, and 8 (24%) in the post-PICA VA. One basilar trunk aneurysm also involved 1 VA and both P1 segments (patient 17; aneurysm 18). Of the 37 aneurysms, 19 were saccular aneurysms (51%), with a mean size of 7.7 mm (range, 1.7—38.0); 17 were fusiform aneurysms (46%), with a mean size of 11.0 mm (range, 4.3—34) and length of 9.9 mm (range, 2.5—32.6); and 1 was a blister aneurysm, with a size of 2.9 mm. Seven were dissecting aneurysms (19%). Branch vessels were incorporated into 14 of the 37 aneurysms (38%). Four aneurysms (patients 19, 32, 33, and 35) had been previously coiled. No other aneurysms had been previously treated.

Clinical and Angiographic Outcomes

The overall procedural complication rate was 14% (5 of 36 sessions), including 2 thromboembolic events (5.6%) and 1 intraparenchymal hemorrhage (2.8%). Patient 15 developed right hemiparesis on postoperative day 1 secondary to VA PED thrombosis. However, the patient recovered to a mRS score 1 by the 6-month follow-up examination. Patient 22 developed a right middle cerebellar peduncle perforator infarction on postoperative day 1 that resulted in diplopia, vertigo, and ataxia. This patient also recovered to an mRS score of 1 within 6 months. Patient 6 experienced a fatal brainstem hemorrhage 1 day after discharge. This patient was receiving DAPT with aspirin and clopidogrel (140 PRUs) while also undergoing conversion to warfarin with an enoxaparin bridge for atrial fibrillation. Patient 10's treatment was complicated by fracture of the PED delivery guidewire, which was not retrieved but caused no clinical sequelae. Patient 29 developed a rectus sheath hematoma on postoperative day 2 that required a blood transfusion. VerifyNow (Accumetrics) testing at that time returned 12 PRUs. Clopidogrel was temporarily withheld until the PRUs were >60 and then resumed at a lower dose.

Angiographic outcomes are summarized in Table 1. Illustrative cases are presented in Figures 1 and 2. Excluding the aneurysm of the patient who died, angiographic follow-up data were available for 34 of the 35 aneurysms (97%). The mean follow-up duration was 14 months (range, 3—59). At the last angiographic follow-up examination, 24 of the 34 aneurysms (71%) were completely occluded and 29 (85%) were completely or near-completely occluded. The 8.9-mm fusiform basilar trunk aneurysm of patient 10 was initially treated with a single PED but had only decreased slightly in size at the 15-month follow-up examination. A second PED was subsequently placed, and no angiographic follow-up examination was obtained since the second procedure. Patient 27's VA aneurysm was treated with endovascular parent artery sacrifice after the 11-month follow-up examination showed that the aneurysm had increased in size by 66%. The angiographic outcomes subdivided by aneurysm size, location, and morphology are presented in Table 4. One PED within a VA thrombosed on postoperative day 1, as previously described (patient 15). Three additional cases of clinically insignificant PED stenosis were documented that caused <50% luminal narrowing (patients 23, 26, and 28). No cases of aneurysm recanalization were found on the follow-up imaging studies after occlusion.

A total of 46 branch vessels in 28 patients were covered by ≥1 PED, including 5 posterior cerebral arteries (PCAs), 13 uperior cerebellar arteries, 10 anterior inferior cerebellar arteries, and 18 PICAs. At the last angiographic follow-up examination, 2 of these 46 branch vessels (4.3%) were occluded. One of these was a large PICA arising from the thrombosed VA in patient 15. The other was an occluded P1 segment that was asymptomatic, because the distal PCA received flow from a posterior communicating artery (patient 21). Also observed were clinically asymptomatic stenosis of >75% in 3 covered PICAs (patients 16, 28, and 33) and 20% stenoses in bilateral covered superior cerebellar arteries (patient 11).

The clinical outcomes of patients presenting with SAH are summarized in Table 2. Hydrocephalus requiring external ventricular drainage developed in 2 of 7 patients (29%), neither of whom experienced related bleeding complications. No patients required ventriculoperitoneal shunts. Of the 6 patients, 5 had an mRS score of ≤2 at the last clinical follow-up visit. Patient 20, who had presented with a brainstem stroke, did not improve clinically after treatment and, ultimately, died. Patient 34, who had been treated for a dissecting pseudoaneurysm caused by a gunshot wound recovered to an mRS score of 0.

Table 2. Summary of Patient and Aneurysm Characteristics and Angiographic Outcomes After Pipeline Embolization of Vertebrobasilar Aneurysms

Pt. No.	Procedure No.	Aneurysm No.	Age, Gender	Location	Morphology	Size (mm)	Neck (mm)	Length (mm)	Incorporated Branch Vessels	Angiographic Follow-Up (months)	Angiographic Outcome
1	1	1	53, F	Vertebral, post-PICA	Saccular	5.7	4.1	NR	NR	6	Complete
2	2	2	47, M	Vertebral, pre-PICA	Saccular	38	17	NR	NR	6	Complete
3	3	3	57, F	Vertebral, PICA origin	Saccular	9.3	8.0	NR	PICA	19	Near-complete
4	4	4	52, F	Vertebral, pre-PICA	Saccular, dissecting	4.9	4.9	NR	NR	0	No follow-up
5	5	5	75, M	Vertebral, post-PICA	Saccular	10.8	6.7	NR	NR	6	Complete
6	6	6	60, M	Vertebral, PICA origin	Fusiform	7.0	NR	5.3	PICA	Died	Died
7	7	7	51, F	Vertebral, post-PICA	Fusiform	6.0	NR	6.0	NR	6	Complete
8	8	8	46, M	Vertebral, PICA origin	Fusiform	11.8	NR	6.0	PICA	6	Residual
9	9	9	62, F	Vertebral, post-PICA	Saccular	15	9.0	NR	NR	12	Complete
10	10	10	51, F	Basilar trunk	Fusiform, dissecting	8.9	NR	10.0	NR	15	Residual
10	11	10								0	No follow-up
11	12	11	43, F	Basilar tip	Saccular	3.9	1.6	NR	SCA	13	Complete
11	12	12		Basilar trunk	Saccular	2.0	1.2	NR	NR	13	Complete
12	13	13	69, M	Vertebral, post-PICA	Saccular	4.8	4.0	NR	NR	12	Complete
13	14	14	40, F	Basilar trunk	Fusiform, dissecting	8	NR	9.0	NR	6	Near-Complete
14	15	15	48, F	Vertebral, post-PICA	Saccular, dissecting	7.0	5.0	NR	NR	18	Complete
15	16	16	38, F	Vertebral, pre-PICA	Fusiform	10	NR	10.0	NR	3	Complete
16	17	17	32, F	Vertebral, PICA origin	Fusiform	7.0	NR	10.0	PICA, ASA	4	Complete
17	18	18	61, M	Basilar trunk	Fusiform	15	NR	26.0	BL AICA, BL SCA	12	Near-complete
18	19	19	56, F	Basilar trunk	Saccular	1.7	0.6	NR	NR	6	Complete
19	20	20	69, F	Basilar tip	Saccular	6.0	6.9	NR	NR	59	Complete
20	21	21	78, M	Basilar trunk	Fusiform	34	NR	32.6	BL AICA, BL SCA	Died	Died
21	22	22	51, F	Basilar trunk	Fusiform, dissecting	4.3	NR	3.4	AICA	6	Complete
22	23	23	75, M	Basilar trunk	Fusiform	18.1	NR	18.1	AICA	7	Residual
22	23	24		Basilar trunk	Blister	2.9	2.9	NR	NR	7	Complete
23	24	25	13, F	Basilar trunk	Fusiform, dissecting	8.3	NR	8.3	NR	12	Complete
24	25	26	67, F	Basilar trunk	Saccular	4.9	4.1	NR	NR	12	Residual
25	26	27	44, F	Basilar trunk	Saccular	2.6	2.0	NR	NR	6	Complete
26	27	28	50, F	Basilar trunk	Saccular	4.7	3.5	NR	NR	6	Complete
27	28	29	44, F	Vertebral, post-PICA	Fusiform, dissecting	6.3	NR	5.4	PICA	26	Complete

28	29	30	54, F	Vertebral, PICA origin	Saccular	11.5	6.0	NR	PICA	5.1	Complete
29	30	31	64, F	Vertebral, PICA origin	Saccular	10.0	6.0	NR	PICA	3	Near-complete
30	31	32	59, M	Vertebral, post-PICA	Fusiform	6.0	NR	2.5	NR	14	Complete
31	32	33	62, F	Vertebral, PICA origin	Fusiform	13.0	NR	12.0	NR	12	Complete
32	33	34	57, F	Vertebral, PICA origin	Fusiform	6.0	NR	3.0	PICA	22.3	Residual
33	34	35	53, F	Vertebral, PICA origin	Saccular	4.0	4.0	NR	NR	9	Complete
34	35	36	44, F	Vertebral, pre-PICA	Fusiform	7.0	NR	2.5	NR	27	Complete
35	36	37	54, M	Vertebral, PICA origin	Saccular	3.0	3.0	NR	PICA	52	Near-complete

Pt. No., patient number; F, female; PICA, posterior inferior cerebellar artery; NR, not reported; M, male; SCA, superior cerebellar artery; ASA, anterior spinal artery; BL, bilateral; AICA, anterior inferior cerebellar artery.

DISCUSSION

Several studies have demonstrated that posterior circulation aneurysms have a greater risk of spontaneous rupture than those in the anterior circulation.[13-15] For example, a 2007 meta-analysis that included 6556 unruptured aneurysms found that a location within the posterior circulation was associated with a relative risk of 2.5 (95% confidence interval [CI], 1.6–4.1).[13] The natural history of fusiform vertebrobasilar aneurysms has been especially poor, as demonstrated in a recent systematic review. That review found a 0.4% overall annual mortality rate that increased to 6.1% when aneurysms showed subsequent growth. The prognosis was especially poor for symptomatic patients, with a 5.9% annual risk of recurrent stroke and 5-year mortality rate of 40% when presenting with symptoms related to brainstem compression.[16]

Endovascular treatment of vertebrobasilar aneurysms is generally preferred to microsurgical clipping owing to multiple studies showing worse outcomes after surgical treatment.[17,18] However, ~10%–20% of coiled aneurysms require retreatment,[19-22] and the recurrence rate is ~3 times greater in aneurysms >10 mm with a neck >4 mm.[21] Additionally, complex vertebrobasilar aneurysms, such as fusiform aneurysms with incorporated branch vessels, often cannot be safely and adequately treated using conventional endovascular techniques.[23]

Several studies have reported encouraging initial results with PED usage in the posterior circulation, including a meta-analysis by Wang et al.[10] of 14 studies that found a 6-month angiographic occlusion rate of 84%. However, the effectiveness of the PED in the posterior circulation can vary according to aneurysm location, size, morphology, and clinical presentation. For example, a prospective series by Phillips et al.[24] of posterior circulation aneurysms treated with the PED reported an overall 6-month occlusion rate of 86% (24 of 28). However, the complete aneurysm occlusion rates were 60% (9 of 15) for basilar aneurysms and 89% (8 of 9) for VA aneurysms. The sizes and morphologies of the individual aneurysms in that series were not reported.[24] Liang et al.[25] published a series composed mostly of VA aneurysms (33 of 38 [87%]) of medium size (mean, 13.7 ± 6.4 mm), in which a 92% complete occlusion rate (35 of 38) was achieved. The investigators found that vertebrobasilar junction aneurysms tended to occlude at a slower rate than VA aneurysms, and no vertebrobasilar junction aneurysms were occluded within 6 months of treatment.[25] In contrast, Bhogal et al.[26] published a case series of 58 nonsaccular posterior circulation aneurysms using the PED or p64 (Phenox, Bochum, Germany). Most were vertebrobasilar (n = 26 [45%]) or basilar (n = 11 [19%]) aneurysms. They achieved an overall complete occlusion rate of only 57% (27 of 47).[26] A recent retrospective analysis by Griessenauer et al.[27] of 131 posterior circulation aneurysms treated with the PED reported comparable occlusion rates for fusiform and saccular aneurysms (37 of 52 [71%] vs. 37 of 47 [79%]). Univariate analysis did not identify size, location, or aneurysm rupture status as a significant predictor of complete or near-complete

Table 3. Ruptured Vertebrobasilar Aneurysms Treated with Pipeline Embolization

Pt. No.	Aneurysm No.	Location	Morphology	Size (mm)	Hunt-Hess Grade	mRS Score	EVD	Follow-Up mRS Score
4	4	Pre-PICA vertebral	Saccular, dissecting	4.9	2	4	Yes	4
10	10	Basilar trunk	Fusiform	8.9	1	2	No	0
13	14	Basilar trunk	Fusiform, dissection	8.0	3	2	Yes	2
14	15	Post-PICA vertebral	Saccular, dissecting	7.0	4	4	No	1
18	19	Basilar trunk	Saccular	1.7	2	1	No	2
30	32	Post-PICA vertebral	Fusiform	6.0	2	3	No	0

Pt. No., patient number; mRS, modified Rankin scale; EVD, external ventricular drain; PICA, posterior inferior cerebellar artery.

occlusion. However, these subgroup analyses were limited by small sample sizes.[27] The present series showed a complete aneurysm occlusion rate of 71% (24 of 34) and 85% rate of complete or near-complete occlusion (29 of 34), comparable to previous series. The aneurysm subgroups were small; however, greater occlusion rates were observed for saccular and VA aneurysms. These differences in occlusion rates might influence treatment plans, and additional data with larger aneurysm subgroups are needed to better quantify the differences in PED effectiveness.

Available data also suggest that the safety of PED treatment is influenced by aneurysm size, morphology, and location. An analysis of the IntrePED multicenter registry included 95 posterior circulation aneurysms (44 basilar artery and 33 VA aneurysms).[28] The neurologic morbidity and mortality rate for fusiform aneurysms was 27% compared with 3.9% and 12% for dissecting and saccular aneurysms, respectively. On multivariate analysis, aneurysm size (hazard ratio [HR], 1.11; 95% CI, 1.04–1.18; P = 0.0008), fusiform shape (HR, 4.93; 95% CI, 1.29–21.73; P = 0.0066), and the use of ≥3 PEDs (HR, 10.0; 95% CI, 2.65–43.38; P <0.0001) were significant predictive factors for combined neurological morbidity and mortality.[28] In the series by Griessenauer et al.,[27] fusiform and saccular aneurysm treatment resulted in comparable rates of thromboembolic (25% [13 of 52] and 23% [11 of 47], respectively) and hemorrhagic (7.7% [4 of 52] and 10% [5 of 47], respectively) complications. However, fusiform aneurysms involving the basilar artery were associated with a 36% (10 of 28) complication rate compared with 16% (3 of 19) for VA aneurysms. Corroborating these findings, the 3 complications

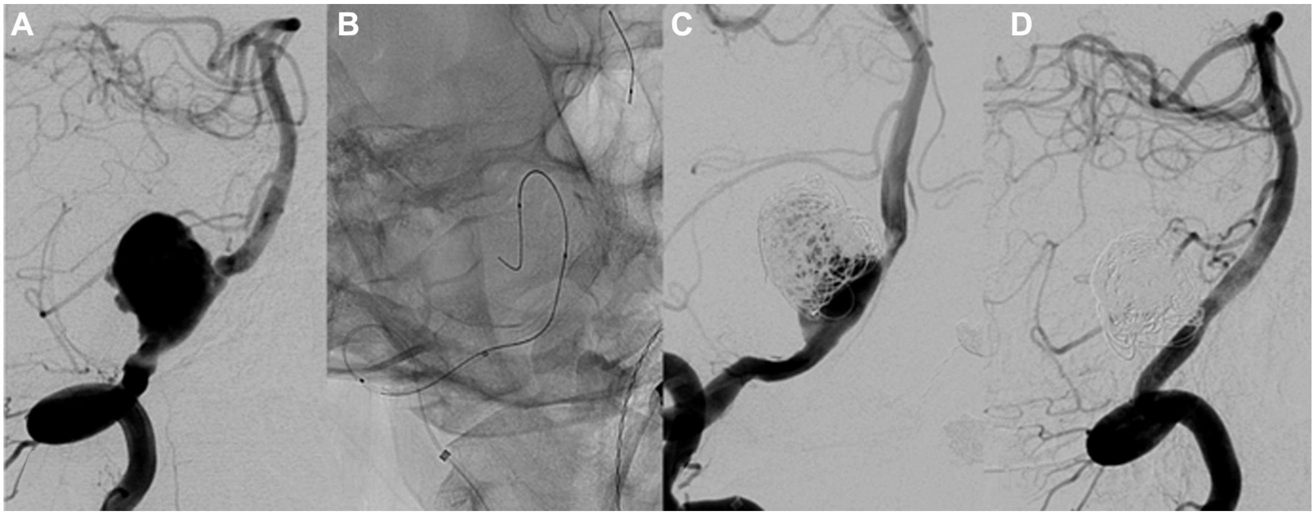

Figure 1. Angiographic views of patient 2, a 47-year-old man. (**A**) A giant right V4 segment vertebral artery aneurysm. (**B, C**) Pipeline embolization with adjunctive partial coiling was performed. (**D**) Follow-up digital subtraction angiography 6 months later showed complete occlusion of the aneurysm and remodeling of the dysplastic vertebral artery segment.

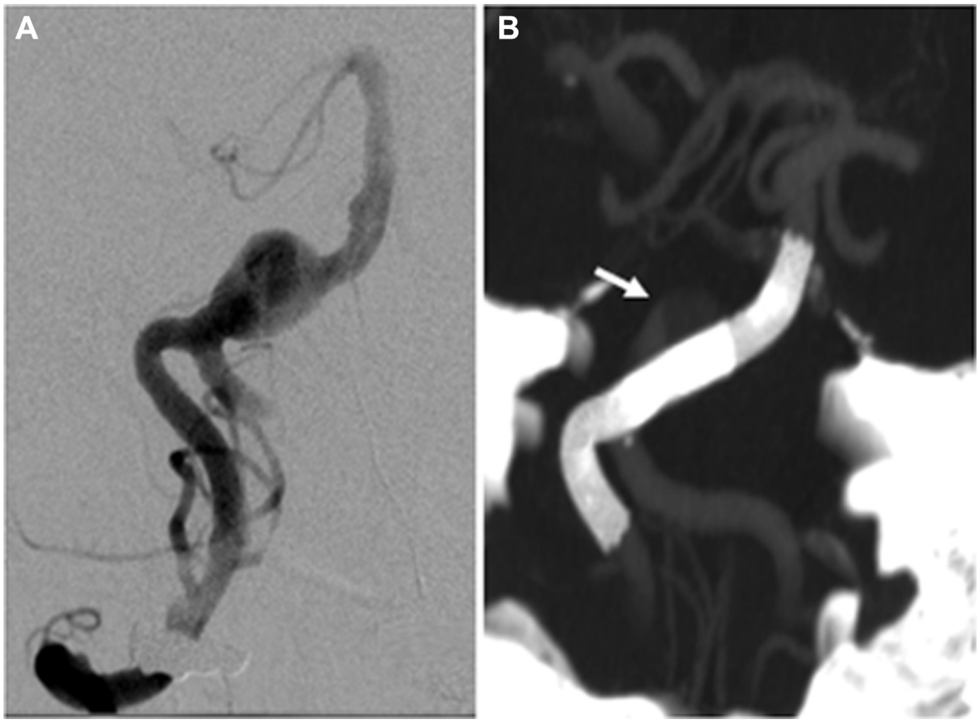

Figure 2. Angiographic views of patient 22, a 75-year-old man. (**A**) A large, fusiform basilar trunk aneurysm. Two overlapping Pipeline Embolization Devices were deployed across the aneurysmal segment of the basilar artery, with the proximal construct extending into the right vertebral artery. (**B**) However, a 7-month follow-up computed tomography angiogram showed residual aneurysm filling (*arrow*).

in our series all occurred in patients with a fusiform aneurysm, a 17% (3 of 18) rate of procedure-related neurologic morbidity in this subgroup.

An additional concern regarding PED treatment of posterior circulation aneurysms is coverage of major branch vessels and perforator arteries that supply the brainstem. In the present

Table 4. Complete or Near-Complete Occlusion Rates of Aneurysm Location, Size, and Morphology Subgroups

	Morphology			Location		
Variable	Saccular	Fusiform	Blister	Vertebral	Basilar	Total
Size (mm)						
<10	92 (12/13)	80 (8/10)	100 (1/1)	92 (11/12)	83 (10/12)	88 (21/24)
≥10	100 (5/5)	60 (3/5)	0 (0/0)	88 (7/8)	50 (1/2)	80 (8/10)
Morphology						
Saccular	-	-	-	100 (11/11)	71 (5/7)	89 (16/18)
Fusiform	-	-	-	78 (7/9)	83 (5/6)	80 (12/15)
Blister	-	-	-	0 (0/0)	100 (1/1)	100 (1/1)
Total	94 (17/18)	73 (11/15)	100 (1/1)	90 (18/20)	79 (11/14)	85 (29/34)

Data presented as % (n/N).

series, 46 major vertebrobasilar branch vessels were covered by a PED, of which 4.3% (2 of 46) were occluded at the angiographic follow-up examination. However, 1 of these cases was associated with parent artery thrombosis (patient 15). In the second patient, proximal occlusion of a covered PCA was asymptomatic because the distal PCA was supplied by an ipsilateral posterior communicating artery. This case is consistent with previous observations that arteries with collateralization can become occluded after PED coverage without clinical consequences.[29-31]

In addition to the retrospective design, similar to other studies of PED treatment of posterior circulation aneurysms, the present study was limited by cohort heterogeneity in terms of aneurysm characteristics, procedure techniques, and antiplatelet regimens. We attempted to mitigate the aneurysm characteristic heterogeneity with subgroup analyses. However, the number of cases in each subgroup was small.

CONCLUSION

Pipeline embolization of vertebrobasilar aneurysms is associated with acceptable occlusion and complication rates. However, the safety and effectiveness can be influenced by aneurysm size and morphology. Thus, studies with larger subgroups are needed to better quantify the differences in outcomes.

REFERENCES

1. Griessenauer CJ, Ogilvy CS, Foreman PM, et al. Pipeline Embolization Device for small intracranial aneurysms: evaluation of safety and efficacy in a multicenter cohort. *Neurosurgery*. 2017;80: 579-587.

2. Chalouhi N, Zanaty M, Whiting A, et al. Safety and efficacy of the Pipeline Embolization Device in 100 small intracranial aneurysms. *J Neurosurg*. 2015;122:1498-1502.

3. Saatci I, Yavuz K, Ozer C, Geyik S, Cekirge HS. Treatment of intracranial aneurysms using the Pipeline flow-diverter embolization device: a single-center experience with long-term follow-up results. *AJNR Am J Neuroradiol*. 2012;33:1436-1446.

4. Yu SC-H, Kwok C-K, Cheng P-W, et al. Intracranial aneurysms: midterm outcome of Pipeline Embolization Device—a prospective study in 143 patients with 178 aneurysms. *Radiology*. 2012;265: 893-901.

5. Lin L-M, Colby GP, Kim JE, Huang J, Tamargo RJ, Coon AL. Immediate and follow-up results for 44 consecutive cases of small (<10 mm) internal carotid artery aneurysms treated with the Pipeline Embolization Device. *Surg Neurol Int*. 2013;4:114.

6. Brinjikji W, Lanzino G, Cloft HJ, Siddiqui AH, Kallmes DF. Risk factors for hemorrhagic complications following Pipeline Embolization Device treatment of intracranial aneurysms: results from the international retrospective study of the Pipeline Embolization Device. *AJNR Am J Neuroradiol*. 2015;36:2308-2313.

7. Kallmes DF, Hanel R, Lopes D, et al. International retrospective study of the Pipeline Embolization Device: a multicenter aneurysm treatment study. *AJNR Am J Neuroradiol*. 2015;36:108-115.

8. Brinjikji W, Murad MH, Lanzino G, Cloft HJ, Kallmes DF. Endovascular treatment of intracranial aneurysms with flow diverters: a meta-analysis. *Stroke*. 2013;44:442-447.

9. Delgado Almandoz JE, Kayan Y, Tenreiro A, et al. Clinical and angiographic outcomes in patients with intracranial aneurysms treated with the Pipeline Embolization Device: intra-procedural technical difficulties, major morbidity, and neurological mortality decrease significantly with increased operator experience in device deployment and patient management. *Neuroradiology*. 2017;59:1291-1299.

10. Wang C-B, Shi W-W, Zhang G-X, Lu H-C, Ma J. Flow diverter treatment of posterior circulation aneurysms: a meta-analysis. *Neuroradiology*. 2016; 58:391-400.

11. Siddiqui AH, Abla AA, Kan P, et al. Panacea or problem: flow diverters in the treatment of symptomatic large or giant fusiform vertebrobasilar aneurysms. *J Neurosurg*. 2012;116: 1258-1266.

12. Wallace AN, Grossberg JA, Almandoz JED, et al. Endovascular treatment of posterior cerebral artery aneurysms with flow diversion: case series and systematic review. *Neurosurgery*. 2018;83: 790-799.

13. Wermer MJH, van der Schaaf IC, Algra A, Rinkel GJE. Risk of rupture of unruptured intracranial aneurysms in relation to patient and aneurysm characteristics. *Stroke*. 2007;38: 1404-1410.

14. Greving JP, Wermer MJ, Brown RD, et al. Development of the PHASES score for prediction of risk of rupture of intracranial aneurysms: a pooled analysis of six prospective cohort studies. *Lancet Neurol*. 2014;13:59-66.

15. Wiebers DO, Whisnant JP, Huston J III, et al. International Study of Unruptured Intracranial Aneurysms Investigators. Unruptured intracranial aneurysms: natural history, clinical outcome, and risks of surgical and endovascular treatment. *Lancet*. 2003;362:103-110.

16. Shapiro M, Becske T, Riina HA, Raz E, Zumofen D, Nelson PK. Non-saccular vertebrobasilar aneurysms and dolichoectasia: a systematic literature review. *J Neurointerv Surg*. 2014;6:389-393.

17. Lusseveld E, Brilstra E, Nijssen P, et al. Endovascular coiling versus neurosurgical clipping in patients with a ruptured basilar tip aneurysm. *J Neurol Neurosurg Psychiatry*. 2002;73:591-593.

18. Spetzler RF, McDougall CG, Zabramski JM, et al. The Barrow ruptured aneurysm trial: 6-year results. *J Neurosurg*. 2015;123:609-617.

19. Raymond J, Guilbert F, Weill A, et al. Long-term angiographic recurrences after selective endovascular treatment of aneurysms with detachable coils. *Stroke*. 2003;34:1398-1403.

20. Ferns SP, Sprengers MES, van Rooij WJ, et al. Coiling of intracranial aneurysms: a systematic review on initial occlusion and reopening and retreatment rates. *Stroke*. 2009;40:e523-e529.

21. Ries T, Siemonsen S, Thomalla G, Grzyska U, Zeumer H, Fiehler J. Long-term follow-up of cerebral aneurysms after endovascular therapy prediction and outcome of retreatment. *AJNR Am J Neuroradiol*. 2007;28:1755-1761.

22. Lozier AP, Connolly ES, Lavine SD, Solomon RA. Guglielmi detachable coil embolization of posterior circulation aneurysms: a systematic review of the literature. *Stroke*. 2002;33:2509-2518.

23. Coert BA, Chang SD, Do HM, Marks MP, Steinberg GK. Surgical and endovascular management of symptomatic posterior circulation fusiform aneurysms. *J Neurosurg*. 2007; 106:855-865.

24. Phillips TJ, Wenderoth JD, Phatouros CC, et al. Safety of the Pipeline Embolization Device in treatment of posterior circulation aneurysms. *AJNR Am J Neuroradiol*. 2012;33:1225-1231.

25. Liang F, Zhang Y, Guo F, et al. Use of Pipeline Embolization Device for posterior circulation aneurysms: single-center experiences with comparison with anterior circulation aneurysms. *World Neurosurg*. 2018;112:e683-e690.

26. Bhogal P, Pérez MA, Ganslandt O, Bäzner H, Henkes H, Fischer S. Treatment of posterior circulation non-saccular aneurysms with flow diverters: a single-center experience and review of 56 patients. *J Neurointerv Surg*. 2017;9: 471-481.

27. Griessenauer CJ, Ogilvy CS, Adeeb N, et al. Pipeline embolization of posterior circulation aneurysms: a multicenter study of 131 aneurysms. *J Neurosurg*. 2018;1:1-13.

28. Lopes DK, Jang D-K, Cekirge S, et al. Morbidity and mortality in patients with posterior circulation aneurysms treated with the Pipeline Embolization Device: a subgroup analysis of the international retrospective study of the pipeline embolization device. *Neurosurgery*. 2018;83: 488-500.

29. Yavuz K, Geyik S, Saatci I, Cekirge HS. Endovascular treatment of middle cerebral artery aneurysms with flow modification with the use of the Pipeline Embolization Device. *AJNR Am J Neuroradiol*. 2014;35:529-535.

30. Rangel-Castilla L, Munich SA, Jaleel N, et al. Patency of anterior circulation branch vessels after Pipeline embolization: longer-term results from 82 aneurysm cases. *J Neurosurg*. 2017;126:1064-1069.

31. Vedantam A, Rao VY, Shaltoni HM, Mawad ME. Incidence and clinical implications of carotid branch occlusion following treatment of internal carotid artery aneurysms with the Pipeline Embolization Device. *Neurosurgery*. 2015;76:173-178.

Conflict of interest statement: Josser E. Delgado Almandoz is a consultant for Medtronic, Microvention, Accriva, Penumbra, and Sequent. Yasha Kayan is a consultant for Medtronic, Microvention, and Penumbra. Christopher J. Moran is a consultant for Medtronic and Microvention. Jacques E. Dion is employed by Microvention. The remaining authors declare that the article content was composed in the absence of any commercial or financial relationships that could be construed as a potential conflict of interest.

Received 25 June 2018; accepted 13 December 2018

Citation: World Neurosurg. (2019) 124:e460-e469.
https://doi.org/10.1016/j.wneu.2018.12.116

Journal homepage: **www.journals.elsevier.com/world-neurosurgery**

Available online: **www.sciencedirect.com**

1878-8750/$ - see front matter © 2019 Elsevier Inc. All rights reserved.
