# Supplementary material for: Management strategies and outcomes of basilar trunk aneurysms: a systematic review and meta-analysis
Source: Langenbecks Arch Surg. 2026 Jan 20;411(1):71. doi: 10.1007/s00423-025-03959-3 (PMC12852281; doi:10.1007/s00423-025-03959-3)
Supplement: Supplementary file 1 — Supplementary file1 (ZIP 55327 KB) [file 423_2025_3959_MOESM1_ESM.zip › literature.zip!/meta/Wang 2021.pdf]

frontiers in Neurology

ORIGINAL RESEARCH
published: 15 February 2021
doi: 10.3389/fneur.2021.625909

Check for updates

Endovascular Therapy for Basilar Arterial Trunk Aneurysms

*Yiheng Wang[1], Kan Xu[1], Jia Song[2] and Jinlu Yu[1]**

[1] Department of Neurosurgery, The First Hospital of Jilin University, Changchun, China, [2] Department of Neurology, The First Hospital of Jilin University, Changchun, China

Background: Although aneurysms rarely occur in the basilar artery (BA) trunk, the majority of those that do are dissection aneurysms. Currently, the mainstream therapy for BA trunk aneurysms is endovascular therapy (EVT), which mainly includes single coiling or conventional low-metal-coverage stent-assisted EVT, but the efficacy remains to be evaluated.

Methods: A retrospective study was performed for the patients who were admitted to our institution for BA trunk aneurysms and underwent EVT. A total of 28 patients were collected in this study.

Results: The patients were aged 23–71 years (53.7 ± 11.5 years on average); nine were female (32.1%, 9/28), and 19 were male (67.9%, 19/28). The patients were given single coiling or conventional low-metal-coverage stent-assisted EVT. Among the 28 patients, 10 (35.7%, 10/28) developed complications, 90% (9/10) of which were ischemic and 10% (1/10) were hemorrhagic. Among the 28 patients, 5 (17.9%, 5/28) died. The surviving 23 patients (82.1%, 23/28) recovered well.

Conclusions: This study found that for BA trunk aneurysms, single coiling or conventional low-metal-coverage stent-assisted EVT still had some risks. The risks are mainly from brainstem ischemia. Therefore, the perforators of the BA trunk must be carefully evaluated and prevented from receiving damage from the EVT procedure. This study also shows that 82.1% of patients recovered well. Therefore, EVT can result in an acceptable prognosis.

Keywords: basilar artery, trunk, aneurysm, endovascular therapy, prognosis

OPEN ACCESS

Edited by:
Yisen Zhang,
Capital Medical University, China

Reviewed by:
Basil Erwin Grüter,
Aarau Cantonal Hospital, Switzerland
Miao Li,
China-Japan Union Hospital of Jilin University, China

Correspondence:
Jinlu Yu
jlyu@jlu.edu.cn;
jinluyu@hotmail.com
orcid.org/0000-0003-2329-7946

Specialty section:
This article was submitted to Endovascular and Interventional Neurology,
a section of the journal Frontiers in Neurology

Received: *12 November 2020*
Accepted: *25 January 2021*
Published: *15 February 2021*

Citation:
Wang Y, Xu K, Song J and Yu J (2021) Endovascular Therapy for Basilar Arterial Trunk Aneurysms. Front. Neurol. 12:625909. doi: 10.3389/fneur.2021.625909

INTRODUCTION

Although aneurysms rarely occur in the basilar artery (BA) trunk, some are routine saccular aneurysms at the initial part of the perforating branch (1), while most are dissection aneurysms involving part of the BA trunk (2). Since the BA trunk is anatomically deep, craniotomy clipping of BA aneurysms is a high-risk procedure. Instead, endovascular therapy (EVT) is mainly used for treatment today (2, 3).

Moreover, the BA trunk is rich in branches, so the application of a flow-diverting stent will lead to the brainstem perforator-related infarction (4). Therefore, single coiling or conventional low-metal-coverage stent-assisted EVT is still mainly used for BA trunk aneurysms. However, is single coiling or conventional low-metal-coverage stent-assisted EVT also very safe for BA trunk aneurysms? Does it have a high incidence of complications?

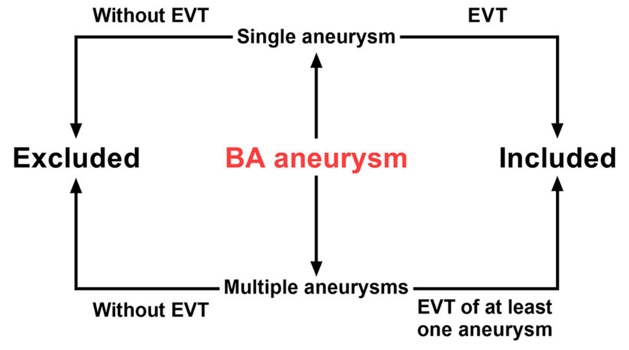

FIGURE 1 | Flow chart of inclusion and exclusion criteria. BA, basilar artery; EVT, endovascular therapy.

In this report, 28 patients with BA trunk aneurysms who were treated with single coiling or conventional low-metal-coverage stent-assisted EVT in our center over the past 5 years were enrolled to evaluate the effect. The results are summarized and reported and will be of great importance as a reference.

MATERIALS AND METHODS

A retrospective study was performed for patients who were admitted to The First Hospital of Jilin University diagnosed with BA trunk aneurysms from January 2015 to January 2020. This study was approved by the institutional ethics committee (No. 2020-588).

Inclusion and Exclusion Criteria

Patients with aneurysms located in the BA trunk and treated with EVT were included. These included EVT performed for a single aneurysm and EVT of at least one aneurysm performed for multiple aneurysms. In addition, patients with aneurysms located at the BA tip or with aneurysms involving the vertebral artery (VA) were excluded. A flow chart is shown in **Figure 1**.

Aneurysm Site and Classification

The length of the BA ranges from 20–40 mm. In terms of basilar artery infarction, BA can be subdivided anatomically into three segments: the inferior segment from the VA to the anterior inferior cerebellar arteries (AICA), the middle segment from the AICA to the origin of the superior cerebellar arteries (SCA) and the superior segment from the SCA to the terminal posterior cerebral arteries (PCA) (5). For BA trunk aneurysms, the segmentation can be referential. However, while the inferior segment was reasonable, the superior segment was too short. Consequently, it is reasonable to divide the part between the BA termination and the AICA into superior and middle segments.

Therefore, in our study, the BA trunk aneurysms were classified into superior, middle and inferior aneurysms according to their anatomical location within the BA trunk, then into saccular, spherical and fusiform aneurysms according to the morphology. The saccular aneurysms were further divided into narrow-necked and wide-necked aneurysms, and the spherical and fusiform aneurysms were divided into lateral and annular aneurysms according to their location within the BA.

EVT Scheme

BA aneurysms were divided into single coiling or low-metal-coverage stent-assisted EVT depending on their treatment.

EVT Evaluation

The effects of EVT were recorded and evaluated with the Modified Raymond-Roy Classification (MRRC) (6). Complications and follow-up results were recorded. The modified Rankin Scale (mRS) was used to grade the results (7).

RESULTS

General Information

The 28 patients were aged 23–71 years old (53.7 ± 11.5 years on average). Among them, nine were female (32.1%, 9/28), and 19 were male (67.9%, 19/28). Among the 28 patients, 13 (46.4%, 13/28) were found to have aneurysms by asymptomatic physical examination, and 5 (17.9%, 5/28) were found to have aneurysms by cerebral infarction examination. Ten patients (35.7%, 10/28) presented with subarachnoid hemorrhage (SAH). There were 3 cases of Grade I, two cases of Grade II and five cases of Grade III SAH according to the Hunt-Hess classification.

Image Characteristics

Among 28 patients, there were 29 BA trunk aneurysms; one patient had two aneurysms. The 29 aneurysms were 1–22 mm in diameter (7.2 ± 6.1 mm on average). Three were superior aneurysms (10.3%, 3/29), 10 were middle aneurysms (34.5%, 10/29), and 16 were inferior aneurysms (55.2%, 16/29) (six of which were located within five fenestrations).

With regard to morphology, there were 21 saccular aneurysms (72.4%, 21/29) (including 17 wide-necked and four narrow-necked saccular aneurysms), five spherical aneurysms (17.2%, 5/29) (three lateral and two annular spherical aneurysms around the BA), and three fusiform aneurysms (10.3%, 3/29) (two lateral and one annular aneurysm around the BA).

EVT Scheme

Among the 29 aneurysms, one aneurysm in a case with two aneurysms was monitored conservatively, and the remaining 28 aneurysms in 28 patients were treated with EVT. Among the 28 treated aneurysms, four were treated by single coiling (14.3%, 4/28), and 24 were treated with low-metal-coverage stent-assisted embolization (85.7%, 24/28). Twenty-three of the EVT-treated aneurysms were MRRC Grade I (82.1%, 23/28), and five were MRRC Grade IIIa (17.9%, 5/28).

EVT Results

Perioperative Results

Of the 28 EVT-treated patients, 18 recovered well (64.3%, 18/28), and 10 patients developed complications (35.7%, 10/28).

TABLE 1 | Clinical Data of BA trunk aneurysm.

No.	Age/sex	Onset	HH Grade	Site	Morphology	Neck	Size	EVT	Immediate MRRC	Complications	Onset time of complication	Recovery time of complication	Follow-up time	Follow-up MRRC	Others	mRS
1	34/M	Occasional	NA	Superior	Spherical (lateral)	NA	13.5 mm	Stent + coil	I	Perforator ischemia/ hemiplegia	Immediate	1 month	9 months	I	N	0
2	60/F	Occasional	NA	Inferior	Saccular	Narrow	3.5 mm	Stent + coil	I	N	NA	NA	6 months	I	N	0
3	54/F	SAH	3	Inferior	Saccular	Wide	4.5 mm	Stent + coil	I	BA ischemia/ coma	Immediate	NA	NA	NA	NA	Death
4	68/M	SAH	3	Inferior	Fusiform (annular)	NA	20 mm	Stent + coil	IIIa	Perforator ischemia/ hemiplegia	2 h postoperatively	6 months	2 years	N	N	0
5	60/F	SAH	2	Inferior (fenestration)	Saccular (2)	Both narrow	1.5 mm & 3 mm	Coil (Large aneurysm)	I and observation	N	NA	NA	10 months	N	Hydrocephalus shunting	1
6	46/M	SAH	1	Middle	Saccular	Wide	8 mm	Stent + coil	I	Perforator ischemia/ hemiplegia	2 hours postoperatively	9 days	2 years	N	N	0
7	63/F	Occasional	NA	Middle	Spherical (annular)	NA	12 mm	Stent + coil	IIIa	N	NA	NA	1 year	IIIa	N	0
8	63/M	Occasional	NA	Inferior	Saccular	Wide	3.5 mm	Stent + coil	I	N	NA	NA	1 year	I	N	0
9	61/M	Occasional	NA	Superior	Saccular	Narrow	10 mm	Coil	I	N	NA	NA	2 years	I	N	0
10	38/M	Occasional	NA	Inferior	Saccular	Wide	11.5 mm	Stent + coil	I	N	NA	NA	2 years	I	N	0
11	59/M	Cerebral infarction	NA	Inferior	Saccular	Wide	5.5 mm	Stent + coil	I	N	NA	NA	6 months	I	N	0
12	59/M	Occasional	NA	Inferior	Spherical (lateral)	NA	22 mm	Coil	IIIa	Aneurysm rupture/coma	Postoperative 1 month	NA	NA	NA	NA	Death
13	53/M	Cerebral infarction	NA	Middle	Saccular	Wide	4 mm	Stent + coil	I	N	NA	NA	6 months	I	N	0
14	67/M	Cerebral infarction	NA	Middle	Saccular	Wide	5 mm	Stent + coil	I	N	NA	NA	1 and a half year	N	N	0
15	52/M	Cerebral infarction	NA	Middle	Saccular	Wide	2.5 mm	Stent + coil	IIIa	N	NA	NA	6 months	IIIa	N	0
16	66/M	Cerebral infarction	NA	Middle	Fusiform (lateral)	NA	4.5 mm	Stent + coil	I	N	NA	NA	1 and a half year	N	N	0
17	49/M	SAH	3	Inferior	Saccular	Wide	2 mm	Stent + coil	I	BA ischemia/ coma	Postoperative 20 days	NA	NA	NA	NA	Death
18	54/M	Occasional	NA	Middle	Saccular	Wide	5 mm	Stent + coil	I	N	NA	NA	1 and a half year	N	N	0
19	56/M	Occasional	NA	Inferior	Spherical (annular)	NA	20 mm	Stent + coil	IIIa	N	NA	NA	6 months	I	N	1
20	49/F	Occasional	NA	Inferior (fenestration)	Saccular	Wide	5.5 mm	Coil	I	N	NA	NA	1 year	N	N	0

(Continued)

TABLE 1 | Continued

No.	Age/sex	Onset	HH Grade	Site	Morphology	Neck	Size	EVT	Immediate MRRC	Complications	Onset time of complication	Recovery time of complication	Follow-up time	Follow-up MRRC	Others	mRS
21	28/F	Occasional	NA	Inferior	Fusiform (lateral)	NA	4 mm	Stent + coil	I	N	NA	NA	1 and a half year	I	N	1
22	71/M	SAH	3	Middle	Saccular	Wide	2.5 mm	Stent + coil	I	BA ischemia/ coma	Immediate	NA	NA	NA	NA	Death
23	53/F	SAH	2	Inferior (fenestration)	Saccular	Wide	4 mm	Stent + coil	I	Perforator ischemia/ hemiplegia	Postoperative 2 days	7 days	2 years	N	N	0
24	23/F	SAH	3	Inferior (fenestration)	Saccular	Wide	5 mm	Stent + coil	I	Perforator ischemia/ hemiplegia	Postoperative 1 day	20 days	3 years	N	N	0
25	60/M	Occasional	NA	Middle	Saccular	Wide	4 mmm	Stent + coil	I	N	NA	NA	3 years	N	N	0
26	50/M	Occasional	NA	Superior	Spherical (lateral)	NA	18 mm	Stent + coil	I	Perforator ischemia/coma	Postoperative 6 months	NA	NA	NA	NA	Death
27	53/M	SAH	1	Middle	Saccular	Wide	2.5 mm	Stent + coil	I	N	NA	NA	1 year	N	N	0
28	54/F	SAH	1	Inferior (fenestration)	Saccular	Wide	8 mm	Stent + coil	I	N	NA	NA	6 months	N	N	0

EVT, endovascular therapy; F, female; HH grade, Hunt-Hess grade; M, male; MRRC, Modified Raymond-Roy Classification; mRS, modified Rankin Scale; N, No; NA, Not applicable; SAH, subarachnoid hemorrhage.

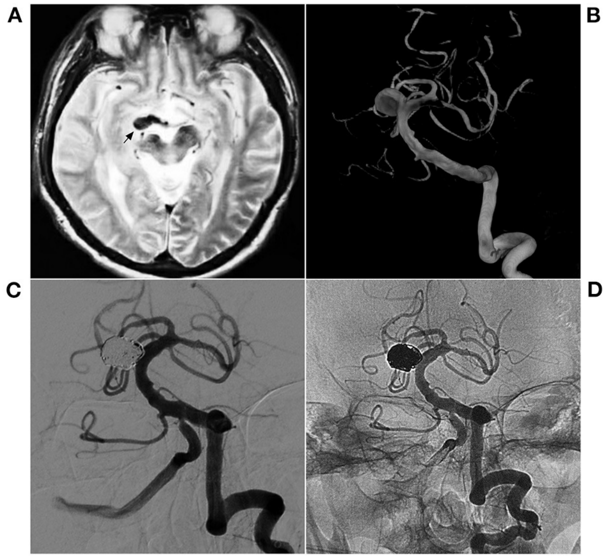

FIGURE 2 | EVT with single coiling of a narrow-neck aneurysm. **(A)** MR T2WI shows an aneurysm in the right midbrain (arrow); **(B)** 3D-reconstructed DSA shows a saccular aneurysm in the superior BA. **(C,D)** Postoperative VA-DSA shows the aneurysm following coiling embolization, MRRC Grade I. BA, basilar artery; DSA, digital subtraction angiography; EVT, endovascular therapy; MR, magnetic resonance; MRRC, Modified Raymond-Roy Classification; VA, vertebral artery.

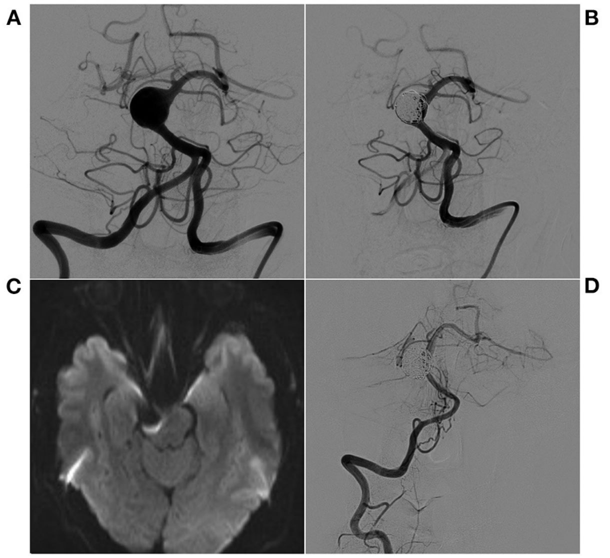

FIGURE 3 | EVT with stent-assisted coiling of a spherical aneurysm. **(A)** Preoperative VA-DSA shows a spherical aneurysm in the superior BA, eccentric type. **(B)** Postoperative VA-DSA shows the aneurysm with stent-assisted embolization, MRRC Grade I. **(C)** After EVT, the patient suffered hemiplegia, and an immediate MR diffusion sequence showed no infarctions. **(D)** Postoperative 9-month follow-up VA-DSA shows no recurrence of the aneurysm. BA, basilar artery; DSA, digital subtraction angiography; EVT, endovascular therapy; MR, magnetic resonance; MRRC, Modified Raymond-Roy Classification; VA, vertebral artery.

Complication

Among the 10 patients (35.7%, 10/28) who experienced complications, 5 (17.9%, 5/28) had hemiplegia due to perforation ischemia, 4 (14.3%, 4/28) developed coma due to perforation and BA ischemia, and 1 (3.6%, 1/28) died due to delayed aneurysm rupture. With regard to the onset of complications, three patients were stricken immediately after EVT, two at 2 h postoperatively, one at 1 day postoperatively, one at 2 days postoperatively, one at 20 days postoperatively, one at 1 month postoperatively, and one at 6 months postoperatively.

Concerning the prognoses of the complications in the 10 patients, in addition to the patient who died due to delayed aneurysm rupture, the four patients who developed coma caused by perforation and BA ischemia all died after the operation. The remaining five patients who experienced hemiplegia caused by perforating ischemia returned to normal between 7 days and 6 months after the operation without sequelae. One patient had hydrocephalus and underwent ventriculoperitoneal shunt implantation.

Long-Term Follow-Up

In addition to the five patients (17.9%, 5/28) who died of complications, the remaining 23 patients (82.1%, 23/28) were followed up for 6–36 months (16.0 ± 9.3 months on average). A total of 20 patients had an mRS score of 0, and three patients had an mRS score of 1, indicating good results. Among these 23 patients, 12 did not undergo follow-up imaging, while the other 11 underwent follow-up digital subtraction anisotropy (DSA). There were nine cases of Grade I and 2 cases of Grade IIIa aneurysms according to MRRC.

The clinical data of BA trunk aneurysms is summarized in **Table 1**. Several key parameters are included, such as the site, morphology and size of the aneurysm, the aneurysm neck, the immediate and follow-up EVT MRRC, the complications and mRS. Typical aneurysms and their EVT results are shown in **Figures 2**–7.

DISCUSSION

The BA trunk contains many perforators supplying the brainstem. Aneurysms rarely occur on the perforators; therefore, BA trunk aneurysms are more often dissection aneurysms rather than saccular bifurcation aneurysms (8). Imaging results of dissection aneurysms of the BA trunk suggest lateral saccular dissection and spherical or fusiform dissection (9). In this study, 72.4% of the aneurysms were characterized by a saccular dissection.

Most posterior circulation dissection aneurysms are stable, similar to VA dissection aneurysms, and rupture hemorrhage is rare (10). In this study, 35.7% of the BA trunk aneurysms ruptured and bled, indicating that BA trunk aneurysms are not always stable and can increase in size over time, as there is an increased risk of SAH formation (8). Therefore, it is necessary to apply appropriate treatment.

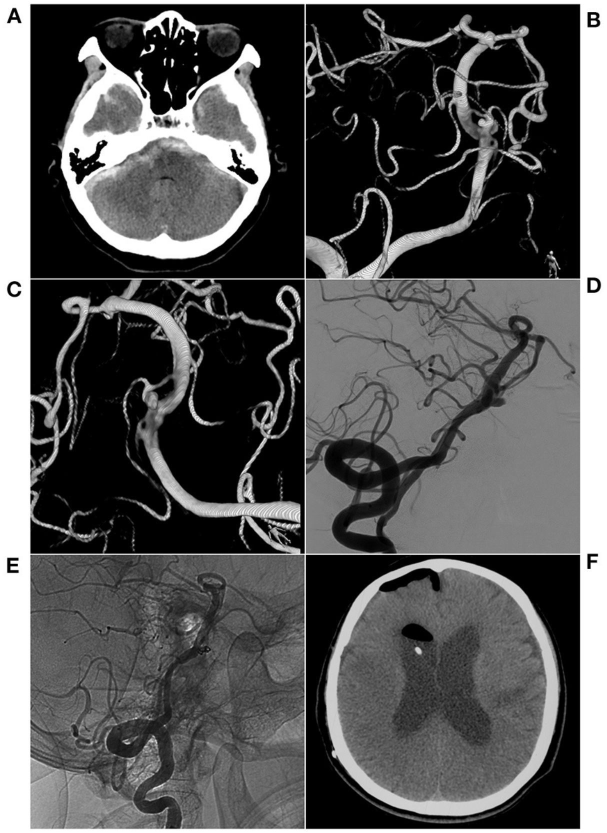

FIGURE 4 | EVT with single coiling of an aneurysm in the fenestration. **(A)** CT shows subarachnoid hemorrhage in the anterior brainstem; **(B,C)** 3D reconstructed DSA shows two fenestrations in the inferior BA and two aneurysms in the superior fenestration. **(D)** Preoperative VA-DSA shows a large aneurysm. **(E)** Postoperative VA-DSA images show a large aneurysm with simple coiling, MRRC Grade I. **(F)** The patient had hydrocephalus after EVT and underwent ventriculoperitoneal shunting at the 10-month follow-up. BA, basilar artery; CT, computed tomography; DSA, digital subtraction angiography; EVT, endovascular therapy; MRRC, Modified Raymond-Roy Classification; VA, vertebral artery.

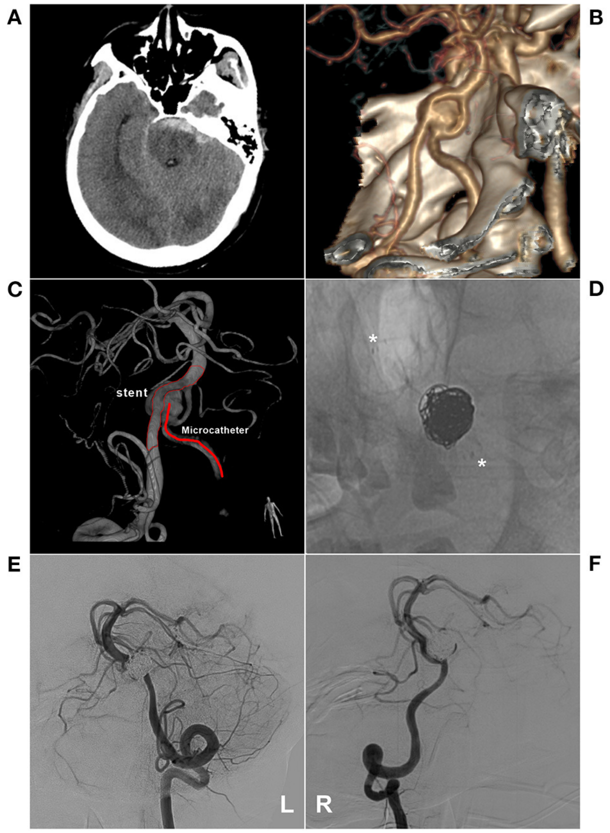

FIGURE 5 | EVT with stent-assisted coiling of an aneurysm in the fenestration. **(A)** CT shows subarachnoid hemorrhage at the front of the brainstem; **(B)** CTA shows an aneurysm in a fenestration of the inferior BA. **(C)** 3D-reconstructed DSA shows the aneurysm and EVT schemes. The stent was placed into a coarse VA and microcatheter-walked to a finer VA to embolize the aneurysm; **(D)** X-ray image shows coiling with stent-assisted EVT (asterisk); E-F: VA-DSA on the left **(E)** and right **(F)** side show the aneurysm embolization, MRRC Grade IIIa. BA, basilar artery; CT, computed tomography; CTA, CT angiography; DSA, digital subtraction angiography; EVT, endovascular therapy; MRRC, Modified Raymond-Roy Classification; VA, vertebral artery.

The BA trunk is relatively short, but it can still be divided into superior, middle and inferior segments, with corresponding aneurysms (11). BA trunk aneurysms occur most frequently in the inferior segment. Among the 28 aneurysms in this study, 55.2% occurred in the inferior BA trunk. This may be because the inferior BA often contains fenestrations, which are the predilection sites of aneurysm formation.

In the study by van Rooij et al. BA fenestrations were detected in 4% of patients with a suspected ruptured aneurysm (12). The rate of fenestration in this study was higher than the above report. Among the 28 cases, the fenestrations were observed in the inferior BA trunk in five patients (17.9%) who were afflicted with a total of 6 aneurysms. The cases are shown in **Figures 4**, **5**.

The majority of BA trunk aneurysms are dissection aneurysms, but those occurring within fenestrations are located in regions of bifurcation and are therefore saccular bifurcation aneurysms (13). Among the 29 combined aneurysms in the 28 patients in this study, the six aneurysms located within fenestrations of the inferior BA were not dissection aneurysms, while the remaining 23 aneurysms were.

Since craniotomy clipping is very difficult, EVT remains the first procedure of choice for BA trunk aneurysms (14). In the current choices for EVT, the flow-diverting stent has been widely used in cerebral aneurysms (2, 15). However, the flow-diverting stent is rarely used for BA trunk aneurysms. This is because the BA is short, with limited space for operations. Furthermore, the perforators of the BA are tiny, and each is extremely important. Therefore, brain stem infarctions caused by perforator ischemia after the application of flow-diverting stents will lead to a poor patient prognosis.

Therefore, conventional low-metal-coverage EVT techniques are still preferred for BA trunk aneurysms (16). These low-metal-coverage stents include the Neuroform stent (Stryker Neurovascular, Fremont, CA, USA), Enterprise stent (Codman

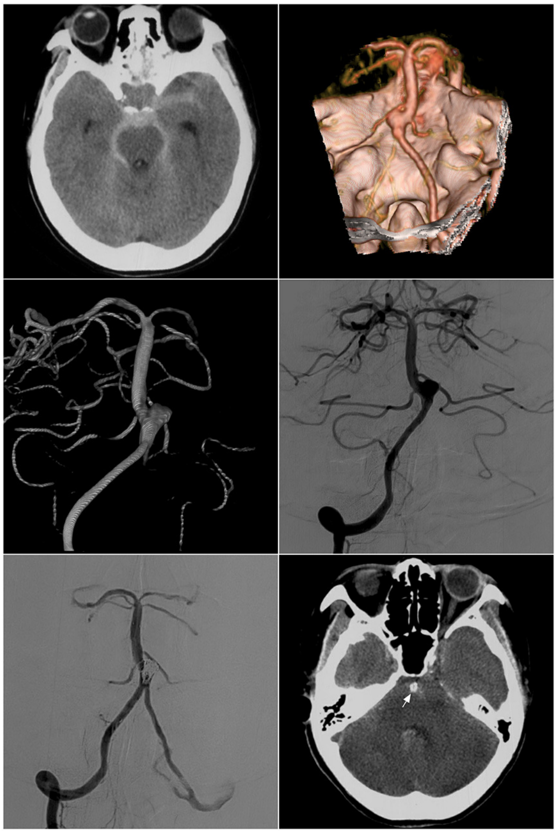

FIGURE 6 | EVT with an ischemic complication. **(A)** CT shows hemorrhage in the anterior brainstem and subarachnoid space of the cisterna; **(B)** CTA shows a saccular wide-necked lateral-wall aneurysm in the inferior BA. **(C,D)** Preoperative VA-DSA (C for 3D reconstruction, D for 2D embolization) shows a saccular aneurysm in the inferior BA, lateral-wall type. **(E)** Postoperative VA-DSA shows the aneurysm following stent-assisted EVT, MRRC Grade I. **(F)** For the patient who immediately developed coma after EVT, the postoperative CT shows no hemorrhage and the coil (arrow) in the BA lumen. BA, basilar artery; CT, computed tomography; CTA, CT angiography; DSA, digital subtraction angiography; EVT, endovascular therapy; MRRC, Modified Raymond-Roy Classification; VA, vertebral artery.

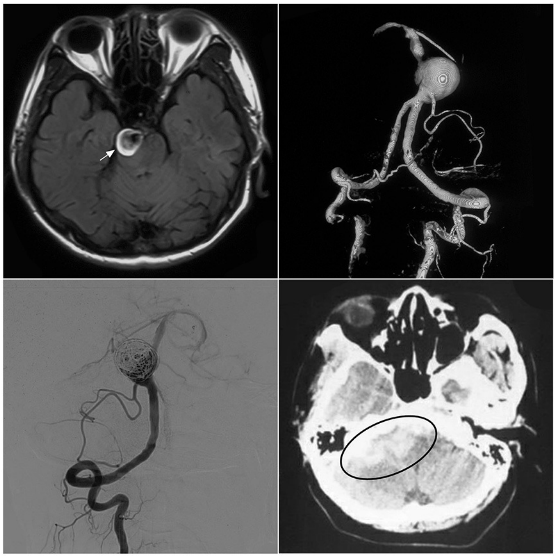

FIGURE 7 | EVT with a delayed hemorrhage. **(A)** MR T2WI shows an aneurysm in the right pons (arrow); **(B)** 3D-reconstructed DSA shows a spherical aneurysm in the inferior BA. **(C)** Postoperative VA-DSA shows the aneurysm following simple coiling embolization, MRRC Grade IIIa. **(D)** 1-month postoperative CT shows hemorrhage in the lateral brain stem (ellipse). BA, basilar artery; CT, computerized tomography; DSA, digital subtraction angiography; EVT, endovascular therapy; MR, magnetic resonance; MRRC, Modified Raymond-Roy Classification; VA, vertebral artery.

Neurovascular, Raynham, MA, USA), Solitaire (Medtronic, Irvine, California, USA), low-profile visualized intraluminal support (LVIS) stent (MicroVention Inc., Aliso Viejo, CA, USA), and LEO stent (Balt Extrusion, Montmorency, France) (17).

Among saccular aneurysms, single coiling can be used for narrow-necked aneurysms, while a low-metal-coverage stent is needed for wide-necked aneurysms to reduce the impact of the stent on BA perforations (18). When aneurysms involve spherical or fusiform dissections of the BA, they can only be coiled at first, followed by BA channel reconstruction with a stent (19). Nevertheless, we realize that even very successful EVT operations may develop serious complications or consequences postoperatively. This is because the greatest obstacle for EVT in treating BA trunk aneurysms lies in the abundant branches of the BA.

Among the 28 patients in this study, nine patients experienced ischemic complications, with an incidence of 32.1%. This high incidence of complications is notable for aneurysms treated by EVT. All nine incidents of ischemic complications were caused by perforator occlusion or BA ischemia. Since hemorrhage was not found on postoperative CT reexamination, perforator ischemia may not necessarily manifest on MR despite the severity of the symptoms, such as the patient in **Figure 3**. Ischemic complications during the EVT of BA trunk aneurysms may be caused by traction or coverage of BA perforations caused by coils or stents. A typical BA ischemia is shown in **Figure 6**.

Our study found that patients with mild complications, such as hemiplegia, gradually recovered with satisfactory prognoses, while the patients who developed coma had poor prognoses and eventually died. In addition, for palliatively embolized aneurysms, coiling may result in a worse prognosis. For example, in our study, a patient experienced aneurysm rupture 1 month after MRRC IIIa embolization, which is also noteworthy (**Figure 7**).

LIMITATIONS

This was a retrospective single-center study between 2015 and 2020. While the data were distributed across 5 years, the follow-up time was short in the cases close to 2020. As a result of the economic status in rural areas in China, angiographic follow-up data could be obtained only for a small proportion of the patients in this series, which affected the interpretation of the angiographic outcomes.

CONCLUSION

This study indicates that conventional EVT is still a high-risk procedure for BA trunk aneurysms, with a 35.7% incidence of complications. Therefore, it is necessary to actively and carefully evaluate the perforators of the BA trunk to ensure that EVT does not damage them. This study also shows that 82.1% of patients recovered well. Therefore, EVT can nevertheless result in an acceptable prognosis.

DATA AVAILABILITY STATEMENT

The raw data supporting the conclusions of this article will be made available by the authors, without undue reservation.

ETHICS STATEMENT

Written informed consent was obtained from the individual(s) for the publication of any potentially identifiable images or data included in this article.

AUTHOR CONTRIBUTIONS

JY and KX: contributed to the conception and design of the manuscript and critically revised the manuscript. YW and JS: collected the medical records of the patients and wrote the manuscript. All authors approved the final version this manuscript.

REFERENCES

1. Granja MF, Monteiro A, Agnoletto GJ, Jamal S, Sauvageau E, Aghaebrahim A, et al. A systematic review of non-trunk basilar perforator aneurysms: is it worth chasing the small fish? *J Neurointerv Surg.* (2020) 12:412–6. doi: 10.1136/neurintsurg-2019-015311
2. Cho KC, Jeon P, Kim BM, Lim SM, Jung WS, Kim JJ, et al. Saccular or dissecting aneurysms involving the basilar trunk: endovascular treatment and clinical outcome. *Neurol Res.* (2019) 41:671–7. doi: 10.1080/01616412.2019.1611185
3. Sai Kiran NA, Kiran Kumar VA, Kumar VA, Agrawal A. Microsurgical clipping of distal basilar trunk aneurysm during adenosine-induced profound hypotension. *Asian J Neu.* (2019) 14:1214–7. doi: 10.4103/ajns.AJNS_157_19
4. van Oel LI, van Rooij WJ, Sluzewski M, Beute GN, Lohle PN, Peluso JP. Reconstructive endovascular treatment of fusiform and dissecting basilar trunk aneurysms with flow diverters, stents, and coils. *AJNR Am J Neuroradiol.* (2013) 34:589–95. doi: 10.3174/ajnr.A3255
5. Ikram A, Zafar A. *Basilar Artery Infarct*. Treasure Island (FL): StatPearls (2020).
6. Mascitelli JR, Moyle H, Oermann EK, Polykarpou MF, Patel AA, Doshi AH, et al. An update to the Raymond-Roy Occlusion Classification of intracranial aneurysms treated with coil embolization. *J Neurointerv Surg.* (2015) 7:496–502. doi: 10.1136/neurintsurg-2014-011258
7. Burn JP. Reliability of the modified Rankin Scale. *Stroke.* (1992) 23:438. doi: 10.1161/str.23.3.438b
8. Gross BA, Puri AS, Du R. Basilar trunk perforator artery aneurysms. Case report and literature review. *Neurosurg Rev.* (2013) 36:163–8. doi: 10.1007/s10143-012-0422-1
9. Saliou G, Sacho RH, Power S, Kostynskyy A, Willinsky RA, Tymianski M, et al. Natural history and management of basilar trunk artery aneurysms. *Stroke.* (2015) 46:948–53. doi: 10.1161/STROKEAHA.114.006909
10. Adeeb N, Ogilvy CS, Griessenauer CJ, Thomas AJ. Expanding the indications for flow diversion: treatment of posterior circulation aneurysms. *Neurosurgery.* (2020) 86:S76–84. doi: 10.1093/neuros/nyz344
11. Saeki N, Rhoton AL Jr. Microsurgical anatomy of the upper basilar artery and the posterior circle of Willis. *J Neurosurg.* (1977) 46:563–78. doi: 10.3171/jns.1977.46.5.0563
12. van Rooij SB, Bechan RS, Peluso JP, Sluzewski M, van Rooij WJ. Fenestrations of intracranial arteries. *AJNR Am J Neuroradiol.* (2015) 36:1167–70. doi: 10.3174/ajnr.A4236
13. Morrison J, Alexander MD, Dowd CF, Cooke DL. Flanking the fenestration: circumferential limb-to-limb stent-assisted coiling of a basilar artery fenestration aneurysm. *J Neurointerv Surg.* (2018) 10:e12. doi: 10.1136/neurintsurg-2017-013252.rep
14. Liu Y, Shi X, Kc KIS, Sun Y, Liu F, Qian H, et al. Microsurgical treatment for complex basilar artery aneurysms with long-term follow-up in a series of 35 cases. *World Neurosurg.* (2018) 111:e710–21. doi: 10.1016/j.wneu.2017.12.158
15. Liang F, Zhang Y, Di Y, Guo F, Jiang C. Pipeline for previously stented basilar trunk aneurysm: a case focusing on how the pipeline should be deployed. *Chin Neurosurg J.* (2018) 4:27. doi: 10.1186/s41016-018-0134-7
16. Koizumi S, Shojima M, Iijima A, Oya S, Matsui T, Yoshikawa G, et al. Stent-assisted coiling for ruptured basilar artery dissecting aneurysms: an initial experience of four cases. *Neurol Med Chir (Tokyo).* (2016) 56:43–8. doi: 10.2176/nmc.cr.2015-0233
17. Oushy S, Rinaldo L, Brinjikji W, Cloft H, Lanzino G. Recent advances in stent-assisted coiling of cerebral aneurysms. *Expert Rev Med Devices.* (2020) 17:519–32. doi: 10.1080/17434440.2020.1778463
18. Mine B, Bonnet T, Vazquez-Suarez JC, Iosif C, Lubicz B. Comparison of stents used for endovascular treatment of intracranial aneurysms. *Expert Rev Med Devices.* (2018) 15:793–805. doi: 10.1080/17434440.2018.1538779
19. Sirakov A, Bhogal P, Mohlenbruch M, Sirakov S. Endovascular treatment of patients with intracranial aneurysms: feasibility and successful employment of a new low profile visible intraluminal support (LVIS) EVO stent. *Neuroradiol J.* (2020) 33:377–85. doi: 10.1177/1971400920941402

Conflict of Interest: The authors declare that the research was conducted in the absence of any commercial or financial relationships that could be construed as a potential conflict of interest.

Copyright © 2021 Wang, Xu, Song and Yu. This is an open-access article distributed under the terms of the Creative Commons Attribution License (CC BY). The use, distribution or reproduction in other forums is permitted, provided the original author(s) and the copyright owner(s) are credited and that the original publication in this journal is cited, in accordance with accepted academic practice. No use, distribution or reproduction is permitted which does not comply with these terms.
