# Supplementary material for: Management strategies and outcomes of basilar trunk aneurysms: a systematic review and meta-analysis
Source: Langenbecks Arch Surg. 2026 Jan 20;411(1):71. doi: 10.1007/s00423-025-03959-3 (PMC12852281; doi:10.1007/s00423-025-03959-3)
Supplement: Supplementary file 1 — Supplementary file1 (ZIP 55327 KB) [file 423_2025_3959_MOESM1_ESM.zip › literature.zip!/meta/Wu 2023.pdf]

Check for updates

Original Research Article

INR INTERVENTIONAL NEURORADIOLOGY

Interventional Neuroradiology
1–8
© The Author(s) 2023
Article reuse guidelines:
sagepub.com/journals-permissions
DOI: 10.1177/15910199231193904
journals.sagepub.com/home/ine
Sage

Complications after endovascular treatment of large basilar trunk aneurysms

Qiaowei Wu[1,*], Changsi Ai[2,*], Yuange Bi[3,*], Jinbiao Yao[1], Qi Sun[1], Shancai Xu[1], Bohan Zhang[1], Pei Wu[1], Yongjian Kui[1], Huaizhang Shi[1] and Yuehua Wang[4]

Abstract

Background and Purpose: Large basilar trunk aneurysms (BTAs) have a poor natural history if left untreated and always pose a significant challenge to endovascular treatment. The present study aimed to analyze the complications after endovascular treatment of large BTAs.

Methods: This was a retrospective, observational, cohort study. Between January 2015 and September 2022, 9116 patients with intracranial aneurysms came to our institution for management, of which 34 patients with 34 large (≥10 mm) BTAs were treated with endovascular treatment. Postprocedural complications, clinical, and angiographic outcomes were evaluated.

Results: All 34 patients (34 aneurysms) were successfully treated, of which 13 aneurysms were treated with flow diversion (FD), and 21 aneurysms were treated with stent-assisted coiling. Neurological complications occurred in 12 (35.3%) patients, with 7 (20.6%) deaths. Ischemic complications occurred in 10 (29.4%) patients, and 4 (11.8%) patients experienced hemorrhagic events. The incidences of favorable outcomes at discharge and last follow-up were 85.3% and 75.8%, respectively. The cumulative survival rates at 1 and 3 years were 86.5% and 71.4%, respectively. Unilateral vertebral artery sacrifice was associated with postprocedural complications (hazard ratio: 3.74, 95% confidence interval: 1.06–13.25, $p = 0.041$). The postprocedural complication rates were comparable between patients treated with FD and stent-assisted coiling (5/13, 38.5% vs. 7/21, 33.3%, $p > 0.99$). Angiography follow-up was available for 21 patients, and complete aneurysm occlusion was observed in 16/21 (76.2%) aneurysms.

Conclusions: Endovascular treatment might be a feasible option for treating large BTAs. However, clinicians should be alerted to procedure-related complications, especially ischemic complications that cause disability or death. Unilateral vertebral artery sacrifice might be associated with postprocedural complications.

Keywords

Flow diversion, stent-assisted coiling, endovascular treatment, basilar artery, intracranial aneurysm

Received 9 May 2023; revised July 2, 2023; accepted 24 July 2023

Introduction

Basilar trunk aneurysms (BTAs) are rare and have a poor natural history. Reportedly, the prevalence of BTAs is around 1–2% of all intracranial aneurysms,[1,2] and aneurysms located in the posterior circulation are associated with a greater tendency to rupture than those in the anterior circulation.[3,4] If left untreated, BTAs have a trend to progressively increase in size, leading to fatal brainstem dysfunction and subarachnoid hemorrhage (SAH).[5,6] Surgical intervention is essential management in those cases, especially large (≥10 mm) BTAs. Due to the anatomy location, pathological features, and relation to the branch vessels, the open surgical options of BTAs are associated with high morbidity and mortality rates.[5] Stent-assisted coiling (SAC) is now the preferred treatment for posterior circulation aneurysms; however, it

[1]Department of Neurosurgery, The First Affiliated Hospital of Harbin Medical University, Harbin, Heilongjiang, China
[2]Department of Neurology, Hongda Hospital of Jiamusi University, Jiamusi, Heilongjiang, China
[3]Department of Neurosurgery, The First Hospital of Jilin University, Changchun, Jilin, China
[4]Department of Neurosurgery, Shanghai Seventh People's Hospital, Shanghai, China

[*]Qiaowei Wu, Changsi Ai, and Yuange Bi contributed equally to this work

Corresponding authors:
Yuehua Wang, Department of Neurosurgery, Shanghai Seventh People's Hospital, Shanghai 200000, China.
Email: yhwang1973@163.com
Huaizhang Shi, Department of Neurosurgery, The First Affiliated Hospital of Harbin Medical University, Harbin, Heilongjiang 150000, China.
Email: huaizhangshi@126.com

can be challenging to apply to BTAs.[7] Flow diversion (FD), as one of the endovascular treatment modalities, has been increasingly used in the treatment of posterior circulation aneurysms in recent years, but the safety and efficiency of FD treatment for large BTAs remain to be further explored.[7–8] Therefore, the aim of this study was to analyze the complications after endovascular treatment of BTAs.

Methods

Patient population

This is a retrospective, observational, cohort study assessing the safety and outcomes of endovascular treatment in patients with large BTAs. The institutional review board of the First Affiliated Hospital of Harbin Medical university approved our study, and written informed consent was obtained for all patients before the treatment. From January 2015 to September 2022, we searched our database retrospectively, including 9116 patients with intracranial aneurysms, 2934 of whom received endovascular treatment. Basilar trunk aneurysms are defined as aneurysms originating from the origin of the basilar artery to the origin of the superior cerebellar artery.[2] The inclusion criteria were as follows: (1) patient harboring BTAs, confirmed by digital subtraction angiography (DSA) or computed tomography (CT) angiography; (2) aneurysm maximum diameter ≥ 10 mm; and (3) target aneurysm was treated with FD or SAC. Exclusion criteria were as follows: (1) basilar tip aneurysm, superior cerebellar artery aneurysm, posterior cerebral artery aneurysm or aneurysm located on branch vessels arising from the basilar artery; (2) patient complicated by other treated intracranial aneurysms; and (3) patients with unfavorable condition before the procedure (modified Rankin Scale [mRS] > 2). We present the following article in accordance with the STROBE reporting checklist.

Procedure

The treatment modalities were decided by the neurointerventionists (with >10 years of experience in neurointerventional surgery), depending on patient preferences and aneurysm anatomic factors. After the guiding catheter was placed in the distal vertebral artery, two-dimensional angiography and three-dimensional rotational angiography were performed. Three-dimensional reconstruction images were used to determine the work projection, measure the parent artery and aneurysm diameter, and determine the stent size. For patients who were treated with FD, Tubridge FD (MicroPort NeuroTech, Shanghai, China) and pipeline embolization device (PED; Covidien, California, USA) were used in our study. A Marksman microcatheter (Covidien, California, USA) or a Fastrack microcatheter (MicroPort NeuroTech, Shanghai, China) was used to deploy the FD. For an aneurysm involving a long segment, multiple FDs deployment technique was used. After the deployment, multi-angle nonsubtraction angiography and Vaso CT were performed to evaluate the FD apposition. Once the improper FD expansion was detected, the balloon angioplasty and/or in-stent "massage" with microcatheter/microwire was performed. For patients who were treated with SAC, aneurysms were coiled with stent-assisted by Low-prole Visualized Intraluminal Support (MicroVention-Terumo, California, USA), Enterprise (Codman Neurovascular, Massachusetts, USA), or Solitaire (ev3, Irvine, California, USA). Coils were packed until satisfactory aneurysm occlusion was achieved and/or additional packing was not possible. Head CT scanning was routinely performed immediately after the procedure to exclude the subarachnoid or intraparenchymal hemorrhage.

Antiplatelet protocol

Daily dual antiplatelet therapy (DAT, 75 mg clopidogrel, and 100 mg aspirin) was administrated for all the patients at least 3–5 days before the procedure. At least 3 days after the DAT, thromboelastography was used to monitor the platelet function. If clopidogrel hyporesponders or nonresponders were detected, clopidogrel would be changed to ticagrelor 90 mg, twice daily. In addition, tirofiban was routinely administrated for all patients in this study. During or immediately after stent deployment, tirofiban was administered as an intravenous bolus (usually 5 μg/kg) over a 3-min period and then maintained (usually 0.05 μg/kg/min) for at least 24–48 h. The DAT would maintain for at least six months (three months for patients treated with SAC) after the procedure, and single antiplatelet therapy (usually 100 mg aspirin) was maintained indefinitely.

Data collection

The clinical outcome data were collected via neurologic examination or telephone call at 30 days, six months, and the annual follow-up. Clinical outcome was evaluated using the mRS score at discharge and at the last clinical follow-up (a mRS score of 0–2 was defined as a favorable outcome). Angiographic follow-up with DSA was performed to determine the aneurysm occlusion status of the patients, which was scheduled at six months and 1–2 years after the procedure. Angiography outcomes were evaluated using the Raymond-Roy grading scale (grade 1 of Raymond-Roy was defined as complete aneurysm occlusion).[9]

Postprocedural neurological complications included ischemic complications and hemorrhagic complications. Ischemic complications were defined as the following: (1) in-stent thrombosis, partial or complete occlusion of the proximal or distal arteries on DSA intraprocedurally or postprocedurally; (2) thromboembolism symptoms (excluding vasospasm) with or without corresponding cerebral infarction on postprocedural magnetic resonance imaging (MRI)/CT. Hemorrhagic complications were defined as (1) intraprocedural contrast extravasation on DSA; (2) postprocedural CT/MRI showed new

intraparenchymal hemorrhage with or without clinical symptoms; and (3) new SAH on CT.

Statistical analysis

Normally distributed continuous variables were summarized as mean ± standard deviation, while non-normally distributed continuous variables were summarized as the median and interquartile range (IQR). Categorical variables were summarized as numbers followed by percentages. Analysis of variables between the two treatment modalities was carried out by using Mann–Whitney *U* test or independent sample t-test for continuous variables, and Fisher exact test for qualitative variables. The Kaplan–Meier survival analysis was used to calculate the cumulative survival rate at follow-up. The univariate and multivariate Cox regression analyses were performed to identify the possible risk factors for treatment-related complications. Cases with missing follow-up data were omitted. A p value < 0.05 (two-sided) was considered to indicate statistical significance. Statistical analysis was performed using SPSS 22.0 software (IBM Corp., Armonk, NY, USA).

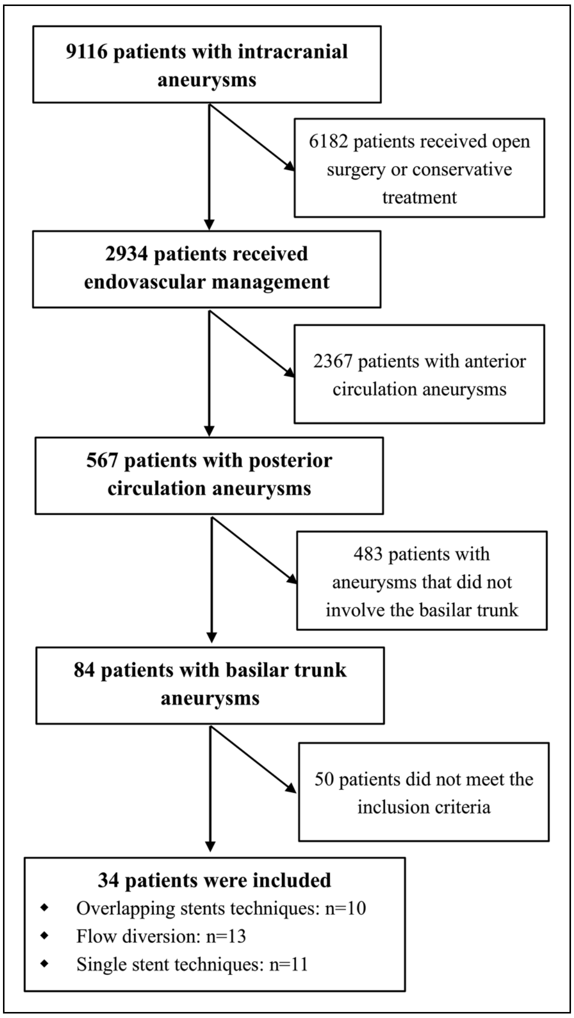

Figure 1. Flowchart of the patient selection process.

Results

Patient and aneurysm characteristics

Among the 9116 patients in our database, 84 patients with BTAs were treated with endovascular methods, and 34 patients with 34 large BTAs who had undergone endovascular treatment were identified and enrolled in this study (Figure 1). Nineteen (55.9%) patients presented with ischemic symptoms before treatment, 12 (35.3%) patients found aneurysm incidentally without specific clinical symptoms, and 3 (8.8%) patients presented with SAH. The median aneurysm size was 14.5 (IQR, 10.9–18.5) mm. Five (14.7%) aneurysms were saccular, and 29 (85.3%) aneurysms were fusiform/dissecting. Eight (23.5%) aneurysms involved the vertebrobasilar junction. The detailed baseline variables are summarized in Table 1.

Procedure-related data

A total of 19 FDs were used to treat 13 aneurysms without adjunctive coil embolization, including 14 (73.7%) PEDs and 5 (26.3%) Tubridge FDs. Of the 13 aneurysms, 4 (30.8%) aneurysms were treated with the overlapping FDs technique, of which 2 (15.4%) aneurysms were treated with three overlapping FDs and 2 (15.4%) aneurysms were treated with two overlapping FDs. Of the 21 aneurysms treated with SAC, 11 (52.4%) aneurysms were treated with single SAC, and 10 (47.6%) aneurysms were treated with overlapping SAC. The median procedure time was 119.3 ± 39.4 min. Five (14.7%) aneurysms were treated with adjunctive unilateral vertebral artery sacrifice (Table 1).

Clinical outcomes

The median clinical follow-up time was 21.0 (IQR, 13.0–40.5) months. The incidences of favorable outcomes at discharge and last follow-up were 85.3% (29/34) and 75.8% (25/33), respectively. The postprocedural neurological complications occurred in 12 (35.3%) patients, with 7 (20.6%) deaths. Ten (29.4%) patients first experienced complications during the periprocedural period and 2 (5.9%) patients first experienced complications during follow-up. Ischemic complications occurred in 10 (29.4%) patients. Four (11.8%) patients experienced hemorrhagic events, of which 2 (5.9%) patients were complicated with ischemic complications (Figures 2 and 3, Table 2).

The cumulative survival rates at 1 and 3 years were 86.5% and 71.4%, respectively. The univariate Cox regression analysis showed that unilateral vertebral artery sacrifice was related to overall postprocedural complications (hazard ratio [HR]: 4.07, 95% confidence interval [CI]: 1.18–14.00, $p = 0.026$). After adjusting for treatment modalities and onset symptoms, unilateral vertebral artery sacrifice was significantly associated with overall postprocedural complications (HR: 3.74, 95% CI: 1.06–13.25, $p = 0.041$).

Table 1. Patients' characteristics and outcomes.

Variables	n = 34 patients (34 aneurysms)
Male, *n* (%)	21(61.8)
Age, years (mean ± SD)	60.2 ± 9.5
Hypertension, *n* (%)	21 (61.8)
Diabetes mellitus, *n* (%)	8 (23.5)
Smoking, *n* (%)	11 (32.4)
Alcohol abuse, *n* (%)	9 (26.5)
Onset symptoms, *n* (%)	
Ischemic stroke	19 (55.9)
Incidental finding	12 (35.3)
Hemorrhagic event	3 (8.8)
mRS before procedure, *n* (%) [a]	
0	11 (32.4)
1	16 (47.1)
2	7 (20.6)
Maximum diameter of aneurysm, median (IQR)	14.5 (10.9–18.5)
Aneurysm type, *n* (%)	
Saccular	5 (14.7)
Fusiform/dissecting	29 (85.3)
Aneurysm involved vertebrobasilar junction, *n* (%)	8 (23.5)
Procedure time, min (mean ± SD)	119.3 ± 39.4
Adjunctive unilateral vertebral artery sacrifice, *n* (%)	5 (14.7)
Type of stents, *n* (%)[a]	
PED	11 (32.4)
Tubridge	2 (5.9)
Enterprise	13 (38.2)
LVIS	1 (2.9)
Enterprise + LVIS	6 (17.6)
Solitaire	1 (2.9)
Postprocedural complications, *n* (%)	
Ischemic events	10 (29.4)
Hemorrhagic events[b]	4 (11.8)
mRS at discharge, *n* (%)	
0–2	29 (85.3)
3–6	5 (14.7)
mRS at last follow-up, *n* (%)[c]	
0–2	25 (75.8)
3–6	8 (24.2)
Follow-up angiographic outcome, *n* (%)[d]	
Complete occlusion	16 (76.2)
Incomplete occlusion	5 (23.8)

SD: standard deviation; IQR: interquartile range; PED: pipeline embolization device; mRS: modified Rankin scale; LVIS: low-prole visualized intraluminal support.
[a]Percentage totaling not 100% due to rounding.
[b]Two patients were complicated with ischemic complications.
[c]Data were missing for one patient due to the death during the periprocedural period.
[d]Data were missing for seven patients due to death, and six patients due to unwillingness to angiographic follow-up.

Angiography outcomes

Angiography follow-up was available for 21 patients with a median follow-up time of 9.0 (IQR, 6.0–15.0) months. Complete aneurysm occlusion (grade 1 of Raymond-Roy) was observed in 16/21 (76.2%) patients, of which 8/9 aneurysms were treated with FD and 8/12 aneurysms were treated with SAC.

Subgroup analysis

The postprocedural complication rates were 38.5% (5/13) and 33.3% (7/21) in patients treated with FD and SAC, respectively, and no statistically significant difference was detected between the two treatment modalities ($p > 0.99$). The mortality rates were also comparable between patients treated with FD and SAC (3/13, 23.1% vs. 4/21, 19.0%, $p > 0.99$).

Discussion

In the present study, we have reviewed our experience of endovascular treatment for patients with large (≥10 mm) BTAs and reported the clinical and angiography outcomes. The favorable outcome rate at discharge and last follow-up (median: 21.0 months) were 85.3% and 75.8%, respectively. Sixteen of 21 (76.2%) patients achieved complete aneurysm occlusion.

The natural prognosis of large BTAs is poor. A retrospective multicenter cohort study by Nakatomi et al.[5] identified 11 patients with giant fusiform and dolichoectatic aneurysms located on the basilar trunk artery or involved vertebrobasilar junction who did not receive surgical intervention and found 10 (90.9%) patients died during follow-up. Microsurgical management is one of the treatment modalities for BTAs, however, due to the limited surgical accessibility and the relation to perforating branches, microsurgical of large BTAs may be associated with relatively high morbidity and mortality rates.[5] Conventional endovascular treatment is now the first-line treatment for BATs, but there still be a relatively high unfavorable outcome rate. Sim et al.[7] reported the clinical outcome of 40 patients with BTAs who had undergone various endovascular modalities. They found the favorable outcome rate was 67.5%, and large/giant BTAs were associated with unfavorable angiographic outcomes. In addition, SAC can be challenging to apply to large BTAs due to the technical difficulty, mass effect after treatment, and relatively high recurrence rate.[8,10] FD has been increasingly used in endovascular treatment of intracranial aneurysms, and it shows reasonable safety and efficiency profiles in treating posterior circulation aneurysms.[11,12] A meta-analysis by Kiyofuji et al.[13] showed the overall morbidity and mortality rates were 26% and 21%, respectively. Another systematic review showed the major complication rate was 22% after FD treatment of overall posterior circulation aneurysms.[11] However, data on the safety and efficiency of endovascular treatment for large BTAs are limited.

We found the overall postprocedural neurological complication rate after endovascular treatment was 35.3%, including seven deaths (20.6%). Ischemic complications accounted for the majority of all complications in our study. Two patients experienced in-stent thrombosis, of which one occurred intraprocedurally, and the other

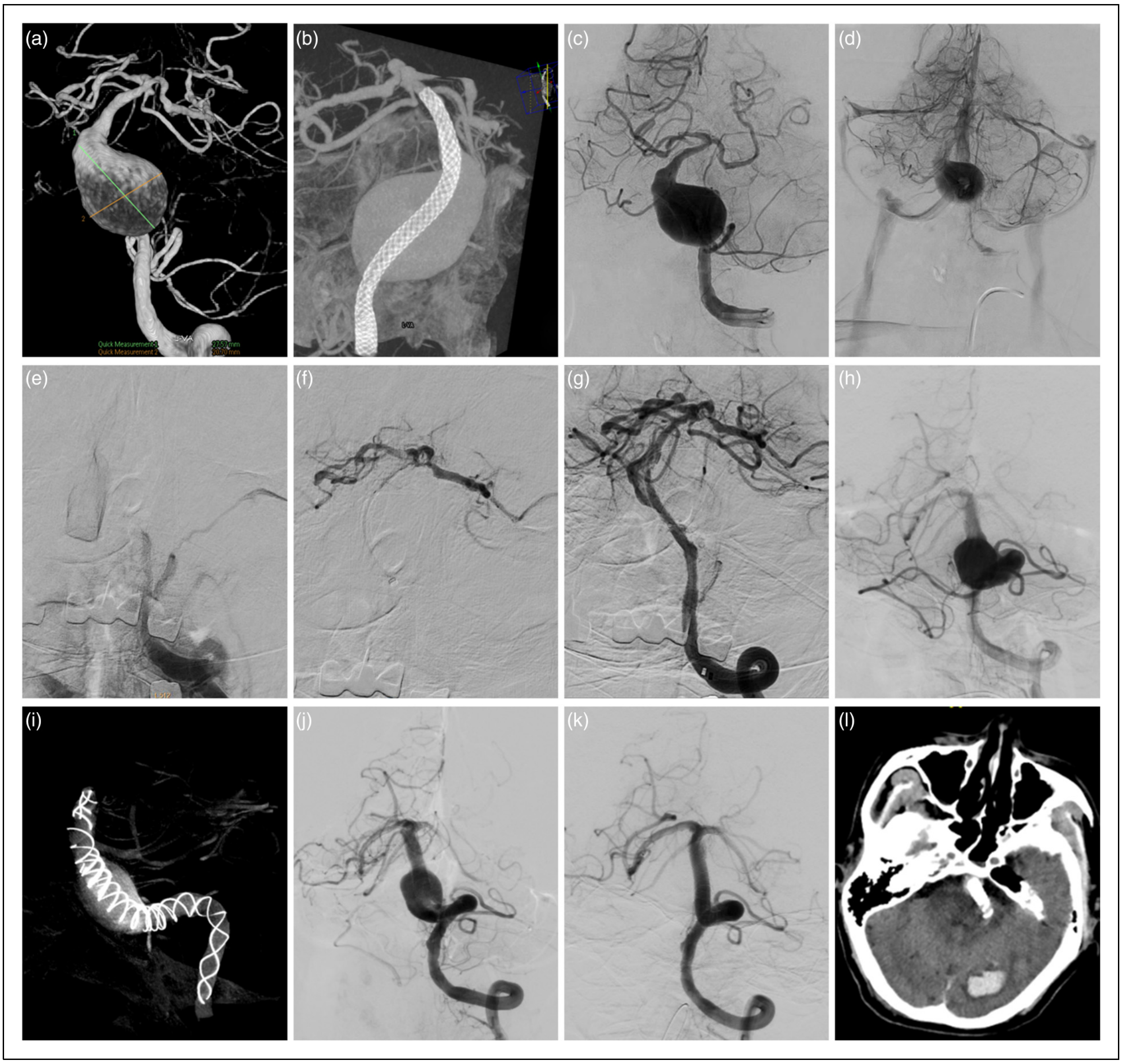

Figure 2. Example of patients treated with flow diversion: (a) Image from a patient with a large basilar trunk aneurysm (BTA). (b) A pipeline embolization device was deployed, and Vaso computed tomography (CT) showed that the stent was deployed properly. Postprocedural digital subtraction angiography (DSA) showed the parent artery patency (c), and blood flow detained was detected (d). (e) Five months after discharge, the patient experienced progressive consciousness disorder and followed DSA showed in-stent thrombosis. (f) Aspiration thrombectomy was performed. (g) Blood flow was restored and the aneurysm neck was residual. The patient died later. (h, i) Overlapping Tubridge flow diverters were used to treat another patient with a large BTA. (j) Postprocedural DSA showed the parent artery patency. The patient experienced bilateral limb weakness and aphasia after the procedure. (k) Three days after the procedure, DSA showed the aneurysm was completely occluded. (l) One week after the procedure, intraparenchymal hemorrhage was detected on CT.

occurred five months later. Improper stent expansion and insufficient preprocedural antiplatelet drug administration may be possible reasons for intraprocedural in-stent thrombosis. Stent migration, irregular antiplatelet drug administration, delayed cobalt allergic reaction, atherosclerosis, and cardioembolism may also attribute to delayed in-stent thrombosis. Eight patients experienced periprocedural ischemic events and five patients re-experienced ischemic events during follow-up. Insufficient antiplatelet drugs administration, distal artery, or branch vessel occlusion due to detachment of vessel mural thrombus or intra-aneurysmal thrombus during mechanical manipulation, cobalt allergic reaction, and branch vessel occlusion due to improper stent expansion may be possible causes for ischemic events.[14] In addition, six patients who were treated with overlapping stents experienced periprocedural ischemic complications. The mechanical manipulation may increase the procedure time and increase the possibility of thrombus detachment. Moreover, the relatively high metal coverage of overlapping stents on the one hand may enhance the FD effect, but on the other hand, increase the possibility of occlusion of the covered perforator branches.

Delayed intraparenchymal hemorrhage is one of the serious complications after endovascular treatment of

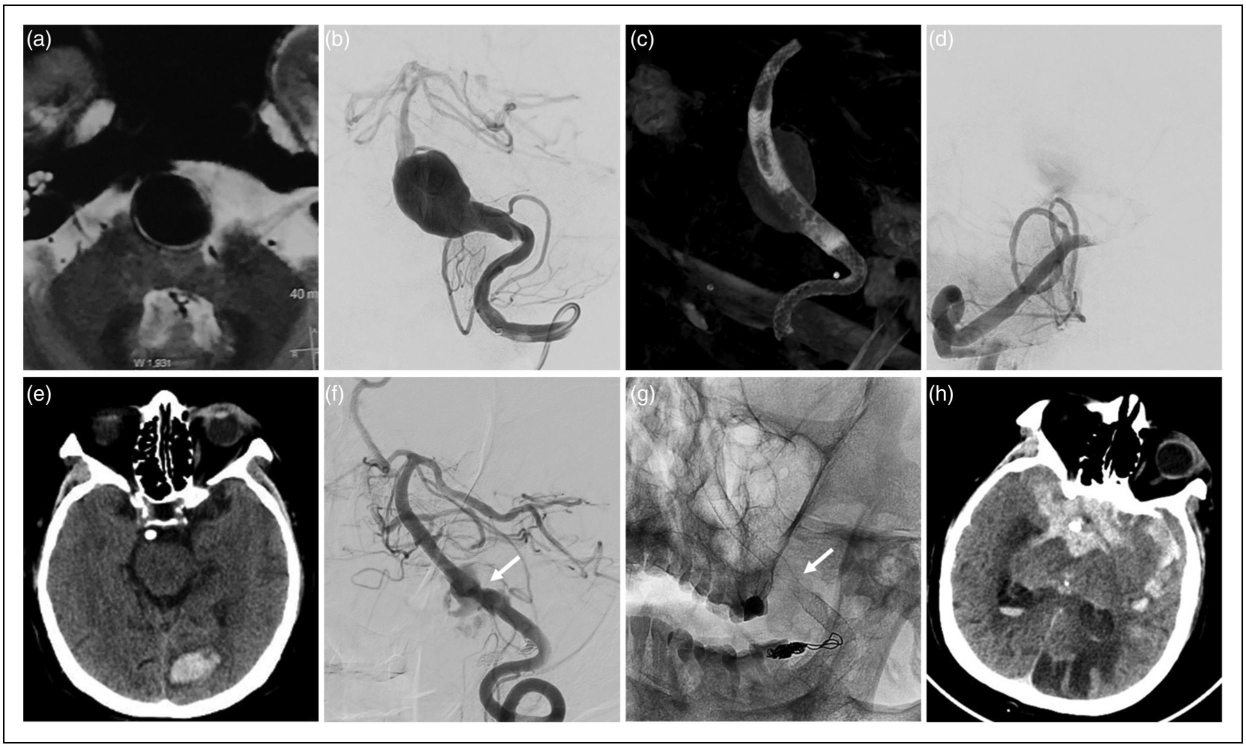

Figure 3. Example of a patient treated unilateral vertebral artery sacrifice: (a, b) Pretreatment image of a patient with a large basilar trunk aneurysm (BTA). (c) Three overlapping pipeline embolization devices were successfully deployed. (d) The unilateral vertebral artery was sacrificed. The patient experienced right-sided motor weakness after the procedure. No hemorrhage was detected on the head computed tomography (CT). After the administration of low-molecular-weight heparin and tirofiban, the motor weakness was temporarily resolved. Head CT showed intraparenchymal hemorrhage later (e). (f, g) During the follow-up, the patient experienced left-sided motor weakness and followed digital subtraction angiography (DSA) showing partial aneurysm residual and mild in-stent stenosis (white arrow). (h) Two days after the admission, the patient developed consciousness disorder and followed head CT showed the aneurysm rupture.

intracranial aneurysms.[15,16] Rouchaud et al.[16] conducted a literature overview of delayed hemorrhagic events after FD treatment for intracranial aneurysms. The results showed 42.5% of patients died after intraparenchymal hemorrhage, 26.0% of patients had poor outcomes, and only 31.5% of patients had good clinical outcomes. A multicenter study by Benaissa et al.[15] found that intraparenchymal hemorrhage usually occurred around seven days after treatment, and patient with large/giant aneurysm was more likely to have an intraparenchymal hemorrhage. In our study, three patients experienced intraparenchymal hemorrhage within seven days after the procedure, and the maximum diameters of the aneurysms were 24.1 mm, 17.4 mm, and 10 mm, respectively. The specific mechanisms are not yet clarified, but multiple causes could contribute to intraparenchymal hemorrhage. Two intraparenchymal hemorrhages in our study were secondary to ischemic complications, ischemia–reperfusion hemorrhage may be a possible cause. Moreover, the administration of tirofiban and low-molecular-weight heparin after the ischemic complication may also contribute to intraparenchymal hemorrhages. The published computational fluid dynamics model revealed that patients with large aneurysm neck diameters may have an increased risk of hyperperfusion syndrome and ensuing subsequent delayed intraparenchymal hemorrhages after FD treatment.[17] In addition, flow reversal in distal collaterals and a history of hypertension may also be possible mechanisms of delayed intraparenchymal hemorrhage after FD treatment.[18,19]

Delayed aneurysm rupture is another serious complication after endovascular treatment. Once this occurred, around 74.7% of patients died in published studies.[15] In our study, two patients experienced delayed aneurysm rupture and subsequently died. A computational fluid dynamic study by Cebral et al.[20] demonstrated that flow modifications after stent placement may cause intra-aneurysmal pressure increases which can potentially lead to rupture, especially for giant intracranial aneurysms that may have very weak walls. Other studies have shown that intra-aneurysmal thrombosis may play a role in the pathophysiological mechanism of aneurysm rupture, as the thrombus is a source of a variety of proteases, which has high proteolytic activity, and could participate in the degradation of aneurysm wall, leading to aneurysm rupture.[21,22] Since large aneurysms are more likely to have intra-aneurysmal thrombus, such aneurysms seem to be more prone to delayed aneurysm rupture.

Our study found that unilateral vertebral artery sacrifice might increase the complication rate in treating large BTAs. For aneurysms involving the vertebrobasilar

Table 2. Complication details.

No.	Treatment strategy	Complication (Peri) Ischemic stroke	Complication (Peri) Hemorrhage	Complication (FU) Ischemic stroke	Complication (FU) Hemorrhage	mRS before admission	mRS at discharge	mRS at last FU
1	PED	In-stent thrombosis	-	Cerebral infarctions	-	1	0	6
2	PED	-	-	In-stent thrombosis	-	1	0	6
3	PED × 3 + UVAS	Cerebral infarctions	Intraparenchymal hemorrhage	Cerebral infarctions	Subarachnoid hemorrhage	0	4	6
4	TUB × 3	Cerebral infarctions	Intraparenchymal hemorrhage	-	-	1	5	4
5	PED	Cerebral infarctions	-	-	-	1	2	1
6	LVIS + UVAS	-	Intraparenchymal hemorrhage	-	-	1	2	0
7	EP + LVIS + UVAS	Cerebral infarctions	-	Cerebral infarctions	-	1	1	6
8	EP + LVIS	Cerebral infarctions	-	Cerebral infarctions	-	2	1	6
9	EP + LVIS	Cerebral infarctions	-	-	-	1	4	4
10	EP + LVIS + UVAS	Cerebral infarctions	-	Cerebral infarctions	-	0	4	6
11	EP + LVIS	-	Subarachnoid hemorrhage	-	-	2	6	-
12	EP	-	-	Cerebral infarctions	-	1	1	1

PED: pipeline embolization device; TUB: Tubridge flow diverter; UVAS: unilateral vertebral artery sacrifice; EP: enterprise stent; LVIS: low-prole visualized intraluminal support; Peri: periprocedure; FU: follow-up; mRS: modified Rankin Scale.

junction, if there was flow from one vertebral artery directly into the aneurysmal sac, unilateral vertebral artery sacrifice using coils could be performed to reduce flow into the aneurysmal sac.[23] The occlusion site was located at the distal end of the posterior inferior cerebellar artery, so we did not perform the balloon occlusion test on the premise of ensuring the patency of the posterior inferior cerebellar artery. However, even if the balloon occlusion test shows no abnormalities, the literature suggests that there is still a risk of delayed ischemic complications, and unpredictable hypoperfusion may be a cause.[24,25] In addition, after vertebral artery occlusion, an organized thrombus in the occlusion site may dislodge at any time due to thrombogenesis. Progressive constriction of the occlusive artery may squeeze the thrombus into the collateral arteries, leading to delayed ischemia complications or ischemia–reperfusion hemorrhage.[25]

The retrospective design with a small sample size is the main limitation of our study which may be insufficient to draw conclusions in multivariate Cox regression analysis and potential bias inherent in retrospective research is unavoidable. Moreover, only 21 patients have received the angiography follow-up, the aneurysm occlusion rate in our study may not be representative. Thus, a multicenter comparative trial with a large sample size would be of great interest.

Conclusions

Endovascular treatment might be a feasible option for treating large BTAs. Favorable clinical outcome and complete aneurysm occlusion rates during follow-up were 75.8% and 76.2%, respectively. Unilateral vertebral artery sacrifice might be associated with postprocedural complications. Clinicians should be alerted to procedure-related complications, especially ischemic complications that cause disability or death.

Declaration of conflicting interests

The author(s) declared no potential conflicts of interest with respect to the research, authorship, and/or publication of this article.

Ethical approval statement

The institutional review board of the First Affiliated Hospital of Harbin Medical University approved our study, and written informed consent was obtained for all patients before the treatment.

Funding

The author(s) disclosed receipt of the following financial support for the research, authorship, and/or publication of this article: This work was supported by the Key Research and

Development Projects in Heilongjiang Province, the Foundation of The First Affiliated Hospital of Harbin Medical University, the Natural Science Foundation of Heilongjiang Province of China, (grant number 2022ZX06C03, 2019L02, YQ2019H05).

ORCID iDs

Qiaowei Wu https://orcid.org/0000-0001-9454-1411
Shancai Xu https://orcid.org/0000-0002-1111-2409
Bohan Zhang https://orcid.org/0000-0003-2442-7036
Yuehua Wang https://orcid.org/0009-0006-6311-3282

References

1. Pandey AS, Koebbe C, Rosenwasser RH, et al. Endovascular coil embolization of ruptured and unruptured posterior circulation aneurysms: review of a 10-year experience. *Neurosurgery* 2007; 60: 626–636.
2. Saliou G, Sacho RH, Power S, et al. Natural history and management of basilar trunk artery aneurysms. *Stroke* 2015; 46: 948–953.
3. Sonobe M, Yamazaki T, Yonekura M, et al. Small unruptured intracranial aneurysm verification study: SUAVe study, Japan. *Stroke* 2010; 41: 1969–1977.
4. Wiebers DO. Unruptured intracranial aneurysms: natural history, clinical outcome, and risks of surgical and endovascular treatment. *Lancet* 2003; 362: 103–110.
5. Nakatomi H, Kiyofuji S, Ono H, et al. Giant fusiform and dolichoectatic aneurysms of the basilar trunk and vertebrobasilar junction-clinicopathological and surgical outcome. *Neurosurgery* 2020; 88: 82–95.
6. Nasr DM, Flemming KD, Lanzino G, et al. Natural history of vertebrobasilar dolichoectatic and fusiform aneurysms: a systematic review and meta-analysis. *Cerebrovasc Dis* 2018; 45: 68–77.
7. Sim SY, Chung J, Choi JH, et al. Basilar artery trunk aneurysm: clinical and angiographic outcomes of endovascular treatment. *J Neurointerv Surg* 2022; 14: 262–267.
8. Li M, Liang H and Wang J. Unfavorable outcomes related to endovascular treatment of giant vertebrobasilar aneurysms. *Front Neurol* 2020; 11: 748.
9. Roy D, Milot G and Raymond J. Endovascular treatment of unruptured aneurysms. *Stroke* 2001; 32: 1998–2004.
10. Kim JH, Ko YS, Kwon SM, et al. Predictive factors of recurrence after endovascular treatment of unruptured vertebrobasilar fusiform aneurysms. *Clin Neuroradiol* 2023; 33: 73–86.
11. Alwakeal A, Shlobin NA, Golnari P, et al. Flow diversion of posterior circulation aneurysms: systematic review of disaggregated individual patient data. *AJNR Am J Neuroradiol* 2021; 42: 1827–1833.
12. Wu Q, Li C, Xu S, et al. Flow diversion vs. stent-assisted coiling in the treatment of intradural large vertebrobasilar artery aneurysms. *Front Neurol* 2022; 13: 917002.
13. Kiyofuji S, Graffeo CS, Perry A, et al. Meta-analysis of treatment outcomes of posterior circulation non-saccular aneurysms by flow diverters. *J Neurointerv Surg* 2018; 10: 493–499.
14. Fujii S, Fujita K, Yamaoka H, et al. Refractory in-stent stenosis after flow diverter stenting associated with delayed cobalt allergic reaction. *J Neurointerv Surg* 2022; 14: e4.
15. Benaissa A, Tomas C, Clarençon F, et al. Retrospective analysis of delayed intraparenchymal hemorrhage after flow-diverter treatment: presentation of a retrospective multicenter trial. *AJNR Am J Neuroradiol* 2016; 37: 475–480.
16. Rouchaud A, Brinjikji W, Lanzino G, et al. Delayed hemorrhagic complications after flow diversion for intracranial aneurysms: a literature overview. *Neuroradiology* 2016; 58: 171–177.
17. Imai T, Izumi T, Isoda H, et al. Factors influencing blood flow resistance from a large internal carotid artery aneurysm revealed by a computational fluid dynamics model. *Nagoya J Med Sci* 2019; 81: 629–636.
18. Hadad S, Pradhan A, Kadirvel R, et al. Flow reversal in distal collaterals as a possible mechanism of delayed intraparenchymal hemorrhage after flow diversion treatment of cerebral aneurysms. *Front Physiol* 2022; 13: 881627.
19. Itazu T, Enomoto Y, Yamauchi K, et al. A case of delayed intraparenchymal hemorrhage due to hyperperfusion after flow diverter treatment. *J Stroke Cerebrovasc Dis* 2020; 29: 104869.
20. Cebral JR, Mut F, Raschi M, et al. Aneurysm rupture following treatment with flow-diverting stents: computational hemodynamics analysis of treatment. *AJNR Am J Neuroradiol* 2011; 32: 27–33.
21. Kulcsár Z, Houdart E, Bonafé A, et al. Intra-aneurysmal thrombosis as a possible cause of delayed aneurysm rupture after flow-diversion treatment. *AJNR Am J Neuroradiol* 2011; 32: 20–25.
22. Fontaine V, Jacob MP, Houard X, et al. Involvement of the mural thrombus as a site of protease release and activation in human aortic aneurysms. *Am J Pathol* 2002; 161: 1701–1710.
23. Natarajan SK, Lin N, Sonig A, et al. The safety of pipeline flow diversion in fusiform vertebrobasilar aneurysms: a consecutive case series with longer-term follow-up from a single US center. *J Neurosurg* 2016; 125: 111–119.
24. Flemming KD, Wiebers DO, Brown RD Jr, et al. The natural history of radiographically defined vertebrobasilar nonsaccular intracranial aneurysms. *Cerebrovasc Dis* 2005; 20: 270–279.
25. Lesley WS and Rangaswamy R. Balloon test occlusion and endosurgical parent artery sacrifice for the evaluation and management of complex intracranial aneurysmal disease. *J Neurointerv Surg* 2009; 1: 112–120.
