# Supplementary material for: Management strategies and outcomes of basilar trunk aneurysms: a systematic review and meta-analysis
Source: Langenbecks Arch Surg. 2026 Jan 20;411(1):71. doi: 10.1007/s00423-025-03959-3 (PMC12852281; doi:10.1007/s00423-025-03959-3)
Supplement: Supplementary file 1 — Supplementary file1 (ZIP 55327 KB) [file 423_2025_3959_MOESM1_ESM.zip › literature.zip!/meta/Yu 2010.pdf]

Endovascular Treatment of Aneurysms Arising from the Basilar Artery Trunk and Branches

J.-L. YU[1], H.-L.WANG[1], N. XU[1], K. XU[1], B. WANG[2], Q. LUO[1]

[1] Department of Neurosurgery; [2] Department of Radiology, First Hospital of JiLin University; Changchun, Jilin Province, China

Keywords: basilar artery trunk, branch, aneurysm, endovascular treatment

Summary

This study reports our experience with the endovascular treatment of basilar artery (BA) trunk and branch aneurysms. Subjects included 16 patients with BA trunk and branch aneurysms who underwent endovascular treatment in our hospital from October 2000 to October 2009, including four patients with associated arteriovenous malformation (AVM), two with associated moyamoya disease, one with multiple aneurysms at adjacent sites, and one with a distant aneurysm. Endovascular coil embolization, together with stent or balloon assistance when necessary, or while occluding the parent artery was performed. Associated diseases were managed intraoperatively or in the second stage, or treated with gamma knife radiotherapy, or followed up. Two patients with unsuccessful embolization died of re-rupture at the fourth month and fifth month follow-up. The remaining 14 patients reported good outcomes and experienced no re-rupture of either the aneurysm or associated disease. Angiographic follow-ups were conducted for the 14 patients for six to 12 months. Digital subtraction angiography (DSA) examination at the last follow-up showed no recurrence of the BA trunk and branch aneurysms. Together, BA trunk and branch aneurysms should be actively managed via endovascular techniques to prevent serious consequences due to aneurysm rupture and bleeding. Favorable outcomes can be obtained by the proper selection of endovascular treatment regimens.

Introduction

Aneurysms arising from the basilar artery (BA) trunk and branches are a rare clinical entity [1-3]. Because of their deep location between the clivus and the brain stem, complex surrounding anatomical structures, and a dense collection of vital perforating arteries and cranial nerves, surgical access to these aneurysms is difficult with current skull base surgical techniques. Surgical clipping carries a high risk of damaging surrounding brain tissue. Patients presenting with subarachnoid hemorrhage (SAH) due to aneurysm rupture pose a particularly daunting surgical challenge for neurosurgeons because of the small operative space [4-6]. In comparison, endovascular embolization of aneurysms enables surgical operations under direct vision and causes less damage to the nerves and vessels around the aneurysms, thereby overcoming the drawbacks of surgical clipping. Recent advances in stent and balloon systems have further refined endovascular techniques [7-9]. Given that the BA trunk and branch aneurysms are rarely encountered in the clinic, few data are available concerning their endovascular treatment. In the present study, we present our experience with the endovascular treatment of 16 patients with aneurysm arising from the BA trunk and branches treated in our hospital from October 2000 to October 2009.

Materials and Methods

Baseline Data

A total of 16 patients (11 men, five women; mean age: 45.8 years, range 14-64 years) were included in this study. Of the 16 patients, there were 13 patients with SAH alone, two patients with SAH and associated intracerebellar hematoma (IH), and one patient with headache. According to the Hunt-Hess scale, two patients were grade I, 11 patients were grade II, and

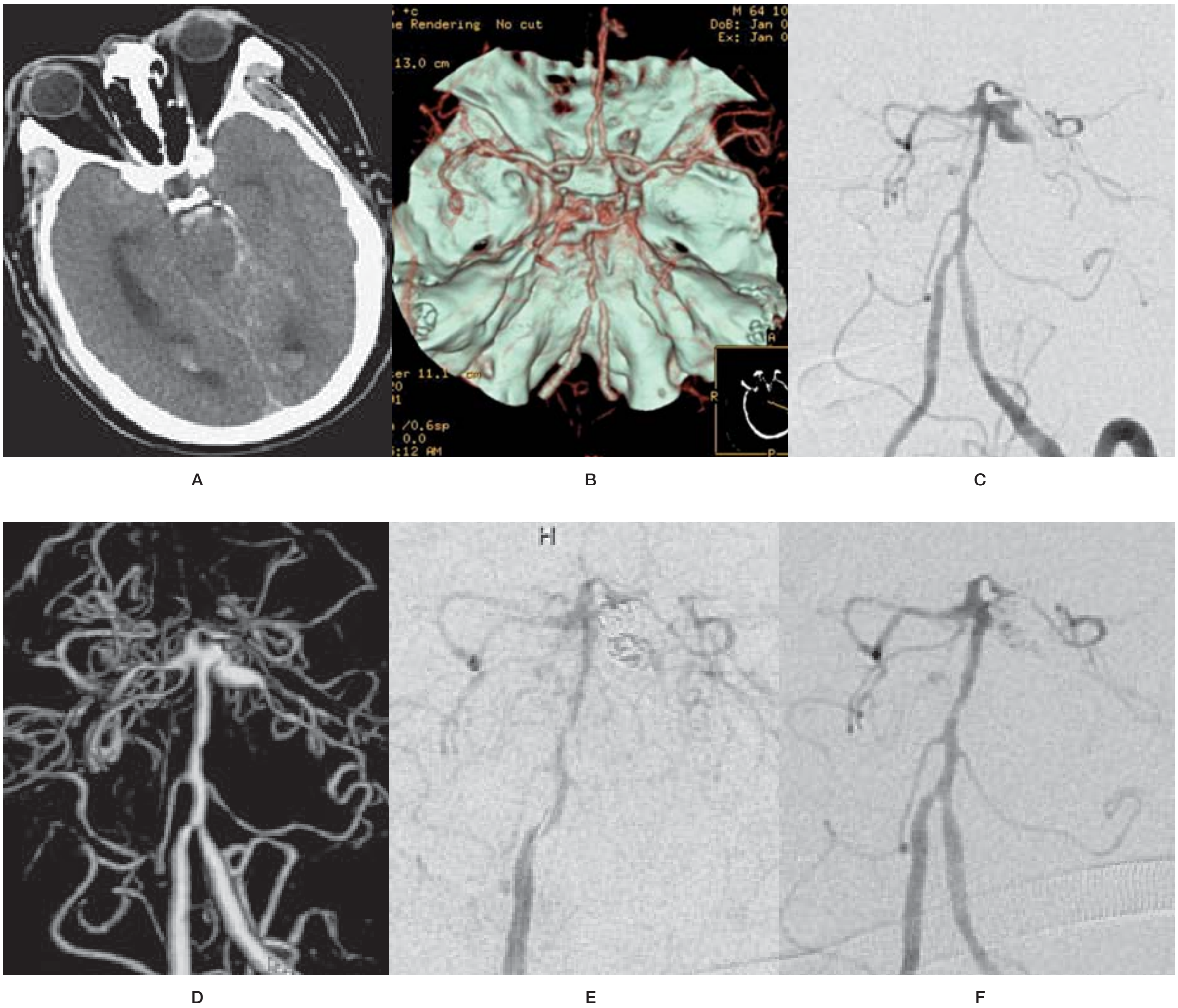

Figure 1 SCA fusiform aneurysm (Case 1). A) CT scan showing SAH and severe bleeding in areas around the interpeduncular cistern. B) CTA showing a fusiform aneurysm at the origin of left SCA and segmental basilar artery due to spasm. C,D) DSA and 3D-DSA of the aneurysm. E) DSA showing occlusion of the aneurysm and the parent artery and subtle neck remnant of the aneurysm. F) Angiographic follow-up examination showing the subtle neck remnant did not grow again.

three patients were grade III. The diagnosis of aneurysm was established using brain computed tomography angiography (CTA) and DSA examinations after disease onset.

Inclusion Criteria

Aneurysms that met the following criteria were enrolled in this study.

– Aneurysms originated from the region of the vertebrobasilar junction to the origin of the superior cerebellar artery (SCA): vertebrobasilar (VB) junction aneurysms, aneurysms arising at the site of a BA fenestration, BA-anterior inferior cerebellar artery (BA-AICA) aneurysms, lateral type berry aneurysms at the BA trunk, and fusiform and dissecting aneurysm involving the BA trunk. These aneurysms were named as BA aneurysms [10].

– Aneurysms localized at the origin of BA branches or were quite near the BA trunk, which looked like the lateral type berry aneurysms at the BA trunk. Therefore in this study this type of aneurysms were pooled together for analysis. These aneurysms were named as BA branch aneurysms.

– Aneurysms were responsible for the hemorrhage. This means that SAH and hematoma focused on the aneurysm site and that the SAH or SAH associated IH was caused by the aneurysm.

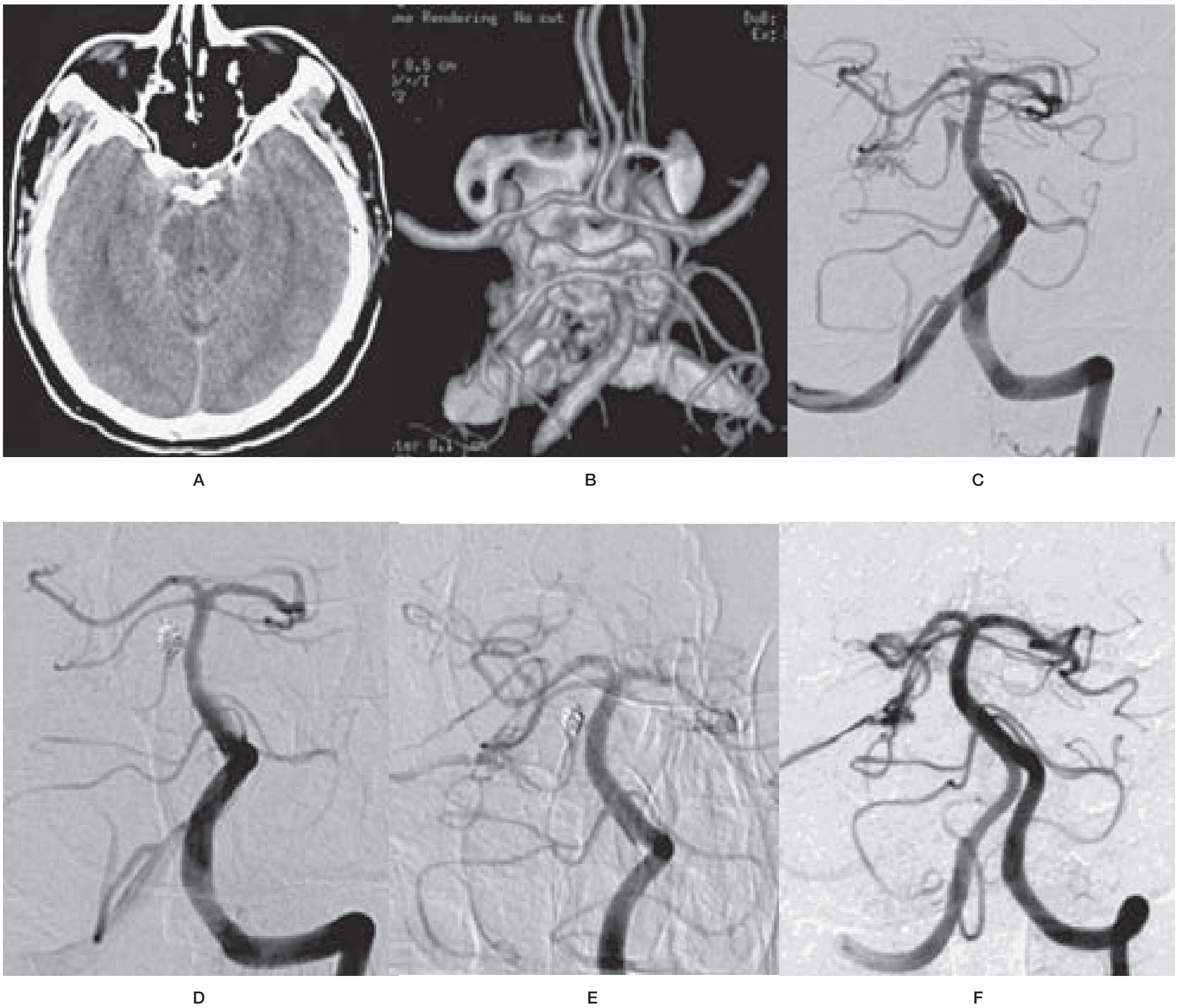

Figure 2 MPB dissecting aneurysm (Case 5). A) CT showing SAH mainly in the right ambient cistern. B,C) CTA and DSA showing an aneurysm at the origin of the right pontine branch of the basilar artery. D) DSA showing embolized aneurysm and patent parent artery was preserved. E) Follow-up DSA showing preserved MPB and AVM. F) DSA showing AVM with Onyx embolization and complete occlusion of parent artery with coils in the 2nd stage.

However those aneurysms localized at the BA branch artery and are far from the origin, including BA bifurcation and vertebral artery (VA) aneurysms, were excluded from this analysis.

Aneurysm Features

Altogether, 16 patients had 16 aneurysms.

Location: (i) 13 cases presented with BA trunk aneurysms, including two cases of BA-SCA aneurysm, one case of BA-middle pontine branch (BA-MPB) aneurysm, one case of middle BA trunk aneurysm, two cases of lower BA trunk aneurysm (one was in the fenestration, and one was lateral type aneurysm), six cases of BA-AICA aneurysm, and one case of BA trunk aneurysm involving the whole BA. (ii) Three cases presented with BA branch aneurysms, including one case of SCA aneurysm, one case of upper pontine branch (UPB) aneurysm, and one case of MPB aneurysm.

Size: Aneurysms were classified as small (<1 cm), large (1-2.5 cm) and giant (>2.5 cm). Of the 16 aneurysms, 13 were small, two were large, and one was giant.

Associated Diseases

In terms of associated disease, there were four cases of AVM, two cases of moyamoya disease, one case of multiple aneurysms at adjacent sites, and one case of distant aneurysm.

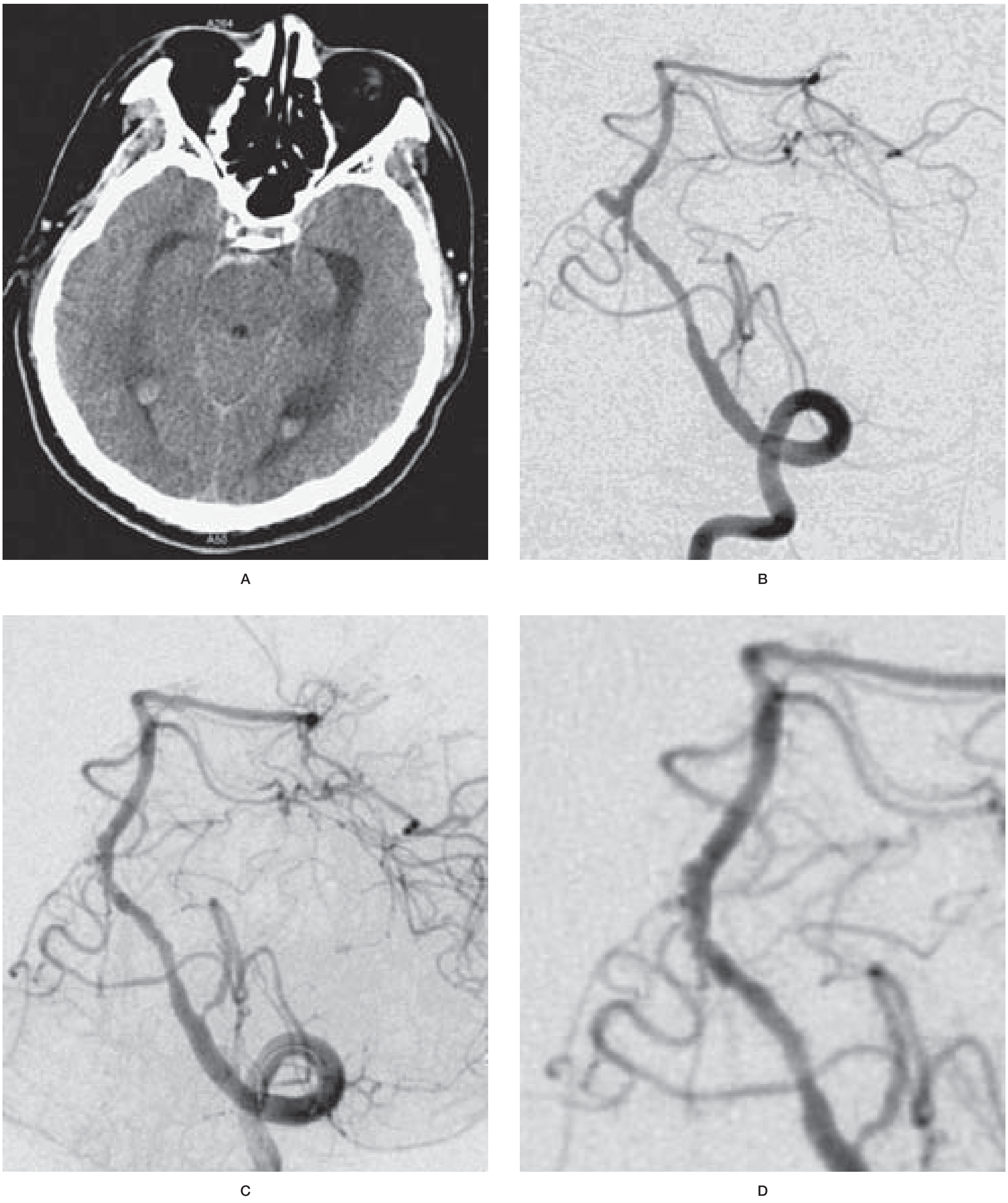

Figure 3 BA-AICA berry aneurysm (Case 10). A) CT showing SAH mainly in front of the ambient cistern and hematocele in the posterior horn of the lateral ventricle. B) DSA showing a side-wall berry aneurysm at the origin of the AICA. C) DSA showing aneurysm occlusion and preserved patent parent artery. D) Follow-up DSA showing no aneurysm recanalization.

Treatment

Preoperative preparation. Patients were treated immediately by coil embolization once the diagnosis was established, but between symptom onset and coil embolization, intravenous infusion of nimotop (4 mL/h, Bayer, Germany) was initiated for patients with SAH and SAH associated IH to prevent vasospasm 11.

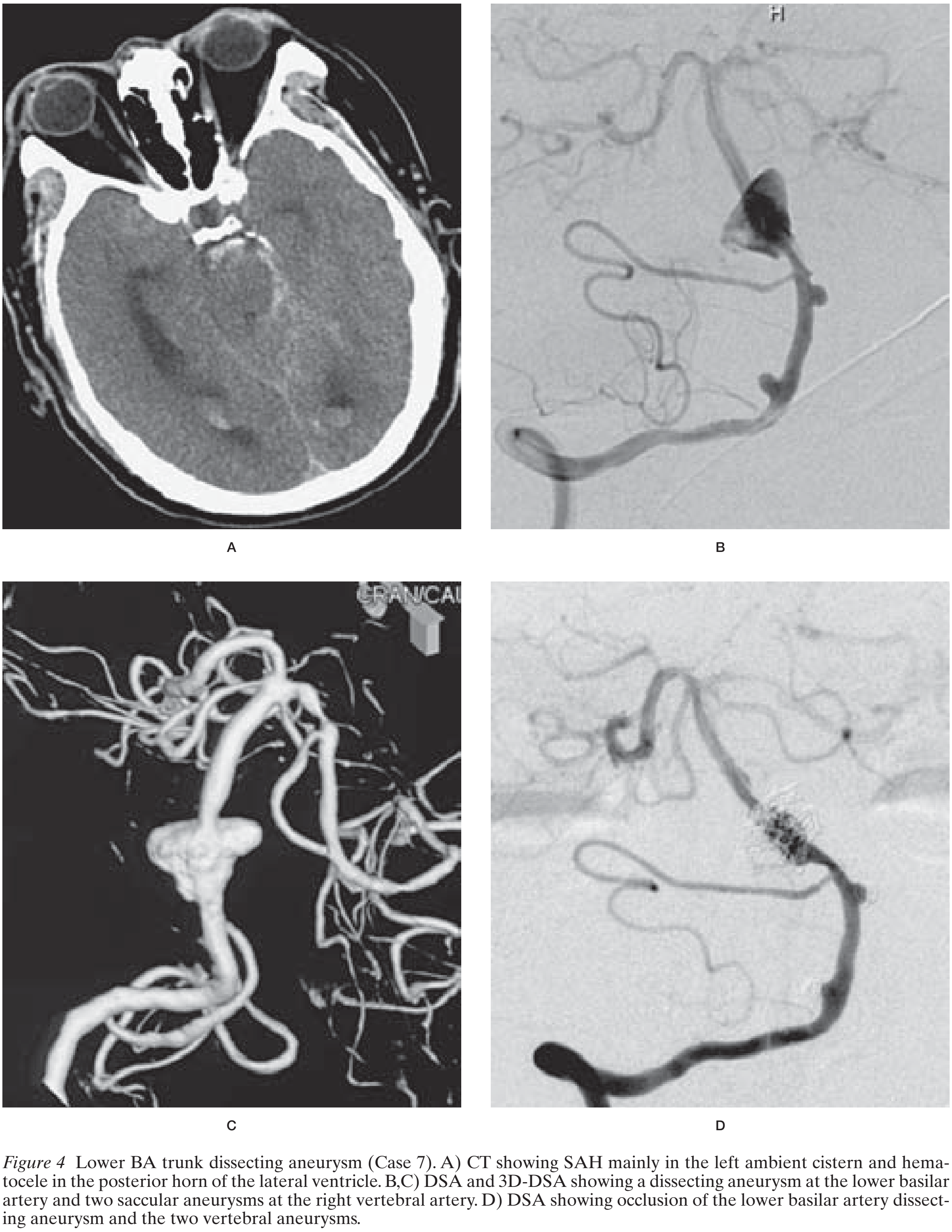

Figure 4 Lower BA trunk dissecting aneurysm (Case 7). A) CT showing SAH mainly in the left ambient cistern and hematocele in the posterior horn of the lateral ventricle. B,C) DSA and 3D-DSA showing a dissecting aneurysm at the lower basilar artery and two saccular aneurysms at the right vertebral artery. D) DSA showing occlusion of the lower basilar artery dissecting aneurysm and the two vertebral aneurysms.

Patients who might need stent-assisted embolization during surgery were administered aspirin (325 mg once in rectum) just at the start of the operation.

Endovascular procedure. Endovascular embolization was performed with the patients under general endotracheal anesthesia. A cannula sheath was placed through the femoral artery

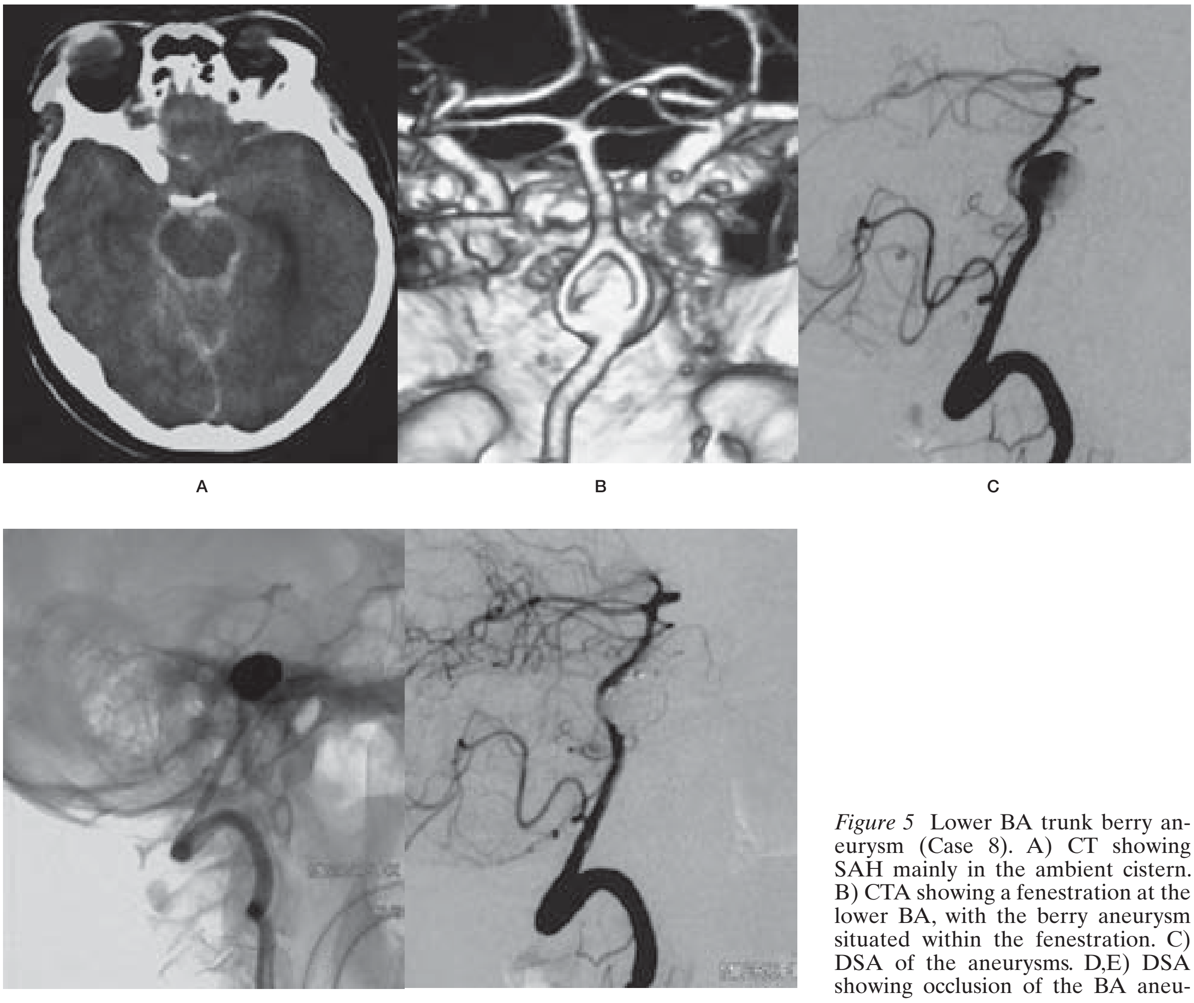

A B C

D E

Figure 5 Lower BA trunk berry aneurysm (Case 8). A) CT showing SAH mainly in the ambient cistern. B) CTA showing a fenestration at the lower BA, with the berry aneurysm situated within the fenestration. C) DSA of the aneurysms. D,E) DSA showing occlusion of the BA aneurysm.

according to the Seldinger technique. Heparin was administered to prevent coagulation, with an initial bolus dose of 5000U and subsequent hourly bolus injection of 1000U. Heparin (5000U/L) was also administered in the flushing saline [11]. A 6-French Envoy guide catheter (Cordis Corporation) was placed in the internal carotid artery. Cerebral angiography, together with three-dimensional reconstruction when necessary, was performed to identify the optimal angle for aneurysm treatment. A SL-10 microcatheter and a Synchro 14 microguidewire (Boston Scientific, Natick, MA, USA) were used to access the aneurysm. Embolization was performed with electrically detachable coils or hydrocoils (including two-dimensional and 3D coils with various degrees of flexibility). For associated AVM that needed to be treated, a liquid embolic material [Onyx (ev3, Irvine, Calif., USA) or Glubran glue (GEM S.R.L., Viareggio, Italy)] was used.

Strategies of coil embolization: (i) BA trunk berry aneurysms were occluded with coils, and the parent artery was preserved whenever possible. Stent or balloon embolization was used when coil embolization alone was insufficient for complete occlusion. (ii) Dissecting or fusiform aneurysms arising from the BA trunk and branches were occluded as much as possible, together with the parent artery. (iii) Associated AVM was also managed during or after surgery. Gamma knife radiotherapy was sometimes used. Follow-ups were performed. Associated moyamoya disease was left untreated.

Postoperative management. Anticoagulation therapy was maintained for five postoperative days by subcutaneous injection of low molecular-weight heparin [11]. Patients who had received

stent implantation continued to take aspirin for at least three months to prevent stent thrombosis [12]. Lumbar drainage of the bloody cerebrospinal fluid was conducted for seven days. An intravenous infusion of nimotop was administered for three weeks to prevent and relieve cerebral vasospasm [13].

Results

The 16 patients had 15 ruptured aneurysms and 1 unruptured aneurysm. No rupture occurred to the associated lesion. Therefore, aneurysms were treated as a priority in AVM cases.

Treatment of the Aneurysms

Anatomical results

The extent of aneurysm occlusion was classified as complete occlusion, neck remnant, or incomplete occlusion. Complete occlusion referred to the occlusion of an aneurysm with no detectable residual neck on any angiographic projection. Neck remnant referred to lesions with residual filling of part of the neck of the aneurysm, and incomplete occlusion was defined as the presence of the contrast agent filling the body and/or dome of the aneurysm because of incomplete packing. The same classification strategy was used to evaluate follow-up angiograms. The changes were observed on follow-up [14].

Of the 11 cases of BA trunk berry aneurysms, seven were treated with coil embolization alone, two with balloon-assisted coil embolization, one with stent-assisted coil embolization. Complete occlusion was achieved in these ten aneurysms. Although the remaining one case also received an advanced stent to cover the aneurysm, coil embolization was failed in this patient and anticoagulation was therefore not administered for this patient.

Of the two cases of BA trunk dissecting aneurysms, one involving the whole BA was treated with coil embolization and BA occlusion, and complete occlusion was achieved. The other aneurysm was localized at middle BA trunk and treated with stent-assisted coil embolization, resulting in an incomplete occlusion.

Of the three cases of BA branch aneurysms, one case of SCA fusiform aneurysm was treated with coil embolization and parent artery occlusion, yielding a neck remnant. One case of MPB dissecting aneurysm with AVM was first given an incomplete occlusion. But during later AVM treatment with Onyx, the aneurysm and parent artery were occluded completely. One case of UPB dissecting aneurysm with moyamoya disease had an unsuccessful treatment because of the failure to transfer the coil system into the sac.

Treatment of Associated Diseases

Of the four AVM cases, in addition to aneurysm coil embolization, one case was treated with embolization using Glubran2 glue, one was treated with embolization using Onyx glue, one was treated with Onyx glue in the second stage, and one was treated with gamma knife radiotherapy.

Moyamoya disease in two cases was left untreated. The one patient with adjacent multiple aneurysms in the VA was concurrently treated with stent-assisted coil embolization. The one patient with associated middle cerebral artery (MCA) aneurysm underwent surgical clipping of the distant aneurysm in the second stage.

Prognosis

The prognosis of the endovascular treatment was determined by the Glasgow outcome scale (GOS). The five categories of the scale were as followed: 5, good recovery; 4, moderate disability; 3, severe disability; 2, vegetative status; and 1, death.

All patients were followed-up for one to two years. Embolization treatment failed in one patient with moyamoya disease, who later experienced re-rupture of the aneurysm and died at the fifth month follow-up (GOS score 1). One patient who had received unsuccessful treatment with stent covering the aneurysm alone developed rebleeding and died at the fourth month follow-up (GOS score 1). The remaining 14 patients reported a favorable outcome and experienced no rupture from the aneurysm or associated disease, and all had a GOS score of 5.

Angiographic Findings

The 14 patients who had a favorable outcome were followed-up with DSA examinations for six to 12 months.

Aneurysm: DSA examination showed no recurrence in the ten berry aneurysms of the BA trunk, one dissection involving the whole BA, and one dissection at MPB. One dissection of middle BA trunk with incomplete occlusion

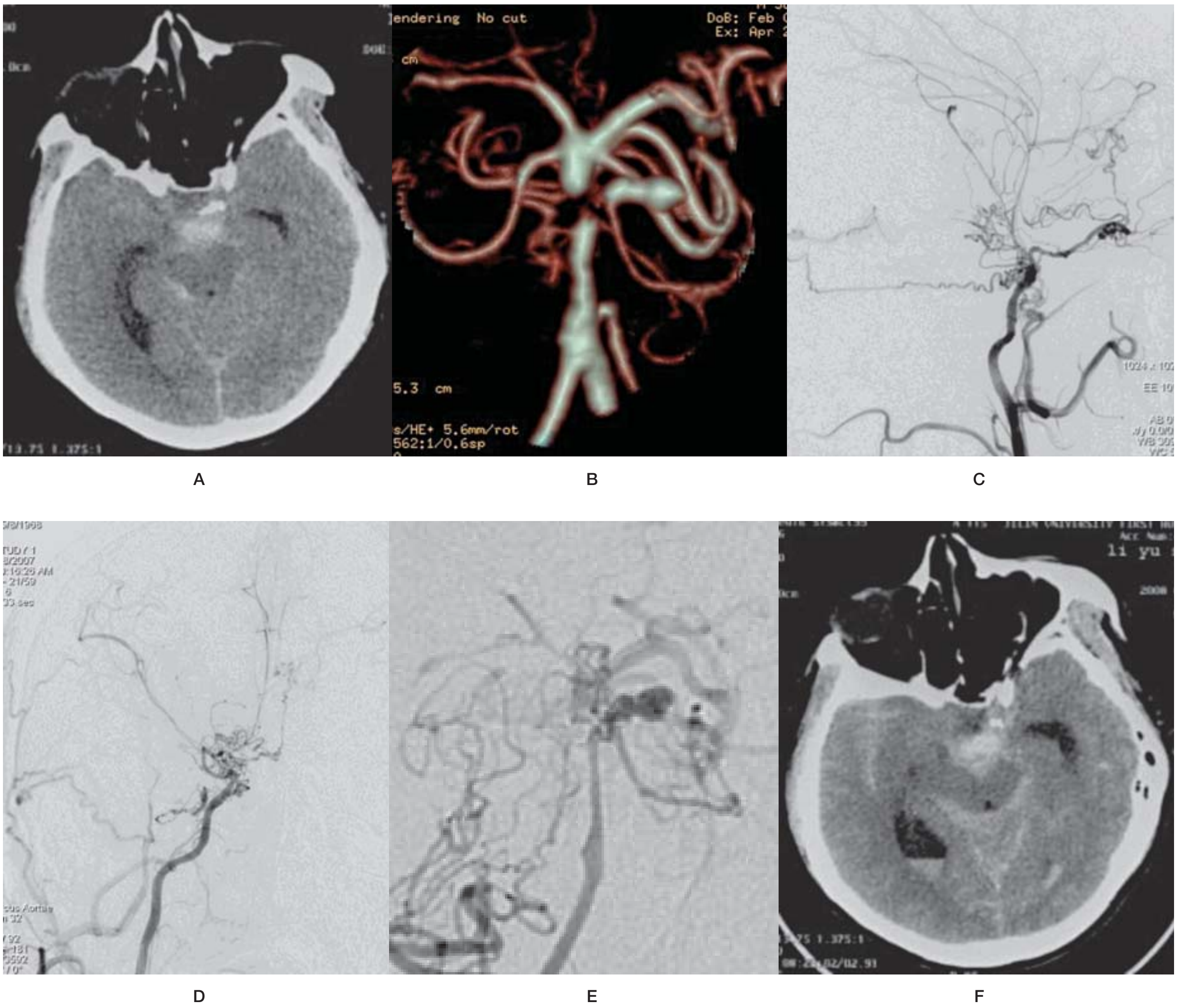

Figure 6 UPB dissecting aneurysm (Case 4). A) CT showing SAH mainly in the interpeduncular cistern. B) CTA showing an aneurysm at the left upper pontine branch of the basilar artery. C,D) DSA revealing the presence of moyamoya disease. E) DSA showing that coil embolization failed to occlude the aneurysm. F) CT showing re-rupture of the aneurysm at the 5-month follow-up.

and one SCA fusiform aneurysm with subtle neck remnant did not grow again.

Associated diseases: AVM did not enlarge or bleed after proper treatment. Moyamoya disease experienced no bleeding. Associated multiple aneurysms had no recurrence.

Discussion

Posterior circulation aneurysms account for 10-20% of all intracranial aneurysms. The most common site of origin for such aneurysms is the basilar tip, followed by the origin of the posterior inferior cerebellar artery [15,16]. However, aneurysms occurring on the BA trunk between the vertebrobasilar junction and the SCA are rarely seen in the clinic, accounting for < 1% of all intracranial aneurysms [1,2,11]. These aneurysms may occur at the origin of the SCA or pontine branch or AICA, at the vertebrobasilar junction (including fenestration of the BA), or at the lateral BA trunk. Now these aneurysms are defined as BA trunk aneurysms 10,16. In the clinic some aneurysms localize at the BA branch beginning or are quite near the BA trunk, we call these aneurysms as BA branch aneurysms. Because this type of aneurysms looks like the lateral type saccular aneurysms at the BA trunk and it is necessary to distin-

Table 1 **Clinical features of the patients.**

Case	Sex/ Age	Presenting symptom	HUNT-HESS	Location	Size	Shape	Associated disease	Treatment	Management of associated disease	Outcome (GOS)
1	M/64	SAH	III	SCA origin	Small	Fusiform	None	Coil+PAO	——	5
2	M/54	SAH	II	BA-SCA	Small	Berry	AVM	Coil	GLUBRAN embolization	5
3	M/51	SAH	II	BA-SCA	Small	Berry	AVM	Coil	Onyx18 embolization	5
4	M/39	SAH	II	UPB origin	Small	Dissecting	Moyamoya	Failed	Untreated	1
5	M/44	SAH	II	MPB origin	Small	Dissecting	AVM	Coil	Onyx embolization in 2nd stage	5
6	M/55	SAH	I	BA-MPB	Small	Berry	None	HyperGlide balloon+Coil	——	5
7	M/50	SAH	II	Middle BA trunk	Large	Dissecting	Vertebral an-eurysm	Neuroform stent+ Coil	Neuroform stent+ Coil	5
8	F/47	SAH	II	Lower BA trunk (fenestration)	Small	Berry	None	Coil	——	5
9	F/49	SAH	II	Lower BA trunk (lateral type)	Small	Berry	MCA aneurysm	HyperGlide balloon+Coil	Clipping in 2nd stage	5
10	M/60	SAH	II	BA-AICA	Small	Berry	None	Coil	——	5
11	M/44	SAH+IH	III	BA-AICA	Small	Berry	None	Coil	——	5
12	F/42	SAH	III	BA-AICA	Large	Berry	None	Failed (Only Neuroform stent)	——	1
13	M /27	SAH+IH	II	BA-AICA	Small	Berry	AVM	Coil	Gamma knife radiotheray	5
14	F/48	SAH	II	BA-AICA	Small	Berry	None	Coil	——	5
15	F/44	SAH	II	BA-AICA	Small	Berry	Moyamoya	Neuroform stent+ Coil	Untreated	5
16	M/14	Headache	I	Whole BA	Giant	Dissecting	None	Coil+PAO	——	5

F, female; M, male. SAH, subarachnoid hemorrhage. IH:intracerebellar hematoma. BA, Basilar artery. SCA, superior cerebellar artery. UPA, upper potine artery. MPA, middle potine artery. AICA, anterior inferior cerebellar artery. AVM, arteriovenous malformation. MCA, middle cerebral artery. PAO, parent artery occlusion.

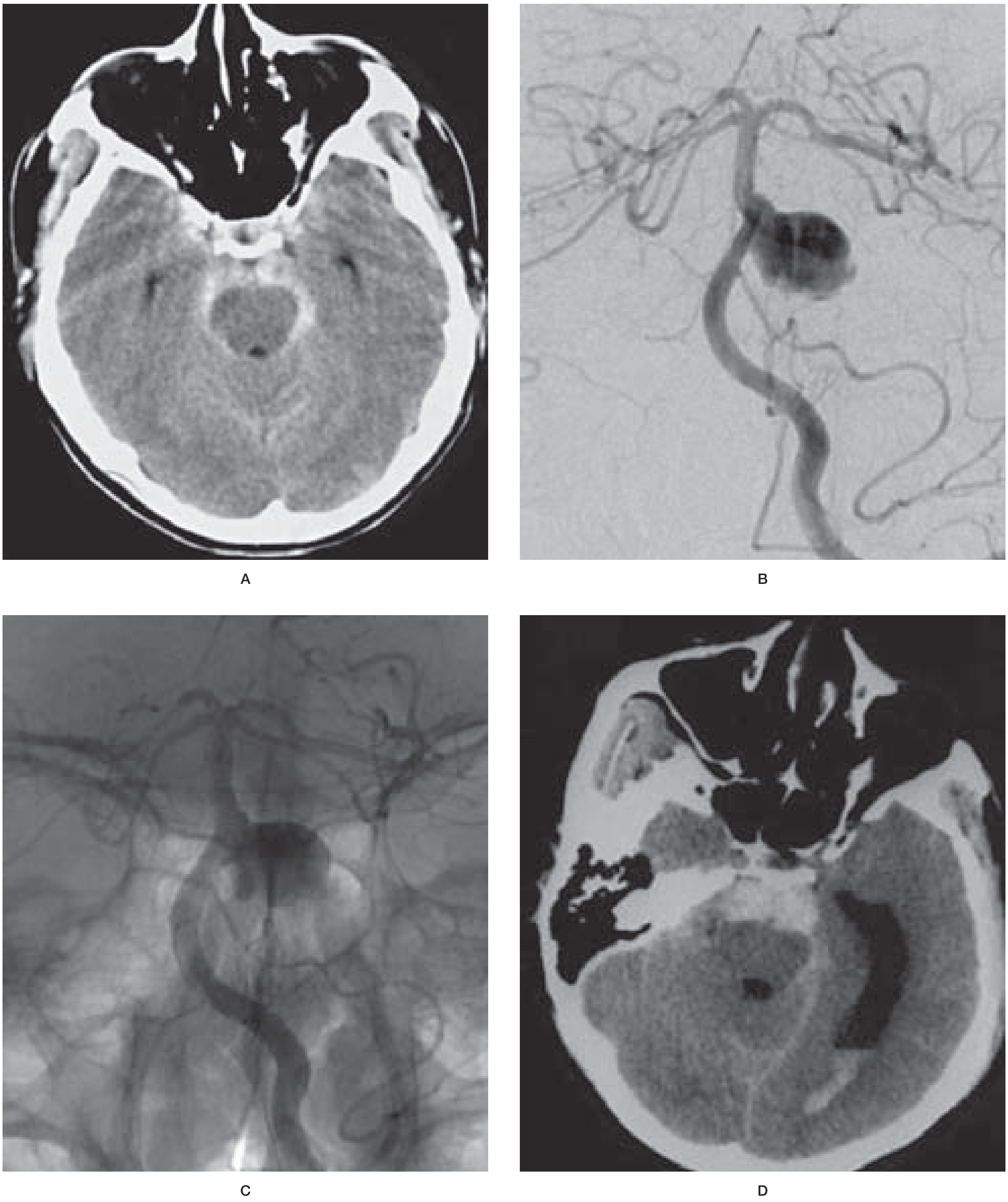

Figure 7 BA-AICA berry aneurysm (Case 12). A) CT showing SAH mainly in the ambient cistern. B) DSA showing a giant aneurysm at the origin of the AICA. C) DSA showing that the implanted stent covered the aneurysm neck. D) CT showing re-rupture of the aneurysm at the 4-month follow-up.

guish them from lateral type berry aneurysms of BA. Therefore, in this study we put this type of aneurysms together to analyze. Because of their rare occurrence, few studies are available regarding endovascular treatment of such aneurysms. Here, we present our single-center experience of endovascular treatment of 16 such cases collected over ten years.

The imaging of BA trunk aneurysms is very complex because different sizes and types of aneurysms may be seen [10,16,17]. Uda et al. reported a series of 39 patients with 41 berry aneurysms who underwent endovascular treatment. This is one of the largest cohorts with BA trunk berry aneurysms, in which 68.3% of 41 were small [14]. In our study, ten of the 11 BA trunk berry aneurysms were small, which is consistent with the study by Uda et al. Apart from berry aneurysms, fusiform and dissecting aneurysms are occasionally observed [18,19], which make the endovascular treatment even more complex. In this study, two BA dissections were involved, of which one was large and the other was giant, in agreement with the study results of Kim et al. [19] In this study, three aneurysms were at the origin of BA branches, of which two were dissecting and one was fusiform. Their imaging features were consistent with Sanchez-Mejia's report [20]. Accordingly, treatment for BA trunk and branch aneurysms is often technically challenging. In addition, previous studies have also established that BA trunk aneurysms frequently coexist with other abnormalities, such as associated persistent primitive sublingual artery, proatlantal intersegmental artery, BA fenestration, or fibromuscular dysplasia [10,21]. With the exception of the SCA and the AICA, the BA trunk branches are usually small in diameter [10]. Associated diseases may lead to hemodynamic changes and thinned arterial walls, predisposing these branches to otherwise rare aneurysms. But none of the abovementioned associated diseases occurred in the 16 patients of this study, although four patients had associated AVM and two moyamoya disease. The high proportion of BA aneurysm in association with AVM and moyamoya disease is different from previous reports involving more patients [10,14]. This discrepancy may be due to the possibility that in the same period we treated more patients who had AVM and moyamoya disease associated with aneurysm than before; therefore, the patient population in this study was somewhat distorted compared with previous studies including more patients. It is also likely that previous studies may exclude the associated diseases from their analyses. Nevertheless, inclusion of associated diseases in our present study is justified because associated diseases complicate BA trunk aneurysm treatment and warrant a comprehensive approach that considers both the aneurysm and the associated disease. In the present study, there were four AVM patients with coexisting aneurysm. These aneurysms originated from the origin of the feeding artery and were type II aneurysms according to the criteria established by Perata et al [22]. Hemodynamic factors are likely to play a role in the development of the aneurysm. In such cases the high capacity and speed of blood flow continually damage the endothelia of the blood vessels, eventually leading to the rupture of the vascular internal elastic membrane, and aneurysms [23]. The cause of BA trunk and branch aneurysms in moyamoya disease is similar with those aneurysms in AVM, because these aneurysms are formed due to increased stress on the vessel wall from the high flow imposed by occlusion of the anterior circulation [24]. These aneurysms are flow-related and easy to re-rupture, and timely treatment is warranted.

Surgical clipping of the BA trunk aneurysm presents a challenging task for the neurosurgeon because of the blockage of the petrous bone on both sides of the aneurysm [25,26]. Since the clinical application of GDC in 1991, the treatment efficacy for aneurysms has been significantly improved, thanks to direct endovascular vision and minimal damage to nerves and vessels adjacent to the aneurysms [10]. With the recent advent of balloon and stent systems and developments in embolic materials, endovascular treatment has demonstrated even greater advantages over traditional surgical clipping [27]. In 2002, the International Subarachnoid Aneurysm Trial group concluded that endovascular coiling is more likely to offer survival benefits than surgical clipping [28]. Accordingly, the 16 patients in this study were treated with endovascular intervention. Because of the complex types for BA trunk and branch aneurysms, different strategies of coil embolization should be adopted. For berry aneurysms of BA trunk, it is easy to treat with coils embolization, with or without the assistance of balloon or stent, and usually the parent artery can be preserved [10]. Ten berry aneurysms of BA trunk undergoing this treatment achieved complete occlusion and had a good outcome. However, treatment for dissecting and fusiform aneurysms is different from that for berry aneurysms, because it is necessary to occlude dissecting or fusiform aneurysm and the parent artery at the same time [29]. Simultaneous occlusion of the aneurysm and the parent artery is more feasible for BA branch aneurysms, since the parent artery in this context more likely to be tolerant to occlusion. However, when a dissecting aneurysm lo-

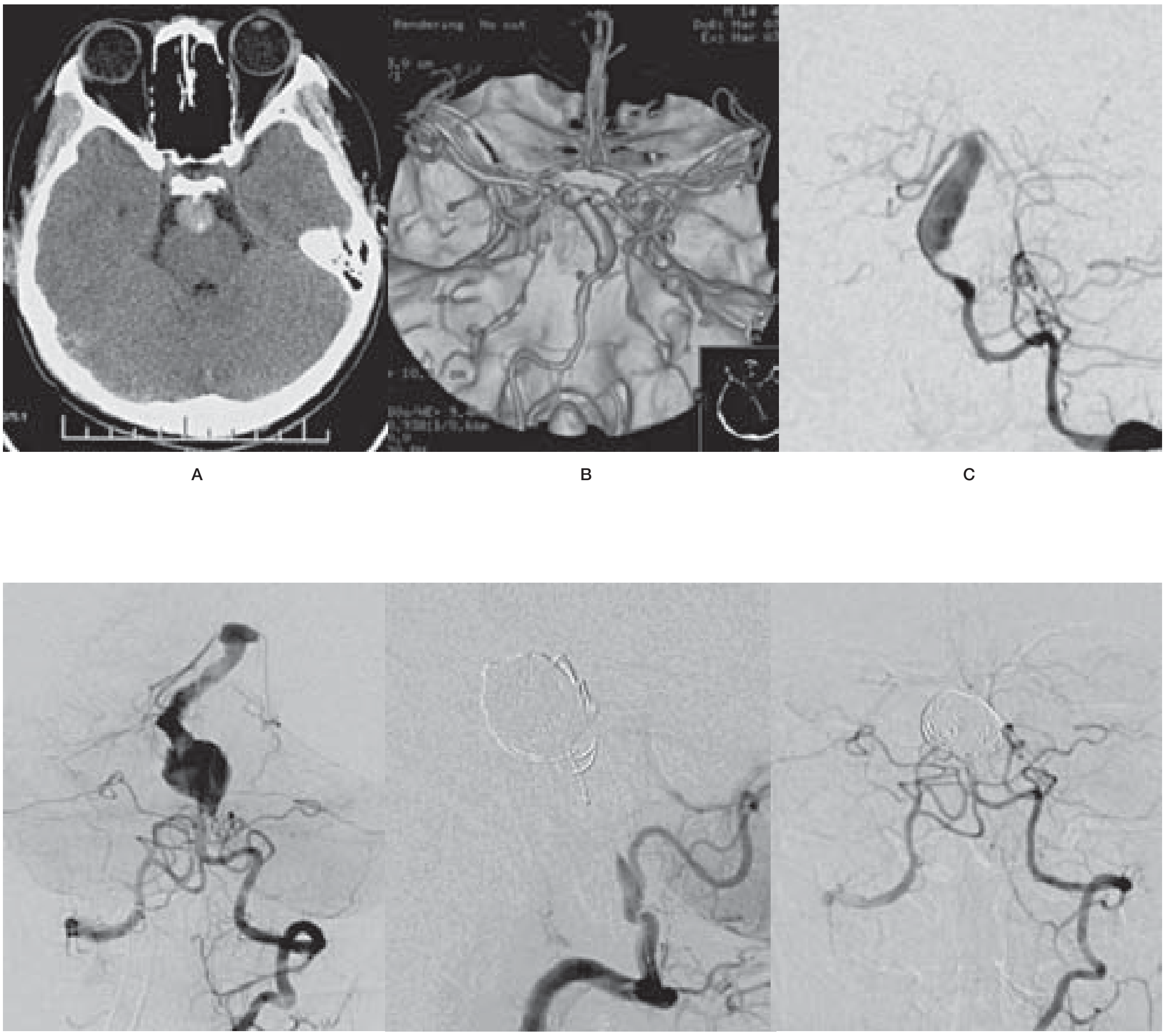

A B C

D E F

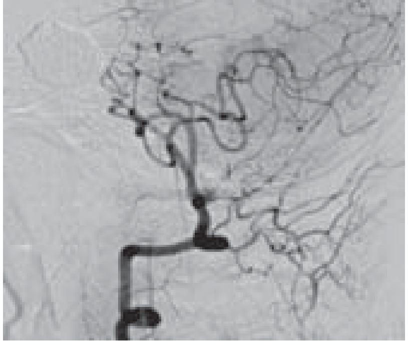

G

Figure 8 Whole BA dissecting aneurysm (Case 16). A) CT shows a lesion anterior to the brain stem. B) CTA depicts a severe and fusiform enlargement in the whole BA with eccentric filling defects extending to the top of the BA. C) DSA demonstrates double lumen of the BA in the coronal section, retention of contrast medium in the venous and the later period of arterial phases. D) DSA reveals more remarkable enlargement in the proximal portion of BA which have extended to left VA. E) After coil embolization, angiographies show no filled aneurysm image on left VA angiography. F,G) Follow-up DSA demonstrates complete obliteration of the dissection by showing a large signal void in the BA area.

calizes at the BA trunk, is it feasible to sacrifice the BA? And if the BA dissection is incorrectly treated, postoperative hemodynamic change may alter the nature history and accelerate the rupture [30]. For dissecting and fusiform aneurysm of BA branches in the present study, after concurrently occluding the aneurysm and the parent artery, good outcomes (GOS 5) were obtained, with no complication. After reviewing previous study concerning BA branch aneurysms, we found it was feasible to occlude the aneurysm and the parent artery altogether and the outcome was satisfactory [20,31]. But for the two cases with BA trunk dissection, according to previous and recent reports, the treatment of choice was to embolize the ruptured dissection with the assistance of stenting while preserving the BA. It is very dangerous to sacrifice BA because there is often lack of good collateral circulation in most patients [32]. Therefore, one such case was treated with the above method, resulting in an incomplete occlusion. The other dissection involving the whole BA was treated with complete coil embolization to occlude BA due to uncontrolled progressive expansion of the lesion and a good outcome was obtained. At present little is still known about the ideal therapeutic measures for BA dissections. However, considering the continually downward extension and dilatation of the lesion, coil embolization is a good, and often inevitable, choice to avoid potentially serious consequences resulted from fatal rupture [33,34]. In this study, all 14 cases with successful treatent reported good outcomes, all with a GOS score of 5, although two cases among them had incomplete occlusion. However, two patients receiving unsuccessful embolization died of re-rupture at the fourth month and fifth month follow-up, respectively. Of the two patients who died, one patient had a UPB dissection comorbid with moyamoya and had an unsuccessful treatment because of the failure to transfer the coil system into the sac. The other received unsuccessful treatment with stent covering the aneurysm alone. Since the aneurysm in this patient did not undergo coil embolization, anticoagulation therapy was not administered to the patient, who experienced rebleeding after the surgery. These findings also suggest that conservative treatment or stent therapy alone is insufficient to provide a good outcome for the vertebrobasilar artery trunk and branch aneurysms [35,36,37,38]. Eight of the 16 cases displayed an associated disease that increased treatment difficulty. Treatment for cases with associated disease should give priority to the aneurysm, to prevent hemodynamic changes (resulting from management of the associated disease) from causing aneurysm rupture. For the four cases with associated AVM, different treatment strategies were used depending on the risk factors for AVM rupture. The focus in these cases was on minimizing AVM rupture risk rather than on overemphasizing the significance of radiological cure. Two of the four cases were embolized in the first stage, one was embolized in the second stage, and one received gamma knife radiotherapy. The moyamoya disease in two cases was left untreated. The two cases with associated aneurysm at another site were coiled in the first stage (one case) or surgically clipped in the second stage (one case). No rupture or bleeding occurred to the associated lesions during follow-ups. Our results indicate that endovascular treatment is associated with good efficacy in most patients, though there remain some issues. The issue of greatest concern is possible occlusion of the parent artery while coiling the aneurysm. Since the parent artery has many perforating vessels supplying the brain stem, parent artery occlusion will lead to severe complications. Rebleeding during or after endovascular coiling must also be considered [10,14,15]. A study by Uda et al. including 41 aneurysms in 39 patients reported that the incidence of ischemic complications was about 10.3% and that no complications resulted from intraoperative or postoperative rebleeding 14. Another study with 52 upper BA aneurysms in 49 patients revealed a 2% incidence of ischemic complications and a 4.2% incidence of postoperative rebleeding [39]. In contrast with these previous studies, we found no ischemia or bleeding complications during surgery and no rupture during the one to two-year follow-up period in 14 of our 16 cases. However, several cases presented with associated AVM and moyamoya disease, a finding not seen in other studies. Our relatively small case size may partly account for the inconsistency. Our present study helps to outline a more complete picture of BA trunk and branch aneurysms and adds to the accumulating experience in managing such lesions.

Conclusions

In conclusion, we recommend that the BA trunk and branch aneurysms should be aggressively managed with endovascular treatment to

prevent the serious consequences resulting from aneurysm rupture. Comorbidities such as AVM, moyamoya disease, or aneurysms at other sites should be considered when designing a suitable treatment strategy for the BA trunk and branch aneurysm so as to ensure a favorable outcome.

References

1 Peerless SJ, Hernesniemi JA, Gutman FB, et al. Early surgery for ruptured vertebrobasilar aneurysms. J Neurosurg. 1994; 80: 643-649.
2 Rice BJ, Peerless SJ, Drake CG. Surgical treatment of unruptured aneurysms of the posterior circulation. J Neurosurg. 1990; 73: 165-173.
3 Hernesniemi J, Karatas A, Ishii K, et al. Anteroinferior cerebellar artery aneurysms: surgical approaches and outcomes--a review of 34 cases. Neurosurgery. 2005; 57: E601.
4 Mizoi K, Yoshimoto T, Takahashi A, et al. Direct clipping of basilar trunk aneurysms using temporary balloon occlusion. J Neurosurg. 1994; 80: 230-236.
5 Seifert V, Stolke D. Posterior transpetrosal approach to aneurysms of the basilar trunk and vertebrobasilar junction. J Neurosurg. 1996; 85: 373-379.
6 Sekhar LN, Kalia KK, Yonas H, et al. Cranial base approaches to intracranial aneurysms in the subarachnoid space. Neurosurgery. 1994; 35:472-481.
7 Guglielmi G, Viñuela F, Sepetka I, et al. Electrothrombosis of saccular aneurysms via endovascular approach. Part 1: Electrochemical basis, technique, and experimental results. J Neurosurg. 1991; 75: 1-7.
8 Guglielmi G, Viñuela F, Dion J, et al. Electrothrombosis of saccular aneurysms via endovascular approach. Part 2: Preliminary clinical experience. J Neurosurg. 1991; 75: 8-14.
9 Strother CM. Historical perspective. Electrothrombosis of saccular aneurysms via endovascular approach: part 1 and part 2. Am J Neuroradiol. 2001; 22: 1010-1012.
10 Higa T, Ujiie H, Kato K, et al. Basilar artery trunk saccular aneurysms: morphological characteristics and management. Neurosurg Rev. 2009; 32 (2): 181-191.
11 Hallacq P, Piotin M, Moret J. Endovascular occlusion of the posterior cerebral artery for the treatment of p2 segment aneurysms: retrospective review of a 10-year series. Am J Neuroradiol. 2002; 23: 1128-1136.
12 Lubicz B, Bandeira A, Bruneau M, et al. Stenting is improving and stabilizing anatomical results of coiled intracranial aneurysms. Neuroradiology. 2009; 51: 419-425.
13 Klimo P Jr, Kestle JR, MacDonald JD, et al. Marked reduction of cerebral vasospasm with lumbar drainage of cerebrospinal fluid after subarachnoid hemorrhage. J Neurosurg. 2004; 100: 215-224.
14 Uda K, Murayama Y, Gobin YP, et al. Endovascular treatment of basilar artery trunk aneurysms with Guglielmi detachable coils: clinical experience with 41 aneurysms in 39 patients. J Neurosurg. 2001; 95: 624-632.
15 Redekop GJ, Durity FA, Woodhurst WB. Management-related morbidity in unselected aneurysms of the upper basilar artery. J Neurosurg. 1997; 87: 836-842.
16 Sugita K, Kobayashi S, Takemae T, et al. Aneurysms of the basilar trunk. J Neurosurg. 1987; 66: 500-505.
17 Liu L, Jiang C, He H, et al. Delayed thrombosis of the basilar artery after stenting for a basilar trunk dissection aneurysm. A case report and review of the literature. Interventional Neuroradiology 2010; 16: 77-82.
18 Crowley RW, Evans AJ, Kassell NF, et al. Endovascular treatment of a fusiform basilar artery aneurysm using multiple “in-stent stents”. Technical note. J Neurosurg Pediatr. 2009; 3(6): 496-500.
19 Kim BM, Suh SH, Park SI, et al. Management and clinical outcome of acute basilar artery dissection. Am J Neuroradiol. 2008; 29: 1937-1941.
20 Sanchez-Mejia RO, Lawton MT. Distal aneurysms of basilar perforating and circumferential arteries. Report of three cases. J Neurosurg. 2007; 107: 654-659.
21 Kimura T, Onda K, Arai H. Multiple basilar artery trunk aneurysms associated with fibromuscular dysplasia. Acta Neurochir (Wien). 2004; 146: 79-81.
22 Perata HJ, Tomsick TA, Tew JM Jr. Feeding artery pedicle aneurysms: association with parenchymal hemorrhage and arteriovenous malformation in the brain. J Neurosurg. 1994; 80: 631-634.
23 Lasjaunias P, Piske R, Terbrugge K, et al. Cerebral arteriovenous malformations (C. AVM) and associated arterial aneurysms (AA). Analysis of 101 C. AVM cases, with 37 AA in 23 patients. Acta Neurochir (Wien). 1988; 91 (1-2): 29-36.
24 Kawaguchi S, Sakaki T, Morimoto T, et al. Characteristics of intracranial aneurysms associated with moyamoya disease. A review of 111 cases. Acta Neurochir (Wien). 1996; 138: 1287-1294.
25 Seifert V. Direct surgery of basilar trunk and vertebrobasilarjunction aneurysms via the combined transpetrosal approach. Neurol Med Chir. 1998; 38 (Suppl): 86-92.
26 Aziz KM, van Loveren HR, Tew JM Jr, et al. The Kawase approach to retrosellar and upper clival basilar aneurysms. Neurosurgery. 1999; 44: 1225-1234.
27 Van Rooij WJ, Sluzewski M, Menovsky T, et al. Coiling of saccular basilar trunk aneurysms. Neuroradiology. 2003; 45:19-21.
28 Molyneux AJ, Kerr RS, Yu LM, et al. International Subarachnoid Aneurysm Trial (ISAT) Collaborative Group. International subarachnoid aneurysm trial (ISAT) of neurosurgical clipping versus endovascular coiling in 2143 patients with ruptured intracranial aneurysms: a randomised comparison of effects on survival, dependency, seizures, rebleeding, subgroups, and aneurysm occlusion. Lancet. 2005; 366: 809-817.
29 Xavier J, Vasconcelos C, Cruz R, et al. Endovascular treatment of dissecting aneurysms of the posterior cerebral artery. Acta Med Port. 2001; 14: 65-70.
30 Kai Y, Hamada J, Morioka M, et al. Successful treatment of a ruptured dissecting basilar artery aneurysm. Case report. J Neurosurg. 2004; 100: 1072-1075.
31 Chaloupka JC, Putman CM, Awad IA. Endovascular therapeutic approach to peripheral aneurysms of the superior cerebellar artery. Am J Neuroradiol. 1996; 17: 1338-1342.
32 Amin-Hanjani S, Ogilvy CS, Buonanno FS, et al. Treatment of dissecting basilar artery aneurysm by flow reversal. Acta Neurochir (Wien). 1997; 139: 44-51.
33 Nakahara T, Satoh H, Mizoue T, et al. Dissecting aneurysm of basilar artery presenting with recurrent subarachnoid hemorrhage. Neurosurg Rev. 1999; 22: 155-158.
34 Yoshimoto Y, Hoya K, Tanaka Y, et al. Basilar artery dissection. J Neurosurg. 2005; 102: 476-481.
35 Hashimoto M, Johkura K, Ichikawa T, et al. Conservative treatment of ruptured vertebrobasilar dissecting aneurysm. Neurol Sci. 2008; 29: 241-244.

36 Ramgren B, Cronqvist M, Romner B, et al. Vertebrobasilar dissection with subarachnoid hemorrhage: a retrospective study of 29 patients. Neuroradiology. 2005; 47: 97-104.
37 Kim BM, Suh SH, Park SI, et al. Management and clinical outcome of acute basilar artery dissection. Am J Neuroradiol. 2008; 29: 1937-1941.
38 Massoud TF, Guglielmi G, Viñuela F, et al. Saccular aneurysms in moyamoya disease: endovascular treatment using electrically detachable coils. Surg Neurol. 1994; 41: 462-467.
39 Redekop GJ, Durity FA, Woodhurst WB. Management-related morbidity in unselected aneurysms of the upper basilar artery. J Neurosurg. 1997; 87: 836-842.

Dr Luo Qi
Department of Neurosurgery, First Hospital of Ji Lin University
130021, Changchun, China
Tel.: 86-431-85612572
E-mail: luoqi@mail.jlu.edu.cn
