# Supplementary material for: Management strategies and outcomes of basilar trunk aneurysms: a systematic review and meta-analysis
Source: Langenbecks Arch Surg. 2026 Jan 20;411(1):71. doi: 10.1007/s00423-025-03959-3 (PMC12852281; doi:10.1007/s00423-025-03959-3)
Supplement: Supplementary file 1 — Supplementary file1 (ZIP 55327 KB) [file 423_2025_3959_MOESM1_ESM.zip › literature.zip!/meta/Zang 2015.pdf]

·临床研究·

血管内技术治疗椎基底动脉夹层动脉瘤的疗效分析

臧贻征　王志刚　王成伟　张媛　丁璇　王小飞

【摘要】 **目的**　评价血管内技术治疗椎基底动脉夹层动脉瘤(VBDA)的有效性和安全性,分析不同血管内技术治疗 VBDA 的疗效差异。**方法**　回顾性分析山东大学第二医院神经外科 2007 年 1 月至 2015 年 12 月采用血管内技术治疗 45 例 VBDA 患者的临床资料。根据是否保持载瘤动脉通畅,治疗方法分为重建性手术和破坏性手术。重建性手术包括支架辅助微弹簧圈栓塞术(24 例)和单纯支架置入术(8 例),破坏性手术采取动脉瘤 + 载瘤动脉近端栓塞术(13 例)。**结果**　围手术期并发症发生率和死亡率分别为 11.1 %(5/45)和 4.4%(2/45),其中重建性手术 1 例发生术中动脉瘤破裂出血、死亡,1 例术中载瘤动脉内血栓形成,溶栓后无神经功能障碍,上述并发症均发生在支架辅助微弹簧圈栓塞术,单纯支架置入术无并发症发生;破坏性手术 3 例术后发生脑缺血,1 例死亡,2 例重度残疾。长期随访改良 Rankin 量表(mRS)评分:0~2 分 41 例,3~6 分 4 例。重建性手术组较破坏性手术组预后好(96.9% 比 76.9%),差异有统计学意义($P<0.05$);并发症发生率低(6.2% 比 23.1%),但差异无统计学意义($P>0.05$)。**结论**　血管内技术治疗 VBDA 安全、有效,重建性手术较破坏性手术并发症发生率低,预后较好。

【关键词】 夹层动脉瘤；　椎基底动脉；　血管内治疗；　栓塞

基金项目:山东大学第二医院青年基金(Y2013010052)

Clinical analysis of endovascular strategies in the treatment of vertebrobasilar dissecting aneurysms

Zang Yizheng, Wang Zhigang, Wang Chengwei, Zhang Yuan, Ding Xuan, Wang Xiaofei. * The Second Hospital of Shandong University, Jinan 250033, China*

Correspongding author: Zang Yizheng, Email: zangyizheng@126.com

【**Abstract**】 **Objective**　To evaluate the efficacy and safety of endovascular strategies for vertebrobasilar dissecting aneurysm (VBDA) and analyze advantages and disadvantages of different endovascular methods for the treatment of VBDA through long-term follow-up. **Methods**　Retrospectively analyzing was used for the clinical information of 45 cases of vertebrobasilar dissecting aneurysms treated with endovascular methods in our department from January 2007 to December 2015. The treatment modalities were classified into reconstructive surgery and destructive surgery that were based on parent artery preservation. Reconstructive surgery included stent-assisted coil embolization (24 patients) and the placement of single or multiple overlapping stents (8 patients). Destructive surgery was coil embolization combined with proximal coil trapping of the dissected segment of the parent artery (CE + PT, 13 patients). **Results**　The incidence of perioperative complications was 11.4%, and the mortality associated with these complications was 4.4%. In the patients undergoing reconstructive surgery, one patient suffered from aneurysm rerupture and died, one patient had intraoperative thrombi in the parent artery and resulted in a complete patency after thrombolysis was performed with Tirofiban. Both perioperative complications occurred in patients treated with stent-assisted coil embolization. No complications occurred in patients treated with single or multiple overlapping stents placements. Perioperative brainstem ischemia occurred in three patients who were treated with destructive surgery, and caused one death and two severe disabilities. Clinical outcome evaluations were performed using the Modified Rankin Scale and resulted in the following scores: 0-2 for 41 patients, 3-6 for 4 patients. The prognosis of reconstructive group was better than that of destructive group, with statistical differences ($P<0.05$). The incidence of perioperative complications in

DOI:10.3760/cma.j.issn.0376-2491.2016.41.011

作者单位:250033 济南,山东大学第二医院神经外科(臧贻征、王成伟、张媛、丁璇、王小飞);山东大学齐鲁医院青岛院区神经外科(王志刚)

通信作者:臧贻征,Email:zangyizheng@126.com

reconstructive group was lower than that in destructive group, without significant differences ($P > 0.05$). **Conclusions** Endovascular treatment is effective and safe for VBDAs. The incidence of perioperative complication in reconstructive group is lower and the prognosis is better than that in destructive group.

【**Key words**】 Dissecting aneurysm; Vertibrobasilar artery; Endovascular treatment; Embolization

Fund program: Youth Fund of the Second Hospital of Shandong University (Y2013010052)

椎基底动脉夹层动脉瘤(vertebrobasilar dissecting aneurysm, VBDA)致死、致残率高,治疗难度大,是神经外科一大难题。本研究通过对血管内技术治疗的45例VBDA患者进行术后长期临床和血管造影随访,探讨其有效性、安全性,并评价不同血管内技术的疗效差异。现分析报道如下。

对象与方法

1. 对象:2007年1月至2015年12月山东大学第二医院神经外科应用血管内技术治疗45例VBDA患者,其中男28例,女17例,年龄25～73岁,平均50.9岁。首发症状蛛网膜下腔出血31例,后循环缺血症状12例,症状性癫痫1例,头痛1例。

2. 影像学资料:45例患者术前均行CT、数字减影血管造影(DSA)检查,11例行高分辨率磁共振检查。CT显示31例蛛网膜下腔出血,14例无特殊表现。DSA显示载瘤动脉对称或不对称扩张,伴有近端或远端狭窄38例,双腔征2例,不规则瘤样扩张5例。高分辨率磁共振显示血管壁内血栓6例,内膜瓣2例。

3. 治疗方式:根据是否保留载瘤动脉通畅分为两类,即保留载瘤动脉的重建性手术和闭塞载瘤动脉的破坏性手术。重建性手术采取支架辅助微弹簧圈栓塞术以及单纯支架置入术,后者又包括单层支架置入、多层支架置入等;破坏性手术采取动脉瘤+载瘤动脉近端栓塞术。

4. 疗效判定标准:治疗的有效性用动脉瘤的栓塞程度评估,用Raymond分级标准来评价动脉瘤的栓塞程度,治疗的安全性用死亡率、围手术期并发症来评估,用修订的Rankin评分(mRS)来评价患者的临床预后,0～2分为预后好,3～6分为预后差。

5. 统计学分析:用SPSS 19.0软件。分类资料用例数和构成比表示,频数分布差异统计学意义比较用χ^2检验,以$P<0.05$为差异有统计学意义。

结 果

本组病例,围手术期死亡2例,重度残疾2例,并发症发生率和死亡率分别为11.1%(5/45)和4.4%(2/45);除上述4例患者外,1例失随访,40例患者分别于术后0.5～72个月行血管造影检查,平均随访时间12.2个月。动脉瘤1次治疗完全栓塞30例(75.0%),长期随访mRS评分:0～2分41例(91.1%),3～6分4例(8.9%)。

一、重建性手术组围手术期并发症及长期随访

重建性手术32例,其中支架辅助微弹簧圈栓塞24例,单纯支架置入术8例。术后即刻造影显示,动脉瘤完全栓塞18例,次全栓塞5例,不全栓塞9例,单纯支架置入术后均显示动脉瘤内造影剂滞留,为不全栓塞。1例术中载瘤动脉内血栓形成,动脉内应用尿激酶接触溶栓,效果差,改用替罗非班效果好,造影显示载瘤动脉通畅,术后无神经系统症状恶化;1例较早期病例,术中栓塞时动脉瘤破裂出血,出现双侧瞳孔散大,虽然给予中和肝素、脱水、止血等积极治疗但仍死亡。长期随访血管造影显示动脉瘤完全栓塞20例,复发10例,其中5例再次行支架辅助微弹簧圈栓塞术,5例继续随访,1例失随访,无新发动脉瘤再次出血和死亡病例。预后mRS评分0～2分31例(96.9%),3～6分1例(3.1%)。

二、破坏性手术组围手术期并发症及长期随访

破坏性手术,即动脉瘤+载瘤动脉近端栓塞术13例:术后即刻造影显示完全栓塞2例,次全栓塞10例,不全栓塞1例。其中3例(6.2%),术后均神志清楚,言语正确,四肢活动正常,但术后4～7 h内发生呼吸停止,应用呼吸机辅助通气,行CT检查无颅内出血,考虑为脑干缺血。2例治疗后自主呼吸恢复,但呈昏迷状态,重度残疾,1例因家属放弃治疗后死亡。除上述3例,长期随访造影显示其余10例,显示动脉瘤完全栓塞。临床预后mRS评分0～2分10例(76.9%),3～6分3例(23.1%)。

三、重建性手术组与破坏性手术组围手术期并发症及长期随访指标比较

重建性手术组预后比破坏性手术组预后好(96.9%比76.9%),差异有统计学意义($P<0.05$);重建性手术组较破坏性手术组并发症发生率较低(6.2%比23.1%),但差异无统计学意义($P>0.05$),表1。

四、典型病例介绍

患者男,29岁,因活动后突发后颈部疼痛、头晕

Q2xvdWRWZXJzaW9uLQo?

伴恶心、呕吐 25 d 入院，CT 无阳性发现，DSA 示左椎动脉夹层动脉瘤。见图 1。

讨　论

VBDA 多表现为蛛网膜下腔出血、脑干缺血或周围脑组织压迫等症状。蛛网膜下腔出血易导致呼吸循环衰竭，且再出血率高达 30%，再出血后死亡率可达 46%[1]。由 VBDA 引起的后循环缺血多呈逐渐进展趋势，同时存在破裂出血的风险。虽然动脉瘤的自发愈合也有报道，但其更倾向于膨胀发展[2]，因此症状性 VBDA 必须尽快予以处置[3]。

由于 VBDA 手术暴露困难，动脉瘤没有明确瘤颈，而且经常有重要穿支动脉起源于动脉瘤，开颅手术风险较大，并发症发生率高，且颅内外血管重建的长期随访也显示预后较差[4]。随着介入技术和材料的发展，血管内技术治疗在治疗 VBDA 方面的作用越来越重要，但 VBDA 具体的血管内治疗方式仍存在争议，对其治疗后长期随访报道也相对较少。

本组患者采用不同的血管内技术治疗 VBDA，总的围手术期并发症发生率为 11.1%，与手术操作相关的并发症仅 1 例(2.9%)，死亡率为 4.4%，长期随访预后 mRs 评分 0～2 分者 91.1%，一次性血管内治疗动脉瘤完全闭塞率 75.0%，效果满意。

治疗 VBDA 理想的目标是既保持载瘤动脉通畅又栓塞了动脉瘤，如闭塞载瘤动脉，间接供血血管可能发生病变，如动脉粥样硬化性狭窄或血管损伤，当一侧椎动脉闭塞后对侧也可发生夹层动脉瘤[5]，而此时的间接血管对患者来说又尤为重要，所以不管 VBDA 首发症状如何，也无论动脉瘤的位置、血流动力学如何，支架辅助微弹簧圈栓塞术均为符合生理需求的治疗方法。动脉瘤栓塞可防止再出血或动脉瘤继续发展，并能降低因对侧逆行供血而导致动脉瘤复发的概率[6]。支架置入后，血流动力学发生改变，可促进动脉瘤腔内血栓形成。对于同侧优

表 1　两组椎基底动脉夹层动脉瘤患者围手术期并发症及长期随访指标比较[例(%)]

组别	例数	围手术期并发症			随访 mRS 评分		随访造影结果			
		无	缺血	再破裂	0～2 分	3～6 分	100% 闭塞	复发	失访	死亡
重建性手术组	32	30(93.8)	1(3.1)	1(3.1)	31(96.9)	1(3.2)	20(62.5)	10(31.3)	1(3.1)	1(3.1)
破坏性手术组	13	10(76.9)	3(23.1)	0(0.0)	10(76.9)	3(23.1)	10(76.9)	0(0.0)	2(15.4)	1(7.7)
P 值		0.089			0.033		0.076			

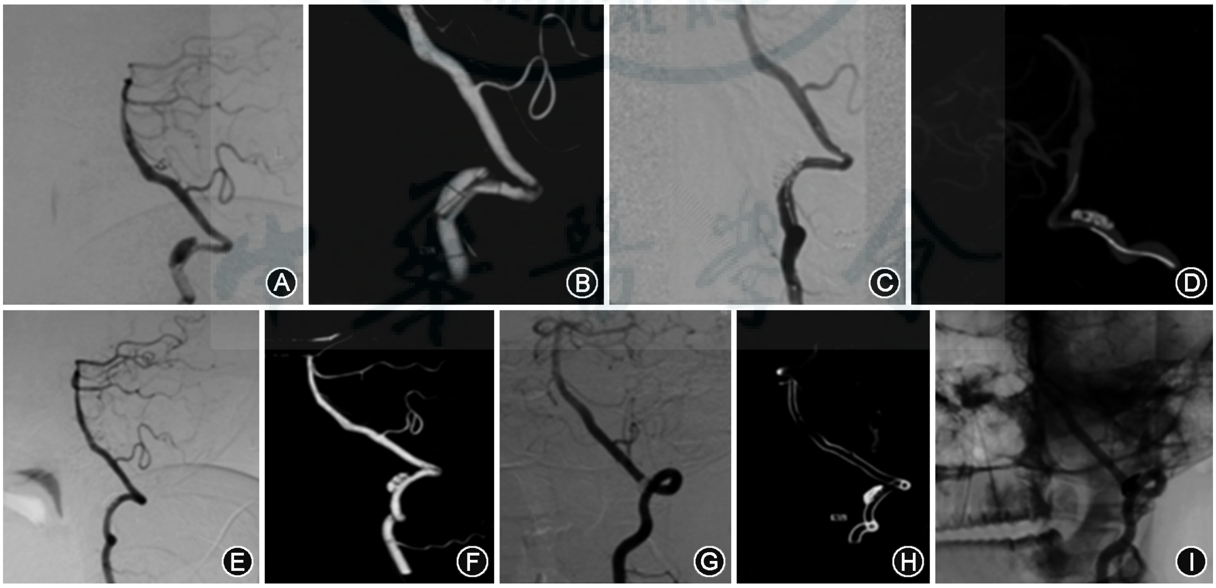

图 1　椎动脉夹层动脉瘤患者 DSA 情况。A，B：左侧椎动脉造影显示左侧椎动脉 V3 段可见夹层动脉瘤，呈现双腔征，动脉瘤大小约 4.6 mm×15.0 mm；C，D：首先将支架(Enterprise 4.5 mm×28 mm)输送到位，送入微微弹簧圈一枚成篮满意后半释放支架，再依次送入 8 枚微微弹簧圈将动脉瘤完全栓塞后，完全释放支架再置入 1 枚支架(Enterprise 4.5 mm×22 mm)完全覆盖瘤颈，动脉瘤完全栓塞，支架在位良好，载瘤动脉通畅及各分支动脉均通畅；E，F：术后 1.5 个月复查见左侧椎动脉夹层脉瘤栓塞致密，夹层动脉瘤消失，微弹簧圈及支架在位良好，椎动脉管腔完全恢复正常，椎动脉、基底动脉及各分支显影正常；G～I：术后 8 个月余复查，左侧椎动脉夹层动脉瘤栓塞致密，夹层动脉瘤消失，微弹簧圈及支架在位良好，椎动脉管腔完全恢复正常，动脉瘤与椎动脉间可见明显隔离带，椎动脉、基底动脉及各分支显影正常

Q2xvdWRWZXJzaW9uLQo?

势供血、起始部累及小脑后下动脉、对侧椎动脉缺如或发育不全的年轻患者,保持载瘤动脉通畅就更加重要。但多层支架及血流导向装置置入术后良好的造影复查结果,对弹簧圈致密栓塞的必要性提出质疑,可能在闭塞动脉瘤方面,支架置入术后血流动力学的改变比弹簧圈致密栓塞的作用更加重要,但这仍需大规模临床试验证实。

近年来单纯支架置入治疗颅内夹层动脉瘤或假性动脉瘤引起人们重视。利用粒子成像测速仪和流体力学研究发现[7],支架置入后血流动力学发生改变,可防止动脉瘤的破裂和继续发展,且降低孔隙率及载瘤血管曲率可使血流动力学向好的方向改变;在稍后的动物模型中也证实了支架能改变动脉瘤内血流的形式,由螺旋型改为杂乱型,这个现象可能诱发血栓进而形成动脉瘤的永久闭塞。一旦动脉瘤内血栓出现,支架内侧面的内膜新生使支架内侧面血管发生重建[8],将动脉瘤排除于循环系统外。

本组共8例采用单纯支架置入,7例长期随访,6(85.7%)例多层支架置入的患者动脉瘤完全闭塞,无一例发生围手术期并发症,效果令人满意。So等[9]做的26例单纯支架置入患者随访显示动脉瘤完全闭塞率71.4%,造影改善率86.1%,无一例发生再出血,多层支架组造影改善率明显高于单层支架组。这提示单纯支架置入治疗VBDA也可取得较好的治疗效果,而且动脉瘤内未行弹簧圈栓塞,操作相对简单,减少了薄弱易碎的动脉瘤壁栓塞时破裂的风险,降低并发症发生率。多层支架或孔隙率低的支架,由于降低了孔隙率,增加了金属覆盖率,更加有效,但尚需更大规模的研究及长期的随访。

动脉瘤+载瘤动脉近端栓塞术虽然前期得到了广泛的应用和较好的长期效果,然而严重的并发症也多有报道,主要包括脑干缺血损伤或逆行性的动脉瘤再通[3]。重建性技术近期得到长足发展,然而,许多作者仍在报道他们在载瘤动脉牺牲手术上的经验,青睐于良好的手术效果(预防远期的破裂和出血),并认为如果侧支血供允许,其为首选治疗方案。但本组3例对侧椎动脉均发育良好,球囊闭塞实验阴性,载瘤动脉闭塞后数小时内还是发生脑干缺血的严重并发症,既往也有报道[10]。本组病例显示,破坏性手术较重建性手术围手术期并发症发生率高,预后差,所以建议首选重建性手术方式。

对于重建性手术均存在困难、风险较高或经济条件不允许的患者,如有良好的代偿、球囊闭塞实验阴性,也可行动脉瘤+载瘤动脉近端栓塞术,但需向患者家属详细讲明潜在风险。为减少脑干缺血的发生,我们将手术方式予以改良,术中我们没有即刻完全闭塞载瘤动脉,而是部分闭塞,减少瘤腔及载瘤动脉内的血流,使血栓缓慢形成并为侧支循环的建立提供时间,由于血流倒流和侧支血管的代偿,可减少相关血管供血区域缺血的发生,小脑后下动脉受累时这一点就更加重要。本组病例采用上述方法,无动脉瘤再次破裂发生。

总之,血管内技术治疗VBDA安全、有效,重建性手术较破坏性手术并发症发生率低,预后好。但是,由于VBDA治疗风险高,难度大,治疗费用高,预后可能不佳,神经外科医师应根据每个患者的动脉瘤位置、发病形式、血流动力学情况、不同患者及家属对病情的理解程度及家庭经济情况等综合分析,制定精准的个体化的治疗方案,达到最优疗效。

参考文献

[1] Oelerich M, Stogbauer F, Kurlemann G, et al. Craniocervical artery dissection: MR imaging and MR angiographic findings[J]. Eur Radiol, 1999, 9(7): 1385-1391.
[2] Lylyk P, Cohen JE, Ceratto R, et al. Combined endovascular treatment of dissecting vertebral artery aneurysms by using stents and coils[J]. J Neurosurg, 2001, 94(3): 427-432. DOI: 10.3171/jns.2001.94.3.0427.
[3] Ahn JY, Han IB, Kim TG, et al. Endovascular treatment of intracranial vertebral artery dissections with stent placement or stent-assisted coiling[J]. AJNR Am J Neuroradiol, 2006, 27: 1514-1520.
[4] Steinberg GK, Drake CG, Peerless SJ. Deliberate basilar or vertebral artery occlusion in the treatment of intracranial aneurysms. Immediate results and long-term outcome in 201 patients[J]. J Neurosurg, 1993, 79(2): 161-173.
[5] Zhao WY, Krings T, Alvarez H, et al. Spontaneous mirror dissections of cervicocephalic arteries. Pathomechanical considerations[J]. Interv Neuroradiol, 2006, 12(1): 73-78.
[6] Wang J, Sun Z, Bao J, et al. Endovascular management of vertebrobasilar artery dissecting aneurysms[J]. Turk. Neurosurg, 2013, 23(3): 323-328. DOI: 10.5137/1019-5149.
[7] Yuki I, Murayama Y, Vinuela F. Endovascular management of dissecting vertebrobasilar artery aneurysms in patients presenting with acute subarachnoid hemorrhage[J]. J Neurosurg, 2005, 103(4): 649-655. DOI: 10.3171/jns.2005.103.4.0649.
[8] Hans FJ, Krings T, Moller-Hartmann W, et al. Endovascular treatment of experimentally induced aneurysms in rabbits using stents: a feasibility study[J]. Neuroradiology, 2003, 45(7): 430-434. DOI: 10.1007/s00234-003-1008-y.
[9] So TY, Mitchell PJ, Dowling RJ, et al. Efficacy, complications and clinical out-come of endovascular treatment for intracranial intradural arterial dissections[J]. Clin Neurol Neurosurg, 2014, 117: 6-11. DOI: 10.1016/j.clineuro.2013.11.015.
[10] Albuquerque FC, Fiorella DJ, Han PP, et al. Endovascular management of intracranial vertebral artery dissecting aneurysms [J]. Neurosurg Focus, 2005, 18(2): E3.

(收稿日期:2016-08-02)
(本文编辑:刘小梅)

Q2xvdWRWZXJzaW9uLQo?
