# Supplementary material for: Management strategies and outcomes of basilar trunk aneurysms: a systematic review and meta-analysis
Source: Langenbecks Arch Surg. 2026 Jan 20;411(1):71. doi: 10.1007/s00423-025-03959-3 (PMC12852281; doi:10.1007/s00423-025-03959-3)
Supplement: Supplementary file 1 — Supplementary file1 (ZIP 55327 KB) [file 423_2025_3959_MOESM1_ESM.zip › literature.zip!/meta/Zhang 2018.pdf]

Accepted Manuscript

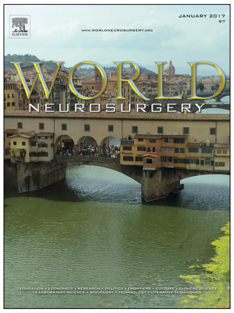

Treatment of symptomatic dolichoectatic vertebrobasilar aneurysms: a single-centre experience in 12 patients

Hongwu Zhang, MD, Xioaguang Tong, MD

PII: S1878-8750(18)31654-1

DOI: 10.1016/j.wneu.2018.07.176

Reference: WNEU 8741

To appear in: *World Neurosurgery*

Received Date: 2 April 2018

Revised Date: 19 July 2018

Accepted Date: 21 July 2018

Please cite this article as: Zhang H, Tong X, Treatment of symptomatic dolichoectatic vertebrobasilar aneurysms: a single-centre experience in 12 patients, *World Neurosurgery* (2018), doi: 10.1016/j.wneu.2018.07.176.

This is a PDF file of an unedited manuscript that has been accepted for publication. As a service to our customers we are providing this early version of the manuscript. The manuscript will undergo copyediting, typesetting, and review of the resulting proof before it is published in its final form. Please note that during the production process errors may be discovered which could affect the content, and all legal disclaimers that apply to the journal pertain.

ACCEPTED MANUSCRIPT

Treatment of symptomatic dolichoectatic vertebrobasilar aneurysms: a single-centre experience in 12 patients

Hongwu Zhang, MD[1], Xioaguang Tong, MD[2]

1. 2016 Level of Graduate of Tianjin Medical University, Tianjin, 300070, China

2. Department of Neurosurgery, Tianjin Huanhu Hospital, Tianjin, 300050, China

Corresponding Author:

Xiaoguang Tong

Address: Department of Neurosurgery, Tianjin Huanhu Hospital, 6 Ji Zhao Road, Jin Nan District, Tianjin, 300050, China.

Telephone: +86 13820088121

E-mail: hongwu1984teda@126.com

Abbreviation List:

BA=basilar artery; DSA=digital subtraction angiography;

mRS=modified Rankin Scale; PCA=posterior cerebral artery;

VA=vertebral artery; STA=superficial temporal artery;

IMA=internal maxillary artery; OA=occipital artery;

RA=radial artery; PICA=posterior inferior cerebellar artery;

PCoA=posterior communicating artery; SCA=superior cerebellar artery;

CHD=coronary heart disease; HLP=hyperlipidaemia;

SAH=subarachnoid haemorrhage; CTA=computed tomography angiography;

ACT=activated clotting time

ACCEPTED MANUSCRIPT

Abstract

Objective. To report our single-centre experience in the treatment of dolichoectatic vertebrobasilar aneurysms.

Methods. We retrospectively reviewed the data, including clinical features, strategies of managing aneurysms, and follow-up results, of 12 patients who were treated at the Tainjin Huanhu Hospital.

Results. Of the 12 patients, one patient was treated with medication, four patie nts were treated using stent reconstruction, and seven patients were treated via revascularization (open surgery). The clinical symptoms of the patients who we re treated with medication and stent reconstruction were stable. The patients w ho were treated with revascularization suffered from pneumonia, and one patien t who presented with intracranial haematoma was treated via a second cranioto my. One patient suffered from new cerebral infraction. Postoperative follow-up from 5-67 months indicated that 2 patients (16.7%) had died (modified Rankin Scale (mRS) score of 6), and both were treated with revascularization. Seven patients had mRS scores of 0-2, and the other 3 patients had mRS scores of 4 -5.

Conclusions. If the condition of the patients is stable and benign, they can be treated with medication or stent reconstruction. Given the serious complications that can be caused by open surgery, care must be taken when selecting the patients to be treated

ACCEPTED MANUSCRIPT

using open surgery, but more studies are needed to support this conclusion. The key points are to avoid aneurysm thrombosis and maintain patency of the perforators.

Keywords. Vertebrobasilar Artery, Dolichoectasia, Reconstruction, Bypass

Introduction

Dolichoectatic vertebrobasilar aneurysms are rare heterogeneous arterial aneurysms associated with significant morbidity and mortality. If left untreated, 80% of patients with dolichoectatic vertebrobasilar aneurysms either become severely disabled or die within 5 years.[10,21,23] However, treatment of dolichoectatic vertebrobasilar aneurysms is challenging for neurosurgeons, because of three life-threatening complications associated with this arterial disorder: thromboembolism causing brain infarction, arterial rupture with subarachnoid haemorrhage (SAH), and arterial enlargement with symptomatic brain compression; as Lawton once said, there is no aneurysm more daunting than dolichoectatic basilar trunk aneurysms.[7] Many strategies have been proposed to treat these aneurysms, and many positive outcomes have been reported. Here, we report our single-centre experience in the treatment of dolichoectatic vertebrobasilar aneurysms (Table 1).

Methods

Data collection

Demographic data, including information on patient age, sex, family history, medical history, connective tissue disorders, and surgical or endovascular treatment, were recorded. Clinical symptoms and neurological examination data were collected to assess the modified Rankin Scale (mRS) scores.

ACCEPTED MANUSCRIPT

ACCEPTED MANUSCRIPT

Patient population

Between 2010 and October 2017, 12 patients (9 males and 3 females) with sy mptomatic dolichoectatic vertebrobasilar aneurysms were treated at the Tianjin Huanhu Hospital. Dolichoectatic aneurysms were defined as fusiform, circumfer ential dilations of the vertebrobasilar artery. Patients with aneurysms involving t he basilar bifurcation, posterior cerebral artery (PCA), superior cerebellar artery (SCA), or posterior inferior cerebellar artery (PICA) were excluded. Patients with saccular aneurysms in the vertebrobasilar artery were also excluded.

Among all the aneurysms, 7 involved the basilar artery (BA), 3 involved the vertebral artery (VA), and 2 involved both the BA and VA.

Patients with no symptoms were managed conservatively, whereas patients wi th clinical symptoms were managed surgically. Six patients underwent open sur gery alone, 4 patients underwent endovascular procedures, 1 patient was treated via endovascular procedures in combination with open surgery, and 1 patient was treated with antiplatelet therapy according to the wishes of the patient.

Clinical presentation

No patient had a history of SAH. Four patients presented with symptoms of d izziness. Eight patients presented with mass effect symptoms or thromboemboli c symptoms (transient ischaemic attacks (TIAs) and/or infarction), such as dysa rthria, cranial nerve deficits, dysphagia, and ataxia.

Preparation

Indications for the treatment of patients with symptomatic dolichoectatic vertebr

ACCEPTED MANUSCRIPT

obasilar aneurysms were discussed in a joint meeting with neurologists, neurosurgeons, interventional neuroradiologists, and neuroradiologists, taking into account collateral blood supply, aneurysm location, surgical accessibility, and patient preferences.

Three-dimensional digital subtraction angiography (DSA) was preoperatively performed on all patients, which provided a good understanding of the aneurysm conformation and its relation to large brainstem perforators, as well as the origin of the branches. In combination with cerebral angiography, we used computed tomography angiography (CTA) and magnetic resonance imaging (MRI) to understand the relationship between the aneurysm and the skull base and to define the true size of the aneurysm.

Open surgery

If a bypass procedure was planned, the patients were put on aspirin (100 mg/day) for one week before the operation, and the dosage was continued postoperatively. Revascularization was performed based on the results of balloon occlusion test, cali-bre of the posterior communicating arteries (PCoAs) and superficial temporal artery (STA), presence of a complete circle of Willis, and perfusion indicated on MRI. If the patients were selected for permanent revascularization with a radial artery (RA) graft, the Allen test was performed to confirm the patency of the palmar arch and collateral vessels to the hand from the ulnar artery.

Because the balloon may occlude brainstem perforators, the balloon occlusion test is not necessarily indicative of failure of Hunterian ligation. According to Steinberg et

ACCEPTED MANUSCRIPT

al., the calibre of bilateral PCoAs is the key factor in the avoidance of postoperative brain ischaemia. When both PCoAs are more than 1 mm in diameter, VA occlusion may be safe. When one PCoA or bilateral PCoAs are less than 1 mm in diameter, a bypass procedure must be considered. However, it remains unclear as to which type of bypass is necessary, namely, a high-flow bypass or a relatively low-flow bypass. Bypass procedures were performed through an orbitozygomatic approach or a far lateral approach.

Intraoperative monitoring and management and revascularization techniques hav e been described in detail in the literature.[24,25,26] Bypass patency was evaluated with intraoperative indocyanine green angiography and postoperative digital an giography or CTA.

For VA narrowing, the patients were positioned in a supine position with a r oll under the ipsilateral shoulder, and their head was slightly hyperextended an- d minimally turned to the opposite side. The skin incision for exposing the V2 segment followed the medial border of the middle portion of the sternocleido mastoid muscle. Then, the platysma muscle was longitudinally opened, and diss ection was performed between the medial border of the sternocleidomastoid m uscle and the lateral border of the internal jugular vein. The fat layer around these structures was laterally retracted. The prevertebral aponeurosis was expos- ed, and the sympathetic chain below it was identified. The aponeurosis was cut along the sympathetic chain and laterally retracted to preserve the connections with the cervical nerve roots. The longus colli muscle, which was under the prevertebral aponeurosis, was cut along the lateral aspect of the vertebral bodie s and transverse processes. Subsequently, the periosteum of the transverse proce sses was elevated with a smooth spatula. The periosteum inside the transverse foramen was then detached from the bone. In the space between bone and peri

ACCEPTED MANUSCRIPT

osteum, the foramen was opened with the drill. The V2 segment was then exp osed between two transverse foramina. The next step was to partly occlude the VA with an aneurysm clip. Postoperative angiography was used to determine stenosis of the left VA.[29]

Of the patients treated surgically, 2 patients (case 5 and case 7) underwent ST A-PCA bypass alone, 1 patient (case 9) underwent left STA-PCA bypass and li gation of the bilateral VA by endovascular techniques, 1 patient (case 2) under went VA-RA-PCA bypass in conjunction with occipital artery (OA)-PICA bypas s and thrombectomy of the aneurysm, 1 patient (case 8) underwent left VA-RA -PCA and left OA-PICA bypass followed by ligation of the left VA, 1 patient (case 2) underwent VA-RA-PCA bypass in conjunction with OA-PICA bypass a nd thrombectomy of the aneurysm, and 1 patient (case 6) was treated with left STA-PCA bypass in combination with VA narrowing (V2 segment) with an an eurysm clip. One patient (case 3) was first treated endovascularly, but she pres ented with deteriorated neurological conditions; thus, 5 years later, she underwe nt right internal maxillary artery (IMA)-RA-PCA bypass and ligation of the left VA by endovascular techniques.

Endovascular treatment

Patients were selected for endovascular treatment based on the following criteri a: 1) patients without considerable thrombus in the aneurysmal lumen; 2) patie nts without severe or progressive thromboembolic symptoms; and 3) patients wi thout SAH.

Prior to the endovascular procedures, a dual antiplatelet therapy with aspirin (1

00 mg) and clopidogrel (75 mg) was started for 7 days. All procedures were performed via the percutaneous transfemoral route under general anaesthesia. After the placement of a 6F sheath introducer, heparin was intravenously administered to keep the activated clotting time (ACT)>2.5 times the control ACT. With the guidance and assistance of a microcatheter, a self-expanding stent (Solitaire, ev3) was advanced over the prepositioned exchangeable guidewire and was deployed to cover the entire lesioned segment. The number and arrangement of the stent was determined according to the location, size and shape of the aneurysm. To further decrease blood flow in the aneurysms in an attempt to promote luminal thrombosis, in 2 of 12 patients, we finally occluded 1 (case 3) or 2 (case 9) VAs with coils in the V4 segment just proximal to the vertebral junction. One of the patients was treated using endovascular procedures (case 11); at first we failed to deploy the stent because of the tortuosity of the left VA. After performing an open surgery to straighten the left VA, we successfully deployed the stent to cover the lesioned segment.

Follow-up

Follow-up vascular imaging was performed using DSA and CTA. Pre- and postoperative neurological functions were evaluated with the mRS scale, and “deterioration” was defined as ≥1 point decrease on the scale.

Results

Our study included 3 women and 9 men with a mean age of 57.3 years (range, 48-68 years; median 56 years). The aneurysms predominantly involved the B

A in 7 cases, and the VA in 3 cases, both BA and VA in 2 cases. All aneurysms remained unruptured before treatment. The most common symptom was dizziness (6 cases, 41.7%).

Among the entire cohort, 4 patients were treated endovascularly, 1 patient was treated with medicine, and 7 patients were treated surgically: 2 patients underwent STA-PCA bypass alone, 1 patient underwent STA-PCA bypass and ligation of the bilateral VA, 1 patient underwent left IMA-RA-PCA bypass and ligation of the left VA, 1 patient underwent LV3-RA-VA bypass in conjunction with OA-PICA bypass and thrombectomy of the aneurysm, and 1 patient underwent left STA-PCA bypass in combination with VA narrowing with an aneurysm clip. Postoperatively, the bypass was patent in all patients.

The most common complication was pneumonia, which affected patients who were treated with open surgery alone, owing to the dysfunction of the lower cranial nerves, prolonged mechanical ventilation, and long duration of hospitalisation.

Other complications associated with initial treatment included 1 case of hydrocephalus, 1 case of haematoma and 1 case of infarction due to perforating artery thrombosis.

Two patients died after open surgery. One patient (case 3) was first treated endovascularly, but she displayed deterioration of her neurological condition. Five years later, she underwent left IMA-RA-PCA bypass and ligation of the left VA. Despite a well-functioning RA bypass graft, this patient died due to respirat

ACCEPTED MANUSCRIPT

ory failure, probably from perforator vessel occlusion that occurred when the a neurysm thrombosed. Case 7 developed haematoma after right STA-PCA bypass, requiring the removal of haematoma and decompression of the bone flap. Ho wever, the patient never woke up, and finally, his relatives withdrew care. Ther e were no progressive symptoms for patients treated endovascularly.

For the entire cohort, the follow-up period was 5-67 months. The mean mRS score was 1.8 (range, 1-5; median, 1) before treatment and 2.8 (range, 1-6; me dian, 2) after treatment. Overall, two patients died (mRS score 6, 16.7%), seve n patients had good recoveries (mRS score 0-2, 58.3%), 3 patients had modera te-severe disabilities (mRS score 3-5, 25%), 5 patients were neurologically wor se after treatment, and 7 patients presented steady neurological conditions. Thes e outcomes indicate that treatment is associated with a high risk of morbidity and mortality.

Illustrative Cases

Case 6

A 63-year-old man, with a history of smoking and hypertension, presented at o ur department with paralysis of the right eyelid and right hemifacial spasm. On CT and MRI, a large and partially thrombosed basilar trunk aneurysm was ap parent. CTA and angiography confirmed an extremely large fusiform dolichoect atic proximal BA (Figure 1). The patient first underwent left STA-PCA bypass to reverse blood flow through the aneurysm and augment flow to the basilar apex. One month later, we partly occluded the left VA (>70% stenosis) to redu

ce blood flow through the aneurysm. Postoperative imaging confirmed the patency of the bypass. After the operation, the patient displayed steady neurological conditions. A follow-up angiogram at 6 months showed that the aneurysm was smaller in size (Figure 2,3).

Case 9

A 50-year-old man, who had a history of smoking, presented with dizziness, dysarthria, weakness of the right limbs. CT, MRI, CTA and cerebral angiography revealed an extremely large, partially thrombosed, dolichoectatic basilar aneurysm associated with significant compression of the brainstem(Figure 4). The patient underwent left STA-PCA bypass with planned delayed (25 days later) endovascular occlusion of bilateral VAs using a combination of coils and stents. Immediate postoperative angiograms revealed that the bypass was patent and that the aneurysm disappeared. However, after the endovascular procedures, the condition of the patient was worse than his preoperative status, but the patient's neurological conditions gradually improved, and he was discharged one month later. At his 1-year follow-up examination, the bypass was patent, and the bilateral VA was completely occluded. The patient could stand up with help from his family(Figure 5).

Case 1

A 61-year-old man presented at our department with dizziness. On CT, MRI and CTA, a large aneurysm was apparent. Digital angiogram confirmed that the aneurysm extended from the V4 segment of the right VA to the trunk of the BA. Under local

anaesthesia, a unilateral femoral 6F sheath was placed through the percutaneous femoral artery using the Seldinger technique, and one 6F ENVOY guiding catheter (Cordis) was then inserted into the right VA. A Rebar-27 catheter (ev3) was conducted through a 0.014-inch microguide wire (Transcend, Target) across the lesion site under road mapping and ultimately positioned at the distal BA. A Solitaire stent (6.0×30 mm) was advanced through the Rebar-27 catheter and inserted into the inner arterial wall. The second 6.0×20 mm Solitaire stent was implanted sequentially overlapping the first stent. Angiography after the procedure revealed good stent placement and patency. After the operation, the patient experienced no complications and was discharged one week later. A follow-up angiogram at 10 months showed that the aneurysm was unchanged in size, with no shifting of the deployed stents. During follow-up, the patient continued to experience dizziness with an unchanged mRS score(Figure 6).

Discussion

Dolichoectatic vertebrobasilar aneurysms are uncommon aneurysms characterized by pathologically enlarged, elongated, and abnormally tortuous VA and BA. In a review by Yu et al.,[14] only 17 patients with dolichoectatic vertebrobasilar an eurysms were identified from a large series of 50,000 cerebral angiograms (0.0 34%). In the report by Pia, 4 dolichoectatic aneurysms were identified in 538 patients (0.7%).[15] Between 2016 and October 2017, 12 dolichoectatic vertebroba silar aneurysms were identified in 2659 patients, and the incidence was 4.5%. Based on the literature, the incidence of dolichoectatic vertebrobasilar aneurysm

ACCEPTED MANUSCRIPT

is as low as 0.06-5.8% in the population.[14,15]

The pathogenesis of this type of aneurysm is not clear. There are several hypotheses: (1) Atherosclerosis: Dandy[17] regarded the atherosclerotic degeneration of the vascular wall as the initial factor of these aneurysms, and a few reports have supported his findings. However, in contrast to the prevalence of atherosclerotic vascular disease, dolichoectatic vertebrobasilar aneurysms are rare. These aneurysms have also been identified in patients without atherosclerosis and in children. (2) Hemodynamic factors: In a trial[18], after the ligation of one or two carotid arteries in rabbits, the BA was found to expand in response to the increased blood flow and shear stress, indicating an association between the variations in the circle of Willis and dolichoectatic vertebrobasilar aneurysms. Additionally, several other factors, such as smoking, male sex, hypertension, syphilis, septic or mycotic emboli, trauma or congenital defects in the vascular wall, can trigger the dolichoectatic process.[5,23]

The clinical symptoms caused by the dolichoectatic aneurysms can be grouped into SAH, ischaemia, or compression of a cranial nerve, the brainstem, or the third ventricle with hydrocephalus.

Cerebral ischaemia, including brain infarction and TIA, may be the most common symptoms. Possible mechanisms include the blocking of perforating arteries originating from the dolichoectatic aneurysms, in situ thrombosis, and emboli from the dolichoectatic artery.[7]

Compared to that with saccular aneurysms, rupture is rare with dolichoectatic

ACCEPTED MANUSCRIPT

ACCEPTED MANUSCRIPT

vertebrobasilar aneurysms. No SAH occurred in our cohort. According to other studies, approximately 7% of patients with dolichoectatic vertebrobasilar aneurysms can experience SAH, and the annual rupture rate is 0.9%; additionally, the risk of rupture may be related to the diameter of the aneurysm; aneurysms more than 10 mm in diameter may have a higher bleeding risk.[5,20,22] Idbaih et al.[16] asserted that the risk of rupture-associated bleeding is related not only to the degree but also to the speed of expansion of the arteries, which may be a sign of SAH in the short term.

Dysarthria, dysphagia, dystaxia, hemifacial spasm, and trigeminal neuralgia are the most common symptoms of dolichoectatic vertebrobasilar aneurysms. In our cohort, 9 patients had symptoms indicative of compression of a nerve by dolichoectatic vertebrobasilar aneurysms. Some patients can also present with hydrocephalus due to the obstruction of the third ventricle or the foramen of Monro.

Treatment Options

Currently, for patients with dolichoectatic vertebrobasilar aneurysms, the application of medication is still controversial. Some studies conclude that the use of anticoagulant or antiplatelet therapy may increase the risk of rupture of dolichoectatic vertebrobasilar aneurysms, especially for aneurysms>10 mm in diameter. However, for patients with progressive thrombosis or distal embolism, anticoagulation or antiplatelet therapy may be effective.[4,9] One patient (case 4) in our cohort underwent clopidogrel (75 mg) antiplatelet, moderate-intensity stati

ACCEPTED MANUSCRIPT

n, and antihypertensive therapy. After one year of follow-up, the patients pres ented steady neurological condition.

Currently, covered stents, flow diversion, stent-assisted coil embolization, and st-ent-only therapy with single or multiple stents comprise the main techniques for treating dolichoectatic vertebrobasilar aneurysms.[6,16] In particular, high density and low porosity flow diversion to modify blood flow are increasingly being used to treat dolichoectatic vertebrobasilar aneurysms. However, in a study including 7 patients, after the deployment of the flow diversion, 4 patients died, and 1 patient suffered from severe disabilities.[1] Therefore, data for long-term efficacy are lacking. Stent-assisted coil embolization in the perforator-rich vertebrobasilar system also contributes to thrombotic occlusion of perforating vessels and subsequent ischaemic complications and ultimately perforator stroke.[30]

The stent-within-a-stent technique was the main technique used in our cohort, a nd this technique can reduce inflow to the aneurysm and induce thrombosis.[28] I n the present cohort of patients, we used Solitaire stents, which are safe and e ffective devices for intravascular interventions and are fully recoverable and eas y to place in the intended location. However, this treatment technique may not be an ideal option for aneurysms originating in the PICA because of the poss ibility of occluding the PICA with the stent as well as occluding the perforatin g arteries. Nevertheless, 4 patients in our cohort were treated with the stent-wit hin-a-stent technique, and their neurological function did not deteriorate further, but after deployment of the stent, 1 patient (case 3) presented repeated new b

rain infarction, and thus, she was switched to open surgery.

Unlike saccular aneurysms, dolichoectatic vertebrobasilar aneurysms usually incorporate 4 branches (bilateral PCAs and SCAs) and many vital perforating arteries, which are the most challenging to treat. At first, Hunterian ligation (obliteration of the BA proximal to the SCA or occlusion of bilateral VAs) advocated by Drake[11,19] was used as a salvage treatment for these lesions, but basilar occlusion is associated with devastating stroke in patients with poor collateral circulation. Therefore, reducing or delaying the enlargement of dolichoectatic vertebrobasilar aneurysms and providing flow to distal vasculature, especially the brainstem perforators, are the two challenges faced during the treatment of these lesions[3,8]. Reversing and reducing blood flow through dolichoectatic vertebrobasilar aneurysms in combination with augmenting blood flow to the upper BA by bypass are the main surgical strategies at present.

The senior author has attempted three methods of blood flow alteration to treat these aneurysms: extracranial-intracranial (EC-IC) bypass alone, EC-IC bypass followed by Hunterian ligation of the ipsilateral VA or bilateral VAs and EC-IC bypass in combination with VA narrowing with an aneurysm clip.

According to our experience in the treatment of anterior circulation aneurysms, EC-IC bypass is an effective surgical method for treating complex aneurysms. However, there have been reports in the literature of large aneurysm rupture following bypass[27] when the anterograde flow is strong, thus reducing the blood flow in the bypass graft and inducing thrombosis.

ACCEPTED MANUSCRIPT

Bypass in conjunction with Hunterian ligation of one or both VAs is a classic flow-diverting option chosen by most surgeons. This strategy consists of an EC-IC bypass, usually connecting the STA to the PCA, followed by Hunterian ligation of the proximal VA. The logic behind this strategy is simple: reducing flow to the aneurysm promotes thrombosis while the bypass revascularizes the territory beyond the aneurysm. However, despite initial treatment success, many patients return with disease progression and need further treatment followed by grim outcomes.[3] In addition, in practice, it is difficult to maintain the balance between perforator preservation and aneurysm thrombosis.

Compared with other treatments of reducing blood flow, artificial VA narrowing (V2 segment) is our new strategy to treat these aneurysms, which can induce spontaneous thrombosis of aneurysms and facilitate parent artery reconstruction through flow remodelling, similar to flow-diverting stents. Moreover, this procedure is easy to perform, can shorten hospitalization time and reduce sequelae and complications. However, we do not know the extent to which VA stenosis is the most effective. Despite these limitations, this treatment strategy still offers us with a new option. Furthermore, to quickly avoid aneurysm thrombosis threatening perforator patency, staging surgery may be reasonable.

Limitations

Our study has several limitations. First, this is not a randomised retrospective study. Second, the number of patients in this cohort was small. Third, the follow-up period for the patients was relatively short. Therefore, we cannot draw

ACCEPTED MANUSCRIPT

ACCEPTED MANUSCRIPT

definitive conclusions.

Conclusion

The ideal treatment of dolichoectatic vertebrobasilar aneurysms remains controversial. In our initial experience, patients with mild or stable neurological symptoms can undergo anticoagulant or antiplatelet therapy or endovascular procedures. Given the serious complications that can be caused by open surgery, care must be taken when selecting the patients to be treated using open surgery. We agree that the goal should always be a perfect patient rather than a perfect angiogram. Reversing and reducing blood flow through the aneurysm are effective in promoting thrombosis of the aneurysm. VA narrowing is a new strategy for revascularization, but further study is necessary to confirm its clinical efficacy and safety.

References

1. Sidddiquib AH,Abla AA,et al. Panacea or prolem:flow diverters in the treatment of symptomatic large or giant fusiform vertebrobasilar aneurisms.J Neurosurg.2012;116:1258-66.

2. Fernando Pico,Jullenr Labreuche AL.Pathophysiology,presentation,prognosis,and management of intracranial arterial dolichoectasia.Lancet Neurol.2015;14:833-45

3. Kalani MY,Zabramski JM,et al.Bypass and Flow Reduction for Complex Basialr and Vertebrobasialr Junction Aneurysms.Neurosurgery.2013;72:763-776.

4. Kobayashi N,.Murayama Y,et al.Natural Course of Dissecting Vertebrobasilar Artery Aneurysms without stroke.AJNR Am J Neuroradiol.2014;35:1371-75.

5. Xu.D.S,Levit.M.R,et al.Dolichectatic aneurysms of the vetebrobasilar system: clinical and radiographic factors that predict poor outcomes.J Neurosurg.2017;A-pril7:1-7.

6. Dabus G,Lin E,et al.Endovascular treatment of fusiform intracranial vertebra-l artery aneurysms using reconstructive techniques.J Neurointervent Surg,2014;6: 589-94.

7. Lawton MT,Abla AA,et al.Bypass Surgery for the Treatment of Dolichoecati-c Basilar Trunk Aneurysms:A Work in Progress.Neurosurgery.2016;79:83-99.

8. Kalani MYS,Zabramski JM.et al.Twenty-Year Follow-up of Flow Reversal a-nd Revascularization for a Giant Sepentine Basilar Artery Aneurysm.Neurosurg-ery.2014;10:E493-E497.

9. Anson JA,Lawton MT,et al.Characteristics and surgical treatment of doichoe-ctatic and fusiform aneurysms.J Neurosurgery.1996;84:185-93.

10. Drake CG,Peerless SJ.Giant fusiform intracranial aneurysms:review of 120 patients treated surgically from 1965 to 1992.J Neurosurgery.1997; 87(2):141-62.

11. Steinberg GK,Drake CG,Peerless SJ.Deliberate basilar or vertebral artery oc-clusion in themtreatment of intracranial aneurysms.Immediate results and long-t-erm outcome in 201 patients.J Neurosurg.1993;79(2):161-73.

12. Flemming KD,Wiebers DO,Brown RD,et al.Prospective risk of hemorrhage in patients with vertebrobasilar nonsaccularnintracranial aneurysm.J Neurosurg.20 04; 101:82-87.

13. Idbaih A, Pico F, Guichard JP,et al.Clinical Course and MRI Changes of Bas

ilar Artery Dolichoectasia:Three Case Reports.Cerebrovasc Dis.2004;17:262-64.

14. Yu YL, Moseley IF, Pullicino P,et al.The clinical picture of etcasia of the i-ntracerebral arteries.Neurosurg Psychiatry.1982;45:29-36.

15. Pia HW.Classification of vertebro-basilar aneurysms.Acta Neurochir.1979;47:3-30.

16. Jun Wang,Xin-feng Liu,et al.Application of parallel stent placement in the treatment of unruptured vertebrobasilar fusiform aneurysms.J Neurosurgery.2017;126:45-51.

17. Dandy We.Intracranial Arterial Aneurysms.NewYork,Hafner,1944

18. Hoi Y,Gao l,Treatment M,et al.In vivo assessment of rapid cerebrovascular morphological adaptation following acute biood flow increase.J Neurosurgery.2008;109:1141-47.

19. Drake CG.Ligation of the vertebral(unilateral or bilateral)or basilar artery in the treatment of large intracranial aneurysms.J Neurosurgery.1975;34:589-95.

20. Passero SG,Rossi S.Natural history of vertebrobasilar doichoectasia.Neurology.2008;70:66-72.

21. Wolters FJ,Rinkel GJ,Vergouwen MD.Clinical course and treatment of vertebraobasilar dolichoectasia:a systematic review of the literature.Neuro Res.2013;35:131-132.

22. Flemming XD,Wiebers DO,Brown RD,et alThe natural history of radiographically defined vertebraobasilar nonsaccular intracranial aneurysms.Cerebrovasc Dis.2005;20:270-79.

ACCEPTED MANUSCRIPT

23. David S,Michael R,Levit,et al.Dolichoectatic aneurysms of the vertebrobasial r system:clinical and radiographic factors that predict poor outcomes.J Neurosu-rgery.2017;April7:1-7.

24.Mai JC,Tariq F,Kim LJ,et al.Flow diversion radial artery bypass graft coupl-ed with terminal basilar artery occlusion for the treatment of complex basilar apex aneurysms:operative nuances.Neurosurgery.2013;72(2):116-126.

25.Sekar LN,Duff JM,Kalavakonda C,et al.Crebral revascularization using radial artery grafts for the treatment of complex intracranial aneurysms:techniques a-nd outcomes for 17 patients. Neurosurgery.2001;49(3):649-659.

26.Cantore G,Santoro A,Guidetti G,et al.Surgical treatment of giant intracranial aneurysms:current viewpoint. Neurosurgery.2008;63(2):279-290.

27.Scott RM,Liu HC,Yuan R,et al.Rupture of a previously unrupture giant midd le cerebral artery aneurysm after extracranial-intracranial bypass surgery. Neuros urgery.1982;10(5):600-603.

28.Mehta B,Burke T,Kole M,et al.Stent-with-a-stent technique for the treatment of dissecting vertebral artery aneurysms.AJNR Am J Neuroradiol.2003;10(24):18 14-1818.

29.Meybodi AT,Lawton MT,Mokhtari P,et al.Exposure of the external carotid art ery through the posterior triangle of the neck:a novel approach to facilitate byp ass procedures to the posterior cerebral circulation. Operative Neurosurgery.2017; 13(3):374-381.

30.Wakhloo AK,Mandel J,Gounis MJ,et al.Stent-assisted reconstructive endovasc

ACCEPTED MANUSCRIPT

ACCEPTED MANUSCRIPT

ular repair of cranial fusiform atherosclerotic and dissecting aneurysms:long-term clinical and angiographic follow-up. Stroke.2008;39:3288-96.

Acknowledgements

We would like to thank all the patients who trusted us and all the physicians and staff who helped us in this study.

Disclosure: The authors report no conflict of interest concerning the materials or methods used in this study or the findings specified in this paper.

Funding: This study did not receive any specific grant from funding agencies in public, commercial, or not-for-profit sectors.

Figure Legends**:**

Figure 1. Preoperative evaluation of case 6: A 63-year-old man with paralysis of the right eyelid and right hemifacial spasm (case 6). CT (A), MRI (B and C) and CTA (D) demonstrated a basilar trunk dolichoectatic aneurysm. Lateral (E) and anteroposterior (F) angiogram revealed the morphology of the aneurys-m.

Figure 2. Follow-up of case 6: Adequate collateral flow through PCoAs did not exist for this case (A). Therefore, the patient first underwent left STA-PCA bypass (B). One month later, he was treated via left VA narrowing (C). CT angiogram and digital angiogram showed stenosis of the left VA after treatment (D and E). A 6-month follow-up digital angiogram demonstrated the dolichoecta

tic aneurysm was smaller (F).

Figure 3. Conceptual scheme of treatment strategies for case 6.

Figure 4. Preoperative evaluation of case 9: A 50-year-old man presented with dizziness, dysarthria, weakness of the right limbs (case 9). CT (A) and axial (B) and sagittal (C) MRI demonstrating an extremely large dolichoectatic basilar aneurysm. CT angiogram (D) and lateral view of left and right vertebral angiogram (E and F) revealed the morphology of the aneurysm.

Figure 5. Follow-up of case 9: The patient was first treated with left STA-PCA bypass (A). Twenty-five days later, bilateral VAs were occluded using a combination of coils and stents (B). Digital angiogram demonstrated that the bypass supplied the apex of the BA after treatment (C). At one year of follow-up, lateral right VA (D) and left VA (E) angiograms revealed occlusion of the aneurysm. Oblique angiogram (F) demonstrated patency of the left STA-PCA bypass and partial development of the aneurysm.

Figure 6. A 61-year-old man with dolichoectatic vertebrobasilar aneurysm presented with dizziness. CT (A), MRI (B) and CTA (C) revealed a large fusiform aneurysm in the vertebrobasilar arterial trunk. A preoperative lateral digital angiogram showed that the right VA was the dominant side, and the aneurysm extended from the V4 segment of the right VA to the trunk of the BA (D). Two Solitaire stents (6.0×30 mm and 6×20 mm) were deployed into the inner arterial wall by the stent-within-a-stent technique. An unsubtracted digital subtraction angiogram of the aneurysm acquired after deployment of stents is shown (E).

ACCEPTED MANUSCRIPT

ACCEPTED MANUSCRIPT

Anteroposterior view of the angiogram acquired 10 months after deployment o f stents (F).

ACCEPTED MANUSCRIPT

Table 1 Clinical data of patients with dolichoectatic vertebrobasilar aneurysms

**Case**	**Age**	**Sex**	**Location**	**History**	**Presentation**	**Treatment**	**Bypass Type**	**Bypass patency**	**Complications**	Preop **mRs**	Postop **mRs**	**Follow-up (months)**
**1**	61	Male	RVA,BA	-	Dizziness	Stents	-	-	-	1	1	22
**2**	48	Male	LVA	Hypertension	Dizziness<br>Dysarthria<br>Dyphagia<br>Weakness	Bypass<br>Aneurysm excision	LV3-RA-VA<br>LOA-PICA	YES	Pneumonia	5	5	12
**3**	52	Female	BA	Hypertension	Weakness<br>Delay speech	Stents<br>Bypass<br>Occlusion of LVA	RIMA-RA-PCA	YES	Pneumonia<br>hydrocephalus	2	6	67
**4**	59	Famale	BA	Hypertentio<br>CHD	Lost conscious	Medical therapy	-	YES	-	4	4	7
**5**	54	Male	LVA,BA	Hypertention<br>Smoking<br>Drinking	Headaches<br>Gait instability	Bypass	LSTA-PCA	YES	Pneumonia<br>Brain ,<br>infarction	1	2	8
**6**	63	Male	BA	Hypertention<br>Smoking	Hemifacial<br>Spasm	Bypass<br>Narrowing VA	LSTA-PCA	YES	-	1	1	11

Table 1 Continued

Case	Age	Sex	Location	History	Presentation	Treatment	Bypass Type	Bypass patency	Complications	Preop mRs	Postop mRs	Follow-up (months)
**7**	56	Male	BA	Hypertention,HLP	Dysarthria,ataxia	Bypass	RSTA-PCA	YES	Hematoma,pneumonia	2	6	12
**8**	53	Male	LVA	Smoking	Headach<br>Syncope<br>Facial paralysis<br>Dyphagia	Bypass<br>Occlusion of LVA	LVA-RA-PCA<br>LOA-PICA	YES	Pneumonia	2	2	5
**9**	50	Male	BA	Smoking<br>Drinking	Dizziness<br>Weakness	Bypass<br>Occlusion of bilateral VA	LSTA-PCA	YES	Pneumonia	1	4	20
**10**	68	Male	BA	NO	Dizziness	Stents	-	YES	-	1	1	15
**11**	56	Male	BA	Hypertention<br>Brain Infarction	Delay Speech	Stents<br>Releasing the tortuosity of VA	-	-	-	1	1	18
**12**	68	Female	BA	Hypertention,CHD	Dizziness	Stents		-	-	1	1	24

ACCEP

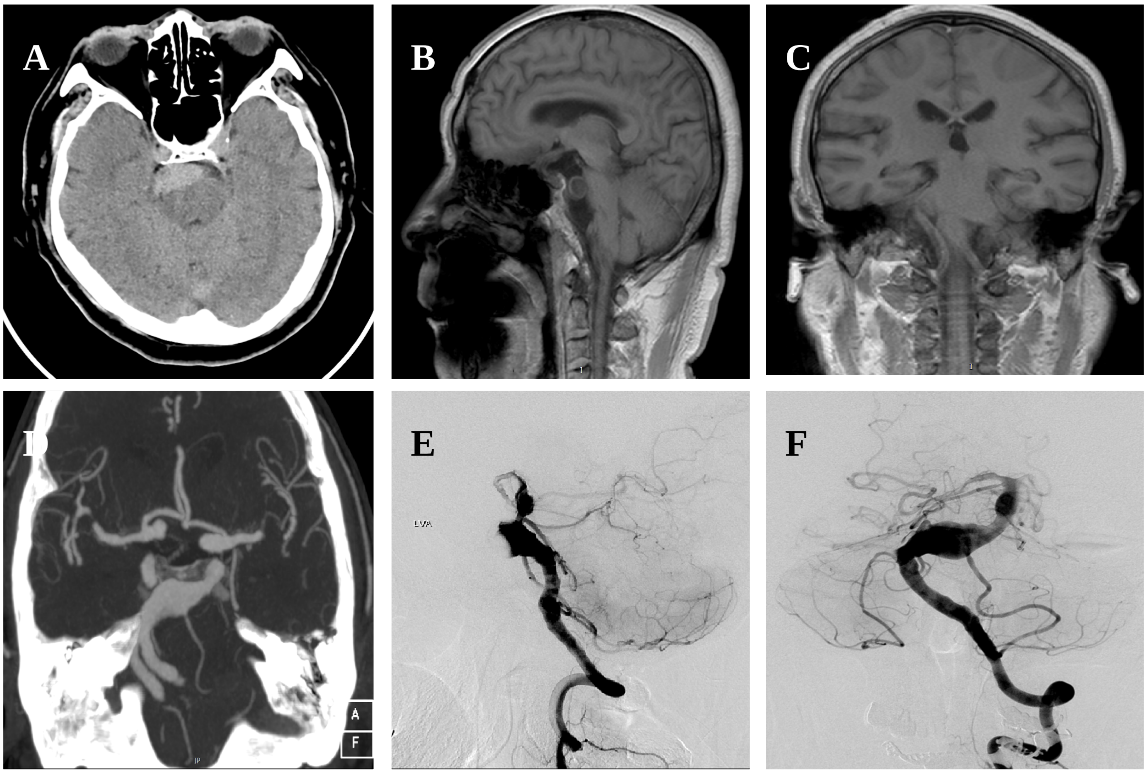

ACCEPTED MANUSCRIPT

ACCEPTED MANUSCRIPT

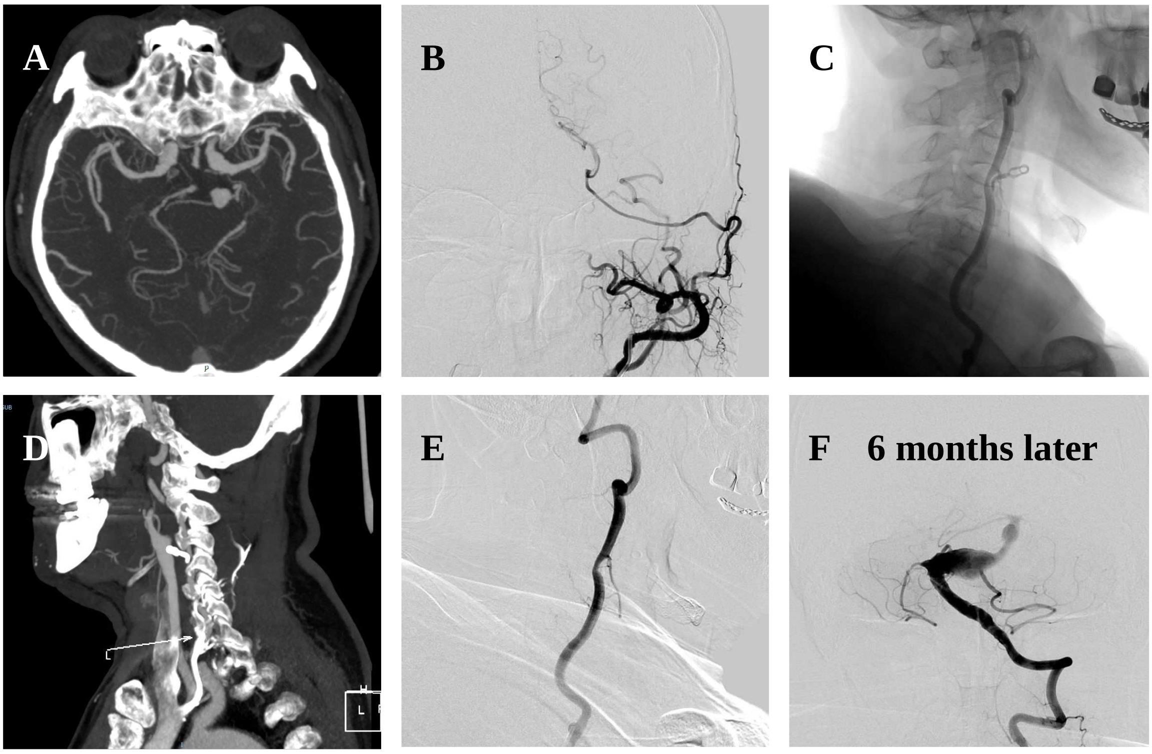

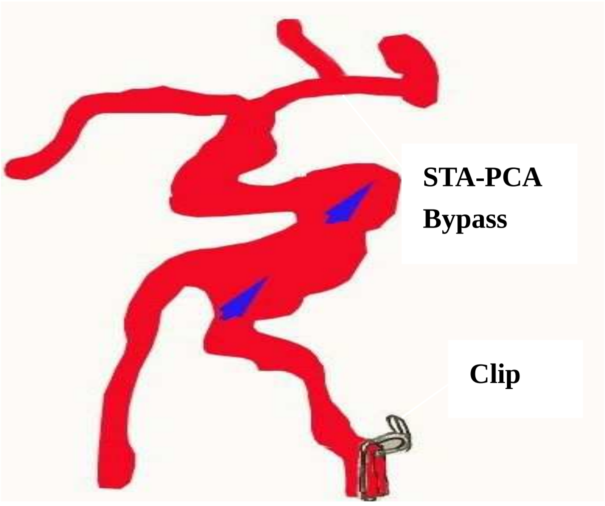

ACCEPTED MANUSCRIPT

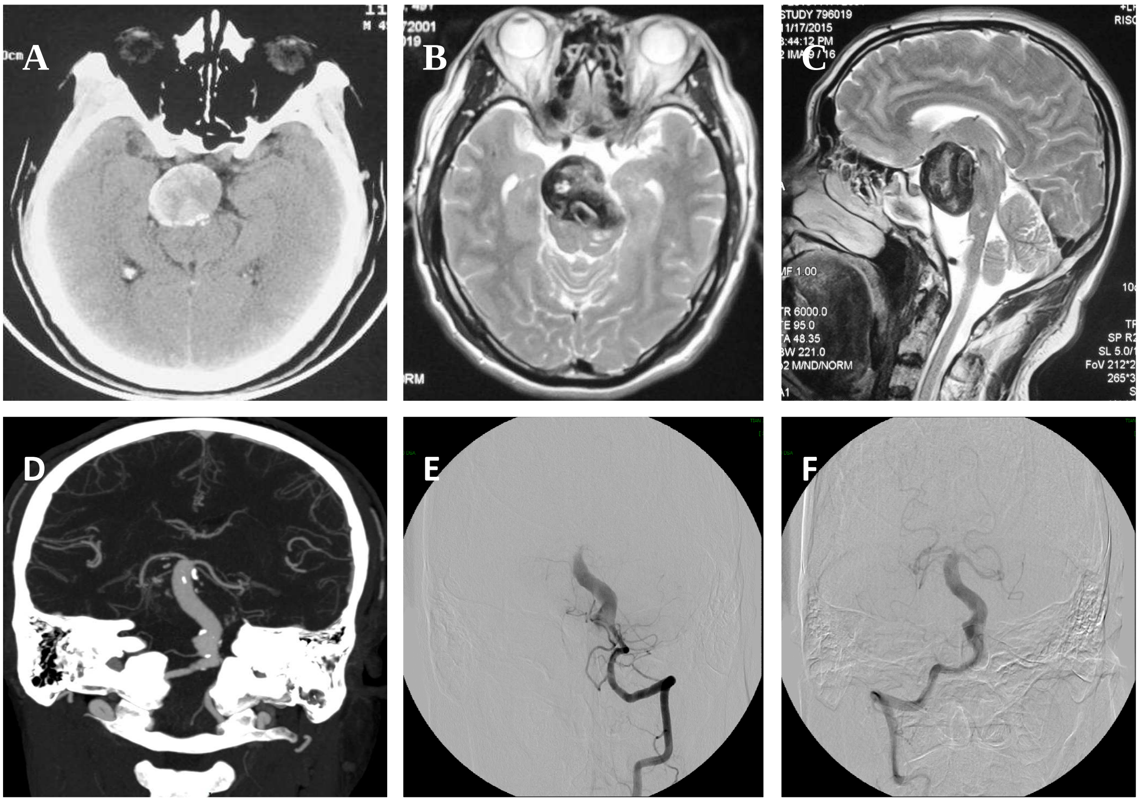

ACCEPTED MANUSCRIPT

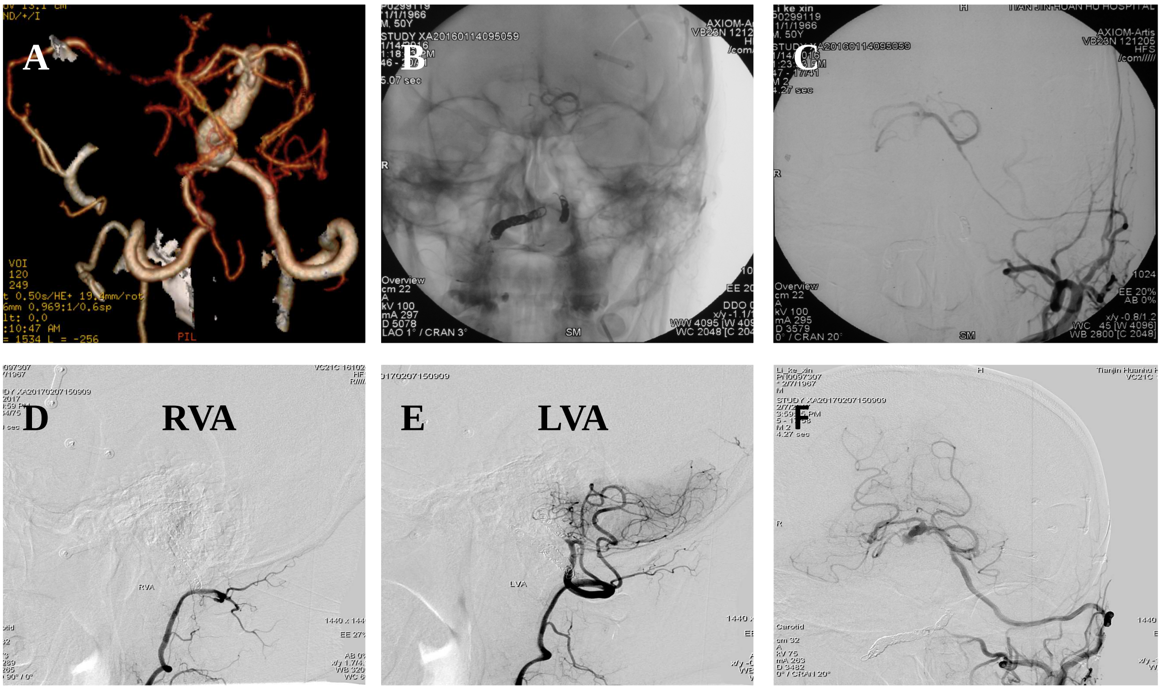

ACCEPTED MANUSCRIPT

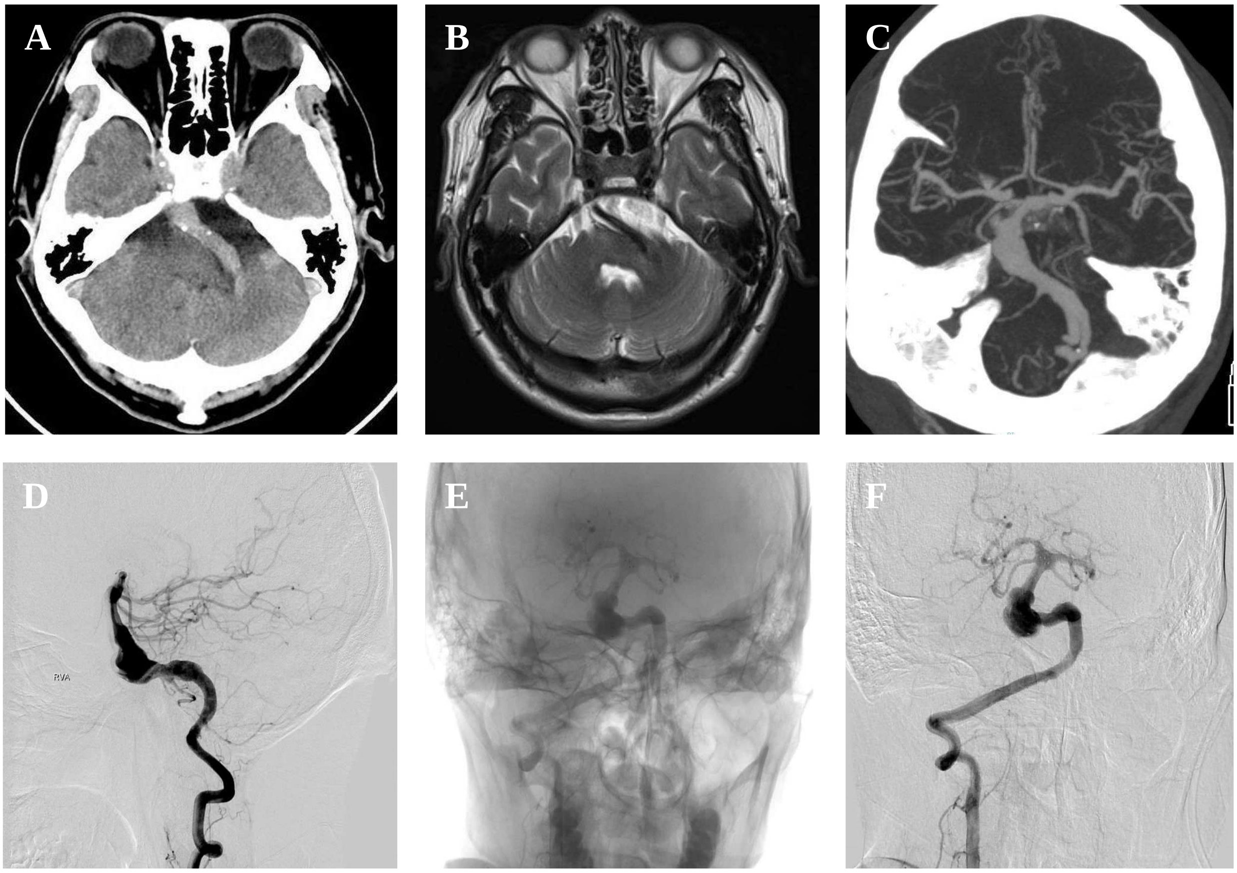

ACCEPTED

Highlights

1. Dolichoectatic vertebrobasilar aneurysms are uncommon cerebral aneurysms, which pose a significant management challenge for both neurosurgeons and interventionalists.
2. The pathogenesis of these difficult aneurysms is still not clear.T-he cause may be a primary dysplasia of the muscularis with se-condary changes in the elastic tissue duo to arterial distention,or, a congenital deficiency of the elastic tissue when subject to oth-er risk factors.
3. The most common clinical symptomms caused such aneurysms can be grouped: SAH, ischemia;compression of a cranial nerve, the brainstem, or the third ventricle with hydrocephalus.
4. For different patients, they can be treated by medication, open s urgery,or,endovascular procedures. Narrowing the parent vertebral artery(V2 segment),which is easier performed, is our new choice to treat such aneurysms.

ACCEPTED MANUSCRIPT

ACCEPTED MANUSCRIPT

Abbreviation List:

BA=basilar artery; DSA=digital subtraction angiography;

mRS=modified Rankin Scale; PCA=posterior cerebral artery;

VA=vertebral artery; STA=superficial temporal artery;

IMA=internal maxillary artery; OA=occipital artery;

RA=radial artery; PICA=posterior inferior cerebellar artery;

PCoA=posterior communicating artery; SCA=superior cerebellar artery;

CHD=coronary heart disease; HLP=hyperlipidaemia;

SAH=subarachnoid haemorrhage; CTA=computed tomography angiography;

ACT=activated clotting time

ACCEPTED MANUSCRIPT
