# Supplementary material for: Management strategies and outcomes of basilar trunk aneurysms: a systematic review and meta-analysis
Source: Langenbecks Arch Surg. 2026 Jan 20;411(1):71. doi: 10.1007/s00423-025-03959-3 (PMC12852281; doi:10.1007/s00423-025-03959-3)
Supplement: Supplementary file 1 — Supplementary file1 (ZIP 55327 KB) [file 423_2025_3959_MOESM1_ESM.zip › literature.zip!/meta/Zhong 2023.pdf]

OPEN ACCESS

Original research

Reconstructive endovascular treatment for basilar artery trunk aneurysms: complications and clinical and angiography outcomes

Weiying Zhong,[1,2,3] Tongfu Zhang,[1,4] Chenran Su,[1] Donglin Zhou,[1] Jianfeng Zhuang,[1] Maogui Li,[1] Yangyang Xu,[1] Ming Liu,[1] Mingxiang Zhang,[2] Yunyan Wang,[1] Donghai Wang,[1] Wandong Su [1]

► Additional supplemental material is published online only. To view, please visit the journal online (http://dx.doi.org/10.1136/jnis-2022-019864).

[1]Department of Neurosurgery, Qilu Hospital of Shandong University and Institute of Brain and Brain-Inspired Science,Shandong University, Jinan, China
[2]Key Laboratory of Cardiovascular Remodeling and Function Research, Chinese Ministry of Education, Chinese Ministry of Health and Chinese Academy of Medical Sciences, Qilu Hospital of Shandong University, Jinan, China
[3]State Key Laboratory of Generic Manufacture Technology of Traditional Chinese Medicine, Lunan Pharmaceutical Group Co. Ltd, Linyi, China
[4]Department of Neurosurgery, Yangxin County People's Hospital, Binzhou, China

Correspondence to
Dr Wandong Su, Qilu Hospital of Shandong University, Jinan, China; suwandong@sina.com

Received 9 November 2022
Accepted 2 January 2023

Check for updates

© Author(s) (or their employer(s)) 2023. Re-use permitted under CC BY-NC. No commercial re-use. See rights and permissions. Published by BMJ.

To cite: Zhong W, Zhang T, Su C, *et al*. *J NeuroIntervent Surg* Epub ahead of print: [*please include* Day Month Year]. doi:10.1136/jnis-2022-019864

ABSTRACT

Background Basilar artery trunk aneurysms (BTAs) are rare intracranial aneurysms. We aim to investigate the procedural complications and clinical and angiographic outcomes of BTAs treated with reconstructive endovascular treatment (EVT).

Methods We retrospectively reviewed the data of 111 patients with BTAs who underwent reconstructive EVT during 2013–2022. The factors associated with procedural complications and clinical and angiographic outcomes were analyzed.

Results The study included 81 men and 30 women (median age 60 years). Overall, 26 (23.4%) cases presented with subarachnoid hemorrhage and 85 (76.6%) presented with unruptured aneurysms. Periprocedural ischemic and hemorrhagic complications occurred in 29 (26.1%) and 4 (3.6%) cases, respectively. The rate of favorable clinical outcomes was 83.8% (92/111) and the mortality rate was 14.4% (16/111). Angiographic follow-up data were available for 77/95 (81.1%) survivors; 57 (74.0%) and 20 (26%) aneurysms exhibited complete and incomplete obliteration, respectively. Old age, high Hunt and Hess grades (IV–V), hemorrhagic complications, and increased aneurysm size were independent risk factors for unfavorable clinical outcomes (p<0.05). Increased aneurysm size and incomplete aneurysm occlusion on immediate angiography were independent risk factors for incomplete occlusion during follow-up (p<0.05).

Conclusion Reconstructive EVTs are a feasible and effective treatment for BTAs but are associated with a high risk of ischemic and hemorrhagic complications and a high mortality rate. Larger aneurysms may predict unfavorable clinical outcomes and aneurysm recurrence during follow-up. Hemorrhagic complications may predict unfavorable clinical outcomes, whereas immediate complete aneurysm occlusion may predict total occlusion during follow-up.

WHAT IS ALREADY KNOWN ON THIS TOPIC

⇒ Endovascular treatment (EVT) has gradually become the first-line therapy for posterior circulation aneurysms, but it is still technically challenging for basilar artery trunk aneurysms (BTAs). To date, few studies have evaluated the efficacy and safety of reconstructive EVT for BTAs.

WHAT THIS STUDY ADDS

⇒ The overall clinical and angiographic outcomes were favorable and acceptable; however, reconstructive EVT still had a high risk of ischemic and hemorrhagic complications and a high mortality rate for BTAs in this study.

HOW THIS STUDY MIGHT AFFECT RESEARCH, PRACTICE OR POLICY

⇒ Reconstructive EVTs are a feasible and effective treatment for BTAs, but larger cohort studies with long-term follow-up data are needed to validate our findings.

INTRODUCTION

Basilar artery (BA) trunk aneurysms (BTAs) are rare conditions comprising 0.95–2.1% of all intracranial aneurysms.[1 2] Non-saccular aneurysms—such as dissecting or fusiform aneurysms—occur more frequently in the BA trunk.[1] These may be incidental findings or present with subarachnoid hemorrhage (SAH), mass compression of surrounding structures, or ischemic stroke induced by perforating artery occlusion, resulting in high morbidity and mortality.[1 3–5] BTAs gradually increase in size and pose a higher risk of rupture than anterior circulation aneurysms.[1 3 4 6] Therefore, aggressive therapies should be considered for patients with symptomatic BTAs.

Surgical treatments—such as clipping or wrapping—are technically difficult for BTAs because of their deep location and proximity to cranial nerves and perforators. Endovascular treatment (EVT) has gradually become the first-line therapy for posterior circulation aneurysms, but it is still technically challenging for BTAs. Deconstructive EVT techniques (such as parent artery occlusion) are effective and have a higher occlusion rate for BTAs, but may cause severe neurologic complications due to insufficient collateral circulation after the parent vessel has been sacrificed.[2] Reconstructive EVT techniques (simple coils, conventional stents with or without coils, and the stent-in-stent technique) may be more suitable for BTAs but are associated with substantial morbidity and mortality and a higher recurrence rate during follow-up.[6 7] Flow

J NeuroIntervent Surg: first published as 10.1136/jnis-2022-019864 on 13 January 2023. Downloaded from http://jnis.bmj.com/ on January 23, 2023 by guest. Protected by copyright.

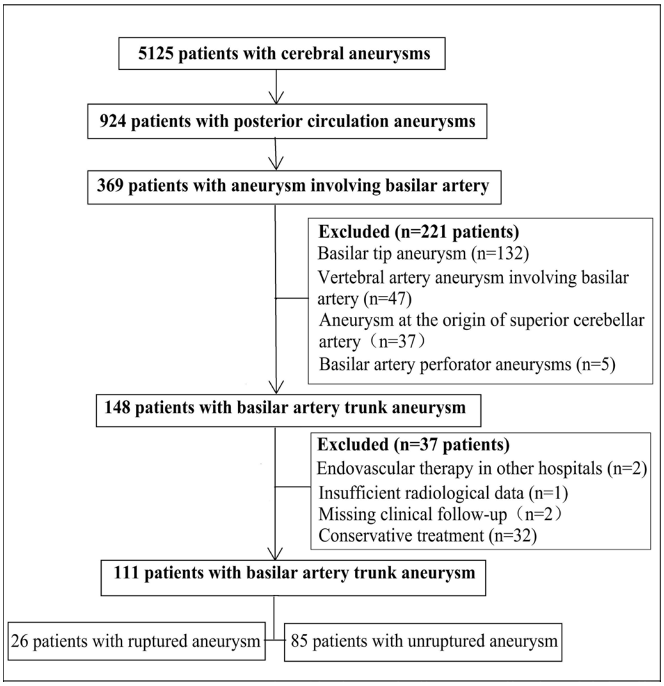

Figure 1 Flow chart of the study population.

diversion is an attractive treatment for BTAs, but it is controversial and off-label.[8] As such, the best reconstructive EVT for BTAs has not yet been established.

To date, few studies have evaluated the efficacy and safety of reconstructive EVT for BTAs. Previous studies often included small sample sizes, and several studies included both basilar and vertebral artery aneurysms,[6 9] the latter of which can differ from BTAs in terms of endovascular complications and disease outcomes.[5] Further studies are needed to evaluate the efficacy and safety of reconstructive EVT for BTAs. We present a large case series of BTAs treated with reconstructive EVT and evaluate the complications and clinical and angiographic outcomes.

MATERIALS AND METHODS

Patients

BTA was diagnosed when angiographic imaging showed a fusiform or irregular dilatation with/without stenosis in the affected BA trunk (from the vertebrobasilar junction to the origin of the superior cerebellar artery). Between January 2013 and February 2022, 5125 consecutive patients with cerebral aneurysms were treated at our tertiary hospital. Of these, 148 cases were diagnosed as BTA. We excluded two cases who received interventional therapy at other hospitals, three cases with insufficient radiological or clinical follow-up data, and 32 cases who received conservative treatment. Finally, 111 cases with BTA who underwent EVT were included (figure 1).

We retrospectively reviewed the medical records of the enrolled patients and collected the following information: patients' demographic, clinical and aneurysmal characteristics; procedural details and complications; and angiographic and clinical outcomes. The aneurysm morphology was classified as follows: saccular aneurysm (partial and lateral dilatation without stenosis); fusiform aneurysm (diffuse dilatation without stenosis); and pearl-and-string (successive dilatation with stenosis). Based on three-dimensional rotational angiography, the aneurysms were classified as circumferential (dilatation portion involving more than half the circumference of the parent artery) or eccentric (dilatation portion involving less than half the circumference of the parent artery) aneurysms. The aneurysm size (maximum measurement from the neck to the dome or the neck width of the dilatation portion) and the lesion length (the entire length of the involved portion of the BA) were recorded. An anterior inferior cerebellar artery (AICA) arising from the neck, body, or dome of the aneurysm indicated perforator involvement.

The main complications during hospitalization were hemorrhage, infarction, or hydrocephalus requiring shunting. Procedural complications included those that occurred intraoperatively or within 24 hours after EVT. Periprocedural complications included those that occurred during hospitalization. Clinical follow-up was performed via neurological examinations or telephone interviews. Clinical outcomes were assigned based on the modified Rankin scale (mRS) score at the last follow-up: 0–2, favorable outcome; 3–6, unfavorable outcome. Cerebral angiography was performed at 6, 12, and 36 months after EVT to confirm aneurysm occlusion and the patency of the BA. Radiological follow-up was performed if the patients experienced new-onset clinical symptoms during follow-up. Aneurysm occlusions on immediate and final follow-up cerebral angiography were categorized as complete occlusion (no filling of the contrast agent in the aneurysm sac) or incomplete occlusion (residual filling of the contrast agent in the aneurysm neck or sac).

Procedural details

Our management strategies included only coiling, stenting with/without coiling, and flow diversion. The optimal treatment strategy was evaluated based on the patients' neurological condition and comorbidities, the angioarchitectural features of the aneurysm, and the decision of the patient and their first-degree relatives. The conventional stents included Neuroform (Stryker Neurovascular, USA), Enterprise (Cordis Neurovascular, USA), and Solitaire (ev3, USA) stents, and a low-profile visualized intraluminal support (LVIS) device (MicroVention Terumo, USA) was used. The flow diverters included Tubridge (MicroPort NeuroTech, China) and Pipeline (Medtronic, USA) devices. In cases treated with stent-assisted coiling, patients with an unruptured aneurysm were routinely administered dual antiplatelet agents (75 mg clopidogrel and 100 mg aspirin) for at least 3 days before the procedure, and patients with a ruptured aneurysm were administered a loading dose of aspirin (300 mg) and clopidogrel (300 mg) 4–8 hours prior to the procedure. The diameter and length of the stent were chosen according to the dimensions of the affected parent vessel.

All EVT procedures were performed via a femoral artery approach under general anesthesia. An intravenous bolus of heparin (5000 IU) was administered before the procedure. Heparin was discontinued at the end of the procedure. A standard 6F or 8F guide catheter was advanced into the subclavian artery, proximal to the vertebral artery. An intermediate catheter (Navien, Medtronic) was then inserted into the V3 segment of the vertebral artery. A stent microcatheter was used to access the true lumen of the aneurysm in the posterior cerebral artery through the guidewire. For cases with additional coil insertion, a coil microcatheter was placed in the aneurysm sac. The stent was partially deployed to cover the aneurysm neck and temporarily jail the microcatheter, after which the aneurysm was loosely or densely packed with detachable coils before the stent was completely deployed. For long segmental lesions in which the aneurysm neck could not be covered completely with a single stent, an additional stent was extended into the bridged segment.

J NeuroIntervent Surg: first published as 10.1136/jnis-2022-019864 on 13 January 2023. Downloaded from http://jnis.bmj.com/ on January 23, 2023 by guest. Protected by copyright.

If a single stent was not sufficient to alter the intra-aneurysmal hemodynamics, overlapping stents were used to reconstruct the lesion.

Patients usually continued receiving dual antiplatelet therapy with clopidogrel (75 mg daily) for 3 months and aspirin (100 mg daily) for ≥6 months after treatment. Thromboelastography data were available for 58 patients. Patients who had insufficient responses to aspirin or clopidogrel received a substitute antiplatelet agent (cilostazol or ticagrelor).

Statistical analysis

SPSS (version 23.0, IBM Corp, Armonk, New York, USA) was used for statistical analysis. The one-sample Kolmogorov–Smirnov test was used to test the normality of the data distribution for continuous variables. Continuous variables are presented as mean and SD, and categorical variables are presented as numbers (frequency). Continuous variables were compared using the Student's t-test or Mann–Whitney U test, as appropriate. Categorical variables were compared using the χ^2 test or Fisher's exact test, as appropriate. Variables identified as potential predictors in univariate analysis ($p<0.1$) were included in binary logistic regression analysis (forward) to determine their status as independent predictors. A p value of <0.05 was considered statistically significant.

RESULTS

Baseline demographic and aneurysmal characteristics

This study included 81 men and 30 women aged 7–79 years (median 60 years) (see online supplemental table 1). At admission, 26 (23.4%) and 85 (76.6%) cases presented with SAH and unruptured aneurysms, respectively. The Hunt and Hess (HH) grades were as follows: grades I–III in 21 (18.9%) cases and grades IV–V in five (4.5%) cases with SAH. The modified Fisher scales were I–II in six (5.4%) cases and III–IV in 20 (18.1%) cases with SAH. Among cases with an unruptured aneurysm, 32 (28.8%) developed ischemic posterior circulation deficits, 31 (27.9%) presented with dizziness or headache, and 22 (19.8%) were asymptomatic (incidental findings).

The most common shape was saccular (62.2%), followed by fusiform (27.9%) and pearl-and-string (9.9%). There were 39 (35.1%) circumferential and 72 (64.9%) eccentric aneurysms; 19 (17.1%) cases involved the AICA and 16 (14.4%) coexisted with BA stenosis (>30%).

Treatments

Successful EVT was achieved in all patients: two cases (1.8%) underwent coiling without stents, 85 (76.6%) cases underwent stent-assisted coiling, and 24 cases (21.6%) underwent stenting without coiling (see online supplemental table 1). In cases with stent implantation, 70 (63.1%) received a single stent (including 14 who received a flow diverter), 28 (25.2%) received two conventional stents, and 11 (9.9%) received three conventional stents in an overlapping or telescoping fashion. Balloon angioplasty was performed in 13 (11.7%) cases, including 10 cases with parent artery stenosis and two cases of inadequate stent expansion. Immediate complete and incomplete aneurysm obliteration was achieved in 58 (52.3%) and 53 (47.7%) cases, respectively.

Complications during hospitalization

Procedure-related ischemic and hemorrhagic complications occurred in 28/111 (25.2%) and 3/111 (2.7%) cases, respectively (see online supplemental table 1). Overall, 27 (24.3%) patients

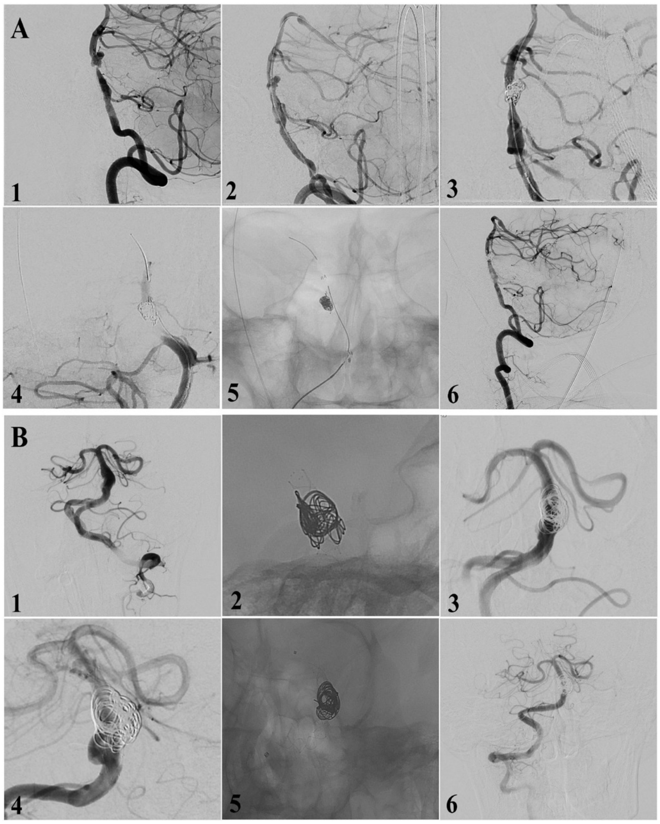

Figure 2 Typical case presentations. (A) A case with brainstem infarction had a basilar artery pearl-and-string aneurysm (1), balloon angioplasty was performed and (2) angiography showed the parent artery stenosis was improved. (3) Enterprise semi-deployed assisted coiling was performed and (4) intraoperative angiography showed intraluminal thrombus after coiling. Intra-microcatheter tirofiban administration, balloon angioplasty, and (5) additional Enterprise implantation were performed because the previous stent became displaced and collapsed. (6) Postoperative angiography showed total aneurysm occlusion with patency of the basilar artery. (B) A patient with headache and dizziness had a fusiform aneurysm (1). Double low-profile visualized intraluminal support device-assisted coiling was performed (2) and (3) postoperative angiography showed subtotal aneurysm occlusion. (4) Follow-up angiography (6 months later) showed residual aneurysms and (5) flow diverter-Tubridge was successfully deployed. (6) Follow-up angiography (12 months after the first treatment) showed total aneurysm occlusion with patency of the parent artery.

only experienced ischemic complications immediately after the procedure. Of these, one patient presented with intraoperative intraluminal thrombus. Intraoperative thrombolysis, balloon angioplasty, and additional implantation of an Enterprise device were performed; a mild neurological deficit was noticed after the procedure, but the patient recovered completely after 3 months (figure 2A). The procedure was successful in the other 26 cases, likely due to the occlusion of the perforator vessel or brainstem compression due to mass effect after treatment. One patient with saccular aneurysms experienced rebleeding during the procedure; however, the aneurysm was completely obliterated under the temporary protection of the balloon. This patient experienced brainstem ischemia immediately after treatment but had no neurological deficit after 3 months. One patient with SAH and one patient with dizziness at admission experienced brainstem hemorrhage within 12 hours after treatment and died within 1 month. Overall, procedure-related ischemic

J NeuroIntervent Surg: first published as 10.1136/jnis-2022-019864 on 13 January 2023. Downloaded from http://jnis.bmj.com/ on January 23, 2023 by guest. Protected by copyright.

and hemorrhagic complications occurred in 30/111 (27.0%) patients. In the 58 cases who underwent thromboelastography, neither adenosine diphosphate nor arachidonic acid inhibition rate was associated with procedural thrombosis or hemorrhagic complications (p>0.05). Aneurysm size was the only predictor of these complications (p<0.05) (see online supplemental table 2). Asymptotic BTAs tended to be associated with a lower rate of procedural ischemic and hemorrhagic complications (p=0.06).

The non-procedural complications were as follows: one patient experienced rebleeding 1 week after EVT and died; another patient with SAH experienced delayed cerebral infarction and died from pulmonary infection and septicemia; five patients (4.5%), including one with a large unruptured aneurysm and four patients with SAH, exhibited symptomatic hydrocephalus and underwent external ventricular drainage or ventriculoperitoneal shunt placement after embolization.

Clinical and radiological follow-up

Clinical follow-up was performed in all survivors for 6–121 months (median 36 months). One patient died in a traffic accident 10 months after discharge, one died from acute myocardial infarction after 7 years, and one died from cancer after 6 years. Moreover, 12 patients experienced new-onset complications during follow-up: two developed epilepsy and one developed progressive neurological defects. The main reason for these complications was mass effect on the brainstem, although aneurysm regrowth or recanalization cannot be excluded. New-onset posterior circulation ischemia occurred in seven cases due to early withdrawal of dual antiplatelet therapy (two cases) and in-stent stenosis after 10 months (one case). Parenchymal hemorrhage during dual antiplatelet therapy occurred in one case. One patient with a larger aneurysm experienced SAH and died after 1 month. The outcomes were favorable in 92/111 (83.8%) cases, and the overall mortality was 16/111 (14.4%). The main reasons for mortality were serious procedural ischemic complications (n=4), procedural brain hemorrhagic complications (n=2), aneurysm rebleeding (n=2), brainstem compression (n=2), new ischemic defects (n=2), parenchymal hemorrhage (n=1), initial poor HH grade (n=1), pulmonary infection (1 case with SAH), and infectious hydrocephalus (1 case with SAH). Older age, poor HH grade, increased aneurysm size, and hemorrhagic complications were independent risk factors for unfavorable clinical outcomes (p<0.05) (table 1).

Angiographic follow-up data for 3–90 months were available for 77/95 (81.1%) surviving patients. Overall, 55 (74.0%) aneurysms exhibited complete occlusion and 20 (26%) exhibited incomplete obliteration, including 11 cases (14.3%) with neck remnants and nine (11.7%) with residual aneurysms. Additional treatment was performed in two cases, resulting in complete occlusion at final follow-up (figure 2). The only patient with a fusiform aneurysm was treated with LVIS-assisted coiling and experienced symptomatic in-stent stenosis after 10 months. Increased aneurysm size and incomplete aneurysm occlusion on immediate angiography were independent risk factors for incomplete occlusion during follow-up (p<0.05) (table 2).

Table 1 Risk factors for unfavorable clinical outcomes in patients with basilar artery trunk aneurysms

	Clinical outcomes		Univariate analysis	Multivariate analysis		
	Favorable (n=92)	Unfavorable (n=19)	P value	OR	95% CI	P value
Age (years)	57±11	63±10	0.037	1.09	1.02 to 1.17	0.008
Women	24 (21.6)	6 (5.4)	0.624			
Smoking	41 (36.9)	6 (5.4)	0.297			
Diabetes	6 (5.4)	4 (3.6)	0.066			
Hypertension	65 (5.85)	16 (14.4)	0.226			
Hyperlipidemia	28 (25.2)	5 (4.5)	0.721			
Ischemic stroke	25 (22.5)	7 (6.3)	0.397			
Incidental finding	20 (18.0)	2 (1.8)	0.355			
Ruptured aneurysm	20 (18.0)	6 (5.4)	0.357			
HH grade (IV–V)	2 (1.8)	3 (2.7)	0.035	30.67	2.39 to 393.19	0.009
mFS score (III–IV)	14 (12.6)	6 (5.4)	0.107			
Sac aneurysm	56 (50.5)	13 (11.7)	0.537			
Circumferential aneurysm	31 (27.9)	8 (7.2)	0.485			
Coexisting BA stenosis	15 (13.8)	1 (0.9)	0.298			
AICA involvement	11 (9.9)	8 (7.2)	0.001			
Multiple stents	32 (28.8)	7 (6.3)	0.864			
Flow diversion	11 (9.9)	3 (2.7)	0.705			
Complete aneurysm obliteration	49 (44.1)	9 (8.1)	0.640			
Infarction complication	19 (17.1)	10 (9.0)	0.004			
Hemorrhage complication	1 (0.9)	3 (2.7)	0.016	33.98	1.12 to 1031.37	0.043
Hydrocephalus requiring shunting	2 (1.8)	3 (2.7)	0.035			
Aneurysm size (mm)	9.2±5.8	15.2±7.7	0.004	1.22	1.10 to 1.34	<0.001
Lesion length (mm)	8.3±6.0	13.7±7.8	0.001			

Variables are expressed as mean±SD or number (%) of patients.
AICA, anterior inferior cerebellar artery; BA, basilar artery; HH, Hunt and Hess; mFS, modified Fisher scale.

J NeuroIntervent Surg: first published as 10.1136/jnis-2022-019864 on 13 January 2023. Downloaded from http://jnis.bmj.com/ on January 23, 2023 by guest. Protected by copyright.

Table 2 Risk factors for unfavorable angiographic outcomes in patients with available cerebral angiographic data

	Angiographic outcomes		Univariate analysis	Multivariate analysis		
	Unfavorable (n=21)	Favorable (n=56)	P value	OR	95% CI	P value
Age (years)	59±9	57±11	0.467			
Women	7 (9.1)	16 (20.8)	0.684			
Smoking	8 (10.4)	22 (28.6)	0.924			
Diabetes	1 (1.3)	5 (6.5)	1.000			
Hypertension	15 (19.5)	42 (54.5)	0.750			
Hyperlipidemia	8 (10.4)	17 (22.1)	0.518			
Ischemic stroke	6 (7.8)	13 (16.9)	0.627			
Incidental finding	4 (5.2)	12 (15.6)	0.819			
Ruptured aneurysm	5 (6.5)	15 (19.5)	0.791			
HH grade (IV–V)	0 (0.0)	3 (3.9)	0.558			
mFS score (III–IV)	4 (5.2)	11 (9.9)	1.000			
Sac aneurysm	8 (10.4)	40 (51.9)	0.007			
Circumferential aneurysm	16 (20.8)	12 (15.6)	< 0.001			
Coexisted with BA stenosis	2 (2.6)	7 (9.1)	1.000			
AICA involved	6 (7.8)	4 (5.2)	0.021			
Multiple stents	10 (13.0)	21 (27.3)	0.420			
Flow diversion	3 (3.9)	4 (5.2)	0.383			
Incomplete obliteration	17 (22.1)	13 (16.9)	< 0.001	7.33	1.84 to 29.28	0.005
Aneurysm size (mm)	15.4±5.7	7.6±4.7	< 0.001	1.22	1.08 to 1.38	0.002
Aneurysm length (mm)	13.5±5.8	5.0±5.5	< 0.001			

Variables are expressed as mean±SD or number (%) of patients.
AICA, anterior inferior cerebellar artery; BA, basilar artery; HH, Hunt and Hess; mFS, modified Fisher scale.

DISCUSSION

Overall, the rates of periprocedural ischemic and hemorrhagic complications in this study were 26.1% and 3.6%, respectively. The rate of favorable clinical outcomes was acceptable (83.8%), but the mortality rate was still high (14.4%). Complete aneurysm obliteration was achieved in 74% of cases with available follow-up angiographic data.

BTAs comprised 2.9% of all intracranial aneurysms treated at our center, were more common in men, and occurred mostly in the fifth to seventh decade of life. Although neuroimaging techniques have improved the detection of BTAs, the natural history of asymptomatic BTAs is still unclear. Non-saccular aneurysms are more common in the BA trunk, tend to be unstable, and increase in size over time.[1] Incidentally detected BTAs had a lower rate of ischemic and hemorrhagic complications during EVT and showed good outcomes.[10] Therefore, this treatment should be considered for patients with asymptomatic BTAs. However, clinicians should fully evaluate the patients, their aneurysmal characteristics, the natural history of the aneurysm, and the feasibility of the technique.

The overall rate of procedure-related ischemic and hemorrhagic complications (27.0%) was relatively high, although previous studies have reported similar rates.[7 11] The most common complication in our study was ischemia, likely due to perforator infarction, mass effects, or intraluminal thrombus formation. Perforator infarction can be caused by compression, distortion, traction, or coverage of the perforator arteries during coiling or stent deployment. Increased aneurysm size predicts procedural complications,[8 12] and larger aneurysms typically exhibit formation of intraluminal thrombi (which can detach and spread during coiling or stent deployment). Coiling and post-treatment aneurysm thrombosis formation could further increase the mass effects. Meanwhile, larger aneurysms typically involve a longer BA segment. The longer the BA segment involved, the higher the number of perforator arteries that would be involved and potentially damaged during treatment. Larger and longer lesions make the treatment challenging and require flow diversion or overlapping stents. Increased metal coverage can increase the risk of perforator artery occlusion. Incomplete embolization or poor vessel wall apposition of the stent is more common in larger aneurysms; the newly-formed thrombus can be fragmented and escape even after the procedure.

Adequate and regular antiplatelet therapy was important for patients who underwent stent implantation. Although platelet function was not associated with procedural thrombosis or hemorrhagic complications in this study, platelet function test data were not available for all cases and statistical bias may exist. Hence, routine platelet function testing for adjusting antiplatelet drugs to reduce complications warrants further study.

Ischemic onset has been reported as a predictor of cerebral ischemic complications.[6] In this study, asymptomatic BTAs tended to be associated with a lower rate of procedural ischemic and hemorrhagic complications. Incidentally detected BTAs may be small in size and are technically simpler to treat. However, symptomatic aneurysms are usually larger and unstable, and cerebrovascular capacity may be inadequate to compensate for perforator occlusion during treatment.

Previous studies have reported a 65–86% rate of favorable clinical outcomes after EVT for BTAs,[8 11–13] which is similar to our results (83.4%). Sim *et al* reported that a serious HH grade at baseline was the only independent risk factor for unfavorable clinical outcomes.[14] A similar association was found in our study. A serious admission HH grade indicated severe brain injury, serious inflammatory and oxidative stress response, and higher

J NeuroIntervent Surg: first published as 10.1136/jnis-2022-019864 on 13 January 2023. Downloaded from http://jnis.bmj.com/ on January 23, 2023 by guest. Protected by copyright.

complication and mortality rates. Older age also predicted unfavorable outcomes in this study. Symptomatic BTAs may be common in older patients; elderly patients have more chronic diseases, perioperative complications, poor tolerance to complications, and slow and incomplete recovery. As mentioned above, larger aneurysms are associated with procedural complications and tend to recur and rupture even after complete occlusion. Aneurysm size was an important predictor of clinical outcome in this study.[8]

In addition, hemorrhagic complications—rather than ischemic complications—were associated with poor clinical outcomes in this study. Although ischemic complications were more common, most such cases were mild or gradually recovered with satisfactory clinical outcomes after the administration of antiplatelet drugs during follow-up. However, most patients with hemorrhagic complications died. AICA involvement may be associated with an unfavorable clinical outcome, indicated a high risk of procedural perforator ischemic events, and such patients had a high risk of residual and recurrent aneurysms during follow-up.

Previous studies have reported a complete occlusion rate of 66.7–75% during follow-up after EVT,[8 12 14 15] which is similar to our results (74% in cases with follow-up angiographic data available). The degree of aneurysm occlusion on immediate angiography was an independent risk factor for unfavorable angiographic outcome. Complete obliteration remains the primary goal of EVT and dense packing with adjunctive coils should be considered, although larger or fusiform aneurysms may not always show immediate complete occlusion. Increased aneurysm size was an important predictor of aneurysm recurrence in this study. Large aneurysms are associated with a lower packing density, more intra-aneurysmal flow, and a higher incidence of intraluminal thrombus formation; therefore, they may be more susceptible to coil compaction and/or coil loosening, even when densely packed during treatment.[16 17]

Saccular aneurysms may be a prognostic factor for favorable angiographic outcome for BTAs. Saccular aneurysms are partial and lateral dilatations with a distinct aneurysm neck; hence, conventional stent-assisted dense packing is easier to achieve. However, fusiform aneurysms are usually circumferentially dilated aneurysms with various sizes and shapes. It is difficult to achieve dense coil packing of every partition of the aneurysm lumen. Although multiple stent deployment can help increase stent metal coverage and reduce blood flow into the aneurysm, potentially reducing the risk of recurrence and rebleeding,[18] multiple stents were not associated with procedural complications in this study. However, favorable angiographic results are rarely reported in cases with multiple stent deployment.[14] The difficulties in the deployment of overlapping stents should also be considered.

Flow diverters may be an alternative for larger and complex BTAs. They have higher metal coverage and provide excellent flow diversion from the aneurysmal lumen. This could promote progressive aneurysm occlusion over time. Flow diversion has a higher complete occlusion rate and a lower retreatment rate compared with traditional coil embolization,[19 20] and it produces more favorable angiographic and clinical outcomes than traditional stent-assisted coiling in patients with large or giant basilar trunk and vertebrobasilar junction aneurysms.[7] However, the effects of flow diverters could not be elucidated in this study, perhaps due to small aneurysm size and short follow-up time. Furthermore, increased metal coverage did not increase the risk of procedural complications.[7] Flow diverters can avoid entering the aneurysm sac, thereby reducing the risk of iatrogenic rupture and intraluminal thrombus escape during coiling, while the vital arterial branches covered by the shunt retain patency. Thromboembolic and hemorrhagic complications have declined with improved operator experience using the flow diverters for posterior circulation aneurysms.[20] Although flow diverters are not a first-line treatment, they may be valuable and could be selectively considered for complex fusiform or giant dissecting aneurysms that are not amenable to conventional EVT. Prospective studies with larger sample sizes are necessary to evaluate the feasibility and effectiveness of flow diverters for treating BTAs.

This study has several limitations. First, the study was conducted retrospectively at a single center and the sample size was small. Second, the method of selecting patients, including the assessment of the morphological and pathological characteristics of the aneurysms, was not standardized throughout the study. Third, diverse aneurysms require non-homogeneous treatment modalities. The strategies and techniques used in the procedures changed over time, and the indications for flow diverters have been expanded. Furthermore, different treatment modalities have different characteristics and may cause different complications and outcomes. Fourth, this study lacks a control group; the small sample size was insufficient to include all variables and to identify all relevant factors in the statistical analysis. Fifth, aneurysm intraluminal thrombus before EVT and platelet function test data were not available for all cases, and the lack of a platelet function test might be related to thromboembolic or hemorrhagic events. Finally, angiographic follow-up data were not available for all included patients. The total angiographic follow-up period was not uniform and the follow-up period might not have been sufficient to observe aneurysm occlusion in flow diverter-treated cases. All the above factors may limit the generalizability of our findings.

CONCLUSION

Reconstructive EVTs are a feasible and effective treatment for BTAs but are associated with a high mortality rate and high risk of ischemic and hemorrhagic complications. Larger aneurysms and hemorrhagic complications may lead to unfavorable clinical outcomes, whereas increased aneurysm size and immediate complete aneurysm occlusion may predict total occlusion during follow-up.

Contributors WZ and TZ: article drafting and writing. CS and DZ: data collection and statistics. JZ, ML, YX, and ML: collection of original data. MZ, YW, DW, and WS: review of the article. All authors contributed to the article and approved the submitted version.WS is the author acting as guarantor.

Funding This study was sponsored by the Natural Science Foundation of Shandong Province (grant number: ZR2022MH131).

Competing interests None declared.

Patient consent for publication Not applicable.

Ethics approval This retrospective study was approved by the ethics committee of Qilu Hospital of Shandong University. This is a retrospective study and the requirement for informed consent was waived.

Provenance and peer review Not commissioned; externally peer reviewed.

Data availability statement Data are available upon reasonable request.

Supplemental material This content has been supplied by the author(s). It has not been vetted by BMJ Publishing Group Limited (BMJ) and may not have been peer-reviewed. Any opinions or recommendations discussed are solely those of the author(s) and are not endorsed by BMJ. BMJ disclaims all liability and responsibility arising from any reliance placed on the content. Where the content includes any translated material, BMJ does not warrant the accuracy and reliability of the translations (including but not limited to local regulations, clinical guidelines, terminology, drug names and drug dosages), and is not responsible for any error and/or omissions arising from translation and adaptation or otherwise.

Open access This is an open access article distributed in accordance with the Creative Commons Attribution Non Commercial (CC BY-NC 4.0) license, which

J NeuroIntervent Surg: first published as 10.1136/jnis-2022-019864 on 13 January 2023. Downloaded from http://jnis.bmj.com/ on January 23, 2023 by guest. Protected by copyright.

permits others to distribute, remix, adapt, build upon this work non-commercially, and license their derivative works on different terms, provided the original work is properly cited, appropriate credit is given, any changes made indicated, and the use is non-commercial. See: http://creativecommons.org/licenses/by-nc/4.0/.

ORCID iD
Wandong Su http://orcid.org/0000-0002-1114-665X

REFERENCES

1 Saliou G, Sacho RH, Power S, *et al*. Natural history and management of basilar trunk artery aneurysms. *Stroke* 2015;46:948–53.
2 Sönmez Ö, Brinjikji W, Murad MH, *et al*. Deconstructive and reconstructive techniques in treatment of vertebrobasilar dissecting aneurysms: a systematic review and meta-analysis. *AJNR Am J Neuroradiol* 2015;36:1293–8.
3 Flemming KD, Wiebers DO, Brown RD, *et al*. The natural history of radiographically defined vertebrobasilar nonsaccular intracranial aneurysms. *Cerebrovasc Dis* 2005;20:270–9.
4 Kim BM, Suh SH, Park SI, *et al*. Management and clinical outcome of acute basilar artery dissection. *AJNR Am J Neuroradiol* 2008;29:1937–41.
5 Yoshimoto Y, Hoya K, Tanaka Y, *et al*. Basilar artery dissection. *J Neurosurg* 2005;102:476–81.
6 Wu Q, Xu S, Wang C, *et al*. Endovascular management of vertebrobasilar trunk artery large aneurysms: complications and long-term results. *Front Neurol* 2022;13:839219.
7 Peng Q, Zhou Y, Li W, *et al*. Reconstructive endovascular treatment of basilar trunk and vertebrobasilar junction aneurysms: a review of 77 consecutive cases. *Front Neurol* 2022;13:885776.
8 Ge H, Chen X, Liu K, *et al*. Endovascular treatment of large or giant basilar artery aneurysms using the pipeline embolization device: complications and outcomes. *Front Neurol* 2022;13:843839.
9 Cho DY, Kim B-S, Choi JH, *et al*. The fate of unruptured intracranial vertebrobasilar dissecting aneurysm with brain stem compression according to different treatment modalities. *AJNR Am J Neuroradiol* 2019;40:1924–31.
10 van Oel LI, van Rooij WJ, Sluzewski M, *et al*. Reconstructive endovascular treatment of fusiform and dissecting basilar trunk aneurysms with flow diverters, stents, and coils. *AJNR Am J Neuroradiol* 2013;34:589–95.
11 Wang Y, Xu K, Song J, *et al*. Endovascular therapy for basilar arterial trunk aneurysms. *Front Neurol* 2021;12:625909.
12 Sim SY, Choi JH, Kim MJ, *et al*. Endovascular treatment of ruptured basilar artery trunk aneurysm in the acute period: risk factors for periprocedural complications. *Neurol Res* 2022;24:1–8.
13 Li L, Li T, Xue J, *et al*. Stent treatment for basilar artery dissection: a single-center experience of 21 patients. *Interv Neuroradiol* 2016;22:260–5.
14 Sim SY, Chung J, Choi JH, *et al*. Basilar artery trunk aneurysm: clinical and angiographic outcomes of endovascular treatment. *J Neurointerv Surg* 2022;14:262–7.
15 Cho K-C, Jeon P, Kim BM, *et al*. Saccular or dissecting aneurysms involving the basilar trunk: endovascular treatment and clinical outcome. *Neurol Res* 2019;41:671–7.
16 Zhang Q, Jing L, Liu J, *et al*. Predisposing factors for recanalization of cerebral aneurysms after endovascular embolization: a multivariate study. *J Neurointerv Surg* 2018;10:252–7.
17 Ferns SP, Sprengers MES, van Rooij WJ, *et al*. Coiling of intracranial aneurysms: a systematic review on initial occlusion and reopening and retreatment rates. *Stroke* 2009;40:e523–9.
18 Zhao K-J, Fang Y-B, Huang Q-H, *et al*. Reconstructive treatment of ruptured intracranial spontaneous vertebral artery dissection aneurysms: long-term results and predictors of unfavorable outcomes. *PLoS One* 2013;8:e67169.
19 Li S, Zeng C, Tao W, *et al*. The safety and efficacy of flow diversion versus conventional endovascular treatment for intracranial aneurysms: a meta-analysis of real-world cohort studies from the past 10 years. *AJNR Am J Neuroradiol* 2022;43:1004–11.
20 Dmytriw AA, Dibas M, Adeeb N, *et al*. The Pipeline Embolization Device: a decade of lessons learned in the treatment of posterior circulation aneurysms in a multicenter cohort. *J Neurosurg* 2022;137:1454–61.

J NeuroIntervent Surg: first published as 10.1136/jnis-2022-019864 on 13 January 2023. Downloaded from http://jnis.bmj.com/ on January 23, 2023 by guest. Protected by copyright.
