# Supplementary material for: Management strategies and outcomes of basilar trunk aneurysms: a systematic review and meta-analysis
Source: Langenbecks Arch Surg. 2026 Jan 20;411(1):71. doi: 10.1007/s00423-025-03959-3 (PMC12852281; doi:10.1007/s00423-025-03959-3)
Supplement: Supplementary file 1 — Supplementary file1 (ZIP 55327 KB) [file 423_2025_3959_MOESM1_ESM.zip › literature.zip!/meta/╤¬╣▄─┌╓╬┴╞╬┤╞╞┴╤╫╡ú¡╗∙╡╫╢»┬÷╝╨▓π╢»┬÷┴÷╡─┴╞╨o╖╓╬÷ (1).pdf]

·临床研究·

血管内治疗未破裂椎-基底动脉夹层动脉瘤的疗效分析

马宁 张宝瑞 冯欣 王璐瑶 彭飞 刘爱华

【摘要】 目的 分析未破裂椎-基底动脉夹层动脉瘤的临床特点及不同血管内治疗技术的效果。**方法** 回顾性分析北京市天坛医院神经介入科 2012 年 1 月至 2016 年 12 月连续 160 例患者(175 个未破裂椎-基底动脉夹层动脉瘤)的临床资料。175 个动脉瘤均行血管内治疗,其中支架辅助弹簧圈栓塞 115 个,单纯支架成形 27 个,血流导向装置置入 21 个,动脉瘤栓塞 + 载瘤动脉闭塞12 个。术后行影像学及临床随访。**结果** 头痛包括颈枕部痛(43.1%)是最常见的临床表现。围手术期并发症发生率 3.75%(6/160),无术中出血病例,无死亡病例。113 个动脉瘤影像学随访(10 ± 7)个月。71 个支架辅助弹簧圈栓塞动脉瘤中,62 个治愈,9 个复发;19 个单纯支架成型治疗的动脉瘤,4 个治愈,11 个好转,2 个稳定,2 个复发;15 个行血流导向装置治疗的动脉瘤,4 个治愈,11 个好转;8 个载瘤动脉闭塞的动脉瘤,8 个均治愈。144 例患者临床随访(17 ± 17)个月,随访改良 Rankin 量表(mRS)评分:0 ~ 2 分 140 例,3 ~ 6 分 4 例。**结论** 未破裂椎-基底动脉夹层动脉瘤患者临床表现复杂,头痛是最常见的临床症状。血管内治疗未破裂椎-基底动脉夹层动脉瘤方法多样,临床总体治疗效果良好。选择治疗方法时应遵循个体化的原则。

【关键词】 夹层动脉瘤; 基底动脉; 支架; 栓塞,治疗性

基金项目:国家自然科学基金(81771233)

Efficacy analysis of the endovascular treatment for 175 unruptured vertebrobasilar dissecting aneurysms

Ma Ning, Zhang Baorui, Feng Xin, Wang Luyao, Peng Fei, Liu Aihua. *Department of Interventional Neuroradiology, Beijing Tiantan Hospital, Capital Medical University, Beijing 100050, China*

Corresponding author: Liu Aihua, Email: liuaihuadoctor@163. com

【Abstrat】 Objective To analyze the clinical characteristics, clinical effect and follow-up outcome of the different endovascular treatment techniques in the treatment of unruptured vertebrobasilar dissecting aneurysms (VBDAs). **Methods** The clinical data of 160 consecutive patients (175VBDAs) from January 2012 to December 2016 in Beijing Tiantan hospital were retrospectively analyzed. All of the 175 aneurysms were treated with endovascular embolization, including 115 stent-assisted coils, 27 simple stents, 21 blood flow diverting devices, and 12 parent arteries occlusion. The imaging and clinical follow-up were performed after the operation. **Results** Headache including cervical-occipital pain (43.1%) was the most common clinical manifestation. The incidence of perioperative complications was 3.75%, no intraoperative bleeding and no deaths. The imaging findings of 113 aneurysms were followed up for (9.9 ± 7.3) months. Of the 71 stent-assisted coils, 62 recovered well and 9 relapsed; of the 19 aneurysms treated with simple stent, 4 recovered well, 11 improved, 2 stable and 2 relapsed; of the 15 aneurysms treated by the blood flow diverting devices, 4 recovered and 11 improved; all of the 8 aneurysms with parent arteries occlusion recovered well. A total of 144 patients were follow-up (17.3 ± 16.6) months by the Modified Rankin Scale (mRS) score: 140 patients were 0 - 2 score and 4 patients were 3 - 6 score. **Conclusions** The clinical manifestations of the unruptured VBDAs are complex, and the headache is the most common clinical symptom. Endovascular treatment for the treatment of unruptured VBDAs is safe and feasible. The principle of individualization should be followed during embolization.

【Key words】 Aneurysm, dissecting; Basilar artery; Stents; Embolization, theatment

Fund program: National Natural Science Foundation of China(81771233)

颅内夹层动脉瘤年发病率为 1 ~ 1.5/100 000 人,通常发生于35 ~ 50 岁青壮年中[1]。夹层动脉瘤一旦破裂出血,二次出血率达到 30%,病死率将高达 46% 以上[2],因此众多学者倾向于积极治疗该疾

DOI:10.3760/cma.j.issn.0376-2491.2018.27.010

作者单位:100050 首都医科大学附属北京天坛医院神经介入科(马宁现在山西医科大学第一医院神经外科)

通信作者:刘爱华,Email:liuaihuadoctor@163.com

Q2xvdWRWZXJzaW9uLQo?

病[3]。本文通过对 160 例患者共 175 个未破裂椎-基底动脉夹层动脉瘤(VBDA)的临床特点及不同血管内治疗技术的随访资料进行分析,以期为临床治疗提供参考。现报道如下。

对象与方法

1. 对象:北京市天坛医院神经介入科 2012 年 1 月至 2016 年 12 月连续收治的 160 例 VBDA 患者,其中男 134 例,女 26 例,年龄 4 ~ 76 岁,平均(51 ± 11)岁。通过数字减影血管造影(DSA)证实,160 例患者共发现 175 个夹层动脉瘤。其中椎动脉夹层动脉瘤 161 个,基底动脉夹层动脉瘤 9 个,椎基底联合部位夹层动脉瘤 5 个。累及小脑下后动脉(PICA)的动脉瘤 55 个。

2. 治疗方法:160 例未破裂 VBDA 患者均择期行血管内介入手术治疗。术前口服阿司匹林肠溶片(100 mg/d),氯吡格雷(75 mg/d)共 3 ~ 5 d。术中全身肝素化,术后行即刻全脑血管造影明确栓塞效果并观察载瘤动脉及分支血管是否通畅。放置支架患者术后继续口服阿司匹林肠溶片(100 mg/d),终身服用;氯吡格雷(75 mg/d),持续 6 周后停药。

3. 随访方法:所有患者术后来医院和(或)电话随访,记录影像学变化及临床预后情况。改良 Rankin(mRS)量表评分评估临床预后。影像学随访结果分为:(1)治愈:动脉瘤腔内无对比剂进入,动脉瘤不显影;(2)好转:动脉瘤腔内有血栓形成,与术后即刻造影相比,瘤腔缩小,对比剂显影减少;(3)稳定:与术后即刻造影比较无明显变化;(4)复发:与术后即刻造影比较瘤腔内对比剂显影增加。

结 果

1. 临床特点:160 例患者中最常见的症状是头痛包括枕颈部痛共 69 例(43.1%),性别差异无统计学意义。单纯以眩晕起病的 44 例(27.5%),颅神经功能障碍的 10 例(6.25%),恶心呕吐、言语不利等症状也均见于男女性患者中。24 例(17.9%)出现偏身运动、感觉障碍,意识障碍、视物不清、复视、失忆、吞咽困难等症状仅表现在男性患者当中。男性患者临床表现较女性复杂。此外,27 例(16.9%)无临床症状,体检发现夹层动脉瘤。表 1。

2. 手术结果:175 个未破裂 VBDA,支架辅助弹簧圈栓塞 115 个,术后即刻造影显示 98 个动脉瘤致密栓塞,17 个大部栓塞;单纯支架成形 27 个,其中 22 个为多重支架置入,5 个为单一支架置入,术后造影显示瘤体内均出现不同程度的对比剂滞留;血流导向装置(Pipeline)置入 21 个,19 个瘤体内对比剂滞留明显,2 个动脉瘤未见显影;动脉瘤栓塞 + 载瘤动脉闭塞 12 个,术后造影发现载瘤动脉完全闭塞,夹层动脉瘤不显影。所有分支血管术后均保持通畅。

表 1 160 例未破裂 VBDA 患者的临床症状[例(%)]

临床症状	合计 (n = 160)	男性 (n = 134)	女性 (n = 26)	P 值
头痛	44(27.5)	32(23.9)	12(46.2)	0.020
枕颈部痛	25(15.6)	20(14.9)	5(19.2)	0.796
眩晕	44(27.5)	34(25.4)	10(38.5)	0.171
体检发现	27(16.9)	25(18.7)	2(7.7)	0.280
偏身运动、感觉障碍	24(15.0)	24(17.9)	0(0.0)	1.000
颅神经功能障碍	10(6.25)	7(5.2)	3(11.5)	0.439
恶心呕吐	7(4.4)	5(3.7)	2(7.7)	0.704
饮水呛咳	6(3.75)	5(3.7)	1(3.9)	1.000
意识障碍	5(3.1)	5(3.7)	0(0.0)	0.700
言语不利	4(2.5)	3(2.2)	1(3.9)	1.000
复视	2(1.25)	2(1.5)	0(0.0)	1.000
视物模糊	2(1.25)	2(1.5)	0(0.0)	1.000
失忆	2(1.25)	2(1.5)	0(0.0)	1.000
吞咽困难	2(1.25)	2(1.5)	0(0.0)	1.000
头皮发麻	2(1.25)	2(1.5)	0(0.0)	1.000
耳鸣	1(0.6)	1(0.8)	0(0.0)	1.000

围手术期无术中破裂出血病例,无死亡病例。6 例(3.75%)出现术后并发症。其中 3 例患者行载瘤动脉闭塞术,术后有共济失调、颅神经功能障碍表现,经积极治疗后症状好转;2 例患者行支架辅助弹簧圈栓塞治疗,术后出现脑干缺血症状,表现为一侧肢体运动及感觉功能障碍,经保守治疗后 1 例病情好转,1 例出现残疾;1 例行 Pipeline 治疗,术后合并脑梗死,对症保守治疗后症状消失,病情恢复。

3. 随访结果(图 1):113 个未破裂 VBDA 术后复查 DSA,随访(10 ± 7)个月。其中 78 个(69.0%)治愈,22 个(19.5%)好转,2 个(1.8%)稳定,11 个(9.7%)复发。复发动脉瘤 9 个行 2 次血管内介入治疗后继续跟踪随访;2 个未治院外观察。表 2。

160 例患者术后行门诊和(或)电话临床随访,16 例患者失随访。144 例患者平均随访时间(17 ± 17)个月。长期随访 mRS 评分:0 ~ 2 分 140 例(97.2%),3 ~ 6 分 4 例(2.8%)。表 3。

讨 论

未破裂 VBDA 临床表现多种多样[4]。头痛尤其是单侧枕颈部的疼痛是最常见的临床症状之一,本组43.1% 患者出现上述症状,与文献报道的

Q2xvdWRWZXJzaW9uLQo?

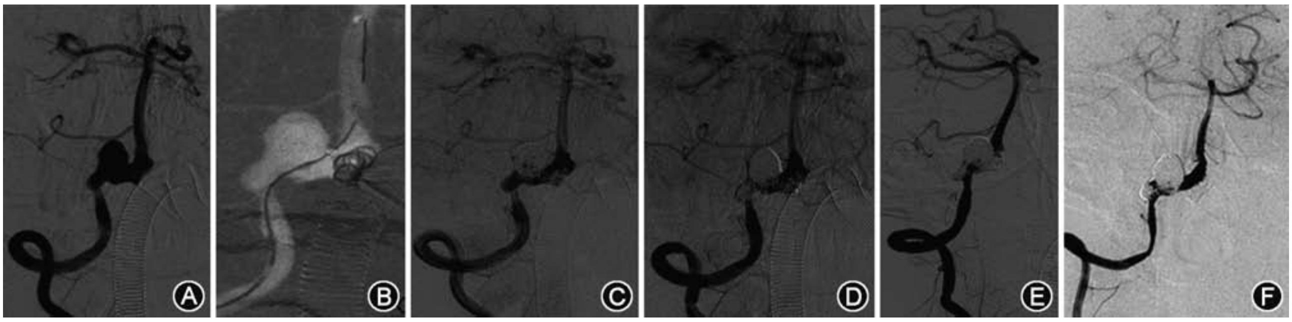

图 1　椎-基底动脉夹层动脉瘤患者 DSA 表现。A：右侧椎动脉造影显示右侧椎-基底动脉夹层动脉瘤，动脉瘤大小约 10.9 mm×11.6 mm；B：经支架微管释放 Enterprise(4.5 mm×37 mm)支架一枚，覆盖动脉瘤颈，并送入弹簧圈栓塞椎-基底结合部夹层动脉瘤；C：依次送入弹簧圈继续栓塞夹层动脉瘤主体部分；D，E：动脉瘤栓塞完毕即刻造影显示支架完全覆盖瘤颈，动脉瘤完全栓塞，载瘤动脉通畅；F：术后 6 个月复查，右侧椎-基底夹层动脉瘤未见复发，弹簧圈及支架位置良好，载瘤动脉形态良好

表 2　113 个未破裂椎-基底动脉夹层动脉瘤患者不同治疗策略的影像学随访结果的单因素分析[例(%)]

组别	个数	未复发组			复发组	风险比(OR)	P 值
		治愈	好转	稳定			
支架辅助弹簧圈栓塞术	71(62.8)	62(87.3)	0(0.0)	0(0.0)	9(12.7)	2.903(0.596～14.136)	0.187
单纯支架置入术	19(16.8)	4(21.1)	11(57.9)	2(10.5)	2(10.5)	1.111(0.220～5.605)	0.898
血流导向装置置入术	15(13.3)	4(26.7)	11(73.3)	0(0.0)	0(0.0)	0.888(0.827～0.953)	0.172
载瘤动脉闭塞术	8(7.1)	8(1.0)	0(0.0)	0(0.0)	0(0.0)	0.895(0.839～0.956)	0.335
合计	113(1.0)	78(69.0)	22(19.5)	2(1.8)	11(9.7)	无统计学意义	无统计学意义

表 3　未破裂椎-基底动脉夹层动脉瘤患者不同治疗策略的临床随访结果的单因素分析[例(%)]

组别	例数	mRS 评分		风险比(OR)	P 值
		0～2 分	3～6 分		
支架辅助弹簧圈栓塞术	95(66.0)	92(96.8)	3(3.2)	1.565(0.159～15.455)	0.699
单纯支架置入术	21(14.6)	20(95.2)	1(4.8)	2.000(0.198～20.192)	0.549
血流导向装置置入术	17(11.8)	17(100)	0(0)	0.969(0.939～1.000)	0.458
载瘤动脉闭塞术	11(7.6)	11(100)	0(0)	0.970(0.941～1.000)	0.560
合计	144(1.0)	140(97.2)	4(2.8)	无统计学意义	无统计学意义

42.4%相符[5]。疼痛原因可能是椎动脉被大量痛觉纤维支配，瘤体扩张牵拉所致。38.2%的患者出现眩晕、呕吐、神经功能障碍的临床表现，考虑巨大的动脉瘤压迫脑干及颅神经而引起，高于国内报道的 24%的发病率[6]；47.5%的患者以共济失调，眩晕、偏身运动、感觉障碍，饮水呛咳等类似于 Wallenberg 综合征的脑干缺血症状起病，符合国外报道的 26%～62%[7]。对于有临床表现特别是头痛的患者，应引起足够的重视，积极进行诊疗。

当前血管内治疗未破裂 VBDA 方法多样，治疗方案选择有争议[3]。

吴中学等[8]建议治疗椎-基底动脉夹层动脉瘤首选支架辅助弹簧圈技术。支架辅助弹簧圈栓塞，支架可以改变动脉瘤腔内的血流模式，促进瘤腔内血栓形成。可以使弹簧圈稳定栓塞瘤腔，防止逃逸。尤其是可回收支架，以及支架半释放技术的应用，支架辅助弹簧圈栓塞成功率大大提高。但是仍存在术中支架移位，术后弹簧圈压缩、新夹层形成，复发率高的缺点。且由于动脉瘤与 PICA 的位置变异较大，支架辅助弹簧圈栓塞后复发率仍较高。Kim 等[9]称夹层动脉瘤累及小脑下后动脉是影响复发的独立危险因素。本组 65.7%个未破裂 VBDA 应用该技术，围手术期仅 2 例出现脑干缺血的并发症。95 例术后临床随访有 96.8%预后良好，疗效满意。71 个动脉瘤术后 12.7%复发。复发病例中 8 个动脉瘤与 PICA 位置关系密切，致密填塞可能导致脑干及小脑缺血而引起严重并发症发生，因此选择支架辅助弹簧圈大部栓塞术。

血管内放置支架可以改变受损伤血管的血流动力学，改变血流对动脉瘤冲击的方向，阻止瘤体继续生长；同时支架释放的张力压迫内膜，使血管内膜进行自身修复，达到解剖愈合，重建的血管壁将动脉瘤

排除在循环系统之外,这就能有效的避免动脉瘤发生破裂出血[10]。此技术操作简便,无需在血管内行弹簧圈栓塞,降低了手术风险。对于瘤体较长的夹层动脉瘤,则应选择多重支架置入。它能够有效的覆盖夹层动脉瘤,并进一步降低支架的孔隙率,增加血管内的径向支撑力,改变载瘤动脉的形态,促进瘤体内血栓形成。同时保证了载瘤动脉及分支血管供血。本组 15.4% 个未破裂 VBDA 应用该技术,围手术期无并发症发生。19 个动脉瘤影像学随访,21.1% 治愈,57.9% 造影显示瘤腔内血栓形成,瘤腔缩小,10.5% 复发,复发病例均为单一支架置入。21 例临床随访患者,95.2% 预后良好,4.8% 术后因肺部感染病情加重死亡。该技术不足之处在于多重支架释放过程中可能出现的支架移位,支架无法完全释放的问题。

近年来出现的 Pipeline 是具有良好的血流导向性及高金属覆盖率的一类支架系统。其特性包括能够明显减少射入瘤腔内的血流,促进腔内血栓形成;更好的促进血管内膜的修复;保持载瘤动脉通畅,避免穿支血管的闭塞,治疗颅内夹层动脉瘤呈现出巨大优势。研究报道,Pipeline 治疗颅内前循环动脉瘤疗效显著优于后循环动脉瘤,治疗后循环动脉瘤及巨大动脉瘤术后并发症发生率明显增多[11]。应用血流导向装置治疗大型 VBDA,术后致残、致死率高于 70%[12],结果令人失望。然而也有研究证实,Pipeline 治疗大型后循环动脉瘤安全有效[13]。本组共 12% 个未破裂 VBDA 使用 Pipeline 治疗,术后 4.8% 例有脑缺血并发症发生。术后即刻造影,90.5% 个瘤体内对比剂滞留明显,9.5% 个动脉瘤未见显影。15 个动脉瘤行影像随访,26.7% 个完全闭塞,73.3% 个好转。17 例临床随访,100% 患者预后良好,疗效满意。本组经验是,Pipeline 能有效保护分支血管;选择的目标血管不能过于迂曲;选择足够长的支架以保证对夹层动脉瘤的完全覆盖并提供良好支撑力;支架导管可放置于健侧椎动脉中,减少对基底动脉的干扰;释放支架时应缓慢并反复拖曳,使其能充分打开保证贴壁良好。Pipeline 治疗未破裂 VBDA 应用前景良好,但本组病例数少,该技术治疗的安全性及有效性仍需要多中心、大样本病例研究来证实。

载瘤动脉闭塞是早期广泛应用于治疗 VBDA 的方法。在 6.9% 个动脉瘤栓塞 + 载瘤动脉闭塞病例中,围手术期 25.0% 出现并发症。术后影像学随访 100% 动脉瘤治愈,临床随访 100% 患者预后良好。此方法操作相对简单,围手术期无特殊处理,临床疗效满意,对于不适宜行血管重建方法的未破裂 VBDA 患者是一种选择[3]。但是该手术方式是一种破坏性的治疗方法,牺牲了正常的载瘤动脉,直接阻断了椎-基底动脉分支的供血,有造成脑干、脊髓或小脑缺血梗死及动脉瘤复发的潜在风险。本组病例显示,载瘤动脉闭塞手术围手术期并发症发生率明显高于其他手术组,并且术后存在缺血损伤的潜在风险,不建议首选此种手术方式。

参 考 文 献

[1] Wouter I, Sehievink. Spontaneous dissection of the carotid and vertebral arteries[J]. N Engl J Med, 2001, 344: 898-906. DOI: 10.1056/nejm200103223441206.

[2] Mizutxni T, Arugx T, Kirino T, et al. Recurrent subarachnoid hemorrhage from untreated ruptured vertebrobasilar dissecting aneurysms[J]. Neurosurgery, 1995, 3: 905-911. DOI: 10.1097/00006123-199505000-00003.

[3] Rabinov JD, Hellinger FR, Morris PP, et al. Endovascular management of vertebrobasilar dissecting aneurysms[J]. AJNR Am J Neuroradiol, 2003, 24(7): 1421-1428.

[4] Hosoya T, Adachi M, Yamaguchi K, et al. Clinical and neuroradiological fetures of intracranial vertebrobasilar artery dissection[J]. Stroke, 1999, 30(5): 1083-1090. DOI: 10.1161/01.str.30.5.1083.

[5] Kim BM, Kin SH, Kim DI, et al. Outcomes and prognostic factors of intracranial unruptured vertebrobasilar artery dissection[J]. Neurology, 2011, 76(20): 1735-1741.

[6] Jiang C, Li Q, Liu JM, et al. Endovascular treatment for the basilar artery dissection[J]. Cardiovasc Intervent Radiol, 2014, 37(3): 646-656. DOI: 10.1007/s00270-013-0737-5.

[7] Sikkema T, Uyttenboooqaart M, Eshqhi O, et al. Intracranial artery dissection[J]. Eur J NEurol, 2014, 21: 820-826.

[8] 吴中学,刘爱华,李佑祥,等. 支架技术在椎动脉梭型及夹层动脉瘤的应用[J]. 介入放射学杂志, 2004, S1: 18-20.

[9] Kim BM, Shin YS, Kim SH, et al. Incidence and risk factors of recurrence after endovascular treatment of intracranial vertebrobasilar dissecting aneurysms[J]. Stroke, 2011, 42(9): 2425. DOI: 10.1161/STROKEAHA.111.617381.

[10] 刘建民,张鑫,周晓平,等. 血管内支架结合电解可脱卸弹簧圈治疗犬颈动脉宽颈动脉瘤的实验研究[J]. 介入放射学杂志, 2004, 13(3): 258-262.

[11] Kallmes DF, Hanel R, Lopes D, et al. International retrospective study of the pipeline embolization device: a multicenter aneurysm treatment study[J]. AJNR, 2015, 36(1): 108-115. DOI: 10.3174/ajnr.a4111.

[12] Siddiqui AH, Abla AA, Kan P, et al. Panacea or problem: flow diverters in the treatment of symptomatic large or giant fusiform vertebrobasilar aneurysms[J]. Neurosurgery, 2012, 116: 1258-1266. DOI: 10.3171/2012.2.jns111942.

[13] Natarajan SK, Lin N, Soniq A, et al. The safety of Pipelin flow diversion in fusiform vertebrobasilar aneurysms: a consecutive case series with longer-term follow-up from a single US center[J]. Neuro-surgery, 2016, 125(1): 111-119.

(收稿日期:2018-03-01)

(本文编辑:刘小梅)

Q2xvdWRWZXJzaW9uLQo?
